# A higher‐level classification of the Pannonian and western Pontic steppe grasslands (Central and Eastern Europe)

**DOI:** 10.1111/avsc.12265

**Published:** 2016-09-16

**Authors:** Wolfgang Willner, Anna Kuzemko, Jürgen Dengler, Milan Chytrý, Norbert Bauer, Thomas Becker, Claudia Biţă‐Nicolae, Zoltán Botta‐Dukát, Andraž Čarni, János Csiky, Ruzica Igić, Zygmunt Kącki, Iryna Korotchenko, Matthias Kropf, Mirjana Krstivojević‐Ćuk, Daniel Krstonošić, Tamás Rédei, Eszter Ruprecht, Luise Schratt‐Ehrendorfer, Yuri Semenishchenkov, Zvjezdana Stančić, Yulia Vashenyak, Denys Vynokurov, Monika Janišová

**Affiliations:** ^1^ Department of Botany and Biodiversity ResearchUniversity of ViennaRennweg 141030WienAustria; ^2^ Vienna Institute for Nature Conservation and AnalysesGießergasse 6/71090WienAustria; ^3^ National Dendrological Park “Sofiyvka” of NAS of UkraineKyivska 12a20300Uman'Ukraine; ^4^ Plant EcologyBayreuth Center for Ecology and Environmental Research (BayCEER)University of BayreuthUniversitätsstr. 3095447BayreuthGermany; ^5^ Synthesis Centre (sDiv)German Centre for Integrative Biodiversity Research (iDiv) Halle‐Jena‐LeipzigDeutscher Platz 5e04103LeipzigGermany; ^6^ Department of Botany and ZoologyMasaryk UniversityKotlářská 261137BrnoCzech Republic; ^7^ Department of BotanyHungarian Natural History MuseumBaross útca 131088BudapestHungary; ^8^ Department of Regional and Environmental Sciences/GeobotanyUniversity of TrierBehringstr. 2154296TrierGermany; ^9^ Institute of BiologyRomanian AcademySplaiul Independentei 296, s.6060031BucharestRomania; ^10^ MTA Centre for Ecological ResearchAlkotmány ú. 2‐42163VácrátótHungary; ^11^ Institute of BiologyScientific Research Center of the Slovenian Academy of Sciences and ArtsNovi trg 21001LjubljanaSlovenia; ^12^ Department of EcologyUniversity of PécsIfjúság útja 67624PécsHungary; ^13^ Department of Biology and EcologyUniversity of Novi SadTrg Dositeja Obradovića 221000Novi SadSerbia; ^14^ Department of Vegetation EcologyUniversity of WroclawKanonia 6/850‐328WroclawPoland; ^15^ M.G. Kholodny Institute of BotanyNAS of UkraineTereshchenkivska 201601KyivUkraine; ^16^ Institute for Integrative Nature Conservation ResearchUniversity of Natural Resources and Life SciencesGregor Mendel‐Str. 331180WienAustria; ^17^ Faculty of ForestryUniversity of ZagrebSvetošimunska 2510000ZagrebCroatia; ^18^ Faculty of Biology and GeologyBabeş‐Bolyai UniversityRepublicii 42400015Cluj‐NapocaRomania; ^19^ Department of BotanyBryansk State UniversityBezhitskaya 14241036BryanskRussia; ^20^ Faculty of Geotechnical EngineeringUniversity of ZagrebHallerova aleja 742000VaraždinCroatia; ^21^ State Inspection of Environmental ProtectionI. Franka 2/229010KhmelnytskyUkraine; ^22^ Institute of BotanySlovak Academy of SciencesĎumbierska 197411Banská BystricaSlovakia

**Keywords:** *Brometalia erecti*, Diagnostic species, Dry grassland, *Festucetalia valesiacae*, *Festuco‐Brometea*, *Galietalia veri*, Phytosociology, *Stipo‐Festucetalia pallentis*, Syntaxonomy, TWINSPAN, Vegetation‐plot database

## Abstract

**Questions:**

What are the main floristic patterns in the Pannonian and western Pontic steppe grasslands? What are the diagnostic species of the major subdivisions of the class *Festuco‐Brometea* (temperate Euro‐Siberian dry and semi‐dry grasslands)?

**Location:**

Carpathian Basin (E Austria, SE Czech Republic, Slovakia, Hungary, Romania, Slovenia, N Croatia and N Serbia), Ukraine, S Poland and the Bryansk region of W Russia.

**Methods:**

We applied a geographically stratified resampling to a large set of relevés containing at least one indicator species of steppe grasslands. The resulting data set of 17 993 relevés was classified using the TWINSPAN algorithm. We identified groups of clusters that corresponded to the class *Festuco‐Brometea*. After excluding relevés not belonging to our target class, we applied a consensus of three fidelity measures, also taking into account external knowledge, to establish the diagnostic species of the orders of the class. The original TWINSPAN divisions were revised on the basis of these diagnostic species.

**Results:**

The TWINSPAN classification revealed soil moisture as the most important environmental factor. Eight out of 16 TWINSPAN groups corresponded to *Festuco‐Brometea*. A total of 80, 32 and 58 species were accepted as diagnostic for the orders *Brometalia erecti*,* Festucetalia valesiacae* and *Stipo‐Festucetalia pallentis*, respectively. In the further subdivision of the orders, soil conditions, geographic distribution and altitude could be identified as factors driving the major floristic patterns.

**Conclusions:**

We propose the following classification of the *Festuco‐Brometea* in our study area: (1) *Brometalia erecti* (semi‐dry grasslands) with *Scabioso ochroleucae‐Poion angustifoliae* (steppe meadows of the forest zone of E Europe) and *Cirsio‐Brachypodion pinnati* (meadow steppes on deep soils in the forest‐steppe zone of E Central and E Europe); (2) *Festucetalia valesiacae* (grass steppes) with *Festucion valesiacae* (grass steppes on less developed soils in the forest‐steppe zone of E Central and E Europe) and *Stipion lessingianae* (grass steppes in the steppe zone); (3) *Stipo‐Festucetalia pallentis* (rocky steppes) with *Asplenio septentrionalis‐Festucion pallentis* (rocky steppes on siliceous and intermediate soils), *Bromo‐Festucion pallentis* (thermophilous rocky steppes on calcareous soils), *Diantho‐Seslerion* (dealpine *Sesleria caerulea* grasslands of the Western Carpathians) and *Seslerion rigidae* (dealpine *Sesleria rigida* grasslands of the Romanian Carpathians).

**Nomenclature**
Euro+Med PlantBase for vascular plants (www.emplantbase.org, accessed Apr 2014), Flora Europaea (Tutin et al. 2001) for vascular plants not covered by the previous source; Grolle & Long (2000) and Hill et al. (2006) for bryophytes; Liška et al. (2008) for lichens, supplemented by LIAS (http://liasnames.lias.net/, accessed Dec 2015) for taxa not included in the previous source; EuroVegChecklist (Mucina et al. 2016) for phytosociological classes, orders and alliances unless stated otherwise 

## Introduction

The Pannonian steppe grasslands of the Carpathian Basin represent a western outpost of the Pontic steppes of E Europe (Walter 1974; Bohn & Neuhäusl 2000–2003). However, most steppe grasslands both in E Europe and in the Carpathian Basin have been ploughed and transformed into arable fields, especially during the last century (Molnár et al. 2008). The remaining sites are refuges for many rare species of plants and animals, and significantly contribute to European biodiversity (Poschlod & WallisDeVries 2002; Hobohm 2005). The semi‐dry grasslands (“meadow steppes”) of this region even hold the world records for vascular plant species richness at spatial scales between 10 and 50 m², in some regions exceeding 100 vascular plant species on plot sizes of 25 m² (Dengler et al. 2012; Merunková et al. 2012; Wilson et al. 2012; Chytrý et al. 2015).

Because of the strong decline of steppe grasslands in recent decades, many of them are listed as priority habitat types in the Habitats Directive of the European Union, e.g. ‘6240 Sub‐Pannonic steppic grasslands’, ‘6250 Pannonic loess steppic grasslands’ and ‘62C0 Ponto‐Sarmatic steppes’ (European Commission 2013). These habitat types were defined on the basis of expert judgement, partly reflecting traditional phytosociological units. However, both their effective conservation and large‐scale biogeographic comparison are impeded by the fact that no consistent classification of the grassland types of Europe is available so far. Grasslands mapped as the same habitat type may refer to floristically different units in different EU member states, while identical communities may be labelled as different habitat types (Evans 2010). Therefore, evaluations of the conservation status of certain habitat types at the European level are likely biased. Comparisons between grasslands of the European Union, on the one hand, and Ukraine and Russia, on the other hand, are even more difficult since vegetation classification systems in these two parts of Europe have developed in relative isolation (Kuzemko et al. 2014).

During the last two decades new tools and methods for large‐scale classification have been developed (De Cáceres et al. 2015), and grassland classifications based on large vegetation‐plot databases have been achieved in several countries or regions (e.g. Chytrý 2007; Hegedüšová Vantarová & Škodová 2014). However, very few attempts have been made to bring idiosyncratic national classifications together into uniform supra‐national classifications (e.g. Illyés et al. 2007; Dúbravková et al. 2010; Terzi 2015). The present paper aims to fill this gap for the Pannonian and W Pontic steppe grasslands of Europe.

Specifically, we aim to: (1) summarize the main floristic patterns within the Pannonian and W Pontic grasslands of the class *Festuco‐Brometea*; (2) clarify the delimitation of this class with respect to other grassland classes, especially *Molinio‐Arrhenatheretea* and *Koelerio‐Corynephoretea*; and (3) determine the diagnostic species of the major subdivisions of *Festuco‐Brometea* within the study area.

## Methods

### Study area

**Figure 1:**
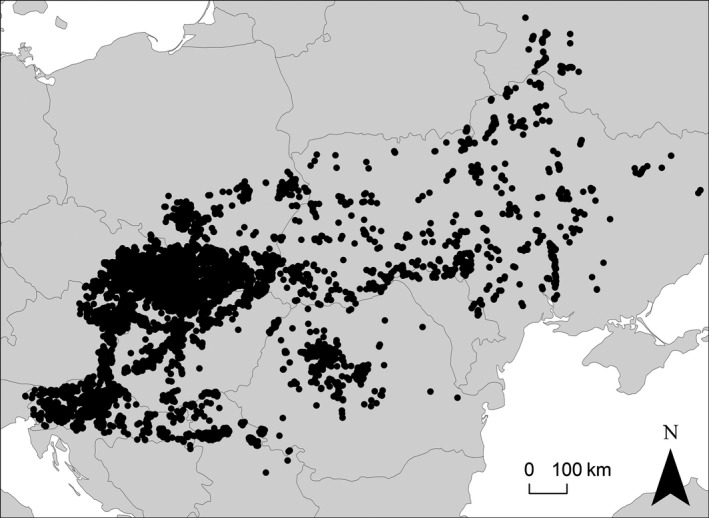
Study area and geographic distribution of the sample plots (after resampling).

The study area comprises the Carpathian Basin (including the surrounding hills and low mountains) and the SW part of E Europe (Fig. 1). The following countries are included: Hungary, E Austria, SE Czech Republic (Moravia), Slovakia, Romania, Slovenia, N Serbia (Vojvodina), N Croatia, Ukraine (except Crimea), S Poland and the Bryansk region in W Russia. The area has a temperate climate with relatively cold winters and a summer maximum of precipitation. Annual precipitation ranges mostly from 450 to 650 mm (Walter & Lieth 1967). The natural vegetation of this area is characterized by deciduous broad‐leaved forests (in the lowlands mostly thermophilous oak forests), forest‐steppes and steppes (Bohn & Neuhäusl 2000–2003). According to the phytogeographic classification of Meusel & Jäger (1992), the Carpathian Basin belongs to the Pannonian Floristic Province, while the E European part of our study area mostly corresponds to the West Pontic Floristic Province.

### Data set

As the first step, vegetation‐plot data of all kinds of grasslands were obtained from databases referenced in the *Global Index of Vegetation‐Plot Databases* (GIVD; Dengler et al. 2011; www.givd.info). These databases, together with their GIVD ID, were the Austrian Vegetation Database (EU‐AT‐001; Willner et al. 2012), the Czech National Phytosociological Database (EU‐CZ‐001; Chytrý & Rafajová 2003), the Phytosociological Database of Non‐forest Vegetation in Croatia (EU‐HR‐001; Stančić 2012), the Hungarian Phytosociological Database (EU‐HU‐003; Csiky et al. 2012), the Polish Vegetation Database (EU‐PL‐001; Kącki & Sliwinski 2012), the Romanian Grassland Database (EU‐RO‐008, formerly EU‐RO‐001 and EU‐RO‐002; Bita‐Nicolae 2012; Ruprecht et al. 2012), the Slovak Vegetation Database (EU‐SK‐001; Šibík 2012) and the Ukrainian Grassland Database (EU‐UA‐001; Kuzemko 2012). Additional unpublished data were contributed by co‐authors of this paper. All relevés were imported into JUICE 7.0 (Tichý 2002) for further data preparation and analysis. Species taxonomy and nomenclature was unified. Taxa recorded with different taxonomic precision were joined into aggregates or broad species concepts (see Appendix S1). Taxa determined only at the genus level were excluded.

From this initial data set, we selected all relevés with the presence of at least one indicator species of steppe grasslands. Indicator species were defined using published lists of character species as well as results of preliminary classifications of regional subsets of our data set (see Appendix S2 for details). Plots from altitudes above 1000 m or with a shrub or tree layer covering >10% were excluded. We further restricted the data set to relevés with a plot size between 10 and 100 m². To avoid biases due to oversampling of some areas, we applied a geographically stratified random resampling. We stratifed the study area into grid cells of 10 × 6 geographic minutes and randomly selected 30 plots from each grid cell. If a cell contained 30 plots or less, all of them were selected. However, a considerable number of plots from Hungary and Ukraine had no coordinates. To avoid losing these data, we stratified them arbitrarily by available header data, which seemed to be the best approximation of locality. After resampling, the data set included 17 993 plots and 3182 taxa (Fig. 1).

Prior to the analysis, we excluded all trees and tall shrubs as they are representatives of later successional stages after abandonment and are traditionally not considered for the classification of grasslands. We also excluded lichens and bryophytes at this stage because they were only recorded in a subset of the relevés (however, we returned them to the data set for characterization of clusters). Thus, the final data set used for classification included 2247 taxa of vascular plants.

### Classification and fidelity calculation

To identify the patterns of floristic variation, we applied the TWINSPAN algorithm using WinTWINS 2.3 (Hill & Šmilauer 2005). Cut levels of pseudospecies were set to 0%, 5% and 25% cover. Species with less than five occurences were excluded. The maximum number of division levels was six, and minimum group size for division was two plots. To evaluate the phytosociological class membership of the TWINSPAN clusters, we used the diagnostic species of classes as listed in the EuroVegChecklist (Mucina et al. 2016). For each relevé, we calculated the total cover of the diagnostic species of the following classes: *Festuco‐Brometea, Molinio‐Arrhenatheretea, Nardetea strictae, Koelerio‐Corynephoretea* and *Elyno‐Seslerietea*. Then we averaged the values for each class among the relevés of each cluster (see Appendix S3 for a full list of class species; note that some species are diagnostic for two classes). For the subsequent analyses, we only used those clusters where the *Festuco‐Brometea* species had the highest average total cover. Within these clusters, we excluded all relevés where the species of another class clearly prevailed (i.e. where the total cover of the species of another class was > 50% and at least 10% higher than the total cover of the *Festuco‐Brometea* species). No transformation of the cover values was applied. The values “r” and “+” of the Braun‐Blanquet scale were calculated as 1%; for other values the average percentage cover was used. Calculation was done in the JUICE program following the algorithm formally described by Fischer (2015).

In the “cleaned” *Festuco‐Brometea* data set, we merged adjacent clusters into larger groups that corresponded most closely to the concepts of phytosociological orders (*Brometalia erecti*,* Festucetalia valesiacae* and *Stipo‐Festucetalia pallentis*). Fidelity of species to orders was calculated using three different fidelity measures: phi coefficient of association (Chytrý et al. 2002; Tichý & Chytrý 2006), constancy ratio (Dengler 2003) and cover ratio (Willner et al. 2009). Cover ratio was only used for species exceeding an average cover of 5% in at least one order. The phi coefficient was calculated assuming equal group size, and positive phi values were only accepted if the difference in species constancy between the target order and the rest of the class was significant according to Fisher's exact test at *P *=* *0.05.

### Formal definition of vegetation units

Unlike many recent studies that apply the Cocktail method to establish formal definitions of vegetation units (e.g. Janišová & Dúbravková 2010; Rodríguez‐Rojo et al. 2014), we use diagnostic species for this purpose. Plots are assigned to the unit with the highest total cover of diagnostic species. This approach is usually applied in a hierchical manner, starting with the assignment to a class, and then successively proceeding to the lower ranks (Willner 2011). Advantages of this approach are that (1) one does not need *a‐priori* diagnostic species groups that are valid for all vegetation classes, and (2) almost all plots can be assigned to one and only one vegetation unit. However, both approaches involve subjective decisions such as the fidelity measure or the applied fidelity threshold. Therefore, the diagnostic species determined by any statistical procedure are just an approximation of the complex reality, dependent on the data set and various subjective choices.

While for the class level we relied on expert‐based diagnostic species lists (Appendix S3), we used a consensus of the three fidelity measures mentioned above to establish the diagnostic species of the orders. Species reaching a phi coefficient of 0.2, a constancy ratio of 2 (i.e. a constancy at least twice as high as in the unit with the second highest constancy value) and a cover ratio of 2 were automatically accepted as diagnostic. Species reaching only one or two of these thresholds were evaluated individually and only accepted if their diagnostic value was supported by external knowledge (either by published references or by our own field experience). Moreover, we only considered species with a constancy of at least 5% in one of the orders. The ecological requirements of the accepted diagnostic species of the orders were compared using the indicator values of Borhidi (1995). We tried to distinguish between differential and character species, but this distinction did not influence the following step of re‐assignment.

### Re‐assignment of relevés

To improve the consistency between the TWINSPAN‐based groups and our formal definitions of the orders, we adjusted the order assignment of the relevés using the total cover of diagnostic species (see Luther‐Mosebach et al. 2012 for a similar approach). Relevés showing a mismatch between the total cover of diagnostic species and the initial, TWINSPAN‐based order assignment were manually re‐assigned (note that this step is similar to the “refined ordination” of the TWINSPAN algorithm). To account for the limited accuracy of cover values, a tolerance of 10% was applied. Thus, a relevé was only re‐assigned to another order if the total cover value of that order was at least 10% higher than the total cover value of the initial one. This adjusted data set was used for the description and interpretation of the internal variability of each order. Within orders, the original TWINSPAN cluster membership of the relevés was preserved.

## Results

### TWINSPAN classification of the total data set

The TWINSPAN table roughly follows a moisture gradient ranging from wet meadows on the left to dry rocky grasslands on the right (Appendix S4). Semi‐dry grasslands are situated in the centre of the table. At the 4th level of division, the following vegetation types were identified: (1) intermittently wet, nutrient‐poor meadows; (2) permanently wet meadows; (3) steppic meadows on rarely flooded river terraces in Eastern Europe; (4) temporarily wet alluvial meadows; (5) grasslands on nutrient‐poor, acidic soils; (6) nutrient‐rich mesic meadows and pastures; (7) floristically impoverished semi‐dry grasslands; (8) species‐rich semi‐dry grasslands on moderately acidic soils; (9) semi‐dry grasslands on calcareous soils; (10) grass steppes; (11) sand steppes; (12) pioneer grasslands on sandy soils; (13) rocky grasslands on calcareous and siliceous bedrock; (14) calcareous rocky grasslands rich in sub‐mediterranean species; (15) dealpine rocky grasslands of the Western Carpathians; (16) dealpine rocky grasslands of the Romanian Carpathians.

According to the average total cover of diagnostic species of classes, groups 1, 2, 4 and 6 belong to the class *Molinio‐Arrhenatheretea*, group 5 to the *Nardetea strictae*, groups 7–10 and 13–16 to the *Festuco‐Brometea* and groups 11–12 to the *Koelerio‐Corynephoretea*. Group 3 is transitional between the *Molinio‐Arrhenatheretea* and the *Koelerio‐Corynephoretea*. The average total covers of class species as well as a preliminary syntaxonomic interpretation of all clusters at the 6th level of division is given in Appendix S5.

### *Diagnostic species of the* Festuco‐Brometea *orders*

Within the *Festuco‐Brometea*, groups 7–9 correspond to the order *Brometalia erecti*, group 10 to the *Festucetalia valesiacae* and groups 13–16 to the *Stipo‐Festucetalia pallentis*. In the *Brometalia erecti*, 66 species reached both the threshold of phi coefficient and constancy ratio. Eight species reached only the phi threshold and 32 species only the constancy ratio threshold. For the *Festucetalia valesiacae*, the respective values are 19 (both), four (only phi) and 31 (only constancy ratio), and for the *Stipo‐Festucetalia pallentis* 51 (both), one (only phi) and 16 (only constancy ratio) (Appendix S6). Seven species had an average cover of ≥5% in at least one order. Six of them (*Brachypodium pinnatum* agg., *Bromus erectus*,* Festuca valesiaca*,* Festuca pallens* agg., *Sesleria caerulea* and *Carex humilis*) reached the threshold of all three fidelity measures, while *Festuca stricta* subsp. *sulcata* only reached the phi and constancy ratio threshold but not the cover ratio threshold. Thus, 135 species were automatically accepted as diagnostic (as they reached the threshold of all three fidelity measures), while 93 species were individually evaluated. Finally, 170 species were accepted as diagnostic (80 species for the *Brometalia erecti*, 32 species for the *Festucetalia valesiacae* and 58 species for the *Stipo‐Festucetalia pallentis*). Phi, constancy and average cover values of all species as well as reasons for accepting or rejecting a species as diagnostic are given in Appendix S6.

**Figure 2:**
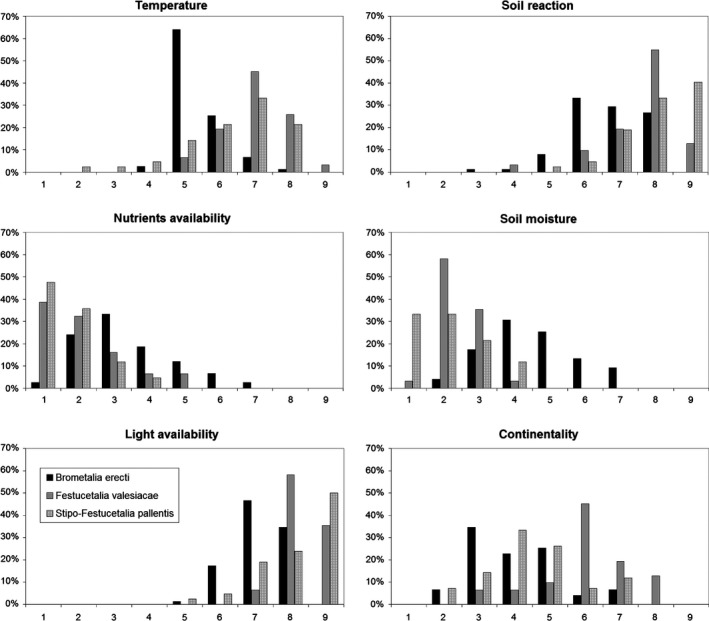
Relative frequency distribution of Borhidi indicator values among the accepted diagnostic species of the orders *Brometalia erecti*,* Festucetalia valesiacae* and *Stipo‐Festucetalia pallentis*.

*Brometalia* species tend to have lower indicator values for temperature, soil reaction, light and continentality, but higher values for nutrients and soil moisture (Fig. 2). Diagnostic species of the *Festucetalia valesiacae* have the highest temperature and continentality values, while for soil reaction, light, nutrients and soil moisture they are intermediate between the two other orders. *Stipo‐Festucetalia* species tend to have the highest values for soil reaction and light and the lowest values for nutrients and soil moisture.

### Internal variability of the orders

Re‐assignment of relevés among the orders led to a revised classification of the *Festuco‐Brometea* (Table 1, Appendices S7, S8). While the TWINSPAN groups corresponding to the *Brometalia erecti* and *Stipo‐Festucetalia pallentis* largely remained unchanged, a considerable portion of group 10 (corresponding to the *Festucetalia valesiacae*) was moved to one of the two other orders (Table 2).

**Table 1:** Synoptic table of the *Festuco‐Brometea*

Group Number	B1	B2	B3	B4	B5	B6	B7	F1	F2	F3	F4	S1	S2	S3	S4	S5	S6	S7
Twinspan Cluster Level 4	7	7	8	8	9	9	10	10	10	10	10	10	13	13	14	14	15	16
Twinspan Cluster Level 5–6	0	1	0	1	0	1		0‐0	0‐1	1‐0	1‐1		0	1	0	1	0	1
No. of Relevés	301	135	994	235	846	765	760	1324	730	1316	177	709	65	690	174	250	192	66
***Brometalia erecti***																		
*Brachypodium pinnatum* agg.	15	8	62	86	68	82	15	4	10	1	1	6	.	6	3	.	5	.
*Briza media*	20	21	78	94	43	38	6	1	1	1	.	2	.	4	1	.	4	.
*Dactylis glomerata*	52	24	65	68	34	45	24	6	6	16	.	1	.	1	.	.	1	.
*Bromus erectus*	15	.	42	94	38	17	23	6	3	1	.	5	.	3	3	5	.	.
*Centaurea jacea*	30	58	44	74	21	19	13	2	2	8	.	1	.	1	.	.	.	.
*Leontodon hispidus*	38	25	67	47	32	19	15	5	5	10	.	7	.	3	1	.	5	.
*Knautia arvensis*	35	53	48	35	35	38	16	4	6	15	2	2	.	2	.	.	11	.
*Lotus corniculatus*	60	39	73	91	48	27	33	15	15	23	3	13	.	6	4	5	23	.
*Arrhenatherum elatius*	47	12	51	42	31	23	23	14	1	4	.	8	2	1	.	.	1	.
*Leucanthemum vulgare* agg.	35	52	62	76	21	21	7	1	1	3	.	2	.	10	3	.	39	6
*Plantago media*	57	38	73	74	53	56	32	10	34	38	15	16	.	8	2	.	7	2
*Trifolium montanum*	10	27	54	72	28	36	11	4	7	13	.	4	.	1	.	.	.	.
*Salvia pratensis*	32	46	56	85	53	64	28	18	20	22	.	15	.	14	5	4	3	.
*Pimpinella saxifraga* agg.	44	59	63	63	57	40	40	24	18	17	2	25	.	16	7	1	19	6
*Trifolium pratense*	54	55	50	53	6	4	10	2	1	12	.	1	.	1	.	.	2	.
*Linum catharticum*	6	6	51	81	35	14	7	2	2	3	2	5	.	11	2	.	23	6
*Anthoxanthum odoratum* agg.	33	4	46	41	4	6	11	4	1	1	.	3	.	1	.	.	.	.
*Thymus pulegioides*	24	58	45	14	26	1	15	5	1	2	.	4	3	4	.	.	5	2
*Centaurea scabiosa*	15	24	38	49	59	66	20	11	22	18	6	9	.	15	13	2	7	.
*Festuca rubra* agg.	16	24	39	12	10	4	9	2	.	1	.	1	.	1	.	.	1	.
*Betonica officinalis*	9	4	29	34	8	24	5	1	1	3	1	1	.	1	.	.	1	.
*Plantago lanceolata*	68	44	70	86	29	18	37	23	18	37	23	13	.	2	.	.	2	.
*Vicia cracca*	13	37	33	37	9	7	5	1	1	3	.	1	.	1	.	.	9	.
*Carex caryophyllea*	12	2	35	68	28	13	14	9	5	3	.	6	.	1	2	1	.	.
*Ranunculus polyanthemos* agg.	34	22	38	23	18	33	14	2	4	19	.	3	.	1	1	1	3	.
*Carlina acaulis*	4	4	44	46	28	10	6	2	1	1	.	4	.	6	5	.	24	2
*Trisetum flavescens*	15	.	37	25	3	1	2	1	1	.	.	1	.	.	.	.	.	.
*Carex flacca*	4	2	21	76	18	4	1	1	.	.	.	1	.	1	.	.	2	.
*Agrostis capillaris*	30	19	35	13	8	5	12	4	1	2	.	1	.	1	.	.	.	.
*Festuca pratensis*	32	37	36	10	5	10	6	1	1	5	.	1	.	.	.	.	1	.
*Daucus carota*	43	59	31	41	19	7	21	4	3	18	3	4	.	1	.	.	.	.
*Veronica chamaedrys*	34	21	34	13	10	6	9	2	1	5	.	2	.	2	.	.	5	6
*Cirsium pannonicum*	1	.	20	47	8	16	1	.	1	1	.	.	.	2	.	.	1	.
*Cruciata glabra*	2	2	34	34	7	8	1	1	1	1	.	1	.	1	.	.	5	2
*Prunella vulgaris*	20	24	27	17	6	7	4	1	1	3	.	1	.	.	.	.	1	.
*Ononis spinosa*	18	2	28	9	16	12	9	3	2	6	1	1	.	.	.	.	.	.
*Tragopogon pratensis*	13	5	37	28	12	7	3	2	6	1	.	2	.	1	1	.	2	.
*Campanula glomerata*	2	1	26	11	14	24	3	1	2	1	.	2	.	2	1	.	3	.
*Luzula campestris* agg.	21	.	34	25	4	4	9	3	.	1	1	3	.	.	.	.	.	3
*Polygala comosa*	10	31	23	60	16	5	6	1	3	4	2	2	.	1	.	.	.	2
*Ranunculus bulbosus*	13	1	23	50	9	2	3	2	1	1	.	1	.	1	1	.	.	.
*Filipendula vulgaris*	22	18	39	36	12	55	14	6	21	18	2	12	.	1	.	3	.	.
*Carex montana*	.	.	22	34	7	9	2	1	1	1	.	1	.	1	.	.	2	.
*Avenula pubescens*	8	3	26	20	7	14	5	2	1	1	.	2	.	1	1	.	.	.
*Viola hirta*	16	1	42	27	27	28	8	3	14	4	1	10	.	10	2	.	34	.
*Koeleria pyramidata*	2	.	9	79	11	2	2	1	1	.	.	1	.	1	.	.	3	.
*Rhinanthus minor*	10	3	25	10	5	3	4	2	1	1	.	1	.	1	.	.	1	.
*Primula veris*	3	1	34	.	8	16	4	1	1	1	.	2	.	7	.	.	1	17
*Campanula patula*	9	2	24	6	1	1	2	1	.	1	.	1	.	1	.	.	.	.
*Prunella grandiflora*	3	1	12	32	15	18	2	1	1	1	.	1	.	3	1	.	4	.
*Rumex acetosa*	13	13	26	11	1	4	3	2	1	2	.	1	.	1	1	.	.	.
*Carex tomentosa*	4	.	17	.	6	12	1	1	1	1	.	1	.	.	1	.	.	.
*Lathyrus pratensis*	12	6	18	19	2	1	1	1	.	1	.	.	.	.	.	.	2	.
*Colchicum autumnale*	6	.	22	3	2	1	1	1	1	.	.	1	.	.	.	.	.	.
*Potentilla erecta*	1	1	18	33	1	1	.	1	1	1	.	1	.	.	.	.	.	.
*Seseli annuum*	6	19	11	3	23	20	9	5	3	3	1	4	.	1	1	.	.	.
*Peucedanum cervaria*	2	.	9	10	11	41	5	2	5	1	.	3	.	6	.	1	3	.
*Taraxacum sect. Ruderalia*	28	24	27	1	5	6	7	2	1	8	1	2	.	1	1	.	5	.
*Danthonia alpina*	1	.	4	41	3	8	1	1	1	.	.	.	.	.	.	.	.	.
*Buphthalmum salicifolium*	1	1	5	79	13	1	1	.	.	.	.	1	.	3	.	.	13	.
*Potentilla heptaphylla*	15	1	35	1	22	5	9	3	1	2	.	1	2	17	11	.	7	2
*Carlina vulgaris* agg.	10	22	16	16	28	17	14	4	6	8	.	7	.	6	5	1	9	3
*Ranunculus acris*	5	19	17	8	1	2	1	1	.	1	.	1	.	.	.	.	1	.
*Thymus longicaulis*	1	.	1	69	3	1	.	.	.	.	.	.	.	.	.	.	.	.
*Clinopodium vulgare*	17	4	15	9	12	10	7	3	5	3	1	3	.	1	.	.	8	.
*Euphorbia verrucosa*	.	.	2	55	6	1	1	.	.	.	.	.	.	.	.	.	.	.
*Cerastium fontanum subsp. vulgare*	22	13	24	3	3	2	7	2	.	7	1	2	.	1	.	.	2	.
*Prunella laciniata*	13	.	10	26	7	5	5	2	1	2	.	1	.	1	.	.	.	.
*Carex michelii*	1	.	7	.	9	21	3	2	2	2	1	1	.	1	.	.	.	.
*Onobrychis viciifolia* agg.	3	3	17	20	15	33	7	2	16	9	8	7	.	1	1	.	.	.
*Polygala vulgaris*	3	1	17	25	5	2	3	1	1	3	.	1	.	1	.	.	2	2
*Alchemilla spec.div*.	2	1	18	4	1	1	1	.	1	.	.	1	.	.	.	.	2	.
*Polygala major*	1	.	10	.	9	21	1	1	3	1	.	3	.	3	1	.	.	.
*Tanacetum corymbosum*	1	1	12	5	8	24	2	2	2	1	.	2	.	5	1	.	6	2
*Trifolium medium*	10	5	20	9	5	5	3	1	1	10	.	1	.	.	.	.	1	.
*Inula salicina*	4	1	10	1	4	8	1	1	1	1	.	1	.	1	.	.	.	.
*Gymnadenia conopsea*	1	.	7	31	4	2	.	1	1	.	.	.	.	2	.	.	5	.
*Hypochaeris maculata*	1	.	9	23	6	13	2	1	2	7	4	1	.	1	.	.	.	.
*Linum flavum*	1	4	1	1	10	19	4	1	5	1	1	3	.	5	.	.	2	2
*Aster amellus*	1	2	1	2	15	26	3	2	11	4	1	4	.	6	1	.	1	9
***Festucetalia valesiacae***																		
*Festuca valesiaca*	25	1	6	1	9	19	30	54	49	68	76	27	2	7	7	10	.	.
*Eryngium campestre*	27	.	6	.	20	27	44	50	60	54	55	32	.	1	3	4	.	.
*Thymus pannonicus* agg.	20	1	10	1	23	36	34	50	65	49	15	32	3	7	6	6	1	3
*Bothriochloa ischaemum*	9	.	1	2	9	13	16	34	71	24	31	26	.	7	21	4	.	.
*Salvia nemorosa*	4	.	1	.	2	10	10	5	27	35	48	5	.	1	.	.	1	.
*Stipa capillata*	.	.	.	.	1	5	8	19	50	17	55	23	.	5	18	8	.	.
*Koeleria macrantha*	15	3	11	.	19	22	29	52	42	31	40	39	.	11	19	2	.	5
*Artemisia austriaca*	1	.	.	.	.	.	1	3	2	18	29	1	.	.	.	.	.	.
*Euphorbia nicaeensis*	.	.	1	.	1	16	5	2	19	22	58	3	.	1	1	3	.	.
*Potentilla argentea*	31	36	3	.	1	1	16	23	4	27	5	5	6	1	.	.	.	.
*Falcaria vulgaris*	5	2	2	.	6	13	12	9	27	24	12	7	.	.	1	.	.	.
*Astragalus onobrychis*	1	.	1	.	5	11	10	10	30	18	27	9	.	1	3	.	.	.
*Salvia nutans*	1	.	1	.	1	4	2	1	23	9	62	6	.	.	.	.	.	.
*Carex praecox*	6	1	1	.	1	2	5	6	5	17	2	1	.	1	1	.	.	.
*Centaurea stoebe*	10	.	3	1	16	14	27	49	42	13	1	32	20	11	21	7	1	2
*Verbascum phoeniceum*	3	.	1	.	1	6	5	10	18	10	8	6	.	1	.	1	.	.
*Trifolium arvense*	11	7	1	.	1	1	9	23	1	11	2	9	.	1	1	1	.	.
*Nonea pulla*	2	12	1	.	2	7	7	3	18	17	9	4	.	.	.	.	.	.
*Chondrilla juncea*	2	.	.	.	1	1	2	10	3	8	2	3	.	1	2	.	.	.
*Cleistogenes serotina*	.	.	.	.	.	.	1	6	19	5	7	7	.	1	2	1	.	.
*Taraxacum serotinum*	.	.	.	.	1	2	4	2	10	9	21	1	.	1	.	.	.	.
*Astragalus austriacus*	1	.	.	.	1	5	4	2	25	9	27	6	.	1	1	.	.	.
*Achillea nobilis*	3	.	1	.	1	1	5	11	1	10	13	3	.	1	.	.	.	.
*Berteroa incana*	2	7	.	.	1	1	5	8	2	11	2	2	2	1	.	.	.	.
*Viola ambigua*	1	.	1	.	1	10	3	1	13	13	40	3	.	1	1	1	.	.
*Artemisia campestris*	1	40	1	.	4	5	10	25	25	14	6	21	18	8	9	1	1	.
*Cytisus austriacus*	2	.	1	.	3	19	6	4	18	16	12	5	.	1	.	.	.	.
*Linum austriacum*	1	.	.	.	1	2	4	3	10	5	25	3	.	1	.	1	.	.
*Veronica prostrata*	4	.	2	.	2	3	7	15	10	5	1	8	.	2	2	.	.	.
*Iris pumila*	.	.	.	.	1	1	1	6	13	3	26	8	.	1	4	1	.	3
*Linaria genistifolia*	.	.	.	.	1	2	4	16	14	4	5	13	25	4	8	3	.	9
*Melica transsilvanica*	.	1	.	.	2	2	4	9	3	5	8	6	3	2	.	.	5	.
***Stipo‐Festucetalia pallentis***																		
*Festuca pallens* agg.	.	1	.	.	2	1	2	6	1	.	.	27	34	58	84	70	28	2
*Carex humilis*	1	3	3	10	17	36	13	15	53	2	1	65	.	66	76	87	29	5
*Teucrium montanum*	.	.	1	12	7	5	8	6	32	5	5	33	2	54	79	69	22	8
*Jovibarba globifera*	.	.	.	.	2	1	1	5	.	1	.	14	42	34	47	18	49	3
*Thymus praecox*	1	.	2	.	7	1	6	15	2	1	.	25	5	31	90	81	4	.
*Anthericum ramosum*	1	.	9	18	23	35	5	6	10	2	2	29	2	55	56	40	40	17
*Sesleria caerulea*	.	.	1	1	2	1	1	1	1	.	.	2	2	32	13	.	86	.
*Asplenium ruta‐muraria*	.	.	.	.	1	.	1	2	1	.	.	6	18	27	10	4	42	73
*Helianthemum canum*	.	.	1	.	1	1	1	1	2	.	.	9	.	19	48	47	1	8
*Leontodon incanus*	1	.	.	8	4	.	.	1	1	.	.	2	.	26	54	8	41	.
*Fumana procumbens*	.	.	.	.	.	.	1	1	1	.	.	6	.	4	58	72	.	.
*Allium lusitanicum*	.	.	1	.	4	2	2	5	1	1	.	15	34	28	25	12	18	.
*Seseli osseum*	1	.	.	.	1	3	7	23	6	1	.	28	28	38	47	6	31	11
*Sedum album*	.	.	1	.	1	1	1	6	.	.	.	11	18	24	32	14	23	.
*Genista pilosa*	1	.	1	.	6	1	3	3	1	.	.	14	5	29	32	5	13	.
*Vincetoxicum hirundinaria*	2	4	3	.	10	18	9	5	9	8	1	17	5	46	16	20	48	36
*Scorzonera austriaca*	.	.	1	.	1	2	1	1	2	.	.	5	.	9	40	62	.	.
*Globularia bisnagarica*	.	.	1	39	13	3	2	3	2	.	.	11	.	17	51	61	2	.
*Stipa eriocaulis*	.	.	.	.	1	1	1	1	2	1	.	5	2	1	12	75	.	.
*Asplenium trichomanes*	.	.	.	.	1	.	1	2	.	.	.	4	35	11	.	1	21	62
*Pulsatilla halleri* subsp. *slavica*	.	.	.	.	1	.	.	.	.	.	.	1	.	13	1	.	53	.
*Phyteuma orbiculare*	.	.	3	2	1	1	.	.	.	.	.	.	.	14	7	2	56	21
*Melica ciliata*	.	.	.	.	1	1	3	13	9	1	.	19	25	20	30	8	4	33
*Hornungia petraea*	1	.	.	.	.	.	.	1	1	.	.	2	2	2	10	53	.	.
*Polygonatum odoratum*	.	.	2	3	4	8	2	1	1	.	.	6	.	23	6	9	25	.
*Poa badensis*	.	.	.	.	.	1	1	3	1	.	.	7	.	8	29	20	.	5
*Biscutella laevigata*	.	.	1	1	3	1	1	1	.	.	.	2	.	17	16	4	11	12
*Saxifraga paniculata*	.	.	.	.	.	.	.	1	.	.	.	1	5	9	.	.	30	50
*Alyssum montanum*	.	.	1	.	2	1	2	7	2	1	.	12	.	12	43	13	.	2
*Arabidopsis arenosa*	1	1	1	.	2	1	2	3	1	1	.	4	22	17	2	2	19	26
*Clinopodium alpinum*	.	.	1	6	3	.	.	1	1	.	.	2	.	17	3	.	28	8
*Campanula rotundifolia*	1	13	3	.	10	8	5	2	1	1	.	8	32	21	22	6	15	14
*Thymus comosus*	.	.	.	.	1	1	1	2	1	.	.	5	2	14	1	.	.	56
*Bromus pannonicus*	.	.	2	.	1	1	.	1	.	.	.	4	.	15	6	2	2	.
*Linum tenuifolium*	.	.	1	2	8	7	4	3	13	1	21	11	.	14	52	31	.	.
*Minuartia setacea*	.	.	.	.	.	.	.	3	2	1	2	6	8	5	19	25	.	3
*Polygala amara* agg.	1	.	3	1	1	.	1	.	1	.	.	1	.	12	3	2	23	11
*Erysimum odoratum*	.	1	1	.	3	5	2	5	3	1	.	8	.	23	4	1	4	27
*Minuartia laricifolia*	.	.	.	.	.	.	.	.	.	.	.	1	.	5	2	.	38	.
*Galium pusillum* agg.	2	1	7	1	2	.	1	1	.	.	.	3	.	10	5	.	50	.
*Seseli leucospermum*	.	.	.	.	.	.	.	.	.	.	.	1	2	2	13	28	.	.
*Erysimum witmannii*	.	.	1	.	1	.	.	.	.	.	.	1	.	6	.	.	31	.
*Hieracium bupleuroides*	.	.	.	.	.	.	.	.	.	.	.	.	.	5	.	.	35	.
*Carduus defloratus*	.	.	1	.	1	.	.	1	.	.	.	1	.	5	.	.	45	3
*Cyanus triumfettii*	1	.	2	.	4	7	2	3	2	.	.	4	.	20	3	2	13	15
*Kernera saxatilis*	.	.	.	.	.	.	.	.	.	.	.	.	.	5	1	.	30	9
*Dianthus praecox*	.	.	.	.	1	.	.	1	.	.	.	1	.	5	16	1	20	.
*Draba lasiocarpa*	.	.	.	.	.	.	.	.	.	.	.	1	2	4	23	9	.	6
*Sesleria rigida*	.	.	.	.	.	.	.	.	.	.	.	1	.	5	.	.	.	82
*Scabiosa lucida*	.	.	1	.	1	.	.	.	.	.	.	.	.	2	.	.	41	2
*Dianthus plumarius*	.	.	.	.	.	.	.	.	.	.	.	1	.	2	5	27	1	.
*Thymus pulcherrimus*	1	.	1	.	.	.	.	.	.	.	.	1	.	2	1	.	40	.
*Thesium alpinum*	.	.	1	1	1	.	.	.	.	.	.	.	.	3	2	.	38	.
*Primula auricula*	.	.	.	.	.	.	.	.	.	.	.	.	.	3	.	.	32	.
*Helictotrichon decorum*	1	.	1	.	1	.	.	1	1	.	.	1	.	6	.	.	.	59
*Paronychia cephalotes*	.	.	.	.	.	.	.	1	1	.	5	1	.	1	3	28	.	2
*Pulsatilla vulgaris*	1	.	1	1	7	11	3	4	4	1	.	8	2	16	13	4	1	5
*Scabiosa canescens*	1	.	1	.	3	4	2	3	3	1	.	5	.	4	17	10	.	.

Values are percentage constancy. Assignment of relevés to orders was done according to the total cover of diagnostic species. Internal classification of the orders follows the original TWINSPAN clusters. A few small clusters have been omitted. Diagnostic species of orders are sorted by descending fidelity to the order (calculated as the phi coefficient). A long version of this table showing all species including bryophytes and lichens can be found in Appendix S7. Data sources are listed in Appendix S8.

**Table 2:** Basic figures on the re‐assignment of relevés according to the total cover of diagnostic species. Relevés that did not change their position are given in bold.

Initial assignment	Re‐assigned to	No. of rel.	%
*Brometalia erecti*	*Brometalia erecti*	**3276**	**94%**
*Brometalia erecti*	*Festucetalia valesiacae*	38	1%
*Brometalia erecti*	*Stipo‐Festucetalia pallentis*	156	4%
*Festucetalia valesiacae*	*Brometalia erecti*	760	15%
*Festucetalia valesiacae*	*Festucetalia valesiacae*	**3547**	**71%**
*Festucetalia valesiacae*	*Stipo‐Festucetalia pallentis*	709	14%
*Stipo‐Festucetalia pallentis*	*Brometalia erecti*	10	1%
*Stipo‐Festucetalia pallentis*	*Festucetalia valesiacae*	3	0%
*Stipo‐Festucetalia pallentis*	*Stipo‐Festucetalia pallentis*	**1459**	**99%**

#### *Brometalia erecti* (Table 1: B1–B7, Fig. 3)

**Figure 3:**
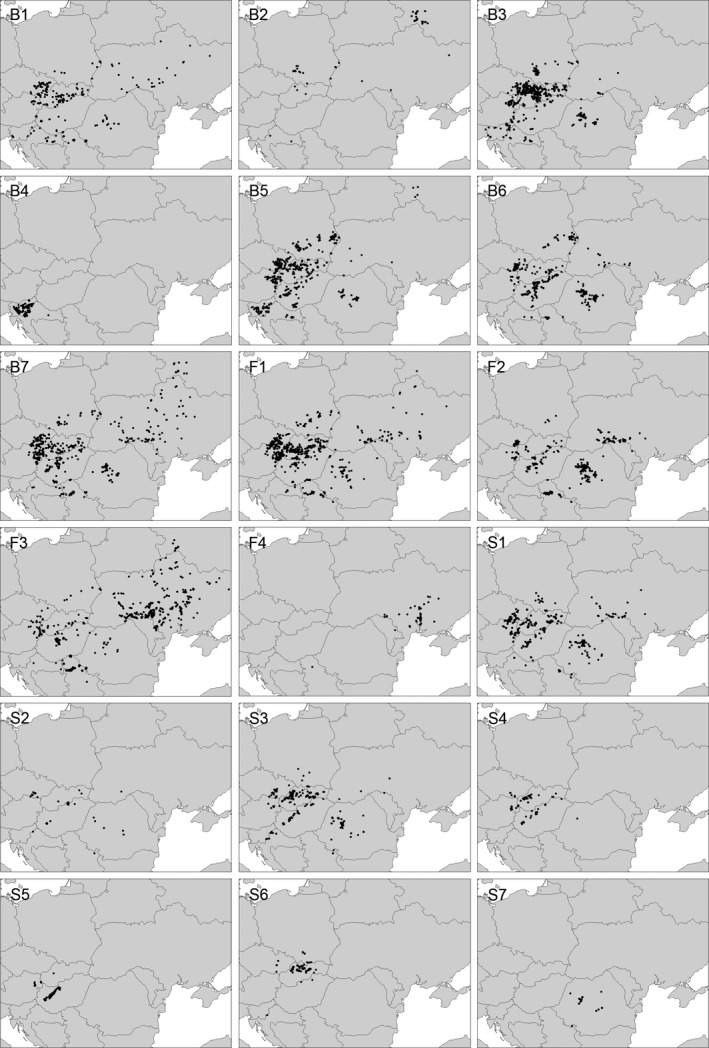
Distribution of the TWINSPAN groups within the orders *Brometalia erecti*,* Festucetalia valesiacae* and *Stipo‐Festucetalia pallentis*.

The further subdivision of the *Brometalia erecti* mainly reflects differences in soil conditions and to a lesser degree also the geographic distribution. Group B1 includes semi‐dry grasslands in floodplains as well as on acidic soils. Both of these subtypes are mostly dominated by *Festuca stricta* subsp. *sulcata*, while *Bromus erectus* is almost absent. Group B2 is mainly restricted to the forest zone of Eastern Europe where several more southerly distributed species are absent. The next two groups include rather mesic, often very species‐rich grasslands on moderately acidic soils. This type is especially widespread in the Carpathians (group B3) and in the northwestern Dinaric mountains (group B4). Groups B5–B7 comprise more xeric grasslands on calcareous soils. Species of the *Festucetalia valesiacae* and *Stipo‐Festucetalia pallentis* are more frequent in this type than in other units of the *Brometalia erecti*. This kind of semi‐dry grassland is widespread throughout the study area and also includes the meadow steppes in the forest‐steppe zone of Ukraine.

#### *Festucetalia valesiacae* (Table 1: F1–F4, Fig. 3)

The grass steppes of the *Festucetalia valesiacae* are subdivided into geographic groups. Although there is considerable overlap in the distribution of the four TWINSPAN clusters, they can be linked to the northwest (group F1), southwest (group F2), northeast (group F3) and southeast (group F4) of the study area. The last group is the most distinct one, with Pontic species such as *Salvia nutans, Phlomis pungens* and *Stipa lessingiana* reaching a high constancy. It is the only type of the studied grasslands that is distributed within the true steppe zone.

#### *Stipo‐Festucetalia pallentis* (Table 1: S1–S7, Fig. 3)

The *Stipo‐Festucetalia pallentis* show the most complex pattern of the three orders. Group S1 includes communities transitional to the *Festucetalia valesiacae*. *Festuca pallens* agg. is mostly replaced by *Festuca stricta* subsp. *sulcata* in this group. Rocky grasslands on siliceous soils are included in group S2, which lacks many calciphilous diagnostic species of the order. Groups S3–S5 represent typical calcareous rocky grasslands. Groups S4 and S5 are rich in sub‐mediterranean species such as *Helianthemum canum* and *Fumana procumbens*. Submontane and montane grasslands with many dealpine species are included in groups S6 and S7. Group S6 is usually dominated by *Sesleria caerulea* and occurs mainly in the Western Carpathians. Group S7 is dominated by *Sesleria rigida* and occurs in the Romanian Carpathians.

## Discussion

### Syntaxonomy

In the classic system of the *Festuco‐Brometea*, all communities of the eastern part of Europe were included in the order *Festucetalia valesiacae*, while all of sub‐atlantic Europe were grouped within the *Brometalia erecti*, in both cases irrespective of soil properties (e.g. Royer 1991). In recent decades, however, most authors argue for a subdivision of the class reflecting the soil conditions at the level of orders (e.g. Korneck 1974; Rodwell et al. 2002; Dengler et al. 2012). This shift in the concept of orders was mainly based on the observation that the semi‐dry grasslands of sub‐atlantic and subcontinental Europe are floristically more similar to each other than to the other types of dry grasslands within each region (Korneck 1974; Mucina et al. 2009). We follow this concept by classifying the main types of steppe grasslands in our study area as orders. Three of them (the *Brometalia erecti*, the *Festucetalia valesiacae* and the *Stipo‐Festucetalia pallentis*) belong to the *Festuco‐Brometea*, while the sandy steppes of the *Festucetalia vaginatae*, in accordance with Dengler (2003) and Mucina et al. (2016), are considered as part of the *Koelerio‐Corynephoretea* (see also Appendix S5).

In contrast to the order level, the identification of our TWINSPAN groups with earlier described alliances is less straightforward. Thus, the following considerations are only preliminary and more detailed studies on the subdivision of each order are needed.

### The order *Brometalia erecti*

The core of the *Brometalia erecti* was divided into a rather mesic, moderately acidic subgroup (columns B3–B4 in Table 1) and a more xeric, calcareous subgroup (columns B5–B6). These two units correspond to the alliances *Bromion erecti* and *Cirsio‐Brachypodion pinnati* in national vegetation surveys, respectively (e.g. Chytrý 2007; Hegedüšová Vantarová & Škodová 2014). However, Willner et al. (2013) argued that the semi‐dry grasslands included here in group B3 contain a large number of species with eastern distribution (e.g. *Festuca stricta* subsp. *sulcata, Filipendula vulgaris, Cirsium pannonicum*), while the character species of the sub‐atlantic *Bromion erecti* are almost absent. Therefore, it seems more appropriate to classify this unit in the *Cirsio‐Brachypodion*, perhaps as a separate suballiance. The *Danthonio‐Stipion stenophyllae*, which was described for slightly acidic semi‐dry grasslands of Transylvania, also formed part of group B3 and should be included in the *Cirsio‐Brachypodion* (see also Dengler et al. 2012). Group B4 corresponds to the association *Bromo‐Danthonietum*, which was included in the *Mesobromion* (= *Bromion erecti*) by Kaligarič & Škornik (2002). It is transitional between the alliances *Bromion erecti, Cirsio‐Brachypodion* and *Scorzonerion villosae* (Terzi 2015), so its syntaxonomic position needs further investigation.

The semi‐dry grasslands of group B1 represent a type that has been completely neglected in all national surveys of the Pannonian region so far. It probably also belongs to the *Cirsio‐Brachypodion*. Some stands in Romania and northern Serbia are transitional towards the Balkanic alliance *Chrysopogono‐Danthonion* (Pedashenko et al. 2013; Aćić et al. 2015).

The syntaxonomic position of the meadow steppes of Eastern Europe varies considerably among authors. In Russia, they are usually included in the *Festucion valesiacae* (e.g. Poluyanov & Averinova 2012). In Ukraine, Korotchenko & Didukh (1997) described a separate alliance *Fragario viridis‐Trifolion montani* within the *Festucetalia valesiacae*. Kuzemko et al. (2014) re‐introduced the forgotten name *Agrostio‐Avenulion schellianae*, coined by Royer (1991), for the same unit and transferred it to the *Brometalia*. Our results support the inclusion of Eastern European meadow steppes in the *Brometalia*. However, there is hardly any support for a separate alliance (see Fig. 3: group B7), so we propose to include them in the *Cirsio‐Brachypodion*. Accordingly, the distribution range of this alliance is more or less the same as that of the *Festucion valesiacae*, which replaces the *Cirsio‐Brachypodion* on less developed soils (see below).

The most obvious discrepancy between our results and current syntaxonomic concepts concerns group B2, which includes semi‐dry grasslands of Eastern Europe north of the forest‐steppe zone. These so‐called “steppe meadows” are known as *Scabioso ochroleucae‐Poion angustifoliae* and have traditionally been classified within the order *Galietalia veri*, class *Molinio‐Arrhenatheretea* (Bulokhov 2001; Ermakov 2012). However, both the TWINSPAN results and the cover of diagnostic species clearly embed the *Scabioso ochroleucae‐Poion angustifoliae* in the *Festuco‐Brometea*. Thus, we consider it as a northern vicariant of the *Cirsio‐Brachypodion* that is connected to the forest zone of Eastern Europe. However, the exact delimitation of this unit, in particular with respect to the *Filipendulo vulgaris‐Helictotrichion pratensis* of northern and northern Central Europe (Dengler et al. 2003) requires further studies.

### The order *Festucetalia valesiacae*

Within the *Festucetalia valesiacae*, group F4 is clearly distinct from the rest of the order by the presence of several Pontic species. This unit can probably be identified with the *Stipion lessingianae*, which is usually understood as a southeastern vicariant of the *Festucion valesiacae* (Kuzemko et al. 2014). From a larger biogeographic perspective, this unit might even be regarded as the core of the *Festucetalia valesiacae* as it represents the zonal vegetation of the steppe zone, while the *Festucion valesiacae* (groups F1–F3) includes extrazonal grass steppes within the forest‐steppe zone. As already mentioned, this concept deviates from the current Russian literature (Ermakov 2012) where also the meadow steppes on deep soils are included in the *Festucion valesiacae*. However, the precise delimitation between the *Festucion valesiacae* and *Stipion lessingianae* remains a task for more detailed studies. In particular, it will depend on this delimitation whether the southeastern unit can retain the name *Stipion lessingianae*. The type association of this alliance, which is from Transylvania (Romania), was included in group F2 in our TWINSPAN classification. If this result is confirmed, the name *Stipion lessingianae* would fall into the synonymy of the *Festucion valesiacae* and the W Pontic zonal steppes would obtain the name *Stipo lessingianae‐Salvion nutantis* (Vynokurov 2014).

### The order *Stipo‐Festucetalia pallentis*

The *Stipo‐Festucetalia pallentis* comprise azonal open steppes restricted to shallow soils on hard bedrock. The two most important environmental factors shaping the internal variability within this order are soil chemistry and temperature. Rocky grasslands on siliceous and intermediate soils (groups S1 p.p., S2) are not very common in our study area. They have only a few diagnostic species, while many species typical for calcareous bedrock are absent. Despite this floristic poverty, silicolous rocky grasslands have been split into several alliances (Mucina et al. 2016). The *Alysso‐Festucion pallentis* is mainly distributed in the Bohemian Massif (Chytrý 2007), therefore it is only marginally represented in our study area. Most rocky grasslands of the study area bound to acidic and intermediate rocks correspond to the *Asplenio septentrionalis‐Festucion pallentis*. However, the concept of this alliance is in need of a revision. Hegedüšová Vantarová & Škodová (2014) consider it as a synonym of the *Festucion valesiacae*. In our analysis, the closed *Festuca pseudodalmatica* grasslands, classified by Borhidi (1996) within the *Asplenio septentrionalis‐Festucion pallentis*, were included in group F1 while the more open types dominated by the same species, as well as the *Festuca pallens* communities on siliceous soils, were clearly assigned to the *Stipo‐Festucetalia pallentis*.

Two main groups of calcareous rocky grasslands can be distinguished: a thermophilous one, rich in sub‐mediterranean species (groups S3–S5), and a submontane‐montane one, characterized by dealpine species (groups S6–S7). Although this basic pattern is well recognized in the phytosociological literature (e.g. Janišová & Dúbravková 2010), the precise delimitation of higher syntaxa along this gradient is still under debate. In the light of our results at least three alliances seem to be well supported: (1) thermophilous rocky grasslands mostly dominated by *Festuca pallens* agg. (groups S1 p.p., S3–S5; *Bromo‐Festucion pallentis* incl. *Diantho‐Seslerion* p.p.); (2) dealpine *Sesleria caerulea* grasslands of the Western Carpathians (group S6; *Diantho‐Seslerion* incl. *Astero alpini‐Seslerion* p.p.); and (3) dealpine *Sesleria rigida* grasslands of the Romanian Carpathians (group S7; *Seslerion rigidae*).

There are two more alliances that were proposed based on data from rather small areas and without sufficient comparison with units from neighbouring regions. The *Chrysopogono‐Festucion dalmaticae* includes grasslands dominated by *Festuca dalmatica* or *Bromus pannonicus* on calcareous outcrops in southern Hungary (Borhidi 1996). The *Bromus pannonicus* communities were included in group S1 but the *Festuca dalmatica* grasslands mostly remained in group F1 because they contain hardly any diagnostic species of the *Stipo‐Festucetalia pallentis*. However, these relevés might be misclassified as they are mostly dominated by species that are extremely rare in our data set (*Festuca dalmatica, Artemisia alba, Trinia glauca, Galium lucidum*). At least some of these species seem to have their optimum in rocky grasslands of southern Europe (Terzi 2015). Thus, a comparison with data from the Balkan Peninsula is necessary to further evaluate the syntaxonomic position of this alliance.

The *Chrysopogono‐Festucion pseudodalmaticae* was described from serpentine rocks in southwestern Romania, just at the edge of our study area (Coldea 2012). The core associations of this alliance mostly correspond to group S2 (the *Asplenio septentrionalis‐Festucion pallentis*), and indeed they are floristically and environmentally very similar to this alliance. However, as in the case of the *Chrysopogono‐Festucion dalmaticae* numerous Balkanic species are present in these grasslands, and so it might well be that they belong to a southern vicariant of the Pannonian alliance, which should be more widespread south of our study area.

### Preliminary syntaxonomic synopsis

In conclusion, we propose the following classification of the Pannonian and western Pontic steppe grasslands (excluding those on sandy soils):

#### Festuco‐Brometea

*Brometalia erecti* (syn. *Brachypodietalia pinnati*)

- *Scabioso ochroleucae‐Poion angustifoliae* – steppe meadows of the forest zone of E Europe.
- *Cirsio‐Brachypodion pinnati* (incl. *Danthonio‐Stipion stenophyllae, Fragario viridis‐Trifolion montani, Agrostio‐Avenulion schellianae*) – meadow steppes on developed soils in the forest‐steppe zone of E Central and E Europe.

#### Festucetalia valesiacae

- *Festucion valesiacae* – grass steppes on less developed soils in the forest‐steppe zone of E Central and E Europe.
- *Stipion lessingianae* (incl. *Stipo lessingianae‐Salvion nutantis*) – grass steppes in the steppe zone.

#### Stipo‐Festucetalia pallentis

- *Alysso‐Festucion pallentis* – rocky steppes of the Bohemian Massif
- *Asplenio septentrionalis‐Festucion pallentis* – rocky steppes on intermediate and siliceous soils of the Pannonian region
- *Bromo‐Festucion pallentis* (syn. *Seslerio‐Festucion pallentis* p.p.) – thermophilous rocky steppes on calcareous soils of the Pannonian region
- *Diantho‐Seslerion* (syn. *Seslerio‐Festucion pallentis* p.p.) – dealpine *Sesleria caerulea* grasslands of the Western Carpathians
- *Seslerion rigidae* – dealpine *Sesleria rigida* grasslands of the Romanian Carpathians

### Methodological issues

It is often overlooked that the Braun‐Blanquet approach involves not only a classification of communities, but also the simultaneous classification of communities and species. Diagnostic species can be used to characterize vegetation units, but they can also be used as tools for assignment of plots to these units. Therefore, groups of diagnostic species can be used as a kind of formal definitions of vegetation units. Unfortunately, numerical fidelity measures such as constancy ratio or the phi coefficient have similar drawbacks as the commonly used unsupervised classification methods (see Tichý et al. 2014). Their results are considerably dependent on the data set used and the algorithms and parameters chosen (Willner et al. 2009). Thus, transferability of diagnostic species from one study to another is limited.

In this paper, we introduced a supervised consensus approach to identification of diagnostic species, which involved three numerical fidelity measures as well as external expert knowledge. A special category of diagnostic species which we did not use in our study is “shared diagnostic species”, i.e. species differentiating two (or more) units against all others. For example, *Galium verum, Securigera varia* and *Medicago falcata* seem to differentiate the *Brometalia* and *Festucetalia valesiacae* against the *Stipo‐Festucetalia pallentis* (Appendix S6). Defining this kind of diagnostic species is particularly challenging as the fidelity thresholds need to be adjusted for them. However, in certain cases it might be important to include shared diagnostic species in the formal definition of vegetation units, especially in less species‐rich plant communities.

### Conclusions and outlook

We established diagnostic species groups for the three main types of Pannonian and western Pontic steppe grasslands, corresponding to the phytosociological orders *Brometalia erecti, Festucetalia valesiacae* and *Stipo‐Festucetalia pallentis*. This is an important step towards unifying classification schemes developed in different parts of the study area. The subdivision of the orders into alliances could only be drafted in a rather preliminary way, as it would require a great deal of additional analyses to develop a robust classification at the lower levels of the Braun‐Blanquet system. However, our formal definitions of the orders will provide a firm basis for these subsequent classification exercises, as they allow for a straightforward selection of data sets representing individual *Festuco‐Brometea* orders. Moreover, we hope that our study may serve as a template for other projects focusing on the broad‐scale revision of vegetation classes.

## Author contribution

M.J., A.K. and W.W. prepared the data set; W.W. did the analyses and led the writing; M.C., J.D., M.J. and A.K. provided substantial input to the first drafts, and all authors critically revised the manuscript.

## Supporting information


**Appendix S1.** List of aggregated species.

**Appendix S2.** Indicator species of steppe grasslands.

**Appendix S3.** Diagnostic species of the classes *Festuco‐Brometea, Molinio‐Arrhenatheretea, Nardetea strictae, Koelerio‐Corynephoretea* and *Elyno‐Seslerietea* following the EuroVegChecklist.

**Appendix S4.** Synoptic table of the TWINSPAN classification.

**Appendix S5.** Average percentage cover of the diagnostic species of five grassland classes in the TWINSPAN clusters.

**Appendix S6.** Fidelity, constancy and average cover of species in the three *Festuco‐Brometea* orders.

**Appendix S7.** Full synoptic table of the *Festuco‐Brometea*.

**Appendix S8.** Data sources of the *Festuco‐Brometea* relevés.
