# Supplementary material for: A higher‐level classification of the Pannonian and western Pontic steppe grasslands (Central and Eastern Europe)
Source: Appl Veg Sci. 2016 Sep 16;20(1):143–58. doi: 10.1111/avsc.12265 (PMC5348766; doi:10.1111/avsc.12265)
Supplement: Supplementary file 1 — Appendix S1. List of aggregated species. [file AVSC-20-143-s001.pdf]

Supporting information to the paper

Willner, W. et al. A higher-level classification of the Pannonian and western Pontic steppe grasslands (Central and Eastern Europe). *Applied Vegetation Science.*

Appendix S1. List of aggregated species. Species concepts and nomenclature of vascular plants follow Euro+Med PlantBase (www.emplantbase.org, accessed in April 2014) or, for taxa not covered by the previous source, Flora Europaea (Tutin et al. 2001). Species concepts and nomenclature of bryophytes follow Grolle & Long (2000) and Hill et al. (2006). Species concepts and nomenclature of lichens follow Liška et al. (2008), supplemented by LIAS (http://liasnames.lias.net/, accessed in December 2015) for taxa not included in this source.

Vascular plants

Achillea millefolium agg.: *A. aspleniifolia, A. carpatica, A. collina, A. distans, A. macrophylla, A. millefolium, A. pannonica, A. pratensis, A. roseoalba, A. setacea, A. styriaca*
Alchemilla spec. div.: all species of the genus *Alchemilla*
Anthoxanthum odoratum agg.: *A. alpinum, A. odoratum*
Arabis hirsuta agg.: *A. hirsuta, A. planisiliqua, A. sagittata, A. sudetica*
Arenaria serpyllifolia agg.: *A. leptoclados, A. serpyllifolia, A. uralensis*
Brachypodium pinnatum agg.: *B. pinnatum, B. rupestre*
Callitriche palustris agg.: *C. cophocarpa, C. palustris*
Camelina sativa agg.: *C. microcarpa, C. satica*
Cardamine pratensis agg.: *C. dentata, C. matthioli, C. pratensis*
Carex flava agg.: *C. demissa, C. flava, C. lepidocarpa, C. viridula*
Carex muricata agg.: *C. divulsa, C. muricata, C. spicata*
Carex vulpina agg.: *C. cuprina, C. vulpina*
Carlina vulgaris agg.: *C. biebersteinii, C. vulgaris*
Centaurea arenaria agg.: *C. arenaria, C. borysthenica*
Centaurea jacea agg.: *C. jacea, C. subjacea*
Dianthus carthusianorum agg.: *D. carthusianorum, D. diutinus, D. giganteiformis, D. giganteus, D. pontederae*
Dorycnium pentaphyllum agg.: *D. germanicum, D. herbaceum*
Eleocharis palustris agg.: *E. mamillata, E. palustris*
Erysimum hieraciifolium agg.: *E. hieraciifolium, E. hungaricum, E. marschallianum*
Euphrasia nemorosa agg.: *E. coerulea, E. micrantha, E. nemorosa*
Euphrasia rostkoviana agg.: *E. kerneri, E. picta, E. rostkoviana*
Euphrasia stricta agg.: *E. pectinata, E. slovaca, E. stricta*
Festuca ovina agg.: *F. filiformis, F. ovina* (incl. *F. guestfalica*)
Festuca pallens agg.: *F. csikhegyensis, F. pallens*
Festuca rubra agg.: *F. heteromalla, F. nigrescens, F. rubra*
Galium boreale agg.: *G. boreale, G. ruboides*
Galium mollugo agg.: *G. album, G. mollugo*
Galium palustre agg.: *G. elongatum, G. palustre*
Galium pusillum agg.: *G. anisophyllon, G. austriacum, G. pumilum, G. sudeticum, G. valdepilosum*
Glechoma hederacea agg.: *G. hederacea, G. hirsuta*
Hylotelephium maximum agg.: *H. maximum, H. telephium*
Chenopodium album agg.: *C. album, C. strictum*
Juncus bufonius agg.: *J. bufonius, J. ranarius*
Leucanthemum vulgare agg.: *L. ircutianum, L. vulgare*
Lotus corniculatus agg.: *L. alpinus, L. borbasii, L. corniculatus, L. ucrainicus*
Luzula campestris agg.: *L. campestris, L. divulgata, L. multiflora*
Minuartia verna agg.: *M. glaucina, M. verna*
Molinia caerulea agg.: *M. arundinacea, M. caerulea*
Myosotis scorpioides agg.: *M. nemorosa, M. scorpioides*
Myosotis sylvatica agg.: *M. stenophylla, M. sylvatica*
Odontites vulgaris agg.: *O. vernus, O. vulgaris*
Oenothera biennis agg.: *O. biennis, O. rubricaulis* etc.
Onobrychis viciifolia agg.: *O. arenaria, O. montana, O. viciifolia*
Ornithogalum pyrenaicum agg: *O. pyrenaicum, O. sphaerocarpum*
Phleum pratense agg.: *P. nodosum, P. pratense*
Pimpinella saxifraga agg.: *P. nigra, P. saxifraga*
Poa pratensis agg.: *P. angustifolia, P. pratensis* (incl. *P. humilis*)
Polygala amara agg.: *P. amara, P. amarella*
Polygonum aviculare agg.: *P. arenastum, P. aviculare, P. rurivagum*
Potentilla collina agg.: *P. collina, P. thyrsiflora*
Potentilla incana agg.: *P. incana, P. tommasiniana*
Potentilla pusilla agg.: *P. pusilla, P. tabernaemontani*
Pulmonaria officinalis agg.: *P. obscura, P. officinalis*
Ranunculus auricomus agg.: *R. auricomus, R. cassubicus* etc.
Ranunculus polyanthemos agg.: *R. polyanthemos* (incl. *R. polyanthemophyllus*), *R. serpens* (incl. *R. nemorosus*)
Scleranthus annuus agg.: *S. annuus, S. polycarpos, S. verticillatus*
Senecio nemorensis agg.: *S. hercynicus, S. nemorensis, S. ovatus*
Silene otites agg.: *S. borysthenica, S. densiflora, S. donetzica, S. exaltata, S. hellmannii, S. otites, S. wolgensis*
Soldanella montana agg.: *S. hungarica, S. montana*
Spergula pentandra agg.: *S. morisonii, S. pentandra*
Symphyotrichum novi-belgii agg.: *S. lanceolatum, S. novi-belgii*
Thymus pannonicus agg.: *T. kosteleckyanus, T. odoratissimus*
Verbascum thapsus agg.: *V. densiflorum, V. thapsus*
Veronica hederifolia agg.: *V. hederifolia, V. sublobata, V. triloba*

Bryophytes
Bryum atrovirens agg.: *B. klinggraefii, B. rubens, B. ruderale, B. sauteri, B. subapiculatum, B. tenuisetum, B. violaceum*
Hedwigia ciliata agg.: *H. ciliata, H. stellata*
Plagiomnium affine agg.: *P. affine, P. curvatulum, P. elatum, P. ellipticum, P. medium*
Racomitrium canescens agg.: *R. canescens, R.elongatum, R. ericoides*
Schistidium apocarpum agg.: *S. apocarpum, S. brunnescens* etc.
Scorpidium revolvens agg.: *S. cossonii, S. revolvens*
Syntrichia ruralis agg.: *S. calcicola, S. ruralis* (incl. *S. ruraliformis*)
Weissia spec. div.: all species of the genus *Weissia*

Lichens
Cladona arbuscula agg.: *C. arbuscula*, *C. mitis*
Cladonia furcata agg: *C. furcata*, *C. subrangiformis*
Collema spec. div.: all species of the genus *Collema*

References

Grolle, R. & Long, D.G. 2000. An annotated check-list of the *Hepaticae* and *Anthocerotae* of Europe and Macaronesia. *Journal of Bryology* 22: 103–140.
Hill, M.O., Bell, N., Bruggeman-Nannenga, M.A., Brugués, M., Cano, M.J., Enroth, J., Flatberg, K.K., Frahm, J.-P., Gallego, M.T., (...) & Söderström, L. 2006. An annotated ckecklist of the mosses of Europe and Macaronesia. *Journal of Bryology* 28: 198–267.
Liška, J., Palice, Z. & Slavíková, Š. 2008. Checklist and Red List of lichens of the Czech Republic. *Preslia* 80: 151–182.
Tutin, T.G., Heywood, V.H., Burges, N.A., Valentine, D.H., Walters, S.M. & Webb, D.A. (eds.) 2001. *Flora Europaea (5 volume set and CD-ROM pack)*. Cambridge University Press, Cambridge, UK.
