# Supplementary material for: A higher‐level classification of the Pannonian and western Pontic steppe grasslands (Central and Eastern Europe)
Source: Appl Veg Sci. 2016 Sep 16;20(1):143–58. doi: 10.1111/avsc.12265 (PMC5348766; doi:10.1111/avsc.12265)
Supplement: Supplementary file 2 — Appendix S2. Indicator species of steppe grasslands. [file AVSC-20-143-s002.pdf]

Supporting information to the paper

Willner, W. et al. A higher-level classification of the Pannonian and western Pontic steppe grasslands (Central and Eastern Europe). *Applied Vegetation Science.*

Appendix S2. Indicator species of steppe grasslands.

This list of steppe indicator species was prepared for selecting relevés with floristic affinity to steppes from a much larger grassland data set. It was intended as a starting point for the analysis and should not be confused with diagnostic species of any phytosociological unit.

A first list of indicator species was derived using two regional data sets from the western and eastern part of the study area, which were preliminarily classified at the alliance level: (a) 2993 grassland relevés from the forest zone and forest-steppe zone of Ukraine (A. Kuzemko, ined.), and (b) 2755 grassland relevés from the Pannonian region of Austria (Willner et al. 2013). Within each data set, we calculated the phi value of the association between each species and each alliance using the JUICE software (phi values based on presence-absence data, size of all groups standardized to equal size). In both data sets we selected the species with a phi value ≥ 0.3 in at least one of the following alliances: *Agrostion vinealis, Fragario viridis-Trifolion montani, Cirsio-Brachypodion* (s.lat. = incl. *Bromion erecti* auct.)*, Festucion valesiacae* and *Seslerio-Festucion pallentis*. This initial list was complemented with *Inula oculus-christi, Ranunculus illyricus, Chrysopogon gryllus* and *Stipa tirsa* from the diagnostic species listed for the *Festucetalia valesiacae* by Borhidi (1996). We also added *Trifolium montanum, Cirsium pannonicum, Filipendula vulgaris, Hypochaeris maculata* and *Galium verum*, which are usually considered as diagnostic for dry or semi-dry grasslands. Moreover, we used a species list prepared by M. Janišová (ined.) for the selection of *Stipo-Festucetalia pallentis* relevés from a larger data set. For the Pannonian sand steppes, we used the species listed as diagnostic for the *Festucion vaginatae* by Borhidi (1996).

Achillea nobilis
Achillea ochroleuca
Adonis vernalis
Agrimonia eupatoria
Agrostis vinealis
Allium flavum
Allium lusitanicum (= A. montanum)
Alyssum montanum
Anthericum ramosum
Anthyllis vulneraria
Artemisia campestris
Asperula cynanchica
Aster amellus
Astragalus austriacus
Astragalus dasyanthus
Astragalus onobrychis
Asyneuma canescens
Bothriochloa ischaemum
Brachypodium pinnatum agg.
Bromus erectus
Bromus inermis
Bromus pannonicus
Bupleurum falcatum
Campanula sibirica
Carex caryophyllea
Carex humilis
Carex praecox
Centaurea diffusa
Centaurea scabiosa
Centaurea stoebe
Chamaecytisus austriacus
Chrysopogon gryllus
Cleistogenes serotina
Clinopodium acinos (= Acinos arvensis)
Clinopodium alpinum (= Acinos alpinus)
Dianthus borbasii
Dianthus carthusianorum
Dianthus diutinus
Dianthus pontederae
Dianthus serotinus
Eryngium campestre
Erysimum odoratum
Euphorbia cyparissias
Euphorbia nicaeensis s.lat. *(*incl. *E. stepposa, E. pannonica)*
Euphorbia seguierana
Falcaria vulgaris
Festuca dalmatica
Festuca pallens s.lat. *(*incl. *F. csikhegyensis)*
Festuca pseudodalmatica
Festuca stricta subsp. *sulcata (= F. rupicola)*
Festuca stricta subsp. *stricta*
Festuca vaginata
Festuca valesiaca
Festuca wagneri
Fragaria viridis
Fumana procumbens
Galatella villosa (= Aster oleifolius)

Galium humifusum
Galium octonarium
Genista pilosa
Globularia bisnagarica
Gypsophila paniculata
Helianthemum canum
Helianthemum nummularium agg.
Helichrysum arenarium
Helictotrichon decorum
Hieracium cymosum (= Pilosella cymosa)
Hieracium virosum
Hypericum elegans
Inula ensifolia
Inula oculus-christi
Jovibarba globifera
Knautia arvensis
Koeleria macrantha (= Koeleria cristata auct.*)*
Leontodon incanus
Linum austriacum
Linum hirsutum
Linum tenuifolium
Medicago falcata
Melica ciliata
Minuartia setacea
Muscari comosum (= Leopoldia comosa)
Nigella arvensis
Onobrychis viciifolia agg.
Oxytropis pilosa
Petrorhagia saxifraga
Phleum phleoides
Pimpinella saxifraga
*Plantago media (*incl. *Plantago urvillei)*
Poa angustifolia
Poa badensis
Poa pannonica
Polygala amara agg.
Polygala sibirica
Polygonatum odoratum
Potentilla incana agg.
*Pulsatilla vulgaris (*incl. *P. grandis)*
Pulsatilla halleri
Ranunculus illyricus
Salvia nemorosa
Salvia nutans
Salvia pratensis
Salvia verticillata
Scabiosa canescens
Scabiosa ochroleuca
Scorzonera austriaca
Securigera varia (= Coronilla varia)
Sedum album
Senecio jacobaea
Seseli annuum
Seseli hippomarathrum
Seseli osseum
Seseli tortuosum
Sesleria caerulea
Sesleria heuflerana
Sesleria sadlerana
Sideritis montana
Stachys recta
Stipa borysthenica
Stipa capillata
Stipa eriocaulis
Stipa lessingiana
Stipa pennata s.str. *(= S. joannis)*
Stipa pulcherrima
Stipa tirsa
Taraxacum serotinum
Teucrium chamaedrys
Teucrium montanum
Teucrium polium
Thesium alpinum
Thesium arvense (= T. ramosum)
Thymus comosus
Thymus pannonicus agg.
Thymus praecox agg.
Trifolium medium
Verbascum phoeniceum
*Veronica austriaca (*incl. *V. jacquinii)*

Veronica prostrata
Vinca herbacea
Viola ambigua
Viola hirta

References

Borhidi, A. 1996. An annotated checklist of the Hungarian plant communities I. The non-forest vegetation. In: Borhidi, A. (ed.) *Critical revision of the Hungarian plant communities*, pp. 43–94. Janus Pannonius University, Pécs, HU.

Willner, W., Sauberer, N., Staudinger, M. & Schratt-Ehrendorfer, L. 2013. Syntaxonomic revision of the Pannonian grasslands of Austria – Part I: introduction and general overview. *Tuexenia* 33: 399–420.
