# Supplementary material for: A higher‐level classification of the Pannonian and western Pontic steppe grasslands (Central and Eastern Europe)
Source: Appl Veg Sci. 2016 Sep 16;20(1):143–58. doi: 10.1111/avsc.12265 (PMC5348766; doi:10.1111/avsc.12265)
Supplement: Supplementary file 3 — Appendix S3. Diagnostic species of the classes Festuco‐Brometea, Molinio‐Arrhenatheretea, Nardetea strictae, Koelerio‐Corynephoretea and Elyno‐Seslerietea following the EuroVegChecklist. [file AVSC-20-143-s003.pdf]

Supporting information to the paper

Willner, W. et al. A higher-level classification of the Pannonian and western Pontic steppe grasslands (Central and Eastern Europe). *Applied Vegetation Science.*

Appendix S3. Diagnostic species of the classes *Festuco-Brometea, Molinio-Arrhenatheretea, Nardetea strictae, Koelerio-Corynephoretea* and *Elyno-Seslerietea* following Mucina et al. (2016), with minor modifications. Note that a species can be diagnostic for more than one class. In the following list, this is only mentioned if a species occurs in two classes treated in this paper.

Festuco-Brometea:

Achillea coarctata
Achillea nobilis
Adonis vernalis
Adonis volgensis
Agropyron pectinatum
Ajuga genevensis
Ajuga laxmannii
Allium atropurpureum
Allium carinatum
Allium denudatum
Allium flavum
Allium fuscum
Allium lusitanicum
Allium moschatum
Allium oleraceum
Allium regelianum
Allium rotundum
Allium sphaerocephalon
Allium strictum
Allium vineale
Althaea cannabina
Alyssum montanum
Alyssum murale
Alyssum repens
Alyssum rostratum
Anacamptis coriophora
Anacamptis morio
Anacamptis pyramidalis
Androsace koso-poljanskii
Anthericum liliago
Anthericum ramosum
Anthyllis vulneraria
Aquilegia transsilvanica
Arabis hirsuta agg.
Arenaria cephalotes
Arenaria longifolia
Arenaria procera
Artemisia alba
Artemisia austriaca
Artemisia campestris
Artemisia pancicii
Artemisia pontica
Artemisia scoparia
Asparagus officinalis
Asperula cynanchica
Asperula purpurea
Asperula rumelica
Asperula tenella
Asperula tephrocarpa
Asperula tinctoria
Aster amellus
Astragalus albicaulis
Astragalus asper
Astragalus austriacus
Astragalus danicus
Astragalus dasyanthus
Astragalus exscapus
Astragalus onobrychis
Astragalus pubiflorus
Astragalus sulcatus
Astragalus ucrainicus
Astragalus vesicarius
Aurinia saxatilis
Avenula pubescens (also *Molinio-Arrhenatheretea*)
Bassia prostrata
Bellevalia speciosa
Bothriochloa ischaemum

Brachypodium pinnatum agg.
Bromus cappadocica
Bromus erectus
Bromus inermis
Bromus riparius
Buphthalmum salicifolium (also *Elyno-Seslerietea*)
Bupleurum affine
Campanula glomerata
Campanula macrostachya
Campanula rotundifolia agg. (also *Nardetea strictae*)
Campanula sibirica
Carduus collinus
Carduus nutans
Carex caryophyllea
Carex hallerana
Carex humilis
Carex michelii
Carex montana
Carex obtusata
Carex pediformis
Carlina acanthifolia
Carlina acaulis
Carlina vulgaris agg.
Centaurea lavrenkoana
Centaurea macroptilon
Centaurea nigra
Centaurea orientalis
Centaurea reichenbachii
Centaurea scabiosa
Centaurea stereophylla
Centaurea stoebe
Centaurium erythraea
Cephalaria radiata
Cephalaria uralensis
Cerastium banaticum
Chrysopogon gryllus
Cirsium acaule
Cirsium pannonicum
Cleistogenes serotina
Colchicum hungaricum
Convolvulus cantabrica
Coronilla vaginalis
Crambe tataria
Crepis nicaeensis
Crepis pannonica
Crepis praemorsa
Cruciata pedemontana
Crupina vulgaris
Cuscuta epithymum
Cymbaria borysthenica
Cynoglottis barrelieri
Cytisus austriacus
Cytisus hirsutus
Cytisus paczoskii
Cytisus podolicus
Cytisus procumbens
Cytisus ratisbonensis
Cytisus ruthenicus
Dactylorhiza sambucina
Danthonia alpina
Dianthus borbasii
Dianthus capitatus
Dianthus carbonatus
Dianthus carthusianorum agg.
Dianthus collinus
Dianthus henteri
Dianthus leptopetalus
Dianthus moravicus
Dianthus petraeus
Dianthus plumarius
Dianthus praecox
Dianthus squarrosus
Diplotaxis cretacea
Dorycnium pentaphyllum agg.
Draba lasiocarpa
Dracocephalum austriacum
Dracocephalum ruyschiana
Echinops banaticus
Echinops ritro
Elytrigia intermedia

Elytrigia stipifolia
Erigeron acris
Eryngium amethystinum
Eryngium campestre
Eryngium planum
Erysimum carniolicum
Erysimum odoratum
Erysimum sylvestre
Euphorbia cyparissias
Euphorbia esula agg.
Euphorbia nicaeensis s.l.
Euphorbia verrucosa
Euphrasia stricta agg.
Festuca dalmatica
Festuca pallens s.l.
Festuca pseudodalmatica
Festuca stricta subsp. *stricta*
Festuca stricta subsp. *sulcata*
Festuca stricta subsp. *trachyphylla*
Festuca valesiaca s.l.
Filipendula vulgaris
Fragaria viridis
Fritillaria ruthenica
Fumana procumbens
Gagea pratensis
Gagea pusilla
Galatella linosyris
Galatella sedifolia
Galatella villosa
Galium glaucum
Galium octonarium
Galium ruthenicum
Genista januensis
Genista pilosa
Genista sagittalis
Genista scythica
Gentiana cruciata
Gentianella amarella
Gentianella austriaca
Gentianella ciliata
Gentianella germanica
Gladiolus illyricus
Gladiolus tenuis
Globularia bisnagarica
Goniolimon besseranum
Goniolimon tataricum
Gymnadenia odoratissima (also *Elyno-Seslerietea*)
Gypsophila paniculata
Haplophyllum suaveolens
Hedysarum grandiflorum
Helianthemum canum
Helianthemum nummularium agg.
Helictochloa adsurgens
Helictochloa hookeri subsp. *schelliana*
Helictochloa praeusta
Helictochloa pratensis
Helictotrichon desertorum
Herniaria incana
Hesperis tristis
Hierochloe odorata
Hierochloe repens
Himantoglossum adriaticum
Hippocrepis comosa
Hyacinthella leucophaea
Hypochaeris maculata
Hyssopus cretaceus
Inula ensifolia
Inula hirta
Inula oculus-christi
Iris aphylla
Iris humilis
Iris pontica
Iris pumila
Iris variegata
Jurinea mollis s.l.
Jurinea stoechadifolia
Klasea erucifolia
Klasea lycopifolia
Klasea radiata
Knautia illyrica

Knautia kitaibelii
Knautia purpurea
Koeleria brevis
Koeleria macrantha
Koeleria pyramidata
Koeleria talievii
Lactuca perennis
Lactuca viminea
Lathyrus pallescens
Lathyrus pannonicus
Leontodon biscutellifolius
Leontodon incanus
Leucanthemum platylepis
Ligularia carpathica
Lilium bulbiferum
Linaria angustissima
Linaria biebersteinii
Linaria genistifolia
Linum austriacum
Linum catharticum
Linum dolomiticum Borb.
Linum flavum
Linum hirsutum
Linum nervosum
Linum tenuifolium
Linum ucranicum
Linum viscosum
Malabaila graveolens
Marrubium peregrinum
Marrubium pestalozzae
Matthiola fragrans
Medicago falcata
Medicago prostrata
Melampyrum arvense
Melica ciliata
Melica transsilvanica
Minuartia setacea
Muscari comosum
Muscari neglectum
Muscari tenuiflorum
Neotinea tridentata
Neotinea ustulata
Nepeta nuda
Nepeta ucranica
Noccaea jankae
Noccaea kovatsii
Noccaea macrantha
Noccaea praecox
Nonea pulla
Odontites luteus
Odontites vulgaris agg.
Onobrychis alba
Onobrychis viciifolia agg.
Ononis pusilla
Ononis spinosa s.l.
Onosma helvetica
Onosma heterophylla
Onosma pseudoarenaria
Onosma simplicissima
Onosma tornensis
Onosma visianii
Ophrys apifera
Ophrys insectifera
Ophrys sphegodes
Orchis mascula
Orchis militaris
Orchis purpurea
Orchis simia
Ornithogalum comosum
Ornithogalum fischerianum
Ornithogalum kochii
Orobanche alba
Orobanche arenaria
Orobanche artemisiae-campestris
Orobanche caryophyllacea
Orobanche coerulescens
Orobanche elatior
Orobanche gracilis
Orobanche lanuginosa
Orobanche lutea

Orobanche purpurea
Orobanche reticulata
Orobanche teucrii
Oxytropis pilosa
Paeonia tenuifolia
Paronychia cephalotes
Pedicularis acaulis
Pedicularis kaufmannii
Peucedanum ruthenicum
Phleum phleoides
Phlomis pungens
Phlomis tuberosa
Pilosella bauhini
Pilosella cymosa
Pilosella densiflora
Pilosella hoppeana
Pilosella pavichii
Pilosella piloselloides agg.
Pimpinella tragium
Plantago holosteum
Plantago media
Poa badensis
Poa molinerii (also *Elyno-Seslerietea*)
Podospermum laciniatum
Podospermum purpureum
Polygala comosa
Polygala cretacea
Polygala major
Polygala nicaeensis
Pontechium maculatum
Potentilla astracanica
Potentilla collina agg.
Potentilla heptaphylla
Potentilla humifusa
Potentilla incana agg.
Potentilla pusilla agg.
Prangos ferulacea
Prunella grandiflora
Prunella laciniata
Psephellus marschallianus
Psephellus sumensis
Psephellus trinervius
Pulsatilla montana
Pulsatilla patens
Pulsatilla pratensis
Pulsatilla taurica
Pulsatilla vulgaris (incl. *P. grandis*)
Ranunculus bulbosus
Ranunculus illyricus
Ranunculus polyanthemos
Rapistrum perenne
Rhaponticoides ruthenica
Rhinanthus borbasii
Rindera umbelata
Salvia aethiopis
Salvia austriaca
Salvia dumetorum
Salvia nemorosa
Salvia nutans
Salvia pratensis
Salvia scabiosifolia
Salvia sclarea
Salvia transylvanica
Salvia verticillata
Sanguisorba minor
Satureja kitaibelii (also *Elyno-Seslerietea*)
Saxifraga bulbifera
Saxifraga granulata
Saxifraga paniculata (also *Elyno-Seslerietea*)
Scabiosa canescens
Scabiosa cinerea subsp. *hladnikiana*
Scabiosa columbaria
Scabiosa ochroleuca
Scabiosa triandra
Schivereckia podolica
Scorzonera austriaca
Scorzonera hispanica
Scorzonera mollis
Scorzonera villosa
Scrophularia rupestris

Scutellaria supina
Securigera varia
Senecio erucifolius
Senecio jacobaea
Seseli annuum
Seseli gracile
Seseli hippomarathrum
Seseli leucospermum
Seseli osseum
Seseli pallasii
Seseli peucedanoides
Seseli tortuosum
Sesleria caerulea (also *Elyno-Seslerietea*)
Sesleria heuflerana
Sesleria rigida (also *Elyno-Seslerietea*)
Sesleria sadlerana
Sesleria tenuifolia subsp. *kalnikensis*
Silene bupleuroides
Silene dichotoma
Spiranthes spiralis
Stachys recta
Sternbergia colchiciflora
Stipa asperella
Stipa capillata
Stipa dasyphylla
Stipa eriocaulis
Stipa joannis
Stipa lessingiana
Stipa pulcherrima
Stipa tirsa
Stipa ucrainica
Stipa zalesskii
Tanacetum millefolium
Taraxacum serotinum
Tephroseris besseriana
Tephroseris integrifolia
Teucrium chamaedrys
Teucrium montanum
Thalictrum foetidum
Thalictrum minus
Thalictrum simplex
Thalictrum uncinatum
Thesium alpinum (also *Elyno-Seslerietea*)
Thesium linophyllon
Thymus calcareus
Thymus longicaulis
Thymus pannonicus agg.
Thymus praecox agg.
Tragopogon dasyrhynchus
Tragopogon dubius
Tragopogon podolicus
Trifolium diffusum
Trifolium montanum
Trifolium ochroleucon
Trifolium pannonicum
Trifolium purpureum
Trigonella gladiata
Trinia glauca
Trinia kitaibelii
Trinia multicaulis
Tulipa biebersteiniana
Verbascum chaixii
Verbascum marschallianum
Verbascum phoeniceum
Verbascum speciosum
Veronica austriaca agg.
Veronica incana
Veronica orchidea
Veronica prostrata
Veronica spicata
Veronica vindobonensis
Vinca herbacea
Vincetoxicum rossicum
Viola hirta
Viola jooi
Viola rupestris

Molinio-Arrhenatheretea:

Achillea ptarmica
Agrostis gigantea
Agrostis stolonifera
Ajuga reptans
Allium angulosum
Allium suaveolens
Alopecurus arundinaceus
Alopecurus geniculatus
Alopecurus pratensis
Alopecurus rendlei
Anacamptis palustris
Angelica sylvestris
Arrhenatherum elatius
Astrantia major
Avenula pubescens
Barbarea vulgaris
Beckmannia eruciformis
Bellis perennis
Bromus commutatus
Bromus racemosus
Caltha palustris
Cardamine parviflora
Cardamine pratensis agg.
Carex brizoides
Carex cespitosa
Carex distans
Carex divisa
Carex hartmanii
Carex hirta
Carex leporina (also *Nardetea strictae*)
Carex pallescens (also *Nardetea strictae*)
Carex panicea
Carex tomentosa
Carum carvi
Centaurea jacea
Centaurea nigrescens
Centaurea phrygia
Cerastium fontanum s.l.
Chaerophyllum hirsutum
Cirsium canum
Cirsium heterophyllum
Cirsium oleraceum
Cirsium palustre
Cirsium rivulare
Clematis integrifolia
Colchicum autumnale
Crepis biennis
Crepis capillaris
Crepis mollis
Crepis paludosa
Crocus vernus
Cynosurus cristatus
Dactylis glomerata
Dactylorhiza incarnata
Dactylorhiza majalis
Daucus carota
Deschampsia cespitosa
Dianthus superbus
Epilobium hirsutum
Epilobium parviflorum
Epilobium tetragonum
Epipactis palustris
Equisetum palustre
Euphorbia palustris
Euphrasia rostkoviana agg.
Festuca arundinacea
Festuca pratensis
Festuca rubra agg. (also *Nardetea strictae*)
Festuca uechtritziana
Fritillaria meleagris
Fritillaria meleagroides
Galega officinalis
Galium uliginosum
Gaudinia fragilis
Gentiana pneumonanthe
Geranium palustre
Geranium pratense
Geranium sylvaticum

Geum rivale
Gladiolus palustris
Gratiola officinalis
Heracleum sphondylium
Herminium monorchis
Holcus lanatus
Hordeum secalinum
Hypericum tetrapterum
Inula britannica
Iris sibirica
Juncus acutiflorus
Juncus atratus
Juncus compressus
Juncus conglomeratus
Juncus effusus
Juncus inflexus
Juncus tenuis
Laserpitium prutenicum
Lathyrus palustris
Lathyrus pratensis
Linum bienne
Lolium multiflorum
Lolium perenne
Lotus pedunculatus
Lotus tenuis
Lychnis flos-cuculi
Lysimachia nummularia
Lysimachia punctata
Lysimachia vulgaris
Lythrum virgatum
Melilotus dentatus
Mentha arvensis
Mentha longifolia
Mentha x dumetorum
Mentha x verticillata
Molinia caerulea agg.
Myosotis scorpioides agg.
Narcissus poeticus
Narcissus pseudonarcissus
Noccaea brachypetala
Noccaea caerulescens
Oenanthe fistulosa
Oenanthe silaifolia
Ophioglossum vulgatum
Ornithogalum umbellatum
Orobanche minor
Pastinaca sativa
Peucedanum coriaceum
Phleum pratense agg.
Pimpinella major
Plantago altissima
Plantago major
Poa palustris
Poa trivialis
Polemonium caeruleum
Polygonum bistorta
Potentilla anglica
Potentilla anserina
Potentilla reptans
Potentilla supina
Prunella vulgaris
Pulicaria dysenterica
Ranunculus acris
Ranunculus flammula
Ranunculus repens
Rhinanthus alectorolophus
Rhinanthus angustifolius
Rorippa austriaca
Rorippa islandica
Rorippa pyrenaica
Rorippa sylvestris
Rumex acetosa
Rumex confertus
Rumex conglomeratus
Rumex crispus
Rumex obtusifolius
Rumex stenophyllus
Sanguisorba officinalis
Scirpoides holoschoenus
Scirpus sylvaticus

Scorzoneroides autumnalis
Scutellaria hastifolia
Selinum carvifolia
Selinum dubium
Senecio aquaticus
Serratula coronata
Serratula tinctoria
Seseli libanotis
Seseli longifolium
Silaum silaus
Silene dioica
Silene multiflora
Stachys palustris
Stellaria graminea (also *Nardetea strictae*)
Succisella inflexa
Symphytum officinale
Tephroseris crispa
Teucrium scordium
Thalictrum flavum
Thalictrum lucidum
Tragopogon tommasinii
Trifolium badium (also *Elyno-Seslerietea*)
Trifolium dubium
Trifolium fragiferum
Trifolium hybridum
Trifolium incarnatum
Trifolium pallidum
Trifolium patens
Trifolium pratense
Trifolium repens
Trifolium spadiceum
Trisetum flavescens
Trollius europaeus
Valeriana dioica agg.
Valeriana officinalis agg.
Verbena officinalis
Veronica chamaedrys
Veronica filiformis
Veronica longifolia
Veronica serpyllifolia
Vicia cracca
Vicia sepium
Viola elatior
Viola persicifolia
Viola pumila

Nardetea strictae:

Agrostis capillaris
Antennaria dioica
Arnica montana
Avenella flexuosa
Botrychium lunaria (also *Elyno-Seslerietea*)
Botrychium matricariifolium
Calluna vulgaris
Campanula rotundifolia (also *Festuco-Brometea*)
Campanula serrata
Carex leporina (also *Molinio-Arrhenatheretea*)
Carex pallescens (also *Molinio-Arrhenatheretea*)
Carex pilulifera
Danthonia decumbens
Dianthus deltoides
Diphasiastrum complanatum
Festuca rubra agg. (also *Molinio-Arrhenatheretea*)
Gentianella lutescens
Hypericum maculatum
Hypochaeris radicata
Juncus squarrosus
Luzula campestris agg.
Lycopodium clavatum
Nardus stricta
Pedicularis sylvatica
Pilosella aurantiaca
Pilosella lactucella
Pilosella officinarum
Platanthera bifolia
Polygala vulgaris
Potentilla erecta
Sagina saginoides
Stellaria graminea (also *Molinio-Arrhenatheretea*)

Thesium pyrenaicum
Vaccinium myrtillus
Veronica officinalis
Viola canina

Koelerio-Corynephoretea:

Achillea micrantha
Agropyron dasyanthum
Agrostis vinealis
Aira caryophyllea
Alkanna tinctoria
Alyssum hirsutum
Alyssum tortuosum
Anchusa gmelinii
Anchusa ochroleuca
Androsace elongata
Androsace septentrionalis
Anthemis ruthenica
Arenaria rigida
Armeria maritima
Asperula graveolens
Astragalus varius
Carex stenophylla
Centaurea diffusa
Centaurea triniifolia
Chondrilla juncea
Colchicum arenarium
Corispermum nitidum
Corynephorus canescens
Dianthus bessarabicus
Dianthus platyodon
Dianthus pseudarmeria
Dianthus serotinus
Dianthus sylvestris
Ephedra distachya
Equisetum ramosissimum
Erysimum diffusum
Festuca arenicola
Festuca beckeri
Festuca polesica
Festuca psammophila
Festuca vaginata
Festuca wagneri
Gypsophila collina
Gypsophila fastigiata
Helichrysum arenarium
Herniaria polygama
Jasione montana
Jurinea cyanoides
Koeleria glauca
Minuartia verna agg.
Minuartia viscosa
Moenchia mantica
Onosma arenaria
Papaver dubium
Petrorhagia saxifraga
Peucedanum arenarium
Polygonum graminifolium
Potentilla inclinata
Secale sylvestre
Sedum annuum
Senecio borysthenicus
Silene chlorantha
Silene lithuanica
Silene viscosa
Stipa borysthenica
Syrenia montana
Teesdalia nudicaulis
Thymus moldavicus
Thymus pallasianus
Thymus serpyllum
Tragopogon borysthenicus
Tragopogon ucrainicus
Trifolium striatum
Trifolium strictum
Viola hymettia

Elyno-Seslerietea:

Achillea clavennae
Androsace chamaejasme
Androsace lactea
Anemone narcissiflora
Arabis ciliata
Asperula capitata
Aster alpinus
Astragalus australis
Bartsia alpina
Bellidiastrum michelii
Biscutella laevigata
Botrychium lunaria (also *Nardetea strictae*)
Buphthalmum salicifolium (also *Festuco-Brometea*)
Bupleurum longifolium
Campanula kladniana
Campanula thyrsoides
Carex firma
Carex sempervirens
Centaurea atropupurea
Centaurea kotschyana
Cephalaria laevigata
Clinopodium alpinum
Crepis jacquinii
Cyanus pinnatifidus
Dianthus spiculifolius
Draba aizoides
Edraianthus graminifolius
Erigeron glabratus
Euphrasia salisburgensis
Ferula heuffelii
Festuca tatrae
Festuca versicolor
Gentiana clusii
Gentiana pumila
Gentiana utriculosa
Gentiana verna
Gentianella aspera
Globularia cordifolia
Gymnadenia odoratissima (also *Festuco-Brometea*)
Gypsophila repens
Helictotrichon decorum
Hieracium bifidum
Hieracium caesium
Hieracium pilosum
Hieracium villosum
Iris reichenbachii
Lathyrus laevigatus
Leontopodium nivale subsp. *alpinum*
Ligusticum mutellina
Lilium carniolicum
Myosotis alpestris
Pedicularis comosa
Pedicularis elongata subsp. *julica*
Pedicularis verticillata
Peucedanum altissimum
Phleum hirsutum
Phyteuma orbiculare
Plantago atrata
Poa alpina
Poa molinerii (also *Festuco-Brometea*)
Poa rehmannii
Podospermum roseum
Potentilla crantzii
Ranunculus carinthiacus
Ranunculus thora
Rhinanthus glacialis
Saponaria bellidifolia
Satureja kitaibelii (also *Festuco-Brometea*)
Saxifraga adscendens
Saxifraga marginata
Saxifraga paniculata (also *Festuco-Brometea*)
Scabiosa lucida
Sedum atratum
Selaginella selaginoides
Senecio doronicum
Sesleria caerulea (also *Festuco-Brometea*)
Sesleria rigida (also *Festuco-Brometea*)
Thesium alpinum (also *Festuco-Brometea*)

Traunsteinera globosa
Trifolium badium (also *Molinio-Arrhenatheretea*)
Trisetum alpestre
Veronica aphylla
Veronica fruticans

Reference

Mucina, L., Bültmann, H., Dierßen, K., Theurillat, J.-P., Raus, T., Čarni, A., Šumberová, K., Willner, W., Dengler, J., (...) & Tichý, L. 2016. Vegetation of Europe: Hierarchical floristic classification system of vascular plant, bryophyte, lichen, and algal communities. *Applied Vegetation Science* (in press).
