# Supplementary material for: A higher‐level classification of the Pannonian and western Pontic steppe grasslands (Central and Eastern Europe)
Source: Appl Veg Sci. 2016 Sep 16;20(1):143–58. doi: 10.1111/avsc.12265 (PMC5348766; doi:10.1111/avsc.12265)
Supplement: Supplementary file 4 — Appendix S4. Synoptic table of the TWINSPAN classification. [file AVSC-20-143-s004.pdf]

Supporting information to the paper

Willner, W. et al. A higher-level classification of the Pannonian and western Pontic steppe grasslands (Central and Eastern Europe). *Applied Vegetation Science.*

Appendix S4. Synoptic table of the Twinspan classification (division level 4). Values are percentage constancy. The first column gives the total number of occurences. Species are sorted according to the data set specific fidelity calculated as phi value. Species not reaching a threshold of phi=0.2 are sorted according to their total number of occurrences. Light shading: phi > 0.2, dark shading: phi > 0.4. Constancy and fidelity of non-vascular plants (NVP) were calculated within the subset of relevés in which these species were recorded.

Cluster number		1	2	3	4	5	6	7	8	9	10	11	12	13	14	15	16
No. of relevés (all)		257	260	213	797	1411	4434	526	1306	1779	5089	201	203	761	429	219	108
- (with NVP recorded)		133	133	51	234	471	1227	173	409	559	1421	46	121	145	59	100	12
**Vascular plants**																	
Molinia caerulea agg.	674	95	19	1	2	1	6	1	5	1	1	.	.	1	.	1	1
Carex hostiana	137	28	2	.	.	1	1	.	.	.	.	.	.	.	.	.	.
Serratula tinctoria	657	46	10	.	19	1	5	1	6	2	1	.	.	1	.	1	.
Sanguisorba officinalis	1248	57	30	1	21	3	16	1	6	1	1	.	.	1	1	2	.
Galium boreale agg.	1052	54	10	20	20	2	9	2	7	4	1	.	.	1	.	3	.
Phragmites australis	250	28	5	.	5	1	1	1	1	1	1	.	.	.	.	.	.
Carex davalliana	139	25	8	.	.	.	1	.	.	.	.	.	.	.	.	.	.
Plantago altissima	103	18	2	.	4	.	1	.	1	.	1	.	.	.	.	.	.
Gentiana pneumonanthe	160	20	4	1	4	1	1	1	1	1	1	.	.	.	.	.	.
Mentha aquatica	140	21	8	.	4	.	1	.	.	.	.	.	.	.	.	.	.
Lotus maritimus	143	16	1	.	1	.	1	2	1	1	1	.	.	.	.	.	.
Salix repens	116	16	2	.	1	1	1	.	1	1	1	2	.	.	.	.	.
Schoenus ferrugineus	38	11	.	.	.	.	1	.	.	.	.	.	.	.	.	.	.
Epipactis palustris	106	15	6	.	1	1	1	.	1	1	.	.	.	.	.	.	.
Taraxacum sect. Palustria	103	14	4	.	4	.	1	.	1	.	.	.	.	.	.	.	.
Schoenus nigricans	31	8	.	.	.	.	1	.	1	.	.	.	.	.	.	.	.
Sesleria uliginosa	93	11	1	.	1	1	1	.	1	1	.	.	.	.	.	.	.
Pulicaria dysenterica	51	9	1	.	1	.	1	.	.	.	.	.	.	.	.	.	.
Parnassia palustris	131	14	5	.	1	1	1	.	1	1	1	.	.	1	.	3	2
Carex pulicaris	42	7	.	.	.	1	1	.	1	.	.	.	.	.	.	.	.
Juncus alpinoarticulatus	25	7	1	.	.	.	1	.	.	.	.	.	.	.	.	.	.
Carex buxbaumii	33	7	.	.	1	1	1	.	.	.	.	.	.	.	.	.	.
Centaurea jacea	4730	58	36	25	33	38	49	39	49	19	5	.	.	1	.	.	.
Laserpitium prutenicum	54	6	.	.	1	.	1	.	1	.	.	.	.	.	.	.	.
Peucedanum coriaceum	27	6	1	.	1	.	1	.	1	.	.	.	.	.	.	.	.
Carex distans	236	13	6	2	7	.	2	1	1	1	1	.	.	.	.	.	.
Silaum silaus	149	10	1	1	6	.	1	1	1	1	1	.	.	.	.	.	.
Myosotis scorpioides agg.	679	4	54	.	8	9	7	.	1	.	.	.	.	.	.	.	.
Scirpus sylvaticus	215	5	42	.	4	1	1	.	.	.	.	.	.	.	.	.	.
Caltha palustris	248	17	40	.	7	1	1	.	.	.	.	.	.	.	.	.	.
Juncus effusus	371	10	39	1	9	2	3	1	.	.	.	.	.	.	.	.	.
Juncus conglomeratus	380	10	36	.	2	2	5	.	1	1	.	.	.	.	.	.	.
Crepis paludosa	134	5	23	.	1	1	1	.	.	.	.	.	.	.	.	1	.
Carex nigra	348	19	34	1	5	3	3	.	.	.	1	.	.	.	.	.	.
Angelica sylvestris	506	12	32	.	3	5	6	.	1	1	1	.	.	.	.	1	.
Mentha longifolia	219	1	21	1	4	1	3	2	1	1	1	.	.	.	.	.	.
Dactylorhiza majalis	199	3	20	1	1	2	2	.	1	.	.	.	.	.	.	.	.
Geum rivale	210	2	18	.	2	1	3	.	1	.	.	.	.	.	.	.	.
Lathyrus pratensis	2600	29	54	1	30	14	36	12	18	2	1	.	.	.	.	1	.
Carex flava agg.	289	17	26	1	2	2	3	.	1	.	.	.	.	.	.	1	1
Cirsium oleraceum	254	5	18	.	3	1	4	1	1	.	1	.	.	.	.	.	.
Juncus inflexus	134	11	19	.	3	.	1	1	1	.	.	.	.	.	.	.	.
Lycopus europaeus	118	6	17	.	5	.	1	1	.	.	.	.	.	.	.	.	.
Galium uliginosum	209	12	20	1	4	1	2	.	1	.	.	.	.	.	.	.	.
Agrostis canina	288	12	22	6	4	1	3	1	1	.	1	.	1	.	.	.	.
Ajuga reptans	1283	7	27	.	5	11	20	1	8	1	1	.	.	1	.	2	.
Cirsium palustre	216	5	13	1	1	5	2	.	.	.	.	.	.	.	.	.	.
Eriophorum angustifolium	71	10	12	.	1	1	1	.	.	.	.	.	.	.	.	.	.
Mentha arvensis	330	4	18	8	12	1	3	.	1	1	1	.	.	.	.	.	.
Carex leporina	350	1	14	4	4	5	4	.	1	.	.	.	.	.	.	.	.
Equisetum fluviatile	53	5	9	.	1	1	1	.	.	.	.	.	.	.	.	.	.
Epilobium palustre	49	2	8	.	2	1	1	.	.	.	1	.	.	.	.	.	.
Betonica officinalis	2035	20	33	1	3	12	21	8	30	14	2	.	.	1	.	2	.
Carex canescens	25	.	5	.	1	1	1	.	.	.	.	.	.	.	.	.	.
Carex paniculata	28	2	6	.	.	1	1	.	1	.	.	.	.	.	.	.	.
Succisella inflexa	65	2	7	.	2	.	1	.	.	.	.	.	.	.	.	.	.
Koeleria delavignei	199	1	.	53	2	1	1	1	.	.	1	.	1	.	.	.	.
Rumex thyrsiflorus	288	.	.	43	3	1	1	4	1	1	1	.	1	.	.	.	.
Rumex confertus	142	.	.	23	5	.	1	.	.	.	1	.	.	.	.	.	.
Dianthus borbasii	104	1	.	15	1	1	1	1	.	.	1	.	2	.	.	.	.
Agrostis gigantea	300	7	1	23	7	1	2	1	1	1	1	1	1	.	.	1	.
Lotus ucrainicus	161	.	1	13	1	.	1	1	1	.	1	.	.	.	.	.	.
Gypsophila muralis	88	.	.	12	2	1	1	1	.	1	1	.	2	.	.	.	.
Stellaria hippoctona	16	.	.	7	.	.	1	.	.	.	1	.	.	.	.	.	.
Elytrigia repens	1657	1	3	32	26	3	12	15	2	4	12	2	7	1	.	.	.
Vicia tetrasperma	563	1	1	19	7	1	4	10	2	1	3	1	1	.	.	.	.

Cluster number		1	2	3	4	5	6	7	8	9	10	11	12	13	14	15	16
No. of relevés (all)		257	260	213	797	1411	4434	526	1306	1779	5089	201	203	761	429	219	108
- (with NVP recorded)		133	133	51	234	471	1227	173	409	559	1421	46	121	145	59	100	12
Juncus atratus	64	1	.	9	5	.	1	.	.	.	.	.	.	.	.	.	.
Achillea salicifolia	41	.	.	7	3	.	.	.	.	.	1	.	.	.	.	.	.
Carex lachenalii	24	.	.	6	1	1	1	.	.	.	.	.	.	.	.	.	.
Eryngium planum	299	.	.	11	2	.	1	4	1	2	3	.	.	1	.	.	.
Euphorbia esula	885	1	.	21	5	1	5	17	7	9	5	.	1	1	1	.	.
Equisetum arvense	1374	15	20	29	15	6	19	9	4	1	1	.	1	.	.	1	.
Hieracium umbellatum	547	4	4	17	1	1	2	4	2	8	3	3	1	1	.	.	.
Alopecurus pratensis	2290	9	30	37	65	3	31	13	4	1	1	.	.	1	.	.	.
Potentilla reptans	1348	14	18	3	51	1	15	12	5	1	1	.	.	.	.	.	.
Symphytum officinale	494	10	6	.	29	.	5	1	1	1	1	.	.	.	.	.	.
Rumex crispus	692	1	5	5	29	1	9	2	1	1	1	.	.	.	.	.	.
Carex vulpina agg.	340	2	16	4	27	1	1	1	.	.	1	.	.	.	.	.	.
Potentilla anserina	576	17	7	15	32	1	5	1	1	1	1	.	.	.	.	.	.
Rorippa sylvestris	160	.	.	2	14	.	1	1	.	1	1	.	.	.	.	.	.
Lythrum virgatum	134	1	.	3	14	.	1	1	.	.	1	.	.	.	.	.	.
Poa palustris	288	2	7	11	23	1	1	1	.	.	1	.	.	.	.	.	.
Scutellaria hastifolia	118	.	.	2	13	.	1	1	.	1	1	.	.	.	.	.	.
Phalaroides arundinacea	124	.	1	1	12	1	1	1	.	1	.	.	.	.	.	.	.
Gratiola officinalis	356	3	10	17	25	.	2	1	.	.	1	.	.	.	.	.	.
Trifolium hybridum	568	5	10	14	27	4	5	1	1	.	1	.	1	.	.	.	.
Veronica longifolia	147	3	1	5	14	.	1	1	.	1	1	.	.	.	.	.	.
Glechoma hederacea agg.	1038	3	10	13	27	1	14	6	3	1	1	.	.	1	.	1	1
Plantago major	706	4	5	7	21	1	10	2	1	1	1	.	.	1	.	.	.
Lotus tenuis	144	2	1	.	10	.	1	1	1	.	1	.	.	.	.	.	.
Iris pseudacorus	98	1	3	.	10	.	1	.	.	.	.	.	.	.	.	.	.
Eleocharis palustris agg.	103	3	2	1	11	.	1	.	.	.	1	.	.	.	.	.	.
Ranunculus auricomus agg.	736	8	15	4	22	5	8	1	4	1	1	.	.	.	.	.	.
Carex acuta	241	12	15	.	17	.	1	.	.	.	.	.	.	.	.	.	.
Selinum dubium	160	3	1	8	12	.	1	1	1	.	1	.	.	.	.	.	.
Trifolium fragiferum	135	1	1	3	9	.	1	1	.	.	1	.	.	.	.	.	.
Cirsium arvense	1096	5	4	1	21	5	14	9	5	2	1	.	1	.	.	1	.
Oenanthe silaifolia	84	.	1	.	6	.	1	1	1	.	1	.	.	.	.	.	.
Rorippa austriaca	63	.	.	1	5	.	1	.	.	.	1	.	.	.	.	.	.
Nardus stricta	1288	2	11	.	1	66	6	1	3	1	1	.	1	.	.	.	.
Hypericum maculatum	1667	2	7	.	1	64	15	1	6	1	1	.	.	1	.	1	.
Polygala vulgaris	1712	1	2	4	2	58	10	2	19	4	2	.	.	1	.	1	2
Agrostis capillaris	3850	1	10	10	4	88	37	25	31	6	4	.	21	1	.	.	5
Alchemilla spec.div.	2607	2	23	.	2	65	31	2	16	1	1	.	.	.	.	2	.
Veronica officinalis	949	1	2	.	1	40	6	1	6	1	1	.	1	1	.	1	1
Viola canina	1349	4	3	2	1	48	10	4	11	1	1	.	.	1	.	.	.
Carex pilulifera	379	.	.	.	.	23	1	1	1	1	.	.	.	.	.	.	.
Luzula luzuloides	532	.	1	.	.	27	3	.	1	1	1	.	.	1	.	2	2
Cruciata glabra	2828	5	28	1	1	65	27	2	35	7	1	.	1	1	.	7	2
Danthonia decumbens	1087	14	2	1	1	38	6	2	13	2	1	.	.	.	.	.	.
Vaccinium myrtillus	260	.	.	.	.	18	1	.	1	.	.	.	.	.	.	1	.
Stellaria graminea	2835	4	19	27	16	52	34	10	10	1	3	.	1	.	.	.	.
Euphrasia rostkoviana agg.	954	8	4	.	1	29	7	1	8	2	1	.	.	2	.	7	.
Antennaria dioica	288	.	.	.	.	15	1	.	3	1	1	1	.	1	1	1	.
Arabidopsis halleri	270	.	.	.	.	12	2	.	1	.	.	.	.	.	.	.	.
Gentiana asclepiadea	208	1	.	.	.	11	1	.	1	1	.	.	.	.	.	1	.
Pilosella officinarum	2814	1	1	1	1	46	8	24	18	14	21	2	24	7	7	5	1
Potentilla aurea	175	.	.	.	.	10	1	.	.	1	.	.	.	1	.	1	.
Avenella flexuosa	222	.	.	.	.	11	1	.	1	1	1	.	1	1	.	1	.
Poa chaixii	141	.	.	.	1	9	1	.	1	1	.	.	.	.	.	.	.
Gnaphalium sylvaticum	170	.	.	.	1	9	1	1	1	.	.	.	.	.	.	.	.
Hieracium lachenalii	166	.	.	.	.	10	1	.	1	1	1	.	.	1	.	1	1
Calluna vulgaris	294	1	1	.	1	13	1	1	2	1	1	.	4	1	.	.	.
Holcus mollis	200	.	1	.	1	8	2	.	1	1	1	.	1	.	.	.	.
Phyteuma spicatum	300	1	1	.	.	11	3	.	1	1	1	.	.	1	.	1	2
Trifolium medium	1300	2	7	1	2	23	9	8	18	4	3	.	.	.	.	1	.
Crepis mollis	202	.	2	.	.	8	2	.	1	1	.	.	.	.	.	1	.
Gladiolus imbricatus	307	1	3	1	1	9	3	.	1	.	.	.	.	.	.	.	.
Crepis biennis	1522	2	1	1	3	5	27	6	12	2	1	.	1	1	.	.	.
Festuca pratensis	4747	30	50	29	50	32	62	33	32	7	3	.	.	.	.	1	.
Cerastium fontanum	3492	11	22	14	27	34	46	20	20	3	4	.	4	1	.	1	1
Bellis perennis	825	2	3	.	4	3	15	3	2	.	1	.	.	.	.	.	.
Carum carvi	1159	.	5	5	4	15	18	1	5	1	1	.	.	.	.	1	.
Heracleum sphondylium	1243	1	5	2	2	11	20	3	8	2	1	.	.	.	.	8	.
Anthriscus sylvestris	364	1	1	.	1	1	7	1	1	.	1	.	.	.	.	.	.
Vicia sepium	750	.	2	.	1	8	13	2	3	1	1	.	1	.	.	1	.
Agrimonia eupatoria	2983	1	3	1	3	4	15	53	32	26	21	.	.	1	.	2	.
Cichorium intybus	1165	1	1	15	7	1	10	33	4	3	7	1	1	1	.	.	.
Convolvulus arvensis	2102	2	1	4	3	1	14	28	11	7	20	1	3	1	1	1	.
Trifolium campestre	1188	2	.	1	7	1	8	23	11	3	8	2	9	1	.	.	1
Vicia sativa	580	.	.	1	5	1	4	14	4	2	3	.	1	.	.	.	.
Ranunculus polyanthemos agg.	2874	2	1	5	4	29	16	33	30	20	8	.	1	1	.	1	.
Bromus erectus	2052	5	1	.	1	1	10	11	51	27	6	.	.	2	5	.	.
Trifolium montanum	2723	8	5	9	2	24	13	15	57	30	8	.	.	1	.	1	2
Carex montana	597	1	.	.	.	3	1	.	24	8	1	.	.	1	.	2	1
Linum catharticum	2411	23	5	1	1	22	11	6	56	25	3	.	.	10	1	24	5
Carex caryophyllea	1766	.	1	.	1	15	5	8	40	21	7	2	4	1	1	.	.

Cluster number		1	2	3	4	5	6	7	8	9	10	11	12	13	14	15	16
No. of relevés (all)		257	260	213	797	1411	4434	526	1306	1779	5089	201	203	761	429	219	108
- (with NVP recorded)		133	133	51	234	471	1227	173	409	559	1421	46	121	145	59	100	12
Thymus longicaulis	198	.	.	.	.	.	.	1	13	1	.	.	.	.	.	.	.
Ranunculus bulbosus	1075	.	.	.	1	6	9	10	27	6	1	.	.	1	1	.	.
Koeleria pyramidata	525	5	1	.	.	1	1	1	22	7	1	.	.	1	.	2	.
Cirsium pannonicum	630	10	.	.	1	1	1	1	24	11	1	.	.	2	.	1	.
Polygala comosa	1141	2	1	2	1	2	6	16	29	11	3	.	.	1	.	.	1
Tragopogon pratensis	2197	2	1	.	3	12	26	14	35	9	3	2	1	1	1	3	.
Euphorbia verrucosa	230	.	.	.	.	1	1	.	11	3	1	.	.	.	.	.	.
Primula veris	1119	2	3	.	.	6	7	2	28	12	1	.	.	6	1	1	15
Viola hirta	1979	2	2	.	1	6	8	13	39	27	6	.	.	9	1	31	4
Buphthalmum salicifolium	456	.	.	.	.	1	1	1	19	7	1	.	.	3	.	13	.
Campanula glomerata	1162	1	1	1	1	9	6	2	24	19	2	.	.	2	1	3	.
Potentilla heptaphylla	1244	.	.	.	1	6	4	10	28	14	3	.	.	16	5	6	1
Scabiosa triandra	175	.	.	.	.	.	1	1	9	2	1	.	.	.	.	.	.
Avenula pubescens	1428	2	7	.	1	5	15	8	25	10	2	1	.	1	1	.	.
Genista sagittalis	148	.	.	.	.	1	1	1	8	1	1	.	.	.	.	.	.
Rhinanthus glacialis	116	.	.	.	.	1	1	.	7	1	.	.	.	.	.	.	.
Danthonia alpina	294	3	1	.	.	.	1	1	11	5	1	.	.	.	.	.	.
Potentilla alba	317	1	1	.	.	1	2	1	10	3	1	.	.	1	.	.	.
Filipendula vulgaris	3015	18	17	16	7	8	19	21	39	31	13	.	.	1	2	.	.
Ononis spinosa	1529	7	3	1	4	8	12	13	24	13	4	.	.	1	.	.	.
Anthyllis vulneraria	2286	1	.	.	.	15	6	6	39	28	7	1	.	27	31	37	2
Peucedanum cervaria	783	1	.	.	1	.	1	1	10	25	2	.	.	6	1	2	2
Aster amellus	650	.	.	.	.	.	1	1	1	21	4	.	.	6	1	1	7
Seseli annuum	831	.	.	.	.	1	1	9	9	21	5	1	1	1	1	.	.
Carex michelii	414	.	.	1	.	.	1	1	5	14	2	.	.	1	.	.	.
Onobrychis viciifolia agg.	1119	.	.	.	.	.	1	4	17	23	8	3	.	1	1	.	.
Thesium linophyllon	1341	.	.	1	.	1	1	1	15	30	7	1	1	14	17	1	2
Scabiosa ochroleuca	2677	1	.	.	1	1	2	20	15	42	24	19	2	22	14	3	3
Polygala major	465	.	.	.	.	1	1	1	8	15	1	.	.	2	1	.	.
Linum flavum	399	.	.	.	.	.	.	1	1	13	2	.	.	4	.	1	1
Asperula cynanchica	3840	1	1	.	1	1	1	13	26	48	39	3	1	41	34	17	2
Adonis vernalis	994	.	.	.	.	.	1	.	2	17	13	.	.	2	3	.	.
Securigera varia	3038	.	.	.	1	3	6	33	31	41	25	.	2	11	1	17	16
Salvia verticillata	1636	.	2	.	1	1	2	11	19	27	13	.	.	9	1	7	4
Melampyrum arvense	381	.	.	.	1	1	1	2	2	10	3	1	.	1	1	.	.
Inula hirta	395	1	.	.	.	.	1	1	4	11	2	.	.	2	2	.	.
Peucedanum alsaticum	226	.	.	.	1	.	1	2	1	7	1	.	.	.	.	.	.
Festuca valesiaca	3348	2	.	11	6	1	4	20	5	14	50	4	12	6	9	.	2
Thymus pannonicus agg.	3206	.	.	.	.	1	1	15	8	30	45	10	10	7	6	1	3
Koeleria macrantha	2716	.	.	1	1	1	1	11	9	21	39	8	3	10	9	.	3
Salvia nemorosa	1103	.	.	.	1	1	1	4	1	6	18	1	.	1	.	1	.
Bothriochloa ischaemum	2049	.	.	.	1	.	1	7	1	11	32	11	4	6	12	.	.
Stipa capillata	1345	.	.	.	.	.	.	.	.	3	23	14	2	4	12	.	.
Salvia nutans	505	.	.	.	.	.	.	1	1	2	9	.	.	.	.	.	.
Centaurea stoebe	2141	.	.	.	1	1	1	6	2	16	31	12	11	12	13	1	1
Euphorbia nicaeensis	774	.	.	.	.	.	1	.	1	7	12	1	.	1	2	.	.
Falcaria vulgaris	1046	.	.	.	1	.	1	4	1	9	16	6	1	1	1	.	.
Astragalus austriacus	517	.	.	.	.	.	1	1	.	3	9	.	.	1	1	.	.
Astragalus onobrychis	973	.	.	.	.	.	1	1	1	8	15	8	1	1	1	.	.
Artemisia austriaca	373	.	.	1	.	.	1	1	.	1	7	1	.	.	.	.	.
Nonea pulla	600	.	.	.	.	.	1	4	1	4	10	.	.	.	.	.	.
Taraxacum serotinum	315	.	.	.	.	.	1	1	.	1	6	.	.	1	.	.	.
Verbascum phoeniceum	595	.	.	.	.	.	1	2	1	3	10	1	2	1	1	.	.
Festuca vaginata	176	.	.	.	.	.	.	.	.	.	1	73	8	.	.	.	.
Koeleria glauca	195	.	.	.	.	1	1	1	1	1	1	64	9	1	.	.	.
Stipa borysthenica	118	.	.	.	.	.	.	.	.	1	1	43	2	.	.	.	.
Polygonum arenarium	109	.	.	.	.	.	1	.	.	.	1	43	5	.	.	.	.
Poa bulbosa	716	.	.	2	1	.	1	1	1	1	9	50	18	1	16	.	.
Alkanna tinctoria	42	.	.	.	.	.	.	.	.	.	.	20	1	.	.	.	.
Secale sylvestre	53	.	.	.	.	.	.	.	.	.	1	21	2	.	.	.	.
Minuartia glomerata	50	.	.	.	.	.	.	.	.	.	1	18	.	1	1	.	.
Centaurea arenaria agg.	52	.	.	.	.	.	.	1	1	.	1	18	2	.	.	.	.
Alyssum tortuosum	59	.	.	.	.	.	.	.	.	.	1	16	.	1	2	.	.
Viola kitaibeliana	105	.	.	.	.	.	.	.	1	1	1	16	.	1	2	.	.
Carex liparocarpos	216	.	.	.	.	.	.	.	1	1	2	23	1	1	13	.	.
Festuca wagneri	55	.	.	.	.	.	.	.	.	.	1	12	.	.	.	.	.
Syrenia cana	36	.	.	.	.	.	.	.	.	1	1	11	1	.	.	.	.
Salsola kali	28	.	.	.	.	.	.	.	.	.	1	11	1	.	.	.	.
Artemisia campestris	1367	.	.	1	1	1	1	11	1	4	19	38	27	9	4	1	.
Crepis foetida	155	.	.	.	1	.	1	1	1	1	2	16	5	1	1	.	.
Dianthus serotinus	74	.	.	.	.	.	.	.	.	.	1	17	7	1	3	.	.
Bassia laniflora	65	.	.	.	.	.	.	.	.	.	1	17	11	.	.	.	.
Silene conica	35	.	.	.	.	.	.	.	.	.	1	11	2	.	.	.	.
Bromus squarrosus	237	.	.	.	.	.	1	.	.	1	4	13	1	.	.	.	.
Echinops banaticus	24	.	.	.	.	.	.	.	.	.	1	8	.	.	.	.	.
Minuartia verna agg.	218	.	.	.	.	.	.	.	1	1	1	22	.	4	14	.	10
Gypsophila fastigiata	83	.	.	.	.	.	.	1	1	1	1	13	4	1	2	.	.
Tragopogon floccosus	16	.	.	.	.	.	.	.	.	.	1	7	.	.	.	.	.
Holosteum umbellatum	245	1	.	.	1	.	1	1	1	1	3	16	6	1	5	.	.
Asparagus officinalis	371	.	.	4	1	.	1	2	.	2	5	15	1	.	1	.	.
Corispermum nitidum	13	.	.	.	.	.	.	.	.	.	.	6	.	.	.	.	.

Cluster number		1	2	3	4	5	6	7	8	9	10	11	12	13	14	15	16
No. of relevés (all)		257	260	213	797	1411	4434	526	1306	1779	5089	201	203	761	429	219	108
- (with NVP recorded)		133	133	51	234	471	1227	173	409	559	1421	46	121	145	59	100	12
Chondrilla juncea	399	.	.	.	.	.	1	1	.	1	6	17	12	1	1	.	.
Pilosella echioides	276	.	1	.	.	.	.	.	.	1	5	12	1	1	2	.	.
Achillea ochroleuca	32	.	.	.	.	.	.	.	.	.	1	6	.	.	.	.	.
Sedum urvillei	12	.	.	.	.	.	.	.	.	.	.	6	.	.	.	.	.
Stipa joannis	459	.	.	.	.	.	.	.	1	5	6	15	1	3	4	.	.
Medicago minima	384	1	.	.	1	.	1	.	1	1	6	14	3	1	3	1	1
Buglossoides arvensis	69	.	.	.	.	.	1	.	.	1	1	7	1	1	1	.	.
Tragus racemosus	10	.	.	.	.	.	.	.	.	.	.	5	.	.	.	.	.
Scirpoides holoschoenus	59	2	.	.	.	.	1	.	1	.	1	8	1	.	.	.	.
Peucedanum arenarium	38	.	.	.	.	.	.	.	.	.	1	5	.	.	1	.	.
Festuca arenicola	9	.	.	.	.	.	.	.	.	.	.	4	.	.	.	.	.
Cynoglossum hungaricum	14	.	.	.	.	.	.	.	.	.	1	5	1	.	.	.	.
Gypsophila paniculata	98	.	.	.	.	.	.	.	.	1	2	6	.	1	.	.	.
Corynephorus canescens	158	.	.	.	.	1	1	.	.	.	1	.	67	.	.	.	.
Thymus serpyllum	252	1	.	.	1	1	1	1	1	1	1	6	49	1	1	.	.
Veronica dillenii	182	.	1	1	.	.	.	.	.	1	2	1	43	.	.	.	.
Jasione montana	204	.	.	.	.	1	1	1	1	1	2	1	37	.	.	.	.
Festuca psammophila	75	.	.	.	.	.	.	.	.	.	1	1	31	1	.	1	.
Rumex acetosella	1193	1	1	25	2	12	7	5	1	1	9	3	57	2	1	.	.
Scleranthus perennis	142	.	.	.	.	1	1	1	1	.	2	.	23	1	1	.	.
Carex supina	216	.	.	.	.	.	1	.	.	1	3	2	25	1	1	.	.
Trifolium arvense	843	.	.	4	1	1	1	9	1	1	12	3	39	1	1	.	.
Helichrysum arenarium	478	.	.	2	.	1	1	8	.	1	6	9	34	1	1	.	.
Spergula pentandra agg.	32	.	.	.	.	.	.	.	.	.	.	.	16	.	.	.	.
Filago minima	31	.	.	.	.	1	.	.	.	.	.	.	15	.	.	.	.
Anthemis ruthenica	95	.	.	2	.	.	1	.	.	1	1	7	20	.	.	.	.
Erysimum diffusum	312	.	.	.	.	.	.	.	.	1	4	16	27	1	4	.	.
Carex stenophylla	82	.	.	.	.	1	.	1	.	.	1	3	16	.	.	.	.
Plantago arenaria	50	.	.	1	.	.	1	.	.	1	1	4	13	.	.	.	.
Myosotis stricta	287	.	.	8	1	1	1	1	.	1	3	15	22	1	1	.	.
Armeria maritima	157	1	.	.	.	1	1	1	1	1	2	.	11	1	1	.	.
Scleranthus annuus agg.	112	.	.	1	1	1	1	1	1	1	1	1	12	.	.	.	.
Minuartia viscosa	16	.	.	.	.	.	.	.	.	.	1	.	7	.	.	.	.
Erophila verna	411	.	.	1	1	1	1	1	1	1	5	16	21	1	8	.	.
Anchusa officinalis	107	.	.	.	1	.	1	1	1	1	1	1	8	1	.	.	.
Veronica verna	200	.	.	5	1	.	1	1	1	1	2	3	12	1	1	.	.
Vicia lathyroides	116	.	.	.	.	1	1	1	1	.	1	1	8	.	.	.	.
Sedum sexangulare	1021	.	1	1	.	2	3	3	9	3	11	1	21	8	10	1	2
Genista pilosa	578	.	.	.	.	1	1	1	1	4	3	.	2	26	16	11	3
Allium lusitanicum	632	.	.	.	.	.	1	.	1	4	4	.	.	29	17	17	10
Bromus pannonicus	213	.	.	.	.	.	1	.	2	1	1	.	.	14	4	2	.
Cyanus triumfettii	410	.	.	.	.	.	1	1	2	6	2	.	.	18	2	11	10
Sedum album	508	.	.	.	.	.	1	.	1	1	3	.	.	23	21	21	9
Helianthemum nummularium	2294	.	1	.	.	9	3	2	32	29	11	9	.	41	39	27	14
Allium flavum	864	.	.	.	.	1	.	.	.	2	11	8	1	21	19	1	12
Campanula rotundifolia	693	.	.	1	1	3	1	4	2	9	3	.	1	22	12	14	9
Pulsatilla vulgaris	497	.	.	.	.	.	1	1	1	9	3	.	.	14	7	1	3
Thymus praecox	1158	.	.	.	.	.	1	1	1	5	9	.	1	29	84	3	.
Stipa eriocaulis	294	.	.	.	.	.	.	.	.	1	1	.	.	1	49	.	.
Scorzonera austriaca	385	.	.	.	.	.	.	.	1	1	1	.	.	8	52	.	.
Globularia bisnagarica	773	.	.	.	.	1	1	.	8	9	3	.	.	16	57	2	.
Hornungia petraea	189	.	.	.	.	.	.	1	.	1	1	.	.	2	35	.	.
Helianthemum canum	453	.	.	.	.	.	.	.	1	1	2	.	.	17	48	1	5
Linum tenuifolium	718	.	.	.	.	.	1	.	1	8	6	.	.	13	40	.	.
Seseli leucospermum	110	.	.	.	.	.	.	.	.	.	1	.	.	2	21	.	.
Paronychia cephalotes	99	.	.	.	.	.	.	.	.	.	1	.	.	1	18	.	1
Dianthus plumarius	90	.	.	.	.	.	.	.	.	.	1	.	.	2	18	1	.
Poa badensis	259	.	.	.	1	.	.	.	.	1	2	.	.	7	23	.	3
Minuartia setacea	242	.	.	.	.	.	.	.	.	1	2	2	.	5	23	.	2
Seseli hippomarathrum	401	.	.	.	.	.	.	.	1	3	5	1	.	2	21	1	.
Cerastium pumilum	410	1	.	.	1	.	1	2	1	1	4	2	8	1	24	.	.
Aethionema saxatile	63	.	.	.	.	.	.	.	1	.	1	.	.	1	12	.	.
Draba lasiocarpa	98	.	.	.	.	.	.	.	.	.	1	.	.	4	14	.	4
Silene otites agg.	922	.	.	.	.	.	1	.	1	3	11	26	22	7	34	1	3
Scabiosa canescens	280	.	.	.	.	1	1	1	1	4	2	.	.	3	13	.	.
Hippocrepis emerus	75	.	.	.	.	.	.	.	.	.	1	.	.	1	9	.	3
Onosma visianii	83	.	.	.	.	.	.	.	.	.	1	.	.	1	7	.	.
Festuca stricta ssp. stricta	58	.	.	.	.	.	.	.	.	1	1	.	.	.	6	.	.
Artemisia alba	88	.	.	.	.	.	.	.	.	.	1	.	.	1	7	.	.
Allium moschatum	21	.	.	.	.	.	.	.	.	.	.	.	.	.	5	.	.
Trinia glauca	128	.	.	.	.	.	.	.	1	1	1	.	.	1	7	.	.
Jurinea mollis	816	.	.	.	.	.	.	1	1	8	10	4	.	7	17	.	3
Pulsatilla halleri ssp. slavica	222	.	.	.	.	.	.	.	1	1	1	.	.	12	1	52	.
Carduus defloratus	169	.	.	.	.	.	1	.	1	1	1	.	.	5	.	46	2
Festuca tatrae	87	.	.	.	.	1	.	.	.	.	.	.	.	1	.	38	.
Thymus pulcherrimus	107	.	.	.	.	1	1	1	1	.	1	.	.	1	1	39	.
Scabiosa lucida	142	1	.	.	.	1	1	.	1	1	.	.	.	2	.	41	1
Minuartia laricifolia	123	.	.	.	.	.	.	.	.	1	1	.	.	4	1	38	.
Thesium alpinum	142	.	.	.	.	1	1	.	1	1	.	.	.	3	1	38	.
Galium pusillum agg.	460	.	.	.	.	5	1	1	6	1	1	.	.	9	2	51	.
Phyteuma orbiculare	406	1	.	.	.	2	1	.	3	1	.	.	.	12	4	55	13

Cluster number		1	2	3	4	5	6	7	8	9	10	11	12	13	14	15	16
No. of relevés (all)		257	260	213	797	1411	4434	526	1306	1779	5089	201	203	761	429	219	108
- (with NVP recorded)		133	133	51	234	471	1227	173	409	559	1421	46	121	145	59	100	12
Primula auricula	94	.	.	.	.	.	.	.	.	.	.	.	.	3	.	33	.
Calamagrostis varia	150	1	.	.	.	1	1	.	1	1	1	.	.	2	.	37	3
Hieracium bupleuroides	107	.	.	.	.	.	1	.	.	1	.	.	.	5	.	32	.
Erysimum witmannii	116	.	.	.	.	.	.	.	1	1	1	.	.	6	.	29	.
Bellidiastrum michelii	68	.	.	.	.	.	1	.	1	1	.	.	.	1	.	24	.
Kernera saxatilis	106	.	.	.	.	.	.	.	.	.	.	.	.	4	1	29	6
Laserpitium latifolium	278	1	.	.	.	1	1	.	5	3	1	.	.	3	.	32	11
Trisetum alpestre	50	.	.	.	.	.	1	.	.	.	1	.	.	1	.	19	2
Campanula cochleariifolia	39	.	.	.	.	.	.	.	.	.	.	.	.	1	.	17	.
Carex ornithopoda	113	.	.	.	.	1	1	1	3	1	1	.	.	1	.	19	.
Gentiana clusii	40	.	.	.	.	.	.	.	1	.	.	.	.	1	.	16	1
Carex sempervirens	35	.	.	.	.	.	1	.	1	1	.	.	.	.	.	15	.
Ranunculus breyninus	70	.	.	.	.	1	1	1	1	1	1	.	.	1	.	17	2
Clinopodium alpinum	297	.	.	.	.	1	1	.	2	2	1	.	.	16	1	28	6
Crepis jacquinii	27	.	.	.	.	.	.	.	.	.	.	.	.	.	.	12	.
Dianthus praecox	123	.	.	.	.	.	1	.	.	1	1	.	.	5	7	20	1
Carex digitata	82	.	.	.	.	.	1	1	1	1	1	.	.	1	.	18	7
Euphrasia salisburgensis	72	.	.	.	.	.	1	1	1	1	1	.	.	1	1	19	9
Hieracium murorum	104	.	.	.	.	2	1	.	1	1	1	.	.	2	.	16	3
Helianthemum oelandicum	32	.	.	.	.	.	.	.	.	1	.	.	.	1	.	11	1
Coronilla vaginalis	68	.	.	.	.	1	.	.	1	1	.	.	.	4	.	13	.
Rubus saxatilis	35	.	.	.	.	.	1	.	.	1	.	.	.	1	.	10	.
Allium ericetorum	77	.	.	.	.	.	.	.	.	1	1	.	.	6	.	14	.
Carex firma	20	.	.	.	.	.	.	.	.	.	.	.	.	.	.	9	.
Veronica fruticans	27	.	.	.	.	.	1	1	.	1	1	.	.	1	.	10	.
Mercurialis perennis	46	.	.	.	.	1	1	.	1	1	1	.	.	1	.	11	.
Aconitum variegatum	21	.	.	.	.	.	.	.	1	.	.	.	.	.	.	9	.
Aster alpinus	62	.	.	.	.	.	.	.	.	1	1	.	.	3	.	13	3
Asplenium viride	48	.	.	.	.	.	.	.	.	1	1	.	.	1	.	13	6
Convallaria majalis	107	.	1	.	.	1	1	.	2	1	1	.	.	1	.	12	.
Polygala amara agg.	310	12	1	.	1	1	1	1	2	1	1	.	.	11	3	25	6
Hieracium villosum	22	.	.	.	.	.	.	.	1	.	1	.	.	.	.	8	.
Carex alba	63	.	.	.	.	.	1	.	1	1	1	.	.	1	.	11	.
Valeriana tripteris	30	.	.	.	.	.	1	1	.	1	1	.	.	1	.	10	1
Seseli libanotis	339	1	.	.	.	1	1	1	3	5	2	.	.	3	.	22	17
Hieracium bifidum	57	.	.	.	.	1	.	.	.	1	1	.	.	2	1	12	4
Digitalis grandiflora	174	.	.	.	.	1	1	1	2	1	1	.	.	3	.	18	15
Knautia kitaibelii	496	1	1	.	.	5	3	1	11	5	1	.	.	2	.	19	.
Melampyrum sylvaticum	50	.	.	.	.	1	1	.	1	1	.	.	.	.	.	8	.
Gymnocarpium robertianum	34	.	.	.	.	.	.	.	.	.	1	.	.	1	.	9	3
Cirsium erisithales	126	1	.	.	.	1	1	.	2	1	.	.	.	1	.	12	6
Cyanus dominii	12	.	.	.	.	.	.	.	.	.	.	.	.	.	.	5	.
Epipactis atrorubens	84	.	.	.	.	.	1	.	1	1	1	.	.	5	1	11	2
Tofieldia calyculata	73	3	.	.	.	1	1	.	1	1	.	.	.	1	.	9	.
Campanula rapunculoides	607	.	.	.	.	3	4	10	7	7	1	.	.	4	.	20	5
Thymus alpestris	48	.	.	.	.	1	1	.	1	1	1	.	.	1	.	7	.
Draba aizoides	22	.	.	.	.	.	.	.	.	.	1	.	.	1	.	6	.
Melica nutans	68	.	1	.	.	.	1	1	1	1	1	.	.	1	.	8	2
Dianthus nitidus	11	.	.	.	.	.	.	.	.	.	.	.	.	1	.	5	.
Sesleria rigida	94	.	.	.	.	.	.	.	.	1	1	.	.	5	.	.	51
Asplenium trichomanes	271	.	.	.	.	1	.	.	.	1	1	.	.	13	1	20	61
Helictotrichon decorum	95	.	.	.	.	.	.	1	1	1	1	.	.	6	.	.	37
Thymus comosus	217	.	.	.	.	.	.	.	.	1	1	.	.	13	1	1	36
Dianthus spiculifolius	48	.	.	.	.	.	.	.	.	.	.	.	.	3	.	.	23
Poa nemoralis	100	.	.	.	.	1	1	.	1	1	1	.	.	2	.	7	23
Polypodium vulgare	60	.	.	.	.	.	.	.	.	.	1	.	.	3	.	2	18
Sesleria heuflerana	132	.	.	.	.	.	.	.	.	3	1	.	.	3	.	.	18
Asperula capitata	23	.	.	.	.	.	.	.	.	.	.	.	.	1	.	.	13
Arabidopsis arenosa	370	.	1	.	.	1	1	1	1	2	2	.	1	17	2	19	32
Selaginella helvetica	36	.	.	.	.	.	.	.	1	1	1	.	.	1	.	1	13
Seseli gracile	62	.	.	.	.	.	.	.	.	1	1	.	.	3	.	.	15
Pistorinia hispanica	68	.	.	.	.	.	.	.	.	1	1	.	.	3	.	.	14
Geranium robertianum	114	.	1	.	.	.	1	1	1	1	1	.	.	3	.	10	20
Moehringia muscosa	20	.	.	.	.	.	.	.	.	.	.	.	.	1	.	1	12
Dianthus petraeus	20	.	.	.	.	.	.	.	.	.	1	.	.	1	.	.	11
Melica ciliata	685	.	.	.	.	.	1	.	.	1	8	1	.	20	17	4	30
Seseli rigidum	31	.	.	.	.	.	.	.	.	.	1	.	.	2	.	.	11
Cystopteris fragilis	44	.	.	.	.	.	.	.	.	.	1	.	.	1	.	5	14
Festuca arvernensis	38	.	.	.	.	.	.	.	.	1	1	.	1	2	.	.	11
Selinum silaifolium	46	.	.	.	1	.	.	.	1	.	1	.	.	4	.	.	12
Centaurea atropupurea	54	.	.	.	.	.	.	.	.	1	1	.	.	1	.	.	10
Hylotelephium maximum agg.	592	1	.	7	.	1	1	2	1	2	7	2	1	12	.	8	26
Sempervivum marmoreum	79	.	.	.	.	.	.	.	.	.	1	.	.	6	.	.	12
Viola jooi	70	.	.	.	.	.	.	.	.	1	1	.	.	6	.	.	12
Taraxacum sect. Erythrocarpa	23	.	.	.	.	.	.	.	.	1	1	.	.	1	.	.	8
Asplenium ceterach	74	.	.	.	.	.	1	.	1	.	1	.	.	3	.	.	10
Doronicum columnae	7	.	.	.	.	.	.	.	.	.	.	.	.	.	.	.	6
Galium mollugo agg.	4393	10	21	1	15	25	48	41	35	21	7	.	.	22	.	22	57
Potentilla thuringiaca	51	.	.	.	.	1	1	1	1	1	1	.	.	1	.	1	8
Securigera elegans	6	.	.	.	.	.	.	.	.	.	.	.	.	.	.	.	6
Pedicularis comosa	37	.	.	.	.	.	1	1	1	1	1	.	.	1	.	.	7

Cluster number		1	2	3	4	5	6	7	8	9	10	11	12	13	14	15	16
No. of relevés (all)		257	260	213	797	1411	4434	526	1306	1779	5089	201	203	761	429	219	108
- (with NVP recorded)		133	133	51	234	471	1227	173	409	559	1421	46	121	145	59	100	12
Cytisus hirsutus	411	1	.	.	.	1	1	.	7	6	1	.	.	10	1	6	19
Rhamnus saxatilis	50	.	.	.	.	.	.	.	1	1	1	.	.	3	1	.	8
Isatis tinctoria	54	.	.	.	.	.	.	.	.	1	1	.	.	2	.	.	7
Hepatica nobilis	15	.	.	.	.	.	.	.	1	1	.	.	.	1	.	1	6
Spiraea chamaedryfolia	10	.	.	.	.	.	.	.	.	.	.	.	.	1	.	.	6
Scrophularia rupestris	5	.	.	.	.	.	.	.	.	.	.	.	.	.	.	.	5
Saxifraga marginata	5	.	.	.	.	.	.	.	.	.	.	.	.	.	.	.	5
Valeriana montana	5	.	.	.	.	.	.	.	.	.	.	.	.	.	.	.	5
Lactuca muralis	38	.	.	.	.	1	1	1	1	.	1	.	.	1	.	5	8
Succisa pratensis	653	60	27	.	2	6	7	1	2	1	1	.	.	.	.	1	.
Carex panicea	1044	71	62	5	10	12	9	.	3	1	1	.	.	1	.	.	.
Valeriana dioica	228	42	21	.	1	1	1	.	1	.	.	.	.	.	.	.	.
Deschampsia cespitosa	1651	60	63	7	29	17	18	2	1	1	1	.	.	.	.	.	.
Lysimachia vulgaris	571	40	41	2	13	3	5	.	1	1	1	.	.	.	.	.	.
Carex flacca	1128	40	15	.	1	4	7	3	30	11	1	.	.	1	.	1	.
Cirsium canum	606	32	12	.	24	.	6	2	1	1	1	.	.	.	.	1	.
Lythrum salicaria	499	36	33	.	21	1	3	1	1	.	1	.	.	.	.	.	1
Equisetum palustre	462	29	43	.	8	1	4	1	1	.	1	.	.	.	.	.	.
Selinum carvifolia	324	20	18	.	3	1	4	1	1	1	1	.	.	.	.	.	.
Juncus articulatus	236	21	22	1	7	1	2	1	.	1	1	.	.	.	.	.	.
Filipendula ulmaria	456	24	38	4	10	1	4	.	1	.	1	.	.	.	.	.	.
Cirsium rivulare	499	23	47	.	2	2	6	.	1	.	.	.	.	.	.	1	.
Eriophorum latifolium	82	11	10	.	.	.	1	.	.	.	.	.	.	.	.	.	.
Lychnis flos-cuculi	1992	14	67	24	49	8	27	2	1	.	1	.	.	.	.	.	.
Galium palustre agg.	422	19	37	1	23	1	2	.	.	.	.	.	.	.	.	.	.
Poa trivialis	1172	3	40	2	30	3	17	1	1	.	1	.	.	.	.	1	.
Holcus lanatus	2350	17	46	.	12	11	39	5	10	1	1	.	1	.	.	.	.
Lysimachia nummularia	1394	18	43	8	50	2	17	2	2	1	1	.	.	.	.	.	.
Ranunculus repens	1729	31	48	10	66	6	20	1	1	1	1	.	.	.	.	.	.
Cardamine pratensis agg.	718	4	28	.	31	2	8	1	1	.	1	.	.	.	.	.	.
Carex pallescens	1677	4	31	.	4	42	19	1	8	1	1	.	.	.	.	.	.
Agrostis stolonifera	808	21	28	1	33	1	7	3	2	2	1	.	.	.	.	.	.
Carex hirta	1552	9	34	15	38	5	18	7	2	1	2	1	26	.	.	.	.
Agrostis vinealis	503	.	.	55	2	1	1	2	1	1	4	.	25	1	.	.	.
Carex praecox	1059	1	.	56	41	2	3	4	1	1	8	.	10	1	1	.	.
Potentilla argentea	1580	.	.	40	2	1	6	31	3	1	17	4	30	2	.	.	1
Poa pratensis agg.	7367	33	38	78	70	23	60	67	45	36	36	6	17	1	.	1	.
Inula britannica	335	5	1	19	17	1	1	3	1	1	1	.	.	.	.	.	.
Allium angulosum	242	10	2	18	17	.	1	1	1	.	1	.	.	.	.	.	.
Taraxacum sect. Ruderalia	3732	9	13	19	49	18	50	28	22	5	4	1	1	1	1	5	1
Campanula patula	3180	2	6	1	5	62	42	7	22	1	1	.	.	1	.	.	.
Luzula campestris agg.	3587	4	22	3	3	71	39	14	32	4	3	.	7	.	.	.	2
Thymus pulegioides	2926	2	1	.	1	66	17	30	39	14	5	.	5	4	.	5	4
Anthoxanthum odoratum agg.	5066	11	47	1	10	84	60	24	46	4	4	.	7	1	.	.	.
Festuca rubra agg.	4551	27	42	25	13	81	50	19	35	7	3	.	6	1	.	1	.
Carlina acaulis	2151	.	.	.	.	53	5	4	45	20	2	.	.	6	2	26	1
Veronica chamaedrys	4336	2	18	1	7	58	56	30	31	8	4	.	.	2	.	5	5
Trifolium repens	4136	3	13	31	38	59	51	25	16	2	5	.	1	1	.	1	.
Briza media	5178	43	46	1	3	71	43	20	81	40	2	.	.	4	1	6	.
Pimpinella saxifraga agg.	5691	12	6	.	1	68	32	47	63	48	23	1	1	14	4	19	8
Cynosurus cristatus	1940	1	13	1	4	29	29	6	10	1	1	.	.	.	.	.	.
Rhinanthus minor	2411	9	10	6	9	32	30	8	22	4	1	.	.	1	.	1	.
Trifolium pratense	5719	18	25	31	40	58	70	55	51	5	5	.	.	1	.	2	.
Lotus corniculatus	7059	33	21	28	30	66	60	55	75	37	19	.	1	6	5	23	.
Achillea millefolium agg.	10767	35	29	65	40	81	79	84	74	61	56	4	25	6	1	21	6
Trisetum flavescens	2991	1	3	.	1	25	46	13	35	2	1	.	.	.	.	.	.
Dactylis glomerata	5976	27	16	1	11	35	67	46	66	37	11	1	4	1	.	1	1
Daucus carota	3394	18	4	2	13	10	38	48	33	12	10	.	.	1	.	.	.
Fragaria viridis	2734	.	.	2	3	2	9	43	29	33	21	.	.	3	1	1	8
Knautia arvensis	3966	2	5	1	2	38	33	41	45	35	9	1	.	1	.	11	.
Brachypodium pinnatum agg.	2988	5	6	.	.	8	6	13	66	73	6	.	.	6	2	5	.
Salvia pratensis	3886	1	.	.	1	3	15	37	62	58	20	1	.	13	4	3	.
Festuca stricta ssp. sulcata	4125	1	1	.	3	5	13	38	53	60	27	2	7	8	3	.	6
Centaurea scabiosa	2887	.	2	.	1	5	4	17	40	62	15	1	1	14	7	7	.
Sanguisorba minor	3333	.	.	.	1	8	5	15	49	39	20	2	2	32	71	9	.
Prunella grandiflora	649	1	.	.	.	1	1	2	16	17	1	.	.	3	1	4	.
Dorycnium pentaphyllum agg.	1907	1	1	.	.	1	1	10	15	36	15	1	.	10	33	1	1
Medicago falcata	3694	1	3	2	2	1	6	27	33	43	39	8	1	3	1	1	.
Bupleurum falcatum	1149	.	.	.	.	1	1	1	6	27	6	.	.	24	3	12	6
Elytrigia intermedia	1551	.	.	.	.	.	1	3	3	20	22	2	.	2	1	.	.
Inula ensifolia	1273	.	.	.	.	.	1	1	1	27	8	.	.	35	9	16	7
Eryngium campestre	3291	.	.	1	1	1	2	19	5	23	49	35	33	1	4	.	.
Potentilla incana agg.	3528	.	.	.	1	1	1	1	3	21	47	35	30	35	70	1	10
Euphorbia seguieriana	1010	.	.	1	1	.	1	1	1	3	12	68	16	1	36	.	.
Arenaria serpyllifolia agg.	1407	.	.	1	1	1	1	4	2	5	17	45	26	9	33	5	6
Bromus tectorum	167	.	.	.	.	.	1	.	.	.	2	18	17	.	.	.	.
Fumana procumbens	447	.	.	.	.	.	.	.	.	1	1	29	.	4	66	.	.
Cerastium semidecandrum	348	.	.	.	.	1	1	1	.	1	4	22	33	1	2	.	.
Cynodon dactylon	420	.	.	.	4	1	1	3	1	1	4	26	49	.	1	.	.
Erigeron canadensis	322	2	1	3	2	1	1	2	.	1	2	22	40	.	.	.	.
Alyssum montanum	491	.	.	.	.	.	.	.	1	2	4	20	4	11	26	.	1

Cluster number		1	2	3	4	5	6	7	8	9	10	11	12	13	14	15	16
No. of relevés (all)		257	260	213	797	1411	4434	526	1306	1779	5089	201	203	761	429	219	108
- (with NVP recorded)		133	133	51	234	471	1227	173	409	559	1421	46	121	145	59	100	12
Festuca pallens agg.	1130	.	.	.	.	.	1	1	.	2	6	.	1	56	75	25	7
Carex humilis	2644	.	.	.	.	1	1	1	4	30	23	1	1	60	83	26	5
Teucrium montanum	1591	.	.	.	.	1	1	.	3	7	13	2	.	50	73	21	6
Anthericum ramosum	1853	3	.	.	.	1	1	1	11	31	9	1	.	50	47	38	14
Seseli osseum	1115	.	.	.	.	.	.	1	.	3	12	2	1	36	23	28	12
Erysimum odoratum	460	.	.	.	.	.	1	1	1	4	3	.	.	21	3	4	19
Sesleria caerulea	503	1	.	.	.	.	1	1	1	2	1	.	.	29	5	84	.
Polygonatum odoratum	499	.	.	.	.	1	1	.	3	7	2	1	1	21	8	23	6
Leontodon incanus	467	.	.	.	.	1	1	1	2	2	1	.	.	23	27	39	.
Saxifraga paniculata	171	.	.	.	.	.	.	.	.	.	1	.	.	8	.	28	35
Asplenium ruta-muraria	455	.	.	.	.	.	.	.	.	1	2	.	.	26	6	37	59
Campanula carpatica	60	.	.	.	.	.	1	.	.	.	.	.	.	2	.	15	12
Potentilla erecta	2747	63	52	7	2	84	21	1	21	1	1	.	.	.	.	1	1
Rumex acetosa	4547	11	52	19	34	59	62	13	23	2	1	1	4	1	1	.	.
Leontodon hispidus	5162	30	7	.	6	62	51	34	63	25	8	1	4	3	1	6	1
Leucanthemum vulgare agg.	5790	28	23	8	23	69	62	40	65	21	2	.	.	9	1	38	4
Plantago lanceolata	8064	30	27	48	38	76	76	61	72	23	27	1	18	2	.	2	2
Arrhenatherum elatius	4244	4	2	.	4	15	49	40	50	26	10	.	2	1	.	1	.
Plantago media	5467	7	6	13	11	32	30	54	73	54	25	.	.	7	1	7	1
Teucrium chamaedrys	4318	1	.	.	.	1	1	8	28	55	47	8	7	46	19	19	14
Vincetoxicum hirundinaria	1315	.	.	.	1	1	1	2	3	15	8	2	1	42	18	45	41
Jovibarba globifera	727	.	.	.	.	.	1	.	.	2	4	.	.	35	30	49	13
Ranunculus acris	5103	71	76	34	52	62	69	9	16	1	1	.	.	.	.	1	.
Galium verum	6406	50	45	46	23	20	40	54	52	49	38	24	8	1	3	2	.
Euphorbia cyparissias	5729	1	1	.	1	21	9	29	48	54	48	18	46	56	38	43	22
Hypericum perforatum	3707	1	1	1	1	16	16	36	35	27	28	5	33	8	6	9	8
Prunella vulgaris	3635	44	38	27	33	46	41	20	25	6	2	.	1	.	.	1	.
Vicia cracca	3385	26	22	17	28	32	40	21	34	8	2	.	.	1	.	9	.
Dianthus carthusianorum agg.	2772	1	.	.	.	11	6	12	30	33	20	9	10	21	9	12	19
Medicago lupulina	2552	3	4	19	13	4	22	27	28	12	12	1	1	2	.	3	2
Stachys recta	2466	1	.	.	1	1	1	2	5	31	30	5	.	29	11	8	19
Phleum pratense agg.	1699	5	11	16	9	21	24	11	5	1	1	.	.	.	.	1	.
Colchicum autumnale	1605	13	15	.	10	9	22	5	20	2	1	.	.	.	.	.	.
Scorzoneroides autumnalis	1578	14	11	18	22	22	18	5	5	2	1	.	.	1	.	1	.
Phleum phleoides	1442	.	.	.	.	1	1	3	7	21	17	13	1	7	1	.	3
Echium vulgare	1440	.	.	1	.	1	2	8	5	9	19	1	7	11	4	2	2
Veronica spicata	1432	1	.	3	1	1	1	5	6	15	17	7	2	9	14	1	.
Clinopodium acinos	1415	.	.	.	1	1	1	7	3	6	18	11	1	16	22	8	6
Pilosella bauhini	1411	.	.	.	1	7	4	12	17	12	9	.	.	11	22	3	.
Campanula sibirica	1365	1	.	.	.	.	1	4	1	12	16	.	.	22	24	1	19
Carlina vulgaris agg.	1317	1	.	.	.	10	2	12	15	23	7	.	3	6	3	9	2
Fragaria vesca	1285	1	1	.	2	24	9	10	14	6	2	.	.	2	.	18	7
Senecio jacobaea	1188	.	2	.	5	3	4	16	9	6	12	1	1	1	3	.	.
Galium glaucum	1127	.	.	.	.	1	1	1	2	15	13	.	.	12	7	11	3
Clinopodium vulgare	1112	1	.	.	1	3	9	13	15	10	4	.	.	2	.	7	2
Thalictrum minus	1104	1	.	.	.	1	1	3	3	16	12	1	.	14	9	10	3
Genista tinctoria	1039	11	1	3	1	8	4	10	12	10	6	.	.	4	1	1	.
Silene vulgaris	1011	.	1	.	1	5	7	9	14	10	3	1	3	4	1	4	7
Calamagrostis epigejos	997	7	7	25	4	2	5	14	6	9	6	8	8	.	1	.	1
Trifolium alpestre	952	1	1	1	1	4	3	4	12	14	6	.	.	2	.	1	1
Picris hieracioides	944	1	5	1	2	1	5	13	9	10	6	.	1	.	.	1	.
Pastinaca sativa	940	5	1	.	5	1	14	16	5	3	1	.	.	.	.	.	.
Pimpinella major	935	5	2	.	2	10	15	1	3	1	1	.	.	1	.	16	1
Sedum acre	921	1	.	12	1	1	1	2	1	2	12	2	19	12	2	6	8
Veronica austriaca	876	.	1	.	.	1	1	3	10	13	8	1	.	9	.	5	10
Origanum vulgare	869	.	.	.	.	1	3	6	7	15	5	.	.	7	.	12	8
Carex tomentosa	833	10	17	.	10	2	6	3	14	8	1	.	.	.	1	.	.
Erigeron annuus	825	1	2	6	3	1	11	10	5	2	2	.	3	1	.	.	.
Bromus hordeaceus	825	1	2	6	11	1	10	4	1	1	4	.	8	.	.	1	.
Arabis hirsuta agg.	808	1	.	.	1	1	2	3	14	10	4	.	.	12	4	14	7
Trifolium dubium	802	2	3	2	5	8	12	6	4	1	1	.	.	.	.	.	.
Bromus inermis	784	1	1	10	2	1	1	6	1	6	10	1	.	.	.	.	.
Hypochaeris radicata	779	1	1	.	1	16	8	3	2	1	2	.	16	.	.	1	.
Poa compressa	749	1	1	1	1	1	2	12	3	6	8	.	3	2	1	2	3
Peucedanum oreoselinum	743	1	.	.	1	1	2	1	15	12	3	13	3	6	3	1	3
Geranium sanguineum	740	.	.	.	.	1	1	1	8	16	4	.	.	12	.	9	9
Centaurea phrygia	739	2	3	.	.	14	9	2	6	2	1	.	.	1	.	.	.
Veronica arvensis	737	1	1	2	6	1	8	9	5	1	4	1	2	1	2	.	.
Lolium perenne	730	.	.	1	7	2	11	7	1	1	2	.	1	.	.	.	.
Festuca ovina agg.	717	2	2	1	.	8	4	3	7	4	3	1	16	2	.	1	.
Verbascum lychnitis	708	.	.	1	.	.	1	2	1	3	11	15	1	6	.	.	9
Galatella linosyris	701	.	.	.	.	.	.	1	1	13	8	14	.	1	6	.	.
Tanacetum corymbosum	698	.	.	.	1	5	1	1	11	15	2	.	.	5	1	9	4
Silene nutans	686	.	.	.	.	9	4	4	14	4	1	.	.	5	.	2	18
Cytisus austriacus	682	.	.	.	.	.	.	1	1	10	10	1	.	1	.	.	1
Linaria genistifolia	659	.	.	.	.	.	.	.	.	1	10	12	16	6	5	.	6
Gymnadenia conopsea	646	9	2	.	.	17	3	1	12	3	1	.	.	2	.	8	.
Viscaria vulgaris	617	.	1	1	.	6	4	3	4	1	4	.	1	2	.	.	2
Hypochaeris maculata	581	.	.	.	.	4	1	1	12	9	3	.	.	1	.	.	.
Veronica prostrata	580	.	.	.	.	1	1	3	2	2	9	.	5	2	1	.	.

Cluster number		1	2	3	4	5	6	7	8	9	10	11	12	13	14	15	16
No. of relevés (all)		257	260	213	797	1411	4434	526	1306	1779	5089	201	203	761	429	219	108
- (with NVP recorded)		133	133	51	234	471	1227	173	409	559	1421	46	121	145	59	100	12
Cytisus nigricans	552	.	.	.	.	1	1	1	2	14	3	.	.	11	1	2	6
Stipa pulcherrima	551	.	.	.	.	.	.	.	1	3	8	.	.	5	8	.	.
Verbascum chaixii	550	.	.	.	.	1	1	3	1	7	6	.	1	7	1	2	7
Carex muricata agg.	545	1	3	1	7	1	6	12	1	1	1	.	1	1	.	1	3
Dianthus deltoides	538	.	.	9	1	11	6	6	2	1	1	.	1	.	.	.	.
Prunella laciniata	530	.	.	.	1	1	2	9	12	6	2	.	.	1	.	.	.
Inula salicina	515	15	2	2	7	1	3	3	8	6	1	2	.	1	.	1	.
Primula elatior	515	1	7	.	.	12	6	1	2	1	1	.	.	1	.	3	1
Euphrasia stricta agg.	513	1	1	4	1	5	2	8	2	3	4	.	.	2	1	1	.
Alyssum alyssoides	504	.	.	.	.	1	.	1	1	1	8	9	7	3	2	.	.
Hippocrepis comosa	495	.	.	.	.	1	1	1	11	8	1	.	.	11	7	12	.
Myosotis arvensis	488	.	.	1	1	1	6	6	5	1	1	.	1	.	1	.	.
Viola ambigua	487	.	.	.	.	.	1	1	1	5	8	.	.	1	1	.	.
Campanula persicifolia	480	1	.	.	.	6	2	2	10	5	1	.	.	2	.	6	10
Rostraria cristata	476	.	.	.	.	1	1	1	1	7	5	6	.	1	12	.	.
Rhinanthus angustifolius	469	2	2	15	4	4	3	2	5	4	1	1	1	1	.	4	.
Helictochloa pratensis	468	.	.	.	.	1	1	2	2	7	5	.	1	1	1	.	.
Vicia hirsuta	468	.	1	.	4	1	4	9	2	1	3	1	.	1	.	.	.
Linaria vulgaris	456	.	1	5	1	2	2	8	1	2	4	.	1	1	.	1	3
Ajuga genevensis	434	.	.	.	.	2	2	6	3	3	4	1	1	2	.	3	2
Berteroa incana	432	.	.	5	1	.	1	4	.	1	7	2	7	1	.	.	.
Chrysopogon gryllus	423	.	.	.	.	.	1	2	1	2	6	3	.	1	11	.	.
Cerastium arvense	417	.	1	1	1	1	2	5	3	5	2	.	7	2	.	2	4
Veronica serpyllifolia	417	1	4	.	11	2	6	1	1	.	1	.	1	.	.	.	.
Geranium pratense	416	1	2	7	5	1	7	2	1	1	1	.	.	.	.	.	.
Valeriana officinalis	414	5	13	.	4	1	2	3	5	3	1	.	.	3	.	1	16
Microthlaspi perfoliatum	411	.	.	.	.	.	1	1	2	3	5	2	1	2	8	.	.
Muscari comosum	399	.	.	.	.	1	1	3	3	4	5	1	.	.	.	.	.
Achillea nobilis	398	.	.	.	.	1	1	2	1	1	7	1	1	1	.	.	.
Anthemis tinctoria	382	.	.	.	.	1	1	6	1	3	5	.	.	1	.	1	1
Melilotus officinalis	382	.	.	.	.	.	1	4	1	3	5	1	.	1	.	.	.
Iris pumila	375	.	.	.	.	.	.	.	.	1	7	1	.	1	3	.	2
Cleistogenes serotina	375	.	.	.	.	.	.	.	.	.	7	3	.	1	1	.	.
Knautia drymeia	374	2	1	.	1	1	4	2	10	2	1	.	.	.	.	1	.
Solidago virgaurea	361	1	.	.	1	8	2	3	3	3	1	4	2	1	.	3	8
Viola tricolor	361	.	.	.	.	5	3	1	1	1	2	.	2	3	.	1	11
Melica transsilvanica	357	.	.	.	.	.	.	1	.	2	6	5	.	2	.	4	.
Tragopogon dubius	352	.	.	1	1	.	1	2	1	1	6	4	1	1	.	.	.
Potentilla recta	351	.	.	1	.	1	1	2	4	3	4	.	.	2	.	.	.
Centaurium erythraea	349	4	.	1	2	1	4	4	2	1	1	.	1	.	.	.	.
Viola arvensis	348	.	.	1	.	1	2	1	1	1	4	5	4	1	1	1	.
Artemisia absinthium	346	.	.	.	1	.	1	5	.	1	5	.	1	.	.	.	.
Carduus acanthoides	342	.	.	1	1	1	1	4	1	2	4	.	.	.	1	.	.
Erigeron acris	329	.	.	2	1	1	2	11	3	2	2	.	4	1	.	1	1
Linum austriacum	326	.	.	.	.	.	1	1	.	2	6	1	1	1	1	.	.
Astrantia major	325	1	2	.	1	8	3	.	4	1	.	.	.	.	.	6	.
Cuscuta epithymum	320	1	1	.	1	2	2	1	3	3	2	.	.	1	1	1	.
Cirsium vulgare	319	1	1	.	2	2	3	6	1	1	2	.	1	.	.	.	.
Astragalus monspessulanus	317	.	.	.	.	.	.	1	1	3	5	.	.	2	1	.	.
Trifolium patens	316	2	5	.	2	1	6	1	1	.	1	.	.	.	.	.	.
Cytisus albus	316	.	.	.	.	.	.	.	2	8	3	.	.	2	.	.	1
Silene latifolia	310	1	.	3	1	1	2	3	1	1	3	1	1	1	.	.	.
Pulsatilla pratensis	309	.	.	.	.	.	.	1	1	3	5	4	1	.	1	.	.
Vinca herbacea	304	.	.	.	.	.	.	.	.	1	5	1	.	1	2	.	1
Artemisia vulgaris	300	.	.	1	2	1	3	7	1	1	2	.	.	.	.	.	.
Leontodon crispus	295	.	.	.	.	1	.	1	1	3	4	.	.	6	1	.	6
Odontites vulgaris agg.	295	5	1	1	5	1	2	5	1	1	2	.	.	1	1	.	.
Aegopodium podagraria	292	3	3	.	2	1	5	1	1	1	1	.	.	.	.	1	.
Lathyrus tuberosus	285	.	.	1	1	.	2	10	3	2	2	.	.	.	.	.	.
Vicia tenuifolia	285	1	.	.	.	1	1	1	5	6	1	.	.	.	.	.	.
Allium oleraceum	285	.	.	1	1	1	2	8	2	2	2	.	.	1	.	1	1
Linum hirsutum	282	.	.	.	.	.	.	1	1	4	4	3	.	1	.	.	.
Reseda lutea	269	.	.	.	1	.	1	1	.	2	4	.	.	1	4	.	.
Medicago x varia	269	.	1	.	1	.	3	5	3	1	1	.	1	.	.	.	.
Asperula tinctoria	268	1	.	.	.	1	1	.	2	3	1	.	.	12	3	13	.
Platanthera bifolia	267	.	1	.	.	10	1	.	2	1	1	.	.	2	.	9	1
Chaerophyllum aromaticum	265	.	2	.	1	4	4	1	1	1	.	.	.	.	.	.	.
Urtica dioica	264	1	2	.	5	1	4	2	1	1	1	.	.	1	.	1	2
Centaurea oxylepis	263	1	1	.	1	7	3	1	2	1	1	.	.	.	.	.	.
Biscutella laevigata	260	.	.	.	.	1	1	.	1	1	1	.	.	15	9	11	7
Petrorhagia saxifraga	256	.	.	.	.	1	1	1	1	1	3	4	6	1	3	.	.
Cerastium brachypetalum	254	.	.	.	1	.	1	2	3	1	2	.	.	1	7	.	.
Festuca pseudodalmatica	253	.	.	1	1	.	1	.	.	1	4	.	1	3	.	.	1
Thesium arvense	251	.	.	.	.	.	1	1	1	2	4	8	1	1	1	.	.
Seseli pallasii	250	.	.	.	.	.	1	.	1	2	3	1	.	4	1	.	.
Anemone sylvestris	246	.	.	.	.	1	1	1	1	7	2	.	.	1	.	1	.
Myosotis ramosissima	246	1	.	.	2	.	2	3	1	1	2	.	1	1	1	.	.
Taraxacum sect. Erythrosperma	245	.	.	.	.	1	1	1	1	1	3	10	1	3	6	1	.
Dictamnus albus	241	.	.	.	.	.	.	.	1	4	3	.	.	2	1	.	1
Bromus racemosus	239	.	2	.	4	.	4	1	1	.	1	.	.	.	.	.	.
Potentilla pusilla agg.	238	.	.	.	.	1	1	2	3	3	1	.	8	1	.	2	.

Cluster number		1	2	3	4	5	6	7	8	9	10	11	12	13	14	15	16
No. of relevés (all)		257	260	213	797	1411	4434	526	1306	1779	5089	201	203	761	429	219	108
- (with NVP recorded)		133	133	51	234	471	1227	173	409	559	1421	46	121	145	59	100	12
Tanacetum vulgare	230	2	2	4	2	1	3	3	1	1	1	.	1	.	.	.	.
Muscari tenuiflorum	228	.	.	.	.	.	1	1	1	1	4	.	.	1	1	.	.
Cytisus ruthenicus	228	.	.	.	.	.	1	5	1	4	2	1	1	.	.	.	.
Neottia ovata	227	1	3	.	.	5	2	.	3	.	.	.	.	.	.	1	.
Muscari neglectum	226	.	.	.	.	.	.	1	1	1	3	1	1	1	8	.	.
Trifolium ochroleucon	224	.	1	.	.	2	1	6	5	1	1	.	.	.	.	.	.
Trifolium rubens	223	.	5	.	1	2	1	1	5	3	1	.	.	1	.	.	.
Cephalaria uralensis	223	.	.	.	.	.	.	.	.	1	4	1	.	1	.	.	.
Allium carinatum	222	7	1	.	1	1	2	.	5	1	1	.	.	1	.	.	.
Cruciata laevipes	220	.	1	.	1	1	3	2	2	1	1	.	.	.	.	1	.
Stipa lessingiana	220	.	.	.	.	.	.	.	.	1	4	.	.	.	.	.	.
Pilosella cymosa	220	.	1	.	.	1	1	1	1	1	3	1	2	3	1	.	.
Veronica orchidea	218	.	.	.	.	1	1	1	2	2	3	.	.	1	1	.	2
Rhinanthus rumelicus	216	.	.	.	1	.	1	6	2	2	1	.	.	.	.	.	.
Odontites luteus	215	.	.	.	.	.	.	.	1	3	3	.	.	1	6	.	.
Astragalus glycyphyllos	212	.	.	.	.	1	1	5	3	3	1	.	1	1	.	.	.
Anacamptis morio	211	.	.	.	.	1	1	1	6	1	1	.	.	.	.	.	.
Campanula bononiensis	210	.	.	.	.	.	1	1	1	5	2	.	.	1	1	.	.
Festuca arundinacea	210	3	2	4	4	1	3	3	1	1	1	.	.	.	.	.	.
Rosa gallica	210	.	.	.	.	1	1	1	3	3	2	.	.	.	.	.	.
Allium scorodoprasum	208	1	1	.	1	1	2	2	4	1	1	.	.	1	1	1	.
Scorzonera hispanica	206	.	.	.	.	.	1	.	1	5	2	.	.	.	1	.	1
Melampyrum nemorosum	204	.	1	.	.	1	2	1	4	2	1	.	.	1	.	4	3
Galium aparine	204	1	6	1	4	1	2	1	1	1	1	2	.	1	.	1	.
Salvia austriaca	204	.	.	.	.	.	1	2	1	2	3	.	.	1	.	.	.
Oxytropis pilosa	204	.	.	.	.	.	.	.	.	2	3	1	.	1	1	.	.
Hypericum elegans	203	.	.	.	.	.	1	1	.	1	3	1	.	1	.	.	1
Allium sphaerocephalon	201	.	.	.	.	.	.	1	.	1	3	1	.	1	5	.	.
Carduus nutans	199	1	.	.	1	1	1	3	1	1	3	3	1	1	1	.	.
Galium octonarium	198	.	.	.	.	.	.	.	.	1	4	.	.	.	.	.	.
Cytisus ratisbonensis	198	.	.	.	.	.	1	.	1	5	1	1	1	1	3	.	.
Viola rupestris	198	.	.	.	.	.	1	1	1	4	1	1	.	1	5	2	.
Saxifraga tridactylites	195	.	.	.	.	.	1	.	.	1	2	1	1	4	11	1	6
Potentilla inclinata	193	.	.	3	1	1	1	1	1	1	3	.	.	1	.	.	.
Phlomis tuberosa	193	.	.	.	.	.	.	1	1	1	3	.	.	.	.	.	.
Capsella bursa-pastoris	193	.	1	2	3	.	3	1	1	1	1	.	1	1	.	.	.
Gentiana cruciata	189	.	.	.	.	1	1	3	5	4	1	.	.	1	.	1	.
Primula acaulis	188	1	.	.	.	1	2	.	5	1	.	.	.	1	.	1	.
Ochlopoa annua	187	.	.	.	3	1	3	.	1	1	1	.	.	.	.	.	.
Astragalus cicer	185	.	.	.	1	.	1	7	2	3	1	.	.	.	.	.	.
Medicago prostrata	181	.	.	.	.	.	1	1	1	2	2	.	.	2	5	.	.
Podospermum purpureum	181	.	.	.	.	1	1	1	1	4	1	1	.	3	1	.	.
Arabidopsis thaliana	180	.	.	1	1	1	1	1	1	1	2	1	1	1	3	2	1
Tussilago farfara	180	2	6	.	1	1	2	1	1	1	1	.	.	.	.	1	.
Moenchia mantica	178	1	2	.	1	1	3	2	1	1	1	.	.	.	.	.	.
Lathyrus latifolius	178	.	.	.	.	1	1	.	6	4	1	.	.	.	.	.	.
Teucrium polium	178	.	.	.	.	.	.	.	.	1	3	2	.	.	.	.	.
Marrubium peregrinum	178	.	.	.	.	.	1	1	.	1	3	.	.	.	.	.	.
Dianthus armeria	176	.	.	1	1	1	2	5	1	1	1	.	1	.	.	.	.
Petrorhagia prolifera	176	.	.	.	.	.	1	1	.	1	3	1	3	1	1	.	.
Scabiosa columbaria	175	1	.	.	.	1	1	1	3	2	1	.	.	4	.	1	7
Rhinanthus alectorolophus	175	.	.	1	1	1	3	1	2	1	1	.	.	.	.	4	.
Senecio erucifolius	174	.	.	.	1	1	1	2	1	2	2	1	.	1	.	.	.
Vicia villosa	173	1	.	1	1	1	1	1	1	1	2	.	1	.	.	.	.
Veronica praecox	172	.	.	.	.	.	.	.	1	1	3	.	.	1	4	.	.
Trollius europaeus	171	4	3	.	.	3	2	.	1	.	.	.	.	.	.	.	.
Anthyllis macrocephala	171	.	.	1	.	.	1	5	1	1	2	.	.	.	.	.	.
Fragaria moschata	170	.	.	.	1	1	1	1	5	2	1	.	.	1	.	1	.
Seseli tortuosum	169	.	.	.	.	.	.	1	.	1	3	.	.	.	.	.	.
Anemone nemorosa	168	1	4	.	.	4	2	.	1	1	1	.	.	1	.	1	.
Sideritis montana	165	.	.	.	.	.	.	.	.	.	3	.	.	1	1	.	.
Cirsium eriophorum	165	.	1	.	.	3	1	1	3	1	1	.	.	.	.	.	.
Geranium columbinum	163	.	.	.	.	1	1	2	1	1	2	.	.	1	.	.	.
Verbascum nigrum	162	.	1	.	1	1	1	1	1	1	2	.	.	1	.	1	4
Ornithogalum kochii	162	1	.	.	1	.	1	1	1	1	2	.	.	1	1	.	.
Centaurea diffusa	161	.	.	1	.	.	1	1	.	1	3	1	.	.	.	.	.
Pulmonaria mollis	161	.	.	.	1	.	1	2	4	3	1	.	.	.	.	.	.
Cruciata pedemontana	161	.	.	.	1	.	1	1	.	1	3	.	.	.	1	.	.
Ophioglossum vulgatum	156	4	5	.	3	1	2	.	1	.	.	.	.	.	.	.	.
Fallopia convolvulus	151	.	.	1	1	1	1	2	1	1	2	1	1	1	.	1	1
Galium lucidum	149	.	.	.	.	.	1	1	1	1	1	.	.	2	7	1	.
Aquilegia vulgaris	149	.	1	.	.	2	1	.	5	1	1	.	.	1	.	4	.
Ajuga laxmannii	148	.	.	.	.	.	.	.	.	1	3	.	.	.	.	.	.
Convolvulus cantabrica	147	1	.	.	2	.	1	1	.	1	2	.	.	1	3	.	.
Campanula serrata	145	.	.	.	.	6	1	.	1	.	.	.	.	.	.	1	.
Lepidium campestre	144	.	.	.	.	.	1	1	1	1	2	.	.	1	1	.	.
Bromus riparius	143	.	.	.	.	.	.	.	.	1	3	.	.	1	.	.	1
Helictochloa praeusta	142	.	.	.	.	1	1	1	1	6	1	1	.	.	1	.	.
Pontechium maculatum	142	.	.	.	.	.	.	1	2	3	1	.	.	1	.	.	.
Iris aphylla	142	.	.	.	.	.	.	.	1	2	2	.	.	1	.	.	.
Rumex obtusifolius	142	.	1	.	2	1	3	.	1	.	1	.	.	.	.	.	.

Cluster number		1	2	3	4	5	6	7	8	9	10	11	12	13	14	15	16
No. of relevés (all)		257	260	213	797	1411	4434	526	1306	1779	5089	201	203	761	429	219	108
- (with NVP recorded)		133	133	51	234	471	1227	173	409	559	1421	46	121	145	59	100	12
Verbena officinalis	141	.	1	.	4	1	1	3	1	1	1	1	.	.	.	.	.
Chaerophyllum hirsutum	139	.	8	.	1	2	1	1	1	1	.	.	.	1	.	1	2
Trifolium aureum	139	.	1	1	1	2	1	3	1	1	1	.	.	.	.	.	.
Cerinthe minor	139	.	.	.	1	.	1	1	2	2	1	.	.	1	.	.	.
Ajuga chamaepitys	138	.	.	.	1	.	1	1	.	.	3	1	.	.	.	.	.
Ranunculus flammula	136	7	9	3	7	1	1	.	1	.	.	.	.	.	.	.	.
Plantago argentea	135	.	.	.	.	.	1	1	1	1	1	.	.	3	3	.	.
Prunus fruticosa	135	.	.	.	.	.	.	.	1	3	1	.	.	1	1	.	.
Minuartia rubra	135	.	.	.	.	1	1	1	1	2	1	1	.	1	6	.	.
Neotinea ustulata	135	.	.	.	.	1	1	.	4	2	1	1	.	1	.	.	.
Viola collina	135	.	.	.	.	1	1	1	1	2	1	.	.	4	1	2	.
Orlaya grandiflora	134	.	.	.	.	.	.	1	.	1	2	.	.	1	1	.	1
Stachys germanica	133	.	.	.	.	.	1	2	1	2	1	.	.	.	.	.	.
Geranium sylvaticum	133	.	1	.	.	3	2	.	1	1	.	.	.	.	.	1	.
Thalictrum lucidum	133	2	3	4	6	1	1	1	1	1	1	.	.	.	.	.	.
Verbascum thapsus agg.	132	.	.	.	.	1	1	1	1	1	2	.	1	1	.	1	3
Verbascum phlomoides	130	.	.	1	1	.	1	1	.	1	2	1	4	1	.	.	.
Allium vineale	129	.	.	.	1	.	1	4	1	1	1	.	.	.	1	.	1
Carex sylvatica	129	.	4	.	1	1	2	1	1	.	.	.	.	.	.	.	.
Erodium cicutarium	129	.	.	.	1	.	1	1	1	.	2	1	3	.	.	.	.
Phlomis pungens	128	.	.	.	.	.	.	.	.	.	2	1	.	.	.	.	.
Euphorbia epithymoides	126	.	.	.	.	.	1	.	1	2	1	.	.	4	.	4	3
Lactuca serriola	125	.	.	.	1	.	1	1	1	1	2	1	1	.	.	.	.
Veronica incana	123	.	.	.	.	1	1	1	1	1	2	.	.	.	.	.	.
Stipa tirsa	123	.	.	.	.	.	.	.	1	3	1	.	.	.	.	.	.
Oenothera biennis agg.	123	.	.	5	1	.	1	1	.	.	1	4	9	.	.	.	.
Crocus vernus	123	.	.	.	.	3	2	.	1	.	.	.	.	.	.	.	.
Dactylorhiza maculata	122	3	5	1	1	3	1	1	1	1	.	.	.	.	.	.	.
Hieracium sabaudum	122	.	.	.	1	1	1	1	1	2	1	.	.	1	.	1	1
Inula oculus-christi	121	.	.	.	.	.	.	.	.	1	2	.	.	1	1	.	.
Polygonum bistorta	121	2	7	.	1	1	2	.	.	.	.	.	.	.	.	1	.
Geum urbanum	120	.	1	1	1	1	2	2	1	1	1	.	.	1	.	.	.
Lactuca perennis	120	.	.	.	.	.	.	.	.	1	2	.	.	4	.	1	1
Salvia transylvanica	120	.	.	.	.	.	1	1	1	1	2	.	.	.	.	.	.
Artemisia pontica	120	.	.	.	.	.	.	.	.	1	2	.	.	1	.	.	.
Orobanche gracilis	119	1	.	.	.	.	1	.	4	1	1	.	.	1	1	.	.
Campanula trachelium	119	.	.	.	.	1	1	1	2	1	1	.	.	1	.	5	5
Senecio erraticus	119	7	2	.	6	1	1	1	1	1	1	.	.	.	.	.	.
Crepis tectorum	118	.	.	11	1	1	1	1	.	1	1	6	8	.	.	.	1
Clematis integrifolia	117	1	.	.	3	.	1	2	1	1	1	.	.	.	.	.	.
Ornithogalum umbellatum	117	.	.	.	1	.	1	3	1	1	1	1	2	1	1	.	.
Sonchus arvensis	117	1	1	2	3	1	1	5	.	1	1	.	.	.	.	.	.
Calystegia sepium	117	2	4	1	3	.	2	1	1	.	.	.	.	.	.	.	.
Eupatorium cannabinum	116	7	10	1	1	1	1	1	1	1	1	.	.	1	.	1	1
Cardaria draba	114	.	.	.	1	.	1	1	.	.	1	.	.	.	.	.	.
Orchis militaris	114	1	.	.	.	.	1	.	2	3	1	.	.	1	1	.	.
Lathyrus pannonicus	114	6	.	.	1	.	1	1	1	2	1	.	.	.	.	.	.
Pulsatilla montana	114	.	.	.	.	.	.	.	1	2	1	.	.	1	.	.	.
Xeranthemum annuum	113	.	.	.	.	.	1	.	.	.	2	.	.	1	.	.	.
Viola odorata	112	.	.	.	.	1	1	1	1	2	1	.	.	1	.	.	.
Iris sibirica	111	6	7	1	6	.	1	.	.	.	.	.	.	.	.	.	.
Asyneuma canescens	111	.	.	.	.	.	.	.	1	1	2	.	.	1	.	.	.
Crambe tataria	111	.	.	.	.	.	.	.	.	1	2	.	.	.	.	.	.
Valerianella locusta	111	.	.	.	1	.	1	2	1	1	1	.	.	1	1	.	.
Tripleurospermum inodorum	111	.	1	1	4	.	1	1	.	1	1	1	.	.	.	.	.
Galatella villosa	110	.	.	.	.	.	.	.	.	.	2	.	.	.	.	.	.
Lathyrus sylvestris	110	1	.	.	1	2	1	1	2	1	1	.	.	.	.	.	.
Scorzonera humilis	110	5	2	.	1	1	2	.	1	1	.	.	.	1	.	.	.
Genista januensis	109	.	.	.	.	.	.	.	2	2	1	.	.	3	.	.	2
Lilium martagon	109	.	1	.	.	1	1	.	3	1	.	.	.	1	.	9	3
Brachypodium sylvaticum	108	.	2	.	1	1	1	2	2	1	1	.	.	1	.	1	2
Carduus hamulosus	108	.	.	.	.	.	1	1	1	1	2	.	.	.	.	.	.
Pilosella piloselloides	108	.	.	.	.	1	1	1	1	1	2	.	.	1	.	1	.
Veratrum album	108	5	1	.	.	2	1	.	1	.	.	.	.	.	.	.	.
Tephroseris integrifolia	107	.	.	.	.	1	1	1	1	2	1	2	.	3	.	2	.
Klasea radiata	107	.	.	.	.	.	.	.	1	1	2	.	.	.	.	.	.
Solidago gigantea	107	3	7	.	2	1	1	1	1	1	1	.	.	.	.	.	.
Centaurea nigrescens	105	1	7	.	1	.	1	.	1	1	.	.	.	.	.	.	.
Asarum europaeum	105	.	2	.	1	1	1	.	2	1	1	.	.	1	.	2	3
Clematis recta	105	1	.	.	.	.	1	.	3	2	1	.	.	1	.	.	.
Stellaria media	104	.	1	.	2	1	2	1	1	.	1	.	.	.	.	.	.
Arabis auriculata	104	.	.	.	.	1	1	.	1	1	1	1	.	1	4	.	.
Myosotis sylvatica agg.	104	.	1	.	1	1	1	1	1	1	1	.	.	1	.	2	1
Arabis glabra	104	.	.	.	.	1	1	1	1	1	1	.	.	1	.	.	.
Hieracium virosum	104	.	.	.	.	.	.	.	.	1	2	.	.	1	.	.	.
Thalictrum flavum	103	5	1	2	7	.	1	.	1	.	1	.	.	.	.	.	.
Setaria viridis	102	1	.	.	1	1	1	1	.	.	1	4	4	.	.	.	.
Centaurea macroptilon	101	.	.	.	1	1	2	1	1	1	.	.	.	.	.	.	.
Polygonum aviculare agg.	101	.	.	1	3	.	1	1	1	1	1	1	1	.	.	.	.
Cynoglossum officinale	100	.	.	.	.	.	1	1	1	1	2	2	.	1	.	.	.
Neotinea tridentata	100	.	.	.	.	.	1	.	5	1	1	.	.	1	1	.	.

Cluster number		1	2	3	4	5	6	7	8	9	10	11	12	13	14	15	16
No. of relevés (all)		257	260	213	797	1411	4434	526	1306	1779	5089	201	203	761	429	219	108
- (with NVP recorded)		133	133	51	234	471	1227	173	409	559	1421	46	121	145	59	100	12
Carex acutiformis	99	7	8	.	4	.	1	1	.	.	.	.	.	.	.	.	.
Calamagrostis arundinacea	99	1	.	.	.	3	1	.	2	1	1	.	.	1	.	1	2
Equisetum sylvaticum	99	.	2	.	.	3	1	.	.	.	1	.	.	.	.	.	.
Gentianella lutescens	98	.	.	.	.	4	1	.	1	1	.	.	.	1	.	7	.
Traunsteinera globosa	98	.	.	.	.	3	1	.	3	1	.	.	.	.	.	1	.
Asplenium septentrionale	97	.	.	.	.	.	.	.	.	.	1	.	.	6	.	.	6
Silene bupleuroides	97	.	2	.	1	.	.	.	.	1	1	2	.	1	1	1	.
Astragalus dasyanthus	96	.	.	.	.	.	.	.	.	.	2	2	.	.	.	.	.
Melampyrum cristatum	96	1	.	1	.	1	1	.	2	2	1	.	.	1	1	.	.
Globularia cordifolia	96	.	.	.	.	.	.	.	1	1	1	.	.	3	6	3	.
Polygala sibirica	95	.	.	.	.	.	.	.	.	1	2	.	.	.	.	.	.
Camelina sativa agg.	95	.	.	.	.	.	1	.	.	1	1	5	.	1	1	.	.
Carduus collinus	95	.	.	.	.	.	1	1	1	1	1	.	.	1	.	1	.
Mentha pulegium	94	5	1	.	7	1	1	1	.	.	1	.	.	.	.	.	.
Prunus tenella	94	.	.	.	.	.	.	.	.	1	2	.	.	.	.	.	.
Symphytum tuberosum	94	.	1	.	.	1	1	1	2	1	.	.	.	1	.	1	.
Brassica elongata	92	.	.	.	.	.	.	.	.	1	2	.	.	.	1	.	.
Cirsium acaulon	92	.	.	.	.	1	1	1	4	1	.	.	.	1	.	1	.
Cephalaria radiata	92	.	.	.	.	.	.	.	.	2	1	.	.	3	.	.	6
Rosa spinosissima	92	.	.	.	.	.	1	.	.	2	1	.	.	3	.	.	5
Anacamptis pyramidalis	90	.	.	.	.	1	1	1	3	2	1	.	.	1	1	.	.
Herniaria glabra	90	.	.	5	1	1	1	1	.	1	1	2	7	.	.	.	.
Agropyron pectinatum	90	.	.	.	.	.	.	.	.	.	2	1	.	.	.	.	.
Melilotus albus	89	.	.	1	1	1	1	2	1	1	1	.	1	.	.	.	.
Aurinia saxatilis	89	.	.	.	.	.	.	.	.	.	1	.	.	6	.	1	4
Torilis japonica	89	.	1	.	1	.	1	1	1	1	1	.	.	.	.	1	.
Lotus pedunculatus	89	3	5	.	1	1	1	.	1	.	.	.	.	.	.	.	.
Dianthus membranaceus	88	.	.	.	.	.	.	1	.	1	2	.	.	.	.	.	.
Stachys palustris	87	1	3	.	3	1	1	.	1	.	1	.	.	.	.	.	.
Leucojum aestivum	87	2	3	.	6	.	1	1	.	.	.	.	.	.	.	.	.
Lappula squarrosa	86	.	.	.	.	.	1	.	1	1	2	2	.	1	.	.	.
Saxifraga bulbifera	86	.	.	.	.	1	1	1	1	1	1	.	.	.	.	.	.
Anacamptis palustris	86	6	4	.	4	.	1	.	.	.	1	.	.	.	.	.	.
Viola reichenbachiana	86	.	2	.	.	1	1	.	1	1	.	.	.	1	.	1	.
Malva thuringiaca	85	.	.	.	.	.	1	1	.	1	1	.	.	.	.	.	.
Veronica vindobonensis	84	.	.	.	1	.	1	2	2	1	1	.	.	1	.	.	.
Elymus uralensis	84	.	.	.	.	.	.	.	.	1	1	1	.	.	.	.	.
Sedum rupestre	83	.	.	.	.	.	1	.	1	1	1	.	1	2	1	.	.
Astragalus danicus	83	.	.	.	.	1	1	.	3	1	1	.	.	1	.	.	.
Noccaea caerulescens	83	.	.	.	.	1	1	.	1	.	1	.	.	.	.	.	.
Psephellus marschallianus	83	.	.	.	.	.	.	.	.	1	2	.	.	.	.	.	.
Ranunculus sardous	83	.	1	1	4	.	1	1	1	1	1	.	.	.	.	.	.
Vicia grandiflora	82	.	.	.	1	.	1	1	.	.	1	2	.	.	.	.	.
Crepis praemorsa	82	.	.	.	.	1	1	.	2	1	1	.	.	1	.	1	.
Trifolium pannonicum	82	.	.	.	.	1	1	.	2	1	1	.	.	.	.	.	.
Pilosella lactucella	82	1	1	.	1	2	1	.	1	1	1	.	.	1	.	.	.
Rapistrum perenne	82	.	.	.	.	.	.	.	1	1	1	.	.	.	.	.	.
Onosma arenaria	81	.	.	.	.	.	.	.	.	.	1	8	3	1	1	.	.
Ranunculus illyricus	81	.	.	.	.	.	1	.	.	1	1	.	.	.	1	.	.
Galium ruthenicum	81	.	.	.	.	.	1	.	.	.	1	2	.	.	.	.	.
Inula conyzae	80	.	.	.	.	.	1	1	1	1	1	.	.	1	1	2	.
Allium rotundum	80	.	.	.	.	.	1	1	.	1	1	.	.	.	.	.	.
Crepis conyzifolia	79	.	.	.	.	4	1	.	.	.	.	.	.	.	.	.	.
Pteridium aquilinum	79	1	1	.	.	2	1	1	1	1	1	.	.	1	.	1	.
Gentiana verna	79	1	.	.	.	.	1	.	4	1	.	.	.	.	.	1	.
Juncus compressus	78	.	2	3	5	1	1	.	1	1	1	.	1	.	.	.	.
Orobanche alba	78	.	.	.	.	.	.	.	1	1	1	1	1	1	.	.	.
Gypsophila collina	78	.	.	.	.	.	.	.	.	1	1	.	.	3	1	.	.
Botrychium lunaria	78	.	.	.	.	3	1	.	1	1	1	1	1	1	1	2	.
Silene chlorantha	78	.	.	.	.	.	.	.	.	.	2	.	.	.	.	.	.
Chenopodium album agg.	77	.	1	1	1	1	1	1	.	1	1	4	1	.	.	.	.
Bromus arvensis	77	.	.	.	1	.	1	2	1	1	1	.	1	1	.	.	.
Bassia prostrata	77	.	.	.	.	.	.	1	.	.	1	1	.	.	.	.	.
Anagallis arvensis	76	.	1	1	1	.	1	1	.	1	1	.	.	.	.	.	.
Melampyrum barbatum	76	.	.	.	.	.	1	1	.	2	1	2	.	1	1	.	.
Carex melanostachya	76	1	1	1	6	.	1	1	.	.	1	.	.	.	.	.	.
Equisetum pratense	76	1	1	2	2	1	1	1	1	1	1	.	.	.	.	.	.
Saxifraga granulata	75	.	.	.	1	1	1	1	1	.	1	.	.	.	.	.	.
Trifolium spadiceum	75	.	4	.	.	2	1	.	.	.	.	.	.	.	.	.	.
Senecio nemorensis agg.	75	1	1	.	.	2	1	.	1	1	.	.	.	.	.	4	.
Lactuca viminea	74	.	.	.	.	.	.	.	1	.	1	.	.	1	1	.	.
Ornithogalum pyramidale	74	.	.	.	1	1	1	3	1	1	1	.	.	1	.	.	.
Setaria pumila	74	.	.	.	1	.	1	1	.	.	1	2	6	1	.	.	.
Dianthus superbus	74	5	.	.	.	1	1	.	1	.	.	.	.	.	.	.	.
Bromus sterilis	74	.	.	.	1	.	1	1	1	1	1	1	.	1	.	.	.
Cytisus procumbens	72	.	.	.	.	.	.	.	1	2	1	.	.	1	.	1	.
Thalictrum simplex	72	1	.	2	1	.	1	.	1	1	1	.	.	.	.	.	.
Aconitum anthora	71	.	.	.	.	.	.	.	1	1	1	.	.	4	.	1	7
Caucalis platycarpos	71	.	.	.	.	.	1	1	1	1	1	.	.	.	1	.	.
Consolida regalis	70	.	.	1	.	.	1	1	.	1	1	.	.	.	.	.	.
Geranium pusillum	70	.	.	.	1	.	1	1	1	1	1	.	.	1	.	.	.

Cluster number		1	2	3	4	5	6	7	8	9	10	11	12	13	14	15	16
No. of relevés (all)		257	260	213	797	1411	4434	526	1306	1779	5089	201	203	761	429	219	108
- (with NVP recorded)		133	133	51	234	471	1227	173	409	559	1421	46	121	145	59	100	12
Potentilla humifusa	70	.	.	.	.	.	.	.	.	1	1	.	.	.	.	.	.
Pilosella caespitosa	69	.	.	.	1	1	1	1	1	1	1	.	.	1	.	.	.
Bromus japonicus	69	.	.	.	1	.	1	1	.	1	1	.	.	.	.	.	.
Podospermum canum	69	.	.	1	2	.	1	1	1	.	1	.	.	.	.	.	.
Polygonum amphibium	69	1	1	.	6	.	1	.	.	.	.	.	.	.	.	.	.
Nigella arvensis	68	.	.	.	.	.	.	.	.	.	1	.	.	.	.	.	.
Herniaria polygama	68	.	.	2	.	.	1	.	.	.	1	1	2	.	.	.	.
Potentilla collina agg.	68	.	.	.	.	1	1	2	2	1	1	.	2	.	.	.	.
Genista germanica	68	.	.	.	.	1	1	.	2	1	1	.	.	1	.	.	.
Valerianella dentata	67	.	.	.	.	.	1	1	.	1	1	.	.	1	.	.	.
Carex brizoides	67	1	3	.	1	1	1	.	.	.	.	.	.	.	.	.	.
Sisymbrium polymorphum	67	.	.	.	.	.	.	.	.	1	1	.	.	.	.	.	.
Polygonatum verticillatum	67	.	.	.	.	3	1	.	1	.	.	.	.	.	.	2	.
Iris variegata	67	.	.	.	.	.	.	.	1	1	1	.	.	1	.	.	.
Carex echinata	67	2	6	.	1	1	1	1	.	.	1	.	.	.	.	.	.
Centaurea orientalis	67	.	.	.	.	.	.	.	.	1	1	.	.	.	.	.	.
Juncus tenuis	66	.	1	1	2	1	1	1	.	.	.	.	1	.	.	.	.
Allium podolicum	66	.	.	.	.	.	.	1	.	1	1	1	.	1	.	.	.
Allium paniculatum	66	.	.	.	.	.	.	.	.	1	1	3	.	1	.	.	.
Cyanus mollis	65	.	2	.	.	1	1	.	1	.	.	.	.	1	.	5	.
Carduus crispus	65	.	1	1	1	.	1	1	.	1	1	.	.	.	.	.	.
Filago arvensis	65	.	.	.	.	.	1	1	.	.	1	1	6	.	.	.	.
Polygala chamaebuxus	64	.	.	.	.	.	1	.	3	1	.	.	.	1	1	.	.
Myosotis laxa	64	.	5	3	2	1	1	.	.	.	.	.	.	.	.	.	.
Linum perenne	64	.	.	1	.	.	1	1	1	1	1	.	.	1	.	1	.
Vaccinium vitis-idaea	64	.	.	.	.	4	1	.	1	1	.	.	.	.	.	3	.
Lamium purpureum	63	.	.	.	1	.	1	1	1	1	1	.	.	.	.	.	.
Peucedanum ruthenicum	63	.	.	.	.	.	.	.	.	1	1	.	.	.	.	.	.
Viola riviniana	62	1	.	.	.	1	1	1	1	1	.	.	.	1	.	1	.
Gaudinia fragilis	62	.	3	.	1	.	1	.	1	.	.	.	.	.	.	.	.
Lathyrus niger	62	.	.	.	.	1	1	1	3	1	1	.	.	.	.	.	.
Gentianella ciliata	61	.	.	.	.	1	1	1	2	1	.	.	.	1	.	1	1
Euphorbia stricta	61	1	1	1	1	1	1	1	1	1	1	.	.	.	.	1	1
Equisetum telmateia	61	1	4	.	1	1	1	.	1	1	.	.	.	.	.	.	.
Knautia dipsacifolia	61	.	.	.	.	1	1	1	1	1	1	.	.	.	.	2	.
Rumex alpestris	61	.	1	.	.	2	1	1	1	.	.	.	.	.	.	.	.
Lathyrus nissolia	60	.	.	.	1	.	1	2	1	1	1	.	.	.	.	.	.
Nepeta nuda	60	.	.	.	.	.	1	1	1	2	1	.	.	1	.	.	.
Convolvulus lineatus	60	.	.	.	.	.	.	.	.	.	1	.	.	.	.	.	.
Viola pumila	59	1	.	.	5	.	1	1	1	.	1	.	.	.	.	.	.
Pilosella aurantiaca	59	.	1	.	1	2	1	.	.	.	1	.	.	1	.	.	.
Centaurea pectinata	59	.	.	.	1	.	1	.	1	.	.	.	.	.	.	.	.
Tanacetum millefolium	58	.	.	.	.	.	.	.	.	1	1	.	.	.	.	.	.
Equisetum ramosissimum	58	1	.	.	1	.	1	.	1	.	1	6	7	.	.	.	.
Galeopsis tetrahit	58	.	.	.	1	1	1	.	1	.	1	.	.	.	.	.	2
Crepis setosa	57	.	.	.	1	1	1	1	.	1	1	.	.	.	.	.	.
Polygonum viviparum	57	.	.	.	.	1	1	.	1	.	.	.	.	.	.	1	.
Apera spica-venti	57	.	.	.	.	.	1	.	1	1	1	4	7	.	.	.	.
Anacamptis coriophora	56	1	.	3	1	.	1	1	1	1	1	.	1	1	.	.	.
Descurainia sophia	56	.	1	1	.	.	1	.	.	1	1	1	1	.	.	.	.
Silene viscosa	56	.	.	.	.	.	1	1	.	1	1	.	2	1	.	.	.
Echinops sphaerocephalus	55	.	.	.	1	.	1	.	1	1	1	1	.	1	.	.	.
Festuca stricta ssp. trachyphylla	55	.	.	.	.	.	1	.	1	1	1	.	1	.	.	.	.
Spiraea media	55	.	.	.	.	.	.	.	.	1	1	.	.	4	.	.	6
Astragalus vesicarius	55	.	.	.	.	.	.	.	.	1	1	.	.	1	2	.	.
Poa pannonica	55	.	.	.	.	.	.	.	.	.	1	.	1	.	.	.	.
Hypochaeris uniflora	54	.	.	.	.	3	1	.	1	.	.	.	.	.	.	.	.
Campanula rapunculus	54	.	.	.	1	.	1	1	1	1	1	.	.	1	1	.	.
Orobanche caryophyllacea	54	.	.	.	.	.	1	.	1	1	1	.	.	1	1	1	1
Filago germanica	53	.	1	1	1	1	1	.	1	1	1	.	2	1	.	.	.
Galium humifusum	53	.	.	.	.	.	1	1	.	.	1	.	.	.	.	.	.
Hyacinthella leucophaea	53	.	.	.	.	.	.	.	.	1	1	.	.	.	.	.	.
Silene nemoralis	53	.	.	.	.	1	1	1	1	1	1	.	.	1	.	2	.
Lilium bulbiferum	53	.	.	.	.	1	1	.	1	1	.	.	.	.	.	.	.
Ranunculus ficaria	53	.	1	.	2	.	1	1	1	1	1	.	.	.	.	.	.
Inula germanica	53	.	.	.	.	.	1	1	1	1	1	.	.	.	.	.	.
Cerastium glomeratum	52	.	.	.	1	1	1	1	1	.	1	1	1	.	.	.	.
Sagina procumbens	52	.	1	1	1	1	1	.	.	.	1	.	1	.	.	.	.
Echium italicum	51	.	.	.	.	.	1	1	.	1	1	.	.	.	.	.	.
Oxalis stricta	51	.	.	.	1	1	1	.	.	1	1	.	.	.	.	.	.
Orchis mascula	51	.	.	.	.	1	1	.	1	1	.	.	.	.	.	.	.
Dactylorhiza fuchsii	51	.	1	.	.	2	1	.	1	.	.	.	.	.	.	.	.
Ononis pusilla	50	.	.	.	.	.	.	.	.	1	1	.	.	.	4	.	.
Papaver dubium	50	.	.	.	.	.	.	.	.	1	1	1	.	1	.	.	.
Gentianella germanica	50	.	.	.	.	1	1	.	2	1	.	.	.	.	.	1	.
Trifolium striatum	50	.	.	.	1	.	1	2	.	.	1	.	.	.	.	.	.
Carthamus lanatus	50	.	.	.	.	.	1	.	.	1	1	.	.	.	.	.	.
Senecio aquaticus	49	5	3	.	1	.	1	.	1	.	.	.	.	.	.	.	.
Stellaria holostea	49	.	.	1	1	1	1	.	1	1	1	.	.	.	.	1	.
Galium odoratum	49	.	.	.	1	1	1	.	1	.	1	.	.	.	.	1	.
Sesleria sadlerana	49	.	.	.	.	.	.	.	.	1	1	.	.	5	2	1	.

Cluster number		1	2	3	4	5	6	7	8	9	10	11	12	13	14	15	16
No. of relevés (all)		257	260	213	797	1411	4434	526	1306	1779	5089	201	203	761	429	219	108
- (with NVP recorded)		133	133	51	234	471	1227	173	409	559	1421	46	121	145	59	100	12
Ferulago sylvatica	49	.	.	.	.	.	1	.	1	2	1	.	.	1	.	.	.
Juncus gerardii	49	1	1	2	3	.	1	.	.	.	1	.	.	.	.	.	.
Ballota nigra	49	.	1	.	.	.	1	1	.	.	1	.	.	1	.	.	.
Herniaria incana	48	.	.	.	.	.	.	.	.	.	1	.	.	.	.	.	.
Daphne cneorum	47	.	.	.	.	.	.	.	1	1	1	.	.	4	1	1	1
Hesperis tristis	47	.	.	.	.	.	.	.	.	1	1	.	.	.	.	.	.
Veratrum nigrum	46	.	1	.	.	.	1	1	1	1	1	.	.	1	1	.	.
Stellaria palustris	46	.	2	.	5	.	1	.	1	1	.	.	.	.	.	.	.
Dipsacus laciniatus	46	.	.	.	1	.	1	2	.	1	1	.	.	.	.	.	.
Anthemis arvensis	46	.	.	.	1	1	1	1	1	.	1	.	1	.	.	.	.
Ephedra distachya	46	.	.	.	.	.	.	.	.	.	1	4	.	.	.	.	.
Dactylorhiza sambucina	45	.	.	.	.	1	1	1	1	.	.	.	.	.	.	.	.
Sedum hispanicum	45	.	.	.	.	.	.	.	.	.	1	.	.	3	.	.	3
Carex elata	45	7	3	.	2	.	1	.	.	.	.	.	.	.	.	.	.
Hierochloe odorata	45	.	.	4	1	.	1	.	.	.	1	1	1	.	.	.	.
Galium schultesii	45	.	.	.	.	1	1	.	1	1	1	.	.	1	.	3	3
Maianthemum bifolium	45	.	1	.	.	2	1	.	1	1	.	.	.	.	.	1	.
Achillea ptarmica	45	2	4	.	1	.	1	.	.	.	1	.	.	.	.	.	.
Linaria angustissima	45	.	.	.	.	.	.	.	.	1	1	.	.	1	1	.	.
Myosotis discolor	44	.	.	.	1	.	1	1	1	.	1	.	.	.	.	.	.
Scrophularia nodosa	44	.	1	.	1	1	1	1	1	1	1	.	.	.	.	.	.
Thalictrum aquilegiifolium	44	1	.	.	1	1	1	.	1	1	1	.	.	1	.	2	.
Epilobium montanum	44	.	1	.	1	1	1	1	.	.	1	.	.	.	.	5	2
Pilosella hoppeana	44	.	.	.	.	.	1	1	1	1	1	.	.	1	1	.	1
Carex umbrosa	43	2	.	.	.	1	1	.	.	.	.	.	.	.	.	.	.
Veronica scutellata	43	1	4	1	3	.	1	.	.	.	.	.	.	.	.	.	.
Orobanche lutea	43	.	.	.	.	.	1	1	1	1	1	.	.	.	.	.	.
Dactylorhiza incarnata	43	3	3	1	1	.	1	.	.	1	.	.	.	.	.	.	.
Lycopodium clavatum	43	.	.	.	.	3	1	.	.	.	.	.	.	.	.	.	.
Astragalus asper	43	.	.	.	.	.	1	.	.	1	1	.	.	.	.	.	.
Carex hartmanii	43	2	3	1	1	1	1	.	.	.	.	.	.	.	.	.	.
Sambucus ebulus	42	.	.	.	1	.	1	1	.	1	1	.	.	.	.	.	.
Ambrosia artemisiifolia	42	.	.	.	1	.	1	1	.	.	1	.	1	.	.	.	.
Solidago canadensis	42	.	1	.	1	.	1	2	1	1	1	.	.	.	.	.	.
Carex colchica	42	1	.	1	.	.	1	.	.	.	1	5	1	.	.	.	.
Aristolochia clematitis	42	.	.	2	1	.	1	1	1	.	1	.	.	.	.	.	1
Pulmonaria angustifolia	42	.	.	.	.	.	1	1	2	1	1	.	.	.	.	.	.
Euphorbia angulata	41	.	.	.	.	.	1	.	1	1	1	.	.	1	.	.	.
Potentilla astracanica	41	.	.	.	.	.	.	.	.	.	1	.	.	.	.	.	.
Pulmonaria officinalis agg.	41	.	1	.	.	1	1	1	1	1	1	.	.	.	.	1	.
Arenaria procera	40	.	.	.	.	.	.	.	.	1	1	1	.	.	.	.	.
Melampyrum pratense	40	.	1	.	1	1	1	1	1	1	1	.	.	1	.	1	1
Stachys sylvatica	40	.	2	.	1	1	1	1	1	.	1	.	.	.	.	.	.
Iris humilis	40	.	.	.	1	.	.	.	.	1	1	2	1	.	3	.	.
Thalictrum foetidum	40	.	.	.	.	.	.	.	.	1	1	.	.	3	.	.	6
Lamium amplexicaule	39	.	.	.	.	.	1	.	.	.	1	1	1	.	1	.	.
Viola elatior	39	4	.	.	2	.	1	1	1	.	.	.	.	.	.	.	.
Stipa dasyphylla	39	.	.	.	.	.	.	.	.	1	1	.	.	.	1	.	.
Seseli longifolium	39	.	.	.	.	.	.	.	.	1	1	.	.	2	.	4	.
Marrubium pestalozzae	38	.	.	.	.	.	.	.	.	.	1	.	.	.	.	.	.
Scutellaria galericulata	38	2	3	1	2	.	1	.	.	.	1	.	.	.	.	.	.
Limonium gmelinii	38	.	.	.	1	.	1	.	.	.	1	.	.	.	.	.	.
Geranium phaeum	38	.	1	.	1	1	1	.	.	.	1	.	.	.	.	1	.
Salvia aethiopis	38	.	.	.	.	.	.	1	.	.	1	.	.	.	.	.	.
Geranium palustre	38	1	3	.	1	.	1	.	1	.	.	.	.	.	.	.	.
Poa versicolor	38	.	.	.	.	.	.	.	.	1	1	.	.	.	.	.	.
Nepeta ucranica	38	.	.	.	.	.	.	.	.	.	1	.	.	.	.	.	.
Lamium maculatum	37	.	.	.	1	.	1	1	.	1	1	.	.	1	.	1	1
Viola suavis	37	.	.	.	.	1	1	.	1	1	1	.	1	.	.	.	.
Xeranthemum inapertum	37	.	.	.	.	.	1	2	.	.	1	.	.	.	.	.	.
Barbarea vulgaris	37	1	.	.	1	.	1	.	1	.	1	.	.	1	.	.	.
Epilobium parviflorum	37	2	5	.	1	.	1	1	.	.	.	.	.	.	.	.	.
Hieracium laevicaule	37	.	.	.	.	3	1	.	.	.	.	.	.	.	.	.	.
Carex divisa	37	.	.	.	1	.	1	1	.	.	1	.	.	.	.	.	.
Hieracium laevigatum	37	.	1	.	1	1	1	1	1	1	1	.	.	1	.	1	1
Noccaea praecox	36	.	.	.	.	.	.	.	2	1	.	.	.	.	.	.	.
Geranium dissectum	36	.	.	.	1	.	1	1	.	.	1	.	.	.	.	.	.
Campanula abietina	36	.	.	.	.	1	1	.	.	.	.	.	.	.	.	.	2
Linum linearifolium	36	.	.	.	.	.	.	.	.	.	1	.	.	.	.	.	.
Carduus candicans	36	.	.	.	.	.	.	.	.	.	1	.	.	2	.	.	1
Trifolium pallidum	36	.	.	.	1	.	1	1	.	.	.	.	.	.	.	.	.
Althaea officinalis	36	.	.	1	2	.	1	1	.	.	1	.	.	.	.	.	.
Torilis arvensis	36	.	.	.	1	.	1	1	.	1	1	.	.	.	.	.	.
Plantago maritima	36	2	.	1	1	.	1	.	1	.	1	.	.	.	.	.	.
Euphorbia palustris	36	1	1	1	3	.	1	1	1	.	1	.	.	.	.	.	.
Pimpinella tragium	36	.	.	.	.	.	.	.	.	.	1	.	.	.	.	.	.
Alyssum murale	36	.	.	.	.	.	.	.	.	.	1	1	.	1	.	.	1
Polycnemum arvense	36	.	.	.	.	.	.	.	.	.	1	1	1	.	.	.	.
Euphorbia amygdaloides	35	.	1	.	.	1	1	.	1	1	1	.	.	1	.	5	1
Carex ericetorum	35	.	1	.	.	.	1	.	1	1	1	.	5	.	1	3	.
Lithospermum officinale	35	.	.	1	.	1	.	1	1	1	1	.	.	.	.	.	.

Cluster number		1	2	3	4	5	6	7	8	9	10	11	12	13	14	15	16
No. of relevés (all)		257	260	213	797	1411	4434	526	1306	1779	5089	201	203	761	429	219	108
- (with NVP recorded)		133	133	51	234	471	1227	173	409	559	1421	46	121	145	59	100	12
Carex riparia	35	1	1	.	4	.	1	.	.	.	1	.	.	.	.	.	.
Campanula cervicaria	35	.	.	.	.	1	1	.	1	1	1	.	.	.	.	.	.
Alyssum rostratum	35	.	.	.	.	.	.	.	.	.	1	.	.	.	.	.	.
Hypericum tetrapterum	35	1	4	.	1	.	1	1	1	1	.	.	.	.	.	.	.
Asparagus tenuifolius	35	.	.	.	1	.	.	.	.	.	1	1	.	.	.	.	.
Hypericum hirsutum	35	.	1	.	.	.	1	1	1	1	.	.	.	.	.	1	.
Teucrium botrys	34	.	.	.	.	.	.	.	.	1	1	.	.	1	1	1	.
Ranunculus zapalowiczii	34	.	.	.	.	.	.	.	.	2	1	.	.	.	.	.	.
Festuca dalmatica	34	.	.	.	.	.	.	.	.	.	1	.	.	1	.	.	1
Arctium lappa	34	.	.	.	2	.	1	.	.	.	1	.	.	.	.	.	.
Symphyotrichum novi-belgii agg.	34	.	.	.	4	.	1	.	.	.	.	.	.	.	.	.	.
Minuartia hirsuta	34	.	.	.	.	.	.	.	.	.	1	.	.	1	.	.	.
Erica carnea	34	1	.	.	.	.	1	.	1	1	.	.	.	1	.	1	.
Astragalus exscapus	33	.	.	.	.	.	.	.	.	1	1	.	.	.	.	.	.
Jurinea stoechadifolia	33	.	.	.	.	.	.	.	.	.	1	.	.	.	.	.	.
Polygonum persicaria	33	1	1	1	3	.	1	.	.	.	.	.	.	.	.	.	.
Echinops ritro	33	.	.	.	.	.	.	.	.	1	1	.	.	2	1	.	.
Alyssum desertorum	33	.	.	.	.	.	.	.	.	.	1	4	1	.	.	.	.
Veronica hederifolia agg.	33	.	.	.	1	.	1	1	1	1	1	.	1	.	.	.	.
Dianthus barbatus	32	.	1	.	.	1	1	.	1	.	.	.	.	.	.	.	.
Cuscuta europaea	32	.	.	.	.	1	1	1	1	1	1	.	.	.	.	.	.
Asperula montana	32	.	.	.	.	.	.	.	.	.	1	.	.	.	.	.	.
Peucedanum palustre	32	3	1	.	1	.	1	.	.	.	.	.	.	.	.	.	.
Euphorbia villosa	32	2	.	.	.	.	1	.	1	1	1	.	.	.	.	.	.
Allium waldsteinii	32	.	.	.	1	.	.	.	.	.	1	.	.	.	.	.	.
Coronilla coronata	32	.	.	.	.	.	1	.	.	1	1	.	.	2	.	3	.
Senecio doria	32	.	.	.	1	.	1	1	1	1	1	.	.	.	.	.	.
Euphorbia lucida	32	.	.	.	4	.	.	1	.	.	.	.	.	.	.	.	.
Psephellus trinervius	32	.	.	.	.	.	.	.	.	.	1	.	.	.	.	.	.
Rhinanthus borbasii	32	.	.	.	.	.	.	.	.	.	1	.	.	.	.	.	.
Senecio subalpinus	31	.	1	.	.	1	1	.	.	.	.	.	.	.	.	1	.
Mercurialis ovata	31	.	.	.	.	.	.	1	1	1	.	.	.	1	.	.	.
Helleborus purpurascens	31	.	.	.	.	1	.	.	1	1	1	.	.	.	.	.	.
Lathyrus vernus	31	.	.	.	.	1	1	.	1	.	1	.	.	.	.	5	2
Laserpitium siler	31	.	.	.	.	.	.	.	1	1	.	.	.	1	.	.	.
Gagea bohemica	31	.	.	.	.	.	.	.	.	.	1	1	1	1	1	.	.
Teucrium scordium	31	2	.	.	3	.	.	.	.	.	1	.	.	.	.	.	.
Thlaspi arvense	31	.	.	1	.	1	1	.	1	1	1	.	.	1	.	.	.
Epilobium tetragonum	31	1	2	.	1	.	1	1	.	1	1	.	.	.	.	.	.
Thymelaea passerina	31	.	.	.	.	.	.	.	.	1	1	.	.	.	1	.	.
Psephellus sumensis	31	.	.	.	.	.	.	.	.	.	1	1	.	.	.	.	.
Silene italica	31	.	.	.	.	.	.	1	1	1	1	.	.	2	.	.	.
Papaver rhoeas	31	.	.	.	.	.	1	1	.	1	1	1	.	.	.	.	.
Eleocharis uniglumis	30	1	1	3	3	.	.	.	.	.	.	.	.	.	.	.	.
Euphorbia helioscopia	30	1	1	.	1	.	1	1	.	1	1	.	.	.	1	.	.
Ophrys apifera	30	.	1	.	.	.	1	.	1	1	.	.	.	1	.	.	.
Oxalis acetosella	30	.	1	.	1	1	1	.	1	.	1	.	.	.	.	3	.
Knautia illyrica	30	.	.	.	.	.	1	1	2	1	.	.	.	.	.	.	.
Arctium tomentosum	30	.	.	1	1	1	1	1	.	.	1	.	.	.	.	.	.
Ranunculus lanuginosus	29	.	1	.	.	1	1	.	1	.	1	.	.	.	.	1	.
Iris graminea	29	.	.	.	.	1	1	1	1	1	1	.	.	.	.	.	.
Alopecurus geniculatus	29	.	3	.	2	.	1	.	.	.	.	.	.	.	.	.	.
Festuca beckeri	29	.	.	1	.	.	1	.	.	.	1	3	1	.	.	.	.
Euphorbia platyphyllos	29	.	.	.	1	.	1	1	1	1	1	.	.	.	.	.	.
Vicia pannonica	29	.	.	.	1	.	1	1	.	.	1	.	.	.	.	.	.
Corydalis solida	29	.	.	.	.	.	1	.	.	.	1	.	.	1	.	.	.
Spiraea hypericifolia	28	.	.	.	.	.	.	.	.	.	1	.	.	.	.	.	.
Silene dioica	28	.	1	.	.	1	1	1	.	1	.	.	.	.	.	1	.
Gypsophila thyraica	28	.	.	.	.	.	.	.	.	1	1	.	.	1	.	.	.
Triglochin palustris	28	2	4	.	2	.	.	.	.	.	.	.	.	.	.	.	.
Erysimum hieraciifolium agg.	28	.	.	1	.	.	1	.	.	1	1	.	.	.	1	.	.
Polygonum hydropiper	28	.	1	.	2	.	1	.	.	.	1	.	1	.	.	.	.
Hypericum montanum	28	1	.	.	.	1	1	.	1	1	1	.	.	1	.	.	.
Pilosella pavichii	28	.	.	.	.	.	.	.	.	.	1	.	.	2	.	.	5
Carex vesicaria	28	.	4	.	2	.	1	.	.	.	.	.	.	.	.	.	.
Sisymbrium orientale	27	.	.	.	.	.	.	.	.	.	1	4	1	.	.	.	.
Phleum rhaeticum	27	.	.	.	.	1	1	.	.	.	.	.	.	.	.	.	1
Galeopsis speciosa	27	.	1	.	1	1	1	.	.	.	.	.	.	.	.	.	.
Cerastium dubium	27	.	.	.	3	.	1	.	.	.	.	.	.	.	.	.	.
Scabiosa cinerea ssp. hladnikiana	27	.	.	.	.	.	.	.	1	1	.	.	.	.	.	.	.
Dipsacus fullonum	27	.	.	.	1	.	1	1	1	1	1	.	.	.	.	.	.
Bidens tripartitus	27	1	1	.	3	.	.	1	.	.	.	.	.	.	.	.	.
Ophrys insectifera	27	.	.	.	.	.	.	.	1	1	.	.	.	2	.	.	.
Arabis turrita	27	.	.	.	.	.	.	.	.	1	1	.	.	2	.	.	6
Peucedanum officinale	26	.	.	.	1	.	1	1	1	1	1	.	.	.	.	.	.
Helleborus odorus	26	.	.	.	.	.	1	1	1	1	1	.	.	.	.	.	.
Festuca heterophylla	26	1	.	.	.	1	1	.	1	1	1	.	.	1	.	.	.
Carex cespitosa	26	.	2	2	1	.	1	1	.	.	.	.	.	.	.	.	.
Senecio vernalis	26	.	.	.	.	.	.	.	.	.	1	4	1	.	.	.	.
Dichoropetalum carvifolia	26	1	.	.	.	.	1	.	1	1	1	.	.	1	.	.	.
Pedicularis kaufmannii	26	.	.	.	1	.	.	.	.	1	1	.	.	.	.	.	.

Cluster number		1	2	3	4	5	6	7	8	9	10	11	12	13	14	15	16
No. of relevés (all)		257	260	213	797	1411	4434	526	1306	1779	5089	201	203	761	429	219	108
- (with NVP recorded)		133	133	51	234	471	1227	173	409	559	1421	46	121	145	59	100	12
Verbascum marschallianum	26	.	.	.	.	.	.	.	.	.	1	.	.	.	.	.	.
Hypochaeris glabra	25	.	.	.	.	1	1	1	.	1	1	.	.	1	.	1	.
Sagina nodosa	25	1	1	2	1	1	1	.	.	.	1	.	.	.	.	.	.
Coeloglossum viride	25	.	.	.	.	1	1	.	1	1	1	.	.	.	.	1	.
Chelidonium majus	25	.	.	.	1	.	1	.	.	.	1	1	.	1	.	1	5
Tragopogon tommasinii	25	.	.	.	.	.	1	1	1	1	.	.	.	.	.	.	.
Sonchus asper	25	.	.	.	1	.	1	1	.	1	1	.	.	.	.	.	1
Dianthus monspessulanus	25	.	.	.	.	.	1	.	1	1	.	.	.	1	.	.	.
Juncus bufonius agg.	25	.	.	2	1	.	1	.	.	.	.	.	.	.	.	.	.
Linum viscosum	25	.	.	.	.	.	.	.	1	1	1	.	.	.	.	.	.
Campanula scheuchzeri	25	.	.	.	.	1	1	.	1	.	.	.	.	.	.	.	.
Juncus subnodulosus	25	4	.	.	.	.	1	.	1	.	.	.	.	.	.	.	.
Alliaria petiolata	25	.	1	.	.	.	1	.	.	1	1	.	.	1	.	.	2
Saponaria officinalis	25	.	.	1	1	.	1	1	.	1	1	.	.	.	.	1	.
Lathyrus palustris	25	3	1	.	2	.	1	.	.	.	.	.	.	.	.	.	.
Lapsana communis	24	.	.	.	1	1	1	1	.	1	1	.	.	.	.	.	3
Saponaria bellidifolia	24	.	.	.	.	.	.	.	.	.	1	.	.	2	.	.	1
Cephalaria transsylvanica	24	.	.	.	.	.	.	.	.	.	1	.	.	.	.	.	.
Goniolimon tataricum	24	.	.	.	.	.	.	.	.	.	1	.	.	.	.	.	.
Plantago holosteum	24	.	.	.	.	.	.	.	2	1	.	.	.	.	.	.	.
Ranunculus pedatus	24	.	.	.	1	.	1	1	.	.	1	.	.	.	.	.	.
Sinapis arvensis	24	.	.	.	1	.	1	1	1	.	1	.	.	.	.	.	.
Orobanche teucrii	24	.	.	.	.	.	.	.	.	1	1	.	.	.	.	.	.
Galeopsis ladanum	24	.	.	.	.	1	1	2	.	.	1	.	.	1	.	1	.
Viola mirabilis	24	.	.	.	.	.	1	1	1	1	1	.	.	1	.	1	.
Epilobium angustifolium	24	.	1	.	.	1	1	1	1	1	1	.	.	1	.	.	.
Gladiolus palustris	24	2	.	.	.	.	1	.	1	.	.	.	.	.	.	.	.
Artemisia scoparia	24	.	.	.	.	.	.	.	.	.	1	.	.	.	.	.	.
Allium paczoskianum	24	.	.	.	.	.	.	.	.	.	1	1	.	.	.	.	.
Lysimachia nemorum	23	1	2	.	.	1	1	.	.	.	.	.	.	.	.	.	.
Stellaria nemorum	23	.	.	.	.	1	1	.	.	.	.	.	.	.	.	.	.
Petasites hybridus	23	.	2	.	1	.	1	.	.	.	.	.	.	.	.	1	.
Rumex alpinus	23	.	.	.	.	1	1	.	.	.	.	.	.	.	.	.	.
Xanthium strumarium	23	.	.	.	1	.	1	.	.	.	1	1	.	.	.	.	.
Fallopia dumetorum	23	.	.	.	.	.	.	.	.	1	1	.	.	1	.	.	.
Galeopsis pubescens	23	.	1	.	.	1	1	.	.	1	1	.	.	1	.	1	.
Cynoglottis barrelieri	23	.	.	.	.	.	.	1	1	1	1	.	.	1	.	.	.
Polygonum lapathifolium	23	.	.	.	1	1	1	.	.	.	1	.	.	.	.	.	.
Onopordum acanthium	23	.	.	.	.	.	1	.	.	1	1	.	1	.	.	.	.
Veronica persica	23	.	.	.	.	.	1	1	.	.	1	.	.	.	.	.	.
Verbascum blattaria	23	.	.	.	1	.	1	1	1	.	1	.	.	.	1	.	.
Galium rivale	23	1	3	.	1	.	1	.	.	.	1	.	.	.	.	.	.
Silene tatarica	23	.	.	.	.	.	1	.	.	.	1	1	.	.	.	.	.
Dianthus pseudarmeria	23	.	.	.	.	.	.	.	.	.	1	.	.	.	.	.	.
Carlina acanthifolia	23	.	.	.	.	.	.	.	.	1	1	.	.	1	.	.	.
Astragalus australis	23	.	.	.	.	.	.	.	.	.	1	.	.	.	.	.	.
Linum nervosum	22	.	.	.	.	.	.	.	1	1	1	.	.	.	.	.	.
Leontodon saxatilis	22	3	1	.	1	.	1	1	.	.	.	.	.	.	.	.	.
Tragopogon ucrainicus	22	.	.	1	.	.	1	.	.	.	1	1	.	.	.	.	.
Melittis melissophyllum	22	.	.	.	.	.	1	.	1	1	.	.	.	1	.	2	.
Elymus caninus	22	.	1	.	1	.	1	.	1	1	1	.	.	.	.	1	.
Glyceria fluitans	22	.	3	.	2	.	1	.	.	.	.	.	.	.	.	.	.
Echium russicum	22	.	.	.	.	.	.	.	.	1	1	.	.	.	.	.	.
Lactuca saligna	22	.	.	.	1	.	1	2	.	.	1	.	.	.	.	.	.
Crupina vulgaris	22	.	.	.	.	.	.	.	.	1	1	.	.	.	1	.	.
Myosotis sparsiflora	22	.	.	.	.	.	1	.	.	.	1	.	.	.	.	.	.
Cyanus pinnatifidus	22	.	.	.	.	.	.	.	.	1	.	.	.	2	.	.	5
Cirsium furiens	22	.	.	.	.	.	1	1	.	1	1	.	.	1	.	.	.
Juncus acutiflorus	22	2	3	.	.	.	1	.	.	.	.	.	.	.	.	.	.
Aquilegia nigricans	21	.	.	.	.	.	.	.	1	1	.	.	.	1	.	.	1
Astragalus ucrainicus	21	.	.	.	.	.	.	.	.	.	1	.	.	.	.	.	.
Senecio vulgaris	21	.	.	.	.	1	1	1	1	.	1	.	.	1	.	.	.
Ornithogalum collinum	21	.	.	.	.	.	.	1	.	1	1	.	.	.	.	.	.
Klasea lycopifolia	21	.	.	.	1	.	1	.	1	1	1	.	.	.	.	.	.
Allium flavescens	21	.	.	.	.	.	.	.	.	.	1	.	.	1	.	.	.
Veronica triphyllos	21	.	.	.	.	.	.	.	.	1	1	.	.	1	.	.	.
Rumex conglomeratus	21	1	1	.	1	1	1	.	.	.	.	.	.	.	.	.	.
Milium effusum	21	.	1	.	1	1	1	.	.	.	1	.	.	.	.	.	.
Viola persicifolia	21	2	.	.	2	.	1	.	.	.	.	.	.	.	.	.	.
Blysmus compressus	21	2	2	.	1	.	1	.	.	.	.	.	.	.	.	.	.
Sonchus oleraceus	21	.	.	.	1	.	1	1	.	.	1	.	.	.	.	.	.
Galeopsis angustifolia	21	.	.	.	.	.	1	1	.	1	1	.	.	1	.	.	.
Anemone ranunculoides	21	.	.	.	1	1	1	1	1	.	.	.	.	.	.	1	.
Cephalanthera rubra	21	.	.	.	1	.	1	1	.	.	1	.	.	1	.	1	.
Lamium album	21	.	.	.	1	.	1	.	.	.	1	.	.	.	.	.	.
Rumex patientia	21	.	.	.	1	.	1	.	.	.	1	.	.	.	.	.	.
Sempervivum montanum	20	.	.	.	.	.	.	.	.	1	1	.	.	1	.	.	.
Senecio umbrosus	20	.	.	.	.	.	1	1	1	.	.	.	.	1	.	1	.
Pilosella auriculoides	20	.	1	.	.	1	1	.	1	1	1	.	.	1	.	1	.
Thesium procumbens	20	.	.	.	.	.	.	.	.	.	1	.	.	.	.	.	.
Sium latifolium	20	.	.	.	2	.	1	.	.	.	.	.	.	.	.	.	.

Cluster number		1	2	3	4	5	6	7	8	9	10	11	12	13	14	15	16
No. of relevés (all)		257	260	213	797	1411	4434	526	1306	1779	5089	201	203	761	429	219	108
- (with NVP recorded)		133	133	51	234	471	1227	173	409	559	1421	46	121	145	59	100	12
Centaurium pulchellum	20	.	1	.	1	.	1	1	1	1	1	.	.	.	.	.	.
Senecio squalidus	20	.	.	.	.	.	.	.	.	1	1	.	.	1	1	.	3
Bromus commutatus	20	.	.	.	1	.	1	1	1	1	1	.	.	.	.	.	.
Astragalus sulcatus	20	.	.	.	.	.	.	.	.	.	1	.	.	.	.	.	.
Arnica montana	20	.	.	.	.	1	1	.	1	.	.	.	.	.	.	.	.
Amygdalus nana	20	.	.	.	.	.	.	.	.	.	1	.	.	.	.	.	.
Salvia dumetorum	20	.	.	.	.	.	.	.	.	.	1	.	.	.	.	.	.
Cirsium decussatum	20	.	.	.	.	.	1	1	.	.	.	.	.	.	.	.	.
Cardamine hirsuta	20	.	1	.	.	.	1	1	.	.	.	.	.	.	.	.	.
Alisma plantago-aquatica	20	1	1	.	2	.	1	.	.	.	.	.	.	.	.	.	.
Salvia glutinosa	19	.	.	.	.	.	1	1	.	1	1	.	.	.	.	2	.
Muscari botryoides	19	.	.	.	.	.	.	.	.	1	1	.	1	.	.	.	.
Epilobium dodonaei	19	.	.	.	.	.	.	1	.	1	1	.	.	1	.	.	.
Genista scythica	19	.	.	.	.	.	.	.	.	.	1	.	.	.	.	.	.
Sesleria tenuifolia ssp. kalnikensis	19	.	.	.	.	.	.	.	.	1	1	.	.	2	.	1	.
Noccaea jankae	19	.	.	.	.	.	.	.	1	1	1	.	.	.	.	.	.
Arabidopsis petraea	19	.	.	.	.	.	.	.	.	.	.	.	.	1	2	.	.
Erucastrum nasturtiifolium	19	.	.	1	.	.	.	.	.	1	1	.	.	.	1	.	.
Ventenata dubia	19	.	.	.	1	.	1	.	.	.	1	.	.	.	.	.	.
Galeopsis bifida	19	.	.	.	.	1	1	.	.	.	.	.	.	.	.	.	.
Geranium pyrenaicum	19	.	.	.	1	.	1	1	.	.	1	.	.	.	.	.	.
Helictochloa adsurgens	19	.	.	.	.	1	1	.	1	1	.	.	.	.	.	.	.
Androsace elongata	19	.	.	.	.	.	.	.	.	.	1	.	.	.	.	.	.
Inula helenium	19	.	.	.	1	.	1	1	.	1	1	.	.	.	.	.	.
Aegonychon purpurocaeruleum	19	.	.	.	.	.	.	.	.	1	1	.	.	1	.	.	.
Valerianella carinata	19	.	.	.	.	.	1	.	.	1	1	.	.	.	.	.	.
Allium schoenoprasum	18	2	.	.	.	.	1	.	1	.	1	.	.	1	.	.	1
Carex hallerana	18	.	.	.	.	.	1	.	1	1	1	.	.	.	.	.	.
Erysimum comatum	18	.	.	.	.	.	.	.	.	.	1	.	.	1	.	.	4
Crepis capillaris	18	.	.	.	.	1	1	1	.	.	1	1	1	.	.	.	.
Trinia kitaibelii	18	.	.	.	.	.	.	1	.	.	1	.	.	.	.	.	.
Mentha x verticillata	18	1	2	.	1	1	1	.	.	.	.	.	.	.	.	.	.
Soldanella montana agg.	18	.	.	.	.	1	.	.	1	.	.	.	.	.	.	1	.
Xanthium orientale	18	.	.	1	1	.	1	1	.	1	1	1	.	.	.	.	.
Orobanche elatior	18	.	.	.	.	.	.	1	.	1	1	.	.	.	.	.	.
Matricaria chamomilla	18	.	.	1	1	1	1	.	.	.	.	.	.	.	.	.	1
Epilobium hirsutum	18	1	3	.	1	.	1	1	.	.	.	.	.	.	.	.	.
Allium albidum ssp. albidum	18	.	.	.	.	.	.	.	.	.	1	.	.	.	.	.	.
Astragalus corniculatus	18	.	.	.	.	.	.	.	.	.	1	.	.	.	.	.	.
Soldanella carpatica	18	.	.	.	.	1	1	.	.	.	.	.	.	.	.	4	.
Lathyrus hirsutus	18	.	.	.	.	.	1	1	.	1	1	.	.	.	.	.	.
Sempervivum ruthenicum	18	.	.	.	.	.	.	.	.	.	1	1	.	1	.	.	.
Polygonatum multiflorum	18	.	.	.	.	1	1	.	1	.	1	.	.	.	.	1	.
Erysimum crepidifolium	18	.	.	.	.	.	.	.	.	.	1	.	.	.	.	.	.
Digitaria sanguinalis	17	.	.	.	.	.	1	.	.	1	1	1	3	.	.	.	.
Vicia cassubica	17	.	.	.	.	1	1	.	1	.	1	.	.	.	.	.	.
Tephroseris crispa	17	.	3	.	1	1	1	.	.	1	.	.	.	.	.	.	.
Jurinea multiflora	17	.	.	.	.	.	.	.	.	.	1	.	.	.	.	.	.
Poa alpina	17	.	.	.	.	1	1	.	1	.	.	.	.	.	.	2	4
Gymnadenia odoratissima	17	1	.	.	.	1	1	.	1	1	.	.	.	1	.	2	.
Erysimum cheiranthoides	17	.	.	.	.	.	1	.	1	1	1	.	.	1	.	.	.
Gentianella austriaca	17	.	.	.	.	1	1	.	1	1	.	.	.	1	.	1	.
Athyrium filix-femina	17	.	1	.	.	1	1	.	.	.	.	.	.	.	.	.	.
Lolium multiflorum	17	.	.	.	1	.	1	.	.	.	.	.	.	.	.	.	.
Noccaea montana	17	.	.	.	.	1	1	.	.	.	1	.	.	1	1	.	.
Paeonia tenuifolia	17	.	.	.	.	.	.	.	1	1	1	.	.	.	.	.	.
Orchis purpurea	17	.	.	.	.	.	.	.	1	1	.	.	.	.	.	.	.
Fritillaria meleagris	17	.	1	.	1	.	1	.	.	.	.	.	.	.	.	.	.
Geranium rotundifolium	17	.	.	.	.	.	1	.	1	.	1	.	.	1	.	.	.
Dianthus fischeri	17	.	.	.	.	.	1	1	.	.	1	.	.	.	.	.	.
Podospermum laciniatum	17	.	.	.	.	.	.	.	1	.	1	.	.	.	.	.	.
Pulsatilla zimmermannii	17	.	.	.	.	.	.	.	.	1	1	.	1	.	.	.	.
Euphorbia agraria	17	.	.	.	.	.	.	.	.	.	1	.	.	.	.	.	.
Verbascum speciosum	17	.	.	.	.	.	.	1	.	.	1	.	.	.	.	.	.
Digitaria ischaemum	17	.	.	.	.	.	1	.	.	1	1	1	5	.	.	.	.
Pinguicula vulgaris	17	2	1	.	.	1	1	.	.	.	.	.	.	.	.	1	.
Tragopogon podolicus	17	.	.	1	.	.	1	1	.	.	1	.	.	.	.	.	.
Leontodon biscutellifolius	17	.	.	.	.	.	.	1	1	1	1	.	.	.	.	.	.
Ornithogalum pyrenaicum agg.	17	.	.	.	.	1	1	.	1	1	.	.	.	.	.	.	.
Orobanche purpurea	17	.	.	.	.	.	1	.	.	1	1	.	.	1	.	1	.
Phleum hirsutum	16	.	.	.	.	1	1	.	1	1	.	.	.	.	.	1	.
Spiraea crenata	16	.	.	.	.	.	.	.	.	.	1	.	.	.	.	.	3
Sanicula europaea	16	1	.	.	.	1	1	.	1	.	.	.	.	.	.	1	.
Epipactis helleborine	16	.	.	.	.	1	1	.	.	.	1	.	.	1	.	1	1
Lamium galeobdolon	16	.	.	.	1	1	1	.	1	.	1	.	.	.	.	2	3
Sternbergia colchiciflora	16	.	.	.	.	.	.	.	.	.	1	.	.	.	.	.	.
Trifolium angulatum	16	.	.	.	1	.	.	1	.	.	1	.	.	.	.	.	.
Linaria x kocianovichii	16	.	.	.	.	.	.	.	.	1	1	.	.	.	.	.	.
Ferulago campestris	16	.	.	.	.	.	.	1	1	.	1	.	.	.	.	.	.
Aurinia petraea	16	.	.	.	.	.	.	.	.	.	1	.	.	1	.	.	4
Primula farinosa	16	2	.	.	.	.	1	.	1	.	.	.	.	.	.	.	.

Cluster number		1	2	3	4	5	6	7	8	9	10	11	12	13	14	15	16
No. of relevés (all)		257	260	213	797	1411	4434	526	1306	1779	5089	201	203	761	429	219	108
- (with NVP recorded)		133	133	51	234	471	1227	173	409	559	1421	46	121	145	59	100	12
Gagea minima	16	.	.	.	.	.	.	.	.	.	1	.	.	.	1	.	.
Galium sylvaticum	16	.	.	.	.	1	1	.	1	1	1	.	.	.	.	1	.
Hieracium racemosum	16	.	.	.	.	1	.	1	1	1	1	.	.	1	.	.	.
Cardamine amara	16	1	2	.	1	1	1	.	.	.	1	.	.	1	.	.	.
Linum ucranicum	16	.	.	.	.	.	.	.	.	.	1	.	.	.	.	.	.
Glyceria maxima	16	.	.	.	2	.	.	.	.	.	.	.	.	.	.	.	.
Gentiana utriculosa	16	.	.	.	.	.	.	.	1	1	.	.	.	.	.	.	.
Cyanus montanus	16	.	.	.	.	.	.	.	1	1	1	.	.	1	.	4	.
Lathyrus pallescens	15	.	.	.	.	.	1	.	1	1	1	.	.	.	.	.	.
Marrubium vulgare	15	.	.	.	.	.	1	.	.	.	1	.	.	.	.	.	.
Carex disticha	15	1	.	1	1	.	1	.	.	.	.	.	.	.	.	.	.
Bupleurum affine	15	.	.	.	.	.	.	.	.	.	1	.	.	.	.	.	.
Achillea atrata	15	.	.	.	.	.	1	.	1	.	.	.	.	.	.	.	.
Xeranthemum cylindraceum	15	.	.	.	.	.	1	1	1	1	1	.	.	.	.	.	.
Carex pilosa	15	.	.	.	.	1	1	.	1	.	.	.	.	.	.	1	.
Pedicularis sylvatica	15	.	.	.	.	1	1	.	.	.	.	.	.	.	.	.	.
Viola palustris	15	1	3	.	1	1	1	.	.	.	.	.	.	.	.	.	.
Artemisia abrotanum	15	.	.	1	1	.	1	.	.	.	1	.	.	.	.	.	.
Pulsatilla patens	15	.	.	.	.	.	.	.	1	1	1	.	1	1	.	.	.
Trifolium micranthum	15	.	.	.	1	.	1	1	.	.	1	.	.	.	.	.	.
Humulus lupulus	15	1	1	.	1	.	1	.	.	.	1	.	.	1	.	.	.
Stachys alopecuros	14	.	.	.	.	.	1	.	1	.	.	.	.	.	.	.	.
Cirsium waldsteinii	14	.	3	.	1	.	1	.	1	.	.	.	.	.	.	.	.
Asperula purpurea	14	.	.	.	.	.	.	.	.	1	1	.	.	1	.	.	.
Dianthus capitatus	14	.	.	.	.	.	.	.	.	.	1	.	.	.	.	.	.
Galega officinalis	14	.	.	.	1	.	1	1	.	.	.	.	.	.	.	.	.
Polygonum mite	14	.	1	.	1	.	1	.	.	.	1	.	.	.	.	.	.
Veronica beccabunga	14	1	3	.	1	.	1	.	.	.	.	.	.	.	.	.	.
Gypsophila oligosperma	14	.	.	.	.	.	.	.	.	1	1	.	.	.	.	.	.
Centaurea reichenbachii	14	.	.	.	.	.	.	.	.	.	1	.	.	1	.	.	.
Echinochloa crus-galli	14	.	.	.	1	.	1	.	.	.	.	1	.	.	.	.	.
Crepis vesicaria	14	1	.	.	.	.	1	1	1	.	.	.	.	.	.	.	.
Bupleurum tenuissimum	14	.	.	.	1	.	1	1	.	.	1	.	.	.	.	.	.
Inula spiraeifolia	14	.	.	.	.	.	.	.	.	.	1	.	.	.	.	.	.
Veronica agrestis	14	.	.	.	.	.	1	.	.	.	1	.	.	1	.	.	.
Cardamine bulbifera	14	.	.	.	.	1	1	.	1	.	.	.	.	1	.	1	.
Scutellaria supina	14	.	.	.	.	.	.	.	.	.	1	.	.	.	.	.	.
Achillea coarctata	14	.	.	.	.	.	.	.	.	.	1	.	.	1	.	.	.
Stachys annua	14	.	.	.	.	.	.	1	.	1	1	.	.	.	.	.	.
Arenaria longifolia	13	.	.	.	.	.	.	.	.	.	1	.	.	.	.	.	.
Vulpia myuros	13	.	.	.	.	.	1	.	.	.	1	.	.	.	.	.	.
Luzula luzulina	13	.	.	.	.	1	1	.	1	.	.	.	.	.	.	.	.
Vicia sylvatica	13	.	.	.	.	1	1	.	1	.	1	.	.	.	.	1	.
Luzula pilosa	13	.	.	.	.	1	1	.	1	.	1	.	.	.	.	.	.
Melica uniflora	13	.	.	.	.	.	1	.	.	1	1	.	.	1	.	1	.
Triglochin maritima	13	2	.	1	1	.	.	.	.	.	1	.	.	.	.	.	.
Equisetum variegatum	13	1	1	.	.	1	.	.	.	1	1	.	.	.	.	.	.
Cyanus segetum	13	.	1	1	.	.	1	.	.	1	1	.	.	.	.	.	.
Oenanthe fistulosa	13	.	1	.	1	.	1	.	.	.	.	.	.	.	.	.	.
Onosma pseudoarenaria	13	.	.	.	.	.	.	.	.	.	1	1	.	.	.	.	.
Doronicum austriacum	13	.	.	.	.	1	1	.	.	.	.	.	.	.	.	.	.
Euphorbia salicifolia	13	.	.	.	.	.	1	.	1	1	1	.	.	.	.	.	.
Lycopus exaltatus	13	.	.	1	2	.	.	.	.	.	.	.	.	.	.	.	.
Lychnis coronaria	13	.	.	.	.	.	.	1	.	1	1	.	.	.	.	.	.
Festuca gigantea	12	.	1	.	1	1	1	.	.	.	.	.	.	.	.	.	.
Campanula latifolia	12	.	.	.	.	.	1	.	1	.	1	.	.	.	.	.	.
Dianthus collinus	12	.	.	.	.	.	1	.	.	1	1	.	.	.	.	.	.
Draba nemorosa	12	.	.	.	.	.	.	.	.	.	1	1	.	.	.	.	.
Ophrys sphegodes	12	.	.	.	.	.	.	.	1	1	1	.	.	.	.	.	.
Stipa ucrainica	12	.	.	.	.	.	.	.	.	.	1	.	.	.	.	.	.
Onosma heterophylla	12	.	.	.	.	.	.	.	.	.	1	.	.	1	.	.	.
Microrrhinum minus	12	.	.	.	.	.	1	1	.	1	1	.	.	1	.	.	.
Seseli austriacum	12	.	.	.	.	.	.	.	.	.	.	.	.	1	2	.	.
Linaria biebersteinii	12	.	.	.	.	.	.	.	.	.	1	.	.	.	.	.	.
Festuca polesica	12	.	.	.	.	.	.	.	.	.	.	5	1	.	.	.	.
Leucanthemum rotundifolium	12	.	.	.	.	1	1	.	.	.	.	.	.	.	.	3	.
Goniolimon besseranum	12	.	.	.	.	.	.	.	.	.	1	.	.	.	.	.	.
Fumaria officinalis	12	.	.	.	.	.	.	.	.	1	1	1	.	.	.	.	.
Rumex pulcher	12	.	.	.	.	.	1	1	.	.	1	.	1	.	.	.	.
Gladiolus illyricus	12	4	.	.	.	.	1	.	1	.	.	.	.	.	.	.	.
Diplotaxis muralis	12	.	.	.	.	.	.	1	.	1	1	.	.	.	.	.	.
Dianthus carbonatus	12	.	.	.	.	.	.	.	.	.	1	.	.	.	.	.	.
Phyteuma persicifolium	12	.	.	.	.	.	1	.	1	.	.	.	.	.	.	.	.
Spergularia rubra	12	.	.	1	1	.	1	.	.	.	1	.	1	.	.	.	.
Thymus pallasianus	12	.	.	.	.	.	.	.	.	.	1	2	.	.	.	.	.
Carex appropinquata	12	1	2	1	1	.	1	.	.	.	.	.	.	.	.	.	.
Anchusa procera	12	.	.	.	.	.	.	.	.	.	1	.	.	.	.	.	.
Haplophyllum suaveolens	12	.	.	.	.	.	.	.	.	.	1	.	.	.	.	.	.
Tripolium pannonicum	12	1	.	1	1	.	.	.	.	.	1	.	.	.	.	.	.
Cardamine parviflora	12	.	2	.	1	.	1	.	.	.	.	.	.	.	.	.	.
Adonis volgensis	12	.	.	.	.	.	.	.	.	.	1	.	.	.	.	.	.

Cluster number		1	2	3	4	5	6	7	8	9	10	11	12	13	14	15	16
No. of relevés (all)		257	260	213	797	1411	4434	526	1306	1779	5089	201	203	761	429	219	108
- (with NVP recorded)		133	133	51	234	471	1227	173	409	559	1421	46	121	145	59	100	12
Bellevalia speciosa	12	.	.	.	.	.	.	.	.	.	1	.	.	.	.	.	.
Senecio paludosus	12	.	.	.	2	.	.	.	.	.	.	.	.	.	.	.	.
Valerianella rimosa	11	.	.	.	1	.	1	.	1	.	1	.	.	1	.	.	.
Solanum dulcamara	11	.	1	.	1	.	1	1	.	.	.	.	.	.	.	.	.
Sisymbrium loeselii	11	.	.	.	.	.	.	.	.	.	1	.	.	.	.	.	.
Potentilla patula	11	.	.	.	.	.	.	.	1	1	1	.	.	.	.	.	.
Sempervivum tectorum	11	.	.	.	.	.	.	.	.	.	1	.	.	1	.	1	1
Rhinanthus alpinus	11	.	.	.	.	1	1	.	1	1	.	.	.	.	.	2	.
Raphanus raphanistrum	11	.	.	.	.	.	1	1	.	.	1	.	.	.	.	.	.
Orthilia secunda	11	.	.	.	.	.	.	.	.	.	1	3	.	.	.	.	.
Crepis nicaeensis	11	1	.	.	.	.	.	.	1	1	1	.	.	.	.	.	.
Homogyne alpina	11	.	.	.	.	1	.	.	.	.	.	.	.	.	.	1	.
Galium debile	11	.	.	.	1	.	1	.	.	.	.	.	.	.	.	.	.
Grindelia squarrosa	11	.	.	.	.	.	.	.	.	.	1	.	.	.	.	.	.
Achillea crithmifolia	11	.	.	.	.	.	.	.	.	.	1	.	.	.	.	.	.
Platanthera chlorantha	11	.	.	.	.	1	1	.	.	.	.	.	.	1	.	.	.
Glyceria notata	11	.	2	.	1	.	1	.	.	.	.	.	.	.	.	.	.
Galium spurium	11	.	.	.	.	.	.	.	.	.	1	.	.	.	.	.	.
Hieracium glaucum	11	.	.	.	.	.	.	.	.	.	1	.	.	.	2	.	.
Silene multiflora	11	.	.	2	.	.	1	.	.	.	1	.	.	.	.	.	.
Bunias orientalis	11	.	.	.	.	.	1	.	.	1	1	.	.	.	.	.	.
Lathyrus aphaca	11	.	.	.	1	.	1	1	.	1	1	.	.	.	.	.	.
Beckmannia eruciformis	11	.	.	1	1	.	.	.	.	.	.	.	.	.	.	.	.
Matricaria discoidea	10	.	.	.	1	.	1	.	.	.	.	.	.	.	.	.	.
Centaurea solstitialis	10	.	.	.	.	.	.	1	.	.	1	.	.	.	.	.	.
Dianthus campestris	10	.	.	.	.	.	.	.	.	.	1	.	.	.	.	.	.
Euphrasia nemorosa agg.	10	.	.	.	.	1	1	.	.	1	.	.	.	.	.	.	.
Gagea pusilla	10	.	.	.	.	.	.	.	.	1	1	.	.	.	1	.	.
Sisymbrium altissimum	10	.	.	.	.	.	.	.	.	.	1	.	1	.	.	.	.
Moehringia trinervia	10	.	.	.	.	.	1	.	1	1	1	.	.	1	.	1	1
Euphorbia saxatilis	10	.	.	.	.	.	.	.	.	.	.	.	.	.	2	.	.
Aegilops cylindrica	10	.	.	.	.	.	.	.	.	.	1	.	.	.	.	.	.
Pedicularis palustris	10	1	1	.	.	1	1	.	.	.	.	.	.	.	.	.	.
Rindera umbelata	10	.	.	.	.	.	.	.	.	.	1	.	.	.	.	.	.
Onosma tornensis	10	.	.	.	.	.	.	.	.	.	1	.	.	1	1	.	.
Dianthus pseudobarbatus	10	.	.	.	.	.	1	.	.	.	1	.	.	.	.	.	.
Calamagrostis canescens	10	1	1	.	1	.	1	1	.	.	.	.	.	.	.	.	.
Potentilla rupestris	10	.	.	.	.	.	.	.	1	1	1	.	.	.	.	.	.
Spergula arvensis	10	.	.	1	1	.	1	.	.	.	1	1	1	.	.	.	.
Lysimachia punctata	10	1	1	.	1	.	1	.	.	.	.	.	.	.	.	.	.
Carduus personata	10	.	.	.	.	.	1	.	1	.	.	.	.	.	.	.	.
Aposeris foetida	10	.	.	.	.	1	1	.	1	1	1	.	.	.	.	.	.
Impatiens noli-tangere	10	.	1	.	1	.	1	.	.	.	.	.	.	.	.	1	.
Seseli peucedanoides	10	.	.	.	.	.	.	.	1	1	1	.	.	.	.	.	.
Gentianella amarella	10	1	.	.	.	1	.	.	1	1	.	.	.	1	.	1	.
Sherardia arvensis	10	.	.	.	.	.	1	1	.	.	1	.	.	.	.	.	.
Koeleria brevis	10	.	.	.	.	.	.	.	.	.	1	.	.	.	.	.	.
Luzula sylvatica	10	.	.	.	.	1	1	.	.	.	.	.	.	.	.	1	.
Rorippa pyrenaica	10	.	.	.	.	.	1	.	1	1	.	.	.	.	.	.	.
Leonurus cardiaca	10	.	.	.	.	.	1	1	.	.	1	.	.	.	.	.	.
Physospermum cornubiense	10	.	.	.	.	.	.	.	.	1	1	.	.	.	.	.	.
Geranium molle	10	.	.	.	.	.	1	.	.	.	1	.	.	.	.	.	.
Scorzonera mollis	10	.	.	.	.	.	.	.	.	.	1	1	.	.	.	.	.
Tephroseris longifolia	10	.	.	.	.	1	1	.	1	.	.	.	.	.	.	.	.
Hypericum humifusum	10	.	.	.	.	1	1	.	.	.	.	.	.	.	.	.	.
Senecio borysthenicus	10	.	.	.	.	.	.	.	.	.	1	1	.	.	.	.	.
Dryopteris filix-mas	10	.	.	.	.	1	1	.	.	.	1	.	.	.	.	1	.
Orobanche laserpitii-sileris	10	.	.	.	.	.	1	.	1	.	.	.	.	.	.	.	.
Prospero autumnale	10	.	.	.	.	.	.	.	.	.	1	.	.	.	1	.	.
Helictotrichon desertorum	9	.	.	.	.	.	.	.	.	1	1	.	.	.	.	.	.
Silene viridiflora	9	.	.	.	1	.	.	.	.	.	1	.	.	1	.	.	.
Asclepias syriaca	9	.	.	.	1	.	1	1	.	.	1	.	.	.	.	.	.
Pseudorchis albida	9	.	.	.	.	1	.	.	.	.	.	.	.	.	.	.	.
Allium marginatum	9	.	.	.	.	.	.	.	.	1	1	.	.	.	.	.	.
Carex rostrata	9	1	2	.	1	.	.	.	.	.	.	.	.	.	.	.	.
Cytisus podolicus	9	.	.	.	.	.	.	.	1	1	1	.	.	.	.	.	.
Campanula macrostachya	9	.	.	.	.	.	.	.	1	1	1	.	.	.	.	.	.
Teucrium scorodonia	9	.	.	.	.	1	.	.	.	.	1	.	.	.	.	.	.
Iris spuria	9	.	.	.	1	.	.	1	.	.	1	.	.	.	.	.	.
Scorzonera parviflora	9	1	.	1	1	.	1	.	.	.	1	.	.	.	.	.	.
Elytrigia elongata	9	.	.	.	.	.	1	.	.	.	1	.	.	.	.	.	.
Orobanche flava	9	.	.	.	.	.	.	.	.	1	1	.	.	.	.	.	.
Veronica polita	9	.	.	.	.	.	1	1	.	1	1	.	.	.	.	.	.
Pulicaria vulgaris	9	.	.	.	.	.	1	1	.	.	.	.	.	.	.	.	.
Aconitum lycoctonum	9	.	.	.	.	1	.	.	.	.	1	.	.	.	.	1	4
Cypripedium calceolus	9	.	.	.	.	.	.	.	.	1	.	.	.	.	.	1	.
Jurinea salicifolia	9	.	.	.	.	.	.	.	.	.	1	1	.	.	.	.	.
Centaurium littorale	9	2	.	.	1	.	1	.	1	.	.	.	.	.	.	.	.
Petasites albus	9	1	1	.	.	1	1	1	.	.	1	.	.	.	.	1	.
Limonium platyphyllum	9	.	.	.	.	.	1	.	.	.	1	.	.	.	.	.	.
Anagallis foemina	9	.	.	.	.	.	.	.	1	1	1	.	.	.	.	.	.

Cluster number		1	2	3	4	5	6	7	8	9	10	11	12	13	14	15	16
No. of relevés (all)		257	260	213	797	1411	4434	526	1306	1779	5089	201	203	761	429	219	108
- (with NVP recorded)		133	133	51	234	471	1227	173	409	559	1421	46	121	145	59	100	12
Festuca uechtritziana	9	.	.	.	1	.	1	.	.	.	.	.	.	.	.	.	.
Hemerocallis lilioasphodelus	9	1	2	.	.	.	1	.	.	.	.	.	.	.	.	.	.
Senecio viscosus	9	.	.	.	.	.	.	.	.	.	1	.	.	.	.	.	.
Polygala nicaeensis	9	.	.	.	.	.	1	1	1	.	.	.	.	.	.	.	.
Trinia multicaulis	9	.	.	.	.	.	.	.	.	1	1	.	.	.	.	.	.
Orobanche arenaria	9	.	.	.	.	.	.	.	.	.	1	1	.	.	.	.	.
Chaerophyllum temulum	9	.	.	.	1	.	1	1	1	.	1	.	.	.	.	.	.
Erodium ciconium	9	.	.	.	.	.	1	.	.	.	1	.	.	.	.	.	.
Rindera tetraspis	9	.	.	.	.	.	.	.	.	.	1	.	.	.	.	.	.
Cytisus blockianus	9	.	.	.	.	.	.	.	.	1	1	.	.	.	.	.	.
Helictochloa planiculmis	9	.	.	.	.	1	1	1	.	.	.	.	.	.	.	.	.
Portulaca oleracea	9	.	.	.	.	.	1	.	.	.	1	.	1	.	.	.	.
Alopecurus aequalis	9	.	.	.	1	.	1	.	.	.	.	.	.	.	.	.	.
Polemonium caeruleum	9	.	.	.	.	1	1	.	.	.	.	.	.	.	.	.	.
Viola lutea	9	.	.	.	.	1	1	.	1	.	.	.	.	.	.	.	.
Armoracia rusticana	9	.	.	.	1	.	1	.	.	.	.	.	.	.	.	.	.
Myosoton aquaticum	9	.	.	.	1	.	1	.	.	.	.	.	.	.	.	.	.
Papaver albiflorum ssp. austromoravicum	9	.	.	.	.	.	1	.	.	1	1	.	.	.	.	.	.
Veronica bachofenii	9	.	.	.	.	.	.	.	.	.	1	.	.	.	.	.	.
Teesdalia nudicaulis	9	.	.	.	.	1	.	.	.	.	.	.	4	.	.	.	.
Anthyllis schiwereckii	9	.	.	.	.	.	.	.	.	1	1	.	.	.	.	.	.
Helictochloa compressa	8	.	.	.	.	.	.	.	.	1	1	.	.	.	.	.	.
Galatella sedifolia	8	.	.	.	1	.	.	1	.	1	1	.	.	.	.	.	.
Sisyrinchium montanum	8	.	.	.	.	1	1	.	1	.	.	.	.	.	.	.	.
Reseda luteola	8	.	.	.	.	.	1	.	.	.	1	.	.	.	.	.	.
Arenaria rigida	8	.	.	.	.	.	.	.	.	.	1	1	.	.	.	.	.
Dracocephalum austriacum	8	.	.	.	.	.	.	.	.	1	1	.	.	1	.	.	.
Astragalus varius	8	.	.	.	.	.	.	.	.	.	1	2	.	.	.	.	.
Galium abaujense	8	.	.	.	.	.	.	.	.	1	1	.	.	.	.	.	.
Orchis simia	8	.	.	.	.	.	1	.	.	.	1	.	.	.	.	.	.
Scorzoneroides helvetica	8	.	.	.	.	.	1	.	1	.	.	.	.	.	.	.	.
Thalictrum uncinatum	8	.	.	.	.	.	.	.	.	1	1	.	.	.	.	.	.
Adenophora liliifolia	8	.	.	.	.	.	1	.	1	1	.	.	.	.	.	.	.
Artemisia annua	8	.	.	.	.	.	.	.	.	.	1	.	.	.	.	.	.
Limonium aureum	8	.	.	1	1	.	.	1	.	.	1	.	.	.	.	.	.
Trisetum sibiricum	8	.	.	1	.	.	.	.	.	.	1	.	.	.	.	.	.
Hordeum murinum	8	.	.	.	1	.	.	.	.	.	1	.	.	.	.	.	.
Schivereckia podolica	8	.	.	.	.	.	.	.	.	.	1	.	.	.	.	.	.
Filago lutescens	8	.	.	.	.	.	1	.	.	.	1	.	.	.	.	.	.
Anthericum liliago	8	.	.	.	.	.	.	.	.	1	1	.	.	.	.	.	.
Carex depressa ssp. transsilvanica	8	.	.	.	.	1	.	.	1	.	1	.	.	.	.	.	.
Solanum nigrum	8	.	.	.	.	.	1	1	.	.	1	.	1	.	.	.	.
Carex buekii	8	.	1	.	1	.	1	.	.	.	.	.	.	.	.	.	.
Crepis pulchra	8	.	.	.	.	.	1	.	.	.	1	.	.	.	.	.	.
Allium inaequale	8	.	.	.	.	.	.	.	.	.	1	.	.	.	.	.	.
Valerianella coronata	8	.	.	.	.	.	.	.	.	.	1	.	.	.	.	.	.
Carex capillaris	8	.	.	.	.	.	1	.	.	.	.	.	.	.	.	.	.
Lotus angustissimus	8	.	.	.	1	.	.	1	.	.	1	.	.	.	.	.	.
Chrysosplenium alternifolium	8	.	1	.	1	.	1	.	.	.	.	.	.	.	.	1	.
Veronica barrelieri	8	.	.	.	.	.	.	.	.	1	1	.	.	.	.	.	.
Anchusa gmelinii	8	.	.	.	.	.	.	.	.	.	1	2	.	.	.	.	.
Laserpitium archangelica	8	.	.	.	.	.	1	.	.	.	.	.	.	.	.	3	.
Himantoglossum adriaticum	8	.	.	.	.	.	.	.	1	1	1	.	.	.	1	.	.
Piptatherum virescens	8	.	.	.	.	.	.	.	.	1	1	.	.	1	.	.	.
Aconitum confertiflorum	7	.	.	.	.	.	.	.	1	1	1	.	.	.	.	.	.
Astragalus peterfii	7	.	.	.	.	.	.	.	.	.	1	.	.	.	.	.	.
Achillea micrantha	7	.	.	.	.	.	.	1	.	.	1	1	.	.	.	.	.
Erodium ruthenicum	7	.	.	.	.	.	.	.	.	.	1	.	.	.	.	.	.
Alyssum repens	7	.	.	.	.	.	.	.	.	.	1	.	.	1	.	.	2
Impatiens parviflora	7	.	1	.	1	.	1	.	.	1	1	.	.	1	.	.	.
Allium suaveolens	7	1	.	.	.	.	1	.	1	.	.	.	.	.	.	.	.
Allium fuscum	7	.	.	.	.	.	.	.	.	.	1	.	.	.	.	.	.
Galium cracoviense	7	.	.	.	.	.	.	.	.	1	1	.	.	.	.	.	.
Ranunculus pseudomontanus	7	.	.	.	.	1	.	.	.	.	.	.	.	.	.	3	.
Rumex aquaticus	7	.	1	.	.	.	1	.	.	.	.	.	.	.	.	.	.
Xanthium spinosum	7	.	.	.	1	.	.	.	.	.	1	.	.	.	.	.	.
Malva moschata	7	.	.	.	.	.	1	1	.	1	.	.	.	.	.	.	.
Swertia perennis	7	1	.	.	.	.	.	.	.	.	.	.	.	.	.	2	.
Draba muralis	7	.	.	.	.	.	1	.	.	.	1	.	.	1	.	.	.
Festuca amethystina	7	.	.	.	.	.	1	.	1	.	.	.	.	.	.	1	.
Bartsia alpina	7	.	.	.	.	.	.	.	.	.	.	.	.	.	.	3	.
Jovibarba heuffelii	7	.	.	.	.	.	.	.	.	.	1	.	.	.	.	.	2
Rumex hydrolapathum	7	.	1	.	1	.	1	.	.	.	.	.	.	.	.	.	.
Melampyrum polonicum	7	.	.	.	.	.	1	.	.	1	1	.	.	.	.	.	.
Aristolochia pallida	7	.	.	.	.	1	1	.	.	1	1	.	.	.	.	.	.
Centaurea salonitana	7	.	.	.	.	.	.	.	.	.	1	.	.	.	.	.	.
Acorus calamus	7	.	1	.	1	.	.	.	.	.	.	.	.	.	.	.	.
Lilium carniolicum	7	.	.	.	.	.	1	.	1	1	.	.	.	.	.	.	.
Alopecurus rendlei	7	.	.	.	1	.	1	.	.	1	.	.	.	.	.	.	.
Alopecurus arundinaceus	7	.	.	.	1	.	.	.	.	.	1	.	.	.	.	.	.

Cluster number		1	2	3	4	5	6	7	8	9	10	11	12	13	14	15	16
No. of relevés (all)		257	260	213	797	1411	4434	526	1306	1779	5089	201	203	761	429	219	108
- (with NVP recorded)		133	133	51	234	471	1227	173	409	559	1421	46	121	145	59	100	12
Cirsium brachycephalum	7	1	.	.	1	.	1	.	.	.	.	.	.	.	.	.	.
Oenanthe banatica	7	.	.	.	1	.	1	.	.	.	.	.	.	.	.	.	.
Adonis aestivalis	7	.	.	.	.	.	1	.	.	.	1	.	.	.	.	.	.
Agrimonia procera	7	.	.	.	.	.	1	.	.	.	1	.	.	.	.	.	.
Carex diluta	7	.	.	1	1	.	.	.	.	.	1	.	.	.	.	.	.
Asperula tenella	7	.	.	.	.	.	.	.	.	.	1	.	.	.	.	.	4
Polygonum graminifolium	7	.	.	.	.	.	.	.	.	.	.	.	3	.	.	.	.
Cyclamen purpurascens	7	.	.	.	.	.	.	.	1	1	.	.	.	1	.	.	.
Sedum dasyphyllum	7	.	.	.	.	.	.	.	.	.	1	1	.	.	.	.	2
Artemisia santonicum	7	.	.	.	1	.	.	1	.	.	1	1	.	.	.	.	.
Colchicum arenarium	7	.	.	.	.	.	.	.	.	.	1	2	.	.	.	.	.
Chaerophyllum aureum	7	.	.	.	1	.	1	.	.	.	.	.	.	.	.	.	.
Juncus filiformis	7	.	1	.	.	.	1	.	.	.	.	.	.	.	.	.	.
Dianthus bessarabicus	6	.	.	.	.	.	.	.	.	.	.	3	.	.	.	.	.
Veronica spuria	6	.	.	.	.	.	.	.	.	1	1	.	.	.	.	.	.
Diplotaxis tenuifolia	6	.	.	.	.	.	.	1	.	.	1	.	.	.	.	.	.
Syrenia montana	6	.	.	.	.	.	.	.	.	.	1	1	.	.	.	.	.
Asplenium x alternifolium	6	.	.	.	.	.	.	.	.	.	1	.	.	1	.	.	3
Carex remota	6	.	1	.	.	.	1	.	.	.	.	.	.	.	.	.	.
Helminthotheca echioides	6	.	.	.	.	.	.	1	.	.	1	.	.	.	.	.	.
Bromus secalinus	6	.	.	1	1	.	1	.	.	.	1	.	.	.	.	.	.
Tulipa biebersteiniana	6	.	.	.	.	.	.	.	.	.	1	.	.	.	.	.	.
Spiranthes spiralis	6	1	.	.	.	1	.	.	1	1	.	.	.	.	.	.	.
Centaurea stereophylla	6	.	.	.	.	.	.	.	.	.	1	.	.	.	.	.	.
Seseli montanum ssp. tommasinii	6	.	.	.	.	.	.	.	.	1	1	.	.	1	.	.	.
Atriplex patula	6	.	.	.	1	.	1	1	.	.	1	.	.	.	.	.	.
Viola accrescens	6	.	.	.	.	.	.	.	.	.	1	.	.	.	.	.	.
Aira caryophyllea	6	.	.	.	.	.	1	.	.	.	1	.	.	.	.	.	.
Euphorbia leptocaula	6	.	.	.	.	.	.	.	.	.	1	.	.	.	.	.	.
Centaurea nigra	6	.	.	.	.	1	1	.	.	.	.	.	.	.	.	.	.
Euphrasia parviflora	6	.	.	.	.	.	.	.	.	1	1	.	.	.	.	.	.
Cortusa matthioli	6	.	.	.	.	.	1	.	.	.	.	.	.	.	.	2	1
Gagea bulbifera	6	.	.	.	.	.	.	.	.	.	1	1	.	.	.	.	.
Rhaponticoides ruthenica	6	.	.	.	.	.	.	.	.	1	1	.	.	.	.	.	.
Ornithogalum comosum	6	.	.	.	.	.	.	.	.	.	1	.	.	.	1	.	.
Asplenium cuneifolium	6	.	.	.	.	.	.	.	.	1	1	.	.	1	.	.	.
Epilobium roseum	6	.	.	.	1	.	1	1	.	.	.	.	.	.	.	.	.
Orobanche coerulescens	6	.	.	.	.	.	.	.	.	.	1	.	.	.	.	1	.
Viola hymettia	6	.	.	.	.	.	.	.	.	.	.	3	.	.	.	.	.
Aethusa cynapium	6	.	.	1	1	.	.	.	.	.	1	.	.	.	.	.	.
Leontopodium nivale ssp. alpinum	6	.	.	.	.	.	.	.	.	.	.	.	.	.	.	3	.
Bromus benekenii	6	.	.	.	.	.	1	.	.	.	1	.	.	.	.	.	.
Nepeta cataria	6	.	.	.	.	.	1	.	1	1	1	.	.	.	.	.	.
Waldsteinia geoides	6	.	1	.	.	.	.	.	.	1	1	.	.	.	.	.	.
Barbarea stricta	6	.	.	.	1	.	1	.	.	.	.	.	.	.	.	.	.
Mentha x dumetorum	6	.	.	.	1	.	1	.	.	.	1	.	.	.	.	.	.
Bupleurum longifolium	6	.	.	.	.	.	.	.	.	.	1	.	.	1	1	1	.
Centaurea triniifolia	6	.	.	.	.	.	.	.	.	.	1	.	.	1	.	.	.
Epilobium ciliatum	6	.	1	.	.	.	1	.	.	.	1	.	.	.	.	.	.
Dianthus moravicus	6	.	.	.	.	.	.	.	.	.	1	.	.	1	.	.	.
Malabaila graveolens	6	.	.	.	.	.	.	.	.	.	1	.	.	.	.	.	.
Iris pontica	6	.	.	.	.	.	.	.	1	1	1	.	.	.	.	.	.
Rorippa amphibia	6	.	.	.	1	.	.	.	.	.	.	.	.	.	.	.	.
Hedysarum grandiflorum	6	.	.	.	.	.	.	.	.	.	1	.	.	.	.	.	.
Equisetum hyemale	6	.	.	.	.	.	1	.	.	1	1	.	.	.	.	.	.
Asperula rumelica	6	.	.	.	.	.	.	.	.	.	1	.	.	.	.	.	.
Thymus calcareus	6	.	.	.	.	.	.	.	.	.	1	.	.	.	.	.	.
Galinsoga parviflora	6	.	.	.	1	.	1	.	.	.	1	.	.	.	.	.	.
Gladiolus tenuis	6	.	.	1	1	.	1	.	.	.	.	.	.	.	.	.	.
Podospermum roseum	6	.	.	.	.	1	1	.	.	1	.	.	.	.	.	.	.
Dianthus henteri	6	.	.	.	.	.	.	.	.	.	1	.	.	.	.	.	.
Carduus uncinatus	6	.	.	.	.	.	.	.	.	.	1	.	.	.	.	.	.
Elytrigia stipifolia	6	.	.	.	.	.	.	.	.	.	1	.	.	.	.	.	.
Astrantia carniolica	6	.	.	.	.	.	1	.	1	.	.	.	.	.	.	.	.
Geranium lucidum	6	.	.	.	.	.	.	.	.	.	1	.	.	.	.	.	2
Lepidium perfoliatum	6	.	.	.	.	.	1	.	.	.	1	.	.	.	.	.	.
Lathyrus linifolius	6	.	1	.	.	1	1	.	.	.	.	.	.	.	.	.	.
Digitalis ferruginea	6	.	.	.	.	.	.	1	1	1	.	.	.	.	.	.	.
Neslia paniculata	6	.	.	.	.	.	1	.	.	.	1	.	.	.	.	.	.
Hieracium prenanthoides	6	.	.	.	.	1	.	.	1	1	.	.	.	.	.	1	.
Scorzonera villosa	6	.	.	.	.	.	1	.	1	1	.	.	.	.	.	.	.
Satureja kitaibelii	6	.	.	.	.	.	.	.	.	.	1	.	.	.	.	.	1
Rumex sanguineus	6	.	1	.	1	.	1	.	.	.	1	.	.	.	.	.	.
Rorippa palustris	6	.	.	.	1	.	1	.	.	.	.	.	.	.	.	.	.
Peucedanum austriacum	5	.	.	.	.	.	1	.	1	.	.	.	.	.	.	.	2
Campanula crassipes	5	.	.	.	.	.	.	.	.	.	1	.	.	.	.	.	4
Lactuca quercina	5	.	.	.	.	.	1	.	.	.	1	.	.	.	.	.	.
Leonurus quinquelobatus	5	.	.	.	.	.	1	1	.	.	1	.	.	.	.	.	.
Melilotus sulcatus	5	.	.	.	.	.	.	.	.	.	1	.	.	1	.	.	.
Pedicularis hacquetii	5	.	.	.	.	.	1	.	1	.	.	.	.	.	.	.	.
Fumana vulgaris	5	.	.	.	.	.	.	.	1	1	.	.	.	.	.	.	.

Cluster number		1	2	3	4	5	6	7	8	9	10	11	12	13	14	15	16
No. of relevés (all)		257	260	213	797	1411	4434	526	1306	1779	5089	201	203	761	429	219	108
- (with NVP recorded)		133	133	51	234	471	1227	173	409	559	1421	46	121	145	59	100	12
Vincetoxicum fuscatum	5	.	.	.	.	.	.	.	.	.	1	.	.	.	.	.	.
Cephalanthera damasonium	5	.	.	.	.	.	.	.	1	1	.	.	.	1	.	1	.
Rumex stenophyllus	5	.	.	1	1	.	.	.	.	.	.	.	.	.	.	.	.
Atriplex sagittata	5	.	.	.	.	.	.	.	.	.	1	.	.	.	.	.	.
Koeleria grandis	5	.	.	.	.	.	.	1	.	.	1	.	.	.	.	.	.
Ranunculus alpestris	5	.	.	.	.	.	.	.	.	.	.	.	.	.	.	2	.
Arabis pauciflora	5	.	.	.	.	.	.	.	1	1	.	.	.	1	.	.	.
Cymbaria borysthenica	5	.	.	.	.	.	.	.	.	.	1	.	.	.	.	.	.
Glycyrrhiza echinata	5	.	.	.	1	.	.	1	.	.	1	.	.	.	.	.	.
Botrychium matricariifolium	5	.	.	.	.	1	.	.	.	.	.	.	.	.	.	.	.
Malva neglecta	5	.	.	.	1	.	1	.	.	.	1	.	.	.	.	.	.
Aruncus dioicus	5	.	.	.	.	1	.	.	.	.	1	.	.	.	.	1	.
Anemone narcissiflora	5	.	.	.	.	.	.	.	1	1	.	.	.	.	.	.	.
Astragalus albicaulis	5	.	.	.	.	.	.	.	.	.	1	.	.	.	.	.	.
Saponaria glutinosa	5	.	.	.	.	.	.	.	.	.	1	.	.	.	.	.	.
Limonium tomentellum	5	.	.	.	.	.	.	.	.	.	1	.	.	.	.	.	.
Doronicum hungaricum	5	.	.	.	.	.	.	.	.	1	1	.	.	.	.	.	.
Hierochloe repens	5	.	.	.	.	.	.	.	.	.	1	.	.	.	.	.	.
Polycnemum majus	5	.	.	.	.	.	.	.	.	.	1	.	.	.	.	.	.
Gentianella fatrae	5	.	.	.	.	.	.	.	.	.	.	.	.	1	.	2	.
Hierochloe australis	5	.	.	.	.	.	.	.	.	1	1	.	.	1	.	.	1
Aphanes arvensis	5	.	.	.	.	.	1	.	.	.	1	.	.	.	.	.	.
Dianthus eugeniae	5	.	.	.	.	.	.	.	.	.	1	.	.	.	.	.	.
Astragalus ponticus	5	.	.	.	.	.	.	.	.	.	1	.	.	.	.	.	.
Pinguicula alpina	5	1	.	.	.	.	.	.	.	.	.	.	.	.	.	1	.
Pulmonaria dacica	5	.	.	.	.	.	1	.	.	.	1	.	.	.	.	.	.
Anthemis austriaca	5	.	.	.	.	.	.	.	.	1	1	.	1	.	.	.	.
Trifolium badium	5	.	1	.	.	1	.	.	.	1	1	.	.	.	.	.	.
Sagina saginoides	5	.	.	.	.	1	.	.	.	.	.	.	.	.	.	.	.
Chenopodium glaucum	5	.	.	.	1	.	.	.	.	.	1	.	.	.	.	.	.
Sporobolus vaginiflorus	5	.	.	.	.	.	1	1	.	.	1	.	.	.	.	.	.
Arabis alpina	5	.	.	.	.	.	.	.	.	.	.	.	.	1	.	1	.
Crepis pannonica	5	.	.	.	.	.	.	.	.	1	1	.	.	.	.	.	.
Allium denudatum	5	.	.	.	.	.	.	.	.	.	1	1	.	.	.	.	.
Ranunculus platanifolius	5	.	.	.	.	1	1	.	.	.	.	.	.	.	.	.	.
Eragrostis minor	5	.	.	.	.	.	.	.	.	.	1	.	.	.	.	.	.
Onobrychis gracilis	5	.	.	.	.	.	.	.	.	.	1	.	.	.	.	.	.
Minuartia taurica	5	.	.	.	.	.	.	.	.	.	1	.	.	.	.	.	.
Gypsophila repens	5	.	.	.	.	.	.	.	1	1	.	.	.	.	.	1	.
Pedicularis acaulis	5	.	.	.	.	.	.	.	1	.	.	.	.	.	.	.	.
Trifolium diffusum	5	.	.	.	.	.	.	.	.	.	1	.	1	.	.	.	.
Veronica peregrina	5	.	.	.	1	.	1	.	.	.	1	.	.	.	.	.	.
Galium verrucosum	5	.	.	.	.	.	1	.	1	.	1	.	.	.	.	.	.
Equisetum x litorale	5	1	.	.	.	.	1	.	.	.	.	.	.	.	.	.	.
Aira elegantissima	5	.	.	.	.	1	.	.	.	1	1	.	.	.	.	.	.
Cirsium esculentum	5	.	.	1	1	.	1	.	.	.	.	.	.	.	.	.	.
Sorghum halepense	5	.	.	.	1	.	1	.	.	.	1	.	.	.	.	.	.
Cirsium alatum	5	.	.	1	1	.	.	.	.	.	.	.	.	.	.	.	.
Trifolium incarnatum	5	.	.	.	.	.	1	1	.	.	.	.	.	.	.	.	.
Polygala cretacea	5	.	.	.	.	.	.	.	.	.	1	.	.	.	.	.	.
Centaurea pugioniformis	5	.	.	.	.	.	.	.	1	1	.	.	.	.	.	.	.
Sisymbrium strictissimum	5	.	.	.	.	.	.	.	.	1	1	.	.	.	.	.	.
Alisma lanceolatum	5	.	.	.	1	.	.	.	.	.	.	.	.	.	.	.	.
Oenanthe aquatica	5	.	.	.	1	.	.	.	.	.	.	.	.	.	.	.	.
Trifolium subterraneum	5	.	.	.	1	.	1	1	.	.	1	.	.	.	.	.	.
Equisetum x moorei	5	1	.	.	.	.	.	.	.	.	.	.	1	.	.	.	.
Taraxacum bessarabicum	5	1	.	.	1	.	.	.	.	.	1	.	.	.	.	.	.
Erysimum repandum	5	.	.	.	.	.	1	.	.	1	1	1	.	.	.	.	.
Salsola tragus	5	.	.	.	.	.	.	.	.	.	1	1	1	.	.	.	.
Bassia scoparia	5	.	.	.	.	.	1	1	.	.	.	.	.	.	.	.	.
Lycopsis arvensis	5	.	.	.	.	1	1	.	.	.	1	.	.	.	.	.	.
Orobanche reticulata	5	.	.	.	.	.	1	1	.	.	1	.	.	.	.	1	.
Aremonia agrimonoides	5	.	.	.	.	1	1	1	1	.	.	.	.	.	.	.	.
Conium maculatum	5	.	.	.	.	.	1	1	.	.	1	.	.	.	.	.	.
Carex pediformis	5	.	.	.	.	.	.	.	.	1	1	.	.	1	.	1	.
Reseda phyteuma	5	.	.	.	.	.	.	.	.	.	1	.	.	.	1	.	.
Vicia dumetorum	5	.	1	.	.	.	1	1	1	1	.	.	.	.	.	.	.
Scabiosa argentea	5	.	.	.	.	.	.	.	.	.	1	1	.	.	.	.	.
Trigonella gladiata	5	.	.	.	.	.	.	.	.	.	1	.	.	.	.	.	.
Dianthus platyodon	5	.	.	.	.	.	.	.	.	.	1	1	.	.	.	.	.
Rumex maritimus	4	.	.	.	1	.	.	.	.	.	.	.	.	.	.	.	.
Puccinellia distans	4	.	.	.	1	.	1	.	.	.	.	.	.	.	.	.	.
Melilotus dentatus	4	.	.	.	1	.	.	.	.	.	.	.	.	.	.	.	.
Epilobium collinum	4	.	.	.	.	.	1	.	.	.	1	.	.	.	.	.	.
Cannabis sativa	4	.	.	.	.	.	.	.	.	.	1	1	.	.	.	.	.
Stachys alpina	4	.	1	.	.	.	1	.	.	.	.	.	.	.	.	.	2
Hyoscyamus niger	4	.	.	.	.	.	.	.	.	.	1	.	.	.	.	.	.
Onosma simplicissima	4	.	.	.	.	.	.	.	.	.	1	.	.	.	.	.	.
Noccaea kovatsii	4	.	.	.	.	.	1	.	1	.	1	.	.	.	.	.	.
Thesium dollineri	4	.	.	.	.	.	.	.	.	.	1	.	.	.	.	.	.
Geranium collinum	4	.	.	1	1	.	.	1	.	.	.	.	.	.	.	.	.

Cluster number		1	2	3	4	5	6	7	8	9	10	11	12	13	14	15	16
No. of relevés (all)		257	260	213	797	1411	4434	526	1306	1779	5089	201	203	761	429	219	108
- (with NVP recorded)		133	133	51	234	471	1227	173	409	559	1421	46	121	145	59	100	12
Bupleurum pachnospermum	4	.	.	.	.	.	.	.	.	.	1	.	.	.	.	.	.
Ajuga orientalis	4	.	.	.	.	.	.	.	.	.	1	.	.	.	.	.	.
Cyanus adscendens	4	.	.	.	.	.	.	.	.	1	.	.	.	.	.	.	.
Potentilla chrysantha	4	.	.	.	.	.	.	.	1	.	1	.	.	.	.	.	.
Dianthus pallens	4	.	.	.	.	.	.	.	.	.	1	.	.	.	.	.	.
Androsace koso-poljanskii	4	.	.	.	.	.	.	.	.	.	1	.	.	.	.	.	.
Typha angustifolia	4	1	.	.	1	.	.	.	.	.	.	.	.	.	.	.	.
Centaurea lavrenkoana	4	.	.	.	.	.	.	.	.	.	1	.	.	.	.	.	.
Myosurus minimus	4	.	.	.	1	.	.	.	.	.	.	.	.	.	.	.	.
Lepidium ruderale	4	.	.	.	.	.	.	.	.	.	1	.	.	.	.	.	.
Blackstonia perfoliata	4	.	.	.	.	.	1	.	.	.	1	.	.	.	.	.	.
Saxifraga cuneifolia	4	.	.	.	.	.	.	.	.	.	.	.	.	.	.	.	4
Cirsium ukranicum	4	.	.	.	.	.	.	.	.	.	1	.	.	.	.	.	.
Tragopogon borysthenicus	4	.	.	.	.	.	.	.	.	.	1	.	.	.	.	.	.
Liparis loeselii	4	1	.	.	.	.	1	.	.	.	.	.	.	.	.	.	.
Pilosella floribunda	4	.	1	.	.	.	.	.	1	.	.	.	.	.	.	.	.
Alyssum hirsutum	4	.	.	.	.	.	.	.	.	.	1	1	.	.	.	.	.
Amaranthus retroflexus	4	.	.	.	1	.	.	.	.	.	1	.	1	.	.	.	.
Helleborus dumetorum	4	.	.	.	.	.	1	.	.	.	.	.	.	.	.	.	.
Orobanche artemisiae-campestris	4	.	.	.	.	.	.	.	.	1	1	.	.	.	.	.	.
Rudbeckia laciniata	4	.	1	.	.	.	1	.	.	.	.	.	.	.	.	.	.
Piptatherum holciforme	4	.	.	.	.	.	.	.	.	.	1	.	.	.	.	.	.
Saxifraga adscendens	4	.	.	.	.	.	.	.	.	.	.	.	.	.	.	.	4
Bolboschoenus maritimus s.lat.	4	1	.	.	1	.	.	1	.	.	.	.	.	.	.	.	.
Anthemis odontostephana	4	.	.	.	.	.	1	1	.	.	.	.	.	.	.	.	.
Gnaphalium norvegicum	4	.	.	.	.	1	1	.	.	.	.	.	.	.	.	.	.
Arctium minus	4	.	.	.	.	.	1	1	.	.	1	.	.	.	.	.	.
Orobanche alsatica	4	.	.	.	.	.	.	.	1	1	.	.	.	.	.	.	.
Carduus carduelis	4	.	.	.	.	.	1	.	.	.	1	.	.	.	.	.	.
Bupleurum praealtum	4	.	.	.	.	.	.	.	.	.	1	.	.	.	.	.	.
Carex elongata	4	1	.	.	1	.	1	.	.	.	.	.	.	.	.	.	.
Paris quadrifolia	4	.	1	.	.	.	1	.	1	.	.	.	.	.	.	1	.
Geranium sibiricum	4	.	.	.	.	.	.	1	.	.	1	.	.	.	.	.	.
Polygonum patulum	4	.	.	.	.	.	1	.	.	.	1	.	.	.	.	.	.
Galium volhynicum	4	.	.	.	.	.	.	.	.	.	1	.	.	.	.	.	.
Medicago monspeliaca	4	.	.	.	.	.	.	.	.	.	1	.	.	.	.	.	.
Pilosella guthnikiana	4	.	.	.	.	.	1	.	.	.	.	.	.	.	.	.	.
Silene subconica	4	.	.	.	.	.	.	.	.	.	1	1	.	.	.	.	.
Gagea lutea	4	.	.	.	.	.	1	.	.	.	1	.	1	.	.	.	.
Tribulus terrestris	4	.	.	.	.	.	.	.	.	.	1	1	.	.	.	.	.
Erysimum sylvestre	4	.	.	.	.	.	.	.	.	.	.	.	.	1	1	.	.
Angelica palustris	4	.	1	1	1	.	1	.	.	.	.	.	.	.	.	.	.
Malva sylvestris	4	.	.	.	.	.	1	.	.	1	.	.	.	.	.	.	.
Veronica urticifolia	4	.	.	.	.	.	.	.	.	.	.	.	.	.	.	.	4
Linum dolomiticum Borb.	4	.	.	.	.	.	.	.	.	.	.	.	.	1	1	.	.
Stipa asperella	4	.	.	.	.	.	.	.	.	.	1	.	.	.	.	.	.
Hieracium caesium	4	.	.	.	.	.	.	.	.	.	1	.	.	.	.	1	.
Asperula tephrocarpa	4	.	.	.	.	.	.	.	.	.	1	.	.	.	.	.	.
Androsace lactea	4	.	.	.	.	.	.	.	.	.	.	.	.	.	.	2	.
Schoenoplectus lacustris	4	1	.	.	.	.	1	.	.	.	.	.	.	.	.	.	.
Minuartia hybrida	4	.	.	.	.	.	.	.	.	.	1	.	.	.	.	.	.
Asperula neilreichii	4	.	.	.	.	.	.	.	.	.	.	.	.	1	.	1	.
Medicago arabica	4	.	.	.	1	.	1	1	.	.	.	.	.	.	.	.	.
Ornithogalum boucheanum	4	.	.	.	.	.	.	.	.	.	1	.	.	.	.	.	1
Dianthus hypanicus	4	.	.	.	.	.	.	.	.	.	1	.	.	.	.	.	.
Euphorbia bessarabica	4	.	.	.	.	.	.	.	.	.	1	.	.	.	.	.	.
Fumaria schleicheri	4	.	.	.	.	.	.	.	.	1	1	.	.	.	.	.	.
Fumaria vaillantii	4	.	.	.	.	.	.	.	.	.	1	.	.	1	.	.	.
Hordeum secalinum	4	.	.	.	1	.	1	.	.	.	.	.	.	.	.	.	.
Rorippa brachycarpa	4	.	.	1	1	.	.	.	.	.	.	.	.	.	.	.	.
Koeleria moldavica	4	.	.	.	.	.	.	.	.	.	1	.	.	.	.	.	.
Helleborus niger	4	.	.	.	.	.	1	.	1	.	.	.	.	.	.	.	.
Onobrychis alba	4	.	.	.	.	.	.	.	.	.	1	.	.	.	.	.	.
Prenanthes purpurea	4	.	.	.	.	.	.	.	.	.	.	.	.	.	.	2	.
Clinopodium menthifolium	4	.	.	.	.	.	.	.	.	1	.	.	.	.	.	.	1
Viola biflora	4	.	.	.	.	.	.	.	.	.	.	.	.	.	.	2	.
Cerastium banaticum	4	.	.	.	.	.	.	.	.	.	1	.	.	1	.	.	1
Silene armeria	4	.	.	.	.	.	.	.	.	.	1	.	.	1	.	.	.
Carex dioica	4	2	.	.	.	.	.	.	.	.	.	.	.	.	.	.	.
Neottia nidus-avis	4	.	.	.	.	.	1	.	1	.	.	.	.	1	.	.	.
Gnaphalium uliginosum	4	1	.	.	.	.	1	.	.	.	1	.	.	.	.	.	.
Scrophularia umbrosa	4	1	1	.	.	.	.	.	.	.	.	.	.	.	.	.	.
Scrophularia scopolii	4	.	.	.	.	.	1	.	.	1	1	.	.	.	.	.	.
Vicia ervilia	4	.	.	.	1	.	1	.	.	.	.	.	.	.	.	.	.
Plantago cornuti	4	.	.	1	1	.	.	.	.	.	.	.	.	.	.	.	.
Festuca altissima	4	.	.	.	.	.	1	.	.	1	.	.	.	.	.	1	.
Poa annua	4	.	.	.	.	.	1	.	.	.	1	.	.	.	.	.	.
Malaxis monophyllos	4	.	.	.	.	.	1	.	1	1	.	.	.	.	.	1	.
Astragalus pseudotataricus	3	.	.	.	.	.	.	.	.	.	1	.	.	.	.	.	.
Poa stiriaca	3	.	.	.	.	.	.	.	.	1	1	.	.	.	.	1	.
Arctium nemorosum	3	.	.	.	.	.	1	.	.	.	1	1	.	.	.	.	.

Cluster number		1	2	3	4	5	6	7	8	9	10	11	12	13	14	15	16
No. of relevés (all)		257	260	213	797	1411	4434	526	1306	1779	5089	201	203	761	429	219	108
- (with NVP recorded)		133	133	51	234	471	1227	173	409	559	1421	46	121	145	59	100	12
Digitalis lanata	3	.	.	.	.	.	.	.	.	1	1	.	.	.	.	.	.
Lathyrus sphaericus	3	.	.	.	.	.	.	.	.	.	1	.	.	.	.	.	.
Glyceria nemoralis	3	.	1	.	1	.	.	.	.	.	.	.	.	.	.	.	.
Vaccaria hispanica	3	.	.	.	.	.	1	.	1	.	.	.	.	.	.	.	.
Lythrum hyssopifolia	3	.	.	.	1	.	.	.	.	.	1	.	.	.	.	.	.
Euphorbia falcata	3	.	.	.	.	.	.	.	1	.	1	.	.	.	.	.	.
Argusia sibirica	3	.	.	.	.	.	.	.	.	.	.	1	.	.	.	.	.
Malva alcea	3	.	.	.	.	.	.	1	.	1	.	.	.	.	.	.	.
Campanula thyrsoides	3	.	.	.	.	.	.	.	.	1	.	.	.	1	.	.	.
Cucubalus baccifer	3	.	.	.	.	.	1	.	.	1	.	.	.	.	.	.	.
Chenopodium bonus-henricus	3	.	.	.	1	.	1	.	.	.	.	.	.	.	.	.	.
Erodium moschatum	3	.	.	.	.	.	.	.	.	.	1	.	.	.	.	.	.
Gypsophila petraea	3	.	.	.	.	.	.	.	.	.	.	.	.	1	.	.	1
Cuscuta campestris	3	.	.	.	.	.	.	.	.	1	1	.	.	.	.	.	.
Ligusticum mutellina	3	.	.	.	.	1	1	.	.	1	.	.	.	.	.	.	.
Rumex scutatus	3	.	.	.	.	.	.	1	.	.	.	.	.	.	.	1	.
Saxifraga aizoides	3	.	.	.	.	.	.	.	.	.	.	.	.	1	.	1	.
Lepidium virginicum	3	.	.	.	.	.	1	.	.	.	.	.	1	.	.	.	.
Foeniculum vulgare	3	.	.	.	.	.	.	.	1	1	.	.	.	.	.	.	.
Cephalanthera longifolia	3	.	.	.	.	.	.	.	1	.	.	.	.	.	.	.	.
Limonium suffruticosum	3	.	.	1	.	.	1	.	.	.	.	.	.	.	.	.	.
Dichoropetalum schottii	3	.	.	.	.	.	.	.	1	1	.	.	.	.	.	.	.
Arenaria grandiflora	3	.	.	.	.	.	.	.	.	.	1	.	.	.	.	1	.
Iva xanthiifolia	3	.	.	.	1	.	.	.	.	.	1	.	.	.	.	.	.
Leymus racemosus	3	.	.	.	.	.	.	.	.	.	.	1	.	.	.	.	.
Noccaea brachypetala	3	.	.	.	.	1	1	.	.	.	.	.	.	.	.	.	.
Euphorbia semivillosa	3	.	.	.	.	.	.	.	.	.	1	.	.	.	.	.	.
Hacquetia epipactis	3	.	.	.	.	.	1	.	1	.	.	.	.	.	.	1	.
Epilobium obscurum	3	.	1	.	.	.	.	1	.	.	.	.	.	.	.	.	.
Sanguisorba verrucosa	3	.	.	.	.	.	.	.	1	.	.	.	.	.	.	.	.
Dianthus tenuifolius	3	.	.	.	.	.	.	.	.	.	.	.	.	1	.	.	2
Astragalus glycyphylloides	3	.	.	.	.	.	.	.	.	.	1	.	.	.	.	.	.
Hesperis sylvestris	3	.	.	.	.	.	.	.	.	.	1	.	.	.	.	.	.
Comarum palustre	3	.	1	.	1	.	.	.	1	.	.	.	.	.	.	.	.
Senecio macrophyllus	3	.	.	1	.	.	.	.	.	.	1	.	.	.	.	.	.
Lupinus polyphyllus	3	.	.	.	.	1	1	.	.	.	1	.	.	.	.	.	.
Potentilla caulescens	3	.	.	.	.	.	.	.	.	.	1	.	.	.	.	.	.
Gypsophila perfoliata	3	.	.	.	.	.	.	.	.	.	.	1	.	.	.	.	.
Amaranthus graecizans	3	.	.	.	1	.	1	.	.	.	.	.	.	.	.	.	.
Vicia serratifolia	3	.	.	.	.	.	.	.	.	.	1	.	.	.	.	.	.
Bupleurum rotundifolium	3	.	.	.	.	.	.	.	.	1	1	.	.	.	.	.	.
Phegopteris connectilis	3	.	.	.	.	.	.	.	.	.	.	.	.	.	.	.	3
Cardamine impatiens	3	.	.	.	.	.	1	.	.	.	.	.	.	.	.	.	.
Typha latifolia	3	.	1	.	1	.	.	.	.	.	.	.	.	.	.	.	.
Astragalus pallescens	3	.	.	.	.	.	.	.	.	.	1	.	.	.	.	.	.
Anthriscus cerefolium	3	.	.	.	.	.	.	.	.	.	1	.	.	.	.	.	.
Jurinea cyanoides	3	.	.	.	.	.	.	.	.	.	1	.	.	.	.	.	.
Thesium ebracteatum	3	.	.	.	.	.	.	.	.	.	1	1	.	.	.	.	.
Glaux maritima	3	.	.	1	1	.	.	.	.	.	.	.	.	.	.	.	.
Lepidium densiflorum	3	.	.	.	.	.	1	.	.	.	.	1	1	.	.	.	.
Melica altissima	3	.	.	.	.	.	.	.	.	.	1	.	.	.	.	.	.
Ranunculus lingua	3	.	.	.	1	.	1	.	.	.	.	.	.	.	.	.	.
Chenopodium polyspermum	3	.	.	.	1	1	1	.	.	.	.	.	.	.	.	.	.
Melilotus altissimus	3	1	.	.	.	.	1	.	1	.	.	.	.	.	.	.	.
Pleurospermum austriacum	3	.	.	.	.	.	.	.	.	.	.	.	.	.	.	1	.
Petasites spurius	3	.	.	1	1	.	.	.	.	.	.	1	.	.	.	.	.
Androsace chamaejasme	3	.	.	.	.	.	.	.	.	.	.	.	.	.	.	1	.
Thesium pyrenaicum	3	.	.	.	.	.	1	.	1	1	.	.	.	.	.	.	.
Rorippa islandica	3	.	.	.	1	.	1	.	.	.	.	.	.	.	.	.	.
Sisyrinchium septentrionale	3	.	.	.	.	.	1	.	.	1	.	.	.	.	.	.	.
Ferula heuffelii	3	.	.	.	.	.	.	.	.	.	1	.	.	.	.	.	1
Arabis ciliata	3	.	.	.	.	.	.	.	1	1	.	.	.	.	.	.	.
Melica picta	3	.	.	.	.	.	.	.	.	1	1	.	.	1	.	.	.
Isatis praecox	3	.	.	.	.	.	.	.	.	.	1	.	.	1	.	.	.
Erysimum carniolicum	3	.	.	.	.	.	.	.	.	1	.	.	.	1	.	.	.
Serratula coronata	3	.	.	.	.	.	1	.	.	.	1	.	.	.	.	.	.
Taeniatherum caput-medusae	3	.	.	.	.	.	.	.	.	.	1	.	.	.	.	.	.
Circaea lutetiana	3	.	1	.	.	.	1	.	.	.	.	.	.	.	.	.	.
Melissa officinalis	3	.	.	.	.	.	.	.	1	.	1	.	.	.	.	.	.
Parietaria officinalis	3	.	.	.	.	.	1	1	.	.	.	.	.	.	.	.	.
Centaurea kotschyana	3	.	.	.	.	.	1	.	.	.	.	.	.	.	.	.	.
Tordylium maximum	3	.	.	.	.	.	.	1	.	.	1	.	.	.	.	.	.
Polygonum minus	3	.	1	.	1	.	.	.	.	.	.	.	.	.	.	.	.
Fritillaria ruthenica	3	.	.	.	1	.	.	.	.	.	1	.	.	.	.	.	.
Arctostaphylos uva-ursi	3	.	.	.	.	.	.	.	.	.	.	.	.	.	.	1	.
Pedicularis verticillata	3	.	.	.	.	.	1	.	.	.	.	.	.	.	.	1	.
Thymus bihoriensis	3	.	.	.	.	.	.	.	.	.	1	.	.	.	.	.	.
Phyteuma vagneri	3	.	.	.	.	1	.	.	.	.	.	.	.	.	.	.	.
Cytisus leiocarpus	3	.	.	.	.	.	.	.	.	1	1	.	.	1	.	.	.
Polygonatum hirtum	3	.	.	.	.	.	1	.	.	.	.	.	.	.	.	1	.
Calamagrostis villosa	3	.	.	.	.	1	1	.	.	.	.	.	.	.	.	.	.

Cluster number		1	2	3	4	5	6	7	8	9	10	11	12	13	14	15	16
No. of relevés (all)		257	260	213	797	1411	4434	526	1306	1779	5089	201	203	761	429	219	108
- (with NVP recorded)		133	133	51	234	471	1227	173	409	559	1421	46	121	145	59	100	12
Atriplex oblongifolia	3	.	.	.	.	.	.	.	.	.	1	.	.	.	.	.	.
Hieracium saxatile	3	.	.	.	.	.	1	.	.	.	1	.	.	.	1	.	.
Galinsoga quadriradiata	3	.	.	.	1	.	1	.	.	.	.	.	.	.	.	.	.
Calepina irregularis	3	.	.	.	.	.	1	.	.	.	1	.	.	.	.	.	.
Taraxacum sect. Obliqua	3	.	.	.	.	.	.	.	.	.	1	.	.	.	.	.	.
Geum montanum	3	.	.	.	.	1	1	.	.	.	.	.	.	.	.	.	.
Silene noctiflora	3	.	.	.	.	.	.	.	.	.	1	.	.	.	.	.	.
Melampyrum bihariense	3	.	.	.	.	.	.	.	1	.	.	.	.	1	.	.	.
Orchis pallens	3	.	.	.	.	.	.	.	.	1	.	.	.	.	1	.	.
Vicia lutea	3	.	.	.	.	.	.	1	.	1	1	.	.	.	.	.	.
Smyrnium perfoliatum	3	.	.	.	.	.	.	1	.	1	1	.	.	.	.	.	.
Carex brachystachys	3	.	.	.	.	.	1	.	.	.	.	.	.	.	.	1	.
Althaea hirsuta	2	.	.	.	.	.	.	.	.	1	1	.	.	.	.	.	.
Sclerochloa dura	2	.	.	.	.	.	.	.	.	.	1	.	.	.	.	.	.
Plantago schwarzenbergiana	2	.	.	.	1	.	.	.	.	.	1	.	.	.	.	.	.
Drosera rotundifolia	2	1	.	.	.	1	.	.	.	.	.	.	.	.	.	.	.
Bifora radians	2	.	.	.	.	.	1	.	.	.	1	.	.	.	.	.	.
Leucanthemum platylepis	2	.	.	.	.	.	.	.	1	1	.	.	.	.	.	.	.
Androsace septentrionalis	2	.	.	.	.	.	.	.	.	.	1	.	.	.	.	.	.
Ranunculus arvensis	2	.	.	.	.	.	1	1	.	.	.	.	.	.	.	.	.
Alyssum linifolium	2	.	.	.	.	.	.	.	.	.	1	.	.	.	.	.	.
Narcissus pseudonarcissus	2	.	.	.	.	.	.	.	.	.	1	.	.	.	.	.	.
Diphasiastrum complanatum	2	.	.	.	.	1	.	.	.	.	.	.	.	.	.	.	.
Taraxacum sect. Dioszegia	2	.	.	.	.	.	.	.	.	.	1	.	.	.	.	.	.
Dipsacus pilosus	2	.	.	.	.	.	.	.	.	1	1	.	.	.	.	.	.
Scilla bifolia	2	.	.	.	.	.	1	.	.	.	.	.	.	.	.	.	.
Veronica opaca	2	.	.	.	1	.	1	.	.	.	.	.	.	.	.	.	.
Geranium macrorrhizum	2	.	.	.	.	.	.	.	.	.	.	.	.	.	.	.	2
Thymus roegneri	2	.	.	.	.	.	1	.	.	.	.	.	.	.	.	.	.
Sedum borissovae	2	.	.	.	.	.	.	.	.	.	1	.	.	.	.	.	.
Linum bienne	2	.	.	.	1	.	.	1	.	.	.	.	.	.	.	.	.
Notholaena marantae	2	.	.	.	.	.	.	.	.	.	1	.	.	1	.	.	.
Atriplex calotheca	2	.	.	.	1	.	.	.	.	.	.	.	.	.	.	.	.
Galium kitaibelianum	2	.	.	.	.	.	.	.	.	.	.	.	.	.	.	.	2
Avena fatua	2	.	.	.	.	.	1	.	.	.	1	.	.	.	.	.	.
Tephroseris czernijevii	2	.	.	.	.	.	.	.	.	.	1	.	.	.	.	.	.
Angelica archangelica	2	.	.	.	.	1	.	.	.	.	1	.	.	.	.	.	.
Phedimus spurius	2	.	.	.	.	.	.	.	.	.	1	.	.	1	.	.	.
Atriplex prostrata	2	.	.	.	.	.	.	.	.	.	1	.	.	.	.	.	.
Veronica filiformis	2	.	.	.	1	.	1	.	.	.	.	.	.	.	.	.	.
Ruscus aculeatus L.	2	.	.	.	.	.	.	.	.	.	1	.	.	.	.	.	.
Galium verticillatum	2	.	.	1	1	.	.	.	.	.	.	.	.	.	.	.	.
Knautia x posoniensis	2	.	.	.	.	.	.	1	.	1	.	.	.	.	.	.	.
Alcea rugosa	2	.	.	.	.	.	.	.	.	.	1	.	.	.	.	.	.
Sisymbrium officinale	2	.	.	.	.	.	1	.	.	.	1	.	.	.	.	.	.
Echinocystis lobata	2	.	.	.	1	.	1	.	.	.	.	.	.	.	.	.	.
Stellaria neglecta	2	.	.	.	1	.	1	.	.	.	.	.	.	.	.	.	.
Gymnadenia borealis	2	.	1	.	.	.	.	.	1	.	.	.	.	.	.	.	.
Bidens cernuus	2	.	.	.	1	.	1	.	.	.	.	.	.	.	.	.	.
Ligularia carpathica	2	.	.	.	.	.	.	.	.	.	.	.	.	.	.	.	2
Selaginella selaginoides	2	.	.	.	.	.	.	.	.	.	.	.	.	.	.	1	.
Hieracium transylvanicum	2	.	.	.	.	.	.	.	.	.	.	.	.	1	.	.	1
Dryas octopetala	2	.	.	.	.	.	.	.	.	.	.	.	.	.	.	1	.
Poa molinerii	2	.	.	.	.	.	.	.	.	.	.	.	.	.	.	1	.
Hieracium glaucinum	2	.	.	.	.	.	.	.	.	.	1	.	.	1	.	.	.
Agropyron dasyanthum	2	.	.	.	.	.	.	.	.	.	1	1	.	.	.	.	.
Delphinium elatum	2	.	.	.	.	.	.	.	.	.	.	.	.	.	.	1	.
Eleocharis quinqueflora	2	1	1	.	.	.	.	.	.	.	.	.	.	.	.	.	.
Erechtites hieraciifolius	2	.	.	.	.	.	1	.	.	.	1	.	.	.	.	.	.
Carex fritschii	2	.	.	.	.	.	1	.	.	.	1	.	.	.	.	.	.
Cimicifuga europaea	2	.	.	.	.	.	.	.	.	1	.	.	.	.	.	1	.
Festuca versicolor	2	.	.	.	.	.	.	.	.	.	.	.	.	.	.	1	.
Actaea spicata	2	.	.	.	.	.	1	.	.	.	.	.	.	.	.	1	.
Crepis alpestris	2	.	.	.	.	.	.	.	.	1	.	.	.	.	.	1	.
Centaurea paniculata	2	.	.	.	.	.	.	.	.	.	1	.	.	.	.	.	.
Helianthus tuberosus	2	.	.	.	.	.	1	.	.	.	.	.	.	.	.	.	.
Glaucium corniculatum	2	.	.	.	.	.	1	.	.	.	1	.	.	.	.	.	.
Schoenus × scheuchzeri	2	1	.	.	.	.	1	.	.	.	.	.	.	.	.	.	.
Galium tenuissimum	2	.	.	.	.	.	.	.	.	.	1	.	.	.	.	.	.
Iris ruthenica	2	.	.	.	.	.	.	.	.	1	.	.	.	1	.	.	.
Pyrola rotundifolia	2	.	.	.	.	.	1	.	.	.	.	.	.	.	.	.	.
Juncus tenageia	2	.	.	.	.	1	.	.	.	.	.	.	.	.	.	.	.
Artemisia pancicii	2	.	.	.	.	.	.	.	.	.	1	.	.	.	.	.	.
Carex brevicollis	2	.	.	.	.	.	.	.	.	.	1	.	.	.	.	.	1
Silene gallica	2	.	.	.	.	.	.	.	.	.	1	.	.	.	.	.	.
Stellaria alsine	2	.	1	.	1	.	.	.	.	.	.	.	.	.	.	.	.
Scutellaria altissima	2	.	.	.	.	.	.	.	.	.	1	.	.	.	.	.	1
Echinophora sibthorpiana	2	.	.	.	.	.	.	.	.	1	1	.	.	.	.	.	.
Geranium divaricatum	2	.	.	.	.	.	.	.	.	.	1	.	.	.	.	.	.
Plantago maxima	2	.	.	.	.	.	.	.	.	.	1	.	.	.	.	.	.
Carex diandra	2	.	1	.	.	.	1	.	.	.	.	.	.	.	.	.	.

Cluster number		1	2	3	4	5	6	7	8	9	10	11	12	13	14	15	16
No. of relevés (all)		257	260	213	797	1411	4434	526	1306	1779	5089	201	203	761	429	219	108
- (with NVP recorded)		133	133	51	234	471	1227	173	409	559	1421	46	121	145	59	100	12
Lysimachia thyrsiflora	2	.	1	.	.	.	.	.	.	.	.	.	.	.	.	.	.
Laphangium luteoalbum	2	.	.	.	.	.	1	.	1	.	.	.	.	.	.	.	.
Dianthus leptopetalus	2	.	.	.	.	.	.	.	.	.	1	.	.	.	.	.	.
Potentilla supina	2	.	.	.	1	.	.	.	.	.	1	.	.	.	.	.	.
Dasypyrum villosum	2	.	.	.	.	.	.	.	.	.	1	.	.	.	.	.	.
Hieracium maculatum	2	.	.	.	.	.	1	.	.	.	.	.	.	1	.	.	.
Aconitum napellus	2	.	.	.	.	.	1	.	.	.	.	.	.	.	.	.	.
Carex pallidula	2	.	.	.	.	1	1	.	.	.	.	.	.	.	.	.	.
Hieracium pilosum	2	.	.	.	.	.	.	.	.	.	1	.	.	.	.	.	.
Asphodelus albus	2	.	.	.	.	.	.	.	.	.	.	.	.	1	.	.	.
Trichophorum alpinum	2	1	.	.	.	.	.	.	1	.	.	.	.	.	.	.	.
Silene pusilla	2	.	.	.	.	.	1	.	1	.	.	.	.	.	.	.	.
Corydalis cava	2	.	.	.	.	.	.	.	.	.	1	.	.	.	.	.	.
Hieracium piliferum	2	.	.	.	1	.	.	.	.	.	.	.	.	.	.	1	.
Menyanthes trifoliata	2	1	.	.	1	.	.	.	.	.	.	.	.	.	.	.	.
Potentilla sterilis	2	.	.	.	.	.	1	.	.	.	.	.	.	.	.	.	.
Trichophorum pumilum	2	1	.	.	.	.	.	.	.	.	.	.	.	.	.	.	.
Carex hordeistichos	2	.	.	.	.	.	1	.	.	.	.	.	.	.	.	.	.
Chrysanthemum zawadskii	2	.	.	.	.	.	.	.	.	.	.	.	.	1	.	1	.
Kickxia spuria	2	.	.	.	.	.	.	.	.	1	1	.	.	.	.	.	.
Helictochloa hookeri ssp. schelliana	2	.	.	.	.	.	.	.	.	1	1	.	.	.	.	.	.
Vinca minor	2	.	.	.	.	.	1	.	.	.	1	.	.	.	.	.	.
Veronica montana	2	.	.	.	.	.	1	.	.	.	.	.	.	.	.	.	.
Achillea virescens	2	.	.	.	.	.	1	.	.	.	.	.	.	.	.	.	.
Calamagrostis pseudophragmites	2	.	.	.	1	.	.	.	.	.	1	.	.	.	.	.	.
Chenopodium ficifolium	2	.	.	.	1	.	.	.	.	.	1	.	.	.	.	.	.
Sedum annuum	2	.	.	.	.	.	.	.	.	.	1	.	.	.	.	.	.
Gagea transversalis	2	.	.	.	.	.	.	.	.	.	1	.	.	.	.	.	.
Veronica anagallis-aquatica	2	.	.	.	1	.	.	.	.	.	.	.	.	.	.	.	.
Jurinea ewersmanii	2	.	.	.	.	.	.	.	.	.	.	1	.	.	.	.	.
Tanacetum macrophyllum	2	.	.	.	.	.	.	1	.	.	1	.	.	.	.	.	.
Bromus rigidus	2	.	.	.	.	.	.	.	.	.	1	.	.	.	.	.	.
Thelypteris palustris	2	.	1	.	1	.	.	.	.	.	.	.	.	.	.	.	.
Cuscuta epilinum	2	.	.	.	.	.	1	.	.	.	.	.	.	1	.	.	.
Aquilegia einseleana	2	.	.	.	.	.	1	.	1	.	.	.	.	.	.	.	.
Euphorbia exigua	2	.	.	.	.	.	.	1	.	.	.	.	.	.	.	.	.
Carex pendula	2	.	1	.	.	.	.	.	.	.	.	.	.	.	.	.	.
Arabidopsis petrogena	2	.	.	.	.	.	.	.	.	.	1	.	.	1	.	.	.
Ornithogalum nutans	2	.	.	.	.	.	1	.	.	.	1	.	.	.	.	.	.
Sonchus palustris	2	1	1	.	.	.	.	.	.	.	.	.	.	.	.	.	.
Crepis sancta	2	.	.	.	.	.	.	.	.	.	1	.	.	.	.	.	.
Taraxacum sect. Alpestria	2	.	.	.	.	1	1	.	.	.	.	.	.	.	.	.	.
Alcea biennis	2	.	.	.	.	.	.	.	.	.	1	.	.	.	.	.	.
Agropyron tanaiticum	2	.	.	.	.	.	.	.	.	.	1	.	.	.	.	.	.
Ranunculus sceleratus	2	.	.	.	1	.	.	.	.	.	.	.	.	.	.	.	.
Euphorbia dulcis	2	.	.	.	.	1	1	.	.	.	.	.	.	.	.	.	.
Eryngium maritimum	2	.	.	.	.	.	.	.	.	.	.	1	.	.	.	.	.
Noccaea goesingensis	2	.	.	.	.	.	1	1	.	.	.	.	.	.	.	.	.
Woodsia ilvensis	2	.	.	.	.	.	.	.	.	.	1	.	.	1	.	.	.
Acanthus hungaricus	2	.	.	.	.	.	.	.	.	.	1	.	.	.	.	.	.
Silene dichotoma	2	.	.	.	.	.	.	.	.	.	1	.	.	.	.	.	.
Astracantha arnacantha	2	.	.	.	.	.	.	.	.	.	1	.	.	.	.	.	.
Dracocephalum ruyschiana	2	.	.	.	.	.	.	.	.	.	1	.	.	.	.	.	.
Veronica acinifolia	2	.	.	.	.	.	.	1	.	.	.	.	.	.	.	.	.
Artemisia hololeuca	2	.	.	.	.	.	.	.	.	.	1	.	.	.	.	.	.
Peucedanum altissimum	2	.	.	.	.	.	.	.	.	1	.	.	.	.	.	.	.
Geum aleppicum	2	.	.	.	1	.	1	.	.	.	.	.	.	.	.	.	.
Impatiens glandulifera	2	.	.	.	1	.	1	.	.	.	.	.	.	.	.	.	.
Cynanchum acutum	2	.	.	.	.	.	.	.	.	.	1	.	.	.	.	.	.
Lupinus luteus	2	.	.	.	.	.	.	.	.	.	.	.	1	.	.	.	.
Thymus moldavicus	2	.	.	.	.	.	.	.	.	.	1	.	.	.	.	.	.
Prangos ferulacea	2	.	.	.	.	.	.	.	.	.	1	.	.	.	.	.	.
Hyparrhenia hirta	2	.	.	.	.	.	.	.	1	.	1	.	.	.	.	.	.
Linaria dulcis	2	.	.	.	.	.	.	.	.	.	1	1	.	.	.	.	.
Cynoglossum germanicum	2	.	.	.	.	.	.	.	.	.	1	.	.	.	.	.	.
Vicia pisiformis	2	.	.	.	.	.	.	.	.	1	1	.	.	.	.	.	.
Gagea pratensis	2	.	.	.	1	.	.	.	.	.	1	.	.	.	.	.	.
Turgenia latifolia	2	.	.	.	.	.	.	.	.	.	1	.	.	.	.	.	.
Potentilla micrantha	2	.	.	.	.	.	.	.	.	1	.	.	.	1	.	.	.
Ranunculus thora	1	.	.	.	.	.	.	.	1	.	.	.	.	.	.	.	.
Colchicum versicolor	1	.	.	.	.	.	.	.	.	.	1	.	.	.	.	.	.
Mentha asiatica	1	.	.	.	1	.	.	.	.	.	.	.	.	.	.	.	.
Cuscuta approximata	1	.	.	.	.	.	.	.	.	.	1	.	.	.	.	.	.
Anthemis cotula	1	.	.	.	.	1	.	.	.	.	.	.	.	.	.	.	.
Phyteuma ovatum	1	.	.	.	.	.	1	.	.	.	.	.	.	.	.	.	.
Cytisus borysthenicus	1	.	.	.	.	.	.	.	.	.	1	.	.	.	.	.	.
Colchicum hungaricum	1	.	.	.	.	.	.	.	.	.	1	.	.	.	.	.	.
Elytrigia bessarabica	1	.	.	.	.	.	.	.	.	.	1	.	.	.	.	.	.
Ranunculus carinthiacus	1	.	.	.	.	.	1	.	.	.	.	.	.	.	.	.	.
Echinops exaltatus	1	.	.	.	.	.	.	.	.	.	1	.	.	.	.	.	.
Epilobium adenocaulon	1	.	.	.	.	.	1	.	.	.	.	.	.	.	.	.	.

Cluster number		1	2	3	4	5	6	7	8	9	10	11	12	13	14	15	16
No. of relevés (all)		257	260	213	797	1411	4434	526	1306	1779	5089	201	203	761	429	219	108
- (with NVP recorded)		133	133	51	234	471	1227	173	409	559	1421	46	121	145	59	100	12
Antennaria carpatica	1	.	.	.	.	.	.	.	1	.	.	.	.	.	.	.	.
Cerinthe glabra	1	.	.	.	.	.	.	.	.	1	.	.	.	.	.	.	.
Crypsis aculeata	1	.	.	.	.	.	.	.	.	.	1	.	.	.	.	.	.
Cladium mariscus	1	.	.	.	.	.	1	.	.	.	.	.	.	.	.	.	.
Clinopodium suaveolens	1	.	.	.	.	.	.	.	.	.	1	.	.	.	.	.	.
Euphrasia minima ssp. tatrae	1	.	.	.	.	1	.	.	.	.	.	.	.	.	.	.	.
Camelina rumelica	1	.	.	.	.	.	.	.	.	.	1	.	.	.	.	.	.
Beta vulgaris	1	.	.	.	.	.	1	.	.	.	.	.	.	.	.	.	.
Galanthus nivalis	1	.	.	.	.	.	.	.	.	.	1	.	.	.	.	.	.
Campanula cespitosa	1	.	.	.	.	.	.	.	.	1	.	.	.	.	.	.	.
Panicum capillare	1	.	.	.	.	.	.	.	.	.	1	.	.	.	.	.	.
Epimedium alpinum	1	.	.	.	.	.	.	.	1	.	.	.	.	.	.	.	.
Campanula kladniana	1	.	.	.	.	1	.	.	.	.	.	.	.	.	.	.	.
Dactylorhiza lapponica	1	.	.	.	.	.	1	.	.	.	.	.	.	.	.	.	.
Dianthus ferrugineus ssp. liburnicus	1	.	.	.	.	.	1	.	.	.	.	.	.	.	.	.	.
Dianthus pseudoserotinus	1	.	.	.	.	.	.	.	.	.	1	.	.	.	.	.	.
Cytisus paczoskii	1	.	.	.	.	.	.	.	.	.	1	.	.	.	.	.	.
Anthriscus nitidus	1	.	.	.	.	.	.	.	.	.	1	.	.	.	.	.	.
Bromus cappadocica	1	.	.	.	.	.	.	.	.	.	1	.	.	.	.	.	.
Althaea cannabina	1	.	.	.	.	.	.	.	.	.	1	.	.	.	.	.	.
Anethum graveolens	1	.	.	.	.	.	1	.	.	.	.	.	.	.	.	.	.
Valeriana tuberosa	1	.	.	.	.	.	.	.	.	.	1	.	.	.	.	.	.
Anemone trifolia	1	.	.	.	.	.	.	.	.	1	.	.	.	.	.	.	.
Carex pseudocyperus	1	.	.	.	1	.	.	.	.	.	.	.	.	.	.	.	.
Butomus umbellatus	1	.	.	.	1	.	.	.	.	.	.	.	.	.	.	.	.
Eleocharis acicularis	1	.	.	.	1	.	.	.	.	.	.	.	.	.	.	.	.
Plantago atrata	1	.	.	.	.	.	1	.	.	.	.	.	.	.	.	.	.
Sparganium emersum	1	.	1	.	.	.	.	.	.	.	.	.	.	.	.	.	.
Ranunculus trichophyllus	1	.	.	.	1	.	.	.	.	.	.	.	.	.	.	.	.
Veronica capsellicarpa	1	.	.	.	.	.	.	.	.	.	1	.	.	.	.	.	.
Mentha piperita	1	.	.	.	.	.	1	.	.	.	.	.	.	.	.	.	.
Agrostemma githago	1	.	.	.	.	.	.	.	.	.	1	.	.	.	.	.	.
Tephroseris palustris	1	.	.	.	1	.	.	.	.	.	.	.	.	.	.	.	.
Helosciadium repens	1	.	1	.	.	.	.	.	.	.	.	.	.	.	.	.	.
Leucojum vernum	1	.	.	.	.	.	1	.	.	.	.	.	.	.	.	.	.
Lythrum tribracteatum	1	.	.	.	1	.	.	.	.	.	.	.	.	.	.	.	.
Eleocharis ovata	1	.	1	.	.	.	.	.	.	.	.	.	.	.	.	.	.
Allium strictum	1	.	.	.	.	.	.	.	.	.	1	.	.	.	.	.	.
Lens nigricans	1	.	.	.	.	.	.	.	.	1	.	.	.	.	.	.	.
Fritillaria meleagroides	1	.	.	.	1	.	.	.	.	.	.	.	.	.	.	.	.
Sagina apetala	1	.	.	.	1	.	.	.	.	.	.	.	.	.	.	.	.
Urtica urens	1	.	.	.	.	.	1	.	.	.	.	.	.	.	.	.	.
Tragopogon dasyrhynchus	1	.	.	.	.	.	.	.	.	.	1	.	.	.	.	.	.
Setaria verticillata	1	.	.	.	.	.	.	.	.	.	1	.	.	.	.	.	.
Corispermum canescens	1	.	.	.	.	.	.	.	.	.	.	.	1	.	.	.	.
Trachystemon orientalis	1	.	.	.	.	.	.	.	.	.	.	.	1	.	.	.	.
Lappula heteracantha	1	.	.	.	.	.	.	.	.	.	1	.	.	.	.	.	.
Melampyrum alboffianum	1	.	.	.	.	.	.	.	.	.	1	.	.	.	.	.	.
Astragalus virgeus	1	.	.	.	.	.	.	.	.	.	.	.	1	.	.	.	.
Ranunculus lateriflorus	1	.	.	.	1	.	.	.	.	.	.	.	.	.	.	.	.
Ceratocephala orthoceras	1	.	.	.	.	.	.	.	.	.	1	.	.	.	.	.	.
Lolium temulentum	1	.	.	.	.	.	1	.	.	.	.	.	.	.	.	.	.
Veronica paniculata	1	.	.	.	.	.	.	.	.	.	1	.	.	.	.	.	.
Erysimum leucanthemum	1	.	.	.	1	.	.	.	.	.	.	.	.	.	.	.	.
Cardamine flexuosa	1	.	.	.	1	.	.	.	.	.	.	.	.	.	.	.	.
Anchusa ochroleuca	1	.	.	.	.	.	.	.	.	.	1	.	.	.	.	.	.
Salvia scabiosifolia	1	.	.	.	.	.	.	.	.	1	.	.	.	.	.	.	.
Glyceria pedicellata	1	.	.	.	1	.	.	.	.	.	.	.	.	.	.	.	.
Artemisia maritima	1	.	.	.	1	.	.	.	.	.	.	.	.	.	.	.	.
Lunaria rediviva	1	.	.	.	.	.	.	.	1	.	.	.	.	.	.	.	.
Callitriche palustris agg.	1	.	.	.	.	.	1	.	.	.	.	.	.	.	.	.	.
Hirschfeldia incana	1	.	.	.	.	.	.	.	.	1	.	.	.	.	.	.	.
Galium flavescens	1	.	.	.	.	.	.	.	.	.	1	.	.	.	.	.	.
Himantoglossum caprinum	1	.	.	.	.	.	.	.	.	1	.	.	.	.	.	.	.
Potentilla argaea	1	.	.	1	.	.	.	.	.	.	.	.	.	.	.	.	.
Arenaria cephalotes	1	.	.	.	.	.	.	.	.	.	1	.	.	.	.	.	.
Sedum atratum	1	.	.	.	.	1	.	.	.	.	.	.	.	.	.	.	.
Alcea rosea	1	.	.	.	.	.	.	.	.	.	1	.	.	.	.	.	.
Asperugo procumbens	1	.	.	.	.	.	.	.	.	.	1	.	.	.	.	.	.
Cirsium boujartii	1	.	.	.	.	.	.	.	.	.	1	.	.	.	.	.	.
Lathyrus laevigatus	1	.	.	.	.	.	1	.	.	.	.	.	.	.	.	.	.
Gentianella aspera	1	.	.	.	.	1	.	.	.	.	.	.	.	.	.	.	.
Aquilegia atrata	1	.	.	.	.	.	.	.	1	.	.	.	.	.	.	.	.
Hypericum richeri ssp. grisebachii	1	.	.	.	.	.	.	.	.	1	.	.	.	.	.	.	.
Senecio doronicum	1	.	.	.	.	.	1	.	.	.	.	.	.	.	.	.	.
Euphorbia nutans	1	.	.	.	.	.	.	.	.	.	1	.	.	.	.	.	.
Anthriscus caucalis	1	.	.	.	.	.	.	.	.	.	1	.	.	.	.	.	.
Herminium monorchis	1	.	.	.	.	.	1	.	.	.	.	.	.	.	.	.	.
Carex obtusata	1	.	.	.	.	.	.	.	.	.	1	.	.	.	.	.	.
Anagallis minima	1	.	.	.	.	.	.	1	.	.	.	.	.	.	.	.	.
Iris reichenbachii	1	.	.	.	.	.	.	.	.	.	1	.	.	.	.	.	.

Cluster number		1	2	3	4	5	6	7	8	9	10	11	12	13	14	15	16
No. of relevés (all)		257	260	213	797	1411	4434	526	1306	1779	5089	201	203	761	429	219	108
- (with NVP recorded)		133	133	51	234	471	1227	173	409	559	1421	46	121	145	59	100	12
Chenopodium hybridum	1	.	.	.	.	.	1	.	.	.	.	.	.	.	.	.	.
Campanula lingulata	1	.	.	.	.	.	.	.	.	.	1	.	.	.	.	.	.
Astragalus pubiflorus	1	.	.	.	.	.	.	.	.	.	1	.	.	.	.	.	.
Hieracium porrifolium	1	.	.	.	.	.	.	.	.	1	.	.	.	.	.	.	.
Oenothera villosa	1	.	.	.	1	.	.	.	.	.	.	.	.	.	.	.	.
Glyceria declinata	1	.	1	.	.	.	.	.	.	.	.	.	.	.	.	.	.
Alyssum smyrnaeum	1	.	.	.	.	.	.	.	.	.	1	.	.	.	.	.	.
Knautia purpurea	1	.	.	.	.	.	1	.	.	.	.	.	.	.	.	.	.
Amaranthus hybridus	1	.	.	.	.	.	.	.	.	.	1	.	.	.	.	.	.
Calamagrostis neglecta	1	.	.	.	1	.	.	.	.	.	.	.	.	.	.	.	.
Aquilegia transsilvanica	1	.	.	.	.	.	.	.	.	.	1	.	.	.	.	.	.
Pedicularis elongata ssp. julica	1	.	.	.	.	.	.	.	1	.	.	.	.	.	.	.	.
Clinopodium nepeta	1	.	.	.	.	.	1	.	.	.	.	.	.	.	.	.	.
Comandra umbellata	1	.	.	.	.	.	.	.	.	.	1	.	.	.	.	.	.
Cirsium heterophyllum	1	.	.	.	.	.	1	.	.	.	.	.	.	.	.	.	.
Crepis neglecta	1	.	.	.	.	.	1	.	.	.	.	.	.	.	.	.	.
Milium vernale	1	.	.	.	.	.	.	.	.	.	1	.	.	.	.	.	.
Brassica napus	1	.	.	.	.	.	1	.	.	.	.	.	.	.	.	.	.
Hieracium pseudobifidum ssp. trebevicianum	1	.	.	.	.	.	.	.	.	.	.	.	.	.	.	.	1
Luzula congesta	1	.	.	.	.	1	.	.	.	.	.	.	.	.	.	.	.
Iberis pinnata	1	.	.	.	.	.	.	.	.	.	1	.	.	.	.	.	.
Avena sativa	1	.	.	.	.	.	.	1	.	.	.	.	.	.	.	.	.
Pilosella rothiana	1	.	.	.	.	.	.	.	.	.	1	.	.	.	.	.	.
Vulpia bromoides	1	.	.	.	.	.	1	.	.	.	.	.	.	.	.	.	.
Myosotis alpestris	1	.	.	.	.	.	.	.	.	.	.	.	.	.	.	.	1
Stipa anomala	1	.	.	.	.	.	.	.	.	.	1	.	.	.	.	.	.
Asplenium scolopendrium	1	.	.	.	.	.	.	.	.	.	.	.	.	.	.	.	1
Lycopodium annotinum	1	.	.	.	.	1	.	.	.	.	.	.	.	.	.	.	.
Asperula graveolens	1	.	.	.	.	.	.	.	.	.	.	1	.	.	.	.	.
Eragrostis cilianensis	1	.	.	.	.	.	.	.	.	.	.	1	.	.	.	.	.
Edraianthus graminifolius	1	.	.	.	.	.	.	.	.	.	.	.	.	1	.	.	.
Hieracium hypochoeroides ssp. wiesbaurianum	1	.	.	.	.	.	.	.	.	.	.	.	.	1	.	.	.
Spergularia marina	1	.	.	.	.	.	.	.	.	.	1	.	.	.	.	.	.
Lemna trisulca	1	.	.	.	.	.	1	.	.	.	.	.	.	.	.	.	.
Orobanche maxima	1	.	.	.	.	.	.	.	.	.	.	1	.	.	.	.	.
Veronica aphylla	1	.	.	.	.	.	.	.	.	.	.	.	.	.	.	1	.
Atriplex rosea	1	.	.	1	.	.	.	.	.	.	.	.	.	.	.	.	.
Hieracium schmidtii	1	.	.	.	.	.	.	.	.	.	.	.	.	.	.	1	.
Campanula alpina	1	.	.	.	.	.	.	.	.	.	.	.	.	.	.	1	.
Stipa zalesskii	1	.	.	.	.	.	.	.	.	.	1	.	.	.	.	.	.
Valerianella costata	1	.	.	.	.	.	.	.	.	.	1	.	.	.	.	.	.
Hippuris vulgaris	1	.	.	.	.	.	.	.	.	1	.	.	.	.	.	.	.
Telekia speciosa	1	.	.	.	.	.	1	.	.	.	.	.	.	.	.	.	.
Potentilla anglica	1	1	.	.	.	.	.	.	.	.	.	.	.	.	.	.	.
Viola epipsila	1	.	.	.	.	1	.	.	.	.	.	.	.	.	.	.	.
Petasites kablikianus	1	.	.	.	.	.	.	.	.	.	.	.	.	.	.	1	.
Potentilla crantzii	1	.	.	.	.	.	.	.	.	.	.	.	.	.	.	1	.
Arabis soyeri	1	.	.	.	.	.	.	.	.	.	.	.	.	.	.	1	.
Sesleria coerulans	1	.	.	.	.	.	.	.	.	.	.	.	.	.	.	1	.
Trifolium retusum	1	.	.	.	.	.	.	.	.	.	1	.	.	.	.	.	.
Helianthus pauciflorus	1	.	.	.	.	.	.	.	.	.	.	.	.	.	.	1	.
Gentiana pumila	1	.	.	.	.	.	.	.	.	.	.	.	.	.	.	1	.
Berula erecta	1	1	.	.	.	.	.	.	.	.	.	.	.	.	.	.	.
Noccaea macrantha	1	.	.	.	.	.	.	.	.	.	1	.	.	.	.	.	.
Chaerophyllum bulbosum	1	.	.	.	.	.	1	.	.	.	.	.	.	.	.	.	.
Orobanche lanuginosa	1	.	.	.	.	.	.	.	.	.	1	.	.	.	.	.	.
Triticum aestivum	1	.	.	.	.	.	1	.	.	.	.	.	.	.	.	.	.
Cakile maritima	1	.	.	.	.	.	.	.	.	.	.	1	.	.	.	.	.
Dipsacus sativus	1	.	.	.	.	.	.	1	.	.	.	.	.	.	.	.	.
Trifolium purpureum	1	.	.	.	.	.	.	.	.	1	.	.	.	.	.	.	.
Pulsatilla taurica	1	.	.	.	.	.	.	.	.	1	.	.	.	.	.	.	.
Pilosella densiflora	1	.	.	.	.	.	.	.	1	.	.	.	.	.	.	.	.
Koeleria talievii	1	.	.	.	.	.	.	.	.	.	1	.	.	.	.	.	.
Artemisia lerchiana	1	.	.	.	.	.	.	.	.	.	1	.	.	.	.	.	.
Stellaria longifolia	1	.	.	.	.	.	1	.	.	.	.	.	.	.	.	.	.
Juncus squarrosus	1	.	.	.	.	1	.	.	.	.	.	.	.	.	.	.	.
Lactuca tatarica	1	.	.	.	.	.	.	.	.	.	1	.	.	.	.	.	.
Erigeron glabratus	1	.	.	.	.	.	.	.	1	.	.	.	.	.	.	.	.
Stachys arvensis	1	.	.	.	.	.	.	.	.	.	1	.	.	.	.	.	.
Klasea erucifolia	1	.	.	.	.	.	.	.	.	.	1	.	.	.	.	.	.
Tephroseris papposa	1	.	.	.	.	.	.	.	.	.	.	.	.	.	.	.	1
Salvia sclarea	1	.	.	.	.	.	.	.	.	.	1	.	.	.	.	.	.
Legousia speculum-veneris	1	.	.	.	.	.	.	1	.	.	.	.	.	.	.	.	.
Cerastium tomentosum	1	.	.	.	.	.	.	.	.	.	.	.	.	1	.	.	.
Conioselinum tataricum	1	.	.	.	.	.	.	.	.	.	.	.	.	.	.	.	1
Silene heuffelii	1	.	.	.	.	.	.	.	.	.	.	.	.	.	.	.	1
Trifolium alpinum	1	.	.	.	.	.	.	.	.	.	1	.	.	.	.	.	.
Lamium garganicum	1	.	.	.	.	.	.	.	.	.	.	.	.	.	.	.	1
Scrophularia heterophylla ssp. laciniata	1	.	.	.	.	.	.	.	.	.	.	.	.	.	.	.	1

Cluster number		1	2	3	4	5	6	7	8	9	10	11	12	13	14	15	16
No. of relevés (all)		257	260	213	797	1411	4434	526	1306	1779	5089	201	203	761	429	219	108
- (with NVP recorded)		133	133	51	234	471	1227	173	409	559	1421	46	121	145	59	100	12
Centaurea chartolepis	1	.	.	1	.	.	.	.	.	.	.	.	.	.	.	.	.
Iris halophila	1	.	.	.	.	.	.	.	.	.	1	.	.	.	.	.	.
Orobanche minor	1	.	.	.	.	.	.	.	.	.	1	.	.	.	.	.	.
Potamogeton crispus	1	.	.	.	.	1	.	.	.	.	.	.	.	.	.	.	.
Poa rehmannii	1	.	.	.	.	.	.	.	.	.	.	.	.	.	.	.	1
Epilobium lanceolatum	1	.	.	.	.	.	.	.	.	.	1	.	.	.	.	.	.
Silene steppicola	1	.	.	1	.	.	.	.	.	.	.	.	.	.	.	.	.
Physalis alkekengi	1	.	.	.	.	.	.	.	.	.	1	.	.	.	.	.	.
Senecio sarracenicus	1	.	.	.	.	.	1	.	.	.	.	.	.	.	.	.	.
Orobanche cumana	1	.	.	.	.	.	.	.	.	.	1	.	.	.	.	.	.
Matthiola fragrans	1	.	.	.	.	.	.	.	.	.	1	.	.	.	.	.	.
Cephalaria laevigata	1	.	.	.	.	.	.	.	.	.	1	.	.	.	.	.	.
Tragopogon brevirostris	1	.	.	.	.	.	.	.	.	.	.	1	.	.	.	.	.
Stellaria crassifolia	1	.	.	1	.	.	.	.	.	.	.	.	.	.	.	.	.
Bromus scoparius	1	.	.	.	.	.	1	.	.	.	.	.	.	.	.	.	.
Polygonum maritimum	1	.	.	.	.	.	.	.	.	.	.	1	.	.	.	.	.
Misopates orontium	1	.	.	.	.	.	.	.	.	.	1	.	.	.	.	.	.
Arenaria ciliata	1	.	.	.	.	.	.	.	.	.	1	.	.	.	.	.	.
Pisum sativum ssp. elatius	1	.	.	.	.	.	.	.	.	.	1	.	.	.	.	.	.
Hydrocotyle vulgaris	1	1	.	.	.	.	.	.	.	.	.	.	.	.	.	.	.
Tephroseris besseriana	1	.	.	.	.	.	.	.	.	1	.	.	.	.	.	.	.
Dianthus squarrosus	1	.	.	.	.	.	.	.	.	.	.	1	.	.	.	.	.
Crepis froelichiana ssp. dinarica	1	.	.	.	.	.	.	.	.	1	.	.	.	.	.	.	.
Torilis ucrainica	1	.	.	.	.	.	1	.	.	.	.	.	.	.	.	.	.
Agrimonia pilosa	1	.	.	.	.	.	.	.	.	.	1	.	.	.	.	.	.
Carex strigosa	1	.	.	.	.	.	.	.	.	1	.	.	.	.	.	.	.
Dracocephalum thymiflorum	1	.	.	.	.	.	.	.	.	.	1	.	.	.	.	.	.
Coronopus squamatus	1	.	.	.	.	.	.	.	.	.	1	.	.	.	.	.	.
Dianthus speciosus	1	.	.	.	.	.	.	.	.	.	1	.	.	.	.	.	.
Dianthus guttatus	1	.	.	.	.	.	.	.	.	.	1	.	.	.	.	.	.
Lathyrus transsilvanicus	1	.	.	.	.	.	1	.	.	.	.	.	.	.	.	.	.
Hyssopus cretaceus	1	.	.	.	.	.	.	.	.	.	1	.	.	.	.	.	.
Mentha spicata	1	.	.	.	.	.	.	.	.	.	1	.	.	.	.	.	.
Cuscuta lupuliformis	1	.	.	.	.	.	1	.	.	.	.	.	.	.	.	.	.
Amaranthus powellii	1	.	.	.	.	.	.	.	.	.	1	.	.	.	.	.	.
Calendula officinalis	1	.	.	.	.	.	.	.	.	1	.	.	.	.	.	.	.
Lathyrus heterophyllus	1	.	.	.	.	.	.	.	.	.	1	.	.	.	.	.	.
Scrophularia canina	1	.	.	.	.	.	.	.	.	.	1	.	.	.	.	.	.
Allium atropurpureum	1	.	.	.	.	.	.	.	.	.	1	.	.	.	.	.	.
Linum strictum	1	.	.	.	.	.	1	.	.	.	.	.	.	.	.	.	.
Fritillaria montana	1	.	.	.	.	.	.	.	.	.	.	1	.	.	.	.	.
Dioscorea communis	1	.	.	.	.	.	.	.	1	.	.	.	.	.	.	.	.
Amaranthus albus	1	.	.	.	.	.	.	.	.	.	1	.	.	.	.	.	.
Trientalis europaea	1	.	.	.	.	1	.	.	.	.	.	.	.	.	.	.	.
Nonea atra	1	.	.	.	.	.	.	.	.	.	1	.	.	.	.	.	.
Hieracium sparsum	1	.	.	.	.	.	.	.	.	.	.	.	.	1	.	.	.
Bidens frondosus	1	.	.	.	.	.	1	.	.	.	.	.	.	.	.	.	.
Viola cretacea	1	.	.	.	.	.	.	.	.	.	1	.	.	.	.	.	.
Knautia fleischmanii	1	.	.	.	.	.	.	.	1	.	.	.	.	.	.	.	.
Diplotaxis cretacea	1	.	.	.	.	.	.	.	.	.	1	.	.	.	.	.	.
Viola dacica	1	.	.	.	.	.	1	.	.	.	.	.	.	.	.	.	.
Bunium bulbocastanum	1	.	.	.	.	.	.	.	.	.	1	.	.	.	.	.	.
Silene lithuanica	1	.	.	1	.	.	.	.	.	.	.	.	.	.	.	.	.
Allium decipiens	1	.	.	.	.	.	.	.	.	.	1	.	.	.	.	.	.
Viola uliginosa	1	.	.	.	.	.	1	.	.	.	.	.	.	.	.	.	.
Trifolium nigrescens	1	.	.	.	.	.	1	.	.	.	.	.	.	.	.	.	.
Achillea clavennae	1	.	.	.	.	.	.	.	.	.	.	.	.	1	.	.	.
Viola alba	1	.	.	.	.	.	.	.	.	.	.	.	.	1	.	.	.
Scilla sibirica	1	.	.	.	.	.	1	.	.	.	.	.	.	.	.	.	.
Centaurea besseriana	1	.	.	.	.	.	.	.	.	.	1	.	.	.	.	.	.
Trifolium strictum	1	.	.	.	.	.	.	.	.	.	1	.	.	.	.	.	.
Galium intermedium	1	.	.	.	.	.	.	.	.	.	1	.	.	.	.	.	.
Myagrum perfoliatum	1	.	.	.	.	.	1	.	.	.	.	.	.	.	.	.	.
Allium regelianum	1	.	.	.	.	.	.	.	.	.	1	.	.	.	.	.	.
Onosma helvetica	1	.	.	.	.	.	.	.	.	.	1	.	.	.	.	.	.
Vincetoxicum rossicum	1	.	.	.	.	.	1	.	.	.	.	.	.	.	.	.	.
Pyrola minor	1	.	.	.	.	1	.	.	.	.	.	.	.	.	.	.	.
Laser trilobum	1	.	.	.	.	.	.	.	.	1	.	.	.	.	.	.	.
Dryopteris carthusiana	1	.	.	.	.	1	.	.	.	.	.	.	.	.	.	.	.
Linum trigynum	1	.	.	.	.	.	.	.	1	.	.	.	.	.	.	.	.
Luzula forsteri	1	.	.	.	.	.	1	.	.	.	.	.	.	.	.	.	.
Eryngium amethystinum	1	.	.	.	.	.	.	.	.	1	.	.	.	.	.	.	.
Hieracium onosmoides	1	.	.	.	.	.	.	.	.	1	.	.	.	.	.	.	.
Ornithogalum fischerianum	1	.	.	.	.	.	.	.	.	.	1	.	.	.	.	.	.
Vicia crocea	1	.	.	.	.	.	1	.	.	.	.	.	.	.	.	.	.
Narcissus poeticus	1	.	.	.	.	.	.	.	.	.	1	.	.	.	.	.	.
Dianthus sylvestris	1	.	.	.	.	.	.	.	.	1	.	.	.	.	.	.	.
Drosera longifolia	1	.	.	.	.	.	1	.	.	.	.	.	.	.	.	.	.

Cluster number		1	2	3	4	5	6	7	8	9	10	11	12	13	14	15	16
No. of relevés (all)		257	260	213	797	1411	4434	526	1306	1779	5089	201	203	761	429	219	108
- (with NVP recorded)		133	133	51	234	471	1227	173	409	559	1421	46	121	145	59	100	12
**Bryophytes**																	
Campylium stellatum	96	33	10	.	1	1	2	.	1	1	.	.	.	.	.	.	.
Leptodictyum riparium	48	15	1	.	6	.	1	.	1	.	.	.	.	.	.	.	.
Fissidens adianthoides	49	14	10	.	.	1	1	.	1	.	.	.	.	.	.	.	.
Plagiomnium affine agg.	599	19	55	.	6	14	24	6	20	5	1	.	.	1	.	.	.
Climacium dendroides	383	11	40	2	6	18	17	.	2	1	1	.	.	.	.	.	.
Brachythecium rivulare	30	.	14	.	1	1	1	.	.	.	.	.	.	.	.	.	.
Bryum pseudotriquetrum	52	5	15	.	1	.	2	.	.	.	.	.	.	.	.	.	.
Cratoneuron filicinum	18	2	8	.	1	.	1	.	.	.	.	.	.	.	.	.	.
Cirriphyllum piliferum	156	.	14	.	1	5	7	.	5	1	.	.	.	.	.	.	.
Pleurozium schreberi	213	.	1	.	.	28	3	1	2	1	1	.	1	6	2	2	.
Rhytidiadelphus squarrosus	374	.	11	.	.	31	14	1	7	1	1	.	.	.	.	1	.
Polytrichum juniperinum	105	.	.	.	.	14	1	.	1	1	2	.	3	.	2	1	.
Fissidens taxifolius	142	.	.	.	1	1	1	3	8	13	1	.	.	.	.	1	.
Homalothecium lutescens	420	1	.	.	1	1	2	11	15	24	11	2	.	8	.	3	8
Abietinella abietina	972	.	1	.	.	14	7	28	31	39	27	.	3	22	8	10	8
Syntrichia ruralis agg.	305	.	.	.	.	1	1	3	1	2	14	65	7	15	14	4	17
Bryum caespiticium	181	.	.	.	1	1	1	1	4	6	7	17	2	1	3	.	8
Ceratodon purpureus	449	.	.	.	1	6	2	5	3	6	18	2	62	9	3	.	.
Polytrichum piliferum	213	.	.	.	1	2	1	1	1	.	9	9	50	6	2	.	.
Orthotrichum anomalum	39	.	.	.	.	.	.	.	.	.	2	.	.	9	.	4	.
Tortella inclinata	105	.	.	.	.	.	1	.	1	2	4	15	.	8	31	4	.
Grimmia orbicularis	5	.	.	.	.	.	.	.	.	.	1	.	.	.	7	.	.
Neckera crispa	8	.	.	.	.	.	.	.	.	.	.	.	.	.	.	8	.
Rhytidium rugosum	292	.	.	.	.	1	1	2	6	15	8	.	.	23	2	26	8
Hypnum vaucheri	10	.	.	.	.	.	.	.	.	.	1	.	.	1	2	7	.
Fissidens dubius	128	.	.	.	1	1	1	.	10	10	1	.	.	2	2	15	.
Distichium capillaceum	13	.	.	.	.	.	.	.	.	1	1	.	.	1	.	7	17
Pseudoleskea catenulata	10	.	.	.	.	.	.	.	.	.	1	.	.	1	.	1	8
Tortula muralis	18	.	.	.	.	.	.	.	.	1	1	.	.	.	.	2	8
Schistidium apocarpum agg.	61	.	.	.	.	.	.	.	1	1	2	.	.	8	2	12	17
Anomodon viticulosus	8	.	.	.	.	.	.	.	.	.	1	.	.	3	.	1	8
Calliergonella cuspidata	327	42	56	.	7	3	12	1	4	1	1	.	.	.	.	.	.
Tortella tortuosa	286	.	.	.	.	1	1	1	2	6	4	28	5	52	37	55	42
Ditrichum flexicaule	117	.	.	.	.	.	1	.	1	1	2	.	.	19	25	37	.
Ctenidium molluscum	40	2	2	.	.	1	1	.	1	1	.	.	.	2	.	18	17
Homalothecium philippeanum	43	.	.	.	.	.	.	.	1	1	1	.	.	12	.	17	17
Hypnum cupressiforme	423	.	.	.	.	4	1	3	8	8	18	.	6	19	8	14	8
Oxyrhynchium hians	366	5	12	.	8	6	13	6	7	8	4	.	.	.	.	.	.
Brachythecium rutabulum	335	2	13	.	10	2	18	8	5	3	1	.	.	.	.	.	.
Weissia spec. div.	264	.	.	.	.	1	1	2	2	7	13	.	1	5	12	.	.
Brachythecium albicans	190	.	.	.	1	7	4	3	5	2	4	2	7	.	.	.	.
Pseudoscleropodium purum	180	2	6	.	1	3	9	5	6	1	1	.	.	.	.	1	.
Thuidium assimile	157	2	8	.	.	6	4	2	11	3	1	.	.	.	.	2	.
Rhytidiadelphus triquetrus	156	.	3	.	.	10	3	1	11	2	.	.	.	3	.	10	8
Plagiomnium undulatum	148	.	13	.	1	1	7	5	5	1	1	.	.	.	.	4	.
Thuidium delicatulum	144	2	.	.	.	7	6	3	4	2	.	.	.	1	.	3	.
Atrichum undulatum	143	.	2	.	1	11	6	.	1	1	.	.	.	.	.	2	.
Bryum argenteum	136	.	.	.	1	1	.	1	1	4	5	.	10	12	2	2	8
Plagiomnium cuspidatum	123	.	1	.	.	3	5	8	5	1	1	.	.	1	.	.	.
Grimmia pulvinata	119	.	.	.	.	.	.	.	.	1	7	.	.	8	8	.	.
Racomitrium canescens agg.	109	.	.	.	.	5	1	1	1	1	4	.	7	3	.	2	.
Hylocomium splendens	105	.	2	.	.	9	1	2	3	2	1	.	.	2	.	13	8
Brachythecium salebrosum	95	.	1	.	1	4	4	1	4	1	1	2	.	.	.	.	.
Bryum capillare	78	1	.	.	1	1	1	1	1	1	4	.	.	.	.	3	.
Homalothecium sericeum	69	.	.	.	.	1	1	.	1	1	2	.	.	6	.	9	8
Brachythecium glareosum	65	1	.	.	1	3	1	3	1	1	1	.	.	.	.	.	.
Thuidium tamariscinum	62	1	1	.	.	6	2	1	1	.	1	.	.	.	.	.	.
Encalypta streptocarpa	59	.	.	.	.	.	.	.	1	1	1	.	1	11	5	11	.
Aulacomnium palustre	58	5	10	2	1	1	3	.	1	.	.	.	.	.	.	.	.
Pleurochaete squarrosa	58	.	.	.	.	.	.	.	1	1	3	2	.	2	5	.	.
Campyliadelphus chrysophyllus	50	.	2	.	.	.	.	.	5	3	1	.	.	.	2	2	.
Brachytheciastrum velutinum	49	.	.	.	3	1	2	.	1	1	1	.	.	.	.	2	.
Encalypta vulgaris	46	.	.	.	.	.	1	.	1	1	1	9	.	6	.	9	.
Barbula unguiculata	45	.	.	.	1	1	1	2	1	2	1	.	.	1	.	.	.
Dicranum scoparium	40	.	.	.	.	5	1	.	1	1	1	.	2	3	.	3	8
Barbula convoluta	37	.	.	.	.	.	1	1	1	2	1	7	.	1	.	1	.
Amblystegium serpens	33	.	.	.	1	1	1	2	1	1	1	.	.	.	.	.	.
Dicranum bonjeanii	32	4	2	.	.	2	1	.	1	.	.	.	.	.	.	.	.
Brachythecium mildeanum	31	2	4	.	1	.	2	.	.	.	.	.	.	.	.	.	.
Mannia fragrans	28	.	.	.	.	.	.	.	.	.	2	.	.	3	2	.	.
Drepanocladus aduncus	28	2	2	.	6	1	1	.	.	.	.	.	.	.	.	.	.
Campylophyllum calcareum	27	1	.	.	.	1	1	1	2	1	1	.	.	.	.	.	.
Tomentypnum nitens	26	5	8	.	1	.	1	.	.	.	.	.	.	.	.	.	.
Rhodobryum roseum	23	.	.	.	1	1	1	3	1	1	1	.	.	.	.	.	.
Calliergonella lindbergii	23	.	.	.	.	3	1	.	.	1	.	.	.	.	.	.	.
Riccia ciliata	21	.	.	.	.	.	.	.	.	.	1	.	.	.	.	.	.
Entodon concinnus	20	.	.	.	.	1	.	.	1	2	.	.	.	1	2	1	.

Cluster number		1	2	3	4	5	6	7	8	9	10	11	12	13	14	15	16
No. of relevés (all)		257	260	213	797	1411	4434	526	1306	1779	5089	201	203	761	429	219	108
- (with NVP recorded)		133	133	51	234	471	1227	173	409	559	1421	46	121	145	59	100	12
Brachythecium campestre	19	.	.	.	.	1	1	5	1	.	1	.	.	.	.	.	.
Breidleria pratensis	19	1	5	.	1	.	1	.	.	.	.	.	.	.	.	.	.
Syntrichia montana	19	.	.	.	.	.	.	.	1	.	1	.	.	1	.	2	.
Lophocolea bidentata	18	1	2	.	.	.	1	.	1	.	1	.	.	.	.	.	.
Thuidium recognitum	17	3	2	.	1	.	1	.	.	.	1	.	.	.	.	.	.
Scorpidium revolvens agg.	16	3	5	.	.	.	1	.	.	.	.	.	.	.	.	.	.
Campylophyllum sommerfeltii	16	.	.	.	.	1	1	.	1	1	1	.	.	1	.	.	.
Phascum cuspidatum	15	.	.	.	1	.	1	1	.	1	1	.	.	.	.	.	.
Polytrichastrum formosum	15	.	.	.	.	2	1	.	.	1	.	.	.	.	.	2	.
Didymodon acutus	15	.	.	.	.	.	.	.	.	1	1	.	.	.	2	.	.
Didymodon fallax	15	1	.	.	.	.	.	.	.	1	1	.	.	.	.	.	.
Rhizomnium punctatum	14	.	2	.	1	1	1	.	1	1	.	.	.	.	.	.	.
Polytrichum commune	13	.	.	.	1	2	1	.	.	.	.	.	.	.	.	.	.
Rhynchostegium megapolitanum	13	.	.	.	.	.	.	.	.	1	1	.	.	.	.	.	.
Eurhynchium striatum	13	.	.	.	1	.	1	.	.	1	.	.	.	.	.	.	.
Orthotrichum cupulatum	13	.	.	.	.	.	.	.	.	.	1	.	.	1	.	1	.
Riccia ciliifera	13	.	.	.	.	.	.	.	.	.	1	.	.	.	2	.	.
Oxyrrhynchium schleicheri	12	.	.	.	1	.	1	.	1	1	1	.	.	.	.	.	.
Leskea polycarpa	11	.	.	.	.	.	.	.	1	.	.	.	.	4	.	3	8
Palustriella commutata	10	3	3	.	.	.	1	.	.	.	.	.	.	.	.	.	.
Bryum atrovirens agg.	10	.	.	.	.	.	1	1	.	.	1	.	.	.	.	.	.
Plagiochila asplenioides	10	.	1	.	.	.	1	.	1	.	.	.	.	.	.	5	.
Leucodon sciuroides	9	.	.	.	.	.	.	.	.	.	1	.	.	1	.	.	.
Dicranella heteromalla	9	.	.	.	1	1	1	.	.	1	1	.	.	1	.	1	.
Dicranum polysetum	9	1	.	.	.	1	1	.	.	1	1	.	.	1	.	.	.
Aneura pinguis	8	1	4	.	.	.	1	.	.	.	.	.	.	.	.	.	.
Calliergon cordifolium	8	.	2	2	2	.	1	.	.	.	.	.	.	.	.	.	.
Pleuridium subulatum	8	.	.	.	1	.	1	1	.	.	1	.	.	.	.	.	.
Oxymitra incrassata	7	.	.	.	.	.	.	.	.	.	1	.	.	.	2	.	.
Eurhynchiastrum pulchellum	7	.	.	.	1	.	1	.	.	1	.	.	.	.	.	.	.
Bryum rubens	7	.	.	.	.	.	.	.	.	1	1	.	.	.	.	.	.
Sphagnum subsecundum	7	.	.	.	.	1	1	.	.	.	.	.	.	.	.	.	.
Tortula lanceolata	7	.	.	.	.	.	.	.	.	1	1	2	.	.	.	.	.
Hamatocaulis vernicosus	7	3	2	.	.	.	1	.	.	.	.	.	.	.	.	.	.
Schistidium brunnescens	6	.	.	.	.	.	.	.	.	.	1	.	.	1	.	.	.
Entodon schleicheri	6	.	.	.	.	.	1	.	.	1	.	.	.	.	.	.	.
Entosthodon fascicularis	6	.	.	.	.	.	.	1	.	.	1	.	.	.	.	.	.
Grimmia anodon	6	.	.	.	.	.	.	.	.	.	1	.	.	.	.	.	.
Calliergon giganteum	5	2	.	.	.	.	1	.	.	.	.	.	.	.	.	.	.
Pogonatum urnigerum	5	.	.	.	.	1	.	.	.	.	1	.	.	.	.	.	.
Bryum archangelicum	5	.	.	.	.	.	1	.	.	1	1	.	.	.	.	.	.
Rhodobryum ontariense	5	.	.	.	.	.	.	.	1	1	.	.	.	.	.	.	.
Leucobryum glaucum	5	.	.	.	.	1	.	.	.	.	.	.	.	.	.	.	.
Cephaloziella divaricata	5	.	.	.	.	1	.	.	.	.	1	.	.	.	.	.	.
Physcomitrium pyriforme	5	.	.	.	.	.	.	1	.	.	1	.	.	.	.	.	.
Fissidens viridulus	5	.	.	.	.	.	.	2	.	.	1	.	.	.	.	.	.
Tortella fragilis	5	.	.	.	.	.	.	.	.	.	1	.	.	.	.	.	.
Trichostomum crispulum	5	.	.	.	.	.	.	.	.	1	1	.	.	.	2	2	.
Hedwigia ciliata agg.	5	.	.	.	.	.	.	.	.	.	1	.	.	1	.	.	.
Pterygoneurum ovatum	5	.	.	.	.	.	.	.	.	1	1	.	.	.	.	.	.
Pohlia nutans	5	.	.	.	.	1	1	.	.	.	1	.	.	.	.	.	.
Sciurohypnum populeum	5	.	.	.	2	.	1	.	.	.	.	.	.	.	.	.	.
Tortula truncata	5	.	.	.	.	.	1	.	1	.	1	.	.	.	.	.	.
Mnium stellare	5	.	.	.	.	1	1	.	.	.	.	.	.	.	.	.	.
Didymodon cordatus	5	.	.	.	.	.	.	.	.	1	1	.	.	.	2	.	.
Protobryum bryoides	5	.	.	.	.	.	.	.	.	.	1	.	.	.	.	.	.
Ptilidium ciliare	4	.	.	.	.	1	.	.	.	.	.	.	.	.	.	.	.
Bryoerythrophyllum recurvirostrum	4	.	.	.	.	.	.	.	.	1	.	.	.	1	.	1	.
Bryum kunzei	4	.	.	.	.	.	.	.	.	.	1	.	.	.	.	.	.
Microbryum davallianum	4	.	.	.	.	.	.	.	.	.	1	.	.	.	.	.	.
Pterygoneurum subsessile	4	.	.	.	.	.	.	.	.	1	1	.	.	.	.	.	.
Sciurohypnum reflexum	4	.	.	.	.	.	1	.	.	.	.	.	.	.	.	.	.
Campyliadelphus elodes	4	2	.	.	.	.	1	.	.	.	.	.	.	.	.	.	.
Drepanocladus longifolius	4	1	.	.	1	.	1	.	1	.	.	.	.	.	.	.	.
Fissidens bryoides	4	.	.	.	.	.	.	1	.	.	1	.	.	.	.	.	.
Sanionia uncinata	4	.	.	.	.	.	1	.	.	.	.	.	.	.	.	.	.
Tortula modica	4	.	1	.	.	.	.	.	.	1	1	.	.	.	.	.	.
Sphagnum palustre	4	1	2	.	.	.	1	.	.	.	.	.	.	.	.	.	.
Pleuridium acuminatum	4	.	.	.	.	.	.	.	.	1	1	.	.	.	.	.	.
**Lichens**																	
Cladonia phyllophora	36	.	.	.	.	1	.	.	.	.	1	.	26	.	.	.	.
Cetraria aculeata	54	.	.	.	.	.	.	.	.	1	2	.	19	2	2	.	.
Cladonia furcata agg.	143	.	.	.	.	4	.	1	1	1	5	.	26	3	5	.	.
Cladonia pocillum	109	.	.	.	.	1	.	1	1	2	3	.	28	4	5	3	8
Cladonia rangiformis	231	.	.	.	.	1	.	2	1	1	12	4	30	7	5	.	.
Cladonia foliacea	169	.	.	.	.	.	.	1	1	1	8	15	29	6	5	.	.
Cladonia coccifera	9	.	.	.	.	.	.	.	.	.	.	.	7	.	.	.	.
Cetraria islandica	40	.	.	.	.	3	.	.	.	.	1	.	9	.	.	1	.
Psora decipiens	21	.	.	.	.	.	.	.	.	.	1	7	.	.	19	3	.

Cluster number		1	2	3	4	5	6	7	8	9	10	11	12	13	14	15	16
No. of relevés (all)		257	260	213	797	1411	4434	526	1306	1779	5089	201	203	761	429	219	108
- (with NVP recorded)		133	133	51	234	471	1227	173	409	559	1421	46	121	145	59	100	12
Squamarina cartilaginea	19	.	.	.	.	.	.	.	.	.	1	.	.	4	12	.	.
Aspicilia contorta	7	.	.	.	.	.	.	.	.	.	1	.	.	.	7	.	.
Fulgensia fulgens	6	.	.	.	.	.	.	.	.	.	1	.	.	1	7	.	.
Solorina saccata	12	.	.	.	.	.	.	.	.	.	.	.	.	.	.	12	.
Cladonia rei	7	.	.	.	.	.	.	.	.	.	1	.	2	.	.	.	8
Cladonia pyxidata	162	.	.	.	.	4	.	2	1	1	6	2	6	14	3	13	.
Cladonia symphycarpia	104	.	.	.	.	.	.	.	1	1	5	.	1	10	3	4	.
Cladonia fimbriata	78	.	.	.	.	1	.	.	1	1	3	.	9	5	3	4	8
Xanthoparmelia stenophylla	65	.	.	.	.	.	.	.	.	.	4	.	2	6	3	.	.
Cladonia arbuscula agg.	62	.	.	.	.	4	.	.	.	1	2	2	9	3	.	.	.
Cladonia rangiferina	59	.	.	.	.	3	.	.	1	1	2	2	.	4	5	.	.
Peltigera rufescens	58	.	.	.	.	1	.	.	1	1	2	.	4	4	8	3	.
Xanthoparmelia pulla	48	.	.	.	.	.	.	.	.	.	3	.	.	5	3	.	.
Cladonia convoluta	46	.	.	.	.	.	.	.	.	1	2	4	2	2	8	.	.
Collema sp.	38	.	.	.	.	.	.	.	.	.	3	.	.	.	.	.	.
Cladonia coniocraea	36	.	.	.	.	1	.	.	.	1	1	.	5	3	2	.	.
Cladonia chlorophaea	29	.	.	.	.	1	.	.	1	1	1	.	1	1	3	3	.
Cladonia glauca	28	.	.	.	.	1	.	1	1	1	1	.	1	.	.	.	.
Protoparmeliopsis muralis	28	.	.	.	.	.	.	.	.	1	2	.	.	1	.	.	.
Xanthoparmelia conspersa	26	.	.	.	.	.	.	.	.	.	2	.	.	1	.	.	.
Toninia sedifolia	24	.	.	.	.	.	.	.	.	1	1	2	.	1	5	1	.
Cladonia subulata	19	.	.	.	.	1	.	1	.	1	1	.	2	2	.	.	.
Diploschistes muscorum	15	.	.	.	.	1	.	1	1	1	1	.	.	.	.	.	.
Bacidia bagliettoana	14	.	.	.	.	1	.	.	1	1	1	.	.	.	2	.	.
Verrucaria muralis	12	.	.	.	.	1	.	1	1	1	1	.	.	.	2	.	.
Stereocaulon incrustatum	11	.	.	.	.	.	.	.	.	.	1	.	2	.	.	.	.
Candelariella vitellina	11	.	.	.	.	.	.	.	.	.	1	.	.	1	.	.	.
Bilimbia sabuletorum	10	.	.	.	.	1	.	.	1	1	1	.	.	1	.	.	.
Cladonia squamosa	9	.	.	.	.	1	.	.	.	.	1	.	2	.	.	2	.
Verrucaria nigrescens	8	.	.	.	.	.	.	1	.	1	1	.	.	1	.	.	.
Cladonia uncialis	8	.	.	.	.	1	.	.	.	.	1	.	2	.	.	.	.
Cladonia rangiferina	7	.	.	.	.	1	.	.	.	.	.	.	.	1	.	.	.
Lobothallia radiosa	7	.	.	.	.	.	.	.	.	.	1	.	.	.	.	.	.
Leptogium lichenoides	6	.	.	.	.	.	.	.	.	1	1	.	.	.	.	1	.
Peltigera canina	6	.	.	.	.	1	.	.	.	1	1	.	.	.	.	.	.
Lecanora campestris	6	.	.	.	.	.	.	.	.	.	1	.	.	.	.	.	.
Lecanora dispersa	6	.	.	.	.	.	.	1	1	1	.	.	.	.	.	.	.
Cladonia magyarica	6	.	.	.	.	.	.	.	.	.	1	.	.	.	2	.	.
Physcia wainioi	6	.	.	.	.	.	.	.	.	.	1	.	.	.	.	.	.
Verrucaria aethiobola	6	.	.	.	.	1	.	1	1	1	1	.	.	.	.	.	.
Parmelia saxatilis	5	.	.	.	.	.	.	.	.	.	1	.	.	1	.	.	.
Cladonia gracilis	5	.	.	.	.	1	.	.	.	.	1	.	1	.	.	.	.
Physcia dubia	5	.	.	.	.	.	.	.	.	.	1	.	.	1	.	.	.
Baeomyces rufus	5	.	.	.	.	.	.	.	.	.	1	.	.	.	.	.	.
Collema tenax	5	.	.	.	.	.	.	.	1	.	1	.	.	.	2	.	.
Cladonia verticillata	5	.	.	.	.	.	.	.	.	.	1	.	1	.	2	.	.
Cladonia polycarpoides	5	.	.	.	.	.	.	.	.	.	1	.	.	.	3	.	.
Rhizocarpon geographicum	5	.	.	.	.	.	.	.	.	.	1	.	.	1	.	.	.
Ramalina capitata	4	.	.	.	.	.	.	.	.	.	1	.	.	1	.	.	.
Candelariella aurella	4	.	.	.	.	.	.	.	.	1	.	.	.	1	.	.	.
Cladonia turgida	4	.	.	.	.	1	.	.	.	.	.	.	.	.	.	.	.
Lecidea fuscoatra	4	.	.	.	.	.	.	.	.	.	1	.	.	1	.	.	.
Xanthoparmelia protomatrae	4	.	.	.	.	.	.	.	.	.	1	.	.	1	.	.	.
Mycobilimbia lurida	4	.	.	.	.	.	.	.	.	.	1	.	.	1	.	.	.
Dibaeis baeomyces	4	.	.	.	.	1	.	.	.	.	.	.	.	.	.	.	.
Agonimia tristicula	4	.	.	.	.	.	.	.	1	.	1	.	.	.	.	1	.
