# Supplementary material for: A higher‐level classification of the Pannonian and western Pontic steppe grasslands (Central and Eastern Europe)
Source: Appl Veg Sci. 2016 Sep 16;20(1):143–58. doi: 10.1111/avsc.12265 (PMC5348766; doi:10.1111/avsc.12265)
Supplement: Supplementary file 5 — Appendix S5. Average percentage cover of the diagnostic species of five grassland classes in the TWINSPAN clusters. [file AVSC-20-143-s005.pdf]

Supporting information to the paper

Willner, W. et al. A higher-level classification of the Pannonian and western Pontic steppe grasslands (Central and Eastern Europe). *Applied Vegetation Science*.

Appendix S5. Average percentage cover of the diagnostic species of five grassland classes in the TWINSPAN clusters (division levels 4-6). The total cover of each diagnostic species group was calculated for each plot (using the Juice function "Percentage cover of <colour> species") and averaged among the plots of each cluster. On the right a preliminary syntaxonomic classification. FES: *Festuco-Brometea*, MOL: *Molinio-Arrhenatheretea*, NAR: *Nardetea strictae*, COR: *Koelerio-Corynephoretea*, SES: *Elyno-Seslerietea*.

level 4	level 5-6	No. of rel.	FES	MOL	NAR	COR	SES	class	order	alliance	comments
**1**	0-0	38	5.70	47.16	6.91	0.05	0.05	MOL	Molinietalia	Molinion caeruleae	
**1**	0-1	21	3.12	52.22	11.94	0.00	0.57	MOL	Molinietalia	Molinion caeruleae	
**1**	1-0	23	3.20	75.30	4.48	0.00	0.00	MOL	Molinietalia	Molinion caeruleae	
**1**	1-1	175	5.12	62.96	4.96	0.03	0.02	MOL	Molinietalia	Molinion caeruleae	
**2**	0-0	147	1.76	70.64	7.13	0.00	0.01	MOL	Molinietalia	Calthion palustris	
**2**	0-1	15	1.33	45.19	1.07	0.00	0.00	MOL	Molinietalia	Calthion palustris	
**2**	1-0	83	1.02	81.02	10.36	0.10	0.00	MOL	Molinietalia	Calthion palustris	
**2**	1-1	15	1.93	60.20	2.51	0.00	0.00	MOL	Molinietalia	Calthion palustris	
**3**	0-0	14	0.57	23.03	0.14	18.64	0.00	MOL	Galietalia veri	Agrostion vinealis	transitional towards Deschampsion
**3**	0-1	35	2.02	14.67	2.12	20.51	0.00	MOL	Galietalia veri	Agrostion vinealis	
**3**	1-0	3	0.67	17.87	0.00	12.67	0.00				(Scorzonero-Juncion gerardii)
**3**	1-1	161	10.07	24.41	6.57	10.80	0.00	MOL	Galietalia veri	Agrostion vinealis	
**4**	0-0	476	3.66	68.26	2.77	0.40	0.00	MOL	Molinietalia	Deschampsion caespitosae	
**4**	0-1	197	1.34	66.53	0.41	0.28	0.01	MOL	Molinietalia	Deschampsion caespitosae	
**4**	1-0	96	5.62	60.03	1.43	0.09	0.00	MOL	Potentillo-Polygonetalia	Potentillion anserinae	
**4**	1-1	28	2.89	44.72	0.00	0.00	0.21	MOL	Potentillo-Polygonetalia	Potentillion anserinae	
**5**	0-0	136	4.19	16.33	72.04	0.00	0.43	NAR	Nardetalia	Violion caninae	
**5**	0-1	400	11.89	26.37	55.79	0.16	0.06	NAR	Nardetalia	Violion caninae	
**5**	1-0	185	2.77	44.16	58.36	0.00	0.11	NAR	Nardetalia	Violion caninae	
**5**	1-1	690	17.15	45.21	47.77	0.08	0.33	NAR	Nardetalia	Violion caninae	
**6**	0-0	128	15.46	59.15	16.60	0.19	0.37	MOL	Molinietalia		transitional towards Nardetalia and Arrhenatheretalia
**6**	0-1	308	8.93	65.85	21.29	0.32	0.10	MOL	Molinietalia		transitional towards Nardetalia and Arrhenatheretalia
**6**	1-0	2019	16.06	55.95	24.05	0.37	0.19	MOL	Arrhenatheretalia		
**6**	1-1	1979	13.91	63.34	5.89	0.63	0.03	MOL	Arrhenatheretalia		
**7**	0-0	98	48.70	16.88	15.01	4.62	0.00	FES	Brometalia erecti	Cirsio-Brachypodion?	acidic, partly transitional towards Chrysopogono-Danthonion
**7**	0-1	286	43.23	34.25	2.93	0.66	0.12	FES	Brometalia erecti	Cirsio-Brachypodion?	
**7**	1-0	91	22.29	16.11	8.75	0.96	0.05	FES	Brometalia erecti	Scabioso-Poion angustifoliae	
**7**	1-1	51	44.65	20.39	4.89	0.80	0.04	FES	Brometalia erecti	Scabioso-Poion angustifoliae	
**8**	0-0	907	60.37	25.07	9.09	0.21	0.67	FES	Brometalia erecti	Cirsio-Brachypodion s.lat.	calcium-poor soils; "Bromion" [Filipendulo-Brometum]
**8**	0-1	164	59.41	32.78	11.26	0.28	0.45	FES	Brometalia erecti	Cirsio-Brachypodion s.lat.	calcium-poor soils; "Bromion" [Brachypodio-Molinietum]
**8**	1-0	112	80.64	19.01	2.84	0.09	5.92	FES	Brometalia erecti	Cirsio-Brachypodion s.lat.	calcium-poor soils; illyric "Bromion"
**8**	1-1	123	75.76	10.13	7.61	0.04	9.04	FES	Brometalia erecti	Cirsio-Brachypodion s.lat.	calcium-poor soils; illyric "Bromion" [Bromo-Danthonietum]
**9**	0-0	217	74.34	3.49	2.18	0.41	6.13	FES	Brometalia erecti	Cirsio-Brachypodion s.lat.	calcareous soils
**9**	0-1	713	66.56	7.65	1.90	0.27	0.43	FES	Brometalia erecti	Cirsio-Brachypodion s.lat.	calcareous soils
**9**	1-0	357	77.24	5.85	1.20	0.17	0.27	FES	Brometalia erecti	Cirsio-Brachypodion s.lat.	calcareous soils
**9**	1-1	492	70.98	7.33	0.94	0.03	0.07	FES	Brometalia erecti	Cirsio-Brachypodion s.lat.	calcareous soils
**10**	0-0	2203	55.67	3.10	2.26	2.65	0.39	FES	Festucetalia valesiacae	Festucion valesiacae	
**10**	0-1	1065	68.72	1.03	0.40	0.86	0.25	FES	Festucetalia valesiacae	Festucion valesiacae	includes also miss-classified Brometalia
**10**	1-0	1642	61.21	7.78	2.60	3.21	0.02	FES	Festucetalia valesiacae	Festucion valesiacae	includes also miss-classified Brometalia
**10**	1-1	179	77.36	0.20	0.14	3.32	0.00	FES	Festucetalia valesiacae	Stipion lessingianae	

level 4	level 5-6	No. of rel.	FES	MOL	NAR	COR	SES		class	order	alliance	comments
**11**	0-0	53	13.51	0.08	0.08	39.40	0.02		COR	Festucetalia vaginatae	Festucion vaginatae	
**11**	0-1	127	21.27	0.93	0.05	33.44	0.00		COR	Festucetalia vaginatae	Festucion vaginatae	
**11**	1-0	11	3.61	1.54	0.00	34.27	0.00		COR	Festucetalia vaginatae	Festucion beckeri	
**11**	1-1	10	7.45	0.40	0.00	22.32	0.00		COR	Festucetalia vaginatae	Festucion beckeri	Festuca polesica probably miss-determined
**12**	0-0	105	8.70	1.52	4.26	43.32	0.02		COR	Corynephoretalia	Corynephorion	includes also Koelerion glaucae
**12**	0-1	44	4.34	2.48	0.68	43.39	0.00		COR	Corynephoretalia	Corynephorion	includes also Koelerion glaucae and Armerion elongatae
**12**	1-0	22	12.34	1.04	1.54	43.12	0.00		COR	?	Bassio-Bromion tectorum	
**12**	1-1	32	19.48	4.07	1.65	14.85	0.00		COR	?	Bassio-Bromion tectorum	
**13**	0-0	6	50.60	0.00	0.00	0.33	0.00		FES	Stipo-Festucetalia pall.	Asplenio-Festucion pallentis	
**13**	0-1	59	23.50	0.03	1.33	0.12	2.19		FES	Stipo-Festucetalia pall.	Asplenio-Festucion pallentis	
**13**	1-0	339	51.98	0.58	0.35	0.55	7.63		FES	Stipo-Festucetalia pall.	Bromo-Festucion pallentis	incl. Diantho-Seslerion p.p.
**13**	1-1	357	61.74	0.55	1.09	0.30	21.41		FES	Stipo-Festucetalia pall.	Bromo-Festucion pallentis	incl. Diantho-Seslerion p.p.
**14**	0-0	162	58.87	0.07	0.69	0.86	0.88		FES	Stipo-Festucetalia pall.	Bromo-Festucion pallentis	
**14**	0-1	14	47.66	0.00	0.79	0.07	14.41		FES	Stipo-Festucetalia pall.	Bromo-Festucion pallentis	
**14**	1-0	238	47.88	0.01	0.02	0.20	0.14		FES	Stipo-Festucetalia pall.	Bromo-Festucion pallentis	
**14**	1-1	15	72.40	0.00	0.13	0.13	4.33		FES	Stipo-Festucetalia pall.	Bromo-Festucion pallentis	
**15**	0-0	155	45.75	2.35	0.99	0.08	41.75		FES	Stipo-Festucetalia pall.	Diantho-Seslerion	incl. Pulsatillo-Caricenion humilis
**15**	0-1	61	34.99	6.27	1.76	0.00	21.40		FES	Stipo-Festucetalia pall.	Diantho-Seslerion	incl. Pulsatillo-Caricenion humilis
**15**	1-0	1	2.00	11.40	0.00	0.00	2.00					clearing vegetation?
**15**	1-1	2	1.50	1.00	1.00	0.00	0.00					clearing vegetation?
**16**	0-0	22	39.95	2.18	0.00	0.00	3.09		FES	Stipo-Festucetalia pall.	?	poor Sesleria heuflerana grasslands
**16**	0-1	18	8.35	2.64	1.11	0.11	0.94					fringe community
**16**	1-0	53	45.89	0.88	0.49	0.34	44.54		FES	Stipo-Festucetalia pall.	Seslerion rigidae	
**16**	1-1	15	34.36	4.53	0.53	0.13	26.21		FES	Stipo-Festucetalia pall.	Seslerion rigidae	
