# Supplementary material for: A higher‐level classification of the Pannonian and western Pontic steppe grasslands (Central and Eastern Europe)
Source: Appl Veg Sci. 2016 Sep 16;20(1):143–58. doi: 10.1111/avsc.12265 (PMC5348766; doi:10.1111/avsc.12265)
Supplement: Supplementary file 6 — Appendix S6. Fidelity, constancy and average cover of species in the three Festuco‐Brometea orders. [file AVSC-20-143-s006.pdf]

Supporting information to the paper

Willner, W. et al. A higher-level classification of the Pannonian and western Pontic steppe grasslands (Central and Eastern Europe). Applied Vegetation Science.

Appendix S6. Fidelity, constancy and average cover of species in the three Festuco-Brometea orders Brometalia erecti (B), Festucetalia valesiacae (Fv) and Stipo-Festucetalia pallentis (S-Fp). Values matching the threshold of one of the three fidelity measures used are marked in colour (see Methods for details). The accepted diagnostic species are indicated in the last column. They are either character species (C) or differential species (D). Character species are diagnostic against all other orders of the study area while differential species are diagnostic only against the two other orders within the class). Species with constancy <5% were not considered. Non-vascular plants are given at the end of the table. Fidelity and constancy of the latter were calculated within the subset of relevés where these species were recorded.

	total no. of occurrences	Phi value x 100 (orange: phi >= 0.2)			Constancy in % (green: const. ratio >= 2)			Average cover in % (blue: cover ratio >= 2)			Diagnostic value	Comment
Phytosociological order		B	Fv	S-Fp	B	Fv	S-Fp	B	Fv	S-Fp		
Number of relevés		3470	5016	1472	3470	5016	1472	3470	5016	1472		
**1. Automatically accepted diagnostic species (i.e., species reaching the threshold of all three fidelity measures)**												
Brachypodium pinnatum agg.	2544	63	---	---	63	6	4	15.80	0.85	0.33	B (D)	only D because with similar constancy in Trifolio-Geranietea
Briza media	1946	60	---	---	52	2	3	2.21	0.09	0.06	B	
Dactylis glomerata	2188	51	---	---	48	10	1	1.51	0.42	0.01	B (D)	
Plantago media	3439	46	---	---	60	25	5	1.78	0.90	0.10	B	
Salvia pratensis	3069	44	---	---	56	20	8	3.18	1.03	0.15	B	
Leontodon hispidus	1805	44	---	---	40	8	2	1.54	0.25	0.06	B (D)	
Trifolium montanum	1718	44	---	---	38	8	1	1.39	0.41	0.01	B	
Lotus corniculatus	2930	43	---	---	54	19	7	1.56	0.62	0.13	B (D)	
Knautia arvensis	1837	43	---	---	40	9	2	0.97	0.24	0.05	B (D)	
Centaurea jacea	1388	42	---	---	33	5	1	1.05	0.15	0.00	B (D)	
Arrhenatherum elatius	1707	40	---	---	35	9	1	2.28	0.63	0.01	B (D)	
Leucanthemum vulgare agg.	1631	40	---	---	39	2	11	0.96	0.06	0.23	B (D)	
Centaurea scabiosa	2595	39	---	---	48	16	10	1.80	0.50	0.23	B (D)	
Bromus erectus	1533	39	---	---	34	7	3	10.39	1.75	0.28	B	
Pimpinella saxifraga agg.	3175	38	---	---	53	23	11	1.26	0.58	0.23	B (D)	
Linum catharticum	1468	37	---	---	34	3	9	0.82	0.09	0.19	B	
Trifolium pratense	1241	37	---	---	28	5	1	0.95	0.21	0.01	B (D)	
Carex caryophyllea	1289	34	---	---	27	7	1	0.92	0.22	0.02	B	
Filipendula vulgaris	1812	33	---	---	32	13	1	1.59	0.68	0.01	B	
Anthoxanthum odoratum agg.	933	33	---	---	22	4	1	0.99	0.19	0.00	B (D)	
Carex flacca	610	33	---	---	17	1	1	0.99	0.01	0.01	B (D)	
Thymus pulegioides	1158	32	---	---	25	5	3	1.14	0.23	0.08	B (D)	
Carlina acaulis	1124	32	---	---	26	2	7	0.83	0.05	0.13	B (D)	
Festuca pratensis	804	32	---	---	19	3	1	0.64	0.08	0.00	B (D)	
Betonica officinalis	756	32	---	---	19	2	1	0.64	0.07	0.01	B (D)	
Trisetum flavescens	531	32	---	---	15	1	0	0.60	0.01	0.00	B (D)	
Ranunculus polyanthemos agg.	1263	31	---	---	25	8	1	0.50	0.34	0.01	B (D)	
Tragopogon pratensis	790	31	---	---	19	3	1	0.38	0.04	0.01	B (D)	
Vicia cracca	744	31	---	---	18	2	1	0.49	0.04	0.03	B (D)	
Festuca rubra agg.	740	31	---	---	18	2	1	0.97	0.17	0.02	B (D)	
Campanula glomerata	727	31	---	---	18	2	1	0.47	0.04	0.03	B	
Polygala comosa	802	30	---	---	18	3	1	0.37	0.11	0.01	B	
Cruciata glabra	607	30	---	---	16	1	1	0.51	0.02	0.03	B (D)	
Cirsium pannonicum	529	30	---	---	15	1	1	0.69	0.01	0.03	B (D)	perhaps C but also frequent in Trifolio-Geranietea
Agrostis capillaris	795	29	---	---	18	4	1	1.19	0.24	0.00	B (D)	
Viola hirta	1489	28	---	---	29	7	10	0.71	0.14	0.19	B (D)	
Daucus carota	1359	28	---	---	24	10	1	0.58	0.28	0.00	B (D)	
Veronica chamaedrys	844	28	---	---	19	4	2	0.41	0.10	0.04	B (D)	
Ononis spinosa	799	28	---	---	17	4	1	0.74	0.20	0.00	B	
Avenula pubescens	618	28	---	---	15	2	1	0.78	0.09	0.01	B (D)	
Prunella vulgaris	596	28	---	---	15	2	1	0.34	0.04	0.00	B (D)	
Ranunculus bulbosus	556	28	---	---	14	1	1	0.37	0.03	0.01	B	
Luzula campestris agg.	700	27	---	---	15	3	1	0.40	0.09	0.00	B (D)	
Prunella grandiflora	574	27	---	---	15	1	2	0.60	0.03	0.06	B	
Carex montana	481	27	---	---	13	1	1	1.37	0.05	0.01	B (D)	perhaps C but also frequent in Trifolio-Geranietea and Quercetea pubescentis
Koeleria pyramidata	432	26	---	---	11	1	1	0.70	0.01	0.02	B	

Phytosociological order	total no. of occurrences	Phi value x 100 (orange: phi >= 0.2)			Constancy in % (green: const. ratio >= 2)			Average cover in % (blue: cover ratio >= 2)			Diagnostic value	Comment
Phytosociological order		B	Fv	S-Fp	B	Fv	S-Fp	B	Fv	S-Fp		
Number of relevés		3470	5016	1472	3470	5016	1472	3470	5016	1472		
Seseli annuum	782	25	---	---	16	5	1	0.31	0.08	0.01	B	
Onobrychis viciifolia agg.	1037	24	---	---	19	8	1	0.74	0.22	0.01	B	
Peucedanum cervaria	747	24	---	---	17	2	4	1.43	0.09	0.09	B (D)	
Primula veris	699	24	---	---	16	1	4	0.54	0.06	0.08	B (D)	
Rumex acetosa	460	24	---	---	11	1	1	0.21	0.03	0.00	B (D)	
Rhinanthus minor	458	24	---	---	11	2	1	0.39	0.07	0.00	B (D)	
Carex tomentosa	358	24	---	---	10	1	1	0.42	0.01	0.01	B (D)	
Campanula patula	327	23	---	---	9	1	1	0.18	0.01	0.00	B (D)	
Lathyrus pratensis	304	23	---	---	8	1	1	0.21	0.01	0.00	B (D)	
Potentilla erecta	283	23	---	---	8	1	0	0.30	0.00	0.00	B (D)	
Taraxacum sect. Ruderalia	714	22	---	---	14	4	1	0.30	0.11	0.02	B (D)	
Colchicum autumnale	294	22	---	---	8	1	0	0.23	0.01	0.00	B (D)	
Polygala major	449	21	---	---	11	1	1	0.24	0.02	0.03	B	
Danthonia alpina	244	21	---	---	7	1	0	1.38	0.01	0.00	B	
Carlina vulgaris agg.	1092	20	---	---	19	7	5	0.38	0.14	0.09	B	
Buphthalmum salicifolium	410	20	---	---	10	1	3	0.48	0.00	0.08	B (D)	
Carex michelii	407	20	---	---	9	2	1	0.37	0.06	0.00	B (D)	perhaps C but also frequent in Trifolio-Geranietea and Quercetea pubescentis
Ranunculus acris	281	20	---	---	7	1	1	0.17	0.02	0.00	B (D)	
Thymus longicaulis	198	20	---	---	6	0	0	0.16	0.00	0.00	B (D)	
Festuca valesiaca	3011	---	47	---	12	50	6	1.20	11.86	0.38	Fv	
Eryngium campestre	3044	---	46	---	16	49	1	0.34	1.40	0.01	Fv	
Thymus pannonicus agg.	3079	---	36	---	20	46	6	0.80	3.43	0.13	Fv	
Bothriochloa ischaemum	1997	---	34	---	7	33	7	0.41	3.90	0.24	Fv	
Koeleria macrantha	2628	---	32	---	16	39	8	0.62	3.18	0.25	Fv	
Stipa capillata	1310	---	30	---	2	23	6	0.09	3.80	0.30	Fv	
Salvia nemorosa	1055	---	30	---	4	18	1	0.12	1.23	0.00	Fv	
Centaurea stoebe	2051	---	27	---	10	31	10	0.24	0.81	0.17	Fv	
Potentilla argentea	1081	---	24	---	6	17	1	0.13	0.61	0.02	Fv (D)	
Falcaria vulgaris	996	---	24	---	6	16	1	0.13	0.45	0.00	Fv (D)	
Trifolium arvense	665	---	24	---	2	12	1	0.07	0.56	0.01	Fv (D)	
Astragalus onobrychis	947	---	23	---	5	15	1	0.13	0.64	0.02	Fv	
Salvia nutans	503	---	22	---	1	9	0	0.05	0.79	0.00	Fv	
Artemisia austriaca	357	---	22	---	1	7	0	0.00	0.47	0.00	Fv	
Artemisia campestris	1149	---	21	---	4	18	6	0.09	0.78	0.17	Fv (D)	perhaps C but also frequent in Koelerio-Corynephoretea
Euphorbia nicaeensis	767	---	20	---	4	12	1	0.21	0.70	0.02	Fv (D)	perhaps C but also frequent in Koelerio-Corynephoretea
Nonea pulla	582	---	20	---	3	10	0	0.04	0.24	0.00	Fv	
Verbascum phoeniceum	574	---	20	---	2	10	1	0.04	0.22	0.00	Fv	
Cleistogenes serotina	365	---	20	---	0	7	1	0.00	0.36	0.01	Fv	
Festuca pallens agg.	1124	---	---	60	1	6	55	0.05	0.78	7.09	S-Fp	
Teucrium montanum	1583	---	---	47	5	13	51	0.15	0.59	2.17	S-Fp	
Jovibarba globifera	709	---	---	45	1	4	34	0.02	0.11	1.03	S-Fp (D)	
Sesleria caerulea	473	---	---	43	1	1	28	0.20	0.10	8.24	S-Fp (D)	
Thymus praecox	1147	---	---	42	3	9	40	0.11	0.33	1.15	S-Fp (D)	
Asplenium ruta-muraria	446	---	---	40	1	2	25	0.00	0.03	0.52	S-Fp (D)	
Carex humilis	2637	---	---	39	17	23	59	2.70	3.77	10.17	S-Fp	
Leontodon incanus	456	---	---	39	2	1	25	0.10	0.01	0.74	S-Fp	
Helianthemum canum	453	---	---	37	1	2	23	0.02	0.09	1.23	S-Fp	
Fumana procumbens	388	---	---	37	1	1	21	0.00	0.05	0.57	S-Fp	
Anthericum ramosum	1798	---	---	34	20	9	46	1.60	0.46	2.15	S-Fp (D)	perhaps C but also frequent in Trifolio-Geranietea and Quercetea pubescentis
Scorzonera austriaca	385	---	---	34	1	1	20	0.01	0.02	0.27	S-Fp	
Vincetoxicum hirundinaria	1265	---	---	33	9	8	36	0.28	0.27	0.91	S-Fp (D)	
Sedum album	495	---	---	33	1	3	21	0.01	0.20	0.98	S-Fp (D)	
Seseli osseum	1098	---	---	32	1	12	30	0.03	0.30	0.88	S-Fp	
Allium lusitanicum	621	---	---	31	2	4	23	0.07	0.22	0.95	S-Fp	
Phyteuma orbiculare	302	---	---	30	2	0	16	0.06	0.00	0.39	S-Fp (D)	
Pulsatilla halleri subsp. slavica	211	---	---	30	1	1	13	0.02	0.00	0.40	S-Fp (D)	
Globularia bisnagarica	771	---	---	29	7	3	25	0.26	0.10	0.47	S-Fp	
Genista pilosa	554	---	---	29	2	3	20	0.13	0.22	1.22	S-Fp (D)	

	total no. of occurrences	Phi value x 100 (orange: phi >= 0.2)			Constancy in % (green: const. ratio >= 2)			Average cover in % (blue: cover ratio >= 2)			Diagnostic value	Comment
Phytosociological order		B	Fv	S-Fp	B	Fv	S-Fp	B	Fv	S-Fp		
Number of relevés		3470	5016	1472	3470	5016	1472	3470	5016	1472		
Stipa eriocaulis	294	---	---	29	1	1	15	0.00	0.19	3.22	S-Fp	
Asplenium trichomanes	257	---	---	28	1	1	14	0.00	0.02	0.33	S-Fp (D)	
Biscutella laevigata	238	---	---	27	1	1	13	0.02	0.01	0.23	S-Fp (D)	
Hornungia petraea	189	---	---	27	1	1	11	0.00	0.01	0.07	S-Fp (D)	
Saxifraga paniculata	165	---	---	27	0	1	11	0.00	0.01	0.77	S-Fp (D)	
Arabidopsis arenosa	337	---	---	25	1	2	14	0.03	0.06	0.28	S-Fp (D)	
Clinopodium alpinum	276	---	---	25	2	1	13	0.04	0.02	0.31	S-Fp (D)	
Polygonatum odoratum	473	---	---	24	4	2	16	0.09	0.04	0.40	S-Fp (D)	
Melica ciliata	678	---	---	23	1	8	18	0.01	0.36	0.74	S-Fp	
Poa badensis	254	---	---	23	1	2	11	0.01	0.08	0.25	S-Fp	
Campanula rotundifolia	604	---	---	22	6	3	18	0.13	0.06	0.35	S-Fp (D)	
Polygala amara agg.	204	---	---	22	1	1	10	0.03	0.00	0.17	S-Fp (D)	
Carduus defloratus	151	---	---	22	1	1	8	0.02	0.00	0.22	S-Fp (D)	
Minuartia laricifolia	112	---	---	22	1	1	7	0.00	0.00	0.21	S-Fp (D)	
Seseli leucospermum	110	---	---	22	0	1	7	0.00	0.00	0.23	S-Fp	
Hieracium bupleuroides	104	---	---	22	1	0	7	0.00	0.00	0.15	S-Fp (D)	
Linum tenuifolium	715	---	---	21	4	6	18	0.09	0.10	0.31	S-Fp	
Alyssum montanum	439	---	---	21	1	4	13	0.02	0.12	0.27	S-Fp	only subsp. montanum while subsp. gmelinii is C of Koelerio-Corynephoretea
Galium pusillum agg.	305	---	---	21	3	1	12	0.06	0.01	0.28	S-Fp (D)	
Minuartia setacea	237	---	---	21	1	2	9	0.00	0.11	0.26	S-Fp (D)	
Thymus comosus	214	---	---	21	1	1	9	0.01	0.07	0.28	S-Fp (D)	
Dianthus praecox	116	---	---	21	1	1	7	0.00	0.00	0.21	S-Fp	
Erysimum witmannii	113	---	---	21	1	1	7	0.00	0.00	0.15	S-Fp (D)	
Kernera saxatilis	99	---	---	21	0	0	7	0.00	0.00	0.14	S-Fp (D)	
Draba lasiocarpa	98	---	---	21	0	1	7	0.00	0.00	0.13	S-Fp	
Erysimum odoratum	454	---	---	20	3	3	13	0.04	0.08	0.26	S-Fp	
Thesium alpinum	114	---	---	20	1	0	7	0.01	0.00	0.14	S-Fp (D)	
Scabiosa lucida	106	---	---	20	1	0	7	0.01	0.00	0.16	S-Fp (D)	
Thymus pulcherrimus	94	---	---	20	1	1	6	0.01	0.00	0.23	S-Fp (D)	
Sesleria rigida	92	---	---	20	1	1	6	0.00	0.00	2.01	S-Fp (D)	
Dianthus plumarius	90	---	---	20	0	1	6	0.00	0.00	0.12	S-Fp	
**2. Individually evaluated species (i.e., species reaching the threshold of only one or two fidelity measures)**												
Festuca stricta subsp. sulcata	3367	40	---	---	55	27	5	7.32	4.09	0.21		only C of class in Mucina & Kolbek (1993) and Dengler (2003), absent in Brometalia W of the study area
Achillea millefolium agg.	5269	35	17	---	68	56	7	1.84	2.35	0.15		aggregate of species with very different habitat preferences
Plantago lanceolata	2945	35	---	---	46	27	1	1.21	0.86	0.03	B (D)	also D in Dengler (2003)
Galium verum	3650	31	12	---	50	38	2	1.81	2.07	0.02		very low constancy ratio
Poa pratensis agg.	3295	27	15	---	43	36	1	2.09	4.29	0.01		aggregate of species with very different habitat preferences
Agrimonia eupatoria	2182	27	---	---	32	21	1	0.91	0.80	0.01		indicator of abandoned sites, very low constancy ratio
Fragaria viridis	2252	25	---	---	33	21	2	1.54	1.28	0.04		indicator of abandoned sites, very low constancy ratio
Securigera varia	2664	21	---	---	36	25	9	1.21	0.77	0.18		very low constancy ratio
Galium mollugo agg.	1608	20	---	0	29	7	18	0.88	0.21	0.66		aggregate of species with very different habitat preferences
Clinopodium vulgare	631	19	---	---	12	4	2	0.30	0.10	0.05	B (D)	phi threshold only missed by 0.01
Cerastium fontanum subsp. vulgatum	564	19	---	---	11	4	1	0.22	0.11	0.01	B (D)	phi threshold only missed by 0.01
Tanacetum corymbosum	546	19	---	---	12	2	4	0.27	0.04	0.07	B (D)	phi threshold only missed by 0.01
Trifolium medium	499	19	---	---	10	3	1	0.53	0.16	0.00	B (D)	phi threshold only missed by 0.01
Prunella laciniata	414	19	---	---	9	2	1	0.21	0.04	0.01	B	phi threshold only missed by 0.01, also D in Hegedüšová Vantarová & Škodová (2014)
Polygala vulgaris	392	19	---	---	9	2	1	0.21	0.06	0.01	B (D)	phi threshold only missed by 0.01, also D in Mucina & Kolbek (1993)
Alchemilla spec.div.	213	19	---	---	6	1	1	0.16	0.00	0.00	B (D)	phi threshold only missed by 0.01
Euphorbia verrucosa	196	19	---	---	6	1	0	0.17	0.00	0.00	B	phi threshold only missed by 0.01, also C in Mucina & Kolbek (1993)
Potentilla heptaphylla	951	18	---	---	19	3	11	0.47	0.08	0.27	B	also C in Dengler (2003)
Danthonia decumbens	235	18	---	---	6	1	0	0.22	0.02	0.00		
Potentilla alba	186	18	---	---	5	1	1	0.31	0.01	0.00		
Silene vulgaris	582	17	---	---	11	3	3	0.27	0.07	0.08		
Hypochaeris maculata	467	17	---	---	9	3	1	0.23	0.13	0.01	B	C of Cirsio-Brachypodion in Mucina & Kolbek (1993) and Borhidi (1996)
Crepis biennis	202	17	---	---	5	1	1	0.11	0.01	0.00		

	total no. of occurrences	Phi value x 100 (orange: phi >= 0.2)			Constancy in % (green: const. ratio >= 2)			Average cover in % (blue: cover ratio >= 2)			Diagnostic value	Comment
Phytosociological order		B	Fv	S-Fp	B	Fv	S-Fp	B	Fv	S-Fp		
Number of relevés		3470	5016	1472	3470	5016	1472	3470	5016	1472		
Knautia drymeia	167	17	---	---	5	1	1	0.12	0.00	0.00		
Trifolium alpestre	734	16	---	---	12	6	1	0.42	0.24	0.03		
Peucedanum oreoselinum	600	16	---	---	12	3	5	0.87	0.14	0.10		
Trifolium repens	594	16	---	---	10	5	1	0.33	0.26	0.00		
Inula salicina	264	16	---	---	6	1	1	0.41	0.03	0.10	B (D)	C of Cirsio-Brachypodion in Mucina & Kolbek (1993) and Borhidi (1996), but also frequent in Molinion
Viola canina	191	16	---	---	5	1	1	0.10	0.01	0.00		
Aster amellus	645	15	---	---	11	4	3	0.49	0.14	0.08	B (D)	also D in Hegedüšová Vantarová & Škodová (2014)
Gymnadenia conopsea	223	15	---	---	6	1	2	0.11	0.00	0.03	B	also C in Mucina & Kolbek (1993) and Dengler (2003)
Pastinaca sativa	209	15	---	---	5	1	0	0.12	0.02	0.00		
Campanula persicifolia	283	14	---	---	7	1	2	0.14	0.01	0.04		
Vicia tenuifolia	233	14	---	---	5	1	0	0.47	0.05	0.00		
Fragaria vesca	496	13	---	---	9	2	4	0.37	0.07	0.07		
Linum flavum	396	13	---	---	7	2	2	0.27	0.07	0.06	B	C of Cirsio-Brachypodion in Mucina & Kolbek (1993)
Knautia kitaibelii	276	13	---	---	6	1	3	0.17	0.00	0.07		
Silene nutans	389	12	---	---	8	1	4	0.15	0.07	0.08		
Inula hirta	385	12	---	---	7	2	1	0.26	0.09	0.03		
Melampyrum arvense	361	11	---	---	6	3	1	0.23	0.09	0.01		
Galium boreale agg.	243	11	---	---	5	1	1	0.17	0.06	0.02		
Elytrigia intermedia	1514	---	23	---	12	22	1	0.66	2.29	0.02		
Convolvulus arvensis	1374	---	22	---	11	20	1	0.27	0.48	0.00		
Medicago falcata	3285	18	21	---	38	39	2	1.78	2.27	0.04		
Potentilla incana agg.	3363	---	21	10	12	47	39	0.50	3.16	1.96		
Elytrigia repens	766	---	19	---	5	12	1	0.19	1.10	0.00		ruderal species, can also have higher cover in disturbed semi-dry grasslands
Astragalus austriacus	515	---	19	---	2	9	1	0.04	0.37	0.02	Fv	also C in Mucina & Kolbek (1993) and Borhidi (1996)
Achillea nobilis	382	---	19	---	1	7	1	0.02	0.28	0.01	Fv	also C in Mucina & Kolbek (1993) and Hegedüšová Vantarová & Škodová (2014)
Berteroa incana	356	---	19	---	1	7	1	0.01	0.19	0.01	Fv (D)	phi threshold only missed by 0.01
Chondrilla juncea	324	---	19	---	1	6	1	0.01	0.16	0.01	Fv	also C in Mucina & Kolbek (1993)
Echium vulgare	1322	---	18	---	7	19	7	0.14	0.42	0.14		
Bromus inermis	659	---	18	---	4	10	0	0.23	1.71	0.00		
Carex praecox	427	---	18	---	1	7	1	0.06	1.27	0.01	Fv (D)	species of sandy grasslands, practically absent in Brometalia and Stipo-Festucetalia
Taraxacum serotinum	306	---	18	---	1	6	1	0.01	0.13	0.00	Fv	also C in Mucina & Kolbek (1993)
Veronica prostrata	547	---	17	---	2	9	1	0.04	0.20	0.02	Fv	also C in Hegedüšová Vantarová & Škodová (2014)
Rumex acetosella	501	---	17	---	1	9	1	0.03	0.33	0.04		
Tragopogon dubius	327	---	17	---	1	6	1	0.02	0.11	0.00		
Verbascum lychnitis	657	---	16	---	2	11	4	0.05	0.25	0.07		
Viola ambigua	485	---	16	---	3	8	1	0.04	0.20	0.00	Fv	also C in Mucina & Kolbek (1993)
Alyssum alyssoides	467	---	16	---	1	8	2	0.02	0.18	0.04		
Iris pumila	369	---	16	---	1	7	1	0.01	0.23	0.05	Fv	also C in Mucina & Kolbek (1993)
Linum austriacum	316	---	16	---	1	6	1	0.02	0.15	0.00	Fv	also C in Mucina & Kolbek (1993)
Artemisia absinthium	298	---	16	---	1	5	0	0.02	0.18	0.00		
Linaria genistifolia	592	---	15	---	1	10	5	0.01	0.19	0.09	Fv	also C in Hegedüšová Vantarová & Škodová (2014)
Helichrysum arenarium	338	---	15	---	1	6	1	0.02	0.23	0.01		
Asparagus officinalis	304	---	15	---	1	5	1	0.02	0.15	0.00		
Vinca herbacea	302	---	15	---	1	5	1	0.01	0.17	0.01		
Pilosella echioides	245	---	15	---	1	5	1	0.00	0.11	0.01		
Cytisus austriacus	677	---	14	---	5	10	1	0.26	0.82	0.01	Fv	also C in Mucina & Kolbek (1993)
Medicago minima	340	---	14	---	1	6	1	0.02	0.14	0.02		
Melica transsilvanica	342	---	13	---	1	6	1	0.02	0.38	0.07	Fv	also C in Hegedüšová Vantarová & Škodová (2014)
Pulsatilla pratensis	296	---	12	---	2	5	1	0.05	0.25	0.00		
Melilotus officinalis	328	---	11	---	2	5	1	0.06	0.13	0.00		
Chrysopogon gryllus	413	---	10	---	1	6	3	0.22	1.19	0.24		
Stipa joannis	427	---	9	---	2	6	3	0.20	1.26	0.43		
Astragalus monspessulanus	317	---	9	---	2	5	1	0.04	0.10	0.03		
Helianthemum nummularium	2006	3	---	20	27	11	37	1.15	0.31	0.84		extremely low constancy ratio
Cyanus triumfettii	405	---	---	19	4	2	13	0.07	0.04	0.26	S-Fp (D)	phi threshold only missed by 0.01
Bromus pannonicus	208	---	---	19	1	1	8	0.16	0.26	1.68	S-Fp	C for Bromo-Festucion pallentis in Borhidi (1996)
Paronychia cephalotes	99	---	---	19	0	1	6	0.00	0.01	0.09	S-Fp	C for Bromo-Festucion pallentis in Mucina & Kolbek (1993)

	total no. of occurrences	Phi value x 100 (orange: phi >= 0.2)			Constancy in % (green: const. ratio >= 2)			Average cover in % (blue: cover ratio >= 2)			Diagnostic value	Comment
Phytosociological order		B	Fv	S-Fp	B	Fv	S-Fp	B	Fv	S-Fp		
Number of relevés		3470	5016	1472	3470	5016	1472	3470	5016	1472		
Helictotrichon decorum	93	---	---	19	1	1	6	0.00	0.00	0.50	S-Fp (D)	phi threshold only missed by 0.01
Primula auricula	82	---	---	19	0	0	6	0.00	0.00	0.21	S-Fp (D)	also D in Mucina & Kolbek (1993) and Borhidi (1996)
Minuartia verna agg.	173	---	---	18	1	1	7	0.00	0.02	0.12		
Calamagrostis varia	115	---	---	18	1	1	6	0.06	0.00	0.88		
Allium ericetorum	77	---	---	18	1	1	5	0.00	0.00	0.13		
Festuca tatrae	72	---	---	18	0	0	5	0.00	0.00	0.36		
Asperula tinctoria	251	---	---	17	2	1	9	0.05	0.01	0.19		
Epipactis atrorubens	80	---	---	16	1	1	5	0.01	0.00	0.08		
Laserpitium latifolium	199	---	---	14	3	1	7	0.10	0.00	0.24		
Saxifraga tridactylites	188	---	---	14	1	2	6	0.00	0.04	0.08		
Digitalis grandiflora	135	---	---	14	1	1	5	0.03	0.01	0.11		
Pulsatilla vulgaris	495	---	---	11	5	4	10	0.20	0.12	0.28	S-Fp	also C in Mucina & Kolbek (1993)
Scabiosa canescens	275	---	---	8	2	2	6	0.06	0.06	0.14	S-Fp	C for Bromo-Festucion pallentis in Mucina & Kolbek (1993)

3. Species not reaching the threshold of any fidelity measure

	total no. of occurrences	Phi B	Phi Fv	Phi S-Fp	Const. B	Const. Fv	Const. S-Fp	Cover B	Cover Fv	Cover S-Fp
Euphorbia cyparissias	4798	---	---	---	49	48	47	1.55	1.41	1.01
Teucrium chamaedrys	4217	---	11	---	40	47	33	2.04	3.72	1.05
Asperula cynanchica	3716	---	5	---	36	40	34	0.85	1.14	0.73
Sanguisorba minor	2943	10	---	9	39	20	39	1.11	0.54	0.89
Hypericum perforatum	2608	15	10	---	31	28	7	0.68	0.69	0.12
Scabiosa ochroleuca	2484	10	---	---	29	24	16	0.64	0.57	0.32
Stachys recta	2431	---	12	---	18	30	21	0.42	1.09	0.39
Dianthus carthusianorum agg.	2246	12	---	---	29	20	17	0.76	0.54	0.33
Dorycnium pentaphyllum agg.	1845	13	---	---	25	15	15	1.76	0.66	0.47
Anthyllis vulneraria	1755	13	---	11	29	7	28	1.07	0.20	0.68
Pilosella officinarum	1746	---	12	---	17	21	6	0.49	0.88	0.13
Salvia verticillata	1504	18	---	---	22	13	6	1.15	0.74	0.14
Phleum phleoides	1376	---	12	---	13	17	4	0.51	0.85	0.09
Veronica spicata	1362	---	12	---	10	17	8	0.23	0.52	0.16
Clinopodium acinos	1351	---	11	6	5	19	16	0.12	0.42	0.26
Campanula sibirica	1345	---	3	11	7	16	20	0.17	0.42	0.37
Medicago lupulina	1302	19	---	---	20	12	1	0.53	0.35	0.03
Inula ensifolia	1267	---	---	16	14	8	23	2.24	0.49	1.15
Thesium linophyllon	1242	16	---	---	21	7	12	0.71	0.16	0.22
Arenaria serpyllifolia agg.	1194	---	11	7	4	17	15	0.10	0.42	0.21
Bupleurum falcatum	1121	8	---	6	16	6	15	0.45	0.22	0.36
Pilosella bauhini	1117	6	---	---	14	9	12	0.33	0.19	0.22
Galium glaucum	1115	---	6	---	8	13	10	0.19	0.34	0.21
Thalictrum minus	1079	---	2	---	9	12	11	0.25	0.44	0.25
Adonis vernalis	990	---	13	---	9	13	2	0.31	0.61	0.02
Senecio jacobaea	916	---	12	---	9	12	1	0.15	0.24	0.02
Allium flavum	842	---	3	18	1	11	17	0.01	0.20	0.31
Veronica austriaca	832	6	---	---	10	8	6	0.27	0.30	0.16
Sedum sexangulare	829	---	7	---	5	11	7	0.12	0.46	0.19
Euphorbia seguieriana	829	---	10	7	1	12	11	0.03	0.52	0.15
Silene otites agg.	818	---	6	12	2	11	14	0.04	0.26	0.22
Jurinea mollis	802	---	7	---	4	11	9	0.09	0.36	0.15
Sedum acre	787	---	14	---	1	12	8	0.03	0.54	0.24
Origanum vulgare	717	11	---	---	11	5	6	0.42	0.20	0.14
Genista tinctoria	705	12	---	---	10	6	2	0.25	0.32	0.05
Geranium sanguineum	699	9	---	1	11	4	8	0.92	0.27	0.31
Arabis hirsuta agg.	689	7	---	5	11	4	10	0.19	0.07	0.16
Trifolium campestre	680	8	8	---	8	8	1	0.22	0.27	0.00
Galatella linosyris	671	---	7	---	7	8	3	0.19	0.34	0.05
Picris hieracioides	640	15	---	---	10	6	1	0.21	0.10	0.00
Poa compressa	636	---	9	---	6	8	2	0.18	0.42	0.06
Cichorium intybus	597	9	7	---	7	7	1	0.15	0.22	0.00

	total no. of occurrences	Phi value x 100 (orange: phi >= 0.2)			Constancy in % (green: const. ratio >= 2)			Average cover in % (blue: cover ratio >= 2)			Diagnostic value	Comment
Phytosociological order		B	Fv	S-Fp	B	Fv	S-Fp	B	Fv	S-Fp		
Number of relevés		3470	5016	1472	3470	5016	1472	3470	5016	1472		
Calamagrostis epigejos	588	13	---	---	9	6	1	0.57	0.50	0.00		
Poa bulbosa	552	---	15	---	1	9	5	0.01	0.60	0.06		
Stipa pulcherrima	551	---	12	---	1	9	5	0.18	2.03	0.59		
Euphorbia esula	544	14	---	---	9	5	1	0.16	0.11	0.01		
Cytisus nigricans	543	5	---	2	8	3	7	0.49	0.18	0.16		
Hylotelephium maximum agg.	527	---	4	8	2	7	8	0.03	0.15	0.17		
Verbascum chaixii	517	---	2	---	5	6	5	0.10	0.12	0.09		
Hippocrepis comosa	490	6	---	10	8	1	10	0.29	0.03	0.25		
Rostraria cristata	450	---	3	---	4	5	4	0.06	0.23	0.07		
Helictochloa pratensis	426	---	6	---	5	5	1	0.15	0.42	0.01		
Seseli hippomarathrum	400	---	1	9	2	5	7	0.03	0.14	0.17		
Microthlaspi perfoliatum	390	---	7	---	2	5	3	0.04	0.10	0.05		
Campanula rapunculoides	386	10	---	2	7	1	5	0.21	0.03	0.11		
Cytisus hirsutus	373	3	---	9	6	1	7	0.21	0.05	0.24		
Anthemis tinctoria	367	---	11	---	3	5	1	0.06	0.13	0.01		
Muscari comosum	365	---	9	---	3	5	0	0.06	0.10	0.00		
Cerastium pumilum	354	---	---	11	1	4	7	0.02	0.10	0.03		
Festuca ovina agg.	353	8	---	---	5	3	1	0.50	0.57	0.09		
Hieracium umbellatum	337	9	---	---	5	3	1	0.10	0.12	0.02		
Stellaria graminea	333	9	---	---	5	3	0	0.11	0.15	0.00		
Cytisus albus	315	9	---	---	5	3	1	0.35	0.16	0.02		
Erophila verna	303	---	9	---	1	5	3	0.01	0.17	0.04		
Seseli libanotis	283	---	---	8	4	2	6	0.19	0.06	0.18		
**4. Species with constancy < 5% in all three orders**												
Sempervivum marmoreum	78	---	---	14	0	1	4	0.00	0.03	0.11		
Aethionema saxatile	63	---	---	15	1	1	4	0.00	0.00	0.02		
Coronilla vaginalis	65	---	---	15	1	0	4	0.02	0.00	0.13		
Taraxacum sect. Erythrosperma	212	---	3	6	1	3	4	0.01	0.07	0.03		
Leontodon crispus	293	---	3	3	1	4	4	0.03	0.06	0.07		
Carex digitata	77	---	---	14	1	1	4	0.01	0.00	0.08		
Viola jooi	68	---	---	15	1	1	4	0.00	0.00	0.08		
Globularia cordifolia	96	---	---	12	1	1	4	0.04	0.03	0.18		
Aster alpinus	60	---	---	15	1	1	4	0.00	0.00	0.14		
Bellidiastrum michelii	60	---	---	15	1	0	4	0.00	0.00	0.10		
Euphrasia salisburgensis	61	---	---	15	1	1	4	0.00	0.00	0.10		
Campanula carpatica	59	---	---	16	0	0	4	0.00	0.00	0.10		
Carex liparocarpos	167	---	---	9	1	2	4	0.01	0.12	0.07		
Aurinia saxatilis	88	---	---	13	0	1	4	0.00	0.08	0.33		
Viola collina	109	---	---	7	1	1	3	0.04	0.02	0.03		
Onosma visianii	83	---	---	9	0	1	3	0.00	0.02	0.04		
Plantago argentea	134	---	---	6	1	1	3	0.01	0.03	0.06		
Medicago prostrata	178	---	---	3	1	2	3	0.04	0.04	0.05		
Trinia glauca	128	---	---	7	1	1	3	0.02	0.03	0.04		
Poa nemoralis	76	---	---	11	1	1	3	0.01	0.02	0.30		
Asplenium septentrionale	96	---	---	11	0	1	3	0.00	0.02	0.13		
Hieracium murorum	62	---	---	13	1	1	3	0.01	0.00	0.06		
Seseli gracile	62	---	---	12	1	1	3	0.00	0.01	0.06		
Selinum silaifolium	45	---	---	14	1	1	3	0.00	0.00	0.06		
Galium lucidum	134	---	---	7	1	1	3	0.02	0.05	0.07		
Hippocrepis emerus	75	---	---	13	0	1	3	0.00	0.01	0.04		
Sesleria sadlerana	48	---	---	14	1	1	3	0.03	0.01	1.61		
Carex ornithopoda	100	---	---	10	1	1	3	0.04	0.00	0.07		
Scabiosa columbaria	130	2	---	5	2	1	3	0.08	0.01	0.05		
Muscari neglectum	220	---	3	4	1	3	3	0.02	0.10	0.01		
Pistorinia hispanica	67	---	---	11	1	1	3	0.00	0.02	0.06		
Aconitum anthora	70	---	---	11	1	1	3	0.00	0.01	0.06		

	total no. of occurrences	Phi value x 100 (orange: phi >= 0.2)			Constancy in % (green: const. ratio >= 2)			Average cover in % (blue: cover ratio >= 2)			Diagnostic value	Comment
Phytosociological order		B	Fv	S-Fp	B	Fv	S-Fp	B	Fv	S-Fp		
Number of relevés		3470	5016	1472	3470	5016	1472	3470	5016	1472		
Hieracium bifidum	53	---	---	15	1	1	3	0.00	0.00	0.07		
Euphorbia epithymoides	121	---	---	7	1	1	3	0.02	0.01	0.06		
Polypodium vulgare	57	---	---	14	0	1	3	0.00	0.00	0.15		
Dianthus spiculifolius	48	---	---	15	0	0	3	0.00	0.00	0.08		
Lactuca perennis	120	---	1	7	1	2	3	0.00	0.03	0.05		
Geranium robertianum	86	---	---	12	1	1	3	0.01	0.01	0.08		
Asplenium viride	44	---	---	13	1	1	3	0.00	0.01	0.06		
Sesleria heuflerana	132	---	---	5	2	1	3	0.88	0.32	0.92		
Cephalaria radiata	91	---	---	4	1	1	2	0.02	0.01	0.04		
Gentiana clusii	28	---	---	11	1	0	2	0.00	0.00	0.07		
Minuartia rubra	123	---	---	5	1	1	2	0.04	0.02	0.04		
Spiraea media	53	---	---	10	1	1	2	0.00	0.01	0.15		
Mercurialis perennis	33	---	---	9	1	1	2	0.00	0.00	0.03		
Euphrasia rostkoviana agg.	181	9	---	---	4	1	2	0.09	0.00	0.05		
Seseli rigidum	31	---	---	11	0	1	2	0.00	0.00	0.04		
Asperula capitata	23	---	---	10	0	0	2	0.00	0.00	0.03		
Euphrasia stricta agg.	351	---	4	---	4	4	2	0.08	0.11	0.05		
Arabis auriculata	99	---	1	4	1	1	2	0.01	0.02	0.03		
Cystopteris fragilis	42	---	---	11	0	1	2	0.00	0.00	0.05		
Sedum hispanicum	45	---	---	8	0	1	2	0.00	0.01	0.03		
Lilium martagon	66	1	---	6	1	0	2	0.02	0.00	0.03		
Festuca stricta subsp. stricta	58	---	---	7	1	1	2	0.00	0.06	0.15		
Convallaria majalis	70	---	---	8	1	1	2	0.06	0.00	0.26		
Gymnocarpium robertianum	32	---	---	11	0	1	2	0.00	0.00	0.05		
Thalictrum foetidum	40	---	---	11	1	1	2	0.00	0.00	0.06		
Helianthemum oelandicum	28	---	---	11	1	0	2	0.00	0.00	0.08		
Campanula cochleariifolia	32	---	---	12	0	0	2	0.00	0.00	0.05		
Artemisia alba	88	---	---	8	0	1	2	0.00	0.12	0.13		
Genista januensis	108	2	---	2	2	1	2	0.03	0.02	0.07		
Viola rupestris	191	4	---	---	3	1	2	0.06	0.03	0.04		
Podospermum purpureum	173	5	---	---	3	1	2	0.06	0.02	0.02		
Seseli longifolium	39	---	---	10	1	1	2	0.00	0.01	0.04		
Ajuga genevensis	319	---	3	---	3	4	2	0.06	0.09	0.03		
Trisetum alpestre	39	---	---	13	0	1	2	0.00	0.00	0.08		
Valeriana officinalis	197	5	---	---	3	1	2	0.06	0.02	0.05		
Pimpinella major	97	1	---	6	1	1	2	0.03	0.00	0.06		
Tephroseris integrifolia	92	2	---	5	1	1	2	0.04	0.00	0.05		
Platanthera bifolia	64	---	---	9	1	1	2	0.01	0.00	0.04		
Rhamnus saxatilis	49	---	---	11	1	1	2	0.00	0.00	0.05		
Holosteum umbellatum	187	---	8	---	1	3	2	0.00	0.07	0.02		
Cerastium brachypetalum	212	---	---	---	2	2	2	0.04	0.06	0.05		
Rosa spinosissima	89	---	---	5	1	1	2	0.03	0.02	0.05		
Seseli pallasii	242	---	6	---	1	3	2	0.02	0.14	0.04		
Melica nutans	54	---	---	6	1	1	2	0.02	0.00	0.04		
Carex alba	57	---	---	8	1	1	2	0.07	0.00	0.09		
Asplenium ceterach	69	---	---	8	1	1	2	0.00	0.01	0.07		
Allium sphaerocephalon	197	---	7	---	1	3	2	0.01	0.08	0.03		
Odontites luteus	215	---	4	---	1	3	2	0.07	0.07	0.03		
Cirsium erisithales	50	---	---	9	1	0	2	0.02	0.00	0.05		
Festuca arvernensis	35	---	---	10	1	1	2	0.00	0.02	0.22		
Cerastium arvense	280	6	---	---	4	2	2	0.12	0.06	0.11		
Viola tricolor	145	---	2	5	1	2	2	0.01	0.05	0.05		
Daphne cneorum	47	---	---	10	1	1	2	0.01	0.02	0.07		
Onosma arenaria	59	---	3	---	0	1	1	0.00	0.01	0.02		
Selaginella helvetica	35	---	---	6	1	1	1	0.00	0.01	0.05		
Dianthus serotinus	25	---	---	8	0	1	1	0.00	0.00	0.04		
Inula conyzae	76	---	---	---	1	1	1	0.02	0.01	0.01		
Allium moschatum	21	---	---	10	0	0	1	0.00	0.00	0.01		

	total no. of occurrences	Phi value x 100 (orange: phi >= 0.2)			Constancy in % (green: const. ratio >= 2)			Average cover in % (blue: cover ratio >= 2)			Diagnostic value	Comment
Phytosociological order		B	Fv	S-Fp	B	Fv	S-Fp	B	Fv	S-Fp		
Number of relevés		3470	5016	1472	3470	5016	1472	3470	5016	1472		
Linum perenne	58	---	2	---	1	1	1	0.01	0.05	0.01		
Potentilla inclinata	173	---	11	---	1	3	1	0.02	0.14	0.01		
Sedum rupestre	64	---	3	---	1	1	1	0.00	0.02	0.02		
Hieracium glaucum	11	---	---	7	0	1	1	0.00	0.00	0.01		
Festuca psammophila	12	---	---	6	0	1	1	0.00	0.00	0.03		
Valeriana tripteris	25	---	---	10	1	1	1	0.00	0.00	0.03		
Viscaria vulgaris	323	---	6	---	3	4	1	0.08	0.16	0.03		
Cytisus ratisbonensis	191	6	---	---	3	1	1	0.13	0.04	0.03		
Arabidopsis thaliana	152	---	6	---	1	2	1	0.01	0.05	0.02		
Carduus collinus	93	---	4	---	1	1	1	0.01	0.03	0.02		
Melampyrum nemorosum	114	6	---	---	2	1	1	0.26	0.02	0.03		
Aquilegia vulgaris	77	7	---	---	2	1	1	0.05	0.00	0.02		
Odontites vulgaris agg.	147	---	4	---	1	2	1	0.02	0.04	0.01		
Crepis jacquinii	20	---	---	10	0	0	1	0.00	0.00	0.04		
Noccaea montana	15	---	---	6	0	1	1	0.00	0.00	0.03		
Carduus candicans	36	---	---	6	0	1	1	0.00	0.01	0.02		
Gypsophila fastigiata	48	---	---	---	1	1	1	0.07	0.01	0.01		
Ononis pusilla	50	---	---	5	1	1	1	0.00	0.01	0.01		
Ophrys insectifera	27	---	---	6	1	0	1	0.01	0.00	0.02		
Taraxacum sect. Erythrocarpa	23	---	---	8	1	1	1	0.00	0.00	0.02		
Iris humilis	33	---	---	4	1	1	1	0.00	0.01	0.01		
Thymus alpestris	23	---	---	5	1	1	1	0.01	0.00	0.02		
Lactuca muralis	28	---	---	8	1	1	1	0.00	0.00	0.02		
Festuca pseudodalmatica	236	---	11	---	1	4	1	0.01	1.31	0.12		
Potentilla recta	326	---	6	---	3	4	1	0.08	0.08	0.01		
Cyanus montanus	15	---	---	7	1	1	1	0.00	0.00	0.03		
Hieracium villosum	15	---	---	6	1	1	1	0.00	0.00	0.01		
Moehringia muscosa	19	---	---	9	0	0	1	0.00	0.00	0.03		
Centaurea atropupurea	54	---	---	5	1	1	1	0.00	0.02	0.03		
Cyanus pinnatifidus	22	---	---	10	1	0	1	0.00	0.00	0.03		
Veronica praecox	171	---	9	---	1	3	1	0.00	0.15	0.00		
Gentianella lutescens	27	---	---	6	1	0	1	0.01	0.00	0.02		
Seseli austriacum	12	---	---	7	0	0	1	0.00	0.00	0.01		
Pilosella pavichii	28	---	---	8	0	1	1	0.00	0.00	0.03		
Laserpitium siler	31	1	---	3	1	0	1	0.05	0.00	0.07		
Spiraea chamaedryfolia	10	---	---	7	0	0	1	0.00	0.00	0.02		
Solidago virgaurea	159	9	---	---	3	1	1	0.07	0.02	0.03		
Reseda lutea	257	---	10	---	1	4	1	0.02	0.08	0.02		
Myosotis stricta	163	---	11	---	1	3	1	0.00	0.06	0.01		
Rhinanthus alectorolophus	38	4	---	---	1	1	1	0.04	0.00	0.03		
Veronica arvensis	296	---	7	---	3	4	1	0.05	0.08	0.01		
Fallopia convolvulus	119	---	6	---	1	2	1	0.01	0.03	0.01		
Rhinanthus angustifolius	177	11	---	---	4	1	1	0.21	0.02	0.02		
Sesleria tenuifolia subsp. kalnikensis	19	---	---	7	1	1	1	0.01	0.01	0.38		
Orobanche gracilis	105	8	---	---	2	1	1	0.04	0.01	0.01		
Melampyrum sylvaticum	25	---	---	7	1	0	1	0.02	0.00	0.08		
Pilosella cymosa	181	---	6	---	1	3	1	0.03	0.14	0.06		
Centaurea reichenbachii	14	---	---	5	0	1	1	0.00	0.00	0.01		
Sempervivum montanum	20	---	---	4	1	1	1	0.00	0.01	0.06		
Koeleria glauca	44	---	2	---	1	1	1	0.00	0.02	0.01		
Euphorbia amygdaloides	25	---	---	5	1	1	1	0.00	0.00	0.01		
Convolvulus cantabrica	123	---	6	---	1	2	1	0.00	0.08	0.04		
Orobanche caryophyllacea	52	---	3	---	1	1	1	0.00	0.01	0.01		
Viola kitaibeliana	72	---	6	---	1	1	1	0.00	0.02	0.00		
Melittis melissophyllum	18	---	---	4	1	0	1	0.01	0.00	0.01		
Euphorbia saxatilis	10	---	---	7	0	0	1	0.00	0.00	0.01		
Aconitum variegatum	20	---	---	9	1	0	1	0.00	0.00	0.04		
Rubus saxatilis	31	---	---	8	1	0	1	0.01	0.00	0.04		

	total no. of occurrences	Phi value x 100 (orange: phi >= 0.2)			Constancy in % (green: const. ratio >= 2)			Average cover in % (blue: cover ratio >= 2)			Diagnostic value	Comment
Phytosociological order		B	Fv	S-Fp	B	Fv	S-Fp	B	Fv	S-Fp		
Number of relevés		3470	5016	1472	3470	5016	1472	3470	5016	1472		
Coronilla coronata	31	---	---	8	1	1	1	0.02	0.00	0.06		
Carex sempervirens	20	---	---	9	1	0	1	0.00	0.00	0.05		
Gypsophila collina	78	---	2	4	1	1	1	0.00	0.03	0.04		
Cyanus mollis	19	---	---	8	1	0	1	0.00	0.00	0.05		
Echinops ritro	33	---	---	7	1	1	1	0.00	0.01	0.02		
Pedicularis comosa	33	---	---	5	1	1	1	0.01	0.00	0.02		
Hepatica nobilis	15	---	---	6	1	0	1	0.00	0.00	0.02		
Petrorhagia saxifraga	224	---	8	---	1	3	1	0.02	0.11	0.01		
Galium schultesii	26	---	---	4	1	1	1	0.01	0.00	0.02		
Asarum europaeum	38	4	---	---	1	1	1	0.02	0.00	0.01		
Pulsatilla montana	114	2	---	---	1	1	1	0.03	0.02	0.01		
Potentilla pusilla agg.	197	6	---	---	3	2	1	0.10	0.11	0.03		
Astrantia major	57	7	---	---	1	0	1	0.06	0.00	0.02		
Hieracium sabaudum	76	4	---	---	1	1	1	0.02	0.01	0.01		
Cyanus dominii	10	---	---	7	0	0	1	0.00	0.00	0.04		
Senecio squalidus	20	---	---	7	1	1	1	0.00	0.00	0.02		
Arabis turrita	25	---	---	8	1	1	1	0.00	0.00	0.02		
Silene italica	28	---	---	5	1	1	1	0.01	0.00	0.02		
Dianthus petraeus	20	---	---	9	0	1	1	0.00	0.00	0.03		
Arabidopsis petraea	19	---	---	9	0	0	1	0.00	0.00	0.03		
Veronica fruticans	24	---	---	9	1	1	1	0.00	0.00	0.05		
Saponaria bellidifolia	24	---	---	9	0	1	1	0.00	0.00	0.03		
Carduus nutans	170	---	9	---	1	3	1	0.01	0.07	0.00		
Primula elatior	54	1	---	2	1	1	1	0.02	0.01	0.02		
Erysimum diffusum	222	---	10	---	1	4	1	0.00	0.09	0.02		
Cerastium semidecandrum	221	---	11	---	1	4	1	0.00	0.19	0.02		
Silene nemoralis	39	---	---	5	1	1	1	0.01	0.00	0.02		
Isatis tinctoria	53	---	---	7	1	1	1	0.00	0.01	0.03		
Astragalus vesicarius	55	---	3	---	1	1	1	0.00	0.02	0.05		
Heracleum sphondylium	146	11	---	---	4	1	1	0.07	0.00	0.02		
Antennaria dioica	64	6	---	---	2	1	1	0.05	0.00	0.01		
Cuscuta epithymum	191	5	---	---	3	2	1	0.06	0.04	0.01		
Dictamnus albus	241	---	3	---	2	3	1	0.08	0.07	0.02		
Draba aizoides	19	---	---	9	0	1	1	0.00	0.00	0.03		
Alyssum tortuosum	26	---	---	5	0	1	1	0.00	0.01	0.02		
Tofieldia calyculata	26	---	---	7	1	0	1	0.01	0.00	0.03		
Carex firma	14	---	---	8	0	0	1	0.00	0.00	0.06		
Campanula trachelium	57	2	---	4	1	1	1	0.02	0.00	0.03		
Potentilla thuringiaca	36	---	---	7	1	1	1	0.01	0.00	0.03		
Ranunculus zapalowiczii	34	6	---	---	1	1	0	0.06	0.00	0.00		
Pedicularis verticillata	1	---	---	---	0	0	1	0.00	0.00	0.00		
Peucedanum altissimum	2	---	---	---	1	0	0	0.00	0.00	0.00		
Peucedanum austriacum	4	---	---	---	1	0	1	0.00	0.00	0.00		
Pedicularis elongata subsp. julica	1	---	---	---	1	0	0	0.00	0.00	0.00		
Paris quadrifolia	2	---	---	---	1	0	1	0.00	0.00	0.00		
Onosma simplicissima	4	---	---	---	0	1	0	0.00	0.02	0.00		
Onosma pseudoarenaria	11	---	4	---	0	1	0	0.00	0.00	0.00		
Onobrychis gracilis	5	---	3	---	0	1	0	0.00	0.00	0.00		
Asplenium x alternifolium	6	---	---	4	0	1	1	0.00	0.00	0.01		
Astragalus pseudotataricus	3	---	---	---	0	1	0	0.00	0.00	0.00		
Dianthus monspessulanus	23	6	---	---	1	0	1	0.01	0.00	0.03		
Pulmonaria angustifolia	36	7	---	---	1	1	0	0.02	0.00	0.00		
Trifolium pannonicum	29	6	---	---	1	1	0	0.02	0.00	0.00		
Pteridium aquilinum	26	4	---	---	1	1	1	0.05	0.00	0.03		
Pilosella caespitosa	41	2	---	---	1	1	1	0.02	0.01	0.01		
Parnassia palustris	9	---	---	3	1	0	1	0.00	0.00	0.01		
Papaver rhoeas	20	---	4	---	1	1	0	0.00	0.01	0.00		
Parietaria officinalis	2	---	---	---	1	0	0	0.00	0.00	0.00		

	total no. of occurrences	Phi value x 100 (orange: phi >= 0.2)			Constancy in % (green: const. ratio >= 2)			Average cover in % (blue: cover ratio >= 2)			Diagnostic value	Comment
Phytosociological order		B	Fv	S-Fp	B	Fv	S-Fp	B	Fv	S-Fp		
Number of relevés		3470	5016	1472	3470	5016	1472	3470	5016	1472		
Potentilla collina agg.	48	7	---	---	1	1	0	0.03	0.00	0.00		
Potentilla rupestris	10	3	---	---	1	1	0	0.01	0.00	0.00		
Gentianella ciliata	48	8	---	---	1	0	1	0.03	0.00	0.00		
Anacamptis pyramidalis	87	8	---	---	2	1	1	0.04	0.00	0.01		
Viola odorata	66	9	---	---	2	1	1	0.02	0.00	0.00		
Neotinea tridentata	99	10	---	---	2	1	1	0.05	0.00	0.00		
Neotinea ustulata	107	10	---	---	2	1	1	0.05	0.01	0.00		
Primula acaulis	72	10	---	---	2	0	1	0.04	0.00	0.00		
Carum carvi	88	10	---	---	2	1	1	0.04	0.01	0.00		
Linum viscosum	25	7	---	---	1	1	0	0.01	0.00	0.00		
Ranunculus auricomus agg.	58	10	---	---	2	1	0	0.03	0.00	0.00		
Molinia caerulea agg.	78	10	---	---	2	1	1	0.15	0.01	0.01		
Cruciata laevipes	51	7	---	---	1	1	1	0.02	0.01	0.00		
Astragalus danicus	76	7	---	---	2	1	1	0.06	0.02	0.00		
Orchis militaris	105	10	---	---	2	1	1	0.05	0.01	0.00		
Cynosurus cristatus	152	16	---	---	4	1	0	0.15	0.02	0.00		
Holcus lanatus	160	16	---	---	4	1	0	0.11	0.00	0.00		
Allium scorodoprasum	87	8	---	---	2	1	1	0.03	0.01	0.01		
Cirsium acaulon	75	11	---	---	2	0	1	0.06	0.00	0.00		
Cirsium eriophorum	72	10	---	---	2	1	0	0.03	0.00	0.00		
Melampyrum cristatum	87	6	---	---	2	1	1	0.05	0.01	0.01		
Clematis recta	87	10	---	---	2	1	1	0.07	0.00	0.01		
Arabis ciliata	3	2	---	---	1	0	0	0.00	0.00	0.00		
Pulmonaria dacica	1	---	---	---	0	1	0	0.00	0.00	0.00		
Pulmonaria officinalis agg.	13	2	---	---	1	1	1	0.00	0.00	0.00		
Arabidopsis petrogena	2	---	---	---	0	1	1	0.00	0.00	0.00		
Arctium lappa	1	---	---	---	0	1	0	0.00	0.00	0.00		
Arabis alpina	5	---	---	5	0	0	1	0.00	0.00	0.01		
Ranunculus flammula	1	---	---	---	1	0	0	0.00	0.00	0.00		
Ranunculus arvensis	1	---	---	---	1	0	0	0.00	0.00	0.00		
Pulsatilla taurica	1	---	---	---	1	0	0	0.00	0.00	0.00		
Ranunculus ficaria	6	---	---	---	1	1	0	0.00	0.00	0.00		
Potentilla chrysantha	4	---	---	---	1	1	0	0.00	0.00	0.00		
Arabis pauciflora	5	---	---	---	1	0	1	0.00	0.00	0.00		
Ranunculus lanuginosus	3	---	---	---	1	1	0	0.00	0.00	0.00		
Pulsatilla zimmermannii	16	4	---	---	1	1	0	0.01	0.01	0.00		
Ranunculus alpestris	2	---	---	3	0	0	1	0.00	0.00	0.01		
Pulicaria vulgaris	4	3	---	---	1	0	0	0.00	0.00	0.00		
Arnica montana	3	2	---	---	1	0	0	0.00	0.00	0.00		
Aristolochia pallida	4	---	---	---	1	1	0	0.00	0.00	0.00		
Potentilla patula	11	2	---	---	1	1	0	0.00	0.01	0.00		
Arenaria rigida	5	---	3	---	0	1	0	0.00	0.00	0.00		
Sanguisorba officinalis	108	10	---	---	3	1	1	0.09	0.01	0.01		
Thalictrum aquilegiifolium	26	3	---	---	1	1	1	0.01	0.00	0.01		
Thalictrum simplex	49	5	---	---	1	1	0	0.02	0.01	0.00		
Sesleria uliginosa	22	7	---	---	1	0	0	0.14	0.00	0.00		
Scabiosa cinerea subsp. hladnikiana	27	7	---	---	1	0	0	0.02	0.00	0.00		
Sonchus arvensis	48	5	---	---	1	1	0	0.02	0.01	0.00		
Orobanche lutea	40	5	---	---	1	1	0	0.01	0.00	0.00		
Plantago major	51	5	---	---	1	1	1	0.02	0.01	0.00		
Lathyrus niger	40	8	---	---	1	1	0	0.02	0.00	0.00		
Knautia illyrica	28	7	---	---	1	0	0	0.02	0.00	0.00		
Senecio doria	21	5	---	---	1	1	0	0.02	0.00	0.00		
Gentianella germanica	27	6	---	---	1	0	1	0.01	0.00	0.00		
Festuca arundinacea	40	5	---	---	1	1	0	0.03	0.02	0.00		
Euphorbia angulata	38	5	---	---	1	1	1	0.02	0.01	0.00		
Crepis praemorsa	55	8	---	---	1	1	1	0.03	0.00	0.00		
Genista germanica	39	6	---	---	1	1	1	0.02	0.00	0.00		

	total no. of occurrences	Phi value x 100 (orange: phi >= 0.2)			Constancy in % (green: const. ratio >= 2)			Average cover in % (blue: cover ratio >= 2)			Diagnostic value	Comment
Phytosociological order		B	Fv	S-Fp	B	Fv	S-Fp	B	Fv	S-Fp		
Number of relevés		3470	5016	1472	3470	5016	1472	3470	5016	1472		
Vicia sepium	58	8	---	---	1	1	1	0.03	0.00	0.00		
Ferulago sylvatica	48	5	---	---	1	1	1	0.02	0.01	0.00		
Erica carnea	32	6	---	---	1	0	1	0.07	0.00	0.01		
Deschampsia cespitosa	28	6	---	---	1	1	0	0.02	0.00	0.00		
Plantago holosteum	24	7	---	---	1	0	0	0.02	0.00	0.00		
Gentiana verna	67	11	---	---	2	0	0	0.04	0.00	0.00		
Gentiana cruciata	147	14	---	---	4	1	1	0.11	0.00	0.01		
Hypericum maculatum	74	10	---	---	2	1	1	0.05	0.00	0.00		
Polygala chamaebuxus	61	8	---	---	2	0	1	0.05	0.00	0.01		
Scabiosa triandra	155	17	---	---	4	1	0	0.10	0.00	0.00		
Carex pallescens	106	14	---	---	3	1	0	0.07	0.00	0.00		
Serratula tinctoria	118	13	---	---	3	1	1	0.08	0.00	0.01		
Lathyrus latifolius	151	15	---	---	4	1	0	0.08	0.01	0.00		
Genista sagittalis	129	15	---	---	4	1	0	0.18	0.00	0.00		
Ajuga reptans	129	12	---	---	3	1	1	0.07	0.01	0.01		
Symphytum tuberosum	36	6	---	---	1	0	1	0.02	0.00	0.00		
Succisa pratensis	30	7	---	---	1	1	0	0.02	0.00	0.00		
Tragopogon tommasinii	23	7	---	---	1	0	0	0.01	0.00	0.00		
Traunsteinera globosa	31	7	---	---	1	0	1	0.02	0.00	0.00		
Pulmonaria mollis	106	13	---	---	3	1	0	0.05	0.00	0.00		
Trifolium rubens	123	13	---	---	3	1	1	0.09	0.01	0.00		
Centaurea phrygia	129	13	---	---	3	1	1	0.09	0.00	0.01		
Allium carinatum	97	12	---	---	3	1	1	0.06	0.00	0.01		
Rhinanthus glacialis	107	14	---	---	3	0	0	0.26	0.00	0.00		
Rumex alpestris	4	3	---	---	1	0	0	0.00	0.00	0.00		
Rumex patientia	3	---	---	---	0	1	0	0.00	0.00	0.00		
Anthriscus sylvestris	19	5	---	---	1	1	0	0.01	0.00	0.00		
Rindera tetraspis	9	---	3	---	0	1	0	0.00	0.08	0.00		
Rorippa sylvestris	9	---	---	---	1	1	0	0.00	0.00	0.00		
Rorippa austriaca	2	---	---	---	0	1	0	0.00	0.00	0.00		
Orobanche teucrii	24	---	3	---	1	1	0	0.00	0.01	0.00		
Rumex crispus	21	3	---	---	1	1	0	0.01	0.00	0.00		
Rumex obtusifolius	3	---	---	---	1	1	0	0.00	0.00	0.00		
Anthyllis schiwereckii	9	---	---	---	1	1	0	0.00	0.00	0.00		
Rumex pulcher	4	---	---	---	1	1	0	0.00	0.00	0.00		
Polygonum lapathifolium	1	---	---	---	0	1	0	0.00	0.00	0.00		
Reseda phyteuma	5	---	---	4	0	1	1	0.00	0.00	0.00		
Aquilegia einseleana	1	---	---	---	1	0	0	0.00	0.00	0.00		
Apera spica-venti	16	---	4	---	1	1	0	0.00	0.01	0.00		
Ruscus aculeatus L.	2	---	---	---	0	1	0	0.00	0.00	0.00		
Aquilegia transsilvanica	1	---	---	---	0	1	0	0.00	0.00	0.00		
Rhaponticoides ruthenica	6	---	---	---	1	1	0	0.00	0.00	0.00		
Rorippa pyrenaica	7	4	---	---	1	0	0	0.00	0.00	0.00		
Rindera umbelata	10	---	4	---	0	1	0	0.00	0.00	0.00		
Ranunculus thora	1	---	---	---	1	0	0	0.00	0.00	0.00		
Rhinanthus alpinus	6	---	---	3	1	0	1	0.00	0.00	0.01		
Aposeris foetida	2	---	---	---	1	1	0	0.00	0.00	0.00		
Aphanes arvensis	2	---	---	---	0	1	0	0.00	0.00	0.00		
Saxifraga cuneifolia	4	---	---	4	0	0	1	0.00	0.00	0.01		
Saxifraga granulata	16	---	---	---	1	1	0	0.00	0.00	0.00		
Salvia scabiosifolia	1	---	---	---	1	0	0	0.00	0.00	0.00		
Saxifraga aizoides	3	---	---	4	0	0	1	0.00	0.00	0.00		
Anthemis austriaca	4	---	---	---	1	1	0	0.00	0.00	0.00		
Saxifraga adscendens	4	---	---	4	0	0	1	0.00	0.00	0.01		
Anthemis odontostephana	2	---	---	---	1	0	0	0.00	0.00	0.00		
Salvia glutinosa	12	---	---	---	1	1	1	0.01	0.00	0.01		
Prenanthes purpurea	4	---	---	4	0	0	1	0.00	0.00	0.01		
Angelica sylvestris	10	2	---	---	1	1	1	0.01	0.00	0.00		

	total no. of occurrences	Phi value x 100 (orange: phi >= 0.2)			Constancy in % (green: const. ratio >= 2)			Average cover in % (blue: cover ratio >= 2)			Diagnostic value	Comment
Phytosociological order		B	Fv	S-Fp	B	Fv	S-Fp	B	Fv	S-Fp		
Number of relevés		3470	5016	1472	3470	5016	1472	3470	5016	1472		
Angelica archangelica	1	---	---	---	0	1	0	0.00	0.00	0.00		
Salvia sclarea	1	---	---	---	0	1	0	0.00	0.00	0.00		
Rumex sanguineus	1	---	---	---	0	1	0	0.00	0.00	0.00		
Sagina nodosa	1	---	---	---	0	1	0	0.00	0.00	0.00		
Anthriscus caucalis	1	---	---	---	0	1	0	0.00	0.00	0.00		
Rumex confertus	14	---	4	---	0	1	0	0.00	0.01	0.00		
Rumex scutatus	2	---	---	---	1	0	1	0.00	0.00	0.00		
Sagina procumbens	1	---	---	---	0	1	0	0.00	0.00	0.00		
Salsola kali	2	---	---	---	0	1	0	0.00	0.00	0.00		
Salvia dumetorum	20	---	5	---	0	1	0	0.00	0.02	0.00		
Salix repens	9	3	---	---	1	1	0	0.01	0.00	0.00		
Anthericum liliago	8	3	---	---	1	1	0	0.00	0.00	0.00		
Anthriscus nitidus	1	---	---	---	0	1	0	0.00	0.00	0.00		
Anthriscus cerefolium	3	---	---	---	0	1	0	0.00	0.00	0.00		
Reseda luteola	6	---	3	---	0	1	0	0.00	0.00	0.00		
Lapsana communis	6	---	---	---	1	1	0	0.00	0.00	0.00		
Laser trilobum	1	---	---	---	1	0	0	0.00	0.00	0.00		
Lamium galeobdolon	8	---	---	3	1	1	1	0.00	0.00	0.01		
Calamagrostis canescens	1	---	---	---	1	0	0	0.00	0.00	0.00		
Lathyrus pallescens	13	4	---	---	1	1	0	0.01	0.00	0.00		
Lathyrus aphaca	3	---	---	---	1	1	0	0.00	0.00	0.00		
Lathyrus heterophyllus	1	---	---	---	0	1	0	0.00	0.00	0.00		
Lepidium ruderale	4	---	---	---	0	1	0	0.00	0.00	0.00		
Leontodon biscutellifolius	15	---	---	---	1	1	0	0.01	0.01	0.00		
Lamium album	2	---	---	---	0	1	0	0.00	0.00	0.00		
Lathyrus hirsutus	13	---	---	---	1	1	0	0.00	0.00	0.00		
Calamagrostis pseudophragmites	1	---	---	---	0	1	0	0.00	0.01	0.00		
Laphangium luteoalbum	1	---	---	---	1	0	0	0.00	0.00	0.00		
Lamium garganicum	1	---	---	---	0	0	1	0.00	0.00	0.00		
Lappula heteracantha	1	---	---	---	0	1	0	0.00	0.00	0.00		
Atriplex patula	2	---	---	---	1	1	0	0.00	0.00	0.00		
Lamium maculatum	6	---	---	4	1	1	1	0.00	0.00	0.01		
Lamium purpureum	20	---	3	---	1	1	0	0.00	0.01	0.00		
Bunias orientalis	7	---	---	---	1	1	0	0.00	0.00	0.00		
Lepidium perfoliatum	3	---	---	---	0	1	0	0.00	0.00	0.00		
Lactuca tatarica	1	---	---	---	0	1	0	0.00	0.00	0.00		
Leontopodium nivale subsp. alpinum	6	---	---	5	0	0	1	0.00	0.00	0.01		
Leonurus cardiaca	8	---	---	---	1	1	0	0.00	0.00	0.00		
Leonurus quinquelobatus	4	---	---	---	1	1	0	0.00	0.00	0.00		
Lathyrus vernus	17	---	---	3	1	1	1	0.00	0.00	0.02		
Bupleurum tenuissimum	7	---	3	---	0	1	0	0.00	0.01	0.00		
Bupleurum pachnospermum	4	---	---	---	0	1	0	0.00	0.00	0.00		
Ligularia carpathica	2	---	---	3	0	0	1	0.00	0.00	0.00		
Ligusticum mutellina	1	---	---	---	1	0	0	0.00	0.00	0.00		
Bupleurum rotundifolium	3	---	---	---	1	1	0	0.00	0.00	0.00		
Raphanus raphanistrum	5	---	---	---	1	1	0	0.00	0.00	0.00		
Ranunculus repens	16	4	---	---	1	1	0	0.01	0.00	0.00		
Arabidopsis halleri	5	3	---	---	1	0	0	0.00	0.00	0.00		
Lathyrus sphaericus	3	---	---	---	0	1	0	0.00	0.00	0.00		
Stachys arvensis	1	---	---	---	0	1	0	0.00	0.00	0.00		
Ranunculus sardous	13	---	---	---	1	1	0	0.00	0.00	0.00		
Moenchia mantica	26	3	---	---	1	1	0	0.02	0.02	0.00		
Aurinia petraea	16	---	---	---	0	1	1	0.00	0.00	0.01		
Minuartia viscosa	1	---	---	---	0	1	0	0.00	0.00	0.00		
Lens nigricans	1	---	---	---	1	0	0	0.00	0.00	0.00		
Bunium bulbocastanum	1	---	---	---	0	1	0	0.00	0.00	0.00		
Bupleurum longifolium	5	---	---	---	0	1	1	0.00	0.00	0.00		
Leucanthemum rotundifolium	7	---	---	6	0	0	1	0.00	0.00	0.01		

	total no. of occurrences	Phi value x 100 (orange: phi >= 0.2)			Constancy in % (green: const. ratio >= 2)			Average cover in % (blue: cover ratio >= 2)			Diagnostic value	Comment
Phytosociological order		B	Fv	S-Fp	B	Fv	S-Fp	B	Fv	S-Fp		
Number of relevés		3470	5016	1472	3470	5016	1472	3470	5016	1472		
Bupleurum praealtum	4	---	---	---	0	1	0	0.00	0.00	0.00		
Leucanthemum platylepis	2	---	---	---	1	0	0	0.00	0.00	0.00		
Moehringia trinervia	8	---	---	---	1	1	1	0.00	0.00	0.01		
Misopates orontium	1	---	---	---	0	1	0	0.00	0.00	0.00		
Bupleurum affine	15	---	4	---	0	1	0	0.00	0.01	0.00		
Orchis mascula	14	5	---	---	1	0	0	0.01	0.00	0.00		
Astragalus pallescens	3	---	---	---	0	1	0	0.00	0.00	0.00		
Aruncus dioicus	3	---	---	---	0	1	1	0.00	0.00	0.00		
Orchis purpurea	17	6	---	---	1	0	0	0.01	0.00	0.00		
Orchis simia	5	---	3	---	0	1	0	0.00	0.00	0.00		
Ophioglossum vulgatum	7	4	---	---	1	0	0	0.01	0.00	0.00		
Orobanche lanuginosa	1	---	---	---	0	1	0	0.00	0.00	0.00		
Orobanche elatior	18	5	---	---	1	1	0	0.01	0.00	0.00		
Orobanche arenaria	7	---	3	---	0	1	0	0.00	0.00	0.00		
Poa molinerii	2	---	---	3	0	0	1	0.00	0.00	0.00		
Orchis pallens	3	---	---	---	1	0	1	0.00	0.00	0.00		
Orobanche laserpitii-sileris	1	---	---	---	1	0	0	0.00	0.00	0.00		
Poa chaixii	3	2	---	---	1	0	0	0.00	0.00	0.00		
Poa palustris	3	---	---	---	1	1	0	0.00	0.00	0.00		
Artemisia scoparia	24	---	6	---	0	1	0	0.00	0.02	0.00		
Poa trivialis	18	---	---	---	1	1	0	0.01	0.02	0.00		
Poa rehmannii	1	---	---	---	0	0	1	0.00	0.00	0.00		
Poa stiriaca	3	---	---	---	1	1	1	0.00	0.00	0.00		
Plantago maxima	2	---	---	---	0	1	0	0.00	0.00	0.00		
Asclepias syriaca	3	---	---	---	0	1	0	0.00	0.00	0.00		
Astragalus glycyphylloides	3	---	---	---	0	1	0	0.00	0.00	0.00		
Asperugo procumbens	1	---	---	---	0	1	0	0.00	0.00	0.00		
Plantago schwarzenbergiana	1	---	---	---	0	1	0	0.00	0.00	0.00		
Plantago maritima	18	---	4	---	1	1	0	0.00	0.01	0.00		
Astragalus albicaulis	5	---	3	---	0	1	0	0.00	0.01	0.00		
Paeonia tenuifolia	17	---	---	---	1	1	0	0.00	0.02	0.00		
Oxalis stricta	4	---	---	---	1	1	0	0.00	0.00	0.00		
Papaver albiflorum subsp. austromoravic	7	---	---	---	1	1	0	0.00	0.00	0.00		
Orobanche coerulescens	6	---	---	---	0	1	1	0.00	0.00	0.00		
Orobanche cumana	1	---	---	---	0	1	0	0.00	0.00	0.00		
Pedicularis hacquetii	1	---	---	---	1	0	0	0.00	0.00	0.00		
Onopordum acanthium	18	---	4	---	1	1	0	0.00	0.01	0.00		
Onosma tornensis	10	---	---	4	0	1	1	0.00	0.00	0.01		
Pedicularis acaulis	5	3	---	---	1	0	0	0.00	0.00	0.00		
Astragalus ponticus	5	---	3	---	0	1	0	0.00	0.00	0.00		
Astracantha arnacantha	2	---	---	---	0	1	0	0.00	0.00	0.00		
Pedicularis kaufmannii	25	---	5	---	1	1	0	0.00	0.02	0.00		
Petasites kablikianus	1	---	---	---	0	0	1	0.00	0.00	0.00		
Astragalus australis	23	---	6	---	0	1	0	0.00	0.01	0.00		
Orobanche artemisiae-campestris	4	---	---	---	1	1	0	0.00	0.00	0.00		
Orobanche flava	9	---	3	---	1	1	0	0.00	0.00	0.00		
Petasites albus	2	---	---	---	1	1	0	0.00	0.00	0.00		
Orobanche purpurea	16	---	2	---	1	1	1	0.00	0.00	0.00		
Orobanche reticulata	2	---	---	---	1	1	0	0.00	0.00	0.00		
Orobanche minor	1	---	---	---	0	1	0	0.00	0.00	0.00		
Petasites hybridus	1	---	---	---	0	0	1	0.00	0.00	0.00		
Oxalis acetosella	8	---	---	3	1	1	1	0.00	0.00	0.01		
Panicum capillare	1	---	---	---	0	1	0	0.00	0.00	0.00		
Ochlopoa annua	9	---	---	---	1	1	0	0.00	0.01	0.00		
Schivereckia podolica	8	---	3	---	0	1	0	0.00	0.02	0.00		
Schoenus nigricans	1	---	---	---	1	0	0	0.00	0.00	0.00		
Sclerochloa dura	2	---	---	---	0	1	0	0.00	0.00	0.00		
Anemone ranunculoides	1	---	---	---	1	0	0	0.00	0.00	0.00		

	total no. of occurrences	Phi value x 100 (orange: phi >= 0.2)			Constancy in % (green: const. ratio >= 2)			Average cover in % (blue: cover ratio >= 2)			Diagnostic value	Comment
Phytosociological order		B	Fv	S-Fp	B	Fv	S-Fp	B	Fv	S-Fp		
Number of relevés		3470	5016	1472	3470	5016	1472	3470	5016	1472		
Anemone narcissiflora	5	3	---	---	1	0	0	0.01	0.00	0.00		
Androsace septentrionalis	2	---	---	---	0	1	0	0.00	0.00	0.00		
Anthemis arvensis	12	---	3	---	1	1	0	0.00	0.00	0.00		
Scirpoides holoschoenus	18	---	4	---	1	1	0	0.00	0.02	0.00		
Scorzonera parviflora	2	---	---	---	0	1	0	0.00	0.00	0.00		
Androsace lactea	2	---	---	3	0	0	1	0.00	0.00	0.01		
Antennaria carpatica	1	---	---	---	1	0	0	0.00	0.00	0.00		
Sambucus ebulus	23	---	4	---	1	1	0	0.00	0.01	0.00		
Saponaria glutinosa	5	---	3	---	0	1	0	0.00	0.00	0.00		
Sanicula europaea	3	---	---	---	1	0	1	0.00	0.00	0.00		
Satureja kitaibelii	6	---	---	---	0	1	1	0.00	0.00	0.00		
Arenaria grandiflora	3	---	---	---	0	1	1	0.00	0.00	0.00		
Arenaria ciliata	1	---	---	---	0	1	0	0.00	0.00	0.00		
Primula farinosa	1	---	---	---	1	0	0	0.00	0.00	0.00		
Anemone trifolia	1	---	---	---	1	0	0	0.00	0.00	0.00		
Scorzonera mollis	7	---	3	---	0	1	0	0.00	0.00	0.00		
Scabiosa argentea	3	---	---	---	0	1	0	0.00	0.00	0.00		
Sanguisorba verrucosa	2	---	---	---	1	0	0	0.00	0.00	0.00		
Saponaria officinalis	14	---	2	---	1	1	1	0.00	0.00	0.00		
Saxifraga marginata	4	---	---	4	0	0	1	0.00	0.00	0.01		
Notholaena marantae	2	---	---	---	0	1	1	0.00	0.00	0.00		
Nonea atra	1	---	---	---	0	1	0	0.00	0.00	0.00		
Astragalus ucrainicus	21	---	5	---	0	1	0	0.00	0.01	0.00		
Astragalus peterfii	7	---	3	---	0	1	0	0.00	0.00	0.00		
Astragalus corniculatus	18	---	5	---	0	1	0	0.00	0.01	0.00		
Ornithogalum pyrenaicum s.lat.	12	5	---	---	1	0	0	0.01	0.00	0.00		
Astragalus sulcatus	19	---	5	---	0	1	0	0.00	0.04	0.00		
Onobrychis alba	4	---	---	---	0	1	0	0.00	0.00	0.00		
Astragalus varius	3	---	---	---	0	1	0	0.00	0.00	0.00		
Onosma helvetica	1	---	---	---	0	1	0	0.00	0.00	0.00		
Onosma heterophylla	12	---	---	---	0	1	1	0.00	0.00	0.01		
Oenanthe silaifolia	3	---	---	---	1	1	0	0.00	0.00	0.00		
Scorzonera villosa	5	3	---	---	1	0	0	0.00	0.00	0.00		
Orthilia secunda	5	---	3	---	0	1	0	0.00	0.01	0.00		
Ornithogalum collinum	21	---	4	---	1	1	0	0.00	0.01	0.00		
Scorzonera humilis	7	2	---	---	1	0	1	0.00	0.00	0.00		
Androsace elongata	19	---	5	---	0	1	0	0.00	0.01	0.00		
Androsace koso-poljanskii	4	---	---	---	0	1	0	0.00	0.03	0.00		
Ornithogalum comosum	6	---	---	4	0	1	1	0.00	0.00	0.00		
Orobanche alsatica	4	3	---	---	1	0	0	0.00	0.00	0.00		
Ophrys sphegodes	12	3	---	---	1	1	0	0.01	0.00	0.00		
Ornithogalum boucheanum	3	---	---	---	0	1	0	0.00	0.00	0.00		
Ornithogalum fischerianum	1	---	---	---	0	1	0	0.00	0.00	0.00		
Astragalus pubiflorus	1	---	---	---	0	1	0	0.00	0.00	0.00		
Agrostis canina	15	---	3	---	1	1	0	0.00	0.01	0.00		
Valeriana montana	5	---	---	5	0	0	1	0.00	0.00	0.01		
Valerianella rimosa	9	---	---	---	1	1	1	0.00	0.00	0.01		
Valerianella carinata	17	---	4	---	1	1	0	0.00	0.01	0.00		
Valerianella coronata	8	---	3	---	0	1	0	0.00	0.01	0.00		
Podospermum laciniatum	17	---	4	---	1	1	0	0.00	0.01	0.00		
Artemisia santonicum	5	---	---	---	1	1	0	0.00	0.00	0.00		
Polygala cretacea	5	---	3	---	0	1	0	0.00	0.01	0.00		
Polygonatum verticillatum	12	1	---	---	1	0	1	0.00	0.00	0.01		
Artemisia lerchiana	1	---	---	---	0	1	0	0.00	0.00	0.00		
Podospermum roseum	1	---	---	---	1	0	0	0.00	0.00	0.00		
Trifolium purpureum	1	---	---	---	1	0	0	0.00	0.00	0.00		
Valeriana dioica	1	---	---	---	1	0	0	0.00	0.00	0.00		
Vaccinium myrtillus	2	---	---	---	1	0	1	0.00	0.00	0.00		

	total no. of occurrences	Phi value x 100 (orange: phi >= 0.2)			Constancy in % (green: const. ratio >= 2)			Average cover in % (blue: cover ratio >= 2)			Diagnostic value	Comment
Phytosociological order		B	Fv	S-Fp	B	Fv	S-Fp	B	Fv	S-Fp		
Number of relevés		3470	5016	1472	3470	5016	1472	3470	5016	1472		
Tripolium pannonicum	2	---	---	---	0	1	0	0.00	0.00	0.00		
Ventenata dubia	13	---	4	---	0	1	0	0.00	0.01	0.00		
Valerianella costata	1	---	---	---	0	1	0	0.00	0.00	0.00		
Vaccinium vitis-idaea	6	---	---	3	1	0	1	0.00	0.00	0.01		
Trifolium patens	10	---	---	---	1	1	0	0.00	0.00	0.00		
Aira elegantissima	4	---	---	---	1	1	0	0.00	0.00	0.00		
Valeriana tuberosa	1	---	---	---	0	1	0	0.00	0.00	0.00		
Turgenia latifolia	2	---	---	---	0	1	0	0.00	0.00	0.00		
Trifolium pallidum	7	4	---	---	1	0	0	0.01	0.00	0.00		
Polygonum aviculare agg.	31	---	4	---	1	1	0	0.00	0.02	0.00		
Pinguicula alpina	3	---	---	4	0	0	1	0.00	0.00	0.01		
Polygonatum multiflorum	5	---	---	---	1	1	1	0.00	0.00	0.00		
Artemisia annua	8	---	3	---	0	1	0	0.00	0.01	0.00		
Polygonum hydropiper	1	---	---	---	0	1	0	0.00	0.00	0.00		
Piptatherum virescens	8	---	---	3	1	1	1	0.00	0.00	0.01		
Plantago arenaria	10	---	3	---	1	1	0	0.00	0.00	0.00		
Pilosella floribunda	3	2	---	---	1	0	0	0.00	0.00	0.00		
Pinguicula vulgaris	1	---	---	---	0	0	1	0.00	0.00	0.00		
Piptatherum holciforme	4	---	---	---	0	1	0	0.00	0.00	0.00		
Phleum hirsutum	7	4	---	---	1	0	0	0.00	0.00	0.00		
Polygonum arenarium	10	---	4	---	0	1	0	0.00	0.01	0.00		
Artemisia pancicii	2	---	---	---	0	1	0	0.00	0.00	0.00		
Polycnemum majus	5	---	3	---	0	1	0	0.00	0.00	0.00		
Physospermum cornubiense	10	---	---	---	1	1	0	0.00	0.00	0.00		
Physalis alkekengi	1	---	---	---	0	1	0	0.00	0.00	0.00		
Asplenium cuneifolium	6	---	---	---	1	1	1	0.00	0.00	0.02		
Polygonatum hirtum	1	---	---	---	0	0	1	0.00	0.00	0.00		
Platanthera chlorantha	2	---	---	3	0	0	1	0.00	0.00	0.00		
Polygala nicaeensis	6	3	---	---	1	0	0	0.00	0.00	0.00		
Poa alpina	9	---	---	5	1	0	1	0.00	0.00	0.01		
Artemisia hololeuca	2	---	---	---	0	1	0	0.00	0.01	0.00		
Pleurospermum austriacum	3	---	---	4	0	0	1	0.00	0.00	0.00		
Taraxacum sect. Dioszegia	2	---	---	---	0	1	0	0.00	0.00	0.00		
Althaea officinalis	6	---	---	---	1	1	0	0.00	0.00	0.00		
Taraxacum bessarabicum	1	---	---	---	0	1	0	0.00	0.00	0.00		
Allium suaveolens	1	---	---	---	1	0	0	0.00	0.00	0.00		
Althaea hirsuta	2	---	---	---	1	1	0	0.00	0.00	0.00		
Stipa zalesskii	1	---	---	---	0	1	0	0.00	0.00	0.00		
Alopecurus arundinaceus	3	---	---	---	0	1	0	0.00	0.00	0.00		
Tripleurospermum inodorum	25	---	4	---	1	1	0	0.00	0.01	0.00		
Althaea cannabina	1	---	---	---	0	1	0	0.00	0.00	0.00		
Alopecurus rendlei	1	---	---	---	1	0	0	0.00	0.00	0.00		
Swertia perennis	4	---	---	4	0	0	1	0.00	0.00	0.01		
Thalictrum flavum	3	---	---	---	1	1	0	0.00	0.00	0.00		
Allium paczoskianum	20	---	5	---	0	1	0	0.00	0.01	0.00		
Teucrium scorodonia	8	---	3	---	0	1	0	0.00	0.05	0.00		
Allium regelianum	1	---	---	---	0	1	0	0.00	0.00	0.00		
Selinum carvifolia	10	3	---	---	1	1	0	0.00	0.00	0.00		
Selinum dubium	8	---	---	---	1	1	0	0.00	0.00	0.00		
Stipa ucrainica	12	---	4	---	0	1	0	0.00	0.11	0.00		
Taraxacum sect. Palustria	1	---	---	---	1	0	0	0.00	0.00	0.00		
Allium strictum	1	---	---	---	0	1	0	0.00	0.00	0.00		
Tephroseris besseriana	1	---	---	---	1	0	0	0.00	0.00	0.00		
Teucrium scordium	2	---	---	---	0	1	0	0.00	0.00	0.00		
Thalictrum lucidum	17	4	---	---	1	1	0	0.01	0.00	0.00		
Asperula purpurea	14	---	---	---	1	1	1	0.00	0.00	0.00		
Trifolium subterraneum	3	---	---	---	1	1	0	0.00	0.00	0.00		
Pilosella densiflora	1	---	---	---	1	0	0	0.00	0.00	0.00		

	total no. of occurrences	Phi value x 100 (orange: phi >= 0.2)			Constancy in % (green: const. ratio >= 2)			Average cover in % (blue: cover ratio >= 2)			Diagnostic value	Comment
Phytosociological order		B	Fv	S-Fp	B	Fv	S-Fp	B	Fv	S-Fp		
Number of relevés		3470	5016	1472	3470	5016	1472	3470	5016	1472		
Pilosella lactucella	17	2	---	---	1	1	1	0.01	0.00	0.01		
Asperula rumelica	6	---	3	---	0	1	0	0.00	0.00	0.00		
Aira caryophyllea	2	---	---	---	0	1	0	0.00	0.00	0.00		
Trisetum sibiricum	7	---	3	---	0	1	0	0.00	0.01	0.00		
Trollius europaeus	2	---	---	---	1	0	0	0.00	0.00	0.00		
Tulipa biebersteiniana	6	---	3	---	0	1	0	0.00	0.00	0.00		
Trifolium strictum	1	---	---	---	0	1	0	0.00	0.00	0.00		
Scorzoneroides helvetica	2	---	---	---	1	0	0	0.00	0.00	0.00		
Asperula neilreichii	3	---	---	4	0	0	1	0.00	0.00	0.00		
Triglochin maritima	1	---	---	---	0	1	0	0.00	0.00	0.00		
Trigonella gladiata	5	---	3	---	0	1	0	0.00	0.00	0.00		
Trifolium retusum	1	---	---	---	0	1	0	0.00	0.00	0.00		
Trinia kitaibelii	18	---	3	---	1	1	0	0.00	0.01	0.00		
Trinia multicaulis	9	---	---	---	1	1	0	0.00	0.00	0.00		
Trifolium fragiferum	7	---	---	---	1	1	0	0.00	0.00	0.00		
Ajuga orientalis	4	---	---	---	0	1	0	0.00	0.00	0.00		
Pilosella rothiana	1	---	---	---	0	1	0	0.00	0.00	0.00		
Trifolium micranthum	9	---	---	---	1	1	0	0.01	0.01	0.00		
Trifolium hybridum	16	4	---	---	1	1	0	0.01	0.00	0.00		
Alcea biennis	2	---	---	---	0	1	0	0.00	0.00	0.00		
Pilosella hoppeana	41	---	---	---	1	1	1	0.02	0.01	0.01		
Thlaspi arvense	17	---	---	---	1	1	1	0.00	0.01	0.00		
Thymus moldavicus	1	---	---	---	0	1	0	0.00	0.00	0.00		
Thesium procumbens	20	---	5	---	0	1	0	0.00	0.06	0.00		
Thesium pyrenaicum	2	---	---	---	1	0	0	0.00	0.00	0.00		
Allium angulosum	8	---	---	---	1	1	0	0.00	0.00	0.00		
Thymus bihoriensis	3	---	---	---	0	1	0	0.00	0.00	0.00		
Thymus calcareus	6	---	3	---	0	1	0	0.00	0.00	0.00		
Allium fuscum	7	---	3	---	0	1	0	0.00	0.00	0.00		
Alcea rosea	1	---	---	---	0	1	0	0.00	0.00	0.00		
Allium inaequale	7	---	3	---	0	1	0	0.00	0.00	0.00		
Urtica dioica	27	---	---	---	1	1	1	0.01	0.01	0.01		
Allium marginatum	9	---	---	---	1	1	0	0.00	0.00	0.00		
Xanthium spinosum	6	---	3	---	0	1	0	0.00	0.00	0.00		
Achillea coarctata	14	---	3	---	0	1	1	0.00	0.01	0.00		
Acanthus hungaricus	2	---	---	---	0	1	0	0.00	0.00	0.00		
Xanthium orientale	7	---	---	---	1	1	0	0.00	0.00	0.00		
Vulpia myuros	11	---	4	---	0	1	0	0.00	0.01	0.00		
Viola mirabilis	19	2	---	---	1	1	1	0.01	0.00	0.01		
Thesium dollineri	4	---	---	---	0	1	0	0.00	0.00	0.00		
Thesium ebracteatum	2	---	---	---	0	1	0	0.00	0.00	0.00		
Thalictrum uncinatum	8	---	---	---	1	1	0	0.00	0.01	0.00		
Xanthium strumarium	12	---	4	---	0	1	0	0.00	0.00	0.00		
Viola lutea	1	---	---	---	1	0	0	0.00	0.00	0.00		
Trifolium alpinum	1	---	---	---	0	1	0	0.00	0.00	0.00		
Trichophorum alpinum	1	---	---	---	1	0	0	0.00	0.00	0.00		
Allium atropurpureum	1	---	---	---	0	1	0	0.00	0.00	0.00		
Tribulus terrestris	1	---	---	---	0	1	0	0.00	0.00	0.00		
Trifolium angulatum	8	---	---	---	1	1	0	0.02	0.01	0.00		
Alcea rugosa	2	---	---	---	0	1	0	0.00	0.00	0.00		
Trifolium diffusum	4	---	---	---	0	1	0	0.00	0.00	0.00		
Trifolium badium	2	---	---	---	1	1	0	0.00	0.00	0.00		
Allium albidum subsp. albidum	18	---	5	---	0	1	0	0.00	0.00	0.00		
Alliaria petiolata	14	---	---	---	1	1	1	0.00	0.00	0.00		
Tragopogon borysthenicus	4	---	---	---	0	1	0	0.00	0.01	0.00		
Allium denudatum	4	---	---	---	0	1	0	0.00	0.00	0.00		
Tordylium maximum	3	---	---	---	1	1	0	0.00	0.00	0.00		
Tragopogon dasyrhynchus	1	---	---	---	0	1	0	0.00	0.00	0.00		

	total no. of occurrences	Phi value x 100 (orange: phi >= 0.2)			Constancy in % (green: const. ratio >= 2)			Average cover in % (blue: cover ratio >= 2)			Diagnostic value	Comment
Phytosociological order		B	Fv	S-Fp	B	Fv	S-Fp	B	Fv	S-Fp		
Number of relevés		3470	5016	1472	3470	5016	1472	3470	5016	1472		
Torilis arvensis	27	---	4	---	1	1	0	0.00	0.01	0.00		
Allium flavescens	21	---	4	---	0	1	1	0.00	0.01	0.00		
Thymus pallasianus	6	---	3	---	0	1	0	0.00	0.02	0.00		
Torilis japonica	24	2	---	---	1	1	1	0.01	0.00	0.00		
Tragopogon floccosus	1	---	---	---	0	1	0	0.00	0.00	0.00		
Tragopogon ucrainicus	14	---	4	---	0	1	0	0.00	0.02	0.00		
Viola accrescens	6	---	3	---	0	1	0	0.00	0.00	0.00		
Allium decipiens	1	---	---	---	0	1	0	0.00	0.00	0.00		
Tragopogon podolicus	12	---	4	---	0	1	0	0.00	0.00	0.00		
Vicia serratifolia	3	---	---	---	0	1	0	0.00	0.00	0.00		
Vicia lutea	3	---	---	---	1	1	0	0.00	0.00	0.00		
Vicia pannonica	20	---	5	---	1	1	0	0.00	0.01	0.00		
Phedimus spurius	2	---	---	---	0	1	1	0.00	0.00	0.00		
Tussilago farfara	36	---	---	---	1	1	1	0.01	0.03	0.00		
Aconitum lycoctonum	8	---	---	5	0	1	1	0.00	0.00	0.01		
Woodsia ilvensis	2	---	---	---	0	1	1	0.00	0.00	0.00		
Aconitum confertiflorum	5	---	---	---	1	1	0	0.00	0.00	0.00		
Waldsteinia geoides	5	---	---	---	1	1	0	0.00	0.00	0.00		
Vicia pisiformis	2	---	---	---	1	1	0	0.00	0.00	0.00		
Achillea salicifolia	1	---	---	---	0	1	0	0.00	0.00	0.00		
Phalaroides arundinacea	2	---	---	---	1	0	0	0.00	0.00	0.00		
Asphodelus albus	2	---	---	3	0	0	1	0.00	0.00	0.00		
Asperula tenella	7	---	---	3	0	1	1	0.00	0.00	0.01		
Pilosella auriculoides	16	---	---	---	1	1	1	0.00	0.00	0.01		
Pisum sativum subsp. elatius	1	---	---	---	0	1	0	0.00	0.00	0.00		
Plantago altissima	3	---	---	---	1	1	0	0.00	0.00	0.00		
Phyteuma spicatum	28	2	---	---	1	1	1	0.02	0.00	0.01		
Phleum rhaeticum	1	---	---	---	0	0	1	0.00	0.00	0.00		
Peucedanum officinale	15	4	---	---	1	1	0	0.01	0.01	0.00		
Phegopteris connectilis	3	---	---	4	0	0	1	0.00	0.00	0.00		
Asperula tephrocarpa	4	---	---	---	0	1	0	0.00	0.01	0.00		
Pilosella aurantiaca	4	---	---	---	0	1	1	0.00	0.00	0.00		
Achillea atrata	2	---	---	---	1	0	0	0.00	0.00	0.00		
Agrimonia pilosa	1	---	---	---	0	1	0	0.00	0.00	0.00		
Achillea clavennae	1	---	---	---	0	0	1	0.00	0.00	0.00		
Viola reichenbachiana	18	5	---	---	1	0	1	0.01	0.00	0.00		
Viola riviniana	12	3	---	---	1	0	1	0.01	0.00	0.00		
Veronica bachofenii	9	---	3	---	0	1	0	0.00	0.00	0.00		
Xeranthemum cylindraceum	12	3	---	---	1	1	0	0.01	0.00	0.00		
Xeranthemum inapertum	33	---	---	---	1	1	0	0.03	0.04	0.00		
Veronica agrestis	4	---	---	---	0	1	1	0.00	0.00	0.00		
Veronica acinifolia	2	---	---	---	1	0	0	0.00	0.00	0.00		
Aethusa cynapium	4	---	---	---	0	1	0	0.00	0.00	0.00		
Viola pumila	8	---	---	---	1	1	0	0.00	0.00	0.00		
Vinca minor	1	---	---	---	0	1	0	0.00	0.00	0.00		
Viola alba	1	---	---	---	0	0	1	0.00	0.00	0.00		
Achillea ochroleuca	18	---	5	---	0	1	0	0.00	0.01	0.00		
Vicia sylvatica	5	---	---	---	1	1	1	0.00	0.00	0.00		
Vincetoxicum fuscatum	5	---	3	---	0	1	0	0.00	0.00	0.00		
Achillea ptarmica	2	---	---	---	0	1	0	0.00	0.00	0.00		
Viola elatior	1	---	---	---	1	0	0	0.00	0.00	0.00		
Achillea crithmifolia	11	---	4	---	0	1	0	0.00	0.01	0.00		
Viola biflora	4	---	---	4	0	0	1	0.00	0.00	0.01		
Viola cretacea	1	---	---	---	0	1	0	0.00	0.00	0.00		
Achillea micrantha	5	---	---	---	1	1	0	0.00	0.03	0.00		
Selaginella selaginoides	1	---	---	---	0	0	1	0.00	0.00	0.00		
Silene gallica	2	---	---	---	0	1	0	0.00	0.00	0.00		
Silene dichotoma	2	---	---	---	0	1	0	0.00	0.01	0.00		

	total no. of occurrences	Phi value x 100 (orange: phi >= 0.2)			Constancy in % (green: const. ratio >= 2)			Average cover in % (blue: cover ratio >= 2)			Diagnostic value	Comment
Phytosociological order		B	Fv	S-Fp	B	Fv	S-Fp	B	Fv	S-Fp		
Number of relevés		3470	5016	1472	3470	5016	1472	3470	5016	1472		
Silene conica	8	---	3	---	0	1	0	0.00	0.00	0.00		
Serratula coronata	2	---	---	---	0	1	0	0.00	0.01	0.00		
Sedum annuum	2	---	---	---	0	1	0	0.00	0.00	0.00		
Amygdalus nana	20	---	5	---	0	1	0	0.00	0.09	0.00		
Alyssum smyrnaeum	1	---	---	---	0	1	0	0.00	0.01	0.00		
Silene viridiflora	8	---	2	---	0	1	1	0.00	0.00	0.00		
Silene noctiflora	3	---	---	---	0	1	0	0.00	0.00	0.00		
Amaranthus retroflexus	2	---	---	---	0	1	0	0.00	0.00	0.00		
Seseli montanum subsp. tommasinii	6	---	---	4	1	1	1	0.00	0.02	0.03		
Silene dioica	3	---	---	---	1	0	1	0.00	0.00	0.00		
Bellis perennis	42	6	---	---	1	1	0	0.02	0.00	0.00		
Brachypodium sylvaticum	74	6	---	---	1	1	1	0.14	0.01	0.01		
Centaurea nigrescens	18	6	---	---	1	0	0	0.01	0.00	0.00		
Carex distans	31	5	---	---	1	1	0	0.05	0.00	0.00		
Aegopodium podagraria	26	5	---	---	1	0	1	0.01	0.00	0.00		
Poa annua	2	---	---	---	0	1	0	0.00	0.00	0.00		
Silene tatarica	20	---	5	---	0	1	0	0.00	0.03	0.00		
Silene subconica	1	---	---	---	0	1	0	0.00	0.00	0.00		
Silaum silaus	8	3	---	---	1	1	0	0.01	0.00	0.00		
Silene armeria	4	---	---	---	0	1	1	0.00	0.00	0.00		
Sherardia arvensis	4	---	---	---	1	1	0	0.00	0.00	0.00		
Sedum borissovae	1	---	---	---	0	1	0	0.00	0.00	0.00		
Androsace chamaejasme	2	---	---	3	0	0	1	0.00	0.00	0.01		
Anchusa procera	12	---	4	---	0	1	0	0.00	0.01	0.00		
Sedum dasyphyllum	4	---	---	---	0	1	1	0.00	0.00	0.00		
Anchusa ochroleuca	1	---	---	---	0	1	0	0.00	0.00	0.00		
Scrophularia nodosa	5	---	---	---	1	1	0	0.00	0.00	0.00		
Adonis aestivalis	6	---	3	---	0	1	0	0.00	0.00	0.00		
Vicia grandiflora	25	---	4	---	1	1	0	0.01	0.02	0.00		
Scrophularia rupestris	5	---	---	5	0	0	1	0.00	0.00	0.01		
Scrophularia canina	1	---	---	---	0	1	0	0.00	0.00	0.00		
Scrophularia scopolii	2	---	---	---	1	1	0	0.00	0.00	0.00		
Senecio macrophyllus	2	---	---	---	0	1	0	0.00	0.00	0.00		
Secale sylvestre	5	---	3	---	0	1	0	0.00	0.01	0.00		
Scrophularia heterophylla subsp. laciniata	1	---	---	---	0	0	1	0.00	0.00	0.00		
Scutellaria supina	14	---	4	---	0	1	0	0.00	0.02	0.00		
Silene multiflora	2	---	---	---	0	1	0	0.00	0.00	0.00		
Securigera elegans	6	---	---	5	0	0	1	0.00	0.00	0.01		
Scutellaria altissima	1	---	---	---	0	1	0	0.00	0.00	0.00		
Senecio nemorensis agg.	10	---	---	5	1	0	1	0.00	0.00	0.01		
Scutellaria hastifolia	4	---	---	---	1	1	0	0.00	0.00	0.00		
Senecio erraticus	6	3	---	---	1	1	0	0.00	0.00	0.00		
Scutellaria galericulata	1	---	---	---	0	1	0	0.00	0.00	0.00		
Anagallis foemina	9	---	---	---	1	1	0	0.00	0.00	0.00		
Portulaca oleracea	5	---	3	---	0	1	0	0.00	0.00	0.00		
Potentilla anserina	10	---	---	---	1	1	0	0.00	0.00	0.00		
Polygonum mite	1	---	---	---	0	1	0	0.00	0.00	0.00		
Potentilla micrantha	2	---	---	---	1	0	1	0.00	0.00	0.00		
Aristolochia clematitis	17	---	2	---	1	1	1	0.00	0.02	0.00		
Artemisia abrotanum	9	---	3	---	0	1	0	0.00	0.00	0.00		
Lysimachia nummularia	40	7	---	---	1	1	0	0.02	0.00	0.00		
Noccaea praecox	36	8	---	---	1	0	0	0.02	0.00	0.00		
Lychnis flos-cuculi	21	6	---	---	1	1	0	0.01	0.00	0.00		
Polygonum patulum	2	---	---	---	0	1	0	0.00	0.00	0.00		
Potentilla aurea	5	---	---	4	1	0	1	0.00	0.00	0.01		
Potentilla caulescens	3	---	---	---	0	1	0	0.00	0.00	0.00		
Arctium tomentosum	7	3	---	---	1	1	0	0.00	0.00	0.00		
Arctium nemorosum	1	---	---	---	0	1	0	0.00	0.00	0.00		

	total no. of occurrences	Phi value x 100 (orange: phi >= 0.2)			Constancy in % (green: const. ratio >= 2)			Average cover in % (blue: cover ratio >= 2)			Diagnostic value	Comment
Phytosociological order		B	Fv	S-Fp	B	Fv	S-Fp	B	Fv	S-Fp		
Number of relevés		3470	5016	1472	3470	5016	1472	3470	5016	1472		
Arctostaphylos uva-ursi	2	---	---	3	0	0	1	0.00	0.00	0.02		
Prospero autumnale	10	---	---	4	0	1	1	0.00	0.00	0.00		
Aremonia agrimonoides	3	2	---	---	1	0	0	0.00	0.00	0.00		
Arctium minus	2	---	---	---	1	1	0	0.00	0.00	0.00		
Arenaria cephalotes	1	---	---	---	0	1	0	0.00	0.00	0.00		
Polygonum viviparum	1	---	---	---	1	0	0	0.00	0.00	0.00		
Prangos ferulacea	2	---	---	---	0	1	0	0.00	0.00	0.00		
Potentilla supina	1	---	---	---	0	1	0	0.00	0.00	0.00		
Arenaria longifolia	13	---	4	---	0	1	0	0.00	0.01	0.00		
Centaurea oxylepis	32	8	---	---	1	1	0	0.03	0.00	0.00		
Aquilegia nigricans	21	5	---	---	1	0	1	0.01	0.00	0.01		
Anemone nemorosa	22	5	---	---	1	1	1	0.04	0.00	0.00		
Myosotis sylvatica agg.	22	4	---	---	1	1	1	0.01	0.00	0.00		
Helleborus purpurascens	29	6	---	---	1	1	0	0.02	0.00	0.00		
Ranunculus pseudomontanus	3	---	---	4	0	0	1	0.00	0.00	0.00		
Carex panicea	44	8	---	---	1	1	1	0.03	0.00	0.00		
Calamagrostis arundinacea	28	3	---	---	1	1	1	0.03	0.01	0.01		
Arabis soyeri	1	---	---	---	0	0	1	0.00	0.00	0.00		
Pulsatilla patens	13	2	---	---	1	1	1	0.01	0.00	0.00		
Ranunculus pedatus	15	---	4	---	1	1	0	0.00	0.01	0.00		
Neottia ovata	38	7	---	---	1	0	1	0.02	0.00	0.00		
Centaurea macroptilon	22	7	---	---	1	0	0	0.02	0.00	0.00		
Mercurialis ovata	30	3	---	---	1	0	1	0.01	0.00	0.00		
Melampyrum barbatum	70	4	---	---	1	1	1	0.02	0.01	0.01		
Nepeta nuda	57	6	---	---	1	1	0	0.02	0.01	0.00		
Ophrys apifera	28	7	---	---	1	0	1	0.02	0.00	0.00		
Veratrum nigrum	32	4	---	---	1	1	1	0.01	0.00	0.00		
Geum urbanum	43	3	---	---	1	1	1	0.02	0.00	0.01		
Nardus stricta	54	9	---	---	1	1	0	0.06	0.01	0.00		
Lathyrus sylvestris	42	8	---	---	1	1	0	0.02	0.01	0.00		
Geranium pratense	43	6	---	---	1	1	0	0.02	0.01	0.00		
Veronica vindobonensis	69	6	---	---	1	1	1	0.03	0.00	0.01		
Veronica spuria	6	---	---	---	1	1	0	0.00	0.02	0.00		
Stipa asperella	4	---	---	---	0	1	0	0.00	0.00	0.00		
Spiraea crenata	16	---	2	---	0	1	1	0.00	0.04	0.00		
Stipa anomala	1	---	---	---	0	1	0	0.00	0.00	0.00		
Stellaria palustris	2	---	---	---	1	0	0	0.00	0.00	0.00		
Sternbergia colchiciflora	16	---	5	---	0	1	0	0.00	0.02	0.00		
Stachys annua	14	---	3	---	1	1	0	0.00	0.01	0.00		
Sisymbrium strictissimum	5	---	---	---	1	1	0	0.00	0.00	0.00		
Smyrnium perfoliatum	2	---	---	---	1	1	0	0.00	0.00	0.00		
Sisyrinchium montanum	1	---	---	---	1	0	0	0.00	0.00	0.00		
Spergularia rubra	5	---	3	---	0	1	0	0.00	0.00	0.00		
Spergularia marina	1	---	---	---	0	1	0	0.00	0.00	0.00		
Stellaria media	19	---	3	---	1	1	0	0.00	0.01	0.00		
Taraxacum sect. Obliqua	2	---	---	---	0	1	0	0.00	0.00	0.00		
Tephroseris crispa	1	---	---	---	1	0	0	0.00	0.00	0.00		
Tephroseris papposa	1	---	---	---	0	0	1	0.00	0.00	0.00		
Tephroseris longifolia	4	3	---	---	1	0	0	0.00	0.00	0.00		
Tephroseris czernijevii	2	---	---	---	0	1	0	0.00	0.00	0.00		
Allium schoenoprasum	12	---	---	3	1	1	1	0.00	0.01	0.01		
Stipa borysthenica	20	---	5	---	1	1	0	0.01	0.06	0.00		
Soldanella montana agg.	2	---	---	---	1	0	1	0.00	0.00	0.00		
Solidago canadensis	22	---	---	---	1	1	0	0.01	0.01	0.00		
Soldanella carpatica	6	---	---	5	0	0	1	0.00	0.00	0.01		
Solanum dulcamara	5	3	---	---	1	0	0	0.00	0.00	0.00		
Syrenia cana	12	---	---	---	1	1	0	0.00	0.01	0.00		
Syrenia montana	5	---	3	---	0	1	0	0.00	0.00	0.00		

	total no. of occurrences	Phi value x 100 (orange: phi >= 0.2)			Constancy in % (green: const. ratio >= 2)			Average cover in % (blue: cover ratio >= 2)			Diagnostic value	Comment
Phytosociological order		B	Fv	S-Fp	B	Fv	S-Fp	B	Fv	S-Fp		
Number of relevés		3470	5016	1472	3470	5016	1472	3470	5016	1472		
Tanacetum macrophyllum	2	---	---	---	1	1	0	0.00	0.00	0.00		
Sempervivum tectorum	11	---	---	5	0	1	1	0.00	0.00	0.01		
Symphytum officinale	10	3	---	---	1	1	0	0.00	0.00	0.00		
Taeniatherum caput-medusae	3	---	---	---	0	1	0	0.00	0.00	0.00		
Senecio viscosus	8	---	3	---	0	1	0	0.00	0.00	0.00		
Senecio umbrosus	14	---	---	4	1	0	1	0.01	0.00	0.01		
Senecio vulgaris	10	---	---	---	1	1	1	0.00	0.00	0.00		
Senecio vernalis	14	---	4	---	0	1	0	0.00	0.01	0.00		
Senecio subalpinus	2	---	---	3	0	0	1	0.00	0.00	0.00		
Senecio borysthenicus	6	---	3	---	0	1	0	0.00	0.01	0.00		
Stachys palustris	2	---	---	---	1	1	0	0.00	0.00	0.00		
Stellaria holostea	16	---	---	---	1	1	1	0.00	0.00	0.00		
Stachys sylvatica	8	3	---	---	1	1	0	0.00	0.00	0.00		
Sisymbrium orientale	17	---	5	---	0	1	0	0.00	0.01	0.00		
Sisyrinchium septentrionale	1	---	---	---	1	0	0	0.00	0.00	0.00		
Alyssum linifolium	2	---	---	---	0	1	0	0.00	0.00	0.00		
Sempervivum ruthenicum	17	---	3	---	0	1	1	0.00	0.01	0.00		
Anchusa gmelinii	3	---	---	---	0	1	0	0.00	0.04	0.00		
Senecio aquaticus	1	---	---	---	1	0	0	0.00	0.00	0.00		
Stellaria hippoctona	1	---	---	---	0	1	0	0.00	0.00	0.00		
Alyssum desertorum	22	---	5	---	0	1	0	0.00	0.01	0.00		
Veronica urticifolia	4	---	---	4	0	0	1	0.00	0.00	0.01		
Agrimonia procera	6	---	3	---	0	1	0	0.00	0.00	0.00		
Agropyron dasyanthum	1	---	---	---	0	1	0	0.00	0.01	0.00		
Veronica capsellicarpa	1	---	---	---	0	1	0	0.00	0.00	0.00		
Agrostemma githago	1	---	---	---	0	1	0	0.00	0.00	0.00		
Verbascum marschallianum	25	---	6	---	0	1	0	0.00	0.01	0.00		
Setaria pumila	27	---	5	---	1	1	1	0.00	0.01	0.00		
Anacamptis palustris	3	---	---	---	0	1	0	0.00	0.00	0.00		
Setaria verticillata	1	---	---	---	0	1	0	0.00	0.00	0.00		
Verbascum speciosum	17	---	4	---	1	1	0	0.00	0.01	0.00		
Amaranthus albus	1	---	---	---	0	1	0	0.00	0.00	0.00		
Veronica barrelieri	8	---	2	---	1	1	0	0.00	0.00	0.00		
Aegonychon purpurocaeruleum	19	---	---	---	1	1	1	0.00	0.00	0.00		
Veronica paniculata	1	---	---	---	0	1	0	0.00	0.00	0.00		
Actaea spicata	1	---	---	---	0	0	1	0.00	0.00	0.00		
Veronica triphyllos	20	---	3	---	1	1	1	0.00	0.01	0.00		
Adonis volgensis	12	---	4	---	0	1	0	0.00	0.01	0.00		
Adenophora liliifolia	3	2	---	---	1	0	0	0.01	0.00	0.00		
Veratrum album	2	---	---	---	1	0	0	0.00	0.00	0.00		
Agropyron tanaiticum	2	---	---	---	0	1	0	0.00	0.00	0.00		
Verbascum blattaria	12	---	---	---	1	1	1	0.00	0.00	0.00		
Vicia dumetorum	3	2	---	---	1	0	0	0.00	0.00	0.00		
Vicia cassubica	2	---	---	---	1	1	0	0.00	0.00	0.00		
Solanum nigrum	4	---	---	---	1	1	0	0.00	0.00	0.00		
Solidago gigantea	12	4	---	---	1	1	0	0.01	0.00	0.00		
Sorghum halepense	3	---	---	---	0	1	0	0.00	0.00	0.00		
Spergula arvensis	3	---	---	---	0	1	0	0.00	0.00	0.00		
Sonchus oleraceus	6	---	---	---	1	1	0	0.00	0.00	0.00		
Sonchus asper	12	---	3	---	1	1	0	0.00	0.00	0.00		
Sisymbrium loeselii	11	---	4	---	0	1	0	0.00	0.01	0.00		
Sisymbrium officinale	1	---	---	---	0	1	0	0.00	0.00	0.00		
Sisymbrium altissimum	7	---	3	---	0	1	0	0.00	0.00	0.00		
Alyssum repens	7	---	---	4	0	1	1	0.00	0.00	0.01		
Sinapis arvensis	11	---	---	---	1	1	0	0.00	0.00	0.00		
Anagallis minima	1	---	---	---	1	0	0	0.00	0.00	0.00		
Veronica peregrina	1	---	---	---	0	1	0	0.00	0.00	0.00		
Veronica longifolia	6	---	---	---	1	1	0	0.00	0.00	0.00		

	total no. of occurrences	Phi value x 100 (orange: phi >= 0.2)			Constancy in % (green: const. ratio >= 2)			Average cover in % (blue: cover ratio >= 2)			Diagnostic value	Comment
Phytosociological order		B	Fv	S-Fp	B	Fv	S-Fp	B	Fv	S-Fp		
Number of relevés		3470	5016	1472	3470	5016	1472	3470	5016	1472		
Veronica serpyllifolia	20	---	---	---	1	1	0	0.00	0.00	0.00		
Anacamptis coriophora	33	---	---	---	1	1	1	0.01	0.01	0.00		
Aegilops cylindrica	10	---	4	---	0	1	0	0.00	0.00	0.00		
Veronica hederifolia agg.	16	---	---	---	1	1	0	0.01	0.00	0.00		
Veronica polita	4	---	---	---	1	1	0	0.00	0.00	0.00		
Amaranthus powellii	1	---	---	---	0	1	0	0.00	0.00	0.00		
Amaranthus hybridus	1	---	---	---	0	1	0	0.00	0.00	0.00		
Veronica persica	4	---	---	---	1	1	0	0.00	0.00	0.00		
Seseli peucedanoides	10	4	---	---	1	1	0	0.00	0.00	0.00		
Narcissus poeticus	1	---	---	---	0	1	0	0.00	0.00	0.00		
Cerastium tomentosum	1	---	---	---	0	0	1	0.00	0.00	0.00		
Dactylorhiza fuchsii	3	2	---	---	1	0	0	0.00	0.00	0.00		
Cynoglottis barrelieri	23	---	2	---	1	1	1	0.00	0.01	0.00		
Crepis alpestris	2	---	---	---	1	0	1	0.00	0.00	0.00		
Chelidonium majus	15	---	---	---	0	1	1	0.00	0.01	0.01		
Dactylorhiza maculata	9	4	---	---	1	0	0	0.01	0.00	0.00		
Cuscuta europaea	21	---	---	---	1	1	0	0.01	0.01	0.00		
Edraianthus graminifolius	1	---	---	---	0	0	1	0.00	0.00	0.00		
Corydalis solida	9	---	2	---	0	1	1	0.00	0.00	0.00		
Crepis vesicaria	5	3	---	---	1	0	0	0.00	0.00	0.00		
Crepis capillaris	2	---	---	---	1	1	0	0.00	0.00	0.00		
Cimicifuga europaea	2	---	---	---	1	0	1	0.00	0.00	0.00		
Valerianella dentata	62	---	7	---	1	1	1	0.00	0.03	0.00		
Centaurea orientalis	67	---	7	---	1	1	0	0.00	0.04	0.00		
Allium paniculatum	58	---	8	---	1	1	1	0.00	0.02	0.00		
Iris variegata	67	---	4	---	1	1	1	0.03	0.07	0.00		
Rapistrum perenne	81	---	5	---	1	1	0	0.01	0.02	0.00		
Ornithogalum umbellatum	59	---	---	---	1	1	1	0.01	0.01	0.00		
Crocus vernus	8	4	---	---	1	0	0	0.00	0.00	0.00		
Dactylorhiza incarnata	1	---	---	---	1	0	0	0.00	0.00	0.00		
Elymus caninus	6	---	---	---	1	1	1	0.00	0.00	0.00		
Poa versicolor	37	---	6	---	1	1	0	0.00	0.07	0.00		
Polycnemum arvense	34	---	7	---	0	1	0	0.00	0.01	0.00		
Cuscuta approximata	1	---	---	---	0	1	0	0.00	0.00	0.00		
Crepis froelichiana subsp. dinarica	1	---	---	---	1	0	0	0.00	0.00	0.00		
Crepis sancta	2	---	---	---	0	1	0	0.00	0.00	0.00		
Cytisus leiocarpus	3	---	---	---	1	1	1	0.00	0.00	0.00		
Cortusa matthioli	5	---	---	5	0	0	1	0.00	0.00	0.01		
Ceratocephala orthoceras	1	---	---	---	0	1	0	0.00	0.00	0.00		
Cerinthe glabra	1	---	---	---	1	0	0	0.00	0.00	0.00		
Eleocharis palustris agg.	2	---	---	---	0	1	0	0.00	0.00	0.00		
Crepis setosa	33	---	3	---	1	1	0	0.00	0.01	0.00		
Chaerophyllum aromaticum	14	5	---	---	1	0	0	0.01	0.00	0.00		
Cytisus podolicus	9	---	---	---	1	1	0	0.02	0.01	0.00		
Cytisus paczoskii	1	---	---	---	0	1	0	0.00	0.00	0.00		
Cirsium boujartii	1	---	---	---	0	1	0	0.00	0.00	0.00		
Corydalis cava	2	---	---	---	0	1	0	0.00	0.00	0.00		
Cucubalus baccifer	2	---	---	---	1	0	0	0.00	0.00	0.00		
Coronopus squamatus	1	---	---	---	0	1	0	0.00	0.00	0.00		
Cuscuta epilinum	1	---	---	---	0	0	1	0.00	0.00	0.00		
Centaurea stereophylla	6	---	3	---	0	1	0	0.00	0.00	0.00		
Corynephorus canescens	19	---	5	---	0	1	0	0.00	0.01	0.00		
Cuscuta campestris	3	---	---	---	1	1	0	0.00	0.00	0.00		
Crepis tectorum	25	---	4	---	1	1	0	0.00	0.01	0.00		
Elytrigia stipifolia	6	---	3	---	0	1	0	0.00	0.04	0.00		
Chenopodium glaucum	2	---	---	---	0	1	0	0.00	0.00	0.00		
Elytrigia bessarabica	1	---	---	---	0	1	0	0.00	0.01	0.00		
Astragalus cicer	152	10	---	---	3	1	0	0.18	0.04	0.00		

	total no. of occurrences	Phi value x 100 (orange: phi >= 0.2)			Constancy in % (green: const. ratio >= 2)			Average cover in % (blue: cover ratio >= 2)			Diagnostic value	Comment
Phytosociological order		B	Fv	S-Fp	B	Fv	S-Fp	B	Fv	S-Fp		
Number of relevés		3470	5016	1472	3470	5016	1472	3470	5016	1472		
Veronica officinalis	107	9	---	---	2	1	1	0.04	0.01	0.00		
Astragalus glycyphyllos	147	11	---	---	3	1	1	0.07	0.02	0.00		
Caucalis platycarpos	66	---	8	---	1	1	1	0.00	0.02	0.00		
Rhinanthus rumelicus	136	9	---	---	3	1	0	0.16	0.05	0.00		
Glechoma hederacea agg.	102	9	---	---	2	1	1	0.04	0.01	0.00		
Equisetum arvense	146	11	---	---	3	1	0	0.07	0.02	0.00		
Carex muricata agg.	166	7	---	---	3	1	1	0.08	0.03	0.01		
Potentilla reptans	147	13	---	---	3	1	0	0.08	0.02	0.00		
Lathyrus pannonicus	87	7	---	---	2	1	0	0.03	0.01	0.00		
Carthamus lanatus	46	---	5	---	1	1	0	0.00	0.02	0.00		
Spiraea hypericifolia	28	---	6	---	0	1	0	0.00	0.16	0.00		
Scorzoneroides autumnalis	158	12	---	---	3	1	1	0.11	0.02	0.01		
Trifolium ochroleucon	147	11	---	---	3	1	0	0.08	0.02	0.00		
Anacamptis morio	142	10	---	---	3	1	0	0.06	0.02	0.00		
Helictochloa praeusta	136	12	---	---	3	1	1	0.23	0.04	0.00		
Myosotis arvensis	169	8	---	---	3	1	1	0.06	0.03	0.00		
Elymus uralensis	82	---	9	---	1	1	0	0.01	0.23	0.00		
Trifolium striatum	35	---	4	---	1	1	0	0.06	0.10	0.00		
Cirsium arvense	182	12	---	---	4	1	0	0.07	0.02	0.00		
Ephedra distachya	32	---	7	---	0	1	0	0.00	0.06	0.00		
Echium italicum	49	---	8	---	1	1	0	0.00	0.03	0.00		
Peucedanum alsaticum	210	12	---	---	4	1	0	0.14	0.05	0.00		
Phleum pratense agg.	151	13	---	---	3	1	1	0.11	0.02	0.00		
Rumex thyrsiflorus	102	---	4	---	1	1	0	0.02	0.03	0.00		
Tanacetum millefolium	58	---	9	---	1	1	0	0.00	0.15	0.00		
Tanacetum vulgare	57	---	---	---	1	1	0	0.01	0.02	0.00		
Inula britannica	102	---	6	---	1	1	0	0.01	0.04	0.00		
Trifolium aureum	63	5	---	---	1	1	0	0.02	0.01	0.00		
Stachys germanica	121	---	4	---	1	1	0	0.02	0.04	0.00		
Lathyrus nissolia	49	---	---	---	1	1	0	0.01	0.01	0.00		
Melilotus albus	62	---	4	---	1	1	0	0.02	0.03	0.00		
Descurainia sophia	44	---	7	---	1	1	0	0.00	0.02	0.00		
Podospermum canum	45	---	6	---	1	1	0	0.00	0.05	0.00		
Inula germanica	52	---	---	---	1	1	0	0.07	0.02	0.00		
Stipa dasyphylla	39	---	5	---	1	1	1	0.00	0.07	0.03		
Ornithogalum pyramidale	54	3	---	---	1	1	1	0.04	0.01	0.00		
Phragmites australis	59	---	5	---	1	1	0	0.01	0.03	0.00		
Allium rotundum	79	---	8	---	1	1	0	0.01	0.03	0.00		
Echinops sphaerocephalus	46	---	5	---	1	1	1	0.01	0.04	0.00		
Koeleria delavignei	30	---	5	---	1	1	0	0.00	0.06	0.00		
Allium waldsteinii	30	---	6	---	0	1	0	0.00	0.02	0.00		
Lotus maritimus	63	5	---	---	1	1	0	0.03	0.01	0.00		
Stipa tirsa	123	---	3	---	1	1	0	0.42	0.49	0.00		
Allium podolicum	65	---	6	---	1	1	1	0.00	0.06	0.02		
Jurinea stoechadifolia	33	---	7	---	0	1	0	0.00	0.16	0.00		
Gypsophila muralis	35	---	5	---	1	1	0	0.00	0.03	0.00		
Crepis paludosa	1	---	---	---	0	0	1	0.00	0.00	0.00		
Epilobium lanceolatum	1	---	---	---	0	1	0	0.00	0.00	0.00		
Epilobium montanum	16	---	---	4	1	1	1	0.00	0.00	0.01		
Epipactis palustris	3	2	---	---	1	0	0	0.00	0.00	0.00		
Equisetum pratense	8	---	---	---	1	1	0	0.00	0.00	0.00		
Epipactis helleborine	7	---	---	4	0	1	1	0.00	0.00	0.01		
Equisetum sylvaticum	1	---	---	---	0	1	0	0.00	0.00	0.00		
Erodium ciconium	6	---	3	---	0	1	0	0.00	0.00	0.00		
Erucastrum nasturtiifolium	18	---	---	---	1	1	1	0.00	0.00	0.00		
Erodium moschatum	3	---	---	---	0	1	0	0.00	0.00	0.00		
Equisetum telmateia	4	3	---	---	1	0	0	0.01	0.00	0.00		
Equisetum ramosissimum	22	---	4	---	1	1	0	0.00	0.01	0.00		

	total no. of occurrences	Phi value x 100 (orange: phi >= 0.2)			Constancy in % (green: const. ratio >= 2)			Average cover in % (blue: cover ratio >= 2)			Diagnostic value	Comment
Phytosociological order		B	Fv	S-Fp	B	Fv	S-Fp	B	Fv	S-Fp		
Number of relevés		3470	5016	1472	3470	5016	1472	3470	5016	1472		
Eragrostis minor	5	---	3	---	0	1	0	0.00	0.00	0.00		
Epilobium collinum	3	---	---	---	0	1	0	0.00	0.00	0.00		
Erysimum cheiranthoides	15	---	---	---	1	1	1	0.00	0.01	0.00		
Epilobium ciliatum	1	---	---	---	0	1	0	0.00	0.00	0.00		
Epimedium alpinum	1	---	---	---	1	0	0	0.00	0.00	0.00		
Epilobium palustre	1	---	---	---	0	1	0	0.00	0.00	0.00		
Erysimum comatum	18	---	---	4	0	1	1	0.00	0.00	0.01		
Epilobium dodonaei	18	---	---	---	1	1	1	0.00	0.02	0.00		
Equisetum variegatum	8	---	---	---	1	1	0	0.00	0.01	0.00		
Epilobium parviflorum	1	---	---	---	1	0	0	0.00	0.00	0.00		
Epilobium roseum	1	---	---	---	1	0	0	0.00	0.00	0.00		
Epilobium tetragonum	4	---	---	---	1	1	0	0.00	0.00	0.00		
Erigeron glabratus	1	---	---	---	1	0	0	0.00	0.00	0.00		
Euphorbia bessarabica	4	---	---	---	0	1	0	0.00	0.00	0.00		
Centaurea lavrenkoana	4	---	---	---	0	1	0	0.00	0.00	0.00		
Erysimum hieraciifolium agg.	25	---	3	---	1	1	1	0.00	0.01	0.00		
Centaurea paniculata	2	---	---	---	0	1	0	0.00	0.00	0.00		
Erysimum repandum	2	---	---	---	1	1	0	0.00	0.00	0.00		
Euphorbia semivillosa	3	---	---	---	0	1	0	0.00	0.00	0.00		
Eupatorium cannabinum	16	3	---	---	1	1	1	0.01	0.00	0.00		
Euphorbia agraria	17	---	5	---	0	1	0	0.00	0.01	0.00		
Euphorbia falcata	3	---	---	---	1	1	0	0.00	0.00	0.00		
Euphorbia helioscopia	15	---	---	---	1	1	1	0.01	0.00	0.00		
Euphorbia exigua	2	---	---	---	1	0	0	0.00	0.00	0.00		
Erechtites hieraciifolius	1	---	---	---	0	1	0	0.00	0.00	0.00		
Galium sylvaticum	12	---	---	---	1	1	1	0.00	0.00	0.00		
Galium tenuissimum	2	---	---	---	0	1	0	0.00	0.00	0.00		
Galium spurium	11	---	4	---	0	1	0	0.00	0.00	0.00		
Erodium ruthenicum	7	---	3	---	0	1	0	0.00	0.01	0.00		
Galium uliginosum	1	---	---	---	1	0	0	0.00	0.00	0.00		
Centaurea pugioniformis	5	3	---	---	1	0	0	0.01	0.00	0.00		
Eryngium amethystinum	1	---	---	---	1	0	0	0.00	0.00	0.00		
Centaurea pectinata	3	2	---	---	1	0	0	0.00	0.00	0.00		
Erysimum carniolicum	3	---	---	---	1	0	1	0.00	0.00	0.00		
Erysimum sylvestre	4	---	---	4	0	0	1	0.00	0.00	0.00		
Erysimum crepidifolium	18	---	5	---	0	1	0	0.00	0.01	0.00		
Colchicum arenarium	2	---	---	---	0	1	0	0.00	0.00	0.00		
Digitaria ischaemum	3	---	---	---	1	1	0	0.00	0.00	0.00		
Colchicum hungaricum	1	---	---	---	0	1	0	0.00	0.00	0.00		
Dracocephalum austriacum	8	---	---	5	1	1	1	0.00	0.00	0.01		
Draba muralis	6	---	---	---	0	1	1	0.00	0.00	0.00		
Centaurium pulchellum	5	---	---	---	1	1	0	0.00	0.00	0.00		
Digitalis lanata	3	---	---	---	1	1	0	0.00	0.00	0.00		
Doronicum hungaricum	5	---	---	---	1	1	0	0.00	0.00	0.00		
Cirsium waldsteinii	1	---	---	---	1	0	0	0.00	0.00	0.00		
Cirsium oleraceum	9	---	---	---	1	1	0	0.01	0.00	0.00		
Clinopodium suaveolens	1	---	---	---	0	1	0	0.00	0.00	0.00		
Cirsium furiens	20	---	---	---	1	1	1	0.00	0.00	0.00		
Draba nemorosa	10	---	4	---	0	1	0	0.00	0.00	0.00		
Cephalanthera damasonium	5	---	---	3	1	0	1	0.00	0.00	0.00		
Cirsium ukranicum	2	---	---	---	0	1	0	0.00	0.00	0.00		
Clinopodium menthifolium	4	---	---	---	1	0	1	0.00	0.00	0.00		
Cirsium rivulare	1	---	---	---	1	0	0	0.00	0.00	0.00		
Digitalis ferruginea	6	3	---	---	1	0	0	0.00	0.00	0.00		
Digitaria sanguinalis	3	---	---	---	1	1	0	0.00	0.00	0.00		
Coeloglossum viride	5	---	---	---	1	1	0	0.00	0.00	0.00		
Cirsium canum	21	3	---	---	1	1	1	0.01	0.00	0.00		
Crepis pannonica	5	---	---	---	1	1	0	0.00	0.00	0.00		

Phytosociological order	total no. of occurrences	Phi value x 100 (orange: phi >= 0.2)			Constancy in % (green: const. ratio >= 2)			Average cover in % (blue: cover ratio >= 2)			Diagnostic value	Comment
		B	Fv	S-Fp	B	Fv	S-Fp	B	Fv	S-Fp		
Number of relevés		3470	5016	1472	3470	5016	1472	3470	5016	1472		
Centaurea triniifolia	6	---	---	---	0	1	1	0.00	0.00	0.00		
Dioscorea communis	1	---	---	---	1	0	0	0.00	0.00	0.00		
Colchicum versicolor	1	---	---	---	0	1	0	0.00	0.00	0.00		
Conioselinum tataricum	1	---	---	---	0	0	1	0.00	0.00	0.00		
Comandra umbellata	1	---	---	---	0	1	0	0.00	0.00	0.00		
Dipsacus pilosus	2	---	---	---	1	1	0	0.00	0.00	0.00		
Diplotaxis tenuifolia	6	---	---	---	1	1	0	0.00	0.00	0.00		
Diplotaxis muralis	12	---	---	---	1	1	0	0.00	0.00	0.00		
Chaerophyllum temulum	4	---	---	---	1	1	0	0.00	0.00	0.00		
Dipsacus fullonum	9	---	---	---	1	1	0	0.00	0.00	0.00		
Dipsacus laciniatus	15	3	---	---	1	1	0	0.00	0.00	0.00		
Dipsacus sativus	1	---	---	---	1	0	0	0.00	0.00	0.00		
Crepis nicaeensis	10	3	---	---	1	1	0	0.00	0.00	0.00		
Conium maculatum	2	---	---	---	1	1	0	0.00	0.00	0.00		
Cytisus blockianus	9	---	---	---	1	1	0	0.01	0.00	0.00		
Cynoglossum hungaricum	3	---	---	---	0	1	0	0.00	0.00	0.00		
Crepis mollis	4	---	---	---	1	0	1	0.00	0.00	0.00		
Cytisus borysthenicus	1	---	---	---	0	1	0	0.00	0.00	0.00		
Chaerophyllum hirsutum	20	3	---	---	1	0	1	0.01	0.00	0.01		
Cynoglossum germanicum	2	---	---	---	0	1	0	0.00	0.00	0.00		
Crepis pulchra	5	---	3	---	0	1	0	0.00	0.00	0.00		
Comarum palustre	1	---	---	---	1	0	0	0.00	0.00	0.00		
Cypripedium calceolus	9	3	---	---	1	0	1	0.01	0.00	0.00		
Diplotaxis cretacea	1	---	---	---	0	1	0	0.00	0.00	0.00		
Doronicum columnae	6	---	---	5	0	0	1	0.00	0.00	0.01		
Veronica dillenii	89	---	11	---	1	2	0	0.00	0.05	0.00		
Verbascum phlomoides	110	---	10	---	1	2	1	0.00	0.04	0.00		
Campanula bononiensis	204	7	---	---	3	2	1	0.05	0.05	0.00		
Carduus hamulosus	106	---	10	---	1	2	0	0.00	0.03	0.00		
Salvia transylvanica	119	---	9	---	1	2	0	0.01	0.07	0.00		
Vicia villosa	121	---	12	---	1	2	0	0.00	0.10	0.00		
Lathyrus tuberosus	189	8	---	---	3	2	0	0.06	0.05	0.00		
Verbascum thapsus agg.	113	---	8	---	1	2	1	0.01	0.04	0.01		
Pilosella piloselloides	95	---	7	---	1	2	1	0.01	0.02	0.00		
Orlaya grandiflora	134	---	11	---	1	2	1	0.00	0.09	0.00		
Veronica verna	134	---	10	---	1	2	1	0.00	0.06	0.02		
Polygala sibirica	95	---	11	---	1	2	0	0.00	0.06	0.00		
Myosotis ramosissima	157	---	7	---	1	2	1	0.02	0.05	0.01		
Gypsophila paniculata	85	---	10	---	1	2	1	0.00	0.11	0.00		
Crepis foetida	103	---	10	---	1	2	1	0.00	0.05	0.00		
Verbascum nigrum	124	---	5	---	1	2	1	0.02	0.04	0.01		
Ornithogalum kochii	117	---	5	---	1	2	1	0.02	0.04	0.00		
Artemisia pontica	120	---	11	---	1	2	1	0.00	0.13	0.00		
Erigeron canadensis	111	---	11	---	1	2	0	0.00	0.06	0.00		
Klasea radiata	107	---	6	---	1	2	0	0.02	0.05	0.00		
Bromus tectorum	95	---	11	---	0	2	0	0.00	0.10	0.00		
Jasione montana	112	---	11	---	1	2	0	0.00	0.05	0.00		
Hieracium virosum	103	---	11	---	1	2	1	0.00	0.04	0.00		
Artemisia vulgaris	132	4	---	---	2	2	0	0.03	0.04	0.00		
Hypochaeris radicata	146	4	---	---	2	2	1	0.04	0.04	0.00		
Carex hirta	165	5	---	---	2	2	0	0.06	0.08	0.00		
Psephellus marschallianus	82	---	10	---	1	2	0	0.00	0.09	0.00		
Erigeron acris	223	8	---	---	3	2	1	0.06	0.04	0.00		
Anemone sylvestris	237	10	---	---	4	2	1	0.24	0.10	0.01		
Cytisus ruthenicus	212	6	---	---	3	2	0	0.21	0.27	0.00		
Cynoglossum officinale	87	---	8	---	1	2	1	0.00	0.04	0.00		
Papaver dubium	48	---	7	---	1	1	1	0.00	0.02	0.00		
Inula oculus-christi	120	---	8	---	1	2	1	0.01	0.09	0.01		

	total no. of occurrences	Phi value x 100 (orange: phi >= 0.2)			Constancy in % (green: const. ratio >= 2)			Average cover in % (blue: cover ratio >= 2)			Diagnostic value	Comment
Phytosociological order		B	Fv	S-Fp	B	Fv	S-Fp	B	Fv	S-Fp		
Number of relevés		3470	5016	1472	3470	5016	1472	3470	5016	1472		
Lactuca serriola	90	---	9	---	1	2	0	0.00	0.02	0.00		
Brassica elongata	92	---	9	---	1	2	1	0.00	0.04	0.00		
Xeranthemum annuum	111	---	11	---	0	2	1	0.00	0.12	0.00		
Veronica incana	119	---	10	---	1	2	0	0.01	0.09	0.00		
Allium oleraceum	196	7	---	---	3	2	1	0.06	0.04	0.02		
Senecio erucifolius	154	---	4	---	2	2	1	0.04	0.05	0.00		
Agropyron pectinatum	86	---	11	---	0	2	0	0.00	0.14	0.00		
Scleranthus perennis	89	---	9	---	1	2	1	0.00	0.15	0.00		
Scorzonera hispanica	203	6	---	---	3	2	1	0.06	0.04	0.00		
Prunus tenella	94	---	10	---	1	2	0	0.00	0.08	0.00		
Erigeron annuus	248	11	---	---	4	2	1	0.11	0.04	0.00		
Rosa gallica	187	6	---	---	3	2	0	0.11	0.12	0.00		
Crambe tataria	111	---	8	---	1	2	0	0.01	0.07	0.00		
Galatella villosa	110	---	12	---	0	2	0	0.00	0.34	0.00		
Cruciata pedemontana	157	---	12	---	1	3	1	0.00	0.07	0.00		
Marrubium peregrinum	176	---	15	---	1	3	0	0.00	0.12	0.00		
Phlomis tuberosa	192	---	12	---	1	3	0	0.04	0.19	0.00		
Galium octonarium	197	---	16	---	1	4	0	0.00	0.16	0.00		
Oxytropis pilosa	202	---	11	---	1	3	1	0.02	0.09	0.01		
Salvia austriaca	193	---	9	---	1	3	1	0.03	0.07	0.00		
Teucrium polium	173	---	15	---	1	3	0	0.00	0.16	0.00		
Sideritis montana	163	---	13	---	0	3	1	0.00	0.06	0.00		
Seseli tortuosum	168	---	15	---	1	3	0	0.00	0.11	0.00		
Centaurea diffusa	153	---	14	---	1	3	0	0.00	0.07	0.00		
Vicia tetrasperma	253	---	7	---	2	3	0	0.08	0.08	0.00		
Thesium arvense	232	---	11	---	1	4	1	0.04	0.12	0.02		
Stipa lessingiana	220	---	17	---	1	4	0	0.00	1.16	0.00		
Bromus squarrosus	207	---	17	---	1	4	0	0.00	0.14	0.00		
Bromus hordeaceus	223	---	13	---	1	4	1	0.02	0.17	0.00		
Cephalaria uralensis	221	---	15	---	1	4	1	0.00	0.14	0.01		
Linum hirsutum	274	---	10	---	2	4	1	0.05	0.20	0.00		
Carduus acanthoides	268	---	12	---	2	4	1	0.03	0.09	0.00		
Agrostis vinealis	245	---	13	---	1	4	1	0.06	0.39	0.00		
Cynodon dactylon	202	---	13	---	1	4	1	0.01	0.34	0.00		
Viola arvensis	243	---	13	---	1	4	1	0.01	0.09	0.01		
Linaria vulgaris	291	---	7	---	3	4	1	0.05	0.09	0.00		
Muscari tenuiflorum	222	---	13	---	1	4	1	0.01	0.07	0.00		
Eryngium planum	211	---	7	---	2	3	1	0.04	0.09	0.00		
Anthyllis macrocephala	141	---	6	---	1	2	0	0.06	0.08	0.00		
Iris aphylla	141	---	5	---	1	2	1	0.03	0.08	0.06		
Silene chlorantha	78	---	10	---	0	2	0	0.00	0.04	0.00		
Armeria maritima	121	---	9	---	1	2	1	0.03	0.16	0.00		
Geranium columbinum	121	---	8	---	1	2	1	0.01	0.04	0.00		
Lolium perenne	137	---	4	---	1	2	0	0.07	0.10	0.00		
Cirsium vulgare	132	---	4	---	1	2	0	0.03	0.03	0.00		
Dianthus membranaceus	87	---	9	---	1	2	0	0.00	0.05	0.00		
Erodium cicutarium	115	---	12	---	1	2	0	0.00	0.06	0.00		
Asyneuma canescens	111	---	8	---	1	2	1	0.01	0.05	0.00		
Lepidium campestre	139	---	7	---	1	2	1	0.01	0.03	0.01		
Astragalus dasyanthus	91	---	11	---	0	2	0	0.00	0.06	0.00		
Phlomis pungens	126	---	13	---	0	3	0	0.00	0.16	0.00		
Bromus riparius	143	---	12	---	1	3	1	0.02	0.29	0.01		
Vicia hirsuta	239	---	6	---	3	3	1	0.06	0.07	0.00		
Hypericum elegans	197	---	11	---	1	3	1	0.01	0.07	0.00		
Petrorhagia prolifera	159	---	12	---	1	3	1	0.00	0.07	0.01		
Silene latifolia	162	---	10	---	1	3	1	0.01	0.06	0.01		
Carex supina	157	---	13	---	1	3	1	0.00	0.12	0.00		
Veronica orchidea	208	---	6	---	2	3	1	0.04	0.05	0.01		

	total no. of occurrences	Phi value x 100 (orange: phi >= 0.2)			Constancy in % (green: const. ratio >= 2)			Average cover in % (blue: cover ratio >= 2)			Diagnostic value	Comment
Phytosociological order		B	Fv	S-Fp	B	Fv	S-Fp	B	Fv	S-Fp		
Number of relevés		3470	5016	1472	3470	5016	1472	3470	5016	1472		
Ajuga laxmannii	148	---	12	---	1	3	0	0.01	0.06	0.00		
Vicia sativa	323	8	---	---	4	3	0	0.12	0.06	0.00		
Ajuga chamaepitys	131	---	13	---	1	3	0	0.00	0.05	0.00		
Galium aparine	58	---	3	---	1	1	1	0.01	0.02	0.00		
Anthemis ruthenica	33	---	5	---	1	1	0	0.00	0.03	0.00		
Malva thuringiaca	81	---	9	---	1	1	0	0.00	0.04	0.00		
Consolida regalis	62	---	6	---	1	1	0	0.01	0.02	0.00		
Hierochloe odorata	27	---	6	---	0	1	0	0.00	0.03	0.00		
Ranunculus illyricus	79	---	9	---	1	1	1	0.00	0.05	0.00		
Anchusa officinalis	70	---	9	---	1	1	1	0.00	0.04	0.00		
Cerinthe minor	125	5	---	---	2	1	1	0.04	0.03	0.00		
Trifolium dubium	105	8	---	---	2	1	0	0.06	0.02	0.00		
Galium humifusum	52	---	8	---	1	1	0	0.00	0.01	0.00		
Festuca stricta subsp. trachyphylla	50	---	5	---	1	1	0	0.01	0.13	0.00		
Limonium gmelinii	32	---	7	---	0	1	0	0.00	0.02	0.00		
Lotus ucrainicus	59	---	6	---	1	1	0	0.01	0.03	0.00		
Festuca wagneri	29	---	6	---	0	1	0	0.00	0.07	0.00		
Linum linearifolium	36	---	7	---	0	1	0	0.00	0.02	0.00		
Thymelaea passerina	31	---	5	---	1	1	1	0.00	0.01	0.00		
Saxifraga bulbifera	54	---	4	---	1	1	0	0.01	0.03	0.00		
Cardaria draba	63	---	8	---	1	1	0	0.00	0.03	0.00		
Filago arvensis	46	---	8	---	1	1	0	0.00	0.02	0.00		
Poa pannonica	54	---	8	---	0	1	0	0.00	0.17	0.00		
Scleranthus annuus agg.	60	---	8	---	1	1	0	0.00	0.06	0.00		
Bromus sterilis	55	---	5	---	1	1	1	0.00	0.02	0.00		
Marrubium pestalozzae	38	---	7	---	0	1	0	0.00	0.01	0.00		
Galium ruthenicum	69	---	10	---	0	1	0	0.00	0.12	0.00		
Dianthus borbasii	47	---	7	---	1	1	0	0.00	0.02	0.00		
Medicago x varia	122	9	---	---	2	1	0	0.08	0.03	0.00		
Capsella bursa-pastoris	46	---	5	---	1	1	1	0.00	0.02	0.00		
Hyacinthella leucophaea	53	---	8	---	1	1	0	0.00	0.05	0.00		
Cytisus procumbens	72	3	---	---	1	1	1	0.05	0.03	0.03		
Valerianella locusta	75	---	5	---	1	1	1	0.01	0.03	0.00		
Carex stenophylla	40	---	7	---	1	1	0	0.00	0.06	0.00		
Alopecurus pratensis	155	9	---	---	3	1	1	0.09	0.03	0.00		
Fragaria moschata	133	9	---	---	3	1	1	0.10	0.03	0.00		
Clematis integrifolia	60	---	3	---	1	1	0	0.01	0.02	0.00		
Camelina sativa agg.	82	---	8	---	1	1	1	0.00	0.03	0.00		
Festuca dalmatica	34	---	5	---	0	1	1	0.00	0.06	0.01		
Teucrium botrys	33	---	3	---	1	1	1	0.00	0.03	0.01		
Dianthus deltoides	87	8	---	---	2	1	0	0.04	0.01	0.00		
Prunus fruticosa	135	4	---	---	2	1	1	0.06	0.08	0.06		
Pimpinella tragium	36	---	7	---	0	1	0	0.00	0.11	0.00		
Asperula montana	32	---	7	---	0	1	0	0.00	0.02	0.00		
Alyssum murale	34	---	3	---	0	1	1	0.00	0.02	0.01		
Sisymbrium polymorphum	66	---	9	---	1	1	0	0.00	0.03	0.00		
Peucedanum ruthenicum	63	---	9	---	1	1	0	0.00	0.02	0.00		
Dianthus armeria	79	5	---	---	1	1	0	0.02	0.01	0.00		
Agrostis stolonifera	94	9	---	---	2	1	0	0.06	0.02	0.00		
Pontechium maculatum	140	7	---	---	2	1	1	0.04	0.02	0.00		
Centaurium erythraea	130	7	---	---	2	1	0	0.04	0.02	0.00		
Nepeta ucranica	38	---	7	---	0	1	0	0.00	0.02	0.00		
Arabis glabra	94	---	5	---	1	1	1	0.01	0.02	0.01		
Asparagus tenuifolius	29	---	6	---	0	1	0	0.00	0.02	0.00		
Ballota nigra	46	---	6	---	1	1	1	0.01	0.01	0.00		
Lappula squarrosa	79	---	9	---	1	1	1	0.00	0.03	0.00		
Salvia aethiopis	37	---	7	---	1	1	0	0.00	0.01	0.00		
Minuartia hirsuta	34	---	6	---	0	1	1	0.00	0.09	0.00		

	total no. of occurrences	Phi value x 100 (orange: phi >= 0.2)			Constancy in % (green: const. ratio >= 2)			Average cover in % (blue: cover ratio >= 2)			Diagnostic value	Comment
Phytosociological order		B	Fv	S-Fp	B	Fv	S-Fp	B	Fv	S-Fp		
Number of relevés		3470	5016	1472	3470	5016	1472	3470	5016	1472		
Rhinanthus borbasii	32	---	7	---	0	1	0	0.00	0.10	0.00		
Geranium pusillum	44	---	4	---	1	1	1	0.00	0.01	0.00		
Verbena officinalis	62	---	6	---	1	1	0	0.01	0.02	0.00		
Peucedanum arenarium	27	---	5	---	0	1	1	0.00	0.02	0.00		
Thymus serpyllum	73	---	4	---	1	1	1	0.02	0.08	0.01		
Lamium amplexicaule	33	---	6	---	0	1	1	0.00	0.02	0.00		
Lactuca viminea	72	---	7	---	1	1	1	0.00	0.02	0.01		
Nigella arvensis	67	---	9	---	0	1	0	0.00	0.02	0.00		
Calluna vulgaris	69	---	---	---	1	1	1	0.03	0.06	0.01		
Agrostis gigantea	61	4	---	---	1	1	1	0.02	0.03	0.00		
Astragalus asper	42	---	6	---	1	1	0	0.00	0.03	0.00		
Buglossoides arvensis	52	---	5	---	1	1	1	0.00	0.02	0.00		
Chenopodium album agg.	48	---	6	---	1	1	0	0.00	0.01	0.00		
Allium vineale	64	---	---	---	1	1	1	0.02	0.01	0.00		
Viola suavis	32	---	5	---	1	1	0	0.00	0.00	0.00		
Vicia lathyroides	81	---	7	---	1	1	0	0.02	0.03	0.00		
Astragalus exscapus	33	---	6	---	1	1	0	0.00	0.01	0.00		
Psephellus sumensis	30	---	6	---	0	1	0	0.00	0.07	0.00		
Silene viscosa	34	---	4	---	1	1	1	0.00	0.02	0.00		
Psephellus trinervius	32	---	7	---	0	1	0	0.00	0.06	0.00		
Arenaria procera	38	---	4	---	1	1	0	0.01	0.01	0.00		
Linaria angustissima	45	---	5	---	1	1	1	0.00	0.02	0.00		
Herniaria polygama	55	---	9	---	0	1	0	0.00	0.04	0.00		
Potentilla astracanica	41	---	7	---	0	1	0	0.00	0.02	0.00		
Carduus crispus	35	---	5	---	1	1	0	0.00	0.01	0.00		
Setaria viridis	67	---	9	---	1	1	0	0.00	0.03	0.00		
Hesperis tristis	46	---	8	---	1	1	0	0.00	0.02	0.00		
Orobanche alba	75	---	5	---	1	1	1	0.01	0.02	0.00		
Alyssum rostratum	35	---	7	---	0	1	0	0.00	0.01	0.00		
Herniaria incana	47	---	8	---	0	1	0	0.00	0.02	0.00		
Bromus arvensis	48	---	4	---	1	1	1	0.00	0.02	0.00		
Herniaria glabra	44	---	7	---	1	1	0	0.00	0.02	0.00		
Silene bupleuroides	84	---	7	---	1	1	1	0.01	0.03	0.01		
Bassia prostrata	75	---	9	---	1	1	0	0.00	0.12	0.00		
Bromus japonicus	63	---	8	---	1	1	0	0.00	0.03	0.00		
Convolvulus lineatus	60	---	9	---	0	1	0	0.00	0.05	0.00		
Anagallis arvensis	52	---	5	---	1	1	0	0.00	0.01	0.00		
Ambrosia artemisiifolia	31	---	5	---	1	1	0	0.00	0.01	0.00		
Potentilla humifusa	70	---	9	---	1	1	0	0.00	0.07	0.00		
Oenothera biennis agg.	60	---	9	---	1	1	0	0.00	0.03	0.00		
Campanula cespitosa	1	---	---	---	1	0	0	0.00	0.00	0.00		
Koeleria moldavica	4	---	---	---	0	1	0	0.00	0.00	0.00		
Iris spuria	7	---	---	---	1	1	0	0.00	0.00	0.00		
Juncus articulatus	2	---	---	---	1	1	0	0.00	0.00	0.00		
Juncus compressus	9	---	---	---	1	1	0	0.01	0.00	0.00		
Koeleria brevis	10	---	4	---	0	1	0	0.00	0.01	0.00		
Malva alcea	3	2	---	---	1	0	0	0.00	0.00	0.00		
Bromus rigidus	2	---	---	---	0	1	0	0.00	0.00	0.00		
Jovibarba heuffelii	7	---	---	---	0	1	1	0.00	0.00	0.00		
Koeleria grandis	5	---	---	---	1	1	0	0.00	0.00	0.00		
Campanula cervicaria	16	5	---	---	1	1	0	0.01	0.00	0.00		
Juncus conglomeratus	2	---	---	---	1	0	0	0.00	0.00	0.00		
Lactuca quercina	4	---	---	---	0	1	0	0.00	0.00	0.00		
Lactuca saligna	11	---	---	---	1	1	0	0.00	0.00	0.00		
Koeleria talievii	1	---	---	---	0	1	0	0.00	0.00	0.00		
Isatis praecox	3	---	---	---	0	1	1	0.00	0.00	0.00		
Campanula alpina	1	---	---	---	0	0	1	0.00	0.00	0.00		
Klasea erucifolia	1	---	---	---	0	1	0	0.00	0.00	0.00		

	total no. of occurrences	Phi value x 100 (orange: phi >= 0.2)			Constancy in % (green: const. ratio >= 2)			Average cover in % (blue: cover ratio >= 2)			Diagnostic value	Comment
Phytosociological order		B	Fv	S-Fp	B	Fv	S-Fp	B	Fv	S-Fp		
Number of relevés		3470	5016	1472	3470	5016	1472	3470	5016	1472		
Klasea lycopifolia	18	4	---	---	1	1	0	0.03	0.00	0.00		
Calepina irregularis	1	---	---	---	0	1	0	0.00	0.00	0.00		
Campanula abietina	2	---	---	3	0	0	1	0.00	0.00	0.00		
Knautia dipsacifolia	21	3	---	---	1	1	1	0.02	0.00	0.01		
Kickxia spuria	2	---	---	---	1	1	0	0.00	0.00	0.00		
Limonium aureum	3	---	---	---	1	1	0	0.00	0.00	0.00		
Dianthus pallens	4	---	---	---	0	1	0	0.00	0.00	0.00		
Limonium tomentellum	5	---	3	---	0	1	0	0.00	0.00	0.00		
Dianthus platyodon	3	---	---	---	0	1	0	0.00	0.03	0.00		
Geranium molle	6	---	3	---	0	1	0	0.00	0.00	0.00		
Bromus secalinus	3	---	---	---	0	1	0	0.00	0.00	0.00		
Bromus commutatus	9	---	---	---	1	1	0	0.01	0.00	0.00		
Linum trigynum	1	---	---	---	1	0	0	0.00	0.00	0.00		
Calendula officinalis	1	---	---	---	1	0	0	0.00	0.00	0.00		
Lithospermum officinale	31	---	---	---	1	1	0	0.01	0.01	0.00		
Lunaria rediviva	1	---	---	---	1	0	0	0.00	0.00	0.00		
Linum ucranicum	16	---	5	---	0	1	0	0.00	0.04	0.00		
Dianthus leptopetalus	2	---	---	---	0	1	0	0.00	0.00	0.00		
Linaria dulcis	1	---	---	---	0	1	0	0.00	0.00	0.00		
Linaria biebersteinii	12	---	4	---	0	1	0	0.00	0.00	0.00		
Bromus racemosus	7	---	---	---	1	1	0	0.00	0.00	0.00		
Linum nervosum	22	4	---	---	1	1	0	0.01	0.00	0.00		
Linaria x kocianovichii	16	---	3	---	1	1	0	0.00	0.01	0.00		
Limonium platyphyllum	7	---	3	---	0	1	0	0.00	0.00	0.00		
Dianthus nitidus	6	---	---	5	0	0	1	0.00	0.00	0.01		
Dianthus hypanicus	4	---	---	---	0	1	0	0.00	0.00	0.00		
Linum dolomiticum Borb.	4	---	---	4	0	0	1	0.00	0.00	0.05		
Lilium bulbiferum	17	6	---	---	1	0	0	0.01	0.00	0.00		
Lilium carniolicum	4	3	---	---	1	0	0	0.00	0.00	0.00		
Campanula scheuchzeri	4	3	---	---	1	0	0	0.00	0.00	0.00		
Campanula rapunculus	39	---	---	---	1	1	1	0.01	0.01	0.00		
Hyssopus cretaceus	1	---	---	---	0	1	0	0.00	0.02	0.00		
Campanula thyrsoides	3	---	---	---	1	0	1	0.00	0.00	0.00		
Impatiens parviflora	3	---	---	---	1	1	1	0.00	0.00	0.00		
Campanula macrostachya	9	---	---	---	1	1	0	0.00	0.00	0.00		
Campanula latifolia	4	---	---	---	1	1	0	0.00	0.00	0.00		
Iris halophila	1	---	---	---	0	1	0	0.00	0.00	0.00		
Iberis pinnata	1	---	---	---	0	1	0	0.00	0.00	0.00		
Inula helenium	4	---	---	---	1	1	0	0.00	0.00	0.00		
Campanula lingulata	1	---	---	---	0	1	0	0.00	0.00	0.00		
Campanula serrata	12	3	---	---	1	0	1	0.01	0.00	0.00		
Knautia x posoniensis	2	---	---	---	1	0	0	0.00	0.00	0.00		
Hypericum tetrapterum	4	3	---	---	1	0	0	0.00	0.00	0.00		
Calystegia sepium	3	2	---	---	1	0	0	0.00	0.00	0.00		
Cardamine amara	4	---	---	3	0	1	1	0.00	0.00	0.01		
Camelina rumelica	1	---	---	---	0	1	0	0.00	0.00	0.00		
Hypochaeris glabra	8	---	---	4	1	1	1	0.00	0.00	0.01		
Homogyne alpina	1	---	---	---	0	0	1	0.00	0.00	0.00		
Hypochaeris uniflora	2	---	---	---	1	0	0	0.00	0.00	0.00		
Hyparrhenia hirta	2	---	---	---	1	1	0	0.00	0.00	0.00		
Hyoscyamus niger	4	---	---	---	0	1	0	0.00	0.00	0.00		
Hypericum hirsutum	16	5	---	---	1	0	1	0.01	0.00	0.00		
Inula spiraeifolia	14	---	4	---	0	1	0	0.00	0.02	0.00		
Gymnadenia borealis	1	---	---	---	1	0	0	0.01	0.00	0.00		
Helianthus pauciflorus	1	---	---	---	0	0	1	0.00	0.00	0.00		
Gymnadenia odoratissima	10	---	---	5	1	0	1	0.00	0.00	0.01		
Jurinea salicifolia	8	---	3	---	0	1	0	0.00	0.01	0.00		
Juncus subnodulosus	1	---	---	---	1	0	0	0.00	0.00	0.00		

	total no. of occurrences	Phi value x 100 (orange: phi >= 0.2)			Constancy in % (green: const. ratio >= 2)			Average cover in % (blue: cover ratio >= 2)			Diagnostic value	Comment
Phytosociological order		B	Fv	S-Fp	B	Fv	S-Fp	B	Fv	S-Fp		
Number of relevés		3470	5016	1472	3470	5016	1472	3470	5016	1472		
Helictochloa adsurgens	3	2	---	---	1	0	0	0.00	0.00	0.00		
Carduus personata	1	---	---	---	1	0	0	0.00	0.00	0.00		
Gypsophila repens	5	---	---	---	1	0	1	0.01	0.00	0.00		
Gypsophila thyraica	28	---	5	---	1	1	1	0.00	0.04	0.00		
Gypsophila petraea	3	---	---	4	0	0	1	0.00	0.00	0.00		
Gypsophila oligosperma	14	---	4	---	1	1	0	0.00	0.03	0.00		
Iva xanthiifolia	2	---	---	---	0	1	0	0.00	0.00	0.00		
Iris reichenbachii	1	---	---	---	0	1	0	0.00	0.00	0.00		
Iris ruthenica	2	---	---	---	1	0	1	0.00	0.00	0.00		
Iris pontica	6	---	---	---	1	1	0	0.00	0.00	0.00		
Campanula crassipes	5	---	---	4	0	1	1	0.00	0.00	0.01		
Iris graminea	12	4	---	---	1	1	0	0.01	0.00	0.00		
Juncus gerardii	5	---	3	---	0	1	0	0.00	0.00	0.00		
Juncus inflexus	1	---	---	---	1	0	0	0.00	0.00	0.00		
Jurinea multiflora	17	---	5	---	0	1	0	0.00	0.01	0.00		
Juncus tenuis	1	---	---	---	1	0	0	0.00	0.00	0.00		
Jurinea cyanoides	3	---	---	---	0	1	0	0.00	0.00	0.00		
Juncus effusus	1	---	---	---	1	0	0	0.00	0.00	0.00		
Laserpitium prutenicum	1	---	---	---	1	0	0	0.00	0.00	0.00		
Noccaea kovatsii	1	---	---	---	0	1	0	0.00	0.00	0.00		
Noccaea goesingensis	1	---	---	---	1	0	0	0.00	0.00	0.00		
Noccaea caerulescens	15	5	---	---	1	1	0	0.01	0.00	0.00		
Atriplex prostrata	2	---	---	---	0	1	0	0.00	0.00	0.00		
Myosotis scorpioides agg.	2	---	---	---	1	0	0	0.00	0.00	0.00		
Milium vernale	1	---	---	---	0	1	0	0.00	0.00	0.00		
Avenella flexuosa	34	---	3	---	1	1	1	0.02	0.05	0.00		
Minuartia taurica	5	---	3	---	0	1	0	0.00	0.01	0.00		
Avena fatua	1	---	---	---	0	1	0	0.00	0.00	0.00		
Minuartia glomerata	13	---	1	---	0	1	1	0.00	0.00	0.00		
Muscari botryoides	18	---	4	---	1	1	0	0.00	0.01	0.00		
Neottia nidus-avis	3	---	---	---	1	0	1	0.00	0.00	0.00		
Delphinium elatum	2	---	---	3	0	0	1	0.00	0.00	0.00		
Dianthus barbatus	9	4	---	---	1	0	0	0.01	0.00	0.00		
Dianthus speciosus	1	---	---	---	0	1	0	0.00	0.00	0.00		
Dactylorhiza majalis	1	---	---	---	1	0	0	0.00	0.00	0.00		
Cerastium glomeratum	19	---	---	---	1	1	0	0.00	0.00	0.00		
Cephalaria transsylvanica	24	---	6	---	0	1	0	0.00	0.02	0.00		
Astrantia carniolica	1	---	---	---	1	0	0	0.00	0.00	0.00		
Nepeta cataria	2	---	---	---	1	1	0	0.00	0.00	0.00		
Noccaea jankae	19	---	3	---	1	1	0	0.00	0.01	0.00		
Laserpitium archangelica	7	---	---	6	0	0	1	0.00	0.00	0.01		
Neslia paniculata	4	---	---	---	0	1	0	0.00	0.00	0.00		
Minuartia hybrida	4	---	---	---	0	1	0	0.00	0.00	0.00		
Narcissus pseudonarcissus	2	---	---	---	0	1	0	0.00	0.00	0.00		
Atriplex oblongifolia	3	---	---	---	0	1	0	0.00	0.00	0.00		
Botrychium lunaria	27	---	---	---	1	1	1	0.00	0.01	0.01		
Malabaila graveolens	6	---	3	---	0	1	0	0.00	0.00	0.00		
Malaxis monophyllos	2	---	---	---	1	0	0	0.00	0.00	0.00		
Myosotis sparsiflora	3	---	---	---	0	1	0	0.00	0.00	0.00		
Marrubium vulgare	14	---	4	---	0	1	0	0.00	0.00	0.00		
Medicago arabica	1	---	---	---	1	0	0	0.00	0.00	0.00		
Matthiola fragrans	1	---	---	---	0	1	0	0.00	0.00	0.00		
Myosotis alpestris	1	---	---	---	0	0	1	0.00	0.00	0.00		
Atriplex sagittata	5	---	3	---	0	1	0	0.00	0.02	0.00		
Maianthemum bifolium	2	---	---	---	1	0	0	0.00	0.00	0.00		
Myosotis discolor	23	---	3	---	1	1	0	0.00	0.01	0.00		
Medicago monspeliaca	4	---	---	---	0	1	0	0.00	0.00	0.00		
Microrrhinum minus	11	---	---	---	1	1	1	0.00	0.00	0.00		

	total no. of occurrences	Phi value x 100 (orange: phi >= 0.2)			Constancy in % (green: const. ratio >= 2)			Average cover in % (blue: cover ratio >= 2)			Diagnostic value	Comment
Phytosociological order		B	Fv	S-Fp	B	Fv	S-Fp	B	Fv	S-Fp		
Number of relevés		3470	5016	1472	3470	5016	1472	3470	5016	1472		
Barbarea vulgaris	17	---	3	---	1	1	1	0.00	0.01	0.00		
Milium effusum	1	---	---	---	0	1	0	0.00	0.00	0.00		
Blackstonia perfoliata	2	---	---	---	0	1	0	0.00	0.00	0.00		
Lythrum salicaria	3	---	---	---	1	1	0	0.00	0.00	0.00		
Lythrum virgatum	2	---	---	---	1	1	0	0.00	0.00	0.00		
Bidens tripartitus	1	---	---	---	1	0	0	0.00	0.00	0.00		
Melampyrum alboffianum	1	---	---	---	0	1	0	0.00	0.00	0.00		
Bifora radians	1	---	---	---	0	1	0	0.00	0.00	0.00		
Dianthus collinus	11	---	---	---	1	1	0	0.00	0.00	0.00		
Dianthus capitatus	14	---	4	---	0	1	0	0.00	0.01	0.00		
Dianthus campestris	10	---	4	---	0	1	0	0.00	0.01	0.00		
Lychnis coronaria	13	---	2	---	1	1	0	0.00	0.00	0.00		
Lysimachia vulgaris	7	---	---	---	1	1	0	0.00	0.00	0.00		
Dianthus carbonatus	12	---	4	---	0	1	0	0.00	0.01	0.00		
Dianthus fischeri	16	---	2	---	1	1	0	0.00	0.00	0.00		
Dianthus guttatus	1	---	---	---	0	1	0	0.00	0.00	0.00		
Dianthus eugeniae	5	---	3	---	0	1	0	0.00	0.00	0.00		
Cephalaria laevigata	1	---	---	---	0	1	0	0.00	0.00	0.00		
Cephalanthera rubra	7	---	---	3	1	1	1	0.00	0.00	0.00		
Lythrum hyssopifolia	1	---	---	---	0	1	0	0.00	0.00	0.00		
Lotus angustissimus	6	---	---	---	1	1	0	0.00	0.00	0.00		
Bromus cappadocica	1	---	---	---	0	1	0	0.00	0.00	0.00		
Lotus pedunculatus	1	---	---	---	1	0	0	0.00	0.00	0.00		
Lotus tenuis	18	---	---	---	1	1	0	0.00	0.00	0.00		
Lupinus polyphyllus	1	---	---	---	0	1	0	0.00	0.00	0.00		
Luzula luzulina	3	2	---	---	1	0	0	0.00	0.00	0.00		
Bromus benekenii	1	---	---	---	0	1	0	0.00	0.00	0.00		
Lycopsis arvensis	1	---	---	---	0	1	0	0.00	0.00	0.00		
Luzula sylvatica	1	---	---	---	0	0	1	0.00	0.00	0.00		
Luzula luzuloides	24	2	---	---	1	1	1	0.02	0.00	0.01		
Luzula pilosa	4	---	---	---	1	1	0	0.00	0.00	0.00		
Dianthus henteri	6	---	3	---	0	1	0	0.00	0.00	0.00		
Cephalanthera longifolia	3	2	---	---	1	0	0	0.00	0.00	0.00		
Dianthus moravicus	6	---	---	4	0	1	1	0.00	0.00	0.01		
Dianthus pseudobarbatus	9	---	3	---	0	1	0	0.00	0.01	0.00		
Dichoropetalum schottii	3	2	---	---	1	0	0	0.00	0.00	0.00		
Dianthus pseudarmeria	23	---	6	---	0	1	0	0.00	0.01	0.00		
Dianthus sylvestris	1	---	---	---	1	0	0	0.00	0.00	0.00		
Dactylorhiza sambucina	8	4	---	---	1	0	0	0.00	0.00	0.00		
Dasypyrum villosum	2	---	---	---	0	1	0	0.00	0.00	0.00		
Dianthus pseudoserotinus	1	---	---	---	0	1	0	0.00	0.00	0.00		
Dianthus tenuifolius	3	---	---	4	0	0	1	0.00	0.00	0.00		
Dianthus superbus	4	3	---	---	1	0	0	0.00	0.00	0.00		
Dichoropetalum carvifolia	12	1	---	---	1	1	1	0.00	0.00	0.00		
Elytrigia elongata	8	---	3	---	0	1	0	0.00	0.01	0.00		
Centaurea solstitialis	10	---	---	---	1	1	0	0.00	0.00	0.00		
Dryopteris filix-mas	3	---	---	---	0	1	1	0.00	0.00	0.00		
Echinophora sibthorpiana	2	---	---	---	1	1	0	0.00	0.00	0.00		
Dryas octopetala	1	---	---	---	0	0	1	0.00	0.00	0.05		
Echium russicum	22	---	---	---	1	1	0	0.01	0.02	0.00		
Dracocephalum thymiflorum	1	---	---	---	0	1	0	0.00	0.00	0.00		
Cerastium banaticum	4	---	---	---	0	1	1	0.00	0.00	0.00		
Dracocephalum ruyschiana	2	---	---	---	0	1	0	0.00	0.02	0.00		
Echinops exaltatus	1	---	---	---	0	1	0	0.00	0.00	0.00		
Echinops banaticus	7	---	3	---	0	1	0	0.00	0.00	0.00		
Epilobium angustifolium	16	---	---	---	1	1	1	0.00	0.01	0.01		
Gagea pratensis	1	---	---	---	0	1	0	0.00	0.00	0.00		
Gagea minima	16	---	3	---	0	1	1	0.00	0.01	0.00		

	total no. of occurrences	Phi value x 100 (orange: phi >= 0.2)			Constancy in % (green: const. ratio >= 2)			Average cover in % (blue: cover ratio >= 2)			Diagnostic value	Comment
Phytosociological order		B	Fv	S-Fp	B	Fv	S-Fp	B	Fv	S-Fp		
Number of relevés		3470	5016	1472	3470	5016	1472	3470	5016	1472		
Galanthus nivalis	1	---	---	---	0	1	0	0.00	0.00	0.00		
Fumaria vaillantii	4	---	---	---	0	1	1	0.00	0.00	0.00		
Gagea pusilla	10	---	---	---	1	1	1	0.00	0.00	0.00		
Galium odoratum	3	---	---	---	1	1	0	0.00	0.00	0.00		
Geranium lucidum	6	---	---	---	0	1	1	0.00	0.00	0.00		
Galium rivale	1	---	---	---	0	1	0	0.00	0.00	0.00		
Fumaria officinalis	11	---	3	---	1	1	0	0.00	0.00	0.00		
Gentianella fatrae	5	---	---	5	0	0	1	0.00	0.00	0.01		
Gagea transversalis	2	---	---	---	0	1	0	0.00	0.00	0.00		
Cyanus segetum	6	---	---	---	1	1	0	0.01	0.00	0.00		
Cyanus adscendens	4	3	---	---	1	0	0	0.00	0.00	0.00		
Cymbaria borysthenica	5	---	3	---	0	1	0	0.00	0.00	0.00		
Festuca vaginata	13	---	4	---	0	1	0	0.00	0.02	0.00		
Cynanchum acutum	2	---	---	---	0	1	0	0.00	0.00	0.00		
Crypsis aculeata	1	---	---	---	0	1	0	0.00	0.00	0.00		
Cyclamen purpurascens	7	---	---	---	1	0	1	0.00	0.00	0.01		
Galatella sedifolia	6	---	---	---	1	1	0	0.00	0.00	0.00		
Chenopodium ficifolium	1	---	---	---	0	1	0	0.00	0.00	0.00		
Chrysanthemum zawadskii	2	---	---	3	0	0	1	0.00	0.00	0.02		
Crupina vulgaris	22	---	4	---	1	1	1	0.00	0.01	0.00		
Geranium macrorrhizum	2	---	---	3	0	0	1	0.00	0.00	0.00		
Foeniculum vulgare	3	2	---	---	1	0	0	0.00	0.00	0.00		
Carex divisa	12	---	3	---	1	1	0	0.00	0.01	0.00		
Carex pilosa	4	3	---	---	1	0	0	0.00	0.00	0.00		
Galium intermedium	1	---	---	---	0	1	0	0.00	0.00	0.00		
Galium kitaibelianum	2	---	---	3	0	0	1	0.00	0.00	0.00		
Gentiana pneumonanthe	4	---	---	---	1	1	0	0.00	0.00	0.00		
Filipendula ulmaria	2	---	---	---	1	1	0	0.00	0.00	0.00		
Filago germanica	29	---	---	---	1	1	1	0.01	0.01	0.00		
Filago lutescens	7	---	3	---	0	1	0	0.00	0.00	0.00		
Carex pediformis	5	---	---	---	1	1	1	0.00	0.00	0.00		
Fumana vulgaris	5	3	---	---	1	0	0	0.01	0.00	0.00		
Galium flavescens	1	---	---	---	0	1	0	0.00	0.00	0.00		
Carex leporina	3	2	---	---	1	0	0	0.00	0.00	0.00		
Galium verrucosum	3	---	---	---	1	1	0	0.00	0.00	0.00		
Carex diluta	1	---	---	---	0	1	0	0.00	0.00	0.00		
Geranium collinum	2	---	---	---	1	0	0	0.00	0.00	0.00		
Gentianella austriaca	10	1	---	---	1	0	1	0.00	0.00	0.00		
Carex hallerana	17	5	---	---	1	1	0	0.03	0.00	0.00		
Carex nigra	1	---	---	---	0	1	0	0.00	0.00	0.00		
Carex melanostachya	13	---	4	---	1	1	0	0.00	0.07	0.00		
Carex fritschii	1	---	---	---	0	1	0	0.00	0.00	0.00		
Galium volhynicum	4	---	---	---	0	1	0	0.00	0.00	0.00		
Gaudinia fragilis	1	---	---	---	1	0	0	0.00	0.00	0.00		
Euphorbia villosa	15	4	---	---	1	1	0	0.01	0.00	0.00		
Equisetum hyemale	5	---	---	---	1	1	0	0.00	0.00	0.00		
Euphrasia parviflora	6	---	---	---	1	1	0	0.00	0.00	0.00		
Ferula heuffelii	3	---	---	---	0	1	1	0.00	0.00	0.00		
Euphrasia nemorosa agg.	1	---	---	---	1	0	0	0.00	0.00	0.00		
Equisetum palustre	4	---	---	---	1	1	0	0.00	0.00	0.00		
Festuca amethystina	4	---	---	3	1	0	1	0.00	0.00	0.01		
Festuca altissima	1	---	---	---	1	0	0	0.00	0.00	0.00		
Fallopia dumetorum	23	---	3	---	1	1	1	0.00	0.01	0.00		
Carex sylvatica	7	4	---	---	1	0	0	0.00	0.00	0.00		
Ferulago campestris	16	---	4	---	1	1	0	0.00	0.01	0.00		
Carex vulpina agg.	9	---	---	---	1	1	0	0.00	0.00	0.00		
Sporobolus vaginiflorus	4	---	---	---	1	1	0	0.01	0.03	0.00		
Stachys alopecuros	4	3	---	---	1	0	0	0.01	0.00	0.00		

	total no. of occurrences	Phi value x 100 (orange: phi >= 0.2)			Constancy in % (green: const. ratio >= 2)			Average cover in % (blue: cover ratio >= 2)			Diagnostic value	Comment
Phytosociological order		B	Fv	S-Fp	B	Fv	S-Fp	B	Fv	S-Fp		
Number of relevés		3470	5016	1472	3470	5016	1472	3470	5016	1472		
Euphorbia palustris	4	---	---	---	1	1	0	0.00	0.00	0.00		
Euphorbia stricta	31	2	---	---	1	1	1	0.01	0.00	0.00		
Noccaea macrantha	1	---	---	---	0	1	0	0.00	0.00	0.00		
Spiranthes spiralis	2	---	---	---	1	0	0	0.00	0.00	0.00		
Euphorbia platyphyllos	18	5	---	---	1	1	0	0.00	0.00	0.00		
Euphorbia nutans	1	---	---	---	0	1	0	0.00	0.00	0.00		
Euphorbia leptocaula	6	---	3	---	0	1	0	0.00	0.00	0.00		
Centaurea arenaria agg.	10	---	---	---	1	1	0	0.00	0.00	0.00		
Euphorbia salicifolia	9	---	---	---	1	1	0	0.00	0.00	0.00		
Centaurea besseriana	1	---	---	---	0	1	0	0.00	0.00	0.00		
Galeopsis angustifolia	20	---	---	---	1	1	1	0.01	0.01	0.00		
Carex pulicaris	2	---	---	---	1	0	0	0.00	0.00	0.00		
Gagea bulbifera	4	---	---	---	0	1	0	0.00	0.00	0.00		
Gagea lutea	1	---	---	---	0	1	0	0.00	0.00	0.00		
Galium abaujense	8	---	---	---	1	1	0	0.00	0.00	0.00		
Carex paniculata	1	---	---	---	1	0	0	0.00	0.00	0.00		
Carex pilulifera	15	5	---	---	1	0	0	0.01	0.00	0.00		
Festuca versicolor	2	---	---	3	0	0	1	0.00	0.00	0.00		
Galeopsis pubescens	8	---	---	---	1	1	1	0.00	0.00	0.00		
Fritillaria ruthenica	1	---	---	---	0	1	0	0.00	0.00	0.00		
Galeopsis ladanum	19	---	---	---	1	1	1	0.00	0.02	0.00		
Fumaria schleicheri	4	---	---	---	1	1	0	0.00	0.00	0.00		
Carex riparia	2	---	---	---	0	1	0	0.00	0.00	0.00		
Festuca beckeri	14	---	4	---	0	1	0	0.00	0.04	0.00		
Carlina acanthifolia	23	3	---	---	1	1	1	0.03	0.01	0.00		
Carex strigosa	1	---	---	---	1	0	0	0.00	0.00	0.00		
Festuca heterophylla	14	2	---	---	1	1	1	0.00	0.00	0.00		
Galium cracoviense	7	---	---	---	1	1	0	0.00	0.01	0.00		
Galeopsis tetrahit	2	---	---	---	1	1	0	0.00	0.00	0.00		
Gagea bohemica	27	---	4	---	0	1	1	0.00	0.02	0.00		
Galega officinalis	3	2	---	---	1	0	0	0.00	0.00	0.00		
Galinsoga parviflora	1	---	---	---	0	1	0	0.00	0.00	0.00		
Carex obtusata	1	---	---	---	0	1	0	0.00	0.00	0.00		
Gentianella amarella	7	---	---	---	1	0	1	0.00	0.00	0.00		
Mentha longifolia	14	3	---	---	1	1	0	0.01	0.00	0.00		
Bartsia alpina	1	---	---	---	0	0	1	0.00	0.00	0.00		
Malva moschata	2	---	---	---	1	0	0	0.00	0.00	0.00		
Malva neglecta	1	---	---	---	0	1	0	0.00	0.00	0.00		
Malva sylvestris	1	---	---	---	1	0	0	0.00	0.00	0.00		
Melilotus sulcatus	5	---	---	4	0	1	1	0.00	0.00	0.01		
Carduus carduelis	1	---	---	---	0	1	0	0.00	0.00	0.00		
Helictotrichon desertorum	9	---	---	---	1	1	0	0.07	0.01	0.00		
Bassia scoparia	3	2	---	---	1	0	0	0.00	0.00	0.00		
Melissa officinalis	3	---	---	---	1	1	0	0.00	0.00	0.00		
Bassia laniflora	9	---	3	---	0	1	0	0.00	0.00	0.00		
Mentha arvensis	12	---	---	---	1	1	0	0.00	0.00	0.00		
Melica altissima	3	---	---	---	0	1	0	0.00	0.00	0.00		
Mentha spicata	1	---	---	---	0	1	0	0.00	0.00	0.00		
Geranium pyrenaicum	1	---	---	---	0	1	0	0.00	0.00	0.00		
Geranium rotundifolium	15	---	2	---	0	1	1	0.00	0.00	0.00		
Geranium phaeum	1	---	---	---	0	1	0	0.00	0.00	0.00		
Mentha x dumetorum	1	---	---	---	0	1	0	0.00	0.00	0.00		
Melampyrum pratense	22	2	---	---	1	1	1	0.02	0.00	0.00		
Melampyrum bihariense	3	---	---	---	1	0	1	0.00	0.00	0.00		
Melampyrum polonicum	6	---	---	---	1	1	0	0.00	0.01	0.00		
Mentha pulegium	2	---	---	---	0	1	0	0.00	0.00	0.00		
Melica uniflora	10	---	---	---	1	1	1	0.00	0.00	0.01		
Helleborus odorus	24	4	---	---	1	1	0	0.02	0.00	0.00		

	total no. of occurrences	Phi value x 100 (orange: phi >= 0.2)			Constancy in % (green: const. ratio >= 2)			Average cover in % (blue: cover ratio >= 2)			Diagnostic value	Comment
Phytosociological order		B	Fv	S-Fp	B	Fv	S-Fp	B	Fv	S-Fp		
Number of relevés		3470	5016	1472	3470	5016	1472	3470	5016	1472		
Hypericum montanum	19	---	---	---	1	1	1	0.01	0.00	0.00		
Cannabis sativa	3	---	---	---	0	1	0	0.00	0.00	0.00		
Hirschfeldia incana	1	---	---	---	1	0	0	0.00	0.00	0.00		
Cardamine bulbifera	3	---	---	---	1	0	1	0.00	0.00	0.00		
Hippuris vulgaris	1	---	---	---	1	0	0	0.01	0.00	0.00		
Humulus lupulus	5	---	---	---	0	1	1	0.00	0.00	0.00		
Hypericum richeri subsp. grisebachii	1	---	---	---	1	0	0	0.00	0.00	0.00		
Knautia fleischmanii	1	---	---	---	1	0	0	0.00	0.00	0.00		
Holcus mollis	5	---	---	---	1	1	0	0.00	0.00	0.00		
Hordeum murinum	3	---	---	---	0	1	0	0.00	0.00	0.00		
Cardamine hirsuta	1	---	---	---	1	0	0	0.00	0.00	0.00		
Helictochloa compressa	8	---	---	---	1	1	0	0.00	0.00	0.00		
Hesperis sylvestris	3	---	---	---	0	1	0	0.00	0.02	0.00		
Bellevalia speciosa	12	---	4	---	0	1	0	0.00	0.01	0.00		
Helminthotheca echioides	6	3	---	---	1	1	0	0.00	0.00	0.00		
Hieracium transylvanicum	2	---	---	3	0	0	1	0.00	0.00	0.00		
Cardamine pratensis agg.	16	5	---	---	1	1	0	0.01	0.00	0.00		
Melica picta	3	---	---	---	1	1	1	0.00	0.00	0.00		
Hieracium caesium	2	---	---	---	0	1	1	0.00	0.00	0.00		
Hieracium lachenalii	18	2	---	---	1	1	1	0.01	0.00	0.01		
Hieracium laevigatum	14	---	---	2	1	1	1	0.00	0.00	0.01		
Hieracium hypochoeroides subsp. wiesba	1	---	---	---	0	0	1	0.00	0.00	0.00		
Hieracium glaucinum	2	---	---	---	0	1	1	0.00	0.00	0.00		
Gladiolus palustris	1	---	---	---	1	0	0	0.00	0.00	0.00		
Glaucium corniculatum	1	---	---	---	0	1	0	0.00	0.00	0.00		
Hieracium saxatile	2	---	---	---	0	1	1	0.00	0.00	0.00		
Hieracium racemosum	14	---	---	---	1	1	1	0.00	0.00	0.00		
Hieracium porrifolium	1	---	---	---	1	0	0	0.00	0.00	0.00		
Carex acutiformis	1	---	---	---	1	0	0	0.00	0.00	0.00		
Hierochloe repens	5	---	3	---	0	1	0	0.00	0.00	0.00		
Himantoglossum adriaticum	8	2	---	---	1	1	1	0.00	0.00	0.00		
Himantoglossum caprinum	1	---	---	---	1	0	0	0.00	0.00	0.00		
Carduus uncinatus	6	---	3	---	0	1	0	0.00	0.00	0.00		
Glycyrrhiza echinata	3	---	---	---	1	1	0	0.00	0.00	0.00		
Hieracium pseudobifidum subsp. trebevic	1	---	---	---	0	0	1	0.00	0.00	0.00		
Geranium divaricatum	2	---	---	---	0	1	0	0.00	0.00	0.00		
Carex depressa subsp. transsilvanica	4	---	---	---	1	1	0	0.00	0.00	0.00		
Geranium dissectum	9	---	3	---	1	1	0	0.00	0.00	0.00		
Genista scythica	19	---	5	---	0	1	0	0.00	0.04	0.00		
Carex echinata	3	---	---	---	1	1	0	0.00	0.00	0.00		
Gentiana asclepiadea	8	---	---	---	1	0	1	0.00	0.00	0.00		
Centaurea salonitana	7	---	3	---	0	1	0	0.00	0.00	0.00		
Hieracium prenanthoides	5	---	---	3	1	0	1	0.00	0.00	0.01		
Carex flava agg.	4	---	---	---	1	0	1	0.00	0.00	0.00		
Gentiana utriculosa	16	6	---	---	1	0	0	0.01	0.00	0.00		
Carex ericetorum	21	---	---	3	1	1	1	0.00	0.00	0.16		
Hieracium schmidtii	1	---	---	---	0	0	1	0.00	0.00	0.00		
Haplophyllum suaveolens	12	---	4	---	0	1	0	0.00	0.00	0.00		
Carex brevicollis	2	---	---	---	0	1	1	0.00	0.00	0.00		
Hacquetia epipactis	2	---	---	---	1	0	1	0.00	0.00	0.00		
Helictochloa hookeri subsp. schelliana	2	---	---	---	1	1	0	0.00	0.00	0.00		
Helictochloa planiculmis	1	---	---	---	1	0	0	0.00	0.00	0.00		
Gnaphalium uliginosum	1	---	---	---	0	1	0	0.00	0.00	0.00		
Grindelia squarrosa	11	---	4	---	0	1	0	0.00	0.00	0.00		
Carex colchica	20	---	5	---	0	1	0	0.00	0.04	0.00		
Gratiola officinalis	2	---	---	---	0	1	0	0.00	0.00	0.00		
Goniolimon besseranum	12	---	4	---	0	1	0	0.00	0.00	0.00		
Goniolimon tataricum	24	---	6	---	0	1	0	0.00	0.02	0.00		

	total no. of occurrences	Phi value x 100 (orange: phi >= 0.2)			Constancy in % (green: const. ratio >= 2)			Average cover in % (blue: cover ratio >= 2)			Diagnostic value	Comment
Phytosociological order		B	Fv	S-Fp	B	Fv	S-Fp	B	Fv	S-Fp		
Number of relevés		3470	5016	1472	3470	5016	1472	3470	5016	1472		
Geranium sylvaticum	10	3	---	---	1	0	1	0.01	0.00	0.00		
Hieracium pilosum	2	---	---	---	0	1	0	0.00	0.00	0.00		
Hierochloe australis	5	---	---	---	1	1	1	0.00	0.00	0.01		
Hieracium maculatum	1	---	---	---	0	0	1	0.00	0.00	0.00		
Hieracium sparsum	1	---	---	---	0	0	1	0.00	0.00	0.00		
Hieracium onosmoides	1	---	---	---	1	0	0	0.00	0.00	0.00		
Hieracium piliferum	1	---	---	---	0	0	1	0.00	0.00	0.00		
Carex cespitosa	1	---	---	---	1	0	0	0.00	0.00	0.00		
Gladiolus imbricatus	6	3	---	---	1	0	0	0.00	0.00	0.00		
Hedysarum grandiflorum	6	---	3	---	0	1	0	0.00	0.01	0.00		
Gnaphalium sylvaticum	3	2	---	---	1	0	0	0.00	0.00	0.00		
Geranium sibiricum	4	---	---	---	1	1	0	0.00	0.00	0.00		
Number of relevés with bryophytes and lichens recorded		1113	1407	297	1113	1407	297					
**Bryophytes**												
Plagiomnium affine agg.	131	24	---	---	11	1	1					
Fissidens taxifolius	118	23	---	---	9	1	1					
Ceratodon purpureus	319	---	21	---	5	18	5					
Tortella tortuosa	250	---	---	54	4	4	49					
Ditrichum flexicaule	110	---	---	39	1	2	26					
Homalothecium philippeanum	41	---	---	27	1	1	11					
Encalypta streptocarpa	58	---	---	21	1	1	10					
Ctenidium molluscum	31	---	---	20	1	0	7					
Oxyrhynchium hians	137	14	---	---	8	4	0					
Thuidium assimile	67	17	---	---	6	1	1					
Weissia spec. div.	250	---	15	---	4	13	5					
Polytrichum piliferum	135	---	17	---	1	9	3					
Rhytidium rugosum	279	---	---	15	10	8	20					
Tortella inclinata	97	---	---	18	1	4	11					
Schistidium apocarpum agg.	59	---	---	17	1	2	8					
Homalothecium sericeum	55	---	---	14	1	2	6					
Encalypta vulgaris	41	---	---	14	1	1	6					
Orthotrichum anomalum	38	---	---	15	0	1	6					
Abietinella abietina	813	15	---	---	35	27	16					
Hypnum cupressiforme	394	---	9	---	8	18	15					
Homalothecium lutescens	382	16	---	---	19	11	5					
Syntrichia ruralis agg.	259	---	12	---	2	14	12					
Bryum caespiticium	155	---	8	---	5	7	2					
Fissidens dubius	121	11	---	4	9	1	6					
Bryum argenteum	120	---	1	7	2	5	7					
Grimmia pulvinata	117	---	10	---	1	7	5					
Brachythecium albicans	93	---	7	---	3	4	0					
Rhytidiadelphus triquetrus	73	7	---	6	5	0	5					
Racomitrium canescens agg.	72	---	9	---	1	4	2					
Bryum capillare	70	---	9	---	1	4	1					
Brachythecium rutabulum	59	14	---	---	4	1	0					
Pleurochaete squarrosa	57	---	7	---	1	3	2					
Campyliadelphus chrysophyllus	48	10	---	---	3	1	1					
Pleurozium schreberi	46	---	---	6	1	1	3					
Hylocomium splendens	46	---	---	12	2	1	5					
Plagiomnium cuspidatum	42	12	---	---	3	1	1					
Pseudoscleropodium purum	42	12	---	---	3	1	1					
Plagiomnium undulatum	39	8	---	---	3	1	1					
Thuidium delicatulum	38	8	---	---	3	0	2					
Barbula unguiculata	37	---	---	---	2	1	1					

	total no. of occurrences	Phi value x 100 (orange: phi >= 0.2)			Constancy in % (green: const. ratio >= 2)			Average cover in % (blue: cover ratio >= 2)			Diagnostic value	Comment
Phytosociological order		B	Fv	S-Fp	B	Fv	S-Fp	B	Fv	S-Fp		
Number of relevés		3470	5016	1472	3470	5016	1472	3470	5016	1472		
Rhytidiadelphus squarrosus	33	11	---	---	3	1	1					
Barbula convoluta	32	---	---	---	1	1	1					
Polytrichum juniperinum	30	---	6	---	1	2	1					
Mannia fragrans	28	---	2	---	0	2	2					
Brachythecium glareosum	28	---	---	---	1	1	0					
Campylophyllum calcareum	24	5	---	---	1	1	0					
Brachythecium salebrosum	24	8	---	---	2	1	0					
Amblystegium serpens	22	8	---	---	2	1	0					
Riccia ciliata	21	---	10	---	0	1	0					
Calliergonella cuspidata	21	10	---	---	2	1	0					
Cirriphyllum piliferum	21	11	---	---	2	0	0					
Syntrichia montana	19	---	3	---	1	1	1					
Entodon concinnus	18	4	---	---	1	0	1					
Tortula muralis	17	---	4	---	1	1	1					
Didymodon acutus	15	---	4	---	1	1	1					
Brachythecium campestre	15	---	---	---	1	1	0					
Didymodon fallax	14	---	---	---	1	1	0					
Phascum cuspidatum	13	---	6	---	1	1	0					
Riccia ciliifera	13	---	5	---	0	1	1					
Dicranum scoparium	13	---	---	12	1	1	3					
Brachytheciastrum velutinum	13	6	---	---	1	1	0					
Rhynchostegium megapolitanum	13	---	5	---	1	1	0					
Orthotrichum cupulatum	13	---	3	---	0	1	1					
Hypnum vaucheri	10	---	---	14	0	1	3					
Climacium dendroides	10	7	---	---	1	1	0					
Campylophyllum sommerfeltii	10	---	---	---	1	1	1					
Leskea polycarpa	10	---	---	14	1	0	3					
Distichium capillaceum	10	---	---	11	1	1	2					
Oxyrrhynchium schleicheri	9	---	---	---	1	1	0					
Leucodon sciuroides	9	---	---	---	0	1	1					
Rhodobryum roseum	8	6	---	---	1	1	0					
Atrichum undulatum	8	7	---	---	1	0	0					
Neckera crispa	8	---	---	13	0	0	3					
Anomodon viticulosus	8	---	---	11	0	1	2					
Oxymitra incrassata	7	---	---	---	0	1	1					
Rhizomnium punctatum	7	6	---	---	1	0	0					
Bryum rubens	7	---	---	---	1	1	0					
Pseudoleskea catenulata	7	---	---	---	0	1	1					
Dicranum polysetum	6	---	---	---	1	1	1					
Grimmia anodon	6	---	5	---	0	1	0					
Hedwigia ciliata agg.	6	---	---	---	0	1	1					
Schistidium brunnescens	6	---	---	---	0	1	1					
Eurhynchiastrum pulchellum	6	---	---	---	1	1	0					
Tortula lanceolata	6	---	---	---	1	1	0					
Thuidium tamariscinum	6	5	---	---	1	1	0					
Plagiochila asplenioides	6	---	---	10	1	0	2					
Entosthodon fascicularis	6	---	---	---	1	1	0					
Pleuridium subulatum	6	---	---	---	1	1	0					
Campylium stellatum	6	6	---	---	1	0	0					
Lecanora campestris	6	---	5	---	0	1	0					
Physcomitrium pyriforme	5	---	---	---	1	1	0					
Rhodobryum ontariense	5	5	---	---	1	0	0					
Lophocolea bidentata	5	---	---	---	1	1	0					
Protobryum bryoides	5	---	5	---	0	1	0					
Grimmia orbicularis	5	---	---	9	0	1	1					
Didymodon cordatus	5	---	---	---	1	1	1					
Dicranella heteromalla	5	---	---	---	1	1	1					
Trichostomum crispulum	5	---	---	7	1	1	1					

	total no. of occurrences	Phi value x 100 (orange: phi >= 0.2)			Constancy in % (green: const. ratio >= 2)			Average cover in % (blue: cover ratio >= 2)			Diagnostic value	Comment
Phytosociological order		B	Fv	S-Fp	B	Fv	S-Fp	B	Fv	S-Fp		
Number of relevés		3470	5016	1472	3470	5016	1472	3470	5016	1472		
Pterygoneurum ovatum	5	---	---	---	1	1	0					
Fissidens viridulus	5	---	---	---	1	1	0					
Tortella fragilis	5	---	5	---	0	1	0					
Fissidens bryoides	4	---	---	---	1	1	0					
Eurhynchium striatum	4	5	---	---	1	0	0					
Bryoerythrophyllum recurvirostrum	4	---	---	---	1	0	1					
Microbryum davallianum	4	---	---	---	0	1	0					
Pterygoneurum subsessile	4	---	---	---	1	1	0					
Bryum archangelicum	4	---	---	---	1	1	0					
Bryum atrovirens agg.	4	---	---	---	1	1	0					
Pleuridium acuminatum	4	---	---	---	1	1	0					
Bryum kunzei	4	---	---	---	0	1	0					
Fissidens adianthoides	3	---	---	---	1	0	0					
Tortula modica	3	---	---	---	1	1	0					
Cephaloziella divaricata	2	---	---	---	0	1	0					
Tortula truncata	2	---	---	---	1	1	0					
Thuidium recognitum	1	---	---	---	0	1	0					
Polytrichastrum formosum	1	---	---	---	1	0	0					
Pohlia nutans	1	---	---	---	0	1	0					
Leptodictyum riparium	1	---	---	---	1	0	0					
Calliergonella lindbergii	1	---	---	---	1	0	0					
Drepanocladus longifolius	1	---	---	---	1	0	0					
Entodon schleicheri	1	---	---	---	1	0	0					
Pogonatum urnigerum	1	---	---	---	0	1	0					
**Lichens**												
Cladonia rangiformis	187	---	19	---	1	12	4					
Cladonia foliacea	127	---	13	---	1	8	4					
Cladonia pyxidata	132	---	---	15	1	6	11					
Cladonia symphycarpia	104	---	3	9	1	5	7					
Cladonia furcata agg.	94	---	10	---	1	5	2					
Cladonia pocillum	74	---	2	6	1	3	4					
Xanthoparmelia stenophylla	64	---	6	---	0	4	4					
Cladonia fimbriata	60	---	4	7	1	3	4					
Collema spec. div.	52	---	8	---	1	3	2					
Peltigera rufescens	51	---	1	9	1	2	4					
Xanthoparmelia pulla	48	---	4	---	0	3	3					
Cladonia convoluta	42	---	3	---	1	2	3					
Cladonia rangiferina	42	---	1	6	1	2	3					
Cladonia arbuscula agg.	33	---	6	---	1	2	1					
Cetraria aculeata	31	---	5	---	1	2	1					
Protoparmeliopsis muralis	29	---	9	---	1	2	1					
Xanthoparmelia conspersa	28	---	8	---	0	2	1					
Cladonia coniocraea	26	---	2	---	1	1	2					
Cladonia glauca	24	---	---	---	1	1	0					
Toninia sedifolia	23	---	0	6	1	1	2					
Cladonia chlorophaea	22	---	---	8	1	1	2					
Squamarina cartilaginea	19	---	---	16	0	1	4					
Psora decipiens	17	---	---	16	0	1	4					
Cladonia subulata	16	---	---	---	1	1	1					
Diploschistes muscorum	14	---	---	---	1	1	0					
Solorina saccata	12	---	---	17	0	0	4					
Cetraria islandica	12	---	8	---	0	1	0					
Verrucaria muralis	11	4	---	---	1	1	1					
Stereocaulon incrustatum	9	---	7	---	0	1	0					
Bilimbia sabuletorum	9	---	---	---	1	1	1					
Candelariella vitellina	9	---	3	---	0	1	1					
Verrucaria nigrescens	8	---	---	---	1	1	1					

	total no. of occurrences	Phi value x 100 (orange: phi >= 0.2)			Constancy in % (green: const. ratio >= 2)			Average cover in % (blue: cover ratio >= 2)			Diagnostic value	Comment
Phytosociological order		B	Fv	S-Fp	B	Fv	S-Fp	B	Fv	S-Fp		
Number of relevés		3470	5016	1472	3470	5016	1472	3470	5016	1472		
Physcia wainioi	6	---	5	---	0	1	0					
Lecanora dispersa	6	6	---	---	1	0	0					
Cladonia magyarica	6	---	---	---	0	1	1					
Fulgensia fulgens	6	---	---	10	0	1	2					
Leptogium lichenoides	6	---	---	---	1	1	1					
Cladonia uncialis	5	---	5	---	0	1	0					
Cladonia polycarpoides	5	---	---	---	0	1	1					
Rhizocarpon geographicum	5	---	---	---	0	1	1					
Cladonia squamosa	5	---	---	---	0	1	1					
Verrucaria aethiobola	5	---	---	---	1	1	0					
Physcia dubia	5	---	---	---	0	1	1					
Lobothallia radiosa	5	---	5	---	0	1	0					
Parmelia saxatilis	5	---	---	---	0	1	1					
Cladonia verticillata	4	---	---	---	0	1	1					
Lecidea fuscoatra	4	---	---	---	0	1	1					
Mycobilimbia lurida	4	---	---	---	0	1	1					
Candelariella aurella	4	---	---	---	1	0	1					
Cladonia phyllophora	4	---	---	---	0	1	0					
Ramalina capitata	4	---	---	---	0	1	1					
Xanthoparmelia protomatrae	4	---	---	---	0	1	1					
Cladonia rei	3	---	---	---	0	1	0					
Peltigera canina	3	---	---	---	1	1	0					
Cladonia gracilis	1	---	---	---	0	1	0					
