# Supplementary material for: A higher‐level classification of the Pannonian and western Pontic steppe grasslands (Central and Eastern Europe)
Source: Appl Veg Sci. 2016 Sep 16;20(1):143–58. doi: 10.1111/avsc.12265 (PMC5348766; doi:10.1111/avsc.12265)
Supplement: Supplementary file 7 — Appendix S7. Full synoptic table of the Festuco‐Brometea. [file AVSC-20-143-s007.pdf]

Supporting information to the paper

Willner, W. et al. A higher-level classification of the Pannonian and western Pontic steppe grasslands (Central and Eastern Europe). Applied Vegetation Science.

Appendix S7. Synoptic table of the Festuco-Brometea. Values are percentage constancy. Assignment of relevés to orders according to the total cover of diagnostic species. Internal classification of the orders follows the original Twinspan clusters. A few small clusters have been omitted. Diagnostic species of orders are sorted by descending fidelity for the order (calculated as phi coefficient). Non-vascular plants are given at the end of the table. Constancy of the latter was calculated only within the subset of relevés where these species were recorded.

Group number		B1	B2	B3	B4	B5	B6	B7	F1	F2	F3	F4	S1	S2	S3	S4	S5	S6	S7
Twinspan cluster level 4		7	7	8	8	9	9	10	10	10	10	10	10	13	13	14	14	15	16
Twinspan cluster level 5-6		0	1	0	1	0	1		0-0	0-1	1-0	1-1		0	1	0	1	0	1
No. of relevés		301	135	994	235	846	765	760	1324	730	1316	177	709	65	690	174	250	192	66
**Brometalia erecti**																			
Brachypodium pinnatum agg.	2544	15	8	62	86	68	82	15	4	10	1	1	6	.	6	3	.	5	.
Briza media	1946	20	21	78	94	43	38	6	1	1	1	.	2	.	4	1	.	4	.
Dactylis glomerata	2188	52	24	65	68	34	45	24	6	6	16	.	1	.	1	.	.	1	.
Bromus erectus	1533	15	.	42	94	38	17	23	6	3	1	.	5	.	3	3	5	.	.
Centaurea jacea	1388	30	58	44	74	21	19	13	2	2	8	.	1	.	1	.	.	.	.
Leontodon hispidus	1805	38	25	67	47	32	19	15	5	5	10	.	7	.	3	1	.	5	.
Knautia arvensis	1837	35	53	48	35	35	38	16	4	6	15	2	2	.	2	.	.	11	.
Lotus corniculatus	2930	60	39	73	91	48	27	33	15	15	23	3	13	.	6	4	5	23	.
Arrhenatherum elatius	1707	47	12	51	42	31	23	23	14	1	4	.	8	2	1	.	.	1	.
Leucanthemum vulgare agg.	1631	35	52	62	76	21	21	7	1	1	3	.	2	.	10	3	.	39	6
Plantago media	3439	57	38	73	74	53	56	32	10	34	38	15	16	.	8	2	.	7	2
Trifolium montanum	1718	10	27	54	72	28	36	11	4	7	13	.	4	.	1	.	.	.	.
Salvia pratensis	3069	32	46	56	85	53	64	28	18	20	22	.	15	.	14	5	4	3	.
Pimpinella saxifraga agg.	3175	44	59	63	63	57	40	40	24	18	17	2	25	.	16	7	1	19	6
Trifolium pratense	1241	54	55	50	53	6	4	10	2	1	12	.	1	.	1	.	.	2	.
Linum catharticum	1468	6	6	51	81	35	14	7	2	2	3	2	5	.	11	2	.	23	6
Anthoxanthum odoratum agg.	933	33	4	46	41	4	6	11	4	1	1	.	3	.	1	.	.	.	.
Thymus pulegioides	1158	24	58	45	14	26	1	15	5	1	2	.	4	3	4	.	.	5	2
Centaurea scabiosa	2595	15	24	38	49	59	66	20	11	22	18	6	9	.	15	13	2	7	.
Festuca rubra agg.	740	16	24	39	12	10	4	9	2	.	1	.	1	.	1	.	.	1	.
Betonica officinalis	756	9	4	29	34	8	24	5	1	1	3	1	1	.	1	.	.	1	.
Plantago lanceolata	2945	68	44	70	86	29	18	37	23	18	37	23	13	.	2	.	.	2	.
Vicia cracca	744	13	37	33	37	9	7	5	1	1	3	.	1	.	1	.	.	9	.
Carex caryophyllea	1289	12	2	35	68	28	13	14	9	5	3	.	6	.	1	2	1	.	.
Ranunculus polyanthemos agg.	1426	34	22	38	23	18	33	14	2	4	19	.	3	.	1	1	1	3	.
Carlina acaulis	1124	4	4	44	46	28	10	6	2	1	1	.	4	.	6	5	.	24	2
Trisetum flavescens	531	15	.	37	25	3	1	2	1	1	.	.	1	.	.	.	.	.	.
Carex flacca	610	4	2	21	76	18	4	1	1	.	.	.	1	.	1	.	.	2	.
Agrostis capillaris	795	30	19	35	13	8	5	12	4	1	2	.	1	.	1	.	.	.	.
Festuca pratensis	804	32	37	36	10	5	10	6	1	1	5	.	1	.	.	.	.	1	.
Daucus carota	1359	43	59	31	41	19	7	21	4	3	18	3	4	.	1	.	.	.	.

Group number		B1	B2	B3	B4	B5	B6	B7	F1	F2	F3	F4	S1	S2	S3	S4	S5	S6	S7
Twinspan cluster level 4		7	7	8	8	9	9	10	10	10	10	10	10	13	13	14	14	15	16
Twinspan cluster level 5-6		0	1	0	1	0	1		0-0	0-1	1-0	1-1		0	1	0	1	0	1
No. of relevés		301	135	994	235	846	765	760	1324	730	1316	177	709	65	690	174	250	192	66
Veronica chamaedrys	844	34	21	34	13	10	6	9	2	1	5	.	2	.	2	.	.	5	6
Cirsium pannonicum	529	1	.	20	47	8	16	1	.	1	1	.	.	.	2	.	.	1	.
Cruciata glabra	607	2	2	34	34	7	8	1	1	1	1	.	1	.	1	.	.	5	2
Prunella vulgaris	596	20	24	27	17	6	7	4	1	1	3	.	1	.	.	.	.	1	.
Ononis spinosa	799	18	2	28	9	16	12	9	3	2	6	1	1	.	.	.	.	.	.
Tragopogon pratensis	790	13	5	37	28	12	7	3	2	6	1	.	2	.	1	1	.	2	.
Campanula glomerata	727	2	1	26	11	14	24	3	1	2	1	.	2	.	2	1	.	3	.
Luzula campestris agg.	700	21	.	34	25	4	4	9	3	.	1	1	3	.	.	.	.	.	3
Polygala comosa	802	10	31	23	60	16	5	6	1	3	4	2	2	.	1	.	.	.	2
Ranunculus bulbosus	556	13	1	23	50	9	2	3	2	1	1	.	1	.	1	1	.	.	.
Filipendula vulgaris	1812	22	18	39	36	12	55	14	6	21	18	2	12	.	1	.	3	.	.
Carex montana	481	.	.	22	34	7	9	2	1	1	1	.	1	.	1	.	.	2	.
Avenula pubescens	618	8	3	26	20	7	14	5	2	1	1	.	2	.	1	1	.	.	.
Viola hirta	1489	16	1	42	27	27	28	8	3	14	4	1	10	.	10	2	.	34	.
Koeleria pyramidata	432	2	.	9	79	11	2	2	1	1	.	.	1	.	1	.	.	3	.
Rhinanthus minor	458	10	3	25	10	5	3	4	2	1	1	.	1	.	1	.	.	1	.
Primula veris	699	3	1	34	.	8	16	4	1	1	1	.	2	.	7	.	.	1	17
Campanula patula	327	9	2	24	6	1	1	2	1	.	1	.	1	.	1	.	.	.	.
Prunella grandiflora	574	3	1	12	32	15	18	2	1	1	1	.	1	.	3	1	.	4	.
Rumex acetosa	460	13	13	26	11	1	4	3	2	1	2	.	1	.	1	1	.	.	.
Carex tomentosa	358	4	.	17	.	6	12	1	1	1	1	.	1	.	.	1	.	.	.
Lathyrus pratensis	304	12	6	18	19	2	1	1	1	.	1	.	.	.	.	.	.	2	.
Colchicum autumnale	294	6	.	22	3	2	1	1	1	1	.	.	1	.	.	.	.	.	.
Potentilla erecta	283	1	1	18	33	1	1	.	1	1	1	.	1	.	.	.	.	.	.
Seseli annuum	782	6	19	11	3	23	20	9	5	3	3	1	4	.	1	1	.	.	.
Peucedanum cervaria	747	2	.	9	10	11	41	5	2	5	1	.	3	.	6	.	1	3	.
Taraxacum sect. Ruderalia	714	28	24	27	1	5	6	7	2	1	8	1	2	.	1	1	.	5	.
Danthonia alpina	244	1	.	4	41	3	8	1	1	1	.	.	.	.	.	.	.	.	.
Buphthalmum salicifolium	410	1	1	5	79	13	1	1	.	.	.	.	1	.	3	.	.	13	.
Potentilla heptaphylla	951	15	1	35	1	22	5	9	3	1	2	.	1	2	17	11	.	7	2
Carlina vulgaris agg.	1092	10	22	16	16	28	17	14	4	6	8	.	7	.	6	5	1	9	3
Ranunculus acris	281	5	19	17	8	1	2	1	1	.	1	.	1	.	.	.	.	1	.
Thymus longicaulis	198	1	.	1	69	3	1	.	.	.	.	.	.	.	.	.	.	.	.
Clinopodium vulgare	631	17	4	15	9	12	10	7	3	5	3	1	3	.	1	.	.	8	.
Euphorbia verrucosa	196	.	.	2	55	6	1	1	.	.	.	.	.	.	.	.	.	.	.
Cerastium fontanum subsp. vulgare	564	22	13	24	3	3	2	7	2	.	7	1	2	.	1	.	.	2	.
Prunella laciniata	414	13	.	10	26	7	5	5	2	1	2	.	1	.	1	.	.	.	.
Carex michelii	407	1	.	7	.	9	21	3	2	2	2	1	1	.	1	.	.	.	.
Onobrychis viciifolia agg.	1037	3	3	17	20	15	33	7	2	16	9	8	7	.	1	1	.	.	.
Polygala vulgaris	392	3	1	17	25	5	2	3	1	1	3	.	1	.	1	.	.	2	2
Alchemilla spec.div.	213	2	1	18	4	1	1	1	.	1	.	.	1	.	.	.	.	2	.

Group number		B1	B2	B3	B4	B5	B6	B7	F1	F2	F3	F4	S1	S2	S3	S4	S5	S6	S7
Twinspan cluster level 4		7	7	8	8	9	9	10	10	10	10	10	10	13	13	14	14	15	16
Twinspan cluster level 5-6		0	1	0	1	0	1		0-0	0-1	1-0	1-1		0	1	0	1	0	1
No. of relevés		301	135	994	235	846	765	760	1324	730	1316	177	709	65	690	174	250	192	66
Polygala major	449	1	.	10	.	9	21	1	1	3	1	.	3	.	3	1	.	.	.
Tanacetum corymbosum	546	1	1	12	5	8	24	2	2	2	1	.	2	.	5	1	.	6	2
Trifolium medium	499	10	5	20	9	5	5	3	1	1	10	.	1	.	.	.	.	1	.
Inula salicina	264	4	1	10	1	4	8	1	1	1	1	.	1	.	1	.	.	.	.
Gymnadenia conopsea	223	1	.	7	31	4	2	.	1	1	.	.	.	.	2	.	.	5	.
Hypochaeris maculata	467	1	.	9	23	6	13	2	1	2	7	4	1	.	1	.	.	.	.
Linum flavum	396	1	4	1	1	10	19	4	1	5	1	1	3	.	5	.	.	2	2
Aster amellus	645	1	2	1	2	15	26	3	2	11	4	1	4	.	6	1	.	1	9
**Festucetalia valesiacae**																			
Festuca valesiaca	3011	25	1	6	1	9	19	30	54	49	68	76	27	2	7	7	10	.	.
Eryngium campestre	3044	27	.	6	.	20	27	44	50	60	54	55	32	.	1	3	4	.	.
Thymus pannonicus agg.	3079	20	1	10	1	23	36	34	50	65	49	15	32	3	7	6	6	1	3
Bothriochloa ischaemum	1997	9	.	1	2	9	13	16	34	71	24	31	26	.	7	21	4	.	.
Salvia nemorosa	1055	4	.	1	.	2	10	10	5	27	35	48	5	.	1	.	.	1	.
Stipa capillata	1310	.	.	.	.	1	5	8	19	50	17	55	23	.	5	18	8	.	.
Koeleria macrantha	2628	15	3	11	.	19	22	29	52	42	31	40	39	.	11	19	2	.	5
Artemisia austriaca	357	1	.	.	.	.	.	1	3	2	18	29	1	.	.	.	.	.	.
Euphorbia nicaeensis	767	.	.	1	.	1	16	5	2	19	22	58	3	.	1	1	3	.	.
Potentilla argentea	1081	31	36	3	.	1	1	16	23	4	27	5	5	6	1	.	.	.	.
Falcaria vulgaris	996	5	2	2	.	6	13	12	9	27	24	12	7	.	.	1	.	.	.
Astragalus onobrychis	947	1	.	1	.	5	11	10	10	30	18	27	9	.	1	3	.	.	.
Salvia nutans	503	1	.	1	.	1	4	2	1	23	9	62	6	.	.	.	.	.	.
Carex praecox	427	6	1	1	.	1	2	5	6	5	17	2	1	.	1	1	.	.	.
Centaurea stoebe	2051	10	.	3	1	16	14	27	49	42	13	1	32	20	11	21	7	1	2
Verbascum phoeniceum	574	3	.	1	.	1	6	5	10	18	10	8	6	.	1	.	1	.	.
Trifolium arvense	665	11	7	1	.	1	1	9	23	1	11	2	9	.	1	1	1	.	.
Nonea pulla	582	2	12	1	.	2	7	7	3	18	17	9	4	.	.	.	.	.	.
Chondrilla juncea	324	2	.	.	.	1	1	2	10	3	8	2	3	.	1	2	.	.	.
Cleistogenes serotina	365	.	.	.	.	.	.	1	6	19	5	7	7	.	1	2	1	.	.
Taraxacum serotinum	306	.	.	.	.	1	2	4	2	10	9	21	1	.	1	.	.	.	.
Astragalus austriacus	515	1	.	.	.	1	5	4	2	25	9	27	6	.	1	1	.	.	.
Achillea nobilis	382	3	.	1	.	1	1	5	11	1	10	13	3	.	1	.	.	.	.
Berteroa incana	356	2	7	.	.	1	1	5	8	2	11	2	2	2	1	.	.	.	.
Viola ambigua	485	1	.	1	.	1	10	3	1	13	13	40	3	.	1	1	1	.	.
Artemisia campestris	1149	1	40	1	.	4	5	10	25	25	14	6	21	18	8	9	1	1	.
Cytisus austriacus	677	2	.	1	.	3	19	6	4	18	16	12	5	.	1	.	.	.	.
Linum austriacum	316	1	.	.	.	1	2	4	3	10	5	25	3	.	1	.	1	.	.
Veronica prostrata	547	4	.	2	.	2	3	7	15	10	5	1	8	.	2	2	.	.	.
Iris pumila	369	.	.	.	.	1	1	1	6	13	3	26	8	.	1	4	1	.	3
Linaria genistifolia	592	.	.	.	.	1	2	4	16	14	4	5	13	25	4	8	3	.	9

Group number		B1	B2	B3	B4	B5	B6	B7	F1	F2	F3	F4	S1	S2	S3	S4	S5	S6	S7
Twinspan cluster level 4		7	7	8	8	9	9	10	10	10	10	10	10	13	13	14	14	15	16
Twinspan cluster level 5-6		0	1	0	1	0	1		0-0	0-1	1-0	1-1		0	1	0	1	0	1
No. of relevés		301	135	994	235	846	765	760	1324	730	1316	177	709	65	690	174	250	192	66
Melica transsilvanica	342	.	1	.	.	2	2	4	9	3	5	8	6	3	2	.	.	5	.
**Stipo-Festucetalia pallentis**																			
Festuca pallens agg.	1124	.	1	.	.	2	1	2	6	1	.	.	27	34	58	84	70	28	2
Carex humilis	2637	1	3	3	10	17	36	13	15	53	2	1	65	.	66	76	87	29	5
Teucrium montanum	1583	.	.	1	12	7	5	8	6	32	5	5	33	2	54	79	69	22	8
Jovibarba globifera	709	.	.	.	.	2	1	1	5	.	1	.	14	42	34	47	18	49	3
Thymus praecox	1147	1	.	2	.	7	1	6	15	2	1	.	25	5	31	90	81	4	.
Anthericum ramosum	1798	1	.	9	18	23	35	5	6	10	2	2	29	2	55	56	40	40	17
Sesleria caerulea	473	.	.	1	1	2	1	1	1	1	.	.	2	2	32	13	.	86	.
Asplenium ruta-muraria	446	.	.	.	.	1	.	1	2	1	.	.	6	18	27	10	4	42	73
Helianthemum canum	453	.	.	1	.	1	1	1	1	2	.	.	9	.	19	48	47	1	8
Leontodon incanus	456	1	.	.	8	4	.	.	1	1	.	.	2	.	26	54	8	41	.
Fumana procumbens	388	.	.	.	.	.	.	1	1	1	.	.	6	.	4	58	72	.	.
Allium lusitanicum	621	.	.	1	.	4	2	2	5	1	1	.	15	34	28	25	12	18	.
Seseli osseum	1098	1	.	.	.	1	3	7	23	6	1	.	28	28	38	47	6	31	11
Sedum album	495	.	.	1	.	1	1	1	6	.	.	.	11	18	24	32	14	23	.
Genista pilosa	554	1	.	1	.	6	1	3	3	1	.	.	14	5	29	32	5	13	.
Vincetoxicum hirundinaria	1265	2	4	3	.	10	18	9	5	9	8	1	17	5	46	16	20	48	36
Scorzonera austriaca	385	.	.	1	.	1	2	1	1	2	.	.	5	.	9	40	62	.	.
Globularia bisnagarica	771	.	.	1	39	13	3	2	3	2	.	.	11	.	17	51	61	2	.
Stipa eriocaulis	294	.	.	.	.	1	1	1	1	2	1	.	5	2	1	12	75	.	.
Asplenium trichomanes	257	.	.	.	.	1	.	1	2	.	.	.	4	35	11	.	1	21	62
Pulsatilla halleri subsp. slavica	211	.	.	.	.	1	.	.	.	.	.	.	1	.	13	1	.	53	.
Phyteuma orbiculare	302	.	.	3	2	1	1	.	.	.	.	.	.	.	14	7	2	56	21
Melica ciliata	678	.	.	.	.	1	1	3	13	9	1	.	19	25	20	30	8	4	33
Hornungia petraea	189	1	.	.	.	.	.	.	1	1	.	.	2	2	2	10	53	.	.
Polygonatum odoratum	473	.	.	2	3	4	8	2	1	1	.	.	6	.	23	6	9	25	.
Poa badensis	254	.	.	.	.	.	1	1	3	1	.	.	7	.	8	29	20	.	5
Biscutella laevigata	238	.	.	1	1	3	1	1	1	.	.	.	2	.	17	16	4	11	12
Saxifraga paniculata	165	.	.	.	.	.	.	.	1	.	.	.	1	5	9	.	.	30	50
Alyssum montanum	439	.	.	1	.	2	1	2	7	2	1	.	12	.	12	43	13	.	2
Arabidopsis arenosa	337	1	1	1	.	2	1	2	3	1	1	.	4	22	17	2	2	19	26
Clinopodium alpinum	276	.	.	1	6	3	.	.	1	1	.	.	2	.	17	3	.	28	8
Campanula rotundifolia	604	1	13	3	.	10	8	5	2	1	1	.	8	32	21	22	6	15	14
Thymus comosus	214	.	.	.	.	1	1	1	2	1	.	.	5	2	14	1	.	.	56
Bromus pannonicus	208	.	.	2	.	1	1	.	1	.	.	.	4	.	15	6	2	2	.
Linum tenuifolium	715	.	.	1	2	8	7	4	3	13	1	21	11	.	14	52	31	.	.
Minuartia setacea	237	.	.	.	.	.	.	.	3	2	1	2	6	8	5	19	25	.	3
Polygala amara agg.	204	1	.	3	1	1	.	1	.	1	.	.	1	.	12	3	2	23	11
Erysimum odoratum	454	.	1	1	.	3	5	2	5	3	1	.	8	.	23	4	1	4	27

Group number		B1	B2	B3	B4	B5	B6	B7	F1	F2	F3	F4	S1	S2	S3	S4	S5	S6	S7
Twinspan cluster level 4		7	7	8	8	9	9	10	10	10	10	10	10	13	13	14	14	15	16
Twinspan cluster level 5-6		0	1	0	1	0	1		0-0	0-1	1-0	1-1		0	1	0	1	0	1
No. of relevés		301	135	994	235	846	765	760	1324	730	1316	177	709	65	690	174	250	192	66
Minuartia laricifolia	112	.	.	.	.	.	.	.	.	.	.	.	1	.	5	2	.	38	.
Galium pusillum agg.	305	2	1	7	1	2	.	1	1	.	.	.	3	.	10	5	.	50	.
Seseli leucospermum	110	.	.	.	.	.	.	.	.	.	.	.	1	2	2	13	28	.	.
Erysimum witmannii	113	.	.	1	.	1	.	.	.	.	.	.	1	.	6	.	.	31	.
Hieracium bupleuroides	104	.	.	.	.	.	.	.	.	.	.	.	.	.	5	.	.	35	.
Carduus defloratus	151	.	.	1	.	1	.	.	1	.	.	.	1	.	5	.	.	45	3
Cyanus triumfettii	405	1	.	2	.	4	7	2	3	2	.	.	4	.	20	3	2	13	15
Kernera saxatilis	99	.	.	.	.	.	.	.	.	.	.	.	.	.	5	1	.	30	9
Dianthus praecox	116	.	.	.	.	1	.	.	1	.	.	.	1	.	5	16	1	20	.
Draba lasiocarpa	98	.	.	.	.	.	.	.	.	.	.	.	1	2	4	23	9	.	6
Sesleria rigida	92	.	.	.	.	.	.	.	.	.	.	.	1	.	5	.	.	.	82
Scabiosa lucida	106	.	.	1	.	1	.	.	.	.	.	.	.	.	2	.	.	41	2
Dianthus plumarius	90	.	.	.	.	.	.	.	.	.	.	.	1	.	2	5	27	1	.
Thymus pulcherrimus	94	1	.	1	.	.	.	.	.	.	.	.	1	.	2	1	.	40	.
Thesium alpinum	114	.	.	1	1	1	.	.	.	.	.	.	.	.	3	2	.	38	.
Primula auricula	82	.	.	.	.	.	.	.	.	.	.	.	.	.	3	.	.	32	.
Helictotrichon decorum	93	1	.	1	.	1	.	.	1	1	.	.	1	.	6	.	.	.	59
Paronychia cephalotes	99	.	.	.	.	.	.	.	1	1	.	5	1	.	1	3	28	.	2
Pulsatilla vulgaris	495	1	.	1	1	7	11	3	4	4	1	.	8	2	16	13	4	1	5
Scabiosa canescens	275	1	.	1	.	3	4	2	3	3	1	.	5	.	4	17	10	.	.
**Other species**																			
Achillea millefolium agg.	5269	84	79	83	31	64	62	66	50	52	71	25	39	2	6	3	1	21	3
Euphorbia cyparissias	4798	42	6	50	39	62	44	49	67	59	22	.	60	8	60	53	27	45	27
Teucrium chamaedrys	4217	12	1	23	51	50	58	42	46	67	37	32	57	5	49	25	14	20	15
Asperula cynanchica	3716	18	1	24	30	48	46	36	42	58	25	12	53	.	45	61	15	20	.
Galium verum	3650	60	30	55	35	50	52	48	35	31	52	6	20	2	1	1	5	3	.
Festuca stricta subsp. sulcata	3367	53	1	51	65	61	61	42	34	43	8	.	26	.	9	6	1	.	11
Potentilla incana agg.	3363	2	1	3	3	18	20	29	60	66	22	49	68	11	37	71	70	2	17
Poa pratensis agg.	3295	69	58	50	24	37	39	47	28	19	60	27	11	.	1	.	.	1	.
Medicago falcata	3285	24	38	37	18	45	43	32	29	49	51	40	31	.	3	1	.	2	.
Sanguisorba minor	2943	18	7	43	78	58	17	24	29	18	5	19	30	2	35	63	77	9	.
Securigera varia	2664	36	29	37	6	46	38	26	20	30	30	36	18	.	12	1	1	18	14
Hypericum perforatum	2608	35	41	38	24	33	21	34	39	17	24	5	29	2	9	6	6	9	5
Scabiosa ochroleuca	2484	16	27	19	1	44	39	30	27	27	18	2	29	2	24	24	6	4	3
Stachys recta	2431	2	4	4	13	19	43	16	27	49	28	38	34	11	31	10	12	9	27
Fragaria viridis	2252	39	59	33	14	32	36	28	16	30	26	1	14	.	3	.	1	1	9
Dianthus carthusianorum agg.	2246	17	1	33	15	37	29	22	34	18	2	.	33	6	22	7	11	13	26
Agrimonia eupatoria	2182	47	80	37	13	29	27	29	6	13	45	7	7	.	1	.	.	2	.
Helianthemum nummularium	2006	3	.	28	53	33	20	10	13	12	1	.	27	.	45	43	36	26	17
Dorycnium pentaphyllum agg.	1845	15	1	11	33	29	45	16	11	42	2	.	22	.	10	32	33	1	.

Group number		B1	B2	B3	B4	B5	B6	B7	F1	F2	F3	F4	S1	S2	S3	S4	S5	S6	S7
Twinspan cluster level 4		7	7	8	8	9	9	10	10	10	10	10	10	13	13	14	14	15	16
Twinspan cluster level 5-6		0	1	0	1	0	1		0-0	0-1	1-0	1-1		0	1	0	1	0	1
No. of relevés		301	135	994	235	846	765	760	1324	730	1316	177	709	65	690	174	250	192	66
Anthyllis vulneraria	1755	5	9	34	62	42	12	10	9	5	1	.	14	.	29	32	29	38	.
Pilosella officinarum	1746	22	36	18	24	21	6	29	23	14	19	3	23	3	8	16	2	4	2
Galium mollugo agg.	1608	30	70	37	28	27	16	13	7	7	4	1	9	8	24	.	.	22	65
Elytrigia intermedia	1514	5	1	4	.	5	37	17	19	38	23	12	16	6	1	1	.	.	.
Salvia verticillata	1504	8	19	23	6	30	24	13	6	23	15	3	12	5	9	1	.	7	6
Phleum phleoides	1376	4	.	9	.	16	27	19	25	19	6	3	21	6	7	1	1	.	5
Convolvulus arvensis	1374	26	31	12	3	9	6	19	11	18	35	32	8	2	1	.	1	1	.
Veronica spicata	1362	7	1	5	15	11	17	17	21	18	10	13	25	.	9	17	10	1	.
Clinopodium acinos	1351	4	16	4	.	9	3	16	32	14	6	2	27	8	17	18	24	9	5
Campanula sibirica	1345	2	10	2	.	7	16	10	8	34	15	12	22	5	24	34	16	1	29
Echium vulgare	1322	7	10	5	6	12	5	16	29	13	15	2	23	3	11	10	.	2	2
Medicago lupulina	1302	33	7	27	35	16	8	18	11	9	15	3	7	.	2	.	.	3	2
Inula ensifolia	1267	1	.	2	.	12	40	5	4	28	2	1	15	.	38	15	4	17	11
Thesium linophyllon	1242	2	.	15	13	21	39	8	5	13	3	1	10	.	16	16	17	1	2
Arenaria serpyllifolia agg.	1194	5	1	3	.	7	2	11	31	8	8	9	23	2	9	25	38	5	3
Bupleurum falcatum	1121	1	.	8	.	21	31	7	4	10	4	.	13	2	26	8	.	14	8
Pilosella bauhini	1117	14	10	17	19	15	9	11	13	10	2	.	12	2	12	33	13	3	.
Galium glaucum	1115	1	.	2	.	5	25	7	15	32	5	.	18	11	13	8	5	11	.
Thalictrum minus	1079	3	4	2	5	7	26	9	3	23	16	11	11	5	15	9	9	10	3
Adonis vernalis	990	.	.	2	.	2	32	8	4	35	11	22	13	.	2	1	5	.	.
Senecio jacobaea	916	7	41	10	4	7	5	12	7	14	16	10	10	.	1	5	2	.	.
Allium flavum	842	.	.	.	.	1	2	6	17	15	2	1	21	37	20	24	16	.	15
Veronica austriaca	832	3	2	9	11	10	16	6	4	8	13	7	8	.	9	.	.	5	17
Euphorbia seguieriana	829	.	.	1	.	2	4	5	6	17	17	46	9	.	1	17	49	.	.
Sedum sexangulare	829	5	.	7	20	5	1	11	24	1	1	.	16	6	9	14	7	1	2
Silene otites agg.	818	.	.	1	.	3	3	6	16	16	4	9	17	2	8	38	31	1	3
Jurinea mollis	802	1	.	1	.	2	12	3	3	29	8	27	14	.	8	23	13	.	5
Sedum acre	787	1	4	1	.	3	1	10	24	3	5	2	19	15	12	4	.	7	5
Elytrigia repens	766	17	9	3	.	4	5	15	8	6	23	9	4	.	1	.	.	.	.
Trifolium alpestre	734	5	4	14	.	7	23	9	9	3	4	.	5	.	3	.	.	1	.
Origanum vulgare	717	6	6	9	2	15	17	7	5	3	6	.	6	2	8	.	.	13	2
Genista tinctoria	705	6	21	13	4	6	14	8	4	5	8	1	7	5	4	1	.	1	.
Geranium sanguineum	699	1	.	8	10	9	22	5	6	2	1	1	7	2	13	.	.	10	9
Arabis hirsuta agg.	689	1	1	15	13	15	4	5	5	2	1	.	7	.	13	7	2	14	11
Trifolium campestre	680	28	9	10	11	3	2	12	13	3	7	.	4	.	1	.	.	.	.
Galatella linosyris	671	1	.	1	.	5	20	6	8	23	1	1	10	.	2	7	5	.	.
Bromus inermis	659	2	15	1	.	3	10	13	2	5	23	13	3	.	.	.	.	.	.
Verbascum lychnitis	657	1	8	1	.	3	3	9	10	10	13	5	11	.	7	.	.	.	12
Picris hieracioides	640	14	8	6	24	13	7	8	4	5	8	3	5	.	.	.	.	1	.
Poa compressa	636	5	35	4	1	8	2	11	9	5	8	6	8	2	3	2	1	3	.
Peucedanum oreoselinum	600	2	.	8	49	13	10	4	4	1	1	.	4	.	7	1	5	1	5

Group number		B1	B2	B3	B4	B5	B6	B7	F1	F2	F3	F4	S1	S2	S3	S4	S5	S6	S7
Twinspan cluster level 4		7	7	8	8	9	9	10	10	10	10	10	10	13	13	14	14	15	16
Twinspan cluster level 5-6		0	1	0	1	0	1		0-0	0-1	1-0	1-1		0	1	0	1	0	1
No. of relevés		301	135	994	235	846	765	760	1324	730	1316	177	709	65	690	174	250	192	66
Cichorium intybus	597	21	66	4	1	4	3	9	3	3	16	1	1	.	1	.	.	.	.
Trifolium repens	594	33	12	19	1	2	1	9	3	2	8	1	1	.	1	.	.	1	.
Calamagrostis epigejos	588	15	15	7	.	11	9	10	4	3	9	1	2	.	.	1	.	.	.
Silene vulgaris	582	9	9	13	13	14	7	5	3	2	2	.	4	2	4	.	1	5	8
Poa bulbosa	552	1	.	1	.	1	1	2	16	4	6	23	11	.	1	5	23	.	.
Stipa pulcherrima	551	.	.	1	.	1	5	3	6	27	2	2	15	.	5	12	4	.	.
Euphorbia esula	544	10	27	8	.	9	9	6	2	6	9	1	1	.	1	.	1	.	.
Cytisus nigricans	543	1	.	2	2	8	19	3	2	9	1	.	7	2	12	1	.	2	8
Hylotelephium maximum agg.	527	3	.	1	.	2	2	6	12	2	3	.	11	34	10	.	.	9	12
Verbascum chaixii	517	4	1	2	1	5	10	6	11	3	2	.	6	.	8	1	1	3	3
Rumex acetosella	501	9	.	1	.	1	.	11	15	1	6	.	10	18	1	2	.	.	.
Fragaria vesca	496	13	3	17	3	8	4	4	3	1	1	.	4	.	2	.	.	19	2
Hippocrepis comosa	490	1	.	3	50	12	3	1	1	1	.	.	3	.	12	12	4	14	.
Alyssum alyssoides	467	1	.	1	.	2	1	4	15	9	2	1	11	8	3	2	2	.	.
Rostraria cristata	450	1	.	1	.	4	11	6	4	8	3	.	7	3	1	8	14	.	.
Stipa joannis	427	.	.	1	.	3	5	5	6	6	6	3	8	.	3	6	2	.	.
Helictochloa pratensis	426	4	.	2	.	8	6	6	8	2	1	.	11	.	1	2	1	.	.
Chrysopogon gryllus	413	3	.	.	1	1	4	3	5	16	5	.	4	2	1	3	16	.	.
Seseli hippomarathrum	400	.	.	1	.	2	3	4	6	7	1	.	9	.	2	20	20	1	.
Microthlaspi perfoliatum	390	1	.	2	.	3	2	4	10	5	1	1	8	.	2	10	7	.	.
Silene nutans	389	4	4	11	25	6	3	2	2	1	1	.	2	.	5	.	.	3	15
Campanula rapunculoides	386	4	21	8	1	8	6	2	1	1	1	.	2	.	4	.	.	21	6
Inula hirta	385	1	.	3	7	4	18	3	2	5	2	.	2	.	2	4	1	.	.
Cytisus hirsutus	373	.	.	6	12	6	5	2	2	1	1	.	2	.	11	1	.	6	23
Anthemis tinctoria	367	1	20	1	.	4	2	4	9	3	3	16	3	.	1	.	.	2	2
Muscari comosum	365	3	1	3	.	2	6	5	6	12	3	.	3	.	.	.	.	.	.
Melampyrum arvense	361	2	1	2	.	9	10	3	3	7	1	.	4	.	1	1	.	.	.
Cerastium pumilum	354	3	.	1	.	2	1	4	9	1	1	1	6	.	1	6	37	.	.
Festuca ovina agg.	353	4	2	6	10	6	1	7	4	.	1	.	6	5	2	.	.	.	.
Euphrasia stricta agg.	351	4	19	2	3	5	2	12	4	2	1	.	5	.	3	1	.	2	.
Medicago minima	340	.	.	1	.	1	2	2	10	7	3	8	5	.	1	2	3	1	2
Helichrysum arenarium	338	1	25	.	.	1	1	4	4	3	11	8	5	.	1	2	.	.	.
Hieracium umbellatum	337	3	8	2	.	5	11	5	3	1	4	1	2	2	1	.	.	.	.
Stellaria graminea	333	10	8	11	.	1	1	3	1	1	10	2	.	.	.	.	.	.	.
Melilotus officinalis	328	4	5	1	1	4	2	3	3	7	7	11	3	.	1	.	.	.	.
Tragopogon dubius	327	2	.	1	.	1	2	2	4	11	8	6	3	.	1	.	.	.	.
Potentilla recta	326	3	.	2	14	3	4	3	5	5	4	6	2	2	1	.	.	.	.
Vicia sativa	323	19	1	5	1	3	2	4	4	2	5	.	1	.	.	.	.	.	.
Viscaria vulgaris	323	4	3	5	.	1	1	8	6	1	4	1	2	25	1	.	.	.	.
Ajuga genevensis	319	7	4	3	2	3	2	4	2	1	7	1	3	.	3	.	.	3	.
Astragalus monspessulanus	317	1	.	1	.	1	6	3	1	22	1	.	7	.	2	1	.	.	.

Group number		B1	B2	B3	B4	B5	B6	B7	F1	F2	F3	F4	S1	S2	S3	S4	S5	S6	S7
Twinspan cluster level 4		7	7	8	8	9	9	10	10	10	10	10	10	13	13	14	14	15	16
Twinspan cluster level 5-6		0	1	0	1	0	1		0-0	0-1	1-0	1-1		0	1	0	1	0	1
No. of relevés		301	135	994	235	846	765	760	1324	730	1316	177	709	65	690	174	250	192	66
Cytisus albus	315	.	.	2	.	2	16	3	1	8	1	.	3	.	2	.	.	.	2
Asparagus officinalis	304	2	1	.	.	1	3	3	2	12	8	5	3	.	.	.	.	.	.
Erophila verna	303	2	.	1	.	1	1	2	10	2	2	1	7	2	1	6	10	.	.
Vinca herbacea	302	.	.	.	.	1	2	2	1	18	4	12	6	.	1	1	2	.	2
Artemisia absinthium	298	1	17	.	.	1	1	4	3	2	12	4	2	.	.	.	.	.	.
Pulsatilla pratensis	296	1	.	1	1	3	2	3	5	6	6	2	3	.	.	2	.	.	.
Veronica arvensis	296	11	.	5	3	1	1	4	8	1	3	2	1	2	.	1	2	.	.
Leontodon crispus	293	1	.	1	.	1	5	2	1	14	1	.	8	.	7	1	.	.	9
Linaria vulgaris	291	5	18	2	.	3	2	7	2	2	7	.	1	.	1	.	.	1	.
Campanula persicifolia	283	2	1	12	2	5	6	1	1	.	1	.	2	.	2	.	.	6	3
Seseli libanotis	283	.	1	4	.	5	5	1	1	1	2	.	3	.	3	.	.	23	6
Cerastium arvense	280	6	1	4	.	8	1	5	3	1	2	1	2	.	2	.	.	2	6
Knautia kitaibelii	276	1	.	13	.	6	3	1	1	1	.	.	.	.	2	.	.	18	.
Linum hirsutum	274	.	.	1	.	2	6	2	1	7	6	27	2	.	1	.	.	.	.
Carduus acanthoides	268	6	1	1	.	1	2	6	3	4	7	1	1	.	.	.	.	.	.
Reseda lutea	257	1	.	.	.	2	1	2	2	4	5	22	3	2	1	3	4	.	.
Vicia tetrasperma	253	14	4	3	1	1	1	4	3	1	7	.	1	.	.	.	.	.	.
Asperula tinctoria	251	.	.	3	.	2	3	1	1	1	1	.	2	.	13	5	2	15	.
Erigeron annuus	248	13	3	4	12	3	2	3	1	1	4	1	1	.	1	.	.	.	.
Agrostis vinealis	245	2	.	2	.	1	1	7	4	.	4	.	8	.	1	.	.	.	.
Pilosella echioides	245	.	.	.	.	1	1	2	7	3	3	3	7	.	1	3	1	.	.
Galium boreale agg.	243	2	1	7	6	3	5	2	1	2	2	.	2	.	1	.	.	3	.
Viola arvensis	243	2	.	1	.	1	1	3	9	1	3	1	4	.	1	1	.	1	.
Seseli pallasii	242	.	.	1	.	1	4	3	2	10	1	.	5	2	4	1	.	.	.
Dictamnus albus	241	.	.	1	.	1	8	2	2	9	1	.	5	.	2	1	1	.	2
Vicia hirsuta	239	13	.	3	.	2	.	4	5	1	2	1	2	.	1	.	.	.	.
Anemone sylvestris	237	1	1	1	.	4	10	4	1	2	3	.	2	.	1	.	.	1	.
Festuca pseudodalmatica	236	.	.	.	.	1	1	3	11	.	1	.	5	25	1	.	.	.	.
Danthonia decumbens	235	3	.	12	18	3	1	2	1	.	1	.	1	.	.	.	.	.	.
Vicia tenuifolia	233	2	1	6	.	5	8	2	1	2	2	.	1	.	.	.	.	.	.
Thesium arvense	232	1	.	1	.	1	2	3	2	5	6	8	2	.	1	2	.	.	.
Petrorhagia saxifraga	224	1	1	1	3	2	.	3	6	4	2	.	3	.	1	3	4	.	.
Bromus hordeaceus	223	6	.	1	.	1	.	2	8	1	5	.	1	.	.	.	.	1	.
Erigeron acris	223	4	30	2	4	2	1	4	2	1	2	.	1	.	1	.	.	1	.
Erysimum diffusum	222	.	.	.	.	1	1	1	5	6	2	5	4	2	1	3	5	.	.
Muscari tenuiflorum	222	1	.	1	.	1	2	2	2	13	2	2	4	.	1	1	.	.	.
Cephalaria uralensis	221	.	.	.	.	.	1	1	1	14	1	22	6	.	1	.	.	.	.
Cerastium semidecandrum	221	1	.	.	.	1	1	4	7	1	3	.	2	.	1	3	1	.	.
Muscari neglectum	220	1	.	1	.	1	1	3	3	2	2	1	6	.	1	2	13	.	.
Stipa lessingiana	220	.	.	.	.	.	1	1	1	15	2	29	4	.	.	.	.	.	.
Odontites luteus	215	.	.	1	.	3	2	2	4	4	1	3	5	.	1	3	8	.	.

Group number		B1	B2	B3	B4	B5	B6	B7	F1	F2	F3	F4	S1	S2	S3	S4	S5	S6	S7
Twinspan cluster level 4		7	7	8	8	9	9	10	10	10	10	10	10	13	13	14	14	15	16
Twinspan cluster level 5-6		0	1	0	1	0	1		0-0	0-1	1-0	1-1		0	1	0	1	0	1
No. of relevés		301	135	994	235	846	765	760	1324	730	1316	177	709	65	690	174	250	192	66
Cerastium brachypetalum	212	2	.	2	8	1	2	1	6	1	1	.	2	.	1	4	10	.	.
Cytisus ruthenicus	212	.	18	1	.	2	5	5	1	1	4	5	1	.	.	.	.	.	.
Taraxacum sect. Erythrosperma	212	1	.	1	.	1	1	2	6	1	1	.	5	.	3	3	8	1	.
Eryngium planum	211	2	10	1	.	1	4	2	1	3	7	.	1	.	1	.	.	.	.
Peucedanum alsaticum	210	2	.	1	.	3	13	2	1	3	2	.	.	.	.	.	.	.	.
Pastinaca sativa	209	14	10	4	9	4	1	3	1	.	1	.	1	.	.	.	.	.	.
Veronica orchidea	208	1	.	3	.	2	2	1	1	10	1	.	6	.	1	1	.	.	3
Bromus squarrosus	207	.	.	.	.	.	1	1	5	5	4	18	2	.	.	.	.	.	.
Campanula bononiensis	204	1	.	1	1	2	9	3	1	1	3	.	2	.	1	1	.	.	.
Scorzonera hispanica	203	.	.	1	.	1	8	1	1	9	.	.	3	.	.	1	.	.	2
Crepis biennis	202	6	3	12	3	2	1	1	1	1	1	.	1	.	1	.	.	.	.
Cynodon dactylon	202	4	.	1	.	1	1	4	4	1	7	1	1	.	.	1	.	.	.
Oxytropis pilosa	202	.	.	.	.	1	3	1	1	10	4	8	3	.	1	1	.	.	.
Laserpitium latifolium	199	.	.	4	5	3	3	1	.	.	.	.	.	.	3	.	.	31	9
Allium sphaerocephalon	197	1	.	.	.	1	1	2	4	5	2	1	3	2	1	10	2	.	.
Galium octonarium	197	.	.	.	.	.	1	1	.	1	11	19	1	.	.	.	.	.	.
Hypericum elegans	197	.	.	.	.	1	2	1	1	7	6	6	1	.	1	.	.	.	.
Potentilla pusilla agg.	197	2	1	4	2	6	1	3	2	1	.	.	2	.	2	.	.	2	.
Valeriana officinalis	197	1	7	6	.	1	4	2	1	1	1	.	1	.	3	.	.	1	20
Allium oleraceum	196	2	24	2	.	3	1	2	2	1	1	.	2	.	1	.	.	1	.
Salvia austriaca	193	1	.	1	.	1	4	1	1	11	2	6	3	.	1	.	.	.	.
Phlomis tuberosa	192	1	.	1	.	1	2	3	1	2	8	8	1	.	.	.	.	.	.
Cuscuta epithymum	191	1	.	3	2	3	2	3	3	2	1	.	1	.	1	3	1	1	.
Cytisus ratisbonensis	191	.	.	1	.	6	3	2	2	1	.	.	4	.	1	2	3	.	.
Viola canina	191	2	9	12	7	1	1	1	1	1	1	.	1	.	1	.	.	.	.
Viola rupestris	191	1	1	1	.	6	2	2	1	1	1	1	3	.	1	6	4	2	.
Lathyrus tuberosus	189	11	1	3	2	3	1	2	1	1	4	2	.	.	.	.	.	.	.
Saxifraga tridactylites	188	.	.	.	.	.	.	1	5	.	.	.	5	.	4	7	14	2	9
Holosteum umbellatum	187	1	.	1	.	1	1	1	6	2	2	5	4	.	1	3	6	.	.
Rosa gallica	187	2	.	4	.	2	4	2	2	5	1	.	2	.	.	.	.	.	.
Potentilla alba	186	2	.	10	7	2	5	1	.	1	1	.	.	.	1	.	.	.	.
Cirsium arvense	182	10	1	7	.	3	1	2	1	1	2	.	1	.	.	.	.	.	.
Euphrasia rostkoviana agg.	181	1	.	10	2	2	2	1	1	1	1	.	1	.	2	.	.	8	.
Pilosella cymosa	181	1	.	1	.	1	2	1	1	2	6	1	2	2	3	1	.	.	.
Medicago prostrata	178	1	.	1	.	1	4	2	2	1	2	.	3	.	2	2	8	.	.
Rhinanthus angustifolius	177	2	1	5	6	4	4	2	1	1	1	.	1	.	1	.	.	4	.
Marrubium peregrinum	176	1	.	.	.	.	1	1	1	2	7	30	1	.	.	.	.	.	.
Minuartia verna agg.	173	.	.	1	.	1	.	1	2	1	.	.	4	2	4	11	16	.	15
Podospermum purpureum	173	1	2	2	.	2	7	2	1	2	1	1	1	.	3	1	1	.	.
Potentilla inclinata	173	1	.	1	.	1	.	1	1	.	9	4	1	2	1	.	.	.	.
Teucrium polium	173	.	.	.	.	.	.	.	1	1	3	66	1	.	.	.	.	.	.

Group number		B1	B2	B3	B4	B5	B6	B7	F1	F2	F3	F4	S1	S2	S3	S4	S5	S6	S7
Twinspan cluster level 4		7	7	8	8	9	9	10	10	10	10	10	10	13	13	14	14	15	16
Twinspan cluster level 5-6		0	1	0	1	0	1		0-0	0-1	1-0	1-1		0	1	0	1	0	1
No. of relevés		301	135	994	235	846	765	760	1324	730	1316	177	709	65	690	174	250	192	66
Veronica praecox	171	.	.	1	.	.	1	2	2	1	8	.	2	.	1	1	7	.	.
Carduus nutans	170	3	6	1	1	1	1	2	4	2	3	3	2	.	1	2	1	.	.
Myosotis arvensis	169	6	1	6	.	1	1	1	2	1	1	.	1	.	.	2	.	.	.
Seseli tortuosum	168	1	.	.	.	.	1	1	1	1	8	21	1	.	.	.	.	.	.
Carex liparocarpos	167	.	.	.	3	1	1	2	3	1	1	.	3	.	1	4	18	.	.
Knautia drymeia	167	2	1	3	41	3	1	1	1	.	1	.	.	.	.	.	.	1	.
Carex muricata agg.	166	8	21	1	1	2	1	3	1	1	1	.	1	.	1	.	.	2	.
Carex hirta	165	9	3	3	.	.	1	3	1	.	3	.	1	.	.	.	.	.	.
Myosotis stricta	163	1	1	.	.	1	1	1	6	1	2	1	5	.	1	2	1	.	.
Sideritis montana	163	.	.	.	.	.	.	1	3	6	3	18	2	.	1	1	1	.	.
Silene latifolia	162	3	1	1	.	1	1	5	2	1	5	1	1	2	1	.	.	.	.
Holcus lanatus	160	6	1	8	18	1	1	1	1	1	1	.	.	.	.	.	.	.	.
Petrorhagia prolifera	159	1	.	.	.	1	.	2	8	1	1	.	1	.	1	1	.	.	.
Solidago virgaurea	159	1	10	3	1	2	5	1	1	1	1	.	1	2	1	.	.	3	6
Scorzoneroides autumnalis	158	5	4	7	.	3	1	2	1	1	1	1	1	.	1	.	.	.	.
Carex supina	157	.	.	.	.	1	1	1	6	3	1	2	4	.	1	1	.	.	.
Cruciata pedemontana	157	1	.	.	.	1	1	2	7	1	2	.	1	.	.	.	1	.	.
Myosotis ramosissima	157	5	.	1	2	1	1	3	6	1	1	.	1	.	1	1	1	.	.
Alopecurus pratensis	155	16	.	4	1	1	1	1	1	.	2	.	.	.	1	.	.	.	.
Scabiosa triandra	155	1	.	1	43	5	.	.	1	.	.	.	.	.	.	.	.	.	.
Senecio erucifolius	154	1	1	1	.	3	2	1	1	1	5	3	1	.	1	.	.	.	.
Centaurea diffusa	153	1	.	.	.	.	1	1	1	4	7	8	1	.	.	.	.	.	.
Arabidopsis thaliana	152	2	.	1	.	1	1	2	6	1	1	.	3	.	1	1	4	3	.
Astragalus cicer	152	3	16	3	.	2	3	3	1	1	2	.	1	.	.	.	.	.	.
Cynosurus cristatus	152	8	.	10	4	1	1	1	1	.	1	.	1	.	.	.	.	.	.
Lathyrus latifolius	151	.	.	7	1	3	5	1	1	1	.	.	.	.	.	.	.	.	.
Phleum pratense agg.	151	5	27	5	.	1	1	1	1	.	1	.	.	.	.	.	.	1	.
Ajuga laxmannii	148	.	.	.	.	1	2	1	1	12	1	.	3	.	.	.	.	.	.
Astragalus glycyphyllos	147	6	3	3	3	3	2	2	1	1	1	.	1	.	1	.	.	.	.
Gentiana cruciata	147	1	8	5	1	4	4	1	1	.	.	.	1	.	1	.	.	1	.
Odontites vulgaris agg.	147	6	5	1	.	1	1	3	2	1	3	.	1	.	1	.	1	.	.
Potentilla reptans	147	13	7	6	1	1	1	1	1	1	1	.	1	.	.	.	.	.	.
Trifolium ochroleucon	147	9	.	6	.	1	1	2	1	1	1	.	1	.	.	.	.	.	.
Equisetum arvense	146	4	19	5	.	1	1	2	1	.	2	.	.	.	.	.	.	.	.
Heracleum sphondylium	146	3	2	8	.	1	3	1	.	.	1	.	.	.	.	.	.	8	.
Hypochaeris radicata	146	4	.	3	1	1	1	3	2	1	1	.	2	.	.	.	.	1	.
Viola tricolor	145	1	2	1	.	.	.	2	3	.	1	.	3	8	3	.	.	2	12
Bromus riparius	143	.	.	.	.	.	1	1	1	1	5	37	1	.	1	.	.	.	2
Anacamptis morio	142	1	.	3	23	1	1	2	1	1	1	.	1	.	.	.	.	.	.
Anthyllis macrocephala	141	1	17	1	.	1	1	3	1	2	3	.	2	.	.	.	.	.	.
Iris aphylla	141	.	.	1	.	.	4	1	1	6	1	.	2	.	1	.	.	.	.

Group number		B1	B2	B3	B4	B5	B6	B7	F1	F2	F3	F4	S1	S2	S3	S4	S5	S6	S7
Twinspan cluster level 4		7	7	8	8	9	9	10	10	10	10	10	10	13	13	14	14	15	16
Twinspan cluster level 5-6		0	1	0	1	0	1		0-0	0-1	1-0	1-1		0	1	0	1	0	1
No. of relevés		301	135	994	235	846	765	760	1324	730	1316	177	709	65	690	174	250	192	66
Pontechium maculatum	140	.	.	2	.	1	8	1	1	4	1	.	2	.	1	.	.	.	.
Lepidium campestre	139	2	.	1	.	1	1	2	5	1	1	.	2	2	1	1	.	.	.
Lolium perenne	137	9	1	2	.	1	1	3	1	1	4	1	1	.	.	.	.	.	.
Helictochloa praeusta	136	1	.	1	.	1	11	1	1	1	1	.	1	.	.	.	1	.	.
Rhinanthus rumelicus	136	6	.	3	.	1	5	1	1	1	2	.	1	.	.	.	.	.	.
Digitalis grandiflora	135	.	1	3	.	1	1	1	1	.	1	.	1	.	3	.	.	19	14
Prunus fruticosa	135	.	.	1	.	1	6	1	2	1	1	1	2	.	1	1	.	.	.
Galium lucidum	134	1	.	1	1	2	1	1	1	1	.	.	6	.	2	8	5	1	.
Orlaya grandiflora	134	1	.	.	.	1	1	1	4	3	1	.	4	.	1	.	1	.	2
Plantago argentea	134	1	.	1	1	1	2	1	.	5	.	.	4	3	3	1	5	.	.
Veronica verna	134	1	.	1	.	1	.	1	6	.	1	1	2	5	1	1	.	.	.
Fragaria moschata	133	1	.	5	1	2	1	1	1	1	.	.	2	.	1	.	.	1	.
Artemisia vulgaris	132	4	15	1	.	2	1	4	1	1	2	.	1	.	.	.	.	.	.
Cirsium vulgare	132	7	4	1	.	1	1	2	1	1	3	2	.	.	.	.	.	.	.
Sesleria heuflerana	132	.	.	.	.	1	5	1	1	2	1	.	1	.	3	.	.	.	2
Ajuga chamaepitys	131	.	1	.	.	.	.	1	1	4	3	23	1	.	.	.	.	.	.
Centaurium erythraea	130	6	.	3	1	2	1	2	1	2	1	.	1	.	.	.	.	.	.
Scabiosa columbaria	130	1	.	1	11	3	1	1	1	.	.	.	2	.	4	.	.	1	12
Ajuga reptans	129	1	.	9	4	1	1	1	1	.	1	.	.	.	1	.	.	2	.
Centaurea phrygia	129	3	.	7	.	1	3	1	1	1	.	.	.	.	1	.	.	.	.
Genista sagittalis	129	1	.	1	37	2	1	1	1	.	1	.	.	.	.	.	.	.	.
Trinia glauca	128	.	.	1	.	1	1	1	1	2	.	.	5	.	1	6	9	.	.
Phlomis pungens	126	.	.	.	.	.	.	.	1	1	5	31	.	.	.	.	.	.	.
Cerinthe minor	125	1	.	2	1	2	3	2	1	1	2	2	1	.	1	.	.	.	.
Verbascum nigrum	124	1	.	1	.	2	1	1	1	1	4	1	2	.	1	.	.	2	.
Convolvulus cantabrica	123	1	.	.	.	1	1	1	2	4	2	.	2	2	1	1	5	.	.
Minuartia rubra	123	1	.	1	.	1	2	1	2	1	.	.	1	.	1	10	2	.	.
Stipa tirsa	123	.	.	1	.	1	6	1	2	3	1	1	1	.	.	.	.	.	.
Trifolium rubens	123	1	.	5	3	1	4	1	1	1	1	.	1	.	1	.	.	.	.
Medicago x varia	122	7	.	3	3	2	1	1	1	.	2	1	.	.	.	.	.	.	.
Armeria maritima	121	1	.	1	.	1	.	2	4	.	.	.	6	.	1	1	.	.	.
Euphorbia epithymoides	121	.	.	1	.	1	4	1	1	1	1	.	2	.	5	.	.	4	2
Geranium columbinum	121	3	.	1	.	1	1	2	5	.	1	.	2	.	1	.	.	.	.
Stachys germanica	121	3	.	1	.	1	2	2	1	1	3	.	1	.	.	.	.	.	.
Vicia villosa	121	1	.	1	.	1	1	1	1	3	5	1	2	.	.	.	.	.	.
Artemisia pontica	120	.	.	.	.	.	1	1	1	11	1	.	2	.	1	.	.	.	.
Inula oculus-christi	120	.	.	.	.	1	1	1	5	2	1	1	2	.	1	1	1	.	.
Lactuca perennis	120	.	.	.	.	.	1	1	3	1	.	.	4	2	5	.	.	1	2
Fallopia convolvulus	119	2	1	.	.	1	1	2	3	1	1	.	3	.	1	.	.	1	.
Salvia transylvanica	119	1	.	1	.	.	1	1	.	9	1	.	3	.	.	.	.	.	.
Veronica incana	119	.	1	1	.	1	1	1	2	3	3	1	2	.	.	.	.	.	.

Group number		B1	B2	B3	B4	B5	B6	B7	F1	F2	F3	F4	S1	S2	S3	S4	S5	S6	S7
Twinspan cluster level 4		7	7	8	8	9	9	10	10	10	10	10	10	13	13	14	14	15	16
Twinspan cluster level 5-6		0	1	0	1	0	1		0-0	0-1	1-0	1-1		0	1	0	1	0	1
No. of relevés		301	135	994	235	846	765	760	1324	730	1316	177	709	65	690	174	250	192	66
Serratula tinctoria	118	2	.	7	.	1	4	1	.	1	1	.	1	.	1	.	.	1	.
Ornithogalum kochii	117	1	.	2	.	1	1	1	3	1	1	1	2	.	1	1	.	.	.
Calamagrostis varia	115	.	.	1	.	1	.	.	.	.	.	.	1	.	2	.	.	36	.
Erodium cicutarium	115	1	.	1	.	.	.	2	5	1	2	1	1	.	.	.	.	.	.
Melampyrum nemorosum	114	2	.	5	.	1	2	1	1	.	1	.	1	.	2	.	.	4	.
Pulsatilla montana	114	.	.	1	2	1	4	1	.	5	.	.	2	.	2	.	.	.	.
Verbascum thapsus agg.	113	1	2	1	.	1	1	1	3	1	3	.	2	2	1	.	.	1	.
Jasione montana	112	1	1	1	.	1	.	5	3	.	1	.	3	.	.	.	.	.	.
Asyneuma canescens	111	.	.	1	.	1	2	1	1	4	2	3	2	.	1	.	.	.	.
Crambe tataria	111	.	.	.	.	1	2	1	1	8	1	.	3	.	.	.	.	.	.
Erigeron canadensis	111	1	2	.	.	1	.	3	3	1	3	.	1	.	.	.	.	.	.
Xeranthemum annuum	111	.	.	.	.	.	.	1	2	4	3	1	1	5	.	.	.	.	.
Galatella villosa	110	.	.	.	.	.	.	.	1	1	3	34	1	.	.	.	.	.	.
Verbascum phlomoides	110	1	.	.	.	.	1	1	3	3	2	.	1	.	1	.	.	.	.
Viola collina	109	.	4	1	.	2	1	1	1	1	1	.	2	.	4	1	1	3	.
Genista januensis	108	.	.	1	11	3	1	.	1	1	.	.	1	.	3	.	.	.	2
Sanguisorba officinalis	108	2	.	7	.	1	2	1	1	.	1	.	1	.	1	.	1	2	.
Klasea radiata	107	.	.	1	.	.	3	1	1	7	1	.	2	.	.	.	.	.	.
Neotinea ustulata	107	.	.	2	14	2	1	1	1	1	.	.	1	.	1	.	.	.	.
Rhinanthus glacialis	107	.	.	.	36	3	.	.	.	.	.	.	.	.	.	.	.	.	.
Veronica officinalis	107	1	.	6	5	1	.	1	1	2	1	.	1	.	1	.	.	1	.
Carduus hamulosus	106	1	.	.	.	.	1	1	.	9	1	.	3	.	.	.	.	.	.
Carex pallescens	106	1	.	8	6	1	1	1	.	1	1	.	.	.	.	.	.	.	.
Pulmonaria mollis	106	2	.	4	.	2	4	1	1	.	1	.	1	.	.	.	.	.	.
Orchis militaris	105	.	.	2	1	3	3	1	1	1	.	.	1	.	1	1	.	.	.
Orobanche gracilis	105	.	.	1	20	2	.	1	1	1	.	.	2	.	1	.	2	.	.
Trifolium dubium	105	8	.	4	.	1	.	1	2	.	1	.	1	.	.	.	.	.	.
Crepis foetida	103	.	.	1	.	1	1	2	2	3	2	3	1	.	1	.	1	.	.
Hieracium virosum	103	.	.	.	.	.	.	1	1	3	4	2	1	.	1	.	.	.	.
Glechoma hederacea agg.	102	7	.	3	.	1	2	1	1	1	1	.	1	.	1	.	.	1	.
Inula britannica	102	4	.	1	.	1	1	2	1	1	3	1	1	.	.	.	.	.	.
Rumex thyrsiflorus	102	4	1	1	.	1	.	1	1	.	3	.	1	.	.	.	.	.	.
Carex ornithopoda	100	1	.	1	8	2	.	.	1	.	.	.	1	.	1	.	.	21	.
Arabis auriculata	99	.	.	1	.	1	1	1	3	1	.	.	2	.	1	7	2	.	.
Neotinea tridentata	99	.	.	1	24	2	1	1	1	1	.	.	1	.	1	.	2	.	.
Allium carinatum	97	.	.	2	21	2	1	1	1	.	.	.	1	.	1	.	.	.	.
Pimpinella major	97	1	1	3	.	1	1	1	.	1	1	.	1	.	1	.	.	16	.
Asplenium septentrionale	96	.	.	.	.	.	.	1	2	.	.	.	2	62	1	.	.	.	3
Globularia cordifolia	96	.	.	.	5	1	.	1	.	1	.	.	2	.	3	10	3	4	.
Bromus tectorum	95	.	.	.	.	.	.	1	3	1	2	1	1	.	.	.	.	.	.
Pilosella piloselloides	95	1	.	1	.	1	1	1	1	3	2	.	1	.	1	.	.	1	.

Group number		B1	B2	B3	B4	B5	B6	B7	F1	F2	F3	F4	S1	S2	S3	S4	S5	S6	S7
Twinspan cluster level 4		7	7	8	8	9	9	10	10	10	10	10	10	13	13	14	14	15	16
Twinspan cluster level 5-6		0	1	0	1	0	1		0-0	0-1	1-0	1-1		0	1	0	1	0	1
No. of relevés		301	135	994	235	846	765	760	1324	730	1316	177	709	65	690	174	250	192	66
Polygala sibirica	95	.	.	.	.	.	.	1	1	5	3	3	1	.	.	.	.	.	.
Agrostis stolonifera	94	4	1	2	1	1	2	2	1	1	1	.	1	.	.	.	.	.	.
Arabis glabra	94	1	.	1	.	1	1	1	3	1	1	.	1	.	1	.	.	.	.
Prunus tenella	94	.	.	.	.	.	1	1	1	8	.	.	3	.	.	.	.	.	.
Carduus collinus	93	1	.	1	.	.	1	1	4	1	1	.	2	.	2	.	.	1	.
Brassica elongata	92	.	.	.	.	.	1	1	1	8	1	2	1	.	.	.	1	.	.
Tephroseris integrifolia	92	.	.	2	.	1	3	1	1	1	.	.	1	2	3	.	.	3	.
Astragalus dasyanthus	91	.	.	.	.	.	.	1	1	5	2	11	1	.	.	.	.	.	.
Cephalaria radiata	91	.	.	.	.	1	4	1	.	3	.	.	1	.	3	.	.	.	8
Lactuca serriola	90	1	.	1	.	1	1	1	1	1	2	2	1	.	.	.	.	.	.
Rosa spinosissima	89	.	.	.	.	1	2	1	1	1	1	.	1	2	3	.	.	.	2
Scleranthus perennis	89	1	.	1	.	.	.	2	3	.	1	.	4	.	1	1	.	.	.
Veronica dillenii	89	.	.	.	.	1	.	1	4	1	1	.	3	.	.	.	.	.	.
Artemisia alba	88	.	.	.	.	.	.	1	1	1	.	.	4	.	1	.	11	.	.
Aurinia saxatilis	88	.	.	.	.	.	.	1	2	.	1	.	1	54	2	.	.	1	5
Carum carvi	88	2	.	6	.	1	1	1	1	.	1	.	1	.	.	.	.	1	.
Allium scorodoprasum	87	2	.	5	.	1	1	1	1	1	1	1	1	.	1	1	.	1	.
Anacamptis pyramidalis	87	1	.	1	11	2	2	1	1	1	.	.	1	.	1	.	1	.	.
Clematis recta	87	.	.	3	1	1	3	1	1	.	1	.	1	.	1	.	.	.	.
Cynoglossum officinale	87	1	1	.	.	.	1	1	1	3	2	1	1	.	1	.	.	.	.
Dianthus deltoides	87	5	6	2	.	1	1	1	1	1	1	.	1	.	.	.	.	.	.
Dianthus membranaceus	87	.	.	.	.	.	1	1	1	1	4	.	1	.	.	.	.	.	.
Lathyrus pannonicus	87	1	.	2	.	1	4	1	1	1	1	1	1	.	.	.	.	.	.
Melampyrum cristatum	87	.	.	2	.	1	4	1	1	1	1	.	1	.	1	1	1	.	.
Agropyron pectinatum	86	.	.	.	.	.	.	1	1	1	3	23	.	.	.	.	.	.	.
Geranium robertianum	86	1	1	1	.	1	.	1	1	.	1	.	2	2	3	.	.	9	12
Gypsophila paniculata	85	.	.	.	.	.	1	1	1	1	4	1	1	.	.	.	.	.	.
Silene bupleuroides	84	.	.	.	.	.	1	1	1	3	1	5	2	.	1	1	.	1	.
Onosma visianii	83	.	.	.	.	.	.	1	1	1	1	15	1	.	1	16	1	.	.
Camelina sativa agg.	82	.	.	.	.	1	1	1	3	2	1	2	1	.	1	1	.	.	.
Elymus uralensis	82	.	.	.	.	.	1	1	1	4	3	.	.	.	.	.	.	.	.
Psephellus marschallianus	82	.	.	.	.	.	.	.	1	1	1	28	2	.	.	.	.	.	.
Malva thuringiaca	81	.	1	.	.	1	1	1	.	1	5	1	.	.	.	.	.	.	.
Rapistrum perenne	81	.	.	1	.	1	1	2	1	3	1	.	1	.	.	.	.	.	.
Vicia lathyroides	81	2	.	1	2	.	.	1	3	1	1	.	1	.	.	.	.	.	.
Epipactis atrorubens	80	.	.	1	1	1	.	.	.	.	.	.	1	.	6	1	1	11	.
Allium rotundum	79	.	1	.	.	.	1	1	1	5	2	.	1	.	.	.	.	.	.
Dianthus armeria	79	6	.	2	.	1	1	1	1	.	1	.	1	.	.	.	.	.	.
Lappula squarrosa	79	.	.	1	.	.	1	1	2	2	1	2	1	.	1	.	.	.	.
Ranunculus illyricus	79	.	.	.	.	.	1	1	2	1	2	.	2	.	.	.	1	.	.
Gypsophila collina	78	.	.	.	.	.	1	.	.	2	.	20	1	.	3	1	.	.	.

Group number		B1	B2	B3	B4	B5	B6	B7	F1	F2	F3	F4	S1	S2	S3	S4	S5	S6	S7
Twinspan cluster level 4		7	7	8	8	9	9	10	10	10	10	10	10	13	13	14	14	15	16
Twinspan cluster level 5-6		0	1	0	1	0	1		0-0	0-1	1-0	1-1		0	1	0	1	0	1
No. of relevés		301	135	994	235	846	765	760	1324	730	1316	177	709	65	690	174	250	192	66
Molinia caerulea agg.	78	1	.	5	4	1	1	1	.	.	1	.	.	.	1	.	.	1	.
Sempervivum marmoreum	78	.	.	.	.	.	.	1	1	1	.	.	1	3	6	.	.	.	18
Silene chlorantha	78	.	.	.	.	.	.	1	1	1	5	4	1	.	.	.	.	.	.
Allium ericetorum	77	.	.	.	.	1	.	.	1	.	.	.	1	.	6	.	.	16	.
Aquilegia vulgaris	77	.	.	6	1	1	1	.	.	.	1	.	.	.	1	.	.	4	.
Carex digitata	77	.	1	1	.	1	.	1	1	.	.	.	1	.	2	.	.	19	9
Astragalus danicus	76	.	.	4	.	1	1	1	1	1	1	1	1	.	1	.	.	.	.
Hieracium sabaudum	76	1	.	1	.	2	2	1	1	1	.	.	2	3	1	.	.	2	.
Inula conyzae	76	1	1	1	.	2	1	1	1	.	1	.	2	.	1	.	1	2	.
Poa nemoralis	76	.	.	1	.	1	1	1	1	.	1	.	1	2	2	.	.	7	27
Bassia prostrata	75	1	.	.	.	.	.	.	1	1	2	19	.	.	.	.	.	.	.
Cirsium acaulon	75	.	1	3	12	2	.	.	.	.	.	.	.	.	1	.	.	.	.
Hippocrepis emerus	75	.	.	.	.	.	.	.	1	1	.	.	3	.	1	1	15	.	5
Orobanche alba	75	.	.	1	.	1	1	1	1	3	1	.	2	.	1	.	.	.	.
Valerianella locusta	75	3	.	1	.	1	1	1	3	1	1	.	1	.	1	1	.	.	.
Brachypodium sylvaticum	74	4	1	2	1	2	.	1	1	1	1	.	1	.	1	.	.	1	.
Hypericum maculatum	74	1	.	6	.	1	1	.	1	.	.	.	.	.	1	.	.	1	.
Thymus serpyllum	73	1	1	1	1	1	.	2	2	1	1	.	1	.	1	1	.	.	.
Cirsium eriophorum	72	2	.	4	.	1	1	1	1	1	1	.	.	.	.	.	.	.	.
Cytisus procumbens	72	.	.	1	.	1	3	1	1	1	1	.	2	.	1	.	.	2	.
Festuca tatrae	72	.	.	.	.	.	.	.	.	.	.	.	.	.	1	.	.	36	.
Lactuca viminea	72	.	.	1	.	.	.	1	4	1	.	.	1	5	1	1	.	.	.
Primula acaulis	72	.	.	1	20	1	.	.	.	.	.	.	.	.	1	.	.	1	.
Viola kitaibeliana	72	.	.	1	.	.	1	.	4	1	1	.	1	.	1	.	3	.	.
Aconitum anthora	70	.	.	1	.	1	1	1	1	.	.	.	1	.	5	.	.	1	8
Anchusa officinalis	70	.	.	1	.	.	1	1	2	1	2	.	1	.	1	.	.	.	.
Convallaria majalis	70	.	.	3	.	1	1	.	1	.	.	.	1	.	1	.	.	13	.
Melampyrum barbatum	70	1	.	.	.	2	3	1	1	1	1	.	2	3	1	.	1	.	.
Potentilla humifusa	70	.	.	.	.	.	.	.	1	.	4	6	1	.	.	.	.	.	.
Asplenium ceterach	69	.	.	1	.	.	.	.	1	1	.	.	3	.	3	.	.	.	15
Calluna vulgaris	69	.	.	1	6	1	.	1	1	.	1	.	3	2	1	.	.	.	.
Galium ruthenicum	69	.	.	.	.	.	.	1	1	.	3	5	.	.	.	.	.	.	.
Veronica vindobonensis	69	3	.	3	.	1	1	1	1	.	.	.	1	.	1	.	.	.	.
Viola jooi	68	.	.	.	.	1	.	1	1	1	.	.	1	.	7	.	.	.	18
Centaurea orientalis	67	.	.	.	.	1	1	1	.	2	2	9	1	.	.	.	.	.	.
Gentiana verna	67	.	.	1	23	1	.	.	.	.	.	.	.	.	.	.	.	.	.
Iris variegata	67	.	.	1	.	.	2	1	1	1	.	.	2	2	.	.	.	.	.
Nigella arvensis	67	.	.	.	.	.	.	1	1	2	2	6	.	.	.	.	.	.	.
Pistorinia hispanica	67	.	.	.	.	1	.	.	1	1	.	.	2	2	3	.	.	.	21
Setaria viridis	67	1	1	.	.	.	.	1	2	1	1	.	1	.	.	.	.	.	.
Caucalis platycarpos	66	1	.	1	.	.	1	.	2	2	1	.	2	.	.	.	1	.	.

Group number		B1	B2	B3	B4	B5	B6	B7	F1	F2	F3	F4	S1	S2	S3	S4	S5	S6	S7
Twinspan cluster level 4		7	7	8	8	9	9	10	10	10	10	10	10	13	13	14	14	15	16
Twinspan cluster level 5-6		0	1	0	1	0	1		0-0	0-1	1-0	1-1		0	1	0	1	0	1
No. of relevés		301	135	994	235	846	765	760	1324	730	1316	177	709	65	690	174	250	192	66
Lilium martagon	66	.	.	3	1	1	1	.	.	.	.	.	.	.	1	.	.	9	5
Sisymbrium polymorphum	66	.	.	.	.	.	1	.	1	.	1	32	.	.	.	.	.	.	.
Viola odorata	66	1	.	2	.	1	4	1	1	1	1	.	1	.	1	.	.	.	.
Allium podolicum	65	1	.	.	.	.	1	1	2	1	2	1	1	.	1	.	.	.	.
Coronilla vaginalis	65	.	.	1	2	1	.	.	.	.	.	.	.	.	4	.	.	14	.
Allium vineale	64	4	1	1	.	1	1	1	1	1	1	.	1	.	.	1	.	.	.
Antennaria dioica	64	.	.	2	9	1	1	.	.	.	.	.	1	.	1	1	.	2	.
Platanthera bifolia	64	.	.	2	1	1	.	.	.	.	1	.	.	.	2	.	.	10	2
Sedum rupestre	64	.	.	1	.	1	.	1	1	.	.	.	5	5	1	1	.	.	.
Aethionema saxatile	63	.	.	1	.	.	.	.	.	.	.	.	1	.	1	4	18	.	.
Bromus japonicus	63	1	.	.	.	1	.	1	3	.	1	.	1	.	.	.	.	.	.
Cardaria draba	63	1	.	.	.	.	.	1	1	1	2	3	1	.	.	.	.	.	.
Lotus maritimus	63	3	.	1	.	1	1	1	1	1	1	.	1	.	.	.	.	.	.
Peucedanum ruthenicum	63	.	.	.	.	.	1	1	.	5	1	.	3	.	.	.	.	.	.
Trifolium aureum	63	2	10	1	.	1	1	1	1	.	1	.	1	.	.	.	.	.	.
Consolida regalis	62	1	1	.	.	1	.	1	1	1	2	1	1	.	.	.	.	.	.
Hieracium murorum	62	.	.	1	.	1	1	1	.	.	.	.	1	2	2	.	.	15	.
Melilotus albus	62	2	2	1	.	1	.	1	1	1	1	1	1	.	.	.	.	.	.
Seseli gracile	62	.	.	.	.	.	1	.	1	1	.	.	1	.	4	.	.	.	24
Valerianella dentata	62	1	.	.	.	1	1	1	2	1	1	.	1	.	1	.	.	.	.
Verbena officinalis	62	3	1	1	.	1	1	1	1	1	2	.	.	.	.	.	.	.	.
Agrostis gigantea	61	1	1	1	.	1	1	2	1	1	1	.	1	.	.	.	.	1	.
Euphrasia salisburgensis	61	.	1	.	.	.	.	1	.	.	.	.	1	.	2	1	1	17	14
Polygala chamaebuxus	61	.	.	1	17	2	.	.	.	.	.	.	.	.	1	2	.	.	.
Aster alpinus	60	.	.	.	.	1	.	.	.	1	.	.	1	.	4	.	.	14	5
Bellidiastrum michelii	60	.	.	.	1	1	.	.	.	.	.	.	.	.	1	.	.	23	.
Clematis integrifolia	60	1	.	1	.	.	1	1	1	1	2	1	1	.	.	.	.	.	.
Convolvulus lineatus	60	.	.	.	.	.	.	.	.	.	1	32	.	.	.	.	.	.	.
Oenothera biennis agg.	60	.	1	.	.	.	.	1	1	.	3	.	1	.	.	.	.	.	.
Scleranthus annuus agg.	60	1	1	1	.	1	.	1	3	1	1	.	1	.	.	.	.	.	.
Campanula carpatica	59	.	.	.	.	.	.	.	.	.	.	.	.	.	2	.	.	17	17
Lotus ucrainicus	59	1	.	1	.	.	.	1	1	1	3	.	.	.	.	.	.	.	.
Onosma arenaria	59	.	.	.	.	.	.	.	1	2	1	.	2	.	1	1	1	.	.
Ornithogalum umbellatum	59	4	.	1	.	1	1	1	1	1	1	.	1	.	1	.	1	.	.
Phragmites australis	59	1	.	1	.	1	1	1	1	5	1	1	1	.	.	.	.	.	.
Allium paniculatum	58	.	.	.	.	.	1	.	1	5	1	.	3	.	1	.	.	.	.
Festuca stricta subsp. stricta	58	.	.	.	.	1	.	1	1	1	.	.	3	.	.	6	5	.	.
Galium aparine	58	1	.	1	.	1	1	1	1	1	1	.	1	.	1	.	.	1	.
Linum perenne	58	2	.	1	.	1	1	1	1	1	2	2	1	.	1	.	.	1	.
Ranunculus auricomus agg.	58	1	.	5	.	1	1	.	1	.	1	.	.	.	.	.	.	.	.
Ranunculus breyninus	58	1	.	1	.	1	1	1	1	.	.	.	.	.	1	.	.	16	3

Group number		B1	B2	B3	B4	B5	B6	B7	F1	F2	F3	F4	S1	S2	S3	S4	S5	S6	S7
Twinspan cluster level 4		7	7	8	8	9	9	10	10	10	10	10	10	13	13	14	14	15	16
Twinspan cluster level 5-6		0	1	0	1	0	1		0-0	0-1	1-0	1-1		0	1	0	1	0	1
No. of relevés		301	135	994	235	846	765	760	1324	730	1316	177	709	65	690	174	250	192	66
Tanacetum millefolium	58	.	.	.	.	.	1	1	.	.	1	27	.	.	.	.	.	.	.
Vicia sepium	58	3	1	3	.	1	1	1	1	.	1	.	.	.	.	.	.	1	.
Astrantia major	57	.	.	4	2	1	1	.	.	.	.	.	.	.	.	.	.	4	.
Campanula trachelium	57	1	1	2	.	1	.	1	.	.	.	.	1	.	1	.	.	5	.
Carex alba	57	.	.	1	.	1	1	1	.	.	.	.	.	.	1	.	.	11	.
Nepeta nuda	57	1	.	1	.	1	4	1	1	1	1	1	1	.	.	.	.	.	.
Polypodium vulgare	57	.	.	.	.	.	.	.	1	.	.	.	1	22	2	.	.	2	14
Tanacetum vulgare	57	1	8	1	.	1	1	1	.	.	2	.	.	.	.	.	.	.	.
Astragalus vesicarius	55	.	.	.	.	.	1	1	1	1	1	16	1	.	1	3	1	.	.
Bromus sterilis	55	2	.	1	.	.	1	1	2	1	1	.	1	.	1	.	.	.	.
Crepis praemorsa	55	.	.	3	1	1	2	.	.	1	1	.	.	.	1	.	.	1	.
Herniaria polygama	55	.	.	.	.	.	.	1	1	.	4	2	.	.	.	.	.	.	.
Centaurea atropupurea	54	.	.	.	.	1	1	1	1	2	.	.	2	.	1	.	.	.	17
Melica nutans	54	1	.	1	.	1	1	1	1	.	1	.	.	.	1	.	.	7	.
Nardus stricta	54	1	.	4	2	1	.	1	.	.	1	.	.	.	.	.	.	.	.
Ornithogalum pyramidale	54	3	.	1	.	1	1	1	1	2	1	.	1	.	1	.	.	.	.
Poa pannonica	54	.	.	.	.	.	.	1	3	1	.	.	2	.	.	.	.	.	.
Primula elatior	54	1	1	2	.	1	1	1	.	.	1	.	1	.	1	.	.	3	2
Saxifraga bulbifera	54	2	.	1	.	1	1	1	2	.	1	.	1	.	.	.	.	.	.
Hieracium bifidum	53	.	.	.	.	1	.	.	.	.	.	.	1	3	2	2	.	13	6
Hyacinthella leucophaea	53	.	.	.	.	.	1	.	1	1	2	7	1	.	.	.	.	.	.
Isatis tinctoria	53	.	.	.	.	.	1	1	1	2	1	.	1	2	2	.	.	.	12
Spiraea media	53	.	.	.	.	.	1	1	1	.	.	.	1	.	4	.	.	.	2
Anagallis arvensis	52	1	1	.	.	1	1	1	1	1	1	1	1	.	.	.	.	.	.
Buglossoides arvensis	52	.	.	.	.	1	1	1	2	.	1	1	2	2	.	.	1	.	.
Galium humifusum	52	.	.	.	.	.	.	1	.	2	2	1	1	.	.	.	.	.	.
Inula germanica	52	1	.	1	.	.	2	1	1	2	1	1	1	.	.	.	.	.	.
Orobanche caryophyllacea	52	.	.	.	.	1	1	1	1	2	1	.	1	.	1	1	.	1	2
Cruciata laevipes	51	2	.	2	1	1	1	1	1	.	1	.	1	.	.	.	.	1	.
Plantago major	51	1	2	1	.	1	1	1	1	1	1	.	.	.	1	.	.	.	.
Cirsium erisithales	50	.	.	2	1	1	1	.	.	.	.	.	.	.	1	.	.	11	9
Festuca stricta subsp. trachyphylla	50	.	.	1	.	1	1	1	3	.	1	.	.	.	.	.	.	.	.
Ononis pusilla	50	.	.	.	.	1	1	1	1	1	1	.	3	.	.	1	6	.	.
Echium italicum	49	.	.	.	.	.	1	1	1	2	2	.	.	.	.	.	.	.	.
Lathyrus nissolia	49	3	.	1	.	1	1	1	1	1	1	.	.	.	.	.	.	.	.
Rhamnus saxatilis	49	.	.	.	1	1	1	1	1	1	.	.	1	2	3	1	.	.	14
Thalictrum simplex	49	.	.	1	4	1	1	1	.	1	1	.	1	.	.	.	.	.	.
Bromus arvensis	48	2	.	1	.	.	1	1	1	1	1	.	.	.	1	.	.	.	.
Chenopodium album agg.	48	2	.	.	.	.	1	1	1	1	1	.	1	.	.	.	.	.	.
Dianthus spiculifolius	48	.	.	.	.	.	.	.	.	.	.	.	.	.	3	.	.	.	38
Ferulago sylvatica	48	.	.	1	.	1	3	.	1	.	1	.	1	.	1	.	.	.	.

Group number		B1	B2	B3	B4	B5	B6	B7	F1	F2	F3	F4	S1	S2	S3	S4	S5	S6	S7
Twinspan cluster level 4		7	7	8	8	9	9	10	10	10	10	10	10	13	13	14	14	15	16
Twinspan cluster level 5-6		0	1	0	1	0	1		0-0	0-1	1-0	1-1		0	1	0	1	0	1
No. of relevés		301	135	994	235	846	765	760	1324	730	1316	177	709	65	690	174	250	192	66
Gentianella ciliata	48	.	.	2	3	2	1	.	.	.	.	.	.	.	1	.	.	1	.
Gypsophila fastigiata	48	.	1	1	.	1	1	1	1	1	1	.	1	.	1	2	2	.	.
Papaver dubium	48	.	.	.	.	1	1	1	2	1	1	.	2	.	1	.	.	.	.
Potentilla collina agg.	48	3	.	2	.	1	1	1	1	1	1	.	.	.	.	.	.	.	.
Sesleria sadlerana	48	.	.	.	.	.	.	.	.	.	.	.	1	2	5	3	1	1	.
Sonchus arvensis	48	1	18	.	.	.	1	1	.	1	1	.	.	.	.	.	.	.	.
Daphne cneorum	47	.	.	1	.	.	1	1	1	.	.	.	1	.	4	.	1	1	2
Dianthus borbasii	47	1	1	.	.	.	.	1	1	1	2	.	1	.	.	.	.	.	.
Herniaria incana	47	.	.	.	.	.	.	.	1	.	2	12	.	.	.	.	.	.	.
Ballota nigra	46	1	1	.	.	.	.	1	1	1	2	.	.	.	1	.	.	.	.
Capsella bursa-pastoris	46	1	.	1	.	1	1	1	1	.	1	1	1	.	1	.	.	.	.
Carthamus lanatus	46	.	.	.	.	1	1	1	1	2	1	.	.	.	.	.	.	.	.
Echinops sphaerocephalus	46	.	.	1	.	1	1	1	1	1	1	.	1	.	1	.	.	.	.
Filago arvensis	46	1	.	.	.	.	.	1	3	.	1	1	1	.	.	.	.	.	.
Hesperis tristis	46	.	.	.	.	.	1	1	2	1	1	1	1	.	.	.	.	.	.
Linaria angustissima	45	.	.	.	.	.	1	1	2	1	1	.	1	.	1	1	.	.	.
Podospermum canum	45	1	.	1	.	.	.	1	1	1	2	.	.	.	.	.	.	.	.
Sedum hispanicum	45	.	.	.	.	.	.	1	1	1	1	.	1	3	3	.	.	.	5
Selinum silaifolium	45	.	.	.	.	.	.	.	.	.	.	.	1	.	4	.	.	.	20
Asplenium viride	44	.	.	.	.	.	.	.	.	.	.	.	1	.	1	.	.	14	5
Carex panicea	44	.	.	4	.	1	.	1	.	.	.	.	.	.	1	.	.	.	.
Descurainia sophia	44	.	.	.	.	.	1	1	1	.	1	3	1	.	.	.	.	.	.
Geranium pusillum	44	1	.	1	.	1	1	1	1	.	1	.	1	.	1	.	.	.	.
Herniaria glabra	44	.	1	.	.	1	.	2	1	.	1	.	1	.	.	.	.	.	.
Koeleria glauca	44	.	1	1	.	1	.	1	1	1	1	.	1	.	1	.	.	.	.
Geranium pratense	43	2	2	2	.	1	1	1	.	1	1	.	.	.	.	.	.	.	.
Geum urbanum	43	3	1	1	.	1	1	1	1	.	1	.	1	.	1	.	.	.	.
Astragalus asper	42	.	.	.	.	1	1	1	1	2	1	.	1	.	.	.	.	.	.
Bellis perennis	42	4	.	2	1	.	.	1	1	.	1	.	.	.	.	.	.	.	.
Cystopteris fragilis	42	.	.	.	.	.	.	.	1	.	.	.	1	5	1	.	.	6	14
Lathyrus sylvestris	42	2	.	2	1	1	1	1	.	.	1	.	.	.	.	.	.	.	.
Pilosella caespitosa	41	1	.	1	.	1	1	1	1	1	1	.	.	.	1	.	.	.	.
Pilosella hoppeana	41	.	.	1	1	1	1	1	1	1	.	.	1	.	1	1	1	.	.
Potentilla astracanica	41	.	.	.	.	.	.	.	.	.	1	20	.	.	.	.	.	.	.
Carex stenophylla	40	1	.	.	.	.	.	1	2	.	1	.	.	.	.	.	.	.	.
Festuca arundinacea	40	5	.	1	.	.	1	1	1	.	1	.	.	.	.	.	.	.	.
Lathyrus niger	40	1	.	3	.	1	.	.	1	.	.	.	1	.	.	.	.	.	.
Lysimachia nummularia	40	2	1	3	.	1	.	1	.	.	1	.	.	.	.	.	.	.	.
Orobanche lutea	40	.	1	1	.	2	1	1	1	1	.	.	1	.	.	.	.	.	.
Thalictrum foetidum	40	.	.	.	.	.	.	.	.	1	1	.	1	.	4	.	.	.	9
Campanula rapunculus	39	2	.	.	1	1	1	1	1	.	1	.	1	.	1	.	1	.	.

Group number		B1	B2	B3	B4	B5	B6	B7	F1	F2	F3	F4	S1	S2	S3	S4	S5	S6	S7
Twinspan cluster level 4		7	7	8	8	9	9	10	10	10	10	10	10	13	13	14	14	15	16
Twinspan cluster level 5-6		0	1	0	1	0	1		0-0	0-1	1-0	1-1		0	1	0	1	0	1
No. of relevés		301	135	994	235	846	765	760	1324	730	1316	177	709	65	690	174	250	192	66
Genista germanica	39	.	.	1	5	1	1	1	1	.	.	.	1	2	1	.	.	.	.
Seseli longifolium	39	.	.	.	.	1	.	.	1	1	.	.	1	.	3	.	.	4	.
Silene nemoralis	39	1	.	1	.	1	1	.	1	.	.	.	1	.	1	.	.	3	.
Stipa dasyphylla	39	.	.	.	.	.	1	1	1	1	1	1	1	.	.	1	.	.	.
Trisetum alpestre	39	.	.	.	.	.	.	.	1	.	.	.	.	.	1	.	.	17	3
Arenaria procera	38	.	.	.	.	.	1	1	1	1	1	3	1	.	.	.	.	.	.
Asarum europaeum	38	.	.	2	.	1	1	1	.	.	.	.	.	.	1	.	.	2	5
Euphorbia angulata	38	.	.	1	.	1	2	1	.	1	1	.	1	.	1	.	.	.	.
Marrubium pestalozzae	38	.	.	.	.	.	.	1	.	2	1	1	1	.	.	.	.	.	.
Neottia ovata	38	.	.	3	2	.	.	.	.	.	.	.	.	.	.	.	.	1	.
Nepeta ucranica	38	.	.	.	.	.	.	.	.	2	1	6	1	.	.	.	.	.	.
Rhinanthus alectorolophus	38	1	.	2	3	1	.	1	1	.	.	.	.	.	.	.	.	4	.
Poa versicolor	37	.	.	.	.	.	1	1	1	1	1	.	1	.	.	.	.	.	.
Salvia aethiopis	37	1	.	.	.	.	.	1	1	1	2	5	.	.	.	.	.	.	.
Carduus candicans	36	.	.	.	.	.	.	1	1	1	.	.	1	3	2	.	.	.	2
Linum linearifolium	36	.	.	.	.	.	.	.	.	.	.	20	.	.	.	.	.	.	.
Noccaea praecox	36	.	.	1	13	1	.	.	.	.	.	.	.	.	.	.	.	.	.
Pimpinella tragium	36	.	.	.	.	.	.	.	.	.	1	18	1	.	.	.	.	.	.
Potentilla thuringiaca	36	.	.	1	.	1	1	.	1	.	.	.	.	.	1	.	.	1	14
Pulmonaria angustifolia	36	1	.	3	.	1	1	1	.	.	1	1	.	.	.	.	.	.	.
Symphytum tuberosum	36	1	.	3	.	.	1	.	.	.	.	.	.	.	1	.	.	1	.
Tussilago farfara	36	.	4	1	.	1	1	1	.	1	1	.	.	.	.	.	.	2	.
Alyssum rostratum	35	.	.	.	.	.	.	.	1	2	1	1	1	.	.	.	.	.	.
Carduus crispus	35	1	1	.	.	1	.	1	1	1	2	.	.	.	.	.	.	.	.
Festuca arvernensis	35	.	.	.	.	.	.	.	1	.	.	.	1	.	2	.	.	.	17
Gypsophila muralis	35	1	.	.	.	1	1	1	1	1	1	1	.	.	.	.	.	.	.
Selaginella helvetica	35	.	.	.	1	1	.	1	1	.	.	.	.	.	1	.	.	1	21
Trifolium striatum	35	2	.	.	.	.	.	1	1	.	1	.	.	.	.	.	.	.	.
Alyssum murale	34	.	.	.	.	.	.	.	2	1	.	1	1	2	1	.	.	.	2
Avenella flexuosa	34	.	.	1	.	.	1	1	1	.	1	.	2	2	1	.	.	.	.
Festuca dalmatica	34	.	.	.	.	.	.	.	2	1	.	.	1	.	1	.	.	.	2
Minuartia hirsuta	34	.	.	.	.	.	.	1	2	.	.	.	1	2	.	.	.	.	.
Polycnemum arvense	34	.	.	.	.	.	.	.	1	1	1	.	1	.	.	.	.	.	.
Ranunculus zapalowiczii	34	.	.	.	.	1	2	1	.	.	1	.	1	.	.	.	.	.	.
Silene viscosa	34	1	2	.	.	.	.	.	1	1	1	1	.	.	1	.	.	.	.
Anacamptis coriophora	33	1	.	1	1	1	1	1	1	.	1	.	.	.	1	.	.	.	.
Anthemis ruthenica	33	.	.	.	.	1	1	1	1	.	2	1	.	.	.	.	.	.	.
Astragalus exscapus	33	.	.	.	.	.	1	1	.	3	1	.	1	.	.	.	.	.	.
Crepis setosa	33	1	.	.	.	1	1	1	1	.	1	.	.	.	.	.	.	.	.
Echinops ritro	33	.	.	.	.	.	1	.	.	1	1	.	1	.	2	1	1	.	.
Iris humilis	33	.	.	.	.	.	1	.	1	1	.	.	1	.	.	1	4	.	.

Group number		B1	B2	B3	B4	B5	B6	B7	F1	F2	F3	F4	S1	S2	S3	S4	S5	S6	S7
Twinspan cluster level 4		7	7	8	8	9	9	10	10	10	10	10	10	13	13	14	14	15	16
Twinspan cluster level 5-6		0	1	0	1	0	1		0-0	0-1	1-0	1-1		0	1	0	1	0	1
No. of relevés		301	135	994	235	846	765	760	1324	730	1316	177	709	65	690	174	250	192	66
Jurinea stoechadifolia	33	.	.	.	.	.	.	.	.	.	.	19	.	.	.	.	.	.	.
Lamium amplexicaule	33	.	.	.	.	.	.	1	2	1	1	.	1	.	.	.	1	.	.
Mercurialis perennis	33	.	.	1	.	1	.	.	.	.	.	.	1	.	1	.	.	10	.
Pedicularis comosa	33	.	.	1	.	.	1	1	.	.	.	.	.	.	1	.	.	.	12
Teucrium botrys	33	.	.	.	.	1	.	1	1	.	.	.	1	.	1	1	.	1	.
Xeranthemum inapertum	33	3	.	.	.	.	.	1	1	.	1	.	.	.	.	.	.	.	.
Asperula montana	32	.	.	.	.	.	.	.	.	1	1	12	.	.	.	.	.	.	.
Campanula cochleariifolia	32	.	.	.	.	.	.	.	.	.	.	.	.	.	1	.	.	16	.
Centaurea oxylepis	32	1	.	2	.	1	1	.	.	.	.	.	1	.	.	.	.	.	.
Ephedra distachya	32	.	.	.	.	.	.	.	1	2	1	7	.	.	.	.	.	.	.
Erica carnea	32	.	.	.	8	1	.	.	.	.	.	.	.	.	1	.	.	1	.
Gymnocarpium robertianum	32	.	.	.	.	.	.	.	.	.	.	.	1	.	1	.	.	10	3
Limonium gmelinii	32	.	.	.	.	.	.	1	1	1	2	.	.	.	.	.	.	.	.
Psephellus trinervius	32	.	.	.	.	.	.	1	.	3	.	2	1	.	.	.	.	.	.
Rhinanthus borbasii	32	.	.	.	.	.	.	1	1	1	1	.	.	.	.	.	.	.	.
Veratrum nigrum	32	.	.	1	.	1	1	1	.	1	1	1	.	.	1	1	.	.	.
Viola suavis	32	.	.	1	.	1	1	.	1	2	.	.	2	.	.	.	.	.	.
Ambrosia artemisiifolia	31	1	1	.	.	.	.	1	1	1	1	1	.	.	.	.	.	.	.
Carex distans	31	2	.	1	.	1	1	1	1	.	1	.	.	.	.	.	.	.	.
Coronilla coronata	31	.	.	.	.	1	1	1	.	1	.	.	.	.	2	.	.	4	.
Euphorbia stricta	31	1	.	1	.	1	.	1	1	.	.	.	1	.	.	.	.	2	.
Laserpitium siler	31	.	.	.	3	1	.	.	.	.	.	.	.	.	2	.	.	.	.
Lithospermum officinale	31	1	.	1	.	1	1	1	1	1	1	.	.	.	.	.	.	.	.
Polygonum aviculare agg.	31	1	.	.	1	.	1	1	1	.	1	.	.	.	.	.	.	.	.
Rubus saxatilis	31	.	.	.	.	1	1	.	.	.	.	.	.	.	1	.	.	10	.
Seseli rigidum	31	.	.	.	.	.	.	.	.	.	.	.	1	.	2	.	.	.	18
Thymelaea passerina	31	.	.	.	.	1	.	1	1	2	1	3	1	.	.	.	1	.	.
Traunsteinera globosa	31	.	.	3	1	1	.	.	.	.	.	.	.	.	.	.	.	1	.
Allium waldsteinii	30	.	.	.	.	.	.	1	1	1	2	.	.	.	.	.	.	.	.
Koeleria delavignei	30	1	.	.	.	.	.	1	.	.	2	.	.	.	.	.	.	.	.
Mercurialis ovata	30	.	.	1	.	1	2	.	.	.	.	.	.	.	1	.	.	.	.
Psephellus sumensis	30	.	.	.	.	.	.	1	1	.	1	3	1	.	.	.	.	.	.
Succisa pratensis	30	1	.	2	3	1	.	.	1	.	1	.	.	.	.	.	.	.	.
Asparagus tenuifolius	29	.	.	.	.	.	.	.	1	1	2	1	1	.	.	.	.	.	.
Festuca wagneri	29	.	.	.	.	.	.	.	1	2	.	.	1	.	.	.	.	.	.
Filago germanica	29	.	.	1	.	1	1	.	1	1	1	.	1	.	1	.	.	.	.
Helleborus purpurascens	29	.	.	1	.	1	3	1	1	.	.	.	.	.	.	.	.	.	.
Trifolium pannonicum	29	.	.	2	.	.	1	1	.	1	1	.	.	.	.	.	.	.	.
Calamagrostis arundinacea	28	.	.	2	.	1	1	1	1	.	1	.	.	2	.	.	.	1	2
Deschampsia cespitosa	28	1	5	1	.	.	1	.	.	.	1	.	1	.	.	.	.	.	.
Gentiana clusii	28	.	.	.	1	.	.	.	.	.	.	.	.	.	1	.	.	13	.

Group number		B1	B2	B3	B4	B5	B6	B7	F1	F2	F3	F4	S1	S2	S3	S4	S5	S6	S7
Twinspan cluster level 4		7	7	8	8	9	9	10	10	10	10	10	10	13	13	14	14	15	16
Twinspan cluster level 5-6		0	1	0	1	0	1		0-0	0-1	1-0	1-1		0	1	0	1	0	1
No. of relevés		301	135	994	235	846	765	760	1324	730	1316	177	709	65	690	174	250	192	66
Gypsophila thyraica	28	.	.	.	.	.	1	.	1	2	1	.	1	2	.	.	.	.	.
Helianthemum oelandicum	28	.	.	.	.	.	.	.	.	.	.	.	.	.	1	.	.	11	2
Knautia illyrica	28	1	.	1	7	1	.	.	.	.	.	.	.	.	.	.	.	.	.
Lactuca muralis	28	.	1	1	.	.	.	1	1	.	.	.	1	.	1	.	.	5	6
Ophrys apifera	28	.	.	1	5	1	1	.	.	.	.	.	.	.	1	.	.	.	.
Phyteuma spicatum	28	.	.	1	.	1	1	.	.	.	1	.	1	.	1	.	.	2	3
Pilosella pavichii	28	.	.	.	.	.	.	.	1	.	.	.	1	.	2	.	.	.	8
Silene italica	28	.	.	1	.	.	1	1	1	1	.	.	1	2	2	.	.	.	.
Spiraea hypericifolia	28	.	.	.	.	.	.	1	1	.	1	1	.	.	.	.	.	.	.
Botrychium lunaria	27	.	.	1	1	1	.	1	1	1	.	.	1	.	1	.	1	2	.
Gagea bohemica	27	.	.	.	.	.	.	.	1	.	.	.	2	.	1	1	.	.	.
Gentianella germanica	27	.	.	1	7	1	.	.	.	.	.	.	.	.	.	.	.	.	.
Gentianella lutescens	27	.	.	1	.	1	.	.	.	.	.	.	.	.	1	.	.	6	.
Hierochloe odorata	27	.	.	.	.	.	.	1	.	.	2	.	.	.	.	.	.	.	.
Ophrys insectifera	27	.	.	1	2	1	.	.	.	.	.	.	.	.	2	.	.	.	.
Peucedanum arenarium	27	.	.	.	.	.	.	1	1	1	1	.	1	.	.	1	.	.	.
Scabiosa cinerea subsp. hladnikiana	27	.	.	.	4	2	.	.	.	.	.	.	.	.	.	.	.	.	.
Setaria pumila	27	.	1	.	.	.	.	1	1	1	1	.	1	.	1	.	.	.	.
Torilis arvensis	27	1	.	.	.	.	1	1	1	1	1	.	1	.	.	.	.	.	.
Urtica dioica	27	2	1	1	.	1	1	1	1	.	1	.	.	.	1	.	.	1	.
Aegopodium podagraria	26	1	.	2	.	1	.	.	.	.	.	.	.	.	.	.	.	2	.
Alyssum tortuosum	26	.	.	.	.	.	.	1	1	1	.	1	1	.	1	3	2	.	.
Galium schultesii	26	.	.	1	.	1	1	1	1	.	1	.	1	.	1	.	.	3	3
Moenchia mantica	26	4	.	1	.	1	.	1	1	.	1	.	.	.	.	.	.	.	.
Pteridium aquilinum	26	1	.	1	.	1	1	1	.	.	.	.	.	.	1	.	.	1	.
Thalictrum aquilegiifolium	26	.	.	1	.	.	2	1	.	1	1	.	.	.	1	.	.	2	.
Tofieldia calyculata	26	.	.	1	2	1	.	.	.	.	.	.	.	.	1	.	.	7	.
Arabis turrita	25	.	.	.	.	1	.	1	1	.	.	.	1	.	2	.	.	.	3
Crepis tectorum	25	1	.	.	.	1	1	1	1	.	1	1	.	.	.	.	.	.	.
Dianthus serotinus	25	.	.	.	.	.	.	.	1	1	.	.	.	.	1	6	1	.	.
Erysimum hieraciifolium agg.	25	.	.	.	.	1	1	1	1	1	1	1	1	.	.	1	.	.	.
Euphorbia amygdaloides	25	.	.	1	.	1	.	1	.	.	1	.	1	.	1	.	.	5	.
Linum viscosum	25	.	.	.	7	1	.	.	.	1	.	.	.	.	.	.	.	.	.
Melampyrum sylvaticum	25	.	.	1	.	1	.	.	.	.	.	.	.	.	.	.	.	8	.
Pedicularis kaufmannii	25	.	.	.	.	1	1	1	.	.	1	.	1	.	.	.	.	.	.
Tripleurospermum inodorum	25	1	.	.	.	.	1	1	1	.	1	.	.	.	.	.	.	.	.
Valeriana tripteris	25	.	1	.	.	1	.	.	.	.	.	.	1	.	1	.	.	9	.
Verbascum marschallianum	25	.	.	.	.	.	.	.	.	.	1	7	.	.	.	.	.	.	.
Vicia grandiflora	25	1	.	.	.	.	.	1	1	.	1	.	.	.	.	.	.	.	.
Artemisia scoparia	24	.	.	.	.	.	.	.	1	1	1	.	1	.	.	.	.	.	.
Cephalaria transsylvanica	24	.	.	.	.	.	.	.	1	1	1	.	.	.	.	.	.	.	.

Group number		B1	B2	B3	B4	B5	B6	B7	F1	F2	F3	F4	S1	S2	S3	S4	S5	S6	S7
Twinspan cluster level 4		7	7	8	8	9	9	10	10	10	10	10	10	13	13	14	14	15	16
Twinspan cluster level 5-6		0	1	0	1	0	1		0-0	0-1	1-0	1-1		0	1	0	1	0	1
No. of relevés		301	135	994	235	846	765	760	1324	730	1316	177	709	65	690	174	250	192	66
Goniolimon tataricum	24	.	.	.	.	.	.	.	.	1	1	2	.	.	.	.	.	.	.
Helleborus odorus	24	1	.	1	1	1	1	1	.	.	.	.	1	.	.	.	.	.	.
Luzula luzuloides	24	.	.	2	.	1	.	1	.	.	.	.	1	2	1	.	.	1	2
Orobanche teucrii	24	.	.	.	.	1	1	1	1	1	.	.	1	.	.	.	.	.	.
Plantago holosteum	24	.	.	.	9	1	.	.	.	.	.	.	.	.	.	.	.	.	.
Saponaria bellidifolia	24	.	.	.	.	.	.	.	.	.	.	.	1	.	3	.	.	.	2
Torilis japonica	24	2	1	1	.	1	1	1	1	.	1	.	1	.	.	.	.	1	.
Veronica fruticans	24	.	1	.	.	.	.	.	.	.	.	.	1	.	1	.	.	10	.
Asperula capitata	23	.	.	.	.	.	.	.	.	.	.	.	.	.	1	.	.	.	21
Astragalus australis	23	.	.	.	.	.	.	.	1	3	1	.	.	.	.	.	.	.	.
Carlina acanthifolia	23	.	.	.	.	1	1	1	.	1	1	.	.	.	1	.	.	.	.
Cynoglottis barrelieri	23	1	.	1	.	.	1	.	.	2	.	.	1	.	1	.	.	.	.
Dianthus monspessulanus	23	.	.	.	8	1	.	.	.	.	.	.	.	.	1	.	.	.	.
Dianthus pseudarmeria	23	.	.	.	.	.	.	1	.	.	1	8	.	.	.	.	.	.	.
Fallopia dumetorum	23	.	.	.	.	1	.	1	1	1	.	.	1	.	1	.	.	.	.
Myosotis discolor	23	2	.	1	.	.	.	1	1	.	1	.	.	.	.	.	.	.	.
Sambucus ebulus	23	1	.	.	.	.	1	1	1	.	1	.	.	.	.	.	.	.	.
Taraxacum sect. Erythrocarpa	23	.	.	.	.	.	.	.	1	1	.	.	1	.	1	.	.	.	14
Thymus alpestris	23	.	.	1	.	1	.	.	.	.	1	.	.	.	1	.	.	5	.
Tragopogon tommasinii	23	1	.	1	7	1	.	.	.	.	.	.	.	.	.	.	.	.	.
Alyssum desertorum	22	.	.	.	.	.	.	1	1	1	1	4	.	.	.	.	.	.	.
Anemone nemorosa	22	.	.	1	1	1	1	.	.	.	1	.	.	.	1	.	.	.	.
Centaurea macroptilon	22	1	1	1	1	1	1	.	.	.	.	.	.	.	.	.	.	.	.
Crupina vulgaris	22	.	.	.	.	.	1	.	1	1	.	.	1	.	.	.	1	.	.
Cyanus pinnatifidus	22	.	.	.	.	.	.	.	.	.	.	.	.	.	2	.	.	.	8
Echium russicum	22	.	.	.	.	.	1	.	.	1	1	.	1	.	.	.	.	.	.
Equisetum ramosissimum	22	.	.	1	.	.	.	1	1	.	1	.	.	.	.	.	.	.	.
Linum nervosum	22	.	.	1	.	.	1	1	.	1	.	.	1	.	.	.	.	.	.
Melampyrum pratense	22	1	.	1	.	1	1	1	.	1	.	.	1	.	1	.	.	1	.
Myosotis sylvatica agg.	22	1	.	1	.	.	1	.	.	.	.	.	1	.	1	.	.	1	.
Sesleria uliginosa	22	.	.	2	.	1	.	.	.	.	.	.	.	.	.	.	.	.	.
Solidago canadensis	22	2	1	1	.	1	.	1	1	.	1	.	.	.	.	.	.	.	.
Allium flavescens	21	.	.	.	.	.	.	.	1	1	1	5	1	2	.	.	.	.	.
Allium moschatum	21	.	.	.	.	.	.	.	.	.	.	.	.	.	.	4	6	.	.
Aquilegia nigricans	21	.	.	1	6	1	.	.	.	.	.	.	.	.	1	.	.	.	2
Astragalus ucrainicus	21	.	.	.	.	.	.	.	.	.	.	12	.	.	.	.	.	.	.
Carex ericetorum	21	.	.	1	.	1	.	1	1	.	.	.	.	.	.	1	.	3	.
Cirsium canum	21	1	.	1	.	.	1	1	.	.	1	.	.	.	.	.	.	1	.
Cuscuta europaea	21	1	.	1	.	.	1	.	1	1	1	.	1	.	.	.	.	.	.
Knautia dipsacifolia	21	1	.	1	1	1	.	.	.	.	.	.	1	.	.	.	.	2	.
Lychnis flos-cuculi	21	3	.	1	.	.	.	.	.	.	1	.	.	.	.	.	.	.	.

Group number		B1	B2	B3	B4	B5	B6	B7	F1	F2	F3	F4	S1	S2	S3	S4	S5	S6	S7
Twinspan cluster level 4		7	7	8	8	9	9	10	10	10	10	10	10	13	13	14	14	15	16
Twinspan cluster level 5-6		0	1	0	1	0	1		0-0	0-1	1-0	1-1		0	1	0	1	0	1
No. of relevés		301	135	994	235	846	765	760	1324	730	1316	177	709	65	690	174	250	192	66
Ornithogalum collinum	21	1	.	.	.	.	1	1	1	1	1	.	1	.	.	.	.	.	.
Rumex crispus	21	2	.	1	.	.	1	.	1	1	1	.	.	.	.	.	.	.	.
Senecio doria	21	1	.	1	.	1	1	1	.	.	1	.	.	.	.	.	.	.	.
Aconitum variegatum	20	.	.	1	.	.	.	.	.	.	.	.	.	.	.	.	.	9	.
Allium paczoskianum	20	.	.	.	.	.	.	.	1	.	1	3	1	.	.	.	.	.	.
Amygdalus nana	20	.	.	.	.	.	.	.	1	.	1	4	.	.	.	.	.	.	.
Carex colchica	20	.	.	.	.	.	.	1	.	.	1	.	.	.	.	.	.	.	.
Carex sempervirens	20	.	.	1	.	1	.	.	.	.	.	.	.	.	.	.	.	9	.
Chaerophyllum hirsutum	20	1	.	1	1	.	.	.	.	.	.	.	.	.	1	.	.	1	.
Cirsium furiens	20	1	.	.	.	1	1	1	.	1	1	.	.	.	1	.	.	.	.
Crepis jacquinii	20	.	.	.	.	.	.	.	.	.	.	.	.	.	.	.	.	10	.
Dianthus petraeus	20	.	.	.	.	.	.	.	.	.	.	.	1	.	1	.	.	.	18
Galeopsis angustifolia	20	1	1	.	.	1	.	1	1	.	.	.	1	.	1	.	.	.	.
Lamium purpureum	20	1	.	1	.	1	.	1	1	.	1	.	1	.	.	.	.	.	.
Papaver rhoeas	20	.	1	.	.	1	.	1	1	1	1	1	1	.	.	.	.	.	.
Salvia dumetorum	20	.	.	.	.	.	.	1	.	.	1	3	.	.	.	.	.	.	.
Sempervivum montanum	20	.	.	.	.	.	.	.	1	.	.	.	1	3	1	.	.	.	.
Senecio squalidus	20	.	.	.	.	.	1	.	1	.	.	.	1	.	1	1	.	.	5
Silene tatarica	20	.	.	.	.	.	.	1	.	.	1	.	.	.	.	.	.	.	.
Stipa borysthenica	20	.	.	.	.	1	.	1	1	.	1	.	.	.	.	.	.	.	.
Thesium procumbens	20	.	.	.	.	.	.	1	.	1	1	.	1	.	.	.	.	.	.
Veronica serpyllifolia	20	2	.	1	.	.	.	1	1	.	1	.	1	.	.	.	.	.	.
Veronica triphyllos	20	.	.	.	.	.	1	1	1	1	1	.	1	.	1	.	.	.	.
Vicia pannonica	20	1	.	.	.	.	.	1	1	1	1	.	.	.	.	.	.	.	.
Aegonychon purpurocaeruleum	19	.	.	.	.	1	1	1	1	1	.	.	1	.	1	.	.	.	.
Androsace elongata	19	.	.	.	.	.	.	1	1	.	1	1	1	.	.	.	.	.	.
Anthriscus sylvestris	19	1	1	1	.	.	.	.	.	.	1	.	1	.	.	.	.	.	.
Arabidopsis petraea	19	.	.	.	.	.	.	.	.	.	.	.	.	3	1	5	.	.	.
Astragalus sulcatus	19	.	.	.	.	.	.	1	.	1	1	1	.	.	.	.	.	.	.
Cerastium glomeratum	19	2	.	1	.	.	.	1	1	.	1	.	.	.	.	.	.	.	.
Corynephorus canescens	19	.	.	.	.	.	.	1	1	.	1	.	1	.	.	.	.	.	.
Cyanus mollis	19	.	.	1	.	.	.	.	.	.	.	.	.	.	1	.	.	4	.
Draba aizoides	19	.	.	.	.	.	.	.	.	.	.	.	1	.	1	.	.	5	.
Galeopsis ladanum	19	.	6	.	.	.	.	1	1	.	1	.	1	.	1	.	.	1	.
Genista scythica	19	.	.	.	.	.	.	.	.	.	.	11	.	.	.	.	.	.	.
Hypericum montanum	19	.	.	1	.	.	1	1	1	.	1	.	1	.	1	.	.	.	.
Moehringia muscosa	19	.	.	.	.	.	.	.	.	.	.	.	.	.	1	.	.	1	15
Noccaea jankae	19	.	.	1	.	.	1	1	1	1	1	.	.	.	.	.	.	.	.
Sesleria tenuifolia subsp. kalnikensis	19	.	.	.	.	1	.	.	.	.	.	.	1	.	2	.	.	1	.
Stellaria media	19	1	.	1	.	.	.	1	1	.	1	.	.	.	.	.	.	.	.
Viola mirabilis	19	1	.	1	.	1	1	1	.	.	.	.	.	.	1	.	.	1	.

Group number		B1	B2	B3	B4	B5	B6	B7	F1	F2	F3	F4	S1	S2	S3	S4	S5	S6	S7
Twinspan cluster level 4		7	7	8	8	9	9	10	10	10	10	10	10	13	13	14	14	15	16
Twinspan cluster level 5-6		0	1	0	1	0	1		0-0	0-1	1-0	1-1		0	1	0	1	0	1
No. of relevés		301	135	994	235	846	765	760	1324	730	1316	177	709	65	690	174	250	192	66
Achillea ochroleuca	18	.	.	.	.	.	.	1	1	.	1	1	.	.	.	.	.	.	.
Allium albidum subsp. albidum	18	.	.	.	.	.	.	1	1	1	1	.	1	.	.	.	.	.	.
Astragalus corniculatus	18	.	.	.	.	.	.	1	.	.	1	8	.	.	.	.	.	.	.
Centaurea nigrescens	18	.	.	1	3	1	1	.	.	.	.	.	.	.	.	.	.	.	.
Epilobium dodonaei	18	.	1	.	.	1	.	1	1	.	1	.	1	.	1	.	.	.	.
Erucastrum nasturtiifolium	18	.	.	.	.	1	.	1	1	1	.	.	1	.	.	.	1	.	.
Erysimum comatum	18	.	.	.	.	.	.	.	1	1	.	.	1	.	1	.	.	.	6
Erysimum crepidifolium	18	.	.	.	.	.	.	.	1	.	.	.	.	.	.	.	.	.	.
Euphorbia platyphyllos	18	1	.	1	.	1	1	.	.	.	1	.	.	.	.	.	.	.	.
Hieracium lachenalii	18	.	.	1	.	1	1	.	.	.	.	.	1	.	1	.	.	1	.
Klasea lycopifolia	18	.	.	1	.	.	1	1	.	.	1	.	1	.	.	.	.	.	.
Lotus tenuis	18	1	.	1	.	.	.	.	1	.	1	.	.	.	.	.	.	.	.
Melittis melissophyllum	18	.	.	1	.	1	1	.	.	.	.	.	.	.	1	.	.	2	.
Muscari botryoides	18	.	.	.	.	1	1	1	1	1	1	.	1	.	.	.	.	.	.
Onopordum acanthium	18	.	.	.	.	1	1	1	1	1	1	2	1	.	.	.	.	.	.
Orobanche elatior	18	.	1	.	.	1	2	.	1	.	.	.	1	.	.	.	.	.	.
Plantago maritima	18	.	.	1	.	.	.	1	1	.	1	.	1	.	.	.	.	.	.
Poa trivialis	18	1	.	1	.	.	.	1	.	1	1	.	1	.	.	.	.	.	.
Scirpoides holoschoenus	18	.	.	1	.	.	.	1	1	.	1	.	.	.	.	.	.	.	.
Trinia kitaibelii	18	1	.	.	.	.	.	1	.	1	1	.	1	.	.	.	.	.	.
Viola reichenbachiana	18	.	.	1	.	.	1	.	.	.	.	.	.	.	1	.	.	.	.
Aristolochia clematitis	17	1	.	1	.	.	.	1	.	.	1	.	.	.	.	.	.	.	2
Barbarea vulgaris	17	.	.	1	.	.	.	1	1	1	1	.	1	.	1	.	.	.	.
Carex hallerana	17	.	.	1	.	1	1	1	.	.	.	.	1	.	.	.	.	.	.
Euphorbia agraria	17	.	.	.	.	.	.	.	.	1	1	3	.	.	.	.	.	.	.
Jurinea multiflora	17	.	.	.	.	.	.	.	.	.	1	8	.	.	.	.	.	.	.
Lathyrus vernus	17	.	.	1	.	.	.	1	.	.	.	.	.	.	.	.	.	3	2
Lilium bulbiferum	17	.	.	1	3	1	.	.	.	.	.	.	.	.	.	.	.	.	.
Orchis purpurea	17	.	.	1	.	1	1	.	.	.	.	.	.	.	.	.	.	.	.
Paeonia tenuifolia	17	.	.	1	.	.	1	.	1	1	1	.	1	.	.	.	.	.	.
Pilosella lactucella	17	.	.	1	.	1	1	1	.	.	.	.	1	.	1	.	.	.	.
Podospermum laciniatum	17	.	.	1	.	.	.	.	1	1	1	.	.	.	.	.	.	.	.
Sempervivum ruthenicum	17	.	.	.	.	.	.	.	1	1	1	.	.	.	1	.	.	.	.
Sisymbrium orientale	17	.	.	.	.	.	.	1	1	.	1	.	.	.	.	.	.	.	.
Thalictrum lucidum	17	1	.	1	.	.	1	1	.	.	1	.	1	.	.	.	.	.	.
Thlaspi arvense	17	.	.	1	.	.	1	1	1	1	1	.	1	.	1	.	.	.	.
Valerianella carinata	17	.	.	.	.	1	.	1	1	1	1	.	1	.	.	.	.	.	.
Verbascum speciosum	17	.	.	.	.	.	.	.	1	1	1	.	1	.	.	.	.	.	.
Apera spica-venti	16	.	.	1	.	1	.	1	1	.	1	.	1	.	.	.	.	.	.
Aurinia petraea	16	.	.	.	.	.	.	.	1	1	.	.	1	.	1	.	.	.	6
Campanula cervicaria	16	.	.	1	.	1	1	.	1	.	.	.	.	.	.	.	.	.	.

Group number		B1	B2	B3	B4	B5	B6	B7	F1	F2	F3	F4	S1	S2	S3	S4	S5	S6	S7
Twinspan cluster level 4		7	7	8	8	9	9	10	10	10	10	10	10	13	13	14	14	15	16
Twinspan cluster level 5-6		0	1	0	1	0	1		0-0	0-1	1-0	1-1		0	1	0	1	0	1
No. of relevés		301	135	994	235	846	765	760	1324	730	1316	177	709	65	690	174	250	192	66
Cardamine pratensis agg.	16	1	.	1	.	.	.	.	.	.	1	.	.	.	.	.	.	.	.
Dianthus fischeri	16	.	3	.	.	.	.	.	1	.	1	.	.	.	.	.	.	.	.
Epilobium angustifolium	16	1	.	1	.	1	.	1	.	.	1	.	.	.	1	.	.	.	.
Epilobium montanum	16	1	1	.	.	.	.	1	.	.	1	.	1	.	.	.	.	4	.
Eupatorium cannabinum	16	1	2	1	.	1	.	.	.	.	1	.	.	.	1	.	.	1	.
Ferulago campestris	16	1	.	1	.	.	.	.	1	.	1	.	.	.	.	.	.	.	.
Gagea minima	16	.	.	.	.	.	.	.	1	1	.	.	1	.	.	1	.	.	.
Gentiana utriculosa	16	.	.	.	3	1	.	.	.	.	.	.	.	.	.	.	.	.	.
Hypericum hirsutum	16	1	.	1	.	1	.	.	.	.	.	.	.	.	.	.	.	1	.
Linaria x kocianovichii	16	.	.	.	.	.	1	.	1	1	1	.	1	.	.	.	.	.	.
Linum ucranicum	16	.	.	.	.	.	.	.	1	1	1	1	1	.	.	.	.	.	.
Orobanche purpurea	16	.	.	.	.	1	1	.	1	1	1	.	.	.	1	.	.	1	.
Pilosella auriculoides	16	.	.	1	.	1	1	1	1	1	1	.	.	.	1	.	.	1	.
Pulsatilla zimmermannii	16	.	.	.	.	.	2	.	1	.	.	.	1	.	.	.	.	.	.
Ranunculus repens	16	1	1	1	.	.	.	1	.	.	1	.	.	.	.	.	.	.	.
Saxifraga granulata	16	1	.	1	.	.	.	1	1	.	.	.	1	.	.	.	.	.	.
Spiraea crenata	16	.	.	.	.	.	.	.	.	.	1	.	1	.	.	.	.	.	5
Stellaria holostea	16	.	.	1	.	1	1	1	1	.	.	.	1	.	.	.	.	1	.
Sternbergia colchiciflora	16	.	.	.	.	.	.	.	.	1	1	.	.	.	.	.	.	.	.
Trifolium hybridum	16	2	.	1	.	.	.	1	1	.	1	.	.	.	.	.	.	.	.
Veronica hederifolia agg.	16	1	.	1	.	1	.	1	1	.	.	.	1	.	.	.	.	.	.
Agrostis canina	15	1	.	1	.	.	.	1	1	1	1	.	.	.	.	.	.	.	.
Bupleurum affine	15	.	.	.	.	.	.	1	1	.	1	.	.	.	.	.	.	.	.
Carex pilulifera	15	1	.	1	.	1	.	.	.	.	.	.	.	.	.	.	.	.	.
Chelidonium majus	15	.	.	.	.	.	.	1	1	.	1	.	1	.	1	.	.	1	.
Cyanus montanus	15	.	.	.	.	1	.	.	.	.	.	.	1	.	1	.	.	4	.
Dipsacus laciniatus	15	2	.	.	.	1	.	1	.	.	1	.	.	.	.	.	.	.	.
Erysimum cheiranthoides	15	.	.	1	.	1	.	.	.	.	1	1	.	.	1	.	.	.	.
Euphorbia helioscopia	15	.	1	.	.	.	1	1	1	1	1	.	1	.	.	.	.	.	.
Euphorbia villosa	15	.	.	1	.	1	1	1	1	1	1	.	.	.	.	.	.	.	.
Geranium rotundifolium	15	.	.	.	.	.	.	1	1	.	.	.	1	.	1	.	.	.	.
Hepatica nobilis	15	.	.	1	.	.	1	.	.	.	.	.	.	.	1	.	.	1	8
Hieracium villosum	15	.	.	.	.	.	.	1	.	.	1	.	.	.	.	.	.	6	.
Leontodon biscutellifolius	15	1	.	.	.	.	1	1	.	1	1	2	1	.	.	.	.	.	.
Noccaea caerulescens	15	.	.	1	.	.	.	.	1	.	.	.	.	.	.	.	.	.	.
Noccaea montana	15	.	.	.	.	.	.	.	.	.	.	.	1	.	1	1	.	.	.
Peucedanum officinale	15	1	.	1	.	.	1	1	.	.	1	.	.	.	.	.	.	.	.
Ranunculus pedatus	15	1	.	.	.	.	.	1	1	.	1	.	.	.	.	.	.	.	.
Achillea coarctata	14	.	.	.	.	.	.	.	1	1	.	.	1	2	.	.	.	.	.
Alliaria petiolata	14	.	.	.	.	1	1	1	1	.	.	.	1	.	1	.	.	.	2
Asperula purpurea	14	.	.	.	.	1	.	.	1	1	.	.	1	2	1	.	.	.	.

Group number		B1	B2	B3	B4	B5	B6	B7	F1	F2	F3	F4	S1	S2	S3	S4	S5	S6	S7
Twinspan cluster level 4		7	7	8	8	9	9	10	10	10	10	10	10	13	13	14	14	15	16
Twinspan cluster level 5-6		0	1	0	1	0	1		0-0	0-1	1-0	1-1		0	1	0	1	0	1
No. of relevés		301	135	994	235	846	765	760	1324	730	1316	177	709	65	690	174	250	192	66
Carex firma	14	.	.	.	.	.	.	.	.	.	.	.	.	.	.	.	.	7	.
Centaurea reichenbachii	14	.	.	.	.	.	.	.	1	1	.	.	1	.	1	.	.	.	.
Chaerophyllum aromaticum	14	.	2	1	.	.	1	.	.	.	.	.	.	.	.	.	.	.	.
Dianthus capitatus	14	.	.	.	.	.	.	.	1	.	1	.	.	.	.	.	.	.	.
Festuca beckeri	14	.	.	.	.	.	.	1	1	.	1	.	.	.	.	.	.	.	.
Festuca heterophylla	14	.	.	1	.	1	.	1	.	.	1	.	.	.	1	.	.	.	.
Gypsophila oligosperma	14	.	.	.	.	.	1	.	.	.	1	.	1	.	.	.	.	.	.
Hieracium laevigatum	14	.	1	1	.	1	.	.	.	.	.	.	1	.	1	.	.	2	.
Hieracium racemosum	14	1	.	1	.	1	.	1	1	.	.	.	.	.	1	.	.	.	.
Inula spiraeifolia	14	.	.	.	.	.	.	1	1	.	.	.	2	.	.	.	.	.	.
Marrubium vulgare	14	.	.	.	.	.	.	.	1	1	1	.	1	.	.	.	.	.	.
Mentha longifolia	14	2	.	1	.	1	1	1	1	1	1	.	.	.	.	.	.	.	.
Orchis mascula	14	.	.	1	1	1	1	.	.	.	.	.	.	.	.	.	.	.	.
Rumex confertus	14	.	.	.	.	.	.	1	1	.	1	.	.	.	.	.	.	.	.
Saponaria officinalis	14	1	.	.	.	.	1	1	1	1	1	.	.	.	.	.	.	1	.
Scutellaria supina	14	.	.	.	.	.	.	.	.	.	1	6	.	.	.	.	.	.	.
Senecio umbrosus	14	.	.	1	.	.	.	.	.	.	.	.	.	.	1	.	.	1	.
Senecio vernalis	14	.	.	.	.	.	.	1	1	.	1	2	.	.	.	.	.	.	.
Stachys annua	14	.	1	.	.	1	.	1	.	1	1	.	1	.	.	.	.	.	.
Tragopogon ucrainicus	14	.	.	.	.	.	.	1	1	.	1	.	.	.	.	.	.	.	.
Arenaria longifolia	13	.	.	.	.	.	.	1	.	1	1	.	1	.	.	.	.	.	.
Carex melanostachya	13	1	.	.	.	.	.	.	.	.	1	1	.	.	.	.	.	.	.
Festuca vaginata	13	.	.	.	.	.	.	.	1	1	.	.	.	.	.	.	.	.	.
Lathyrus hirsutus	13	1	.	.	.	.	1	.	.	.	1	.	.	.	.	.	.	.	.
Lathyrus pallescens	13	.	.	1	.	.	1	1	.	1	.	.	.	.	.	.	.	.	.
Lychnis coronaria	13	.	.	.	.	.	1	1	1	.	.	.	1	.	.	.	.	.	.
Minuartia glomerata	13	.	.	.	.	.	.	.	1	.	1	.	1	.	1	.	1	.	.
Pulmonaria officinalis agg.	13	1	.	1	.	1	1	1	.	.	.	.	.	.	.	.	.	2	.
Pulsatilla patens	13	.	.	1	.	.	1	.	.	.	1	.	1	.	1	.	.	.	.
Ranunculus sardous	13	1	.	1	.	1	.	1	.	.	1	.	.	.	.	.	.	.	.
Ventenata dubia	13	.	.	.	.	.	.	1	1	.	1	.	.	.	.	.	.	.	.
Adonis volgensis	12	.	.	.	.	.	.	1	.	.	1	4	.	.	.	.	.	.	.
Allium schoenoprasum	12	.	.	1	.	.	.	.	1	1	.	.	1	.	1	.	.	.	2
Anchusa procera	12	.	.	.	.	.	.	.	.	.	1	.	.	.	.	.	.	.	.
Anthemis arvensis	12	1	.	1	.	.	.	.	1	.	1	.	1	.	.	.	.	.	.
Bellevalia speciosa	12	.	.	.	.	.	.	.	.	.	1	5	.	.	.	.	.	.	.
Campanula serrata	12	.	.	1	.	.	.	.	.	.	.	.	.	.	.	.	.	1	.
Carex divisa	12	1	.	.	.	.	.	1	.	1	1	.	.	.	.	.	.	.	.
Dianthus carbonatus	12	.	.	.	.	.	.	.	.	.	1	2	.	.	.	.	.	.	.
Dichoropetalum carvifolia	12	.	.	1	.	1	1	.	.	1	.	.	.	.	1	.	.	.	.
Diplotaxis muralis	12	1	.	.	.	1	.	1	1	1	1	.	.	.	.	.	.	.	.

Group number		B1	B2	B3	B4	B5	B6	B7	F1	F2	F3	F4	S1	S2	S3	S4	S5	S6	S7
Twinspan cluster level 4		7	7	8	8	9	9	10	10	10	10	10	10	13	13	14	14	15	16
Twinspan cluster level 5-6		0	1	0	1	0	1		0-0	0-1	1-0	1-1		0	1	0	1	0	1
No. of relevés		301	135	994	235	846	765	760	1324	730	1316	177	709	65	690	174	250	192	66
Festuca psammophila	12	.	.	.	.	.	.	1	1	.	.	.	1	.	1	.	.	2	.
Galium sylvaticum	12	.	.	1	.	1	.	.	1	.	1	.	1	.	.	.	.	1	.
Goniolimon besseranum	12	.	.	.	.	.	.	.	1	.	.	6	.	.	.	.	.	.	.
Haplophyllum suaveolens	12	.	.	.	.	.	.	.	.	.	.	7	.	.	.	.	.	.	.
Iris graminea	12	1	.	1	.	.	1	1	.	.	.	.	.	.	.	.	.	.	.
Linaria biebersteinii	12	.	.	.	.	.	.	.	.	.	1	6	.	.	.	.	.	.	.
Mentha arvensis	12	.	.	1	.	1	1	1	.	.	1	.	1	.	.	.	.	.	.
Onosma heterophylla	12	.	.	.	.	.	.	.	1	1	.	.	1	.	1	.	.	.	.
Ophrys sphegodes	12	.	.	.	1	1	1	1	1	.	.	.	.	.	.	.	.	.	.
Ornithogalum pyrenaicum s.lat.	12	.	.	.	4	1	.	.	.	.	.	.	.	.	.	.	.	.	.
Polygonatum verticillatum	12	.	.	1	.	.	.	.	.	.	.	.	.	.	.	.	.	2	.
Salvia glutinosa	12	1	.	.	.	1	.	.	.	.	1	.	.	.	.	.	.	2	.
Seseli austriacum	12	.	.	.	.	.	.	.	.	.	.	.	.	.	1	6	.	.	.
Solidago gigantea	12	1	.	1	.	1	.	.	1	.	.	.	.	.	.	.	.	.	.
Sonchus asper	12	1	.	.	.	.	1	1	1	1	.	1	1	.	.	.	.	.	.
Stipa ucrainica	12	.	.	.	.	.	.	.	.	.	.	7	.	.	.	.	.	.	.
Syrenia cana	12	.	.	.	.	.	.	.	1	.	1	1	.	.	.	.	.	.	.
Tragopogon podolicus	12	.	.	.	.	.	.	1	1	1	1	.	.	.	.	.	.	.	.
Verbascum blattaria	12	1	.	1	.	.	.	1	1	1	1	.	.	.	.	1	.	.	.
Viola riviniana	12	1	.	1	.	1	.	.	.	.	.	.	.	.	1	.	.	1	.
Xanthium strumarium	12	.	.	.	.	.	.	1	.	1	1	.	1	.	.	.	.	.	.
Xeranthemum cylindraceum	12	1	1	1	.	.	1	.	1	1	.	.	.	.	.	.	.	.	.
Achillea crithmifolia	11	.	.	.	.	.	.	1	1	.	.	.	1	.	.	.	.	.	.
Dianthus collinus	11	.	.	.	.	.	1	1	1	.	.	.	.	.	.	.	.	.	.
Fumaria officinalis	11	.	.	.	.	.	1	.	1	.	.	.	1	.	.	.	.	.	.
Galium spurium	11	.	.	.	.	.	.	1	1	.	1	.	.	.	.	.	.	.	.
Grindelia squarrosa	11	.	.	.	.	.	.	.	.	.	1	5	.	.	.	.	.	.	.
Hieracium glaucum	11	.	.	.	.	.	.	.	.	.	.	.	1	.	.	6	.	.	.
Lactuca saligna	11	2	.	.	.	.	.	.	1	.	1	.	.	.	.	.	.	.	.
Microrrhinum minus	11	.	1	.	.	1	.	1	1	1	1	.	1	.	1	.	.	.	.
Onosma pseudoarenaria	11	.	.	.	.	.	.	1	1	1	.	.	.	.	.	.	.	.	.
Potentilla patula	11	.	.	1	.	.	1	.	.	1	1	.	.	.	.	.	.	.	.
Sempervivum tectorum	11	.	.	.	.	.	.	.	1	1	.	.	1	.	1	.	.	1	2
Sinapis arvensis	11	1	.	1	.	.	.	1	.	1	1	.	.	.	.	.	.	.	.
Sisymbrium loeselii	11	.	.	.	.	.	.	.	1	.	1	1	1	.	.	.	.	.	.
Vulpia myuros	11	.	.	.	.	.	.	1	1	.	1	.	1	.	.	.	.	.	.
Aegilops cylindrica	10	.	.	.	.	.	.	.	.	1	.	4	1	.	.	.	.	.	.
Angelica sylvestris	10	.	.	1	.	1	.	.	.	.	1	.	.	.	.	.	.	1	.
Centaurea arenaria agg.	10	1	.	1	.	.	.	1	1	1	1	.	.	.	.	.	.	.	.
Centaurea solstitialis	10	1	.	.	.	.	.	1	1	1	1	.	.	.	.	.	.	.	.
Crepis nicaeensis	10	.	.	1	.	.	1	.	1	1	.	.	1	.	.	.	.	.	.

Group number		B1	B2	B3	B4	B5	B6	B7	F1	F2	F3	F4	S1	S2	S3	S4	S5	S6	S7
Twinspan cluster level 4		7	7	8	8	9	9	10	10	10	10	10	10	13	13	14	14	15	16
Twinspan cluster level 5-6		0	1	0	1	0	1		0-0	0-1	1-0	1-1		0	1	0	1	0	1
No. of relevés		301	135	994	235	846	765	760	1324	730	1316	177	709	65	690	174	250	192	66
Cyanus dominii	10	.	.	.	.	.	.	.	.	.	.	.	.	.	.	.	.	5	.
Dianthus campestris	10	.	.	.	.	.	.	1	.	.	1	.	.	.	.	.	.	.	.
Draba nemorosa	10	.	.	.	.	.	.	1	1	.	1	.	.	.	.	.	.	.	.
Euphorbia saxatilis	10	.	.	.	.	.	.	.	.	.	.	.	.	.	.	6	.	.	.
Gagea pusilla	10	.	.	.	.	.	1	.	1	.	1	.	1	.	.	.	1	.	.
Gentianella austriaca	10	.	.	1	.	1	.	.	.	.	.	.	.	.	1	.	.	1	.
Geranium sylvaticum	10	.	.	1	.	1	.	.	.	.	.	.	.	.	.	.	.	1	.
Gymnadenia odoratissima	10	.	.	1	.	1	.	.	.	.	.	.	.	.	1	.	.	2	.
Koeleria brevis	10	.	.	.	.	.	.	.	.	.	1	5	.	.	.	.	.	.	.
Melica uniflora	10	.	.	.	.	.	1	1	.	.	.	.	1	.	1	.	.	.	.
Onosma tornensis	10	.	.	.	.	.	.	.	1	.	.	.	1	.	1	1	.	.	.
Physospermum cornubiense	10	.	.	.	.	.	1	.	.	1	.	.	.	.	.	.	.	.	.
Plantago arenaria	10	.	.	.	.	1	.	.	1	.	1	.	.	.	.	.	.	.	.
Polygonum arenarium	10	.	.	.	.	.	.	.	1	1	1	.	.	.	.	.	.	.	.
Potentilla anserina	10	1	.	1	.	.	1	1	1	.	1	.	.	.	.	.	.	.	.
Potentilla rupestris	10	.	.	1	.	.	1	.	1	.	.	.	.	.	.	.	.	.	.
Prospero autumnale	10	.	.	.	.	.	.	.	.	1	1	.	1	.	.	.	2	.	.
Rindera umbelata	10	.	.	.	.	.	.	.	1	1	.	.	1	.	.	.	.	.	.
Selinum carvifolia	10	1	.	1	.	.	1	1	.	.	1	.	.	.	.	.	.	.	.
Senecio nemorensis agg.	10	.	.	1	.	1	.	.	.	.	.	.	.	.	.	.	.	4	.
Senecio vulgaris	10	.	2	1	.	.	.	.	1	.	.	.	1	.	1	.	.	.	.
Seseli peucedanoides	10	.	.	1	.	.	1	.	.	1	.	.	.	.	.	.	.	.	.
Spiraea chamaedryfolia	10	.	.	.	.	.	.	.	.	.	.	.	.	.	1	.	.	.	9
Symphytum officinale	10	1	.	1	.	1	1	.	1	.	1	.	.	.	.	.	.	.	.
Trifolium patens	10	.	.	1	.	.	.	.	.	.	1	.	.	.	.	.	.	.	.
Allium marginatum	9	.	.	.	.	.	1	.	1	1	1	.	.	.	.	.	.	.	.
Anagallis foemina	9	.	.	1	.	1	.	.	1	1	1	.	1	.	.	.	.	.	.
Anthyllis schiwereckii	9	.	.	.	.	.	1	.	.	.	1	.	1	.	.	.	.	.	.
Artemisia abrotanum	9	.	.	.	.	.	.	1	1	.	1	.	.	.	.	.	.	.	.
Bassia laniflora	9	.	.	.	.	.	.	.	1	.	1	1	.	.	.	.	.	.	.
Bromus commutatus	9	1	.	1	.	.	1	.	1	.	1	.	.	.	.	.	.	.	.
Campanula macrostachya	9	.	.	1	.	.	1	1	1	.	.	.	.	.	.	.	.	.	.
Carex vulpina agg.	9	1	.	.	.	.	.	.	1	.	1	.	.	.	.	.	.	.	.
Cirsium oleraceum	9	1	1	1	.	.	.	1	.	.	.	.	.	.	.	.	.	.	.
Corydalis solida	9	.	.	.	.	.	.	.	.	.	.	.	1	.	1	.	.	.	.
Cypripedium calceolus	9	.	.	.	.	1	1	.	.	.	.	.	.	.	.	.	.	1	.
Cytisus blockianus	9	.	.	.	.	.	1	1	1	.	1	.	.	.	.	.	.	.	.
Cytisus podolicus	9	.	.	1	.	.	1	.	1	.	1	.	.	.	.	.	.	.	.
Dactylorhiza maculata	9	1	.	1	.	1	1	.	.	.	.	.	.	.	.	.	.	.	.
Dianthus barbatus	9	.	.	1	3	.	.	.	.	.	.	.	.	.	.	.	.	.	.
Dianthus pseudobarbatus	9	.	.	.	.	.	.	1	1	.	1	.	.	.	.	.	.	.	.

		B1	B2	B3	B4	B5	B6	B7	F1	F2	F3	F4	S1	S2	S3	S4	S5	S6	S7
Group number		B1	B2	B3	B4	B5	B6	B7	F1	F2	F3	F4	S1	S2	S3	S4	S5	S6	S7
Twinspan cluster level 4		7	7	8	8	9	9	10	10	10	10	10	10	13	13	14	14	15	16
Twinspan cluster level 5-6		0	1	0	1	0	1		0-0	0-1	1-0	1-1		0	1	0	1	0	1
No. of relevés		301	135	994	235	846	765	760	1324	730	1316	177	709	65	690	174	250	192	66
Dipsacus fullonum	9	1	.	1	.	1	.	1	.	.	1	.	.	.	.	.	.	.	.
Euphorbia salicifolia	9	.	.	1	.	1	1	1	1	1	1	.	.	.	.	.	.	.	.
Geranium dissectum	9	1	.	.	.	.	.	1	1	1	1	.	.	.	.	.	.	.	.
Helictotrichon desertorum	9	.	.	.	.	.	1	1	.	.	1	.	1	.	.	.	.	.	.
Juncus compressus	9	.	.	1	.	.	1	1	.	.	1	.	.	.	.	.	.	.	.
Ochlopoa annua	9	.	.	1	.	1	1	1	1	.	1	.	.	.	.	.	.	.	.
Orobanche flava	9	.	.	.	.	1	.	.	1	.	1	.	.	.	.	.	.	.	.
Parnassia palustris	9	.	.	1	1	1	.	.	.	.	.	.	.	.	1	.	.	2	2
Poa alpina	9	.	.	.	.	.	.	.	.	.	.	.	.	.	.	.	.	2	6
Rindera tetraspis	9	.	.	.	.	.	.	.	.	.	.	5	.	.	.	.	.	.	.
Rorippa sylvestris	9	1	.	.	.	1	.	1	1	.	1	.	.	.	.	.	.	.	.
Salix repens	9	.	.	1	2	1	.	1	.	.	.	.	1	.	.	.	.	.	.
Trifolium micranthum	9	1	.	.	.	.	.	.	.	.	1	.	.	.	.	.	.	.	.
Trinia multicaulis	9	.	.	.	.	.	.	.	1	1	1	.	1	.	.	.	.	.	.
Valerianella rimosa	9	.	.	1	.	.	.	.	1	1	.	.	.	.	1	.	.	.	.
Veronica bachofenii	9	.	.	.	.	.	.	.	1	.	.	.	1	.	.	.	.	.	.
Aconitum lycoctonum	8	.	.	.	.	.	.	.	.	.	.	.	1	.	.	.	.	2	6
Allium angulosum	8	1	.	1	.	.	.	.	.	.	1	.	.	.	.	.	.	.	.
Anthericum liliago	8	.	.	.	.	1	1	1	.	1	.	.	.	.	.	.	.	.	.
Artemisia annua	8	.	.	.	.	.	.	.	1	1	1	.	.	.	.	.	.	.	.
Crocus vernus	8	.	.	1	3	.	.	.	.	.	.	.	.	.	.	.	.	.	.
Dactylorhiza sambucina	8	1	.	1	.	.	.	.	.	.	.	.	.	.	.	.	.	.	.
Dracocephalum austriacum	8	.	.	.	.	.	1	.	.	.	.	.	1	.	1	.	.	.	.
Elytrigia elongata	8	.	.	.	.	.	.	.	.	.	1	2	.	.	.	.	.	.	.
Equisetum pratense	8	1	.	1	.	1	.	.	.	1	1	.	.	.	.	.	.	.	.
Equisetum variegatum	8	.	.	.	.	1	1	1	.	.	1	.	.	.	.	.	.	.	.
Galeopsis pubescens	8	.	.	.	.	1	.	1	.	.	.	.	1	.	1	.	.	.	.
Galium abaujense	8	.	.	.	.	.	1	1	1	1	.	.	.	.	.	.	.	.	.
Gentiana asclepiadea	8	.	.	1	1	1	.	.	.	.	.	.	.	.	.	.	.	2	.
Helictochloa compressa	8	.	.	.	.	.	1	1	1	1	.	.	.	.	.	.	.	.	.
Himantoglossum adriaticum	8	.	.	1	.	.	1	1	.	.	.	.	.	.	.	1	.	.	.
Hypochaeris glabra	8	1	.	.	.	1	.	1	.	.	.	.	.	.	1	.	.	2	.
Jurinea salicifolia	8	.	.	.	.	.	.	.	.	.	1	1	.	.	.	.	.	.	.
Lamium galeobdolon	8	.	.	1	.	.	.	.	.	.	.	.	1	.	.	.	.	2	.
Leonurus cardiaca	8	1	.	.	.	.	.	.	1	1	1	1	.	.	.	.	.	.	.
Moehringia trinervia	8	.	.	1	.	1	1	1	.	.	.	.	.	.	1	.	.	1	.
Oxalis acetosella	8	.	.	1	.	.	.	1	1	.	.	.	.	.	.	.	.	2	.
Piptatherum virescens	8	.	.	.	.	.	.	1	1	.	.	.	.	.	1	.	.	.	.
Schivereckia podolica	8	.	.	.	.	.	.	.	1	1	.	.	1	.	.	.	.	.	.
Selinum dubium	8	.	2	1	.	.	.	.	.	.	1	.	.	.	.	.	.	.	.
Senecio viscosus	8	.	.	.	.	.	.	1	1	.	.	.	1	.	.	.	.	.	.

Group number		B1	B2	B3	B4	B5	B6	B7	F1	F2	F3	F4	S1	S2	S3	S4	S5	S6	S7
Twinspan cluster level 4		7	7	8	8	9	9	10	10	10	10	10	10	13	13	14	14	15	16
Twinspan cluster level 5-6		0	1	0	1	0	1		0-0	0-1	1-0	1-1		0	1	0	1	0	1
No. of relevés		301	135	994	235	846	765	760	1324	730	1316	177	709	65	690	174	250	192	66
Silaum silaus	8	1	.	1	.	1	.	.	.	.	.	1	.	.	.	.	.	.	.
Silene conica	8	.	.	.	.	.	.	.	1	1	.	.	1	.	.	.	.	.	.
Silene viridiflora	8	.	.	.	.	.	.	1	1	1	1	.	.	.	1	.	.	.	.
Stachys sylvatica	8	1	1	1	.	.	.	.	.	.	1	.	.	.	.	.	.	.	.
Teucrium scorodonia	8	.	.	.	.	.	.	1	1	.	.	.	1	.	.	.	.	.	.
Thalictrum uncinatum	8	.	.	.	.	.	1	.	.	1	1	.	1	.	.	.	.	.	.
Trifolium angulatum	8	1	.	.	.	.	.	.	.	.	1	.	.	.	.	.	.	.	.
Valerianella coronata	8	.	.	.	.	.	.	.	1	1	1	.	.	.	.	.	.	.	.
Veronica barrelieri	8	.	.	.	.	.	1	1	1	.	1	1	.	.	.	.	.	.	.
Viola pumila	8	1	.	1	.	.	.	1	.	1	1	.	.	.	.	.	.	.	.
Allium fuscum	7	.	.	.	.	.	.	1	.	1	.	.	1	.	.	.	.	.	.
Allium inaequale	7	.	.	.	.	.	.	.	.	.	.	4	.	.	.	.	.	.	.
Alyssum repens	7	.	.	.	.	.	.	.	.	.	.	.	1	.	1	.	.	.	3
Arctium tomentosum	7	.	4	.	.	.	.	.	.	.	1	.	.	.	.	.	.	.	.
Asperula tenella	7	.	.	.	.	.	.	.	.	.	.	.	1	.	.	.	.	.	6
Astragalus peterfii	7	.	.	.	.	.	.	.	.	1	.	.	1	.	.	.	.	.	.
Bromus racemosus	7	1	.	1	.	.	.	1	.	.	1	.	.	.	.	.	.	.	.
Bunias orientalis	7	.	.	.	.	.	1	1	1	.	1	.	.	.	.	.	.	.	.
Bupleurum tenuissimum	7	.	.	.	.	.	.	.	.	.	1	.	.	.	.	.	.	.	.
Carex sylvatica	7	1	.	1	.	.	.	.	.	.	.	.	.	.	.	.	.	.	.
Centaurea salonitana	7	.	.	.	.	.	.	.	.	.	1	3	.	.	.	.	.	.	.
Cephalanthera rubra	7	1	.	.	.	.	.	.	.	.	1	.	.	.	1	.	.	1	.
Cyclamen purpurascens	7	.	.	.	1	1	.	.	.	.	.	.	.	.	1	.	.	.	.
Echinops banaticus	7	.	.	.	.	.	.	.	1	1	.	.	1	.	.	.	.	.	.
Epipactis helleborine	7	.	.	.	.	.	.	.	1	.	.	.	1	.	1	.	.	2	2
Erodium ruthenicum	7	.	.	.	.	.	.	.	.	.	1	.	1	.	.	.	.	.	.
Filago lutescens	7	.	.	.	.	.	.	.	1	.	.	.	.	.	.	.	.	.	.
Galium cracoviense	7	.	.	.	.	1	.	1	.	.	.	.	1	.	.	.	.	.	.
Gentianella amarella	7	.	.	1	.	1	.	.	.	.	.	.	.	.	1	.	.	1	.
Iris spuria	7	1	.	.	.	.	.	1	1	.	1	.	.	.	.	.	.	.	.
Jovibarba heuffelii	7	.	.	.	.	.	.	.	1	.	.	.	1	.	.	.	.	.	3
Laserpitium archangelica	7	.	.	.	.	.	.	.	.	.	.	.	.	.	.	.	.	4	.
Leucanthemum rotundifolium	7	.	.	.	.	.	.	.	.	.	.	.	.	.	.	.	.	4	.
Limonium platyphyllum	7	.	.	.	.	.	.	.	.	.	1	2	.	.	.	.	.	.	.
Lysimachia vulgaris	7	.	.	1	.	.	1	1	.	1	.	.	.	.	.	.	.	.	.
Ophioglossum vulgatum	7	.	.	1	.	.	.	.	.	.	.	.	.	.	.	.	.	.	.
Orobanche arenaria	7	.	.	.	.	.	.	.	1	.	1	.	.	.	.	.	.	.	.
Papaver albiflorum subsp. austromora	7	.	.	.	.	1	.	1	1	.	1	.	.	.	.	.	.	.	.
Phleum hirsutum	7	.	.	1	1	.	1	.	.	.	.	.	.	.	.	.	.	.	.
Rorippa pyrenaica	7	.	.	1	.	.	1	.	.	.	.	.	.	.	.	.	.	.	.
Scorzonera humilis	7	.	.	1	.	1	.	.	.	.	.	.	.	.	1	.	.	.	.

Group number		B1	B2	B3	B4	B5	B6	B7	F1	F2	F3	F4	S1	S2	S3	S4	S5	S6	S7
Twinspan cluster level 4		7	7	8	8	9	9	10	10	10	10	10	10	13	13	14	14	15	16
Twinspan cluster level 5-6		0	1	0	1	0	1		0-0	0-1	1-0	1-1		0	1	0	1	0	1
No. of relevés		301	135	994	235	846	765	760	1324	730	1316	177	709	65	690	174	250	192	66
Scorzonera mollis	7	.	.	.	.	.	.	.	1	.	.	1	.	.	.	.	.	.	.
Sisymbrium altissimum	7	.	.	.	.	.	.	.	1	.	1	.	.	.	.	.	.	.	.
Trifolium fragiferum	7	1	.	.	.	.	.	1	.	.	1	.	.	.	.	.	.	.	.
Trifolium pallidum	7	2	.	.	.	.	.	.	.	.	.	.	.	.	.	.	.	.	.
Trisetum sibiricum	7	.	.	.	.	.	.	.	.	.	1	.	.	.	.	.	.	.	.
Xanthium orientale	7	1	.	.	.	1	.	.	.	.	1	.	.	.	.	.	.	.	.
Adonis aestivalis	6	.	.	.	.	.	.	1	1	1	1	.	.	.	.	.	.	.	.
Agrimonia procera	6	.	.	.	.	.	.	.	.	1	1	1	.	.	.	.	.	.	.
Althaea officinalis	6	1	.	.	.	.	.	1	.	.	1	.	.	.	.	.	.	.	.
Asperula rumelica	6	.	.	.	.	.	.	.	.	1	1	.	1	.	.	.	.	.	.
Asplenium cuneifolium	6	.	.	.	.	.	1	.	.	.	.	.	1	.	1	.	.	.	.
Asplenium x alternifolium	6	.	.	.	.	.	.	.	1	.	.	.	.	3	.	.	.	.	.
Carduus uncinatus	6	.	.	.	.	.	.	.	.	.	1	2	.	.	.	.	.	.	.
Centaurea stereophylla	6	.	.	.	.	.	.	1	.	1	1	1	1	.	.	.	.	.	.
Centaurea triniifolia	6	.	.	.	.	.	.	.	1	.	.	.	1	2	.	.	.	.	.
Cyanus segetum	6	.	.	.	.	1	.	1	.	1	1	.	.	.	.	.	.	.	.
Dianthus henteri	6	.	.	.	.	.	.	.	1	.	.	.	.	.	.	.	.	.	.
Dianthus moravicus	6	.	.	.	.	.	.	.	.	.	.	.	1	2	1	.	.	.	.
Dianthus nitidus	6	.	.	.	.	.	.	.	.	.	.	.	.	.	1	.	.	3	.
Digitalis ferruginea	6	.	1	1	.	1	.	.	.	.	.	.	.	.	.	.	.	.	.
Diplotaxis tenuifolia	6	.	1	.	.	.	.	.	1	1	.	.	.	.	.	.	.	.	.
Doronicum columnae	6	.	.	.	.	.	.	.	.	.	.	.	.	.	.	.	.	.	9
Draba muralis	6	.	.	.	.	.	.	1	1	.	.	.	.	.	1	.	.	.	.
Elymus caninus	6	.	.	1	.	1	.	1	.	.	1	.	.	.	.	.	.	1	.
Elytrigia stipifolia	6	.	.	.	.	.	.	.	.	.	.	3	.	.	.	.	.	.	.
Erodium ciconium	6	.	.	.	.	.	.	.	.	.	1	.	.	.	.	.	.	.	.
Euphorbia leptocaula	6	.	.	.	.	.	.	.	.	.	.	3	.	.	.	.	.	.	.
Euphrasia parviflora	6	.	.	.	.	.	1	.	.	1	.	.	1	.	.	.	.	.	.
Galatella sedifolia	6	1	.	.	.	.	1	.	.	.	1	.	.	.	.	.	.	.	.
Geranium lucidum	6	.	.	.	.	.	.	.	1	.	.	.	.	.	.	.	.	.	3
Geranium molle	6	.	.	.	.	.	.	.	.	1	1	.	.	.	.	.	.	.	.
Gladiolus imbricatus	6	.	.	1	.	.	.	.	.	.	.	.	.	.	.	.	.	.	.
Hedysarum grandiflorum	6	.	.	.	.	.	.	.	.	.	1	2	1	.	.	.	.	.	.
Helminthotheca echioides	6	2	.	.	.	.	.	.	.	.	1	.	.	.	.	.	.	.	.
Iris pontica	6	.	.	1	.	.	1	.	.	1	.	1	.	.	.	.	.	.	.
Lamium maculatum	6	.	.	.	.	1	.	.	.	.	1	.	.	.	1	.	.	1	.
Lapsana communis	6	1	.	.	.	1	1	1	1	.	.	.	1	.	.	.	.	.	.
Leontopodium nivale subsp. alpinum	6	.	.	.	.	.	.	.	.	.	.	.	.	.	.	.	.	3	.
Lotus angustissimus	6	1	.	.	.	.	.	.	.	.	1	.	.	.	.	.	.	.	.
Malabaila graveolens	6	.	.	.	.	.	.	.	.	.	1	3	.	.	.	.	.	.	.
Melampyrum polonicum	6	.	.	.	.	.	1	.	1	.	.	.	1	.	.	.	.	.	.

Group number		B1	B2	B3	B4	B5	B6	B7	F1	F2	F3	F4	S1	S2	S3	S4	S5	S6	S7
Twinspan cluster level 4		7	7	8	8	9	9	10	10	10	10	10	10	13	13	14	14	15	16
Twinspan cluster level 5-6		0	1	0	1	0	1		0-0	0-1	1-0	1-1		0	1	0	1	0	1
No. of relevés		301	135	994	235	846	765	760	1324	730	1316	177	709	65	690	174	250	192	66
Ornithogalum comosum	6	.	.	.	.	.	.	.	.	.	.	.	1	.	.	.	2	.	.
Orobanche coerulescens	6	.	.	.	.	.	.	.	1	.	1	.	.	.	.	.	.	1	.
Polygala nicaeensis	6	1	.	1	.	.	.	.	.	.	.	.	.	.	.	.	.	.	.
Ranunculus ficaria	6	1	.	1	.	.	1	.	.	1	1	.	.	.	.	.	.	.	.
Reseda luteola	6	.	.	.	.	.	.	1	1	.	1	.	1	.	.	.	.	.	.
Rhaponticoides ruthenica	6	.	.	.	.	.	1	1	.	1	1	.	.	.	.	.	.	.	.
Rhinanthus alpinus	6	.	.	.	.	1	.	.	.	.	.	.	.	.	.	.	.	2	.
Satureja kitaibelii	6	.	.	.	.	.	.	.	.	.	.	.	1	.	.	.	.	.	2
Securigera elegans	6	.	.	.	.	.	.	.	.	.	.	.	.	.	.	.	.	.	.
Senecio borysthenicus	6	.	.	.	.	.	.	.	.	.	1	.	.	.	.	.	.	.	.
Senecio erraticus	6	1	.	1	.	1	1	.	.	1	.	.	.	.	.	.	.	.	.
Seseli montanum subsp. tommasinii	6	.	.	.	.	.	.	.	.	.	.	.	1	.	1	.	.	.	.
Soldanella carpatica	6	.	.	.	.	.	.	.	.	.	.	.	.	.	.	.	.	3	.
Sonchus oleraceus	6	.	1	.	.	.	.	1	1	.	1	.	.	.	.	.	.	.	.
Thymus calcareus	6	.	.	.	.	.	.	1	.	.	1	.	1	.	.	.	.	.	.
Thymus pallasianus	6	.	.	.	.	.	.	.	1	.	1	.	.	.	.	.	.	.	.
Tulipa biebersteiniana	6	.	.	.	.	.	.	.	1	.	1	1	.	.	.	.	.	.	.
Vaccinium vitis-idaea	6	.	.	1	.	.	1	.	.	.	.	.	.	.	.	.	.	2	.
Veronica longifolia	6	.	1	.	.	.	1	1	.	.	1	.	.	.	.	.	.	.	.
Veronica spuria	6	.	.	.	.	.	1	1	.	1	1	.	.	.	.	.	.	.	.
Viola accrescens	6	.	.	.	.	.	.	.	.	.	1	.	1	.	.	.	.	.	.
Xanthium spinosum	6	.	.	.	.	.	.	1	1	1	.	.	.	.	.	.	.	.	.
Achillea micrantha	5	1	1	.	.	.	.	.	.	.	1	.	.	.	.	.	.	.	.
Aconitum confertiflorum	5	.	.	.	.	.	1	.	.	1	.	.	1	.	.	.	.	.	.
Anemone narcissiflora	5	.	.	.	.	.	1	.	.	.	.	.	.	.	.	.	.	.	.
Arabidopsis halleri	5	.	.	1	.	.	.	.	.	.	.	.	.	.	.	.	.	.	.
Arabis alpina	5	.	.	.	.	.	.	.	.	.	.	.	.	.	1	.	.	1	.
Arabis pauciflora	5	.	.	1	.	.	1	.	.	.	.	.	.	.	1	.	.	.	.
Arenaria rigida	5	.	.	.	.	.	.	.	1	.	1	1	.	.	.	.	.	.	.
Artemisia santonicum	5	1	1	.	.	.	.	.	.	.	1	1	.	.	.	.	.	.	.
Astragalus albicaulis	5	.	.	.	.	.	.	.	.	.	1	1	1	.	.	.	.	.	.
Astragalus ponticus	5	.	.	.	.	.	.	.	.	.	1	2	.	.	.	.	.	.	.
Atriplex sagittata	5	.	.	.	.	.	.	1	1	1	1	.	.	.	.	.	.	.	.
Bupleurum longifolium	5	.	.	.	.	.	.	1	1	.	.	.	.	.	1	1	.	.	.
Campanula crassipes	5	.	.	.	.	.	.	.	.	.	.	.	1	.	.	.	.	.	6
Carex pediformis	5	.	.	.	.	1	.	1	.	.	1	.	.	.	1	.	.	1	.
Centaurea pugioniformis	5	.	.	1	.	.	1	.	.	.	.	.	.	.	.	.	.	.	.
Centaurium pulchellum	5	1	.	1	.	1	.	.	1	1	.	.	.	.	.	.	.	.	.
Cephalanthera damasonium	5	.	.	1	.	1	.	.	.	.	.	.	.	.	1	.	.	1	.
Coeloglossum viride	5	.	.	.	.	1	1	.	.	.	.	.	1	.	.	.	.	.	.
Cortusa matthioli	5	.	.	.	.	.	.	.	.	.	.	.	.	.	.	.	.	2	2

Group number		B1	B2	B3	B4	B5	B6	B7	F1	F2	F3	F4	S1	S2	S3	S4	S5	S6	S7
Twinspan cluster level 4		7	7	8	8	9	9	10	10	10	10	10	10	13	13	14	14	15	16
Twinspan cluster level 5-6		0	1	0	1	0	1		0-0	0-1	1-0	1-1		0	1	0	1	0	1
No. of relevés		301	135	994	235	846	765	760	1324	730	1316	177	709	65	690	174	250	192	66
Crepis pannonica	5	.	.	.	.	.	1	.	.	1	.	.	.	.	.	.	.	.	.
Crepis pulchra	5	.	.	.	.	.	.	.	1	1	.	.	.	.	.	.	.	.	.
Crepis vesicaria	5	1	.	1	.	.	.	.	.	.	.	.	.	.	.	.	.	.	.
Cymbaria borysthenica	5	.	.	.	.	.	.	.	.	.	.	3	.	.	.	.	.	.	.
Dianthus eugeniae	5	.	.	.	.	.	.	1	.	.	1	.	.	.	.	.	.	.	.
Doronicum hungaricum	5	.	.	.	.	.	1	1	1	.	.	.	1	.	.	.	.	.	.
Equisetum hyemale	5	.	.	.	.	1	1	1	.	.	.	.	.	.	.	.	.	.	.
Eragrostis minor	5	.	.	.	.	.	.	.	1	.	1	.	.	.	.	.	.	.	.
Fumana vulgaris	5	.	.	.	1	1	.	.	.	.	.	.	.	.	.	.	.	.	.
Gentianella fatrae	5	.	.	.	.	.	.	.	.	.	.	.	.	.	1	.	.	2	.
Gypsophila repens	5	.	.	.	1	1	.	.	.	.	.	.	.	.	.	.	.	1	.
Hieracium prenanthoides	5	.	.	.	.	.	.	.	.	.	.	.	.	.	.	.	.	2	.
Hierochloe australis	5	.	.	.	.	.	1	1	.	.	.	.	.	.	1	.	.	.	2
Hierochloe repens	5	.	.	.	.	.	.	1	.	.	1	.	.	.	.	.	.	.	.
Holcus mollis	5	.	.	1	1	1	.	.	.	.	1	.	.	.	.	.	.	.	.
Humulus lupulus	5	.	.	.	.	.	.	.	1	.	.	.	1	.	1	.	.	.	.
Juncus gerardii	5	.	.	.	.	.	.	.	.	.	1	.	.	.	.	.	.	.	.
Koeleria grandis	5	.	1	.	.	.	.	.	.	1	1	.	.	.	.	.	.	.	.
Limonium tomentellum	5	.	.	.	.	.	.	.	.	.	1	1	.	.	.	.	.	.	.
Melilotus sulcatus	5	.	.	.	.	.	.	.	.	.	.	.	1	.	1	.	.	.	.
Minuartia taurica	5	.	.	.	.	.	.	.	.	1	.	.	1	.	.	.	.	.	.
Onobrychis gracilis	5	.	.	.	.	.	.	.	.	.	1	2	.	.	.	.	.	.	.
Orchis simia	5	.	.	.	.	.	.	.	1	1	.	.	1	.	.	.	.	.	.
Orthilia secunda	5	.	.	.	.	.	.	.	1	.	.	.	.	.	.	.	.	.	.
Pedicularis acaulis	5	.	.	.	2	.	.	.	.	.	.	.	.	.	.	.	.	.	.
Polycnemum majus	5	.	.	.	.	.	.	1	1	.	1	.	.	.	.	.	.	.	.
Polygala cretacea	5	.	.	.	.	.	.	.	.	.	1	.	1	.	.	.	.	.	.
Polygonatum multiflorum	5	.	.	1	.	.	.	.	.	.	.	.	1	.	.	.	.	1	.
Portulaca oleracea	5	.	.	.	.	.	.	.	1	1	1	.	.	.	.	.	.	.	.
Potentilla aurea	5	.	.	.	.	1	.	.	.	.	.	.	.	.	1	.	.	1	.
Raphanus raphanistrum	5	1	.	.	.	.	.	.	1	.	1	.	.	.	.	.	.	.	.
Reseda phyteuma	5	.	.	.	.	.	.	.	1	.	.	.	.	.	.	.	2	.	.
Saponaria glutinosa	5	.	.	.	.	.	.	.	1	1	.	.	.	.	.	.	.	.	.
Scorzonera villosa	5	.	.	.	2	1	.	.	.	.	.	.	.	.	.	.	.	.	.
Scrophularia nodosa	5	1	.	1	.	1	.	.	.	.	.	.	1	.	.	.	.	.	.
Scrophularia rupestris	5	.	.	.	.	.	.	.	.	.	.	.	.	.	.	.	.	.	8
Secale sylvestre	5	.	.	.	.	.	.	.	1	.	1	.	.	.	.	.	.	.	.
Sisymbrium strictissimum	5	.	.	.	.	1	.	1	.	.	.	.	1	.	.	.	.	.	.
Solanum dulcamara	5	1	1	.	.	.	.	.	.	.	.	.	.	.	.	.	.	.	.
Spergularia rubra	5	.	.	.	.	.	.	1	1	.	1	.	.	.	.	.	.	.	.
Syrenia montana	5	.	.	.	.	.	.	.	.	.	1	.	.	.	.	.	.	.	.

Group number		B1	B2	B3	B4	B5	B6	B7	F1	F2	F3	F4	S1	S2	S3	S4	S5	S6	S7
Twinspan cluster level 4		7	7	8	8	9	9	10	10	10	10	10	10	13	13	14	14	15	16
Twinspan cluster level 5-6		0	1	0	1	0	1		0-0	0-1	1-0	1-1		0	1	0	1	0	1
No. of relevés		301	135	994	235	846	765	760	1324	730	1316	177	709	65	690	174	250	192	66
Trigonella gladiata	5	.	.	.	.	.	.	.	1	.	.	.	.	.	.	.	.	.	.
Valeriana montana	5	.	.	.	.	.	.	.	.	.	.	.	.	.	.	.	.	.	8
Vicia sylvatica	5	.	.	1	.	.	.	.	1	.	1	.	.	.	.	.	.	1	.
Vincetoxicum fuscatum	5	.	.	.	.	.	.	.	1	.	.	2	.	.	.	.	.	.	.
Waldsteinia geoides	5	.	.	.	.	1	1	.	.	.	.	.	1	.	.	.	.	.	.
Aethusa cynapium	4	.	.	.	.	.	.	1	.	.	.	.	1	.	.	.	.	.	.
Aira elegantissima	4	.	.	.	.	.	1	1	1	.	.	.	.	.	.	.	.	.	.
Ajuga orientalis	4	.	.	.	.	.	.	.	.	.	1	1	.	.	.	.	.	.	.
Allium denudatum	4	.	.	.	.	.	.	.	1	1	.	.	1	.	.	.	.	.	.
Androsace koso-poljanskii	4	.	.	.	.	.	.	.	.	.	1	.	1	.	.	.	.	.	.
Anthemis austriaca	4	.	.	.	.	.	1	.	1	.	1	.	.	.	.	.	.	.	.
Aristolochia pallida	4	.	.	.	.	.	.	.	1	.	.	.	1	.	.	.	.	.	.
Asperula tephrocarpa	4	.	.	.	.	.	.	.	.	.	1	.	1	.	.	.	.	.	.
Bupleurum pachnospermum	4	.	.	.	.	.	.	.	1	.	.	.	1	.	.	.	.	.	.
Bupleurum praealtum	4	.	.	.	.	.	.	1	1	.	.	.	.	.	.	.	.	.	.
Campanula latifolia	4	.	.	1	.	.	.	.	.	.	1	.	.	.	.	.	.	.	.
Campanula scheuchzeri	4	.	.	1	.	.	.	.	.	.	.	.	.	.	.	.	.	.	.
Cardamine amara	4	.	.	.	.	.	.	.	.	.	.	.	1	.	1	.	.	.	.
Carex depressa subsp. transsilvanica	4	.	.	1	.	.	.	.	1	.	.	.	1	.	.	.	.	.	.
Carex flava agg.	4	.	.	1	.	.	.	.	.	.	.	.	.	.	.	.	.	1	.
Carex pilosa	4	.	.	1	.	.	.	.	.	.	.	.	.	.	.	.	.	.	.
Centaurea lavrenkoana	4	.	.	.	.	.	.	1	.	.	1	.	.	.	.	.	.	.	.
Cerastium banaticum	4	.	.	.	.	.	.	.	.	.	.	.	1	2	.	.	.	.	2
Chaerophyllum temulum	4	1	.	1	.	.	.	.	1	.	1	.	.	.	.	.	.	.	.
Clinopodium menthifolium	4	.	.	.	.	.	1	.	.	.	.	.	.	.	.	.	.	.	2
Crepis mollis	4	.	.	1	.	1	.	.	.	.	.	.	.	.	.	.	.	1	.
Cyanus adscendens	4	.	.	.	.	1	.	.	.	.	.	.	.	.	.	.	.	.	.
Dianthus hypanicus	4	.	.	.	.	.	.	.	1	.	1	.	.	.	.	.	.	.	.
Dianthus pallens	4	.	.	.	.	.	.	.	.	.	1	2	.	.	.	.	.	.	.
Dianthus superbus	4	.	.	1	.	.	.	.	.	.	.	.	.	.	.	.	.	.	.
Epilobium tetragonum	4	1	.	.	.	1	.	1	.	.	1	.	.	.	.	.	.	.	.
Equisetum palustre	4	1	.	1	.	.	.	.	.	1	.	.	.	.	.	.	.	.	.
Equisetum telmateia	4	.	.	1	.	.	1	.	.	.	.	.	.	.	.	.	.	.	.
Erysimum sylvestre	4	.	.	.	.	.	.	.	.	.	.	.	.	.	1	2	.	.	.
Euphorbia bessarabica	4	.	.	.	.	.	.	.	.	.	1	2	.	.	.	.	.	.	.
Euphorbia palustris	4	1	1	1	.	.	.	.	.	.	1	.	.	.	.	.	.	.	.
Festuca amethystina	4	.	.	1	.	.	.	.	.	.	.	.	.	.	.	.	.	2	.
Fumaria schleicheri	4	.	.	.	.	1	1	.	1	.	1	.	.	.	.	.	.	.	.
Fumaria vaillantii	4	.	.	.	.	.	.	.	1	.	.	.	.	.	1	.	.	.	.
Gagea bulbifera	4	.	.	.	.	.	.	.	1	.	.	2	.	.	.	.	.	.	.
Galium volhynicum	4	.	.	.	.	.	.	.	.	.	1	1	.	.	.	.	.	.	.

Group number		B1	B2	B3	B4	B5	B6	B7	F1	F2	F3	F4	S1	S2	S3	S4	S5	S6	S7
Twinspan cluster level 4		7	7	8	8	9	9	10	10	10	10	10	10	13	13	14	14	15	16
Twinspan cluster level 5-6		0	1	0	1	0	1		0-0	0-1	1-0	1-1		0	1	0	1	0	1
No. of relevés		301	135	994	235	846	765	760	1324	730	1316	177	709	65	690	174	250	192	66
Gentiana pneumonanthe	4	1	.	1	.	.	.	.	.	.	.	.	1	.	.	.	.	.	.
Geranium sibiricum	4	.	1	.	.	.	.	.	.	.	1	.	.	.	.	.	.	.	.
Hyoscyamus niger	4	.	.	.	.	.	.	1	1	.	1	.	.	.	.	.	.	.	.
Hypericum tetrapterum	4	1	.	1	.	1	.	.	.	.	.	.	.	.	.	.	.	.	.
Inula helenium	4	1	.	.	.	.	1	.	.	.	1	.	.	.	.	.	.	.	.
Koeleria moldavica	4	.	.	.	.	.	.	.	.	.	.	2	.	.	.	.	.	.	.
Lactuca quercina	4	.	.	.	.	.	.	1	.	1	1	.	.	.	.	.	.	.	.
Leonurus quinquelobatus	4	.	1	.	.	.	.	1	.	.	1	.	.	.	.	.	.	.	.
Lepidium ruderale	4	.	.	.	.	.	.	.	.	.	1	.	1	.	.	.	.	.	.
Lilium carniolicum	4	.	.	.	1	1	.	.	.	.	.	.	.	.	.	.	.	.	.
Linum dolomiticum Borb.	4	.	.	.	.	.	.	.	.	.	.	.	.	.	1	2	.	.	.
Luzula pilosa	4	.	.	1	.	.	.	1	.	.	1	.	.	.	.	.	.	.	.
Medicago monspeliaca	4	.	.	.	.	.	.	.	1	.	.	.	1	.	.	.	.	.	.
Minuartia hybrida	4	.	.	.	.	.	.	.	.	1	1	1	.	.	.	.	.	.	.
Neslia paniculata	4	.	.	.	.	.	.	.	1	1	.	.	1	.	.	.	.	.	.
Onobrychis alba	4	.	.	.	.	.	.	.	1	1	.	.	1	.	.	.	.	.	.
Onosma simplicissima	4	.	.	.	.	.	.	.	.	.	1	.	1	.	.	.	.	.	.
Orobanche alsatica	4	.	.	1	.	1	1	.	.	.	.	.	.	.	.	.	.	.	.
Orobanche artemisiae-campestris	4	.	.	.	.	1	.	.	1	.	.	.	.	.	.	.	.	.	.
Oxalis stricta	4	.	.	.	.	.	1	1	1	.	.	.	.	.	.	.	.	.	.
Peucedanum austriacum	4	.	.	1	.	.	.	.	.	.	.	.	.	.	.	.	.	.	3
Pilosella aurantiaca	4	.	.	.	.	.	.	.	1	.	.	.	1	.	1	.	.	.	.
Piptatherum holciforme	4	.	.	.	.	.	.	.	.	.	.	.	1	.	.	.	.	.	.
Potentilla chrysantha	4	.	.	1	.	.	.	.	.	.	.	.	1	.	.	.	.	.	.
Prenanthes purpurea	4	.	.	.	.	.	.	.	.	.	.	.	.	.	.	.	.	2	.
Pulicaria vulgaris	4	1	.	.	.	.	.	.	.	.	.	.	.	.	.	.	.	.	.
Rumex alpestris	4	1	.	1	.	.	.	.	.	.	.	.	.	.	.	.	.	.	.
Rumex pulcher	4	1	.	.	.	.	.	.	.	.	1	.	.	.	.	.	.	.	.
Saxifraga adscendens	4	.	.	.	.	.	.	.	.	.	.	.	.	.	.	.	.	.	6
Saxifraga cuneifolia	4	.	.	.	.	.	.	.	.	.	.	.	.	.	.	.	.	.	6
Saxifraga marginata	4	.	.	.	.	.	.	.	.	.	.	.	.	.	.	.	.	.	6
Scutellaria hastifolia	4	1	.	.	.	.	1	.	.	.	1	.	.	.	.	.	.	.	.
Sedum dasyphyllum	4	.	.	.	.	.	.	.	1	1	.	.	.	.	.	.	.	.	3
Sherardia arvensis	4	1	.	.	.	.	.	1	.	.	1	.	.	.	.	.	.	.	.
Silene armeria	4	.	.	.	.	.	.	.	.	.	.	.	1	.	1	.	.	.	.
Solanum nigrum	4	1	.	.	.	.	.	.	1	.	.	.	.	.	.	.	.	.	.
Sporobolus vaginiflorus	4	.	1	.	.	.	.	1	.	.	1	.	.	.	.	.	.	.	.
Stachys alopecuros	4	.	.	1	1	.	.	.	.	.	.	.	.	.	.	.	.	.	.
Stipa asperella	4	.	.	.	.	.	.	.	.	.	.	2	.	.	.	.	.	.	.
Swertia perennis	4	.	.	.	.	.	.	.	.	.	.	.	.	.	.	.	.	2	.
Tephroseris longifolia	4	.	.	1	.	.	.	.	.	.	.	.	.	.	.	.	.	.	.

Group number		B1	B2	B3	B4	B5	B6	B7	F1	F2	F3	F4	S1	S2	S3	S4	S5	S6	S7
Twinspan cluster level 4		7	7	8	8	9	9	10	10	10	10	10	10	13	13	14	14	15	16
Twinspan cluster level 5-6		0	1	0	1	0	1		0-0	0-1	1-0	1-1		0	1	0	1	0	1
No. of relevés		301	135	994	235	846	765	760	1324	730	1316	177	709	65	690	174	250	192	66
Thesium dollineri	4	.	.	.	.	.	.	1	1	.	.	.	.	.	.	.	.	.	.
Tragopogon borysthenicus	4	.	.	.	.	.	.	.	.	.	1	.	.	.	.	.	.	.	.
Trifolium diffusum	4	.	.	.	.	.	.	.	1	.	1	.	.	.	.	.	.	.	.
Veronica agrestis	4	.	.	.	.	.	.	.	1	.	1	.	.	.	1	.	.	.	.
Veronica persica	4	.	1	.	.	.	.	.	.	1	1	.	.	.	.	.	.	.	.
Veronica polita	4	1	.	.	.	.	.	.	1	.	1	.	.	.	.	.	.	.	.
Veronica urticifolia	4	.	.	.	.	.	.	.	.	.	.	.	.	.	.	.	.	.	6
Viola biflora	4	.	.	.	.	.	.	.	.	.	.	.	.	.	.	.	.	2	.
Adenophora liliifolia	3	.	.	1	.	.	1	.	.	.	.	.	.	.	.	.	.	.	.
Alopecurus arundinaceus	3	.	.	.	.	.	.	1	.	.	1	.	.	.	.	.	.	.	.
Anacamptis palustris	3	.	.	.	.	.	.	.	.	1	1	.	.	.	.	.	.	.	.
Anchusa gmelinii	3	.	.	.	.	.	.	.	.	.	1	.	.	.	.	.	.	.	.
Anthriscus cerefolium	3	.	.	.	.	.	.	.	1	.	1	.	.	.	.	.	.	.	.
Arabis ciliata	3	.	.	.	1	1	.	.	.	.	.	.	.	.	.	.	.	.	.
Aremonia agrimonoides	3	1	.	1	.	.	.	.	.	.	.	.	.	.	.	.	.	.	.
Arenaria grandiflora	3	.	.	.	.	.	.	.	1	.	.	.	.	.	.	.	.	1	.
Arnica montana	3	.	.	.	1	.	.	.	.	.	.	.	.	.	.	.	.	.	.
Aruncus dioicus	3	.	.	.	.	.	.	.	.	.	1	.	.	.	.	.	.	1	.
Asclepias syriaca	3	.	.	.	.	.	.	1	1	.	1	.	.	.	.	.	.	.	.
Asperula neilreichii	3	.	.	.	.	.	.	.	.	.	.	.	.	.	1	.	.	1	.
Astragalus glycyphylloides	3	.	.	.	.	.	.	1	.	.	1	.	.	.	.	.	.	.	.
Astragalus pallescens	3	.	.	.	.	.	.	.	.	.	.	2	.	.	.	.	.	.	.
Astragalus pseudotataricus	3	.	.	.	.	.	.	.	.	.	.	2	.	.	.	.	.	.	.
Astragalus varius	3	.	.	.	.	.	.	.	.	.	1	.	.	.	.	.	.	.	.
Atriplex oblongifolia	3	.	.	.	.	.	.	.	.	.	1	.	.	.	.	.	.	.	.
Bassia scoparia	3	1	.	.	.	.	.	.	.	.	.	.	.	.	.	.	.	.	.
Bromus secalinus	3	.	.	.	.	.	.	.	1	1	.	.	.	.	.	.	.	.	.
Bupleurum rotundifolium	3	.	.	.	.	.	1	.	.	1	.	1	.	.	.	.	.	.	.
Calystegia sepium	3	1	.	1	.	.	.	.	.	.	.	.	.	.	.	.	.	.	.
Campanula thyrsoides	3	.	.	.	.	1	.	.	.	.	.	.	.	.	1	.	.	.	.
Cannabis sativa	3	.	.	.	.	.	.	.	.	.	1	.	.	.	.	.	.	.	.
Cardamine bulbifera	3	.	.	1	.	.	.	.	.	.	.	.	.	.	1	.	.	.	.
Carex echinata	3	.	1	.	.	.	.	1	.	.	.	.	.	.	.	.	.	.	.
Carex leporina	3	.	.	1	.	.	.	.	.	.	.	.	.	.	.	.	.	.	.
Centaurea pectinata	3	.	.	1	.	.	.	.	.	.	.	.	.	.	.	.	.	.	.
Cephalanthera longifolia	3	.	.	1	.	.	.	.	.	.	.	.	.	.	.	.	.	.	.
Cuscuta campestris	3	.	.	.	.	1	1	.	.	.	1	.	.	.	.	.	.	.	.
Cynoglossum hungaricum	3	.	.	.	.	.	.	.	1	.	.	.	1	.	.	.	.	.	.
Cytisus leiocarpus	3	.	.	.	.	.	.	.	.	.	.	.	1	.	1	.	.	.	.
Dactylorhiza fuchsii	3	.	.	1	.	.	.	.	.	.	.	.	.	.	.	.	.	.	.
Dianthus platyodon	3	.	.	.	.	.	.	.	.	.	1	.	.	.	.	.	.	.	.

Group number		B1	B2	B3	B4	B5	B6	B7	F1	F2	F3	F4	S1	S2	S3	S4	S5	S6	S7
Twinspan cluster level 4		7	7	8	8	9	9	10	10	10	10	10	10	13	13	14	14	15	16
Twinspan cluster level 5-6		0	1	0	1	0	1		0-0	0-1	1-0	1-1		0	1	0	1	0	1
No. of relevés		301	135	994	235	846	765	760	1324	730	1316	177	709	65	690	174	250	192	66
Dianthus tenuifolius	3	.	.	.	.	.	.	.	.	.	.	.	.	.	1	.	.	.	3
Dichoropetalum schottii	3	.	.	.	1	1	.	.	.	.	.	.	.	.	.	.	.	.	.
Digitalis lanata	3	.	.	.	.	1	1	1	.	.	.	.	.	.	.	.	.	.	.
Digitaria ischaemum	3	.	.	.	.	1	.	.	.	.	.	.	1	.	.	.	.	.	.
Digitaria sanguinalis	3	.	.	.	.	1	.	.	1	.	.	.	.	.	.	.	.	.	.
Dryopteris filix-mas	3	.	.	.	.	.	.	1	1	.	.	.	.	.	.	.	.	1	.
Epilobium collinum	3	.	.	.	.	.	.	1	1	.	.	.	.	.	.	.	.	.	.
Epipactis palustris	3	.	.	1	.	.	.	.	.	.	.	.	.	.	.	.	.	.	.
Erodium moschatum	3	.	.	.	.	.	.	.	1	.	1	.	.	.	.	.	.	.	.
Erysimum carniolicum	3	.	.	.	.	1	.	.	.	.	.	.	.	.	1	.	.	.	.
Euphorbia falcata	3	.	.	1	.	.	.	.	.	1	1	.	.	.	.	.	.	.	.
Euphorbia semivillosa	3	.	.	.	.	.	.	.	.	.	1	.	.	.	.	.	.	.	.
Ferula heuffelii	3	.	.	.	.	.	.	.	.	.	.	.	1	.	.	.	.	.	2
Foeniculum vulgare	3	.	.	.	1	1	.	.	.	.	.	.	.	.	.	.	.	.	.
Galega officinalis	3	1	.	.	.	.	.	.	.	.	.	.	.	.	.	.	.	.	.
Galium odoratum	3	.	.	1	.	.	.	1	.	.	1	.	.	.	.	.	.	.	.
Galium verrucosum	3	.	.	1	.	.	.	.	1	.	.	.	.	.	.	.	.	.	.
Glycyrrhiza echinata	3	1	.	.	.	.	.	.	.	.	1	.	.	.	.	.	.	.	.
Gnaphalium sylvaticum	3	1	.	1	.	.	.	.	.	.	.	.	.	.	.	.	.	.	.
Gypsophila petraea	3	.	.	.	.	.	.	.	.	.	.	.	.	.	1	.	.	.	2
Helictochloa adsurgens	3	.	.	1	.	.	1	.	.	.	.	.	.	.	.	.	.	.	.
Hesperis sylvestris	3	.	.	.	.	.	.	.	1	.	.	.	.	.	.	.	.	.	.
Hordeum murinum	3	.	.	.	.	.	.	.	.	.	1	.	.	.	.	.	.	.	.
Impatiens parviflora	3	.	.	.	.	1	.	.	.	.	.	.	1	.	1	.	.	.	.
Isatis praecox	3	.	.	.	.	.	.	.	.	1	.	.	.	.	1	.	.	.	.
Jurinea cyanoides	3	.	.	.	.	.	.	.	.	.	1	.	.	.	.	.	.	.	.
Lathyrus aphaca	3	1	.	.	.	.	1	.	.	.	1	.	.	.	.	.	.	.	.
Lathyrus sphaericus	3	.	.	.	.	.	.	.	1	.	.	.	.	.	.	.	.	.	.
Lepidium perfoliatum	3	.	.	.	.	.	.	.	.	.	1	1	.	.	.	.	.	.	.
Limonium aureum	3	1	.	.	.	.	.	1	.	.	1	.	.	.	.	.	.	.	.
Luzula luzulina	3	.	.	1	.	.	.	.	.	.	.	.	.	.	.	.	.	.	.
Lythrum salicaria	3	1	.	1	.	.	.	.	.	.	1	.	.	.	.	.	.	.	.
Malva alcea	3	1	.	.	.	1	.	.	.	.	.	.	.	.	.	.	.	.	.
Melampyrum bihariense	3	.	.	1	.	.	.	.	.	.	.	.	.	2	.	.	.	.	.
Melica altissima	3	.	.	.	.	.	.	.	1	.	1	.	.	.	.	.	.	.	.
Melica picta	3	.	.	.	.	.	1	.	.	1	.	.	.	.	1	.	.	.	.
Melissa officinalis	3	.	.	1	.	.	.	.	.	.	1	.	.	.	.	.	.	.	.
Myosotis sparsiflora	3	.	.	.	.	.	.	.	1	.	.	.	.	.	.	.	.	.	.
Neottia nidus-avis	3	.	.	1	.	.	.	.	.	.	.	.	.	.	1	.	.	.	.
Oenanthe silaifolia	3	.	.	1	.	.	.	.	.	.	1	.	.	.	.	.	.	.	.
Orchis pallens	3	.	.	.	.	.	1	.	.	.	.	.	.	.	.	1	.	.	.

Group number		B1	B2	B3	B4	B5	B6	B7	F1	F2	F3	F4	S1	S2	S3	S4	S5	S6	S7
Twinspan cluster level 4		7	7	8	8	9	9	10	10	10	10	10	10	13	13	14	14	15	16
Twinspan cluster level 5-6		0	1	0	1	0	1		0-0	0-1	1-0	1-1		0	1	0	1	0	1
No. of relevés		301	135	994	235	846	765	760	1324	730	1316	177	709	65	690	174	250	192	66
Ornithogalum boucheanum	3	.	.	.	.	.	.	1	.	.	1	.	.	.	.	.	.	.	.
Phegopteris connectilis	3	.	.	.	.	.	.	.	.	.	.	.	.	.	.	.	.	.	5
Pilosella floribunda	3	.	.	1	.	.	.	.	.	.	.	.	.	.	.	.	.	.	.
Pinguicula alpina	3	.	.	.	.	.	.	.	.	.	.	.	.	.	.	.	.	2	.
Plantago altissima	3	.	.	1	.	.	.	1	.	.	.	.	.	.	.	.	.	.	.
Pleurospermum austriacum	3	.	.	.	.	.	.	.	.	.	.	.	.	.	.	.	.	2	.
Poa chaixii	3	.	.	1	.	1	.	.	.	.	.	.	.	.	.	.	.	.	.
Poa palustris	3	1	.	.	.	.	.	.	.	.	1	.	.	.	.	.	.	.	.
Poa stiriaca	3	.	.	.	.	1	.	.	1	.	.	.	.	.	.	.	.	1	.
Potentilla caulescens	3	.	.	.	.	.	.	.	1	.	.	.	.	.	.	.	.	.	.
Ranunculus lanuginosus	3	.	.	1	.	.	.	1	.	.	.	.	.	.	.	.	.	.	.
Ranunculus pseudomontanus	3	.	.	.	.	.	.	.	.	.	.	.	.	.	.	.	.	2	.
Rumex obtusifolius	3	.	.	1	.	.	.	1	.	1	.	.	.	.	.	.	.	.	.
Rumex patientia	3	.	.	.	.	.	.	.	.	.	1	.	.	.	.	.	.	.	.
Sanicula europaea	3	.	.	1	.	.	.	.	.	.	.	.	.	.	.	.	.	1	.
Saxifraga aizoides	3	.	.	.	.	.	.	.	.	.	.	.	.	.	1	.	.	1	.
Scabiosa argentea	3	.	.	.	.	.	.	.	.	.	1	.	.	.	.	.	.	.	.
Silene dioica	3	.	.	.	.	.	.	.	.	.	.	.	.	.	.	.	.	1	.
Silene noctiflora	3	.	.	.	.	.	.	.	1	.	1	.	1	.	.	.	.	.	.
Sorghum halepense	3	.	.	.	.	.	.	1	.	.	1	.	.	.	.	.	.	.	.
Spergula arvensis	3	.	.	.	.	.	.	.	.	.	1	.	.	.	.	.	.	.	.
Taeniatherum caput-medusae	3	.	.	.	.	.	.	1	1	.	.	1	.	.	.	.	.	.	.
Thalictrum flavum	3	.	.	1	.	.	.	.	.	.	1	.	.	.	.	.	.	.	.
Thymus bihoriensis	3	.	.	.	.	.	.	.	1	1	.	.	.	.	.	.	.	.	.
Tordylium maximum	3	1	.	.	.	.	.	.	1	.	.	.	.	.	.	.	.	.	.
Trifolium subterraneum	3	1	.	.	.	.	.	.	.	.	1	.	.	.	.	.	.	.	.
Vicia dumetorum	3	1	.	1	.	.	.	.	.	.	.	.	.	.	.	.	.	.	.
Vicia lutea	3	1	.	.	.	.	1	.	1	.	.	.	.	.	.	.	.	.	.
Vicia serratifolia	3	.	.	.	.	.	.	.	1	.	1	.	.	.	.	.	.	.	.
Acanthus hungaricus	2	.	.	.	.	.	.	.	1	.	.	.	1	.	.	.	.	.	.
Achillea atrata	2	.	.	1	.	.	.	.	.	.	.	.	.	.	.	.	.	.	.
Achillea ptarmica	2	.	.	.	.	.	.	.	1	.	.	.	1	.	.	.	.	.	.
Agropyron tanaiticum	2	.	.	.	.	.	.	.	.	.	1	.	.	.	.	.	.	.	.
Aira caryophyllea	2	.	.	.	.	.	.	1	.	.	.	.	.	.	.	.	.	.	.
Alcea biennis	2	.	.	.	.	.	.	.	.	.	1	.	.	.	.	.	.	.	.
Alcea rugosa	2	.	.	.	.	.	.	.	.	.	.	1	.	.	.	.	.	.	.
Althaea hirsuta	2	.	.	.	.	.	.	.	.	1	.	.	.	.	.	.	.	.	.
Alyssum linifolium	2	.	.	.	.	.	.	.	.	1	.	1	.	.	.	.	.	.	.
Amaranthus retroflexus	2	.	.	.	.	.	.	1	.	1	.	.	.	.	.	.	.	.	.
Androsace chamaejasme	2	.	.	.	.	.	.	.	.	.	.	.	.	.	.	.	.	1	.
Androsace lactea	2	.	.	.	.	.	.	.	.	.	.	.	.	.	.	.	.	1	.

Group number		B1	B2	B3	B4	B5	B6	B7	F1	F2	F3	F4	S1	S2	S3	S4	S5	S6	S7
Twinspan cluster level 4		7	7	8	8	9	9	10	10	10	10	10	10	13	13	14	14	15	16
Twinspan cluster level 5-6		0	1	0	1	0	1		0-0	0-1	1-0	1-1		0	1	0	1	0	1
No. of relevés		301	135	994	235	846	765	760	1324	730	1316	177	709	65	690	174	250	192	66
Androsace septentrionalis	2	.	.	.	.	.	.	.	1	.	.	.	.	.	.	.	.	.	.
Anthemis odontostephana	2	.	1	.	.	.	.	.	.	.	.	.	.	.	.	.	.	.	.
Aphanes arvensis	2	.	.	.	.	.	.	.	1	.	.	.	.	.	.	.	.	.	.
Aposeris foetida	2	.	.	.	.	.	.	.	.	1	.	.	.	.	.	.	.	.	.
Arabidopsis petrogena	2	.	.	.	.	.	.	.	.	.	.	.	1	.	1	.	.	.	.
Arctium minus	2	.	1	.	.	.	.	.	.	.	1	.	.	.	.	.	.	.	.
Arctostaphylos uva-ursi	2	.	.	.	.	.	.	.	.	.	.	.	.	.	.	.	.	1	.
Artemisia hololeuca	2	.	.	.	.	.	.	.	.	.	1	.	1	.	.	.	.	.	.
Artemisia pancicii	2	.	.	.	.	.	.	.	.	1	.	.	.	.	.	.	.	.	.
Asphodelus albus	2	.	.	.	.	.	.	.	.	.	.	.	.	.	1	.	.	.	.
Astracantha arnacantha	2	.	.	.	.	.	.	1	.	.	1	.	.	.	.	.	.	.	.
Atriplex patula	2	1	.	.	.	.	.	.	.	.	1	.	.	.	.	.	.	.	.
Atriplex prostrata	2	.	.	.	.	.	.	.	.	.	1	.	.	.	.	.	.	.	.
Blackstonia perfoliata	2	.	.	.	.	.	.	1	.	.	.	.	.	.	.	.	.	.	.
Bromus rigidus	2	.	.	.	.	.	.	.	1	.	.	.	.	.	.	.	.	.	.
Campanula abietina	2	.	.	.	.	.	.	.	.	.	.	.	.	.	.	.	.	.	3
Carex brevicollis	2	.	.	.	.	.	.	.	.	.	.	.	1	.	.	.	.	.	2
Carex pulicaris	2	.	.	.	1	.	.	.	.	.	.	.	.	.	.	.	.	.	.
Carex riparia	2	.	.	.	.	.	.	1	.	.	.	.	.	.	.	.	.	.	.
Centaurea paniculata	2	.	.	.	.	.	.	.	1	1	.	.	.	.	.	.	.	.	.
Chenopodium glaucum	2	.	.	.	.	.	.	.	.	.	1	.	.	.	.	.	.	.	.
Chrysanthemum zawadskii	2	.	.	.	.	.	.	.	.	.	.	.	.	.	1	.	.	1	.
Cimicifuga europaea	2	.	.	.	.	.	1	.	.	.	.	.	.	.	.	.	.	1	.
Cirsium ukranicum	2	.	.	.	.	.	.	.	.	.	1	.	.	.	.	.	.	.	.
Colchicum arenarium	2	.	.	.	.	.	.	.	.	1	.	.	.	.	.	.	.	.	.
Conium maculatum	2	1	.	.	.	.	.	.	.	.	1	.	.	.	.	.	.	.	.
Corydalis cava	2	.	.	.	.	.	.	.	.	.	.	.	1	.	.	.	.	.	.
Crepis alpestris	2	.	.	.	.	1	.	.	.	.	.	.	.	.	.	.	.	1	.
Crepis capillaris	2	.	.	.	.	.	.	1	.	.	.	.	.	.	.	.	.	.	.
Crepis sancta	2	.	.	.	.	.	.	.	.	.	1	1	.	.	.	.	.	.	.
Cucubalus baccifer	2	.	.	.	.	.	1	.	.	.	.	.	.	.	.	.	.	.	.
Cynanchum acutum	2	.	.	.	.	.	.	.	.	.	.	1	.	.	.	.	.	.	.
Cynoglossum germanicum	2	.	.	.	.	.	.	.	1	.	.	.	.	.	.	.	.	.	.
Dasypyrum villosum	2	.	.	.	.	.	.	.	.	1	.	.	.	.	.	.	.	.	.
Delphinium elatum	2	.	.	.	.	.	.	.	.	.	.	.	.	.	.	.	.	1	.
Dianthus leptopetalus	2	.	.	.	.	.	.	.	.	.	1	.	.	.	.	.	.	.	.
Dipsacus pilosus	2	.	.	.	.	.	.	.	.	.	1	.	.	.	.	.	.	.	.
Dracocephalum ruyschiana	2	.	.	.	.	.	.	.	1	.	.	.	.	.	.	.	.	.	.
Echinophora sibthorpiana	2	.	.	.	.	.	.	.	.	.	.	.	1	.	.	.	.	.	.
Eleocharis palustris agg.	2	.	.	.	.	.	.	1	.	.	.	.	.	.	.	.	.	.	.
Erysimum repandum	2	.	.	.	.	1	.	.	1	.	.	.	.	.	.	.	.	.	.

Group number		B1	B2	B3	B4	B5	B6	B7	F1	F2	F3	F4	S1	S2	S3	S4	S5	S6	S7
Twinspan cluster level 4		7	7	8	8	9	9	10	10	10	10	10	10	13	13	14	14	15	16
Twinspan cluster level 5-6		0	1	0	1	0	1		0-0	0-1	1-0	1-1		0	1	0	1	0	1
No. of relevés		301	135	994	235	846	765	760	1324	730	1316	177	709	65	690	174	250	192	66
Euphorbia exigua	2	.	1	.	.	.	.	.	.	.	.	.	.	.	.	.	.	.	.
Festuca versicolor	2	.	.	.	.	.	.	.	.	.	.	.	.	.	.	.	.	1	.
Filipendula ulmaria	2	.	.	1	.	.	.	.	1	.	.	.	.	.	.	.	.	.	.
Gagea transversalis	2	.	.	.	.	.	.	.	.	.	1	.	.	.	.	.	.	.	.
Galeopsis tetrahit	2	.	.	1	.	.	.	.	.	.	1	.	.	.	.	.	.	.	.
Galium kitaibelianum	2	.	.	.	.	.	.	.	.	.	.	.	.	.	.	.	.	.	.
Galium tenuissimum	2	.	.	.	.	.	.	.	1	.	.	.	.	.	.	.	.	.	.
Geranium collinum	2	1	1	.	.	.	.	.	.	.	.	.	.	.	.	.	.	.	.
Geranium divaricatum	2	.	.	.	.	.	.	1	1	.	.	.	.	.	.	.	.	.	.
Geranium macrorrhizum	2	.	.	.	.	.	.	.	.	.	.	.	.	.	.	.	.	.	3
Gratiola officinalis	2	.	.	.	.	.	.	.	.	.	1	.	.	.	.	.	.	.	.
Hacquetia epipactis	2	.	.	1	.	.	.	.	.	.	.	.	.	.	.	.	.	1	.
Helictochloa hookeri subsp. schelliana	2	.	.	.	.	.	.	1	.	.	.	.	.	.	.	.	.	.	.
Hieracium caesium	2	.	.	.	.	.	.	.	.	.	.	.	1	.	.	.	.	1	.
Hieracium glaucinum	2	.	.	.	.	.	.	.	.	.	1	.	.	.	1	.	.	.	.
Hieracium pilosum	2	.	.	.	.	.	.	.	1	.	.	.	.	.	.	.	.	.	.
Hieracium saxatile	2	.	.	.	.	.	.	.	.	.	.	.	1	.	.	1	.	.	.
Hieracium transylvanicum	2	.	.	.	.	.	.	.	.	.	.	.	.	.	1	.	.	.	2
Hyparrhenia hirta	2	.	.	1	.	.	.	1	.	.	.	.	.	.	.	.	.	.	.
Hypochaeris uniflora	2	.	.	1	.	.	.	.	.	.	.	.	.	.	.	.	.	.	.
Iris ruthenica	2	.	.	.	.	.	.	.	.	.	.	.	.	.	1	.	.	.	.
Iva xanthiifolia	2	.	.	.	.	.	.	.	.	.	1	.	.	.	.	.	.	.	.
Juncus articulatus	2	.	.	.	.	.	1	1	.	.	.	.	.	.	.	.	.	.	.
Juncus conglomeratus	2	.	.	1	.	.	1	.	.	.	.	.	.	.	.	.	.	.	.
Kickxia spuria	2	.	.	.	.	.	1	.	.	1	.	.	.	.	.	.	.	.	.
Knautia x posoniensis	2	1	.	.	.	1	.	.	.	.	.	.	.	.	.	.	.	.	.
Lamium album	2	.	.	.	.	.	.	.	.	.	1	.	.	.	.	.	.	.	.
Leucanthemum platylepis	2	.	.	.	1	1	.	.	.	.	.	.	.	.	.	.	.	.	.
Ligularia carpathica	2	.	.	.	.	.	.	.	.	.	.	.	.	.	.	.	.	.	3
Lythrum virgatum	2	1	.	.	.	.	.	.	.	.	1	.	.	.	.	.	.	.	.
Maianthemum bifolium	2	.	.	1	.	.	1	.	.	.	.	.	.	.	.	.	.	.	.
Malaxis monophyllos	2	.	.	1	.	1	.	.	.	.	.	.	.	.	.	.	.	.	.
Malva moschata	2	1	.	.	.	1	.	.	.	.	.	.	.	.	.	.	.	.	.
Mentha pulegium	2	.	.	.	.	.	.	1	.	.	.	.	.	.	.	.	.	.	.
Myosotis scorpioides agg.	2	.	.	1	.	.	.	.	.	.	.	.	.	.	.	.	.	.	.
Narcissus pseudonarcissus	2	.	.	.	.	.	.	1	.	.	.	.	.	.	.	.	.	.	.
Nepeta cataria	2	.	.	.	.	.	1	.	.	.	1	.	.	.	.	.	.	.	.
Notholaena marantae	2	.	.	.	.	.	.	.	1	.	.	.	.	2	.	.	.	.	.
Orobanche reticulata	2	1	.	.	.	.	.	.	1	.	.	.	.	.	.	.	.	.	.
Parietaria officinalis	2	1	.	.	.	.	.	.	.	.	.	.	.	.	.	.	.	.	.
Paris quadrifolia	2	.	.	1	.	.	.	.	.	.	.	.	.	.	.	.	.	1	.

Group number		B1	B2	B3	B4	B5	B6	B7	F1	F2	F3	F4	S1	S2	S3	S4	S5	S6	S7
Twinspan cluster level 4		7	7	8	8	9	9	10	10	10	10	10	10	13	13	14	14	15	16
Twinspan cluster level 5-6		0	1	0	1	0	1		0-0	0-1	1-0	1-1		0	1	0	1	0	1
No. of relevés		301	135	994	235	846	765	760	1324	730	1316	177	709	65	690	174	250	192	66
Petasites albus	2	.	1	.	.	.	.	.	.	.	1	.	.	.	.	.	.	.	.
Peucedanum altissimum	2	.	.	.	.	1	.	.	.	.	.	.	.	.	.	.	.	.	.
Phalaroides arundinacea	2	1	.	.	.	.	1	.	.	.	.	.	.	.	.	.	.	.	.
Phedimus spurius	2	.	.	.	.	.	.	.	.	.	1	.	.	.	1	.	.	.	.
Plantago maxima	2	.	.	.	.	.	.	.	.	.	1	.	.	.	.	.	.	.	.
Platanthera chlorantha	2	.	.	.	.	.	.	.	.	.	.	.	.	.	1	.	.	.	.
Poa annua	2	.	.	.	.	.	.	.	.	.	1	.	.	.	.	.	.	.	.
Poa molinerii	2	.	.	.	.	.	.	.	.	.	.	.	.	.	.	.	.	1	.
Polygonum patulum	2	.	.	.	.	.	.	.	1	.	1	.	.	.	.	.	.	.	.
Potentilla micrantha	2	.	.	.	.	.	.	.	.	.	.	.	.	.	1	.	.	.	.
Prangos ferulacea	2	.	.	.	.	.	.	.	1	.	.	.	1	.	.	.	.	.	.
Ranunculus alpestris	2	.	.	.	.	.	.	.	.	.	.	.	.	.	.	.	.	1	.
Rorippa austriaca	2	.	.	.	.	.	.	.	.	.	1	.	.	.	.	.	.	.	.
Rumex scutatus	2	.	1	.	.	.	.	.	.	.	.	.	.	.	.	.	.	1	.
Ruscus aculeatus L.	2	.	.	.	.	.	.	.	.	.	.	.	1	.	.	.	.	.	.
Salsola kali	2	.	.	.	.	.	.	1	1	.	.	.	.	.	.	.	.	.	.
Sanguisorba verrucosa	2	.	.	1	.	.	.	.	.	.	.	.	.	.	.	.	.	.	.
Sclerochloa dura	2	.	.	.	.	.	.	.	1	.	1	.	.	.	.	.	.	.	.
Scorzonera parviflora	2	.	.	.	.	.	.	.	.	1	.	.	.	.	.	.	.	.	.
Scorzoneroides helvetica	2	.	.	1	.	.	.	.	.	.	.	.	.	.	.	.	.	.	.
Scrophularia scopolii	2	.	.	.	.	.	1	.	.	1	.	.	.	.	.	.	.	.	.
Sedum annuum	2	.	.	.	.	.	.	.	1	1	.	.	.	.	.	.	.	.	.
Senecio macrophyllus	2	.	.	.	.	.	.	.	.	.	1	.	.	.	.	.	.	.	.
Senecio subalpinus	2	.	.	.	.	.	.	.	.	.	.	.	.	.	.	.	.	1	.
Serratula coronata	2	.	.	.	.	.	.	.	.	.	1	.	.	.	.	.	.	.	.
Silene dichotoma	2	.	.	.	.	.	.	.	1	.	1	.	.	.	.	.	.	.	.
Silene gallica	2	.	.	.	.	.	.	.	1	.	.	.	1	.	.	.	.	.	.
Silene multiflora	2	.	.	.	.	.	.	.	1	.	.	1	.	.	.	.	.	.	.
Smyrnium perfoliatum	2	.	.	.	.	1	.	.	.	.	.	.	1	.	.	.	.	.	.
Soldanella montana agg.	2	.	.	.	.	.	.	.	.	.	.	.	.	.	.	.	.	1	.
Spiranthes spiralis	2	.	.	.	1	1	.	.	.	.	.	.	.	.	.	.	.	.	.
Stachys palustris	2	.	.	1	.	.	.	.	.	.	1	.	.	.	.	.	.	.	.
Stellaria palustris	2	.	.	1	.	.	1	.	.	.	.	.	.	.	.	.	.	.	.
Tanacetum macrophyllum	2	.	1	.	.	.	.	.	.	.	.	.	1	.	.	.	.	.	.
Taraxacum sect. Dioszegia	2	.	.	.	.	.	.	.	1	.	.	.	.	.	.	.	.	.	.
Taraxacum sect. Obliqua	2	.	.	.	.	.	.	1	.	.	.	.	1	.	.	.	.	.	.
Tephroseris czernijevii	2	.	.	.	.	.	.	.	.	.	1	.	1	.	.	.	.	.	.
Teucrium scordium	2	.	.	.	.	.	.	1	.	.	.	.	.	.	.	.	.	.	.
Thesium ebracteatum	2	.	.	.	.	.	.	.	1	.	1	.	.	.	.	.	.	.	.
Thesium pyrenaicum	2	.	.	1	.	1	.	.	.	.	.	.	.	.	.	.	.	.	.
Trifolium badium	2	.	.	.	.	1	.	.	.	.	.	.	1	.	.	.	.	.	.

Group number		B1	B2	B3	B4	B5	B6	B7	F1	F2	F3	F4	S1	S2	S3	S4	S5	S6	S7
Twinspan cluster level 4		7	7	8	8	9	9	10	10	10	10	10	10	13	13	14	14	15	16
Twinspan cluster level 5-6		0	1	0	1	0	1		0-0	0-1	1-0	1-1		0	1	0	1	0	1
No. of relevés		301	135	994	235	846	765	760	1324	730	1316	177	709	65	690	174	250	192	66
Tripolium pannonicum	2	.	.	.	.	.	.	.	.	.	1	.	.	.	.	.	.	.	.
Trollius europaeus	2	.	.	1	.	.	.	.	.	.	.	.	.	.	.	.	.	.	.
Turgenia latifolia	2	.	.	.	.	.	.	.	1	.	.	.	.	.	.	.	.	.	.
Vaccinium myrtillus	2	.	.	1	.	.	.	.	.	.	.	.	.	.	.	.	.	1	.
Veratrum album	2	.	.	1	.	.	.	.	.	.	.	.	.	.	.	.	.	.	.
Veronica acinifolia	2	1	.	.	.	.	.	.	.	.	.	.	.	.	.	.	.	.	.
Vicia cassubica	2	.	.	1	.	.	.	.	1	.	.	.	.	.	.	.	.	.	.
Vicia pisiformis	2	.	.	.	.	1	.	1	.	.	.	.	.	.	.	.	.	.	.
Woodsia ilvensis	2	.	.	.	.	.	.	.	.	.	.	.	1	2	.	.	.	.	.
Achillea clavennae	1	.	.	.	.	.	.	.	.	.	.	.	.	.	1	.	.	.	.
Achillea salicifolia	1	.	.	.	.	.	.	.	.	.	1	.	.	.	.	.	.	.	.
Actaea spicata	1	.	.	.	.	.	.	.	.	.	.	.	.	.	.	.	.	1	.
Agrimonia pilosa	1	.	.	.	.	.	.	.	.	.	1	.	.	.	.	.	.	.	.
Agropyron dasyanthum	1	.	.	.	.	.	.	.	.	.	1	.	.	.	.	.	.	.	.
Agrostemma githago	1	.	.	.	.	.	.	.	.	.	1	.	.	.	.	.	.	.	.
Alcea rosea	1	.	.	.	.	.	.	.	.	.	1	.	.	.	.	.	.	.	.
Allium atropurpureum	1	.	.	.	.	.	.	.	.	1	.	.	.	.	.	.	.	.	.
Allium decipiens	1	.	.	.	.	.	.	.	.	.	1	.	.	.	.	.	.	.	.
Allium regelianum	1	.	.	.	.	.	.	.	.	.	.	.	1	.	.	.	.	.	.
Allium strictum	1	.	.	.	.	.	.	.	.	.	.	.	1	.	.	.	.	.	.
Allium suaveolens	1	.	.	1	.	.	.	.	.	.	.	.	.	.	.	.	.	.	.
Alopecurus rendlei	1	.	.	.	.	.	1	.	.	.	.	.	.	.	.	.	.	.	.
Althaea cannabina	1	.	.	.	.	.	.	.	.	1	.	.	.	.	.	.	.	.	.
Alyssum smyrnaeum	1	.	.	.	.	.	.	.	.	.	1	.	.	.	.	.	.	.	.
Amaranthus albus	1	.	.	.	.	.	.	1	.	.	.	.	.	.	.	.	.	.	.
Amaranthus hybridus	1	.	.	.	.	.	.	.	.	.	1	.	.	.	.	.	.	.	.
Amaranthus powellii	1	.	.	.	.	.	.	.	1	.	.	.	.	.	.	.	.	.	.
Anagallis minima	1	1	.	.	.	.	.	.	.	.	.	.	.	.	.	.	.	.	.
Anchusa ochroleuca	1	.	.	.	.	.	.	.	.	.	1	.	.	.	.	.	.	.	.
Anemone ranunculoides	1	.	.	1	.	.	.	.	.	.	.	.	.	.	.	.	.	.	.
Anemone trifolia	1	.	.	.	.	1	.	.	.	.	.	.	.	.	.	.	.	.	.
Angelica archangelica	1	.	.	.	.	.	.	.	.	.	1	.	.	.	.	.	.	.	.
Antennaria carpatica	1	.	.	.	1	.	.	.	.	.	.	.	.	.	.	.	.	.	.
Anthriscus caucalis	1	.	.	.	.	.	.	.	1	.	.	.	.	.	.	.	.	.	.
Anthriscus nitidus	1	.	.	.	.	.	.	.	.	.	1	.	.	.	.	.	.	.	.
Aquilegia einseleana	1	.	.	1	.	.	.	.	.	.	.	.	.	.	.	.	.	.	.
Aquilegia transsilvanica	1	.	.	.	.	.	.	.	.	.	1	.	.	.	.	.	.	.	.
Arabis soyeri	1	.	.	.	.	.	.	.	.	.	.	.	.	.	.	.	.	1	.
Arctium lappa	1	.	.	.	.	.	.	.	.	.	1	.	.	.	.	.	.	.	.
Arctium nemorosum	1	.	.	.	.	.	.	.	.	.	1	.	.	.	.	.	.	.	.
Arenaria cephalotes	1	.	.	.	.	.	.	.	.	.	.	1	.	.	.	.	.	.	.

Group number		B1	B2	B3	B4	B5	B6	B7	F1	F2	F3	F4	S1	S2	S3	S4	S5	S6	S7
Twinspan cluster level 4		7	7	8	8	9	9	10	10	10	10	10	10	13	13	14	14	15	16
Twinspan cluster level 5-6		0	1	0	1	0	1		0-0	0-1	1-0	1-1		0	1	0	1	0	1
No. of relevés		301	135	994	235	846	765	760	1324	730	1316	177	709	65	690	174	250	192	66
Arenaria ciliata	1	.	.	.	.	.	.	.	1	.	.	.	.	.	.	.	.	.	.
Artemisia lerchiana	1	.	.	.	.	.	.	.	.	.	.	1	.	.	.	.	.	.	.
Asperugo procumbens	1	.	.	.	.	.	.	1	.	.	.	.	.	.	.	.	.	.	.
Astragalus pubiflorus	1	.	.	.	.	.	.	.	.	.	1	.	.	.	.	.	.	.	.
Astrantia carniolica	1	.	.	1	.	.	.	.	.	.	.	.	.	.	.	.	.	.	.
Avena fatua	1	.	.	.	.	.	.	.	1	.	.	.	.	.	.	.	.	.	.
Bartsia alpina	1	.	.	.	.	.	.	.	.	.	.	.	.	.	.	.	.	1	.
Bidens tripartitus	1	.	1	.	.	.	.	.	.	.	.	.	.	.	.	.	.	.	.
Bifora radians	1	.	.	.	.	.	.	.	.	1	.	.	.	.	.	.	.	.	.
Bromus benekenii	1	.	.	.	.	.	.	.	1	.	.	.	.	.	.	.	.	.	.
Bromus cappadocica	1	.	.	.	.	.	.	.	.	.	1	.	.	.	.	.	.	.	.
Bunium bulbocastanum	1	.	.	.	.	.	.	1	.	.	.	.	.	.	.	.	.	.	.
Calamagrostis canescens	1	.	1	.	.	.	.	.	.	.	.	.	.	.	.	.	.	.	.
Calamagrostis pseudophragmites	1	.	.	.	.	.	.	.	.	1	.	.	.	.	.	.	.	.	.
Calendula officinalis	1	.	.	.	.	1	.	.	.	.	.	.	.	.	.	.	.	.	.
Calepina irregularis	1	.	.	.	.	.	.	.	.	.	1	.	.	.	.	.	.	.	.
Camelina rumelica	1	.	.	.	.	.	.	.	.	.	.	1	.	.	.	.	.	.	.
Campanula alpina	1	.	.	.	.	.	.	.	.	.	.	.	.	.	.	.	.	1	.
Campanula cespitosa	1	.	.	.	.	1	.	.	.	.	.	.	.	.	.	.	.	.	.
Campanula lingulata	1	.	.	.	.	.	.	.	.	.	.	.	1	.	.	.	.	.	.
Cardamine hirsuta	1	1	.	.	.	.	.	.	.	.	.	.	.	.	.	.	.	.	.
Carduus carduelis	1	.	.	.	.	.	.	.	1	.	.	.	.	.	.	.	.	.	.
Carduus personata	1	.	.	1	.	.	.	.	.	.	.	.	.	.	.	.	.	.	.
Carex acutiformis	1	1	.	.	.	.	.	.	.	.	.	.	.	.	.	.	.	.	.
Carex cespitosa	1	1	.	.	.	.	.	.	.	.	.	.	.	.	.	.	.	.	.
Carex diluta	1	.	.	.	.	.	.	.	.	.	1	.	.	.	.	.	.	.	.
Carex fritschii	1	.	.	.	.	.	.	1	.	.	.	.	.	.	.	.	.	.	.
Carex nigra	1	.	.	.	.	.	.	.	.	.	1	.	.	.	.	.	.	.	.
Carex obtusata	1	.	.	.	.	.	.	.	1	.	.	.	.	.	.	.	.	.	.
Carex paniculata	1	.	.	.	.	.	.	.	.	.	.	.	.	.	.	.	.	.	.
Carex strigosa	1	.	.	.	.	1	.	.	.	.	.	.	.	.	.	.	.	.	.
Centaurea besseriana	1	.	.	.	.	.	.	.	1	.	.	.	.	.	.	.	.	.	.
Cephalaria laevigata	1	.	.	.	.	.	.	.	.	.	.	.	1	.	.	.	.	.	.
Cerastium tomentosum	1	.	.	.	.	.	.	.	.	.	.	.	.	.	1	.	.	.	.
Ceratocephala orthoceras	1	.	.	.	.	.	.	.	1	.	.	.	.	.	.	.	.	.	.
Cerinthe glabra	1	.	.	.	.	.	.	.	.	.	.	.	.	.	.	.	.	.	.
Chenopodium ficifolium	1	.	.	.	.	.	.	.	.	.	1	.	.	.	.	.	.	.	.
Cirsium boujartii	1	.	.	.	.	.	.	1	.	.	.	.	.	.	.	.	.	.	.
Cirsium rivulare	1	.	.	1	.	.	.	.	.	.	.	.	.	.	.	.	.	.	.
Cirsium waldsteinii	1	.	.	.	1	.	.	.	.	.	.	.	.	.	.	.	.	.	.
Clinopodium suaveolens	1	.	.	.	.	.	.	.	.	.	.	.	1	.	.	.	.	.	.

Group number		B1	B2	B3	B4	B5	B6	B7	F1	F2	F3	F4	S1	S2	S3	S4	S5	S6	S7
Twinspan cluster level 4		7	7	8	8	9	9	10	10	10	10	10	10	13	13	14	14	15	16
Twinspan cluster level 5-6		0	1	0	1	0	1		0-0	0-1	1-0	1-1		0	1	0	1	0	1
No. of relevés		301	135	994	235	846	765	760	1324	730	1316	177	709	65	690	174	250	192	66
Colchicum hungaricum	1	.	.	.	.	.	.	.	1	.	.	.	.	.	.	.	.	.	.
Colchicum versicolor	1	.	.	.	.	.	.	.	.	.	1	.	.	.	.	.	.	.	.
Comandra umbellata	1	.	.	.	.	.	.	.	.	1	.	.	.	.	.	.	.	.	.
Comarum palustre	1	.	.	1	.	.	.	.	.	.	.	.	.	.	.	.	.	.	.
Conioselinum tataricum	1	.	.	.	.	.	.	.	.	.	.	.	.	.	.	.	.	.	2
Coronopus squamatus	1	.	.	.	.	.	.	.	.	.	.	.	1	.	.	.	.	.	.
Crepis froelichiana subsp. dinarica	1	.	.	.	.	1	.	.	.	.	.	.	.	.	.	.	.	.	.
Crepis paludosa	1	.	.	.	.	.	.	.	.	.	.	.	.	.	.	.	.	1	.
Crypsis aculeata	1	.	.	.	.	.	.	.	.	.	1	.	.	.	.	.	.	.	.
Cuscuta approximata	1	.	.	.	.	.	.	.	1	.	.	.	.	.	.	.	.	.	.
Cuscuta epilinum	1	.	.	.	.	.	.	.	.	.	.	.	.	.	1	.	.	.	.
Cytisus borysthenicus	1	.	.	.	.	.	.	.	.	.	1	.	.	.	.	.	.	.	.
Cytisus paczoskii	1	.	.	.	.	.	.	1	.	.	.	.	.	.	.	.	.	.	.
Dactylorhiza incarnata	1	.	.	.	.	.	1	.	.	.	.	.	.	.	.	.	.	.	.
Dactylorhiza majalis	1	.	.	1	.	.	.	.	.	.	.	.	.	.	.	.	.	.	.
Dianthus guttatus	1	.	.	.	.	.	.	.	.	.	.	1	.	.	.	.	.	.	.
Dianthus pseudoserotinus	1	.	.	.	.	.	.	.	.	.	.	.	1	.	.	.	.	.	.
Dianthus speciosus	1	.	.	.	.	.	.	.	.	.	1	.	.	.	.	.	.	.	.
Dianthus sylvestris	1	.	.	.	.	1	.	.	.	.	.	.	.	.	.	.	.	.	.
Dioscorea communis	1	.	.	1	.	.	.	.	.	.	.	.	.	.	.	.	.	.	.
Diplotaxis cretacea	1	.	.	.	.	.	.	.	.	.	.	.	1	.	.	.	.	.	.
Dipsacus sativus	1	1	.	.	.	.	.	.	.	.	.	.	.	.	.	.	.	.	.
Dracocephalum thymiflorum	1	.	.	.	.	.	.	.	.	.	1	.	.	.	.	.	.	.	.
Dryas octopetala	1	.	.	.	.	.	.	.	.	.	.	.	.	.	.	.	.	1	.
Echinops exaltatus	1	.	.	.	.	.	.	.	.	1	.	.	.	.	.	.	.	.	.
Edraianthus graminifolius	1	.	.	.	.	.	.	.	.	.	.	.	.	.	1	.	.	.	.
Elytrigia bessarabica	1	.	.	.	.	.	.	.	.	.	1	.	.	.	.	.	.	.	.
Epilobium ciliatum	1	.	.	.	.	.	.	.	.	.	.	.	1	.	.	.	.	.	.
Epilobium lanceolatum	1	.	.	.	.	.	.	.	1	.	.	.	.	.	.	.	.	.	.
Epilobium palustre	1	.	.	.	.	.	.	.	.	.	.	.	1	.	.	.	.	.	.
Epilobium parviflorum	1	1	.	.	.	.	.	.	.	.	.	.	.	.	.	.	.	.	.
Epilobium roseum	1	.	1	.	.	.	.	.	.	.	.	.	.	.	.	.	.	.	.
Epimedium alpinum	1	.	.	.	1	.	.	.	.	.	.	.	.	.	.	.	.	.	.
Equisetum sylvaticum	1	.	.	.	.	.	.	1	.	.	.	.	.	.	.	.	.	.	.
Erechtites hieraciifolius	1	.	.	.	.	.	.	.	1	.	.	.	.	.	.	.	.	.	.
Erigeron glabratus	1	.	.	.	1	.	.	.	.	.	.	.	.	.	.	.	.	.	.
Eryngium amethystinum	1	.	.	.	.	1	.	.	.	.	.	.	.	.	.	.	.	.	.
Euphorbia nutans	1	.	.	.	.	.	.	.	.	.	1	.	.	.	.	.	.	.	.
Euphrasia nemorosa agg.	1	.	.	.	.	1	.	.	.	.	.	.	.	.	.	.	.	.	.
Festuca altissima	1	.	.	.	.	1	.	.	.	.	.	.	.	.	.	.	.	.	.
Fritillaria ruthenica	1	.	.	.	.	.	.	.	.	.	1	.	.	.	.	.	.	.	.

Group number		B1	B2	B3	B4	B5	B6	B7	F1	F2	F3	F4	S1	S2	S3	S4	S5	S6	S7
Twinspan cluster level 4		7	7	8	8	9	9	10	10	10	10	10	10	13	13	14	14	15	16
Twinspan cluster level 5-6		0	1	0	1	0	1		0-0	0-1	1-0	1-1		0	1	0	1	0	1
No. of relevés		301	135	994	235	846	765	760	1324	730	1316	177	709	65	690	174	250	192	66
Gagea lutea	1	.	.	.	.	.	.	.	1	.	.	.	.	.	.	.	.	.	.
Gagea pratensis	1	.	.	.	.	.	.	.	.	.	1	.	.	.	.	.	.	.	.
Galanthus nivalis	1	.	.	.	.	.	.	.	.	.	.	.	1	.	.	.	.	.	.
Galinsoga parviflora	1	.	.	.	.	.	.	.	.	.	1	.	.	.	.	.	.	.	.
Galium flavescens	1	.	.	.	.	.	.	.	.	1	.	.	.	.	.	.	.	.	.
Galium intermedium	1	.	.	.	.	.	.	.	.	.	1	.	.	.	.	.	.	.	.
Galium rivale	1	.	.	.	.	.	.	.	.	.	1	.	.	.	.	.	.	.	.
Galium uliginosum	1	.	.	1	.	.	.	.	.	.	.	.	.	.	.	.	.	.	.
Gaudinia fragilis	1	.	.	1	.	.	.	.	.	.	.	.	.	.	.	.	.	.	.
Geranium phaeum	1	.	.	.	.	.	.	.	.	.	1	.	.	.	.	.	.	.	.
Geranium pyrenaicum	1	.	.	.	.	.	.	.	.	.	1	.	.	.	.	.	.	.	.
Gladiolus palustris	1	.	.	1	.	.	.	.	.	.	.	.	.	.	.	.	.	.	.
Glaucium corniculatum	1	.	.	.	.	.	.	.	.	1	.	.	.	.	.	.	.	.	.
Gnaphalium uliginosum	1	.	.	.	.	.	.	.	.	.	1	.	.	.	.	.	.	.	.
Gymnadenia borealis	1	.	.	1	.	.	.	.	.	.	.	.	.	.	.	.	.	.	.
Helianthus pauciflorus	1	.	.	.	.	.	.	.	.	.	.	.	.	.	.	.	.	1	.
Helictochloa planiculmis	1	1	.	.	.	.	.	.	.	.	.	.	.	.	.	.	.	.	.
Hieracium hypochoeroides subsp. wie	1	.	.	.	.	.	.	.	.	.	.	.	.	.	1	.	.	.	.
Hieracium maculatum	1	.	.	.	.	.	.	.	.	.	.	.	.	.	1	.	.	.	.
Hieracium onosmoides	1	.	.	.	.	.	.	.	.	.	.	.	.	.	.	.	.	.	.
Hieracium piliferum	1	.	.	.	.	.	.	.	.	.	.	.	.	.	.	.	.	1	.
Hieracium porrifolium	1	.	.	.	.	1	.	.	.	.	.	.	.	.	.	.	.	.	.
Hieracium pseudobifidum subsp. treb	1	.	.	.	.	.	.	.	.	.	.	.	.	.	.	.	.	.	2
Hieracium schmidtii	1	.	.	.	.	.	.	.	.	.	.	.	.	.	.	.	.	1	.
Hieracium sparsum	1	.	.	.	.	.	.	.	.	.	.	.	.	.	1	.	.	.	.
Himantoglossum caprinum	1	.	.	.	.	.	1	.	.	.	.	.	.	.	.	.	.	.	.
Hippuris vulgaris	1	.	.	.	.	.	1	.	.	.	.	.	.	.	.	.	.	.	.
Hirschfeldia incana	1	.	.	.	.	.	1	.	.	.	.	.	.	.	.	.	.	.	.
Homogyne alpina	1	.	.	.	.	.	.	.	.	.	.	.	.	.	.	.	.	1	.
Hypericum richeri subsp. grisebachii	1	.	.	.	.	.	1	.	.	.	.	.	.	.	.	.	.	.	.
Hyssopus cretaceus	1	.	.	.	.	.	.	.	.	.	.	.	1	.	.	.	.	.	.
Iberis pinnata	1	.	.	.	.	.	.	1	.	.	.	.	.	.	.	.	.	.	.
Iris halophila	1	.	.	.	.	.	.	.	.	.	.	1	.	.	.	.	.	.	.
Iris reichenbachii	1	.	.	.	.	.	.	.	1	.	.	.	.	.	.	.	.	.	.
Juncus effusus	1	1	.	.	.	.	.	.	.	.	.	.	.	.	.	.	.	.	.
Juncus inflexus	1	.	.	1	.	.	.	.	.	.	.	.	.	.	.	.	.	.	.
Juncus subnodulosus	1	.	.	1	.	.	.	.	.	.	.	.	.	.	.	.	.	.	.
Juncus tenuis	1	1	.	.	.	.	.	.	.	.	.	.	.	.	.	.	.	.	.
Klasea erucifolia	1	.	.	.	.	.	.	.	.	.	.	1	.	.	.	.	.	.	.
Knautia fleischmanii	1	.	.	.	1	.	.	.	.	.	.	.	.	.	.	.	.	.	.
Koeleria talievii	1	.	.	.	.	.	.	.	.	.	1	.	.	.	.	.	.	.	.

Group number		B1	B2	B3	B4	B5	B6	B7	F1	F2	F3	F4	S1	S2	S3	S4	S5	S6	S7
Twinspan cluster level 4		7	7	8	8	9	9	10	10	10	10	10	10	13	13	14	14	15	16
Twinspan cluster level 5-6		0	1	0	1	0	1		0-0	0-1	1-0	1-1		0	1	0	1	0	1
No. of relevés		301	135	994	235	846	765	760	1324	730	1316	177	709	65	690	174	250	192	66
Lactuca tatarica	1	.	.	.	.	.	.	.	.	.	1	.	.	.	.	.	.	.	.
Lamium garganicum	1	.	.	.	.	.	.	.	.	.	.	.	.	.	.	.	.	.	2
Laphangium luteoalbum	1	.	.	1	.	.	.	.	.	.	.	.	.	.	.	.	.	.	.
Lappula heteracantha	1	.	.	.	.	.	.	.	1	.	.	.	.	.	.	.	.	.	.
Laser trilobum	1	.	.	.	.	.	1	.	.	.	.	.	.	.	.	.	.	.	.
Laserpitium prutenicum	1	.	.	1	.	.	.	.	.	.	.	.	.	.	.	.	.	.	.
Lathyrus heterophyllus	1	.	.	.	.	.	.	.	.	1	.	.	.	.	.	.	.	.	.
Lens nigricans	1	.	.	.	.	.	1	.	.	.	.	.	.	.	.	.	.	.	.
Ligusticum mutellina	1	.	.	.	.	.	.	.	.	.	.	.	.	.	.	.	.	.	.
Linaria dulcis	1	.	.	.	.	.	.	.	.	.	1	.	.	.	.	.	.	.	.
Linum trigynum	1	.	.	1	.	.	.	.	.	.	.	.	.	.	.	.	.	.	.
Lotus pedunculatus	1	.	.	1	.	.	.	.	.	.	.	.	.	.	.	.	.	.	.
Lunaria rediviva	1	.	.	1	.	.	.	.	.	.	.	.	.	.	.	.	.	.	.
Lupinus polyphyllus	1	.	.	.	.	.	.	.	1	.	.	.	.	.	.	.	.	.	.
Luzula sylvatica	1	.	.	.	.	.	.	.	.	.	.	.	.	.	.	.	.	1	.
Lycopsis arvensis	1	.	.	.	.	.	.	.	.	.	.	.	1	.	.	.	.	.	.
Lythrum hyssopifolia	1	.	.	.	.	.	.	1	.	.	.	.	.	.	.	.	.	.	.
Malva neglecta	1	.	.	.	.	.	.	.	.	.	1	.	.	.	.	.	.	.	.
Malva sylvestris	1	.	.	.	.	.	1	.	.	.	.	.	.	.	.	.	.	.	.
Matthiola fragrans	1	.	.	.	.	.	.	.	.	.	.	.	1	.	.	.	.	.	.
Medicago arabica	1	1	.	.	.	.	.	.	.	.	.	.	.	.	.	.	.	.	.
Melampyrum alboffianum	1	.	.	.	.	.	.	.	.	.	1	.	.	.	.	.	.	.	.
Mentha spicata	1	.	.	.	.	.	.	1	.	.	.	.	.	.	.	.	.	.	.
Mentha x dumetorum	1	.	.	.	.	.	.	.	.	.	1	.	.	.	.	.	.	.	.
Milium effusum	1	.	.	.	.	.	.	.	.	.	.	.	1	.	.	.	.	.	.
Milium vernale	1	.	.	.	.	.	.	1	.	.	.	.	.	.	.	.	.	.	.
Minuartia viscosa	1	.	.	.	.	.	.	.	.	.	.	.	1	.	.	.	.	.	.
Misopates orontium	1	.	.	.	.	.	.	.	1	.	.	.	.	.	.	.	.	.	.
Myosotis alpestris	1	.	.	.	.	.	.	.	.	.	.	.	.	.	.	.	.	.	2
Narcissus poeticus	1	.	.	.	.	.	.	1	.	.	.	.	.	.	.	.	.	.	.
Noccaea goesingensis	1	1	.	.	.	.	.	.	.	.	.	.	.	.	.	.	.	.	.
Noccaea kovatsii	1	.	.	.	.	.	.	1	.	.	.	.	.	.	.	.	.	.	.
Noccaea macrantha	1	.	.	.	.	.	.	.	.	1	.	.	.	.	.	.	.	.	.
Nonea atra	1	.	.	.	.	.	.	.	.	1	.	.	.	.	.	.	.	.	.
Onosma helvetica	1	.	.	.	.	.	.	.	.	1	.	.	.	.	.	.	.	.	.
Ornithogalum fischerianum	1	.	.	.	.	.	.	.	.	.	1	.	.	.	.	.	.	.	.
Orobanche cumana	1	.	.	.	.	.	.	.	.	.	1	.	.	.	.	.	.	.	.
Orobanche lanuginosa	1	.	.	.	.	.	.	1	.	.	.	.	.	.	.	.	.	.	.
Orobanche laserpitii-sileris	1	.	.	1	.	.	.	.	.	.	.	.	.	.	.	.	.	.	.
Orobanche minor	1	.	.	.	.	.	.	.	1	.	.	.	.	.	.	.	.	.	.
Panicum capillare	1	.	.	.	.	.	.	.	1	.	.	.	.	.	.	.	.	.	.

Group number		B1	B2	B3	B4	B5	B6	B7	F1	F2	F3	F4	S1	S2	S3	S4	S5	S6	S7
Twinspan cluster level 4		7	7	8	8	9	9	10	10	10	10	10	10	13	13	14	14	15	16
Twinspan cluster level 5-6		0	1	0	1	0	1		0-0	0-1	1-0	1-1		0	1	0	1	0	1
No. of relevés		301	135	994	235	846	765	760	1324	730	1316	177	709	65	690	174	250	192	66
Pedicularis elongata subsp. julica	1	.	.	.	1	.	.	.	.	.	.	.	.	.	.	.	.	.	.
Pedicularis hacquetii	1	.	.	1	.	.	.	.	.	.	.	.	.	.	.	.	.	.	.
Pedicularis verticillata	1	.	.	.	.	.	.	.	.	.	.	.	.	.	.	.	.	1	.
Petasites hybridus	1	.	.	.	.	.	.	.	.	.	.	.	.	.	.	.	.	1	.
Petasites kablikianus	1	.	.	.	.	.	.	.	.	.	.	.	.	.	.	.	.	1	.
Phleum rhaeticum	1	.	.	.	.	.	.	.	.	.	.	.	.	.	.	.	.	.	2
Physalis alkekengi	1	.	.	.	.	.	.	1	.	.	.	.	.	.	.	.	.	.	.
Pilosella densiflora	1	.	.	1	.	.	.	.	.	.	.	.	.	.	.	.	.	.	.
Pilosella rothiana	1	.	.	.	.	.	.	.	1	.	.	.	.	.	.	.	.	.	.
Pinguicula vulgaris	1	.	.	.	.	.	.	.	.	.	.	.	.	.	.	.	.	1	.
Pisum sativum subsp. elatius	1	.	.	.	.	.	.	.	1	.	.	.	.	.	.	.	.	.	.
Plantago schwarzenbergiana	1	.	.	.	.	.	.	.	.	.	1	.	.	.	.	.	.	.	.
Poa rehmannii	1	.	.	.	.	.	.	.	.	.	.	.	.	.	.	.	.	.	2
Podospermum roseum	1	.	.	.	.	.	1	.	.	.	.	.	.	.	.	.	.	.	.
Polygonatum hirtum	1	.	.	.	.	.	.	.	.	.	.	.	.	.	.	.	.	1	.
Polygonum hydropiper	1	.	.	.	.	.	.	.	.	.	1	.	.	.	.	.	.	.	.
Polygonum lapathifolium	1	.	.	.	.	.	.	.	.	.	1	.	.	.	.	.	.	.	.
Polygonum mite	1	.	.	.	.	.	.	.	.	.	1	.	.	.	.	.	.	.	.
Polygonum viviparum	1	.	.	1	.	.	.	.	.	.	.	.	.	.	.	.	.	.	.
Potentilla supina	1	.	.	.	.	.	.	.	.	.	1	.	.	.	.	.	.	.	.
Primula farinosa	1	.	.	1	.	.	.	.	.	.	.	.	.	.	.	.	.	.	.
Pulmonaria dacica	1	.	.	.	.	.	.	.	1	.	.	.	.	.	.	.	.	.	.
Pulsatilla taurica	1	.	.	.	.	.	.	.	.	.	.	.	.	.	.	.	.	.	.
Ranunculus arvensis	1	.	1	.	.	.	.	.	.	.	.	.	.	.	.	.	.	.	.
Ranunculus flammula	1	.	.	1	.	.	.	.	.	.	.	.	.	.	.	.	.	.	.
Ranunculus thora	1	.	.	.	1	.	.	.	.	.	.	.	.	.	.	.	.	.	.
Rumex sanguineus	1	.	.	.	.	.	.	.	.	.	1	.	.	.	.	.	.	.	.
Sagina nodosa	1	.	.	.	.	.	.	.	.	.	1	.	.	.	.	.	.	.	.
Sagina procumbens	1	.	.	.	.	.	.	.	.	.	1	.	.	.	.	.	.	.	.
Salvia scabiosifolia	1	.	.	.	.	.	.	.	.	.	.	.	.	.	.	.	.	.	.
Salvia sclarea	1	.	.	.	.	.	.	.	.	.	1	.	.	.	.	.	.	.	.
Schoenus nigricans	1	.	.	1	.	.	.	.	.	.	.	.	.	.	.	.	.	.	.
Scrophularia canina	1	.	.	.	.	.	.	.	.	.	1	.	.	.	.	.	.	.	.
Scrophularia heterophylla subsp. lacir	1	.	.	.	.	.	.	.	.	.	.	.	.	.	.	.	.	.	2
Scutellaria altissima	1	.	.	.	.	.	.	.	1	.	.	.	.	.	.	.	.	.	.
Scutellaria galericulata	1	.	.	.	.	.	.	1	.	.	.	.	.	.	.	.	.	.	.
Sedum borissovae	1	.	.	.	.	.	.	.	.	.	1	.	.	.	.	.	.	.	.
Selaginella selaginoides	1	.	.	.	.	.	.	.	.	.	.	.	.	.	.	.	.	1	.
Senecio aquaticus	1	.	.	1	.	.	.	.	.	.	.	.	.	.	.	.	.	.	.
Setaria verticillata	1	.	.	.	.	.	.	.	.	.	1	.	.	.	.	.	.	.	.
Silene subconica	1	.	.	.	.	.	.	.	1	.	.	.	.	.	.	.	.	.	.

Group number		B1	B2	B3	B4	B5	B6	B7	F1	F2	F3	F4	S1	S2	S3	S4	S5	S6	S7
Twinspan cluster level 4		7	7	8	8	9	9	10	10	10	10	10	10	13	13	14	14	15	16
Twinspan cluster level 5-6		0	1	0	1	0	1		0-0	0-1	1-0	1-1		0	1	0	1	0	1
No. of relevés		301	135	994	235	846	765	760	1324	730	1316	177	709	65	690	174	250	192	66
Sisymbrium officinale	1	.	.	.	.	.	.	.	1	.	.	.	.	.	.	.	.	.	.
Sisyrinchium montanum	1	.	.	1	.	.	.	.	.	.	.	.	.	.	.	.	.	.	.
Sisyrinchium septentrionale	1	.	.	.	.	.	1	.	.	.	.	.	.	.	.	.	.	.	.
Spergularia marina	1	.	.	.	.	.	.	.	.	1	.	.	.	.	.	.	.	.	.
Stachys arvensis	1	.	.	.	.	.	.	.	.	.	1	.	.	.	.	.	.	.	.
Stellaria hippoctona	1	.	.	.	.	.	.	1	.	.	.	.	.	.	.	.	.	.	.
Stipa anomala	1	.	.	.	.	.	.	1	.	.	.	.	.	.	.	.	.	.	.
Stipa zalesskii	1	.	.	.	.	.	.	.	.	.	1	.	.	.	.	.	.	.	.
Taraxacum bessarabicum	1	.	.	.	.	.	.	.	.	1	.	.	.	.	.	.	.	.	.
Taraxacum sect. Palustria	1	.	.	1	.	.	.	.	.	.	.	.	.	.	.	.	.	.	.
Tephroseris besseriana	1	.	.	.	.	.	1	.	.	.	.	.	.	.	.	.	.	.	.
Tephroseris crispa	1	.	.	.	.	1	.	.	.	.	.	.	.	.	.	.	.	.	.
Tephroseris papposa	1	.	.	.	.	.	.	.	.	.	.	.	.	.	.	.	.	.	2
Thymus moldavicus	1	.	.	.	.	.	.	.	.	1	.	.	.	.	.	.	.	.	.
Tragopogon dasyrhynchus	1	.	.	.	.	.	.	.	.	.	1	.	.	.	.	.	.	.	.
Tragopogon floccosus	1	.	.	.	.	.	.	.	1	.	.	.	.	.	.	.	.	.	.
Tribulus terrestris	1	.	.	.	.	.	.	.	.	.	1	.	.	.	.	.	.	.	.
Trichophorum alpinum	1	.	.	1	.	.	.	.	.	.	.	.	.	.	.	.	.	.	.
Trifolium alpinum	1	.	.	.	.	.	.	.	1	.	.	.	.	.	.	.	.	.	.
Trifolium purpureum	1	.	.	.	.	1	.	.	.	.	.	.	.	.	.	.	.	.	.
Trifolium retusum	1	.	.	.	.	.	.	.	.	.	1	.	.	.	.	.	.	.	.
Trifolium strictum	1	.	.	.	.	.	.	.	.	.	1	.	.	.	.	.	.	.	.
Triglochin maritima	1	.	.	.	.	.	.	.	.	.	1	.	.	.	.	.	.	.	.
Valeriana dioica	1	.	.	1	.	.	.	.	.	.	.	.	.	.	.	.	.	.	.
Valeriana tuberosa	1	.	.	.	.	.	.	1	.	.	.	.	.	.	.	.	.	.	.
Valerianella costata	1	.	.	.	.	.	.	.	1	.	.	.	.	.	.	.	.	.	.
Veronica capsellicarpa	1	.	.	.	.	.	.	.	.	.	1	.	.	.	.	.	.	.	.
Veronica paniculata	1	.	.	.	.	.	.	1	.	.	.	.	.	.	.	.	.	.	.
Veronica peregrina	1	.	.	.	.	.	.	1	.	.	.	.	.	.	.	.	.	.	.
Vinca minor	1	.	.	.	.	.	.	.	.	.	1	.	.	.	.	.	.	.	.
Viola alba	1	.	.	.	.	.	.	.	.	.	.	.	.	.	1	.	.	.	.
Viola cretacea	1	.	.	.	.	.	.	.	.	.	1	.	.	.	.	.	.	.	.
Viola elatior	1	.	.	1	.	.	.	.	.	.	.	.	.	.	.	.	.	.	.
Viola lutea	1	.	.	1	.	.	.	.	.	.	.	.	.	.	.	.	.	.	.

Group number		B1	B2	B3	B4	B5	B6	B7	F1	F2	F3	F4	S1	S2	S3	S4	S5	S6	S7
Twinspan cluster level 4		7	7	8	8	9	9	10	10	10	10	10	10	13	13	14	14	15	16
Twinspan cluster level 5-6		0	1	0	1	0	1		0-0	0-1	1-0	1-1		0	1	0	1	0	1
No. of relevés		301	135	994	235	846	765	760	1324	730	1316	177	709	65	690	174	250	192	66
Number of relevés with bryophytes and lichens recorded		78	78	377	15	302	181	238	555	145	201	5	263	15	131	55	4	89	2
**Bryophytes**																			
Plagiomnium affine agg.	131	8	4	21	.	5	8	4	1	.	.	.	.	.	1	.	.	.	.
Fissidens taxifolius	118	5	1	8	.	12	13	3	1	2	.	.	.	.	.	.	.	1	.
Ceratodon purpureus	319	9	3	3	.	8	2	15	23	8	1	20	25	20	8	4	.	.	.
Tortella tortuosa	250	.	1	2	.	7	1	3	4	5	1	.	9	.	57	36	50	56	.
Ditrichum flexicaule	110	.	.	1	.	1	1	1	1	.	.	.	5	.	21	24	50	38	.
Homalothecium philippeanum	41	.	.	.	20	.	.	.	.	.	.	.	1	.	13	.	.	19	.
Encalypta streptocarpa	58	.	.	1	.	1	.	3	1	.	.	.	3	7	11	4	25	12	.
Ctenidium molluscum	31	.	.	1	.	1	1	.	.	.	.	.	.	.	2	.	.	19	100
Abietinella abietina	813	24	29	31	47	41	35	34	26	32	19	20	24	.	24	7	25	11	.
Hypnum cupressiforme	394	6	.	9	.	9	6	19	25	6	1	.	25	40	18	9	.	12	.
Homalothecium lutescens	382	17	1	16	7	20	30	14	10	16	12	.	8	.	8	.	.	3	.
Rhytidium rugosum	279	4	.	4	53	15	16	5	8	3	.	.	19	.	25	2	.	27	.
Syntrichia ruralis agg.	259	6	.	1	.	3	1	6	18	17	4	60	21	7	16	15	.	4	.
Weissia spec. div.	250	4	.	2	.	4	8	8	7	43	18	80	10	.	5	9	50	.	.
Bryum caespiticium	155	1	1	5	.	10	1	9	7	10	3	.	6	.	2	.	50	.	.
Oxyrhynchium hians	137	12	1	7	.	8	11	5	1	8	9	.	2	.	.	.	.	.	.
Polytrichum piliferum	135	1	.	1	.	.	.	5	11	.	3	.	16	27	4	2	.	.	.
Fissidens dubius	121	.	.	10	.	9	14	1	1	.	.	.	1	.	2	2	.	17	.
Bryum argenteum	120	1	.	1	.	6	.	1	7	3	3	.	9	.	14	2	.	2	.
Grimmia pulvinata	117	.	.	.	.	.	.	2	9	5	1	20	14	.	8	7	25	.	.
Tortella inclinata	97	.	.	.	7	3	1	1	4	3	.	.	9	.	8	29	50	4	.
Brachythecium albicans	93	8	.	5	.	3	1	3	7	1	4	.	1	.	.	.	.	.	.
Rhytidiadelphus triquetrus	73	1	.	11	7	3	2	.	.	.	.	.	.	.	4	.	.	11	.
Racomitrium canescens agg.	72	1	.	1	.	1	.	3	6	.	.	.	8	13	2	.	.	2	.
Bryum capillare	70	1	.	1	.	2	1	1	5	7	6	20	2	.	.	.	.	3	.
Thuidium assimile	67	5	.	11	.	5	1	1	1	.	.	.	.	.	.	.	.	2	.
Brachythecium rutabulum	59	15	.	5	.	1	7	2	1	.	1	.	1	.	.	.	.	.	.
Schistidium apocarpum agg.	59	.	.	1	.	1	.	1	2	4	1	.	3	.	8	2	.	13	.
Pleurochaete squarrosa	57	.	.	.	7	1	1	1	3	4	.	.	8	.	2	4	25	.	.
Homalothecium sericeum	55	.	.	1	.	1	.	.	2	1	.	.	4	7	6	.	.	10	.
Campyliadelphus chrysophyllus	48	.	.	5	.	3	4	1	1	1	1	.	.	.	.	.	25	2	.
Pleurozium schreberi	46	1	.	2	20	1	.	2	1	.	.	.	3	.	6	2	.	1	.
Hylocomium splendens	46	3	.	3	20	2	1	1	1	.	.	.	1	.	2	.	.	13	.
Plagiomnium cuspidatum	42	8	6	6	.	1	1	1	1	.	.	.	.	.	1	.	.	.	.
Pseudoscleropodium purum	42	9	.	6	20	1	.	1	1	.	.	.	1	.	.	.	.	1	.
Encalypta vulgaris	41	.	.	1	.	1	.	1	1	.	.	.	4	7	5	.	.	10	.
Plagiomnium undulatum	39	5	1	5	13	1	2	1	1	.	.	.	.	.	.	.	.	4	.

Group number		B1	B2	B3	B4	B5	B6	B7	F1	F2	F3	F4	S1	S2	S3	S4	S5	S6	S7
Twinspan cluster level 4		7	7	8	8	9	9	10	10	10	10	10	10	13	13	14	14	15	16
Twinspan cluster level 5-6		0	1	0	1	0	1		0-0	0-1	1-0	1-1		0	1	0	1	0	1
No. of relevés		301	135	994	235	846	765	760	1324	730	1316	177	709	65	690	174	250	192	66
Thuidium delicatulum	38	5	.	5	.	2	2	.	.	.	.	.	.	.	2	.	.	3	.
Orthotrichum anomalum	38	.	.	.	.	.	.	.	1	2	.	.	5	.	10	.	.	4	.
Barbula unguiculata	37	5	.	1	.	2	2	1	1	1	2	.	1	.	1	.	.	.	.
Rhytidiadelphus squarrosus	33	1	.	6	7	1	1	1	.	.	.	.	.	.	.	.	.	1	.
Barbula convoluta	32	3	.	1	.	2	1	1	1	.	.	.	3	.	2	.	.	1	.
Polytrichum juniperinum	30	.	.	1	.	.	1	2	2	.	1	.	2	.	.	2	.	1	.
Mannia fragrans	28	.	.	.	.	.	.	.	3	.	.	.	2	7	3	2	.	.	.
Brachythecium glareosum	28	4	1	1	.	1	1	1	1	1	1	.	.	.	.	.	.	.	.
Campylophyllum calcareum	24	.	3	2	.	1	.	1	1	.	1	.	1	.	.	.	.	.	.
Brachythecium salebrosum	24	1	.	4	.	1	.	1	1	.	1	.	.	.	.	.	.	.	.
Amblystegium serpens	22	1	3	2	.	1	2	2	.	1	.	.	.	.	.	.	.	.	.
Riccia ciliata	21	.	.	.	.	.	.	.	3	.	.	.	2	.	.	.	.	.	.
Calliergonella cuspidata	21	1	.	4	.	1	1	1	.	.	.	.	1	.	.	.	.	.	.
Cirriphyllum piliferum	21	.	.	5	.	1	.	.	.	.	.	.	.	.	.	.	.	.	.
Syntrichia montana	19	.	.	1	.	.	.	.	3	.	.	.	1	.	1	.	.	2	.
Entodon concinnus	18	.	.	1	7	3	1	.	.	.	.	.	.	.	1	.	25	1	.
Tortula muralis	17	.	.	.	.	.	1	1	2	1	.	.	1	.	.	.	.	2	.
Didymodon acutus	15	.	.	.	.	1	1	1	1	1	.	.	1	.	.	2	.	.	.
Brachythecium campestre	15	.	10	1	.	.	.	3	.	.	.	.	.	.	.	.	.	.	.
Didymodon fallax	14	.	.	.	.	1	3	1	1	1	.	.	1	.	.	.	.	.	.
Phascum cuspidatum	13	1	.	.	.	1	.	1	1	2	.	.	.	.	.	.	.	.	.
Riccia ciliifera	13	.	.	.	.	.	.	.	1	.	.	.	4	.	.	2	.	.	.
Dicranum scoparium	13	.	.	.	7	1	.	1	.	.	.	.	1	.	3	.	.	3	.
Brachytheciastrum velutinum	13	.	.	1	.	1	3	1	.	1	.	.	.	.	.	.	.	.	.
Rhynchostegium megapolitanum	13	.	.	.	.	1	.	1	1	1	1	.	.	.	.	.	.	.	.
Orthotrichum cupulatum	13	.	.	.	.	.	.	1	1	1	.	.	1	.	1	.	.	1	.
Hypnum vaucheri	10	.	.	.	.	.	.	1	.	.	.	.	.	.	1	2	.	8	.
Climacium dendroides	10	.	.	2	.	1	.	1	.	.	.	.	.	.	.	.	.	.	.
Campylophyllum sommerfeltii	10	.	.	1	.	1	1	1	.	1	.	.	1	.	1	.	.	.	.
Leskea polycarpa	10	.	.	1	.	.	.	.	.	.	.	.	.	.	5	.	.	3	.
Distichium capillaceum	10	.	.	.	.	1	.	.	.	.	.	.	1	7	.	.	.	7	.
Oxyrrhynchium schleicheri	9	.	.	1	.	.	.	1	1	1	.	.	1	.	.	.	.	.	.
Leucodon sciuroides	9	.	.	.	.	.	.	.	1	.	.	.	2	.	2	.	.	.	.
Rhodobryum roseum	8	3	.	1	.	1	.	.	.	.	1	.	.	.	.	.	.	.	.
Atrichum undulatum	8	.	.	1	.	1	1	.	.	.	.	.	.	.	.	.	.	.	.
Neckera crispa	8	.	.	.	.	.	.	.	.	.	.	.	.	.	.	.	.	9	.
Anomodon viticulosus	8	.	.	.	.	.	.	.	1	.	.	.	1	.	3	.	.	1	.
Oxymitra incrassata	7	.	.	.	.	.	.	.	.	.	.	.	2	.	.	2	.	.	.
Rhizomnium punctatum	7	.	.	1	.	1	.	.	.	.	.	.	.	.	.	.	.	.	.
Bryum rubens	7	.	.	.	.	1	.	.	1	1	1	.	.	.	.	.	.	.	.
Pseudoleskea catenulata	7	.	.	.	.	.	.	1	.	.	.	.	1	.	1	.	.	1	.

Group number		B1	B2	B3	B4	B5	B6	B7	F1	F2	F3	F4	S1	S2	S3	S4	S5	S6	S7
Twinspan cluster level 4		7	7	8	8	9	9	10	10	10	10	10	10	13	13	14	14	15	16
Twinspan cluster level 5-6		0	1	0	1	0	1		0-0	0-1	1-0	1-1		0	1	0	1	0	1
No. of relevés		301	135	994	235	846	765	760	1324	730	1316	177	709	65	690	174	250	192	66
Dicranum polysetum	6	.	.	.	.	1	.	.	.	.	.	.	2	.	1	.	.	.	.
Grimmia anodon	6	.	.	.	.	.	.	.	1	1	.	.	1	.	.	.	.	.	.
Hedwigia ciliata agg.	6	.	.	.	.	.	.	.	1	.	.	.	1	7	1	.	.	.	.
Schistidium brunnescens	6	.	.	.	.	.	.	.	.	1	.	.	2	.	1	.	.	.	.
Eurhynchiastrum pulchellum	6	.	.	.	.	1	1	.	1	.	.	.	.	.	.	.	.	.	.
Tortula lanceolata	6	.	.	.	.	1	.	1	1	1	.	.	1	.	.	.	.	.	.
Thuidium tamariscinum	6	1	.	1	.	.	.	.	.	.	.	.	1	.	.	.	.	.	.
Plagiochila asplenioides	6	.	.	1	.	.	.	.	.	.	.	.	.	.	.	.	.	6	.
Entosthodon fascicularis	6	3	.	.	.	.	.	.	1	.	.	.	.	.	.	.	.	.	.
Pleuridium subulatum	6	1	.	.	.	.	.	1	1	.	.	.	.	.	.	.	.	.	.
Campylium stellatum	6	.	.	1	.	1	.	.	.	.	.	.	.	.	.	.	.	.	.
Lecanora campestris	6	.	.	.	.	.	.	.	.	.	.	.	2	.	.	.	.	.	.
Physcomitrium pyriforme	5	1	.	.	.	.	.	1	1	.	1	.	.	.	.	.	.	.	.
Rhodobryum ontariense	5	.	.	1	.	.	1	.	.	.	.	.	.	.	.	.	.	.	.
Lophocolea bidentata	5	.	.	1	.	.	.	1	.	.	.	.	.	.	.	.	.	.	.
Protobryum bryoides	5	.	.	.	.	.	.	1	.	1	1	.	.	.	.	.	.	.	.
Grimmia orbicularis	5	.	.	.	.	.	.	.	.	.	.	.	1	.	.	7	.	.	.
Didymodon cordatus	5	.	.	.	.	1	.	.	.	.	.	.	1	.	.	.	25	.	.
Dicranella heteromalla	5	.	.	.	.	1	1	.	.	.	.	.	1	.	1	.	.	1	.
Trichostomum crispulum	5	.	.	.	.	.	1	.	1	.	.	.	.	.	.	2	.	2	.
Pterygoneurum ovatum	5	.	.	.	.	1	1	1	1	.	.	.	.	.	.	.	.	.	.
Fissidens viridulus	5	4	.	.	.	.	.	1	1	.	.	.	.	.	.	.	.	.	.
Tortella fragilis	5	.	.	.	.	.	.	.	1	.	.	.	1	.	.	.	.	.	.
Fissidens bryoides	4	3	.	.	.	.	.	.	1	.	.	.	.	.	.	.	.	.	.
Eurhynchium striatum	4	.	.	.	.	1	.	.	.	.	.	.	.	.	.	.	.	.	.
Bryoerythrophyllum recurvirostrum	4	.	.	.	.	1	.	.	.	.	.	.	.	.	1	.	.	1	.
Microbryum davallianum	4	.	.	.	.	.	.	.	.	.	2	.	.	.	.	.	.	.	.
Pterygoneurum subsessile	4	.	.	.	.	1	.	.	1	1	.	.	.	.	.	.	.	.	.
Bryum archangelicum	4	.	.	.	.	1	.	.	1	1	.	.	.	.	.	.	.	.	.
Bryum atrovirens agg.	4	3	.	.	.	.	.	.	1	.	.	.	.	.	.	.	.	.	.
Pleuridium acuminatum	4	.	.	.	.	.	1	.	1	.	.	.	.	.	.	.	.	.	.
Bryum kunzei	4	.	.	.	.	.	.	.	.	2	1	.	.	.	.	.	.	.	.
Fissidens adianthoides	3	.	.	1	.	.	.	.	.	.	.	.	.	.	.	.	.	.	.
Tortula modica	3	.	.	.	.	1	.	1	1	.	.	.	.	.	.	.	.	.	.
Cephaloziella divaricata	2	.	.	.	.	.	.	1	.	.	.	.	1	.	.	.	.	.	.
Tortula truncata	2	.	.	1	.	.	.	.	1	.	.	.	.	.	.	.	.	.	.
Thuidium recognitum	1	.	.	.	.	.	.	.	.	.	1	.	.	.	.	.	.	.	.
Polytrichastrum formosum	1	.	.	.	.	.	.	.	.	.	.	.	.	.	.	.	.	.	.
Pohlia nutans	1	.	.	.	.	.	.	.	.	.	.	.	1	.	.	.	.	.	.
Leptodictyum riparium	1	.	.	1	.	.	.	.	.	.	.	.	.	.	.	.	.	.	.
Calliergonella lindbergii	1	.	.	.	.	.	.	.	.	.	.	.	.	.	.	.	.	.	.

Group number		B1	B2	B3	B4	B5	B6	B7	F1	F2	F3	F4	S1	S2	S3	S4	S5	S6	S7
Twinspan cluster level 4		7	7	8	8	9	9	10	10	10	10	10	10	13	13	14	14	15	16
Twinspan cluster level 5-6		0	1	0	1	0	1		0-0	0-1	1-0	1-1		0	1	0	1	0	1
No. of relevés		301	135	994	235	846	765	760	1324	730	1316	177	709	65	690	174	250	192	66
Drepanocladus longifolius	1	.	.	1	.	.	.	.	.	.	.	.	.	.	.	.	.	.	.
Entodon schleicheri	1	.	.	.	.	.	.	.	.	.	.	.	.	.	.	.	.	.	.
Pogonatum urnigerum	1	.	.	.	.	.	.	.	1	.	.	.	.	.	.	.	.	.	.
**Lichens**																			
Cladonia rangiformis	187	4	.	1	.	1	1	11	16	.	.	.	19	7	7	5	.	.	.
Cladonia pyxidata	132	3	1	1	.	2	.	4	6	.	.	.	17	13	14	4	.	12	.
Cladonia foliacea	127	.	1	1	.	1	1	5	11	.	.	.	14	.	6	5	.	.	.
Cladonia symphycarpia	104	.	.	1	.	1	.	2	6	1	.	.	13	.	11	4	.	4	.
Cladonia furcata agg.	94	.	1	1	.	3	.	4	8	1	.	.	7	.	3	5	.	.	.
Cladonia pocillum	74	1	.	1	.	2	.	3	2	.	.	.	11	.	5	5	.	3	.
Xanthoparmelia stenophylla	64	.	.	.	.	.	.	1	3	.	.	.	14	20	5	4	.	.	.
Cladonia fimbriata	60	.	.	1	.	1	.	3	5	.	.	.	5	.	5	4	.	3	.
Collema spec. div.	52	.	.	1	.	.	.	.	1	19	2	40	2	.	1	2	.	3	.
Peltigera rufescens	51	.	.	1	.	1	.	2	3	.	.	.	5	.	5	7	25	2	.
Xanthoparmelia pulla	48	.	.	.	.	.	.	.	3	.	.	.	8	20	3	4	.	.	.
Cladonia convoluta	42	.	.	.	.	.	.	.	3	4	.	.	5	.	2	7	25	.	.
Cladonia rangiferina	42	.	.	.	7	1	.	2	1	.	.	.	8	.	5	5	.	.	.
Cladonia arbuscula agg.	33	.	.	.	.	1	.	2	2	.	.	.	5	.	3	.	.	.	.
Cetraria aculeata	31	.	.	.	.	1	.	1	1	.	.	.	8	.	2	2	.	.	.
Protoparmeliopsis muralis	29	.	.	.	.	1	.	.	2	.	.	.	6	7	.	.	.	.	.
Xanthoparmelia conspersa	28	.	.	.	.	.	.	1	3	1	.	.	3	13	.	.	.	.	.
Cladonia coniocraea	26	.	.	.	.	1	.	1	3	.	.	.	2	.	3	2	.	.	.
Cladonia glauca	24	1	1	1	.	2	.	.	1	.	.	.	3	.	.	.	.	.	.
Toninia sedifolia	23	.	.	.	.	1	.	.	1	1	.	.	4	.	2	5	.	1	.
Cladonia chlorophaea	22	.	.	1	.	1	1	1	2	.	.	.	.	7	1	4	.	3	.
Squamarina cartilaginea	19	.	.	.	.	.	.	.	1	.	.	.	1	.	5	13	.	.	.
Psora decipiens	17	.	.	.	.	.	.	.	1	.	.	.	1	.	.	20	.	2	.
Cladonia subulata	16	1	.	.	.	.	1	1	2	.	.	.	1	13	1	.	.	.	.
Diploschistes muscorum	14	.	1	1	.	1	.	.	1	.	.	.	1	.	.	.	.	.	.
Solorina saccata	12	.	.	.	.	.	.	.	.	.	.	.	.	.	.	.	.	13	.
Cetraria islandica	12	.	.	.	.	.	.	1	.	.	.	.	4	.	.	.	.	.	.
Verrucaria muralis	11	.	1	1	.	2	.	.	.	.	.	.	1	.	.	2	.	.	.
Stereocaulon incrustatum	9	.	.	.	.	.	.	1	1	.	.	.	1	.	.	.	.	.	.
Bilimbia sabuletorum	9	.	.	1	.	1	.	.	.	.	.	.	1	.	1	.	.	.	.
Candelariella vitellina	9	.	.	.	.	.	.	1	1	.	.	.	2	7	.	.	.	.	.
Verrucaria nigrescens	8	.	1	.	.	1	.	.	1	.	.	.	.	.	2	.	.	.	.
Physcia wainioi	6	.	.	.	.	.	.	.	1	.	.	.	1	.	.	.	.	.	.
Lecanora dispersa	6	.	1	1	.	1	.	.	.	.	.	.	.	.	.	.	.	.	.
Cladonia magyarica	6	.	.	.	.	.	.	.	1	1	.	.	1	.	.	2	.	.	.
Fulgensia fulgens	6	.	.	.	.	.	.	.	1	.	.	.	.	.	1	7	.	.	.

Group number		B1	B2	B3	B4	B5	B6	B7	F1	F2	F3	F4	S1	S2	S3	S4	S5	S6	S7
Twinspan cluster level 4		7	7	8	8	9	9	10	10	10	10	10	10	13	13	14	14	15	16
Twinspan cluster level 5-6		0	1	0	1	0	1		0-0	0-1	1-0	1-1		0	1	0	1	0	1
No. of relevés		301	135	994	235	846	765	760	1324	730	1316	177	709	65	690	174	250	192	66
Leptogium lichenoides	6	.	.	.	.	.	.	.	1	.	.	.	1	.	.	.	.	1	.
Cladonia uncialis	5	.	.	.	.	.	.	.	1	.	.	.	1	.	.	.	.	.	.
Cladonia polycarpoides	5	.	.	.	.	.	.	.	1	.	.	.	.	.	.	4	.	.	.
Rhizocarpon geographicum	5	.	.	.	.	.	.	.	1	.	.	.	1	7	.	.	.	.	.
Cladonia squamosa	5	.	.	.	.	.	.	.	1	.	.	.	.	.	.	.	.	1	.
Verrucaria aethiobola	5	.	1	1	.	1	.	.	.	.	.	.	1	.	.	.	.	.	.
Physcia dubia	5	.	.	.	.	.	.	.	.	.	.	.	1	7	1	.	.	.	.
Lobothallia radiosa	5	.	.	.	.	.	.	1	1	.	.	.	1	.	.	.	.	.	.
Parmelia saxatilis	5	.	.	.	.	.	.	.	1	.	.	.	.	7	1	.	.	.	.
Cladonia verticillata	4	.	.	.	.	.	.	.	.	.	.	.	1	.	.	2	.	.	.
Lecidea fuscoatra	4	.	.	.	.	.	.	.	1	.	.	.	1	7	.	.	.	.	.
Mycobilimbia lurida	4	.	.	.	.	.	.	.	1	.	.	.	.	.	1	.	.	.	.
Candelariella aurella	4	.	.	.	.	1	.	.	.	.	.	.	.	.	1	.	.	.	.
Cladonia phyllophora	4	.	.	.	.	.	.	1	1	.	.	.	1	.	.	.	.	.	.
Ramalina capitata	4	.	.	.	.	.	.	.	1	.	.	.	1	7	1	.	.	.	.
Xanthoparmelia protomatrae	4	.	.	.	.	.	.	.	1	1	.	.	.	13	.	.	.	.	.
Cladonia rei	3	.	.	.	.	.	.	.	1	.	.	.	1	.	.	.	.	.	.
Peltigera canina	3	.	.	.	.	.	1	.	.	.	.	.	1	.	.	.	.	.	.
Cladonia gracilis	1	.	.	.	.	.	.	1	.	.	.	.	.	.	.	.	.	.	.
