# Supplementary material for: A higher‐level classification of the Pannonian and western Pontic steppe grasslands (Central and Eastern Europe)
Source: Appl Veg Sci. 2016 Sep 16;20(1):143–58. doi: 10.1111/avsc.12265 (PMC5348766; doi:10.1111/avsc.12265)
Supplement: Supplementary file 8 — Appendix S8. Data sources of the Festuco‐Brometea relevés. [file AVSC-20-143-s008.pdf]

Supporting information to the paper

Willner, W. et al. A higher-level classification of the Pannonian and western Pontic steppe grasslands (Central and Eastern Europe). *Applied Vegetation Science.*

Appendix S8. Data sources for the three orders of the *Festuco-Brometea*. "Other sources" are mostly unpublished relevés present in the databases. However, some of them may have been published after their inclusion in the database.

Brometalia erecti

Ambrozek 1989: 28 rel.
Ardelean 1983: 4 rel.
Auer 1982: 1 rel.
Babczyńska 1978: 11 rel.
Babczyńska-Sendek 2005: 126 rel.
Babczyńska-Sendek & Andrzejczuk 1977: 9 rel.
Babić 1965: 3 rel.
Bača 2010: 6 rel.
Badarau et al. 2001: 1 rel.
Badarau et al. 1995: 7 rel.
Balátová-Tuláčková & Hájek 1998: 1 rel.
Baláz 1991: 2 rel.
Bednář & Velísek 1967: 5 rel.
Bernátová et al. 2002: 1 rel.
Blanár 2005: 2 rel.
Blaženčić 1982: 2 rel.
Bosáčková 1972: 2 rel.
Bosáčková 1975: 1 rel.
Bosáčková 1978: 1 rel.
Bosáčková et al. 1974: 3 rel.
Bozková 2005: 5 rel.
Bravencová 2003: 15 rel.
Brzeg 2005: 20 rel.
Bulokhov 2001: 40 rel.
Butorac 1989: 10 rel.
Cachovanová 2010: 27 rel.
Čechová 1999: 2 rel.
Čechová 1999: 2 rel.
Celinski & Babczynska Magiera 1997: 9 rel.
Chlumská 1961: 4 rel.
Chytrý & Vicherek 1996: 1 rel.
Chytrý & Vicherek 2003: 8 rel.
Chytrý et al. 1997: 48 rel.
Cristea & Csűrős 1976: 3 rel.
Csűrős et al. 1961: 23 rel.
Danáková 2000: 3 rel.
Danihelka 1998: 8 rel.
Danihelka & Grulich 2000: 1 rel.
Dengler et al. 2012: 34 rel.
Denk 2000: 25 rel.
Denk 2005: 37 rel.
Didukh & Vashenyak 2012: 13 rel.
Dubová 1978: 5 rel.
Dubová & Unar 1986: 7 rel.
Dúbravková-Michálková et al. 2008: 2 rel.
Dzubinová 1978: 1 rel.
Ebenberger 1993: 1 rel.
Eggler 1959: 1 rel.
Eijsink et al. 1978: 34 rel.
Eliás 1986: 3 rel.
Eliás 1987: 3 rel.
Eliás jun. & Díte 2005: 1 rel.
Eliás ml. et al. 2002: 2 rel.
Fajmon 2006: 3 rel.
Falťan 1999: 5 rel.
Fijałkowski 1958: 11 rel.
Fijałkowski & Izdebski 1957: 35 rel.
Filić 1984: 4 rel.
Fiserová 1991: 3 rel.
Florian 1992: 2 rel.
Frink 2010: 21 rel.
Gawłowska 1958: 1 rel.
Gaži-Baskova et al. 1983: 4 rel.
Głazek 1968: 31 rel.
Głowacki 1984: 4 rel.
Gergely 1970: 6 rel.
Germany 2013: 72 rel.
Ghisa 1941: 4 rel.

Ghisa 1962: 2 rel.
Gogela 1971: 17 rel.
Gomlya 2005: 5 rel.
Grodzińska 1970: 3 rel.
Grodzińska 1979: 3 rel.
Grodzińska & Szarek-Łukaszewska 2009: 10 rel.
Grüll 1953: 2 rel.
Grüll 1960: 2 rel.
Grüll 1989: 10 rel.
Grüll 1991: 1 rel.
Grüll 1984b: 2 rel.
Grüll 1984e: 6 rel.
Grüll 1985a: 9 rel.
Grüll 1985c: 5 rel.
Grüll 1985d: 1 rel.
Grüll 1986a: 1 rel.
Grüll 1986b: 4 rel.
Grüll 1988a: 3 rel.
Grüll 1988b: 4 rel.
Grüll 1988c: 1 rel.
Háberová et al. 1985: 8 rel.
Hadač et al. 1997: 7 rel.
Hadinec & Lustyk 2008: 1 rel.
Hantáková 1991: 13 rel.
Hartman 1958: 10 rel.
Hodisan 1968: 1 rel.
Holubová 1968: 7 rel.
Horáková 1972: 4 rel.
Horvatić & Gospodarić 1960: 3 rel.
Huspeka 1993: 18 rel.
Jarosová & Mucina 1988: 11 rel.
Jongepier & Jongepierová 1993: 1 rel.
Jongepierová & Jongepier 2008: 1 rel.
Jurko 1964: 2 rel.
Jurko 1969: 3 rel.
Jurko 1970: 2 rel.
Jurko 1980: 7 rel.
Jurko 1958a: 1 rel.
Jurko 1971a: 14 rel.
Jurko 1971b: 2 rel.
Kaźmierczakowa 2004: 2 rel.
Kaligarič 1997: 4 rel.
Karrer 1985a: 5 rel.
Klika 1929a: 2 rel.
Klika 1929b: 5 rel.
Klika 1931: 1 rel.
Kliment 1998: 2 rel.
Kliment 2002: 1 rel.
Kliment et al. 2000: 15 rel.
Klimes 1983: 29 rel.
Kochjarová 1997: 2 rel.
Kochjarová 2002: 4 rel.
Kochjarová 2009: 9 rel.
Kochjarová 1998a: 1 rel.
Kochjarová 1998b: 4 rel.
Kochjarová et al. 1997: 1 rel.
Kochjarová & Valachovič 2006: 19 rel.
Kochjarová et al. 2005: 1 rel.
Koó 1994: 2 rel.
Korotchenko & Didukh 1997: 4 rel.
Korotchenko et al. 2009: 3 rel.
Kostuch & Misztal 2007: 4 rel.
Kovács 2001: 16 rel.
Kovács 2003: 25 rel.
Kovács & Manoliu 1972: 5 rel.
Kozłowska 1928: 4 rel.
Krippelová 1967: 9 rel.
Krippelová 1968: 2 rel.
Krstonošić. 2013: 60 rel.
Kubíková & Kučera 1999: 8 rel.
Kusák 1996: 14 rel.
Kuyper et al. 1978: 3 rel.
Kuzemko 2009: 5 rel.
Kuzemko 2011: 4 rel.
Kuzemko et al. 2014: 22 rel.
Leputsch 1997: 3 rel.
Maglocký 1979: 9 rel.
Májovský 1958a: 17 rel.
Májovský 1958b: 1 rel.
Májovský & Jurko 1958: 1 rel.

Majzlanová 1981: 1 rel.
Mann 1997: 1 rel.
Michalik 1999: 1 rel.
Michalko 1957: 1 rel.
Michálková et al. 2006: 1 rel.
Milka 1973: 3 rel.
Mirek 1978: 6 rel.
Mochnacký & Maglocký 1993: 8 rel.
Mucina 1986: 2 rel.
Nejezchlebová 2009: 8 rel.
Neuhäusl & Neuhäuslová-Novotná 1964: 3 rel.
Neuhauslová-Novotná 1968: 2 rel.
Nowak 2003: 4 rel.
Parabućski 1990: 2 rel.
Parabućski & Butorac 1993: 7 rel.
Perný 1999: 5 rel.
Petkovsek 1977: 15 rel.
Petkovsek 1978: 6 rel.
Petrinec 1999: 11 rel.
Pfusterschmid 1998: 8 rel.
Pöcheim 2004: 18 rel.
Pohoriljaková 1992: 2 rel.
Poldini 1989: 9 rel.
Pop 1996: 26 rel.
Pop et al. 1964: 1 rel.
Pospíšil 1957a: 1 rel.
Pospíšil 1957b: 4 rel.
Pospíšil 1962: 9 rel.
Pospíšil 1964: 6 rel.
Procházková 1996: 2 rel.
Purger 1993: 9 rel.
Rathmayer 1985: 1 rel.
Raţiu et al. 1969: 7 rel.
Regula-Bevilaqua 1983: 4 rel.
Řehořek 1969: 1 rel.
Reichenberger 1990: 3 rel.
Removčíková 1981: 11 rel.
Rotter 2002: 11 rel.
Rötzer 2004: 15 rel.
Ruzičková 1982: 2 rel.
Ruzičková 1986: 6 rel.
Ruzičková 1987: 2 rel.
Ruzičková 1997: 4 rel.
Ruzičková 2002: 1 rel.
Ruzičková & Halada 2005: 2 rel.
Ruzičková & Michalko 1982: 3 rel.
Ruzičková et al. 1999: 9 rel.
Sádovský 2004: 2 rel.
Sádovský & Eliás ml. 2003: 2 rel.
Sanda et al. 2005: 3 rel.
Sauberer & Buchner 2001: 5 rel.
Schneeweiss et al. 2002: 6 rel.
Schneider 1997: 6 rel.
Schneider-Binder 1970: 2 rel.
Schneider-Binder 1971: 19 rel.
Schuster 1977: 13 rel.
Schwarz 1991: 1 rel.
Seda 1975a: 1 rel.
Seda 1975b: 1 rel.
Seda 1975c: 2 rel.
Seda 1975d: 5 rel.
Seda 1976a: 1 rel.
Seda 1976b: 2 rel.
Šegulja 1974: 1 rel.
Šegulja 1977: 1 rel.
Seljak 1976: 8 rel.
Semenishchenkov 2009: 107 rel.
Sendtko 1997: 1 rel.
Sibík 2011: 1 rel.
Sillinger 1929: 2 rel.
Simon 1977: 4 rel.
Siroki Z 1956: 2 rel.
Sitásová 2002: 1 rel.
Sitásová & Kaduková 1997: 1 rel.
Six 1986: 3 rel.
Škodová et al. 2005: 12 rel.
Škornik 2000: 246 rel.
Škornik 2003: 6 rel.
Škovirová & Očka 2005: 3 rel.
Šmarda 1961: 1 rel.

Šmarda 1967: 2 rel.
Šmeralová 1980: 6 rel.
Šmiták & Grüll 1984: 1 rel.
Soo 1949: 32 rel.
Stavělová 1976: 5 rel.
Stevanović 1984: 13 rel.
Stjepanović-Veselčić 1953: 1 rel.
Stojanović 1981: 11 rel.
Šugar 1972: 28 rel.
Suňalová 1999: 1 rel.
Suteu 1979: 14 rel.
Täuber & Weber 1976: 5 rel.
Tichý 2006: 1 rel.
Tlusták 1972: 31 rel.
Tlusták 1975: 3 rel.
Tlusták 1976: 8 rel.
Tlusták 1990: 5 rel.
Toman 1975: 1 rel.
Tomazič 1949: 6 rel.
Towpasz & Kotańska 1999: 1 rel.
Trávníček 1987: 8 rel.
Trávníčková 1992: 3 rel.
Uhliarová 2005: 2 rel.
Uhlířová & Bernátová 2002: 3 rel.
Uhrin 2002: 4 rel.
Ujházy et al. 2003: 1 rel.
Unar 1975: 12 rel.
Unar 1979a: 1 rel.
Unar 1980: 28 rel.
Unar 2002: 2 rel.
Unar & Grüll 1984: 3 rel.
Urban 1992: 7 rel.
Urbanová 1971: 1 rel.
Urbanová 1977: 17 rel.
Urbanová 2002: 3 rel.
Urvichiarová 1963: 2 rel.
Valachovič 2004: 5 rel.
Válková-Tomisová 1978: 15 rel.
Veselá 2007: 13 rel.
Vicherek 1957a: 6 rel.
Vicherek 1957b: 7 rel.
Vicherek 1959: 1 rel.
Vicherek 1967: 10 rel.
Vicherek 1975: 1 rel.
Vicherek & Unar 1971: 22 rel.
Vozárová 1986: 2 rel.
Vučković 1991: 26 rel.
Vynokurov 2014b: 1 rel.
Wagner 1941: 3 rel.
Wagner 1950: 1 rel.
Willner et al. 2004: 13 rel.
Willner et al. 2013: 14 rel.
Wójciak & Urban 2011: 19 rel.
Wurm 1991: 2 rel.
Zahirović 2000: 6 rel.
Zahradníková-Rosetzká 1965: 6 rel.
Zinöcker 1992: 1 rel.
Zlinská 1987: 1 rel.
Zlinská 2000: 5 rel.
Zlinská 2004: 2 rel.
Other sources: 1502 rel.

Festucetalia valesiacae

Ambrozek 1989: 12 rel.
Ambrozek & Chytrý 1990: 2 rel.
Anioł-Kwiatkowska & Świerkosz 1992: 1 rel.
Ardelean 1983: 3 rel.
Babczyńska-Sendek 2005: 2 rel.
Babić 1965: 3 rel.
Badarau et al. 1995: 2 rel.
Badarau et al. 2001: 29 rel.
Benčaťová & Ujházy 1998: 1 rel.
Bergerová 1985: 1 rel.
Blanár 2005: 1 rel.
Blaženčić 1982: 5 rel.
Bojko 1934: 9 rel.
Borhidi & Dénes 1997: 2 rel.
Boşcaiu et al. 1966: 14 rel.

Boşcaiu et al. 1984: 13 rel.
Butorac 1989: 47 rel.
Celinski & Babczynska Magiera 1997: 10 rel.
Chlumská 1961: 1 rel.
Chytrý & Vicherek 1996: 13 rel.
Chytrý & Vicherek 2003: 8 rel.
Chytrý et al. 1997: 70 rel.
Cigánek 1998: 1 rel.
Ciufudeanu 1972: 1 rel.
Cristea & Csűrős 1976: 8 rel.
Csűrős et al. 1961: 21 rel.
Csűrős-Kaptalan 1962: 7 rel.
Cvachová 1983: 1 rel.
Danáková 2000: 4 rel.
Danihelka 1998: 3 rel.
Dengler et al. 2012: 20 rel.
Denk 2005: 3 rel.
Didukh & Vashenyak 2012: 104 rel.
Domin 1932: 1 rel.
Dúbravková et al. 2010: 5 rel.
Dúbravková-Michálková et al. 2008: 16 rel.
Dvořáková 1999a: 1 rel.
Dvořáková 1999b: 1 rel.
Eijsink et al. 1978: 18 rel.
Eliás 1986: 2 rel.
Fijałkowski & Izdebski 1957: 6 rel.
Filić 1984: 1 rel.
Fiserová 1991: 7 rel.
Frink 2010: 28 rel.
Frink & Szabo 2009: 12 rel.
Futák 1947: 1 rel.
Futák 1960: 2 rel.
Gawłowska 1958: 1 rel.
Głazek 1968: 42 rel.
Głowacki 1984: 2 rel.
Gergely &Raţiu 1965: 4 rel.
Gergely 1970: 6 rel.
Germany 2013: 11 rel.
Gomlya 2005: 12 rel.
Grodzińska 1970: 2 rel.
Grodzińska & Szarek-Łukaszewska 2009: 2 rel.
Grüll 1953: 4 rel.
Grüll 1976: 2 rel.
Grüll 1991: 4 rel.
Grüll 1984c: 11 rel.
Grüll 1984d: 6 rel.
Grüll 1985a: 1 rel.
Grüll 1985b: 1 rel.
Grüll 1985d: 2 rel.
Grüll 1988a: 2 rel.
Háberová et al. 1985: 1 rel.
Hadač & Šmarda 1944: 2 rel.
Hadinec et al. 2004: 1 rel.
Hantáková 1991: 8 rel.
Hartman 1958: 8 rel.
Hodisan 1968: 8 rel.
Holubová 1968: 2 rel.
Horáková 1972: 5 rel.
Horvatić & Gospodarić 1960: 1 rel.
Huspeka 1993: 1 rel.
Jurko 1951: 4 rel.
Jurko 1964: 1 rel.
Jurko 1969: 5 rel.
Jurko 1958a: 2 rel.
Jurko 1958b: 1 rel.
Kaleta 1965: 1 rel.
Karasová 1991: 2 rel.
Karasová 1996: 1 rel.
Kárpáti & Kárpáti 1965: 1 rel.
Klika 1931: 4 rel.
Klika 1937: 3 rel.
Klika 1938: 14 rel.
Klika 1945: 1 rel.
Kliment 1998: 1 rel.
Kliment et al. 2000: 29 rel.
Klimes 1983: 8 rel.
Koó 1994: 26 rel.
Korotchenko & Didukh 1997: 8 rel.
Korotchenko et al. 2009: 75 rel.
Kovács 2001: 11 rel.

Kovács & Máthé 1964: 13 rel.
Kovacs & Manoliu 1972: 1 rel.
Kozłowska 1928: 8 rel.
Krajina 1936: 2 rel.
Krippel 1954: 7 rel.
Krippelová 1967: 21 rel.
Krippelová 1968: 1 rel.
Krstonošić. 2013: 7 rel.
Kuzemko 2009: 4 rel.
Kuzemko 2011: 23 rel.
Kuzemko et al. 2014: 165 rel.
Magic 1969: 1 rel.
Maglocký 1973: 13 rel.
Maglocký 1979: 14 rel.
Maglocký 1982: 2 rel.
Májovský 1954: 10 rel.
Májovský 1955: 11 rel.
Májovský 1958a: 2 rel.
Májovský 1958b: 1 rel.
Májovský & Jurko 1956: 22 rel.
Májovský & Jurko 1958: 13 rel.
Margl 1973: 1 rel.
Miadok 1987: 9 rel.
Michalko 1957: 19 rel.
Michalko & Dzatko 1965: 1 rel.
Michálková et al. 2006: 4 rel.
Mirek 1978: 5 rel.
Mochnacký & Maglocký 1993: 8 rel.
Mucina 1986: 2 rel.
Nejezchlebová 2009: 2 rel.
Neuhäusl & Neuhäuslová-Novotná 1964: 10 rel.
Neuhauslová-Novotná 1968: 2 rel.
Niklfeld 1964: 3 rel.
Parabućski 1990: 1 rel.
Parabućski & Butorac 1993: 29 rel.
Parabućski et al. 1986: 7 rel.
Pavlíková 1981: 4 rel.
Petrík 2006: 1 rel.
Piwowarczyk & Przemyski 2010: 5 rel.
Pop 1996: 5 rel.
Pop et al. 1964: 2 rel.
Pospíšil 1954: 1 rel.
Pospíšil 1957b: 6 rel.
Purger 1993: 17 rel.
Ráčilová 1964: 4 rel.
Rafajová 1998: 9 rel.
Rathmayer 1985: 1 rel.
Raţiu et al. 1969: 16 rel.
Removčíková 1981: 2 rel.
Resmeriţa et al. 1967: 11 rel.
Rötzer 2004: 17 rel.
Ruprecht et al. 2009: 19 rel.
Ruzičková 1971: 3 rel.
Sanda et al. 2005: 40 rel.
Sauberer & Buchner 2001: 2 rel.
Schneeweiss et al. 2002: 5 rel.
Schneider 1997: 1 rel.
Schneider-Binder 1970: 2 rel.
Schneider-Binder 1971: 3 rel.
Schneider-Binder 1975: 11 rel.
Schneider-Binder 1977: 16 rel.
Schuster 1977: 33 rel.
Seda 1975a: 1 rel.
Seda 1975c: 1 rel.
Seda 1975d: 1 rel.
Seda 1976a: 1 rel.
Semenishchenkov 2009: 31 rel.
Sendtko 1997: 5 rel.
Shevchyk et al 1996: 3 rel.
Sillinger 1931: 1 rel.
Simon 1964: 5 rel.
Simon 1977: 14 rel.
Siroki Z 1956: 18 rel.
Sitásová 1999: 1 rel.
Sitásová 2000: 2 rel.
Škodová et al. 2005: 1 rel.
Šmarda 1942: 1 rel.
Šmarda 1961: 1 rel.
Šmarda 1967: 1 rel.
Šmarda 1975: 3 rel.

Soo 1949: 33 rel.
Stevanović 1984: 58 rel.
Stjepanović-Veselčić 1953: 30 rel.
Stojanović 1981: 49 rel.
Suteu 1979: 2 rel.
Szabo & Galan 1966: 4 rel.
Takács & Takácsné Kovács 1999: 1 rel.
Tichý et al. 1997: 7 rel.
Tlusták 1990: 2 rel.
Toman 1975: 3 rel.
Trávníček 1987: 2 rel.
Uhlmann 1938: 7 rel.
Unar 1975: 1 rel.
Unar 1980: 4 rel.
Unar & Grüll 1984: 3 rel.
Valachovič 1992: 2 rel.
Válková-Tomisová 1978: 1 rel.
Vicherek 1957b: 1 rel.
Vicherek 1975: 2 rel.
Vicherek & Unar 1971: 38 rel.
Vozárová 1986: 39 rel.
Vozárová 1990: 24 rel.
Vučković 1991: 24 rel.
Vučković 1980: 15 rel.
Vynokurov 2014a: 17 rel.
Vynokurov 2014b: 24 rel.
Wagner 1941: 9 rel.
Waitzbauer 1990: 1 rel.
Willner et al. 2004: 14 rel.
Wójciak & Urban 2011: 4 rel.
Wünschová 2003: 1 rel.
Wurm 1991: 1 rel.
Záborský 1971: 1 rel.
Zahirović 2000: 3 rel.
Zajacová 1961: 3 rel.
Zinöcker 1992: 10 rel.
Zlinská 2000: 1 rel.
Zolyomi 1939: 2 rel.
Other sources: 1501 rel.

Stipo-Festucetalia pallentis

Ambrozek 1989: 7 rel.
Ambrozek & Chytrý 1990: 3 rel.
Babczyńska 1978: 4 rel.
Babczyńska-Sendek 2005: 12 rel.
Badarau et al. 2001: 1 rel.
Baláz 1991: 9 rel.
Baňacká 1982: 12 rel.
Bernátová & Kliment 1982: 5 rel.
Bernátová & Uhlířová 1994: 3 rel.
Blanár 2005: 2 rel.
Blanár & Letz 2005: 2 rel.
Borhidi & Dénes 1997: 8 rel.
Bosáčková 1978: 6 rel.
Bosáčková et al. 1974: 17 rel.
Boşcaiu et al. 1966: 15 rel.
Boşcaiu et al. 1984: 1 rel.
Cachovanová 2010: 1 rel.
Celinski & Babczynska Magiera 1997: 1 rel.
Chlumská 1961: 15 rel.
Chytrý & Vicherek 1996: 18 rel.
Chytrý & Vicherek 2003: 10 rel.
Chytrý et al. 1997: 52 rel.
Ciufudeanu 1972: 7 rel.
Csergo 2009: 11 rel.
Csűrős 1958: 14 rel.
Csűrős-Kaptalan 1962: 8 rel.
Csűrős et al. 1961: 5 rel.
Cvachová et al. 1980: 1 rel.
Cvachová & Urbanová 1981a: 1 rel.
Cvachová & Urbanová 1981b: 1 rel.
Dančák & Duchoslav 2006: 7 rel.
Dengler et al. 2012: 8 rel.
Denk 2000: 4 rel.
Denk 2005: 4 rel.
Didukh & Vashenyak 2012: 6 rel.
Domin 1931: 3 rel.
Domin 1932: 5 rel.

Dúbravková et al. 2010: 1 rel.
Dúbravková-Michálková et al. 2008: 5 rel.
Dvořáková 1999a: 1 rel.
Dvořáková 1999b: 1 rel.
Eijsink et al. 1978: 10 rel.
Eliás 1986: 3 rel.
Eliás 1988: 3 rel.
Eliás jun. & Díte 2005: 1 rel.
Fajmonová 1995: 13 rel.
Fiserová 1991: 2 rel.
Frink 2010: 1 rel.
Futák 1947: 36 rel.
Futák 1960: 38 rel.
Gawłowska 1958: 2 rel.
Głazek 1968: 1 rel.
Gergely 1967: 5 rel.
Gergely 1970: 4 rel.
Gergely & Raţiu 1965: 5 rel.
Germany 2013: 2 rel.
Grodzińska 1970: 17 rel.
Grodzińska & Szarek-Łukaszewska 2009: 7 rel.
Grüll 1953: 1 rel.
Grüll 1976: 1 rel.
Grüll 1984a: 1 rel.
Grüll 1984c: 1 rel.
Grüll 1984d: 2 rel.
Grüll 1985b: 4 rel.
Grüll 1985c: 2 rel.
Grüll 1986b: 2 rel.
Grüll 1988b: 3 rel.
Háberová et al. 1985: 4 rel.
Háberová & Karasová 1990: 2 rel.
Hadač & Šmarda 1944: 1 rel.
Hantáková 1991: 2 rel.
Hartman 1958: 5 rel.
Hodişan 1965: 8 rel.
Horáková 1972: 3 rel.
Horváth 1946: 7 rel.
Jarosová & Mucina 1988: 3 rel.
Jurko 1951: 14 rel.
Kaźmierczakowa 2004: 8 rel.
Kaleta 1965: 4 rel.
Karasová 1991: 2 rel.
Karasová 1996: 1 rel.
Karasová 2004: 1 rel.
Karrer 1985a: 2 rel.
Karrer 1985b: 10 rel.
Klika 1926: 3 rel.
Klika 1929a: 16 rel.
Klika 1929b: 9 rel.
Klika 1931: 13 rel.
Klika 1932: 5 rel.
Klika 1936: 5 rel.
Klika 1937: 20 rel.
Klika 1945: 1 rel.
Kliment 1978: 7 rel.
Kliment 2002: 2 rel.
Kliment & Bernátová 2000: 5 rel.
Klimes 1983: 2 rel.
Knapp 1944: 3 rel.
Kochjarová 1997: 1 rel.
Kochjarová 1998b: 5 rel.
Kochjarová et al. 1997: 2 rel.
Kolbek et al.: 1 rel.
Koó 1994: 19 rel.
Kornaś 1958: 1 rel.
Kovačević-Viličić 1974: 12 rel.
Kovács & Máthé 1964: 2 rel.
Kovacs & Manoliu 1972: 4 rel.
Kozłowska 1928: 19 rel.
Krippelová 1968: 3 rel.
Krstonošić. 2013: 2 rel.
Kuzemko et al. 2014: 17 rel.
Láfersová-Jamrichová 1981: 2 rel.
Maglocký 1970: 18 rel.
Maglocký 1979: 41 rel.
Májovský 1954: 4 rel.
Májovský 1955: 4 rel.
Májovský & Jurko 1958: 5 rel.
Majzlanová & Somsák 1991: 1 rel.

Miadok 1987: 12 rel.
Michalko 1957: 16 rel.
Michálková 2003: 2 rel.
Michálková 2006: 13 rel.
Michálková et al. 2006: 8 rel.
Mikyska 1933: 4 rel.
Nejezchlebová 2009: 2 rel.
Niklfeld 1964: 2 rel.
Pavlíková 1981: 10 rel.
Petrík 1978: 16 rel.
Petrík 2006: 3 rel.
Petrík et al. 1982: 2 rel.
Pfusterschmid 1998: 6 rel.
Pietorová 1996: 14 rel.
Pop et al. 1960: 1 rel.
Pop et al. 1964: 24 rel.
Ráčilová 1964: 11 rel.
Rafajová 1998: 3 rel.
Raţiu et al. 1969: 1 rel.
Regula-Bevilacqua 1978: 4 rel.
Reichenberger 1990: 1 rel.
Ruprecht et al. 2009: 12 rel.
Ruzičková 1982: 1 rel.
Sanda et al. 2005: 57 rel.
Sauberer & Buchner 2001: 23 rel.
Schneider-Binder 1970: 21 rel.
Schneider-Binder 1971: 1 rel.
Schneider-Binder 1977: 4 rel.
Schuster 1977: 7 rel.
Schwarz 1991: 7 rel.
Semenischenkov 2009: 2 rel.
Sillinger 1931: 5 rel.
Sillinger 1933: 15 rel.
Simeková & Pitoniak 1976: 1 rel.
Simon 1977: 7 rel.
Sitásová 2000: 3 rel.
Sitásová 2002: 2 rel.
Sitásová & Kaduková 1997: 2 rel.
Six 1986: 4 rel.
Skolek 2004: 1 rel.
Škovirová & Očka 2005: 2 rel.
Šmarda 1961: 1 rel.
Šmarda 1967: 1 rel.
Šmarda 1970: 7 rel.
Šmarda 1975: 2 rel.
Šmarda & Šmarda 1968: 1 rel.
Soo 1944: 1 rel.
Soo 1949: 9 rel.
Stjepanović-Veselčić 1953: 6 rel.
Šumberová 1995: 1 rel.
Suteu 1968: 16 rel.
Suteu 1969: 13 rel.
Suteu 1979: 4 rel.
Suza & Zlatník 1928: 6 rel.
Svandová-Ursíniová 1966: 13 rel.
Szaboóvá-Baxandale 1994: 2 rel.
Tichý & Chytrý 1996: 2 rel.
Tichý et al. 1997: 10 rel.
Toman 1975: 7 rel.
Tracey 1980: 2 rel.
Trávníček 1987: 5 rel.
Trinajstić et al. 2006: 13 rel.
Uhlířová 2006: 12 rel.
Uhlířová & Bernátová 2003: 2 rel.
Uhlířová & Bernátová 2004: 3 rel.
Uhlířová & Petrík 2006: 4 rel.
Uhlmann 1938: 5 rel.
Unar 1975: 7 rel.
Unar 1979b: 1 rel.
Unar 1980: 4 rel.
Valachovič 1992: 4 rel.
Valachovič 1995: 4 rel.
Valachovič & Jarolímek 1994: 4 rel.
Vicherek & Unar 1971: 10 rel.
Vozárová 1986: 24 rel.
Vrbek 2005: 4 rel.
Wagner 1941: 8 rel.
Waitzbauer 1990: 2 rel.
Willner et al. 2004: 6 rel.
Willner et al. 2013: 1 rel.

Zlatník 1928: 7 rel.
Zolyomi 1939: 48 rel.
Other sources: 915 rel.

References

Ambrozek, L. 1989. *Vybrané komplexy xerotermní vegetace na jižní Moravě*. Manuscript, Dipl. pr., PřF UK, Praha, CZ.

Ambrozek, L. & Chytrý, M. 1990. Die Vegetation der Zwergstrauchheiden im xerothermen Bereich am Südostrand des Böhmischen Massivs. *Čas. Morav. Muz. Brno* 75: 169–184.

Anioł-Kwiatkowska, J. & Świerkosz, K. 1992. Flora i roślinność rezerwatu Ostrzyca Proboszczowicka oraz jego otoczenia. *Prace Bot.* 48: 45–115.

Ardelean, A. 1983. O noua asociaţie pe Valea Crişului Alb *Agrostio-Festucetum valesiacae*. *Contribuţii Botanice* 145–149.

Auer, M. 1982. *Wiesengesellschaften im Wiener Wald*. Diplomarbeit, Univ. f. Bodenkultur, Wien, AT.

Babczyńska, B. 1978. Zbiorowiska murawowe okolic Olsztyna koło Częstochowy. *Acta Biologica* 5: 169–215.

Babczyńska-Sendek, B. 2005. Problemy fitogeograficzne i syntaksonomiczne kserotermów Wyżyny Śląskiej. *Prace Naukowe Uniwersytetu Śląskiego w Katowicach* 2296: 1–237.

Babczyńska-Sendek, B. & Andrzejczuk, I. 1977. Goryczka krzyżowa *Gentiana cruciata* L. w okolicach Tarnowskich Gór. *Natura Silesiae Superioris* 1: 33–42.

Babić, N. 1965. *Močvarna i livadska vegetacija Koviljskog rita*. Doktorska disertacija, Univerzitet u Beogradu, Prirodno-matematički fakultet, Beograd, RS.

Bača, A. 2010. *Sukcesné zmeny opustených podhorských travinnobylinných porastov na Hornom Pozitaví vo vzťahu k ekologickým faktorom*. Dizertačná práca, Depon. in Ústav krajinnej ekológie SAV, Bratislava, SK.

Badarau, A. S., Derzsi, St. & Man, T. 2001. Cercetari biogeografice asupra speciilor stepice-silvostepice de *Astragalus* L. din depresiunea Transilvaniei (II). Studia Universitatis Babeş-Bolyai, Geographia, 46: 51–67.

Badarau, A. S., Groza, G. & Peştina, C. 1995. *Centaurea orientalis* L. (element biogeografic sarmatic nord-balcanic) şi *Centaurea ruthenica* Lam. (element sarmato-turanic) in flora podişului Transilvaniei. *Contribuţii Botanice* 33: 13–20.

Balátová-Tuláčková, E. & Hájek, M. 1998. Feuchtwiesengesellschaften des südlichen Teiles des Landschaftsschutzgebietes Bílé Karpaty (Südost-Mähren). *Verh. Zool.-Bot. Ges. Österreich* 135: 1–40.

Baláz, D. 1991. *Fytocenologický a floristický výskum projektovanej SPR Macková v Nízkych Tatrách*. Dipl. Pr. (msc.) depon. in Prí. Fak. UK, Bratislava, SK.

Baňacká, O. 1982. *Xerotermná vegetácia Drieňovca v CHKO Slovenský kras*. Dipl. Pr. (msc.) depon in Pri. Fak. UK, Bratislava, SK.

Bednář, V. & Velísek, V. 1967. Příspìvek k poznání současného stavu xerotermní vegetace Grygova u Olomouce. *Acta Univ. Palack. Olomuc*., 25, *Biol*. 9: 5–14.

Benčaťová, B. & Ujházy, K. 1998. *Floristický kurz Zvolen 1977*. Techn. Univ., Zvolen, SK.

Bergerová, J. 1985. *Floristico-fytocenologické pomery Plesivecké planiny*. Dipl. práca (msc.), Depon. in NP Slovenský kras, Brzotín, SK.

Bernátová, D. & Kliment, J. 1982. Seslerio variae-Caricetum approximatae – nové rastlinné spoločenstvo v centrálnych pohoriach Západných Karpát. *Preslia* 54: 341–348.

Bernátová, D. & Uhlířová, J. 1994. Globulario cordifoliae-Caricetum humilis ass. nova in the rocky part of the Veľká Fatra Mts. *Biologia* 49: 1–11.

Bernátová, D., Jarolímek, I., Kliment, J. & Zaliberová, M. 2002. Floristické novinky a zaujímavosti z niektorých pohorí, kotlín a nízin Slovenska. *Bull. Slov. Bot. Spoločn*. 24: 101–111.

Blanár, D. 2005. Nález druhu Asyneuma canescens na Muránskej planine vo vzťahu k výskytu na Slovnesku. *Reussia, Revúca*, 2/2: 95–128.

Blanár, D. & Letz, D.R. 2005. Sempervivum marmoreum agg. na Muránskej planine. *Reussia, Revúca,* 2/2: 129–151.

Blaženčić, Ž. 1982. Zajednica Andropogoneto-Euphorbietum pannonicae u stepskim fragmentima Fruške gore. *Ekologija* 17: 1–13.

Bojko, H. 1934. Die Vegetationsverhältnisse im Seewinkel. *Beih. Bot. Centralbl*. 51B: 600–747.

Borhidi, A. & Dénes, A. 1997. A Mecsek és a Villányi hegység sziklagyepjei. In: Borhidi, A. & Szabó, L. (eds.): *Studia Phytologica Jubilaria. Dissertationes in honorem jubilantis Adolf Olivér Horvát doctor academiae in anniversario nonagesimo nativitatis 1907-1997*. JPTE, Növény, HU.

Bosáčková, E. 1972. *Súčasný stav a ochrana slatinnej vegetácie Zitného ostrova*. Práce a stúdie ČSOP, Bratislava, seria 1, 82 pp.

Bosáčková, E. 1975. Rastlinné spoločenstvá slatinných lúk na Záhorskej nízine.čs. *Ochr. Prír., Bratislava,* 15: 173–273.

Bosáčková, E. 1978. *Inventarizačný prieskum Státnej prírodnej rezervácie Tiesňavy (CHKO Malá Fatra)*. časť: nelesná vegetácia. SÚPSOP, depon in správa ChKO Malá Fatra, SK.

Bosáčková, E., Cvachová, A. & Urbanová, V. 1974. Floristický a fytocenologický náčrt Suľovských skál. In: Stollmann, A. (ed.), *Súlovské skaly SPR*. Vyd. Osveta, Martin, SK.

Boşcaiu, N., Gergely, I., Codoreanu, V., Raţiu, O & Micle, F. 1966. Descrierea asociaţiilor vegetale. In: Flora şi vegetaţia rezervaţiei naturale "Defileul Crişului Repede". *Contribuţii Botanice*: 167–258.

Boşcaiu, N., Täuber, F. & Raulea, M. 1984. Aspecte de vegetaţie de la Rapa Roşie (judetul Alba). *Ocrotirea Naturii* 28: 36–44.

Bozková, J. 2005. *Luční a pastvinná vegetace v území středního toku řeky Svratky*. Ms. dipl. pr., depon. in Knih. kat. bot., Přír. Fak. MU, Brno, CZ.

Bravencová, L. 2003. *Vegetace Národní přírodní rezervace čertoryje*. Ms. dipl. pr., depon. in Knih. kat. bot., Přír. Fak. MU, Brno, CZ.

Brzeg, A. 2005. *Zespoły kserotermofilnych ziołorośli okrajkowych z klasy Trifolio-Geranietea sanguinei Th. Müller 1962 w Polsce*. Bogucki Wydawnictwo Naukowe, Poznań, PL.

Bulohov, A.D. 2001. *Travyanaya rastitel'nost' Yugo-Zapadnogo Nechernozem'ya Rossii* [*Herbal vegetation of South-West Nechernozemia of Russia*]. Izd-vo BGU, Bryansk, RU. [in Russian.].

Butorac, B. 1989. *Vegetacija Sremskog lesnog platoa*. Doktorska dis., Univerzitet u Novom Sadu, Prirodno-matematički fakultet, Novi Sad, RS.

Cachovanová, L. 2010. *Rastlinné spoločenstvá pastvín a lúk v Strázovských vrchoch*. Dipl. Pr. (msc.), depon in Prír. fak. Univerzity Palackého v Olomouci, CZ.

Čechová, J. 1999. *Botanický inventarizační prùzkum NPP Malhotky*. Ms., depon. AOPaK ČR, Praha, CZ.

Čechová, J. 1999. *Botanický inventarizační prùzkum PP Navdavky u Nìmčan*. Ms., depon. AOPaK ČR, Praha, CZ.

Celinski, F., Babczynska, B. & Magiera, A. 1997. Les pelouses psammophiles a *Armeria elongata* pres de Myslowice (Plateau Silesien, Pologne). *Colloques phytosociologiques* VI, Les peleuses seches, Lille, FR.

Chlumská, L. 1961. *Rostlinná společenstva čebínky*. Ms. dipl. pr., depon. in Knih. kat. bot., Přír. Fak. MU, Brno, CZ.

Chytrý, M. & Vicherek, J. 1996. Přirozená a polopřirozená vegetace údolí řek Oslavy, Jihlavy a Rokytné. *Přírod. Sborn. Západomor. Muz. Třebíč*, 22: 1–125.
Chytrý, M. & Vicherek, J. 2003.Travinná, keříčková a křovinná vegetace Národního parku Podyjí/Thayatal. *Thayensia* 5: 11–84.
Chytrý, M., Mucina, L., Vicherek, J., Pokorny-Strudl, M., Strudl, M., Koó, A.J. & Maglocký, S. 1997. Die Pflanzengesellschaften der westpannonischen Zwergstrauchheiden und azidophilen Trockenrasen. *Diss. Bot*. 277: 1–118.
Cigánek, D. 1998. *Synantropní vegetace Národního parku Podyjí*. Ms. dipl. pr., depon. in Knih. kat. bot., Přír. Fak. MU, Brno, CZ.
Ciufudeanu, F. 1972. Aspecte de floră şi de vegetaţie din rezervaţia Calcarele de la Ampoiţa (Piatra Ampoiţei) (jud. Alba). *Ocrotirea Naturii* 16: 217–219.
Cristea, V. & Csuros, S. 1976. Aspecte din vegetatia pantelor erodate ale Podisului Secaselor. *Contribuţii Botanice* 103–110.
Csergo, A. 2009. *Phytosociology, ecology and phylogeography of Saponaria bellidifolia Sm. in the Apuseni Mountains (Southeastern Carpathians, Romania)*. PhD thesis, Cluj-Napoca, RO.
Csűrős, I. 1958. Cerecetari de vegetaţie pe Masivul Scarişoara-Beldioara. *Studia Universitatis Babes-Bolyai, Biologia* 105–128.
Csűrős-Kaptalan, M. 1962. Aspecte din vegetaaia Cheii Turului. *Studia Universitatis Babes-Bolyai, Biologia* 17–32.
Csűrős, S., Resmerita, I., Csuros-Kaptalan, M. & Gergely, I. 1961. Contributii la cunoasterea pajistilor din Campia Transilvaniei si unele consideratiuni cu privire la organizarea terenului. *Studia Universitatis Babes-Bolyai, Biologia* 15–61.
Cvachová, A. 1983. *Imventarizačný výskum botanický PP Prielom Muránky*. Mmsc. depon. in Správa NP Slovensky kras, Brzotin, SK.
Cvachová, A., Skovirová, K. & Urbanová, V. 1980. Charakteristika niektorých bezlesných rastlinných spoločenstiev Gaderskej doliny a Blatnickej doliny. *Ochr. Prír., 3B, Bratislava*, 200–240.
Cvachová, A. & Urbanová, V. 1981a. Charakteristika bezlesných spoločenstiev Choča. *Liptov, Martin*, 6: 195–212.
Cvachová, A. & Urbanová, V. 1981b. Spoločentvá skál, sutín a reliktných borín Státnej prírodnej rezervácie Rozsutec. In: Janík M., Stollman A. (eds.), *Rozsutec*, Osveta, Martin, SK.
Danáková, A. 2000. *Vegetácia na banských haldách v okolí Banskej Stiavnice*. KDP, Depon in Katedra Botaniky Pri. Fak. UK, Bratislava, SK.
Dančák, M. & Duchoslav, M. 2006. Flóra a vegetace Národní přírodní rezervace Spranìk (Javoříčský kras). *Čas. Slez. Zem. Muz., ser. A*: 201–227.
Danihelka, J. 1998. *Botanický inventarizační prùzkum národní přírodní památky Dunajovické kopce s námìty pro přípravu plánu péče*. Ms., depon. AOPaK ČR, Brno, CZ.
Danihelka, J. & Grulich, V. 2000. Pampeliska pozdní (Taraxacum serotinum) v české republice. *Zpr. Čes. Bot. Společ.* 34 (1999): 123–134.
Dengler, J., Becker, T., Ruprecht, E., Szabó, A., Becker, U., Beldean, M., Bita-Nicolae, C., Dolnik, C., Goia, I., (...) & Uğurlu, E. 2012. *Festuco-Brometea* communities of the Transylvanian Plateau (Romania) – a preliminary overview on syntaxonomy, ecology, and biodiversity. *Tuexenia* 32: 319–359.
Denk, T. 2000. Flora und Vegetation der Trockenrasen des tertiären Hügellandes nördlich von St. Pölten aus arealkundlicher sowie naturschutzfachlicher Sicht. *Stapfia* 72.
Denk, T. 2005. Flora und Xerothermvegetation der Schotterterrassen im Unteren Traisental. *Wiss. Mitt. Niederösterr. Landesmus*. 17: 7–182.
Didukh, Y.P. & Vasheniak, Y.A. 2012. Stepova roslynnist Tsentralnogo Podillya. [Steppe vegetation of Central Podillya]. *Ukrainian Botanical Journal* 69: 789–817. [in Ukrainian.]
Domin, K. 1931. Tři snímky Seslerieta z Rachsturnu v Malých Karpatech. *Vìda Přír., Praha*, 12: 118–121.
Domin, K. 1932. Nejvýznačnejsí travinná společenstva čachtických kopcu v jihozápadním Slovensku. *Rozpr. II. Tr. Čs. Akad., Praha*, 52/24: 1–10.
Dubová, J. 1978. *Vegetační a floristické pomìry východnì od Vizovic*. Ms., dis. pr., depon. in Knih. kat . biol. rostl. UJEP, Brno, CZ.
Dubová, J. & Unar, J. 1986. Anthoxantho-Agrostietum Sill. 1933 emend. Jurko 1969 in the Vizovice Hills (Vizovická pahorkatina). *Scripta Fac. Sci. Nat. Univ. Purk. Brun., Biol.*, 16: 41–50.
Dúbravková, D., Hegedüsová, K., Janišová M. & Škodová, I. 2010. New vegetation data of dry grasslands in the Western Carpathians and the northern Pannonian Basin. *Tuexenia* 30: 357–374.
Dúbravková-Michálková, D., Janisová, M., Kolbek, J. et al. 2008. Dry grasslands in the Slovenský kras Mts (Slovakia) and the Aggteleki karszt Mts (Hungary) - a comparison of two classification approaches. *Hacquetia* 7: 123–140.
Dvořáková, O. 1999a. Ekobiologická studie ohroženého druhu Bromus squarrosus L. *Thayensia* 2: 24–37.
Dvořáková, O. 1999b. *Ekobiologická studie ohrozených druhù: Bromus squarrosus L. a Mercurialis ovata Sternb. et Hoppe*. Ms. Dipl. pr.; depon. in: PřF MU Brno, CZ.
Dzubinová, Ľ. in Pitoniak, P. 1978. Flora a vegetácia Chránenej krajinnej oblasti Slovenský Raj. *Biologické práce*, XXIV, 6: 81–93.
Ebenberger, H. 1993. *Die Vegetation von Sophienalpe und Mostalm im Wienerwald*. Diplomarbeit, Univ. f. Bodenkultur, Wien, AT.
Eggler, J. 1959. Wiesen und Wälder im oststeirisch-burgenländischen Grenzgebiet. *Mitt. Naturwiss. Ver. Steiermark* 89: 5–34.
Eijsink, J.G.H.M., Ellenbroek, G.A., Holzner, W. & Werger, M.J.A. 1978. Dry and semi-dry grasslands in the Weinviertel , Lower Austria. *Vegetatio* 36: 129–148.
Eliás, P. 1986. Vegetácia SPR Hrdovická a Solčiansky háj a projektovanej SPR Kovarecká dubina. *Rosalia, Nitra*, 3: 33–79.
Eliás, P. 1987. Vegetácia severovýchodnej časti Tríbeča (Skupina Sokolca) 3. Spolčenstvá rúbanísk a lesných lemov. *Rosalia, Nitra*, 4: 173–192.
Eliás, P. 1988. Príspevok k poznaniu rastlinných spoločenstiev Chráneného náleziska Dobrotínske skaly (pohorie Tríbeč). *Rosalia, Nitra*, 5: 67–75.
Eliás, P. jun. & Díte, D. 2005. Nová lokalita jazýčkovca jadranského (Himantoglossum adriaticum Baum.) na Slovensku. *Ochr. Prír, Banská Bystrica*, 24: 125–127.
Eliás, P. ml., Galvánek, D. & Kosťál, J. 2002. Nové lokality prerastlíka prútnatého (Bupleurum affine) v Strázovských vrchoch. *Rosalia, Nitra*, 16: 31–34.
Fajmon, K. 2006. *Inventarizační prùzkum přírodní rezervace Machová. Závìrečná zpráva z oboru botanika. Stav v roce 2006*. Ms. Inventariz. prùzk.; depon. in: Správa CHKO Bílé Karpaty, Veselí n. Moravou, CZ.
Fajmonová, E. 1995. Xerotermná vegetácia v juhozápadnej časti Chránenej krajinnej oblasti Strázovské vrchy. *Naturae Tutela, Zb. Slov. Múz. Ochr. Prír. Jaskyn., Liptovský Mikulás*, 3: 213–221.
Falťan, V. 1999. *Vegetačnogeografická charakteristika okolia Kysuckého Nového Mesta s ohľadom na vegetáciu biotopov*. Dipl. Pr. (msc.) depon. in KFGG, Pri. Fak. UK, Bratislava, SK.
Fijałkowski, D. 1958. Roślinność leśno-stepowa w řabuniach koło Zamościa. *Ann. Univ., Lublin* XIII(6).
Fijałkowski, D. & Izdebski, K. 1957. Zbiorowiska stepowe na Wyżynie Lubelskiej. *Annales Universitatis, Lublin* XII.
Filić, Đ. 1984. *Biljni pokrov kanala jugoistočne Slavonije*. Magistarski rad. Prirodoslovno-matematički fakultet, Sveučilišta u Zagrebu, Zagreb, HR.

Fiserová, D. 1991. *Mapování vegetace části Silické planiny pomocí metod DPZ. Metodický příspìvek*. Msc., ČÚOP Praha, CZ.
Florian, B. 1992. *Phänologische und ökologische Untersuchungen in Wienerwaldwiesen*. Diplomarbeit, Univ. Wien.
Frink, J. P. 2010. *Pajistile de pe Valea Ariesului intre Lupsa si Turda*. Presa Universitara Clujeana, Cluj-Napoca, RO.
Frink, J.P. & Szabo, A. 2009. Distribution of Ephedra distachya L. subsp. distac. *Kanitzia* 16: 119–132.
Futák, J. 1947. *Xerotermná vegetácia skupiny Kňazného stola*. Sp. Sv. Vojtecha, Trnava, SK.
Futák, J. 1960. *Xerotermná vegetácia juznej časti Strázovskej hornatiny*. Kand. dizert. práca (msc.) depon in BU SAV, Bratislava, SK.
Gawłowska, W. 1958. Ochrona naturalnych zasobów a możliwości uprawy miłka wiosennego *Adonis vernalis* L. *Ochr. Przyr*. 25: 111–140.
Gaži-Baskova, V., Ehrlich, I. & Jerčić, J. 1983. Fitocenološka istraživanja pašnjaka ovaca na području Žumberka. *Poljopr. znanst. smotra* 63: 587–595.
Gergely, I. 1967. Pajişti de stancarii din partea nordica a munţilor Trascau. *Contribuţii Botanice* 131–143.
Gergely, I. 1970. Asociaţii stepice montane din partea nordica a munţilor Trascaului. *Contribuţii Botanice* 167–181.
Gergely, I. & Raţiu, F. 1965. Aspecte de vegetaţie din Cheile Aiudului. *Contribuţii Botanice* 177–187.
Germany, M. 2013. *Inselbiogeographie der Steppenrasen auf Mergelrutschhügeln in der Tarnava Mare-Region in Transsylvanien (Rumänien)*. Diplomarbeit, Johannes Gutenberg-Universität Mainz, DE.
Ghişa, E. 1941. Cercetari asupra as. *Stipetum stenophyllae* cu *Danthonia calycina* in Transilvania. *Buletinul Gradinii Botanice si al Muz. Bot. Cluj, la Timişoara*, 21: 56–67.
Ghişa, E. 1962. Rezervaţia botanica de la Zaul de Campie. *Ocrotirea Naturii* 6: 11–30.
Głazek, T. 1968. Roślinność kserotermiczna Wyżyny Sandomierskiej i Przedgórza Iłżeckiego. *Mon. Bot.* 25: 1–131.
Głowacki, Z. 1984. *Interesująca roślinność skarpy w okolicy wsi Skalka i jej antropogeniczne zmiany*. Zeszyty Naukowe 4, seria Nauki Przyrodnicze, WSRP, Siedlce, pp. 177–188.
Gogela, J. 1971. *Rostlinná společenstva luční a pastvinné vegetace Hostýnských vrchù*. Ms. dipl. pr., depon. in Knih. kat. bot., Přír. Fak. MU Brno, CZ.
Gomlya, L.M. 2005. Roslynnist’ dolyny richky Khorol [Vegetation of the Khorol River valley]. *Ukrayins’kyi fitocenologichnyi zbirnyk, Ser. A,* 1 (22): 1–187. [in Ukrainian.].
Grodzińska, K. 1970. Zbiorowiska kserotermiczne Skalic Nowotarskich i Spiskich (Pieniński Pas Skałkowy). *Fragm. Flor. Geobot*. 16: 401–432.
Grodzińska, K. 1979. Mapa zbiorowisk roślinnych rezerwatu Przełom Białki pod Krempachami (Pieniński Pas Skałkowy). *Ochrona Przyr*. 42: 29–74.
Grodzińska, K. & Szarek-Łukaszewska, G. 2009. Heavy metal vegetation in the Olkusz region (Southern Poland). *Polish Botanical Journal* 54: 105–112.
Grüll, F. 1953. Stepní reservace na okraji Zdánských lesù. *Ochr. Přír.* 8: 36–39.
Grüll, F. 1960. Přírodní reservace "Baračka" u Bučovic. *Ochr. Přír*. 15: 79–81.
Grüll, F. 1976. *Státní přírodní rezervace "Velatická slepencová stráň" (Geobotanická studie).* Ms., depon. AOPaK ČR, Brno, CZ.
Grüll, F. 1984a. *Inventarizační prùzkum vegetačních pomìrù přírodní rezervace Býčí skála*. Ms., depon. AOPaK ČR, Praha, CZ.
Grüll, F. 1984b. *Inventarizační prùzkum vegetačních pomìrù přírodní rezervace Hádecká planinka.* Ms., depon. AOPaK ČR, Praha, CZ.
Grüll, F. 1984c. *Inventarizační prùzkum vegetačních pomìrù přírodní rezervace Pustý kopec u Konic.* Ms., depon. AOPaK ČR, Praha, CZ.
Grüll, F. 1984d. *Inventarizační prùzkum vegetačního krytu přírodní rezervace Na Kocourkách*. Ms., depon. AOPaK ČR, Praha, CZ.
Grüll, F. 1984e. *Inventarizační prùzkum vegetačního krytu státní přírodní rezervace Skalka-Polomsko.* Ms., depon. AOPaK ČR, Praha, CZ.
Grüll, F. 1985a. *Inventarizační prùzkum vegetačního krytu přírodní rezervace Křéby - okres Kromìříz.* Ms., depon. AOPaK ČR, Praha, CZ.
Grüll, F. 1985b. *Inventarizační prùzkum vegetačního krytu přírodní rezervace Pekárka u Ivančic.* Ms., depon. AOPaK ČR, Praha, CZ.
Grüll, F. 1985c. *Inventarizační prùzkum vegetačního krytu přírodní rezervace Vìtrník (okres Vyskov).* Ms., depon. AOPaK ČR, Praha, CZ.
Grüll, F. 1985d. Novì zjistìná a přehlízená ruderální společenstva na území Brna. *Preslia* 57: 53–64.
Grüll, F. 1986a. *Inventarizační prùzkum vegetačního krytu přírodní rezervace Baračka u Klobouček, okres Vyskov*. Ms., depon. AOPaK ČR, Praha, CZ.
Grüll, F. 1986b. *Inventarizační prùzkum vegetačního krytu přírodní rezervace Na Adamcích*. Ms., depon. AOPaK ČR, Praha, CZ.
Grüll, F. 1988a. *Inventarizační prùzkum vegetačních pomìrù CHPV Nosperk*. Ms., depon. AOPaK ČR, Brno, CZ.
Grüll, F. 1988b. *Inventarizační prùzkum vegetačních pomìrù CHPV Zázmoníky u Bořetic*. Ms., depon. AOPaK ČR, Brno, CZ.
Grüll, F. 1988c. *Inventarizační prùzkum vegetačních pomìrù chránìného přírodního výtvoru "Obřanská stráň".* Ms., depon. AOPaK ČR, Praha, CZ.
Grüll, F. 1989. *Inventarizační prùzkum vegetačních pomìrù CHPV Netopýrky*. Ms., depon. AOPaK ČR, Brno, CZ.
Grüll, F. 1991. *Inventarizační prùzkum chránìné přírodní památky člupy*. Ms., depon. AOPaK ČR, Praha, CZ.
Háberová, I. et al. 1985. *Vegetácia krasových oblastí SSR z hľadiska ochrany prírody*. Záv. správa výskumnej úlohy VI-3-3/03 (msc.) depon. in PrFUK Bratislava, SK.
Háberová, I. & Karasová, E. 1990. *Inventarizačný výskum botanický SPR Zádielska tiesňava*. Msc. depon. in Správa NP Slovensky kras, Brzotin, SK.
Hadač, E., Terray, J., Klescht, V. & Andresova, J. 1997. Some herbaceous plant communities from the Bukovské vrchy hills in NE Slovakia. *Thaiszia* 7: 191–220.
Hadač, E.& Smarda, J. 1944. Příspìvek k výzkumu společenstev nasich reservací. *Krása Naseho Domova* 36: 120–122.
Hadinec, J. & Lustyk, P. (eds.) 2008. Additamenta ad floram Reipublicae Bohemicae. VII. Zpr. Čes. Bot. Společ. 43/2: 251–336.
Hadinec, J., Lustyk, P. & Procházka, F. (eds.) 2004. Additamenta ad floram Republicae Bohemicae IV. *Zpr. Čes. Bot. Společ*. 40/1: 77–149.
Hantáková, L. 1991. *Semixerotermní nelesní společenstva Hané*. Ms. dipl. pr., depon. PřF UP, Olomouc, CZ.
Hartman, Z. 1958. *Xerothermní kvìtena území jiznì Zdánického lesa*. Ms. dipl. pr., depon. in Knih. kat. bot., Přír. Fak. MU, Brno, CZ.
Hodişan, I. 1965. Vegetaţia saxicola de la Cheile Feneşului (raion Alba. Reg. Hunedoara). *Studia Universitatis Babeş-Bolyai, Ser. Biol*., 2: 9–22.
Hodişan, I. 1968. Cercetari fitocenologice asupra pajiştilor din bazinul Feneşului (raion Alba. Reg. Hunedoara). *Contribuţii Botanice* 209–230.
Holubová, J. 1968. *Rostlinná společenstva kopce "Hády" u Brna*. Ms. dipl. pr., depon. in Knih. kat. bot., Přír. Fak. MU, Brno, CZ.
Horáková, E. 1972. *Geobotanická studie o xerotermní vegetaci jizní části Moravské brány*. Ms. dipl. pr., depon. PřF UP, Olomouc, CZ.
Horváth, A.O. 1946. A pécsi Mecsek (Misina) természetes növényszövetkezetei. Dunántúli *Tudományos Intézet közleményei* 8: 1–52.

Horvatić, S. & Gospodarić, LJ. 1960. *Sporobolus vaginaeflorus* (Torr.) Wood. u biljnom pokrovu Hrvatske. *Acta Bot. Croat*. 18/19: 79–103.
Huspeka, J. 1993. *Wiesen und Wiesenbrachen an der Norabdachung des Wienerwaldes*. Diplomarbeit, Univ. Wien, Wien, AT.
Jarosová, E. & Mucina, L. 1988. On Thermophilous Fringe Communities of the Slovak Karst. *Abst. Botanica* 12: 143–162.
Jongepier, J. W. & Jongepierová, I. 1993. *Inventarizační prùzkum. Přírodní památka Hlozí*. Ms. depon. AOPaK ČR, Praha, CZ.
Jongepierová, I. & Jongepier, J.W. 2008. *Inventarizační prùzkum PP Hlozí z oboru botanika*. Ms. Inventariz. prùzk., depon. in Krajský úřad Jihomoravského kraje, Brno, CZ.
Jurko, A. 1951. *Vegetácia Stredného Pohornádia*. SAVU, Bratislava, SK.
Jurko, A. 1958a. Ďalšie lokality zlatej brady (*Chrysopogon gryllus* Trin.) na južnom Slovensku. *Acta Fac. Rer. Natur. Univ. Comen., Botanica, Bratislava*, 2: 315–322.
Jurko, A. 1958b. *Pôdne ekologické pomery a lesné spoločenstvá Podunajskej nížiny*. Vydavateľstvo SAV, Bratislava, SK.
Jurko, A. 1964. Feldheckengesellschaften und Uferweidengebusche des Westkarpatengebietes. *Biol. Pr., SAV Bratislava*, 10: 1–100.
Jurko, A. 1969. Die Weidegesellschaften des Strázover Berglandes in der Nordwestslowakei und die syntaxonomischen Probleme des Cynosurion-Verbandes in den Westkarpaten. *Folia Geobot. Phytotax*. 4: 101–132.
Jurko, A. 1970. Weidengesellschaften des Kremnitzer Gebirges, Slowakei. *Folia Geobot. Phytotax.* 5: 117–132.
Jurko, A. 1971a. Beitrag zu den Weidengesellschaften des Bezirkes Presov (Ostslowakei). *Preslia* 43: 234–248.
Jurko, A. 1971b. Vegetationskundliches Material zu den Weidengesellschaften aus dem Orava Gebiet. *Biologia* 26: 317–334.
Jurko, A. 1980. Extenzívne pasienky na andezitoch východného Slovenska. *Biologia* 35: 733–742.
Kaźmierczakowa, R. (ed.) 2004. *Charakterystyka i mapa zbiorowisk roślinnych Pienińskiego Parku Narodowego*. Studia Naturae 49.
Kaleta, M. 1965. *Vegetačné pomery Devínskej Kobyly*. Dipl. práca msc. depon. in PriFUK Bratislava, SK.
Kaligarič, M. 1997. *Vegetecija primorskega Krasa in slovenske Istre*. Zgodovinsko društvo za južno Primorsko, Znanstevno raziskovalno središče Republike Slovenije Koper, Koper, SL.
Karasová, E. 1991. *Inventarizačný výskum botanický SPR Kečovské skrapy*. Mmsc. depon. in Správa NP Slovensky kras, Brzotin, SK.
Karasová, E. 2004. *Inventarizačný výskum botanický PR Zemné hradisko*. Mmsc. depon. in Správa NP Slovensky kras, Brzotin, SK.
Karasová, K. 1996. *Inventarizačný výskum botanický NPR Turniansky hradný vrch*. Msc. SAZP-COPK, Správa NP Slovenský kras, Brzotín, SK.
Kárpáti, I. & Kárpáti, V. 1965. Contribution to the ecology of the steppe vegetation of the Tihany peninsula I. Description of the sample area and of the plant coenosis anlysed. *Annal. Biol. Tihany* 32: 265–274.
Karrer, G. 1985a. Die Vegetation des Peilsteins, eines Kalkberges im Wienerwald. *Verh. Zool.-Bot. Ges. Österreich* 123: 331–414.
Karrer, G. 1985b. Waldgrenzstandorte an der Thermenlinie (Niederösterreich). *Stapfia* 14: 85–103.
Klika, J. 1926. Poznámky ke geobotanickému prùzkumu Velké Fatry. *Sborník přírodovìdecké společnosti v Moravské Ostravì* 3: 38–85.
Klika, J. 1929a. Botanicko-sociologický náčrt Sulovských kopcú. *Sborník přírodovìdecké společnosti v Moravské Ostravì* 5: 49–71.
Klika, J. 1929b. Zweiter Beitrag zur geobotanischen Durchforschung der Hohen Fatra (Velká Fatra). Die Felsen- und Hanggesellschaften I. *Preslia* 8: 33–50.
Klika, J. 1931. Studien über die xerotherme Vegetation Mitteleuropas I. Die Pollauer Berge im südlichen Mähren. *Beih. Bot. Cbl.* 47B: 343–398.
Klika, J. 1932. Der Seslerion coeruleae-Verband in den Westkarpathen. *Beih. Bot. Cbl.* 49B: 133–175.
Klika, J. 1936. Ekologická a sociologická studie pastvin vápencové a dolomitové západokarpatské hornatiny (čachtické kopce). I. Sociologický rozbor. *Sb. Čs. Akad. Zemìd., Praha,* 11: 330–336.
Klika, J. 1937. Xerotherme und Waldgesellschaften der Westkarpathen (Brezover Berge). *Beih. Bot. Cbl*. 57B: 295–342.
Klika, J. 1938. Xerotherme Pflanzengesellschaften der Kováčover Hûgel in der Südslowakei. *Beih. Bot. Centralbl*. 58B: 435–465.
Klika, J. 1945. O vlivu pastvy na rostlinná společenstva krasového území (Fytosociologický příspìvek). *Příroda, Brno*, 37/10: 297–301.
Kliment, J. 1978. *Rastlinstvo vápencov medzi strednými tokmi riečok Muráň a Blh*. Dipl. Pr. (msc.) depon in PriF UK Bratislava, SK.
Kliment, J. 1998. Niekoľko zaujímavejsích floristických nálezov v Drienčanskom krase. *Bull. Slov. Bot. Spoločn., Bratislava,* 20: 151–158.
Kliment, J. 2002. Lemové spoločenstvá s *Geranium sanguineum* v horskom stupni Lúčanskej a Veľkej Fatry. *Bull. Slov. Bot. Spoločn., Bratislava*, 24: 201–207
Kliment, J. & Bernátová, D. 2000. Asociácia Orthantho luteae-Caricetum humilis v Turčianskej kotline. *Kmetianum, Zb. SNM-Múzea A. Kmeťa, Martin,* 9: 53–68.
Kliment, J., Hrivnák, R., Jarolímek, I. & Valachovič, M. 2000. Nelesné spoločenstvá Drienčanského krasu. In: Kliment J. (ed.) *Príroda Drienčanského krasu*. SOP SR, Banská Bystrica, pp. 155-190.
Klimes, L. 1983. *Stepní a luční vegetace východního okraje Drahanské vrchoviny*. Ms. dipl. pr., depon. PřF UP, Olomouc, CZ.
Knapp, R. 1944. *Die Trockenrasen und Felsfluren der Hainburger Berge*. Manuskript, Halle (Saale), DE.
Kochjarová, J. 1997. Poznámky k rozsíreniu, cenol\ogii a ohrozenosti populácií zástupcov rodu Tephroseris (Rchb.)Rchb. na Slovensku I. *Bull. Slov. Bot. Spoločn., Bratislava*, 19: 50–61.
Kochjarová, J. 1998a. Poznámky k rozsíreniu, cenologii a ohrozenosti populácií zástupcov rodu Tephroseris (Rchb.) Rchb. na slovensku. II.: T. longifolia subsp. moravica v Západných Karpatoch. *Bull. Slov. Bot. Spoločn., Bratislava*, 20: 69–77.
Kochjarová, J. 1998b. *Rod Tephroseris (Rchb.) Rchb. v geografickom priestore Západných Karpát (taxonomicko-chorologická stúdia)*. Kand. dizert. práca (msc), Blatnica, SK.
Kochjarová, J. 2002. Druhová diverzita cievnatých rastlín horských lúk a pasienkov vo Veľkej Fatre (predbezná analýza na vybraných lokalitách Lysec a čremosné). *Matthias Belivs. Univ. Proc.,* Suppl. 1: 43–56.
Kochjarová, J. 2009. Floristicko-fytocenologická analýza vybraných lúčno-pasienkových biotopov na severovýchodnom okraji Veľkej Fatry (Kopa - Hubová - černovské lúky). *Bull. Slov. Bot. Spoločn., Bratislava,* 31: 35–58.
Kochjarová, J. & Valachovič, M. 2006. Krovinová a lemová vegetácia ekotonových stanovísť Muránskej planiny. *Reussia* 3/2: 71–114.
Kochjarová, J., Marhold, K. & Hrouda, L. 1997. Príspevok k flore a vegetácii komplexu Jánskej doliny a Ohnisťa v Nízkych Tatrách. *Preslia* 69: 333–358.
Kochjarová, J., Zaliberová, M., Jarolímek, I., Blanár, D. & Hrivnák, R. 2005. Nové floristické a fytocenologické nálezy z Muránskej planiny a blízkeho okolia. *Bull. Slov. Bot. Spoločn., Bratislava,* 27: 109–120.
Kolbek, J., Boublík, K., Černý, T. & Petřík, P. 2005. Vegetační vazba druhu Cardaminopsis petraea v české republice. *Zpr. čes. Bot. Společ., Praha*, 40: 243–277.
Koó, A.J. 1994. Pflegekonzept für die Naturschutzgebiete des Burgenlandes. *Biol. Forsch. Burgenland* 82: 1–203.
Kornaś, J. 1958. Reliktowa kolonia roślin wysokogórskich w Małych Pieninach. *Ochr. Przyr*. 25: 238-247.
Korotchenko, I.A. & Didukh, Y.P. 1997. Stepova roslinnist pivdennoyi chastyny Livoberezhnogo Lisostepu Ukrayiny. II. Klas *Festuco-Brometea*. [The steppe vegetation of the southern part of the Left-Bank Forest-Steppe of the Ukraine. II. Class *Festuco-Brometea*.] *Ukrainskij fitocenologichnij zbirnik, Ser. A*, 1 (6): 20–39. [in Ukrainian.]

Korotchenko, I.A., Mala, Y.I. & Fitsailo, T.V. 2009. Syntaksonomiya stepovoyi roslynnosti krainiogo pivdnia Pravoberezhnogo Lisostepu Ukrayiny. [Syntaxonomy of steppe vegetation of the extreme south of Forest-Steppe zone of Right Bank Dnieper region of the Ukraine]. *Naukovi zapysky NaUKMA, Ser. Biologiya ta ecologiya,* 93: 54–69. [in Ukrainian.]
Kostuch, R. & Misztal, A. 2007. Roślinność kserotermiczna istotnym elementem bioróżnorodności Wyżyny Małopolskiej. *Woda – Środowisko – Obszary Wiejskie* 7(2b): 99–110.
Kovačević-Viličić, M. 1974. *Flora i vegetacija glavnog kalničkog bila s posebnim obzirom na vegetaciju stijena.* Diplomski rad. Prirodoslovno-matematički Fakultet, Sveučilišta u Zagrebu, Zagreb, HR.
Kovacs, A. & Manoliu, E. 1972. Contribuţii la cunoaşterea florei şi vegetaţiei din împrejurimile satului Corpadea (judeţul Cluj). *Contribuţii Botanice*: 223–235.
Kovács, J.A. 2001. A gyepvegetáció sajátosságai Erdélyben. *Kanitzia* 9: 85–150.
Kovács, J.A. 2003. Meso-xerophilous grassland and fringe communities in the Eastern Part of the Transylvanian Basin. *Kanitzia* 11: 97–126.
Kovács, M. & Máthé, I. 1964. A mátrai flórajárás (Agriense) sziklavegetációja. *Bot. Közlem*. 51: 1–18.
Kozłowska, A. 1928. Naskalne zbiorowiska roślin na wyżynie Małopolski. Rozprawy Wydziału matem.-przyrod. *Polskiej Akademii Umiejętności* LXVII: 325–373.
Krajina, V. 1936. Nová nalezistì rumenice turňanské. *Vìda přír., Praha*, 17: 18–20.
Krippel, E. 1954. Rozsírenie zlatej brady (Chrysopogon gryllus Trin.) na Slovensku. *Biologia* 9: 248–262.
Krippelová, T. 1967. Vegetácia Zitného ostrova. *Biol. Pr., Bratislava*, 13: 1–108.
Krippelová, T. 1968. *Pôvodné lesné spoločenstvá Malých Karpát a Myjavskej pahorkatiny*. Záv. správa (msc.), depon in OG BU SAV, Bratislava, SK.
Krstonošić, D. 2013. *Sukcesija vegetacije na mezofilnim i kserofilnim travnjacima Slavonskoga gorja*. Doktorska disertacija, Šumarski fakultet, Sveučilište u Zagrebu, Zagreb, HR.
Kubíková, J. & Kučera, T. 1999. Diverzita vegetace Bílých Karpat na příkladu Předních luk a okolí. *Sborn. Přírod. Klubu Uherské Hradistì* 4: 19–58.
Kusák, P. 1996. Luční porosty na severním okraji mìsta Zlín. *Acta Muzealia, Muzeum jihovýchodní Moravy ve Zlínì* B 8/96: 1-43.
Kuyper, T.W., Leewenberg, H.F.M. & Hübl, E. 1978. Vegetationskundliche Studie an Feucht-, Moor- und Streuwiesen im Burgenland und östlichen Niederösterreich. *Linz. Biol. Beitr.* 10: 231–321.
Kuzemko, A. 2009. *Roslynnist' Ukrayiny. Luchna roslynnist'. Klas Molinio-Arrhenatheretea* [*Vegetation of Ukraine. Meadow Vegetation. Molinio-Arrhenatheretea class*]. Phytosociocentre, Kyyiv, UA. [in Ukrainian.].
Kuzemko, A. 2011. Stepova i luchna roslynnist dolyny richky Girskyi Tikych [Steppe and meadow vegetation of the Girskyi Tikych River valley]. *Visnyk Donetskogo Natsionalnogo universytetu, Ser. A,* 1: 141–150. [in Ukrainian].
Kuzemko, A., Becker, T., Didukh, Y.P., Ardelean, I.V., Becker, U., Beldean, M., Dolnik, C., Jeschke, M., Naqinezhad, A., (...) & Jürgen Dengler, J. 2014. Dry grassland vegetation of Central Podolia (Ukraine) – a preliminary overview of its syntaxonomy, ecology and biodiversity. *Tuexenia* 34: 391–430.
Láfersová-Jamrichová, J. 1981. *Skalné a nelesné spoločenstvá Hájskej doliny*. Dipl. práca (msc.).
Leputsch, S. 1997. *Die Wiesen des Lainzer Tiergartens unter besonderer Berücksichtigung der Jagd-Tradition und der Erholungsnutzung*. Diplomarbeit, Univ. f. Bodenkultur, Wien, AT.
Magic, D. 1969. *Sprievodná správa k listu M-34-124-C (Cinobaňa).* Msc. depon in odd. geobotaniky BU SAV Bratislava, SK.
Maglocký, S. 1970. Carici (humilis)-Seslerietum calcariae Sillinger 1930. *Biológia* 25: 709–722.
Maglocký, S. 1973. Ranunculo (illyrici)-Festucetum valesiacae Klika 1931 p.p. (Festuca vallesiaca-Ranunculus illyricus Klika 1931) v Povazskom Inovci. *Botan. Práce, Bratislava*, 35–55.
Maglocký, S. 1979. Xerotermná vegetácia v Povazskom Inovci. *Biol. Pr., Bratislava*, 25/3: 5–129.
Maglocký, S. 1982. In: Spániková A. (ed.), Vegetácia Východoslovenskej a Záhorskej níziny. *Acta Bot. Slov. ser. A*, 6.
Májovský, J. 1954. Geobotanické pomery Kapusianských kopcov (okres Presov). *Biologia* 9: 144–165.
Májovský, J. 1955. Asociácia Festuca pseudodalmatica-Potentilla arenaria na východnom Slovensku. *Biologia* 10: 659–677.
Májovský, J. 1958a. Spoločenstvá s psinčekom obyčajným (Agrostis vulgaris) na Krupinskej vrchovine. *Acta Fac. Rer. Natur. Univ. Comen., Botanica, Bratislava*, 2: 267–283.
Májovský, J. 1958b. Poznámky ku floristike Bratislavy. *Acta Fac. Rer. Natur. Univ. Comen., Botanica, Bratislava* 2: 369–372.
Májovský, J. & Jurko, A. 1956. Asociácia Festuca pseudodalmatica-Inula oculus-christi na juznom Slovensku. *Biologia* 11: 129–145.
Májovský, J. & Jurko, A. 1958. Xerotermné spoločenstvo s Festuca pseudodalmatica a jeho syngenetická stúdia v doline Hrona. *Acta Fac. Rer. Natur. Univ. Comen., Bot.* 2: 285–311.
Majzlanová, E. 1981. Wiesen- und Weidengesellschaften des westlichen Teiles der Kleinen Karpaten. *Acta Fac. Rer. Natur. Univ. Comen., Botanica*, 28: 37–58.
Majzlanová, E. & Somsák, L. 1991. Súčasný stav vegetácie blízkeho okolia kameňolomu Obyce. *Rosalia, Nitra*, 7: 73–80.
Mann, M. 1997. *Die Verbreitungsmuster der Gefäßpflanzen des Gemeindegebietes von Gaaden in Niederösterreich und ihre standörtlich-vegetationskundlichen Grundlagen*. Dissertation, Universität Wien, AT.
Margl, H. 1973. Pflanzengesellschaften und ihre standortsgebundene Verbreitung in teilweise abgedämmten Donauauen (Untere Lobau). *Verh. Zool.-Bot. Ges. Österr.* 113: 5–51.
Miadok, D. 1987. Phytozonologisches Material aus den Waldsteppen des Koniar Plateaus und des Karstgebiet Jelsavsky kras. *Acta Fac. Rer. Nat. Univ. Comen., Bratislava*, 34: 93–111.
Michalik, S. 1999. Sorbus intermedia (Rosaceae) - nowy gatunek we florze Ojcowskiego Parku Narodowego. *Fragm. Flor. Geobot. Polon*. 6: 265–268.
Michalko, J. 1957. *Geobotanické pomery pohoria Vihorlatu*. Vyd. SAV, Bratislava, SK.
Michalko, J. & Dzatko, M. 1965. Fytocenologická a ekologická charakteristika rastlinných spoločenstiev lesa Dubník pri Seredi. *Biol. Pr., Bratislava*, 11/5: 47–113.
Michálková, D. 2003. *Vegetácia a flora vrchu Rohatín, Strázovské vrchy*. Dipl. Pr., (msc.) depon in PrFUK Bratislava, SK.
Michálková, D. 2006. Diverzita prirodzených rastlinných spoločenstiev vrchu Rohatín v Strázovských vrchoch. *Bull. Slov. Bot. Spoločn., Bratislava*, Suppl. 2 (14): 59–90.
Michálková, D., Skodová, I. & Mertanová, S. 2006. Príspevok k fytocenológii xerotermných rastlinných spoločenstiev v Povazskom Inovci. In: Rajcová, K. (ed.), *Najvzácnejsie prírodné hodnoty Tematínskych vrcho*v. KOZA, Pre Prírodu, Trenčín, 2006: 35–44.
Mikyska, R. 1933. Vegetationsanalyse nebst einigen ökologischen Beobachtungen auf dem Berge Holík im Stiavnické středohoří (Schemnitzer Mittelgebirge). *Beih. Bot. Centralbl*. 51/II: 354–373.
Milka, D. 1973. *Rostlinná společenstva aluviální nivy Dyje mezi Novými mlýny a Podivínem*. Ms. dipl. pr., depon. in Knih. kat. bot., Přír. Fak. MU, Brno, CZ.
Mirek, Z. .1978. *Brassica elongata* Rhrh. - nowy gatunek we florze Polski. *Fragm. Flor. Geobot.* 24: 373–382.

Mochnacký, S. & Maglocký, S. 1993. Plant communities of the Trifolio-Geranietea sanguinei Th. Müller 1961 in East Slovakia. *Thaiszia* 3: 101–110.
Mucina, L. 1986. Vegetation dynamics within the complex of xeto-thermophilous plant communities on a riverine gravel terrace. *Proceedings* C 89/4: 405–424.
Nejezchlebová, M. 2009. *Vegetace suchých trávníkù Moravského krasu ve vztahu k abiotickým faktorùm prostředí*. Ms. Dipl. pr. depon in knih Úst. Bot a zool., Přir. Fak. MU, Brno, CZ.
Neuhäusl, R. & Neuhäuslová-Novotná, Z. 1964. Vegetationsverhältnisse am Südrand des Schemnitzer Gebirges. *Biol. Pr., Bratislava*, 10/4: 5–76.
Neuhäuslová-Novotná, Z. 1968. Beitrag zu den floristisch-phytozönologischen Verhältnissen der Gegend von Lučenec. *Biol. Pr., Bratislava*, 14: 1–70.
Niklfeld, H. 1964. Zur xerothermen Vegetation im Osten Niederösterreichs. *Verh. Zool.-Bot. Ges. Wien* 103/104: 152–181.
Nowak, E. 2003. *Ausgesuchte Wiesen im Gütenbachtal - Vegetationsanalyse und Pflegekonzept.* Diplomarbeit, Univ. f. Bodenkultur, Wien, AT.
Parabućski, S. 1990. Neke odlike dolinskih livada na području Vojvodine. *Zbornik Matice srpske za prirodne nauke* 79: 107–118.
Parabućski, S. & Butorac, B. 1993. *Stepska vegetacija severoistočne Bačke*. Glasnik Instituta za botaniku i Botaničke bašte Univerziteta u Beogradu, Tom XXIV-XXV, 1990-1991 (1993), Beograd, RS.
Parabućski, S., Stojanović, S. & Vučković, M. 1986. Zajednica Festucetum vaginatae danubiale Soo 1929. *Zbornik Matice srpske za prirodne nauke* 70: 129–134.
Pavlíková, H. 1981. *Xerotermná vegetácia juzných svahov Horného vrchu v Slovenskom krase.* Dipl. Pr. (msc.) depon in PrírFUK Bratislava, SK.
Perný, M. 1999. *Flóra a vegetácia Chocholanskej doliny (Biele Karpaty)*. Dipl. Pr., (msc.), depon in Kat. botaniky, Bratislava, SK.
Petkovsek, V. 1977. Travna zdruzba Bromo-Brachypodietum pinnati assoc. nova v Sloveniji. *Razprave* 20(3): 197–214.
Petkovsek, V. 1978. Travniska zdruzba Bromo-Danthonietum calycinae Sugar 1972 in njena razsirjenost v Sloveniji. *Mitt. Ostalpin-dinarischen Ges. f. Vegetationskunde* 14: 271–277.
Petrík, A. 1978. Skalné spoločenstvá. In: Pitoniak P. et al., Flóra a vegetácia Chránenej krajinnej oblasti Slovenský raj. *Biol. Práce, Bratislava*, 24:/6: 68–81.
Petrík, A. 2006. *Program záchrany druhu Onosma tornensis Jáv*. Záv. správa (msc.), Bratisalava, depon in Správa NP Slovenký kras, Brzotín, SK.
Petrík, A. et al. 1982. Geobotanické mapovanie štátnej prírodnej rezervácie Sokol v CHKO Slovenský raj. *Ochr. Prír*. 3: 207–227.
Petrinec, V. 1999. *Vegetationsmonographie von Sturmovci (NO-Slowenien).* Diplomarbeit, Univ. Wien, AT.
Pfusterschmid, S. 1998. *Die Trockenrasengesellschaften der westlichen Steilhänge des Bisamberges bei Wien.* Diplomarbeit, Univ. f. Bodenkultur, Wien, AT.
Pietorová, E. 1996. *Rastlinné spoločenstvá NPR Maninská tiesňava.* In: Kolektív, Mainská tiesòava (výsledky inventarizaèného výskumu), SAZP Zilina, Správa CHKO Strázovské vrchy Povazská Bystrica.
Piwowarczyk, R. & Przemyski, A. 2010. The distribution and habitat preferences of the declining species *Orobanche arenaria* Borkh. at the northern limit of its geographical range. *Acta Soc. Bot. Polon*. 79: 43–50.
Pöcheim, S. 2004. *Grünlandgesellschaften des Südburgenlandes in Beziehung zu Landnutzung; und Landschaftsentwicklung*. Dissertation, Univ. Bodenkultur, Wien, AT.
Pohoriljaková, I. 1992. *Floristické a fytocenologické zhodnotenie lúčnych porastov v Drietomskej doline (Biele Karpaty)*. Dipl. práca msc. depon. in: PriFUK Bratislava, SK.
Poldini, L. 1989. *La vegetatione del Carso isontino e triestino*. Lint, Trieste, IT.
Pop, A. 1996. Floristisch-ökologische Bemerkungen zur Vegetation der Klausenburger Berge. *Stapfia* 45: 103–134.
Pop, I., Csűrős, I., Kovacs, A., Hodişan, I. & Moldovan, I. 1964. Flora şi vegetaţia Cheilor Runc (Reg. Cluj, Raion Turda). *Contribuţii Botanice* 205–224.
Pop, I., Hodişan, I., Raţiu, O. & Pall, Ş. 1960. Vegetaţia masivelor calcaroase de la Cheile Intregalde şi Piatra Caprii. *Contribuţii Botanice* 195–219.
Pospíšil, V. 1954. Nástin vegetace přírodních reservací u Hranic a jejich geobotanický význam. *Ochr. Přír*. 9: 165–168.
Pospíšil, V. 1957a. Výspa pannonské kvìteny ve Vsackých horách. *Ochr. Přír*. 12: 129–135.
Pospíšil, V. 1957b. "Lhotka", lokalita xerothermní kvìteny na jihozápadních výbìzcích Oderských hor. *Ochr. Přír*. 12: 281–285.
Pospíšil, V. 1962. Jak pronikaly thermofyty do nitra severozápadních Karpat. *Čas. Morav. Mus. Brno* 47: 69–108.
Pospíšil, V. 1964. Die Mährische Pforte, eine pflanzengeographische Studie. *Čas. Morav. Mus. Brno* 49: 103–190.
Procházková, L. 1996. *Floristické pomìry nelesní vegetace Hostýnských vrchù*. Ms. dipl. pr., depon. PřF UK, Praha, CZ.
Purger, D. 1993. *Vegetacija u okolini Doroslova (zapadna Bačka)*. Magistarski rad, Univerzitet u Novom Sadu, Prirodno-matematički fakultet, Novi Sad, RS.
Ráčilová, M. 1964. *Rostlinná společenstva hadcové stepi u Mohelna*. Ms., depon. PřF MU, Brno, CZ.
Rafajová, M. 1998. *Přirozená a polopřirozená vegetace údolí Jevisovky*. Ms. dipl. pr., depon. in Knih. kat. bot., Přír. Fak. MU, Brno, CZ.
Rathmayer, E. 1985. *Die Vegetation des Naturschutzgebietes Eichkogel bei Mödling und die Problematik der Erhaltung menschlich bedingter, seltener Vegetationstypen*. Diplomarbeit, Univ. f. Bodenkultur, Wien, AT.
Raţiu, O., Kovács, A. & Silaghi, G. 1969. Fitocenoze caracteristice imprejurimilor Blajului. *Contribuţii Botanice* 169–189.
Regula-Bevilacqua, L. 1978. *Biljni pokrov Strahinščice u Hrvatskom zagorju*. Disertacija, Sveučilište u Zagrebu, Prirodoslovno-matematički fakultet, Zagreb, HR.
Regula-Bevilaqua, L. 1983. Onobrychido-Brometum (Scherrer 1925) Th. Müller 1966 in Nordwest-Kroatien. *Acta Bot. Croat*. 42: 83–87.
Řehořek, V. 1969. *Udolné lúky nízinného stupňa v povodí Slanej*. KDP, Kat. bot. VSP v Nitre, SK.
Reichenberger, G. 1990. *Das Naturschutzgebiet Glaslauterriegel-Heferlberg. Vegetation und Struktur*. Diplomarbeit, Univ. f. Bodenkultur, Wien, AT.
Removčíková, O. 1981. *Lúky a pasienky východnej časti Slovenského krasu*. Dipl. Pr. (msc.) depon in PrírFUK Bratislava, SK.
Resmeriţa, I. Spirchez, Z. & Csűrős, I. 1967. Vegetaţia nisipurilor din nord-vestul Romaniei. *Contribuţii Botanice* 349–361.
Rotter, D. 2002. *Einfluß der Heißländen-Sukzession auf Arten und Artengemeinschaften (Blütenbesucher, Bodenarthropoden) der Unteren Lobau*. Dissertation, Univ. Wien, AT.
Rötzer, H. 2004. *Die Entwicklung der pannonischen Steppenlandschaft und der sie bestimmenden gesellschaftlichen Werthaltungen am Beispiel des österreichischen Marchfeldes.* Dissertation, Univ. Bodenkultur, Wien, AT.
Ruprecht, E., Szabo, A., Enyedi, M.Z. & Dengler, J. 2009. Steppe-like grasslands in Transylvania (Romania): characterization and influence of management on species diversity and composition. *Tuexenia* 29: 353–368.
Ruzičková, H. 1971. Rastlinné spoločenstvá lúk a slatín v povodí čiernej vody (Východoslovenská nízina). *Biol. Pr., Bratislava*, 17: 1–131.

Ruzičková, H. 1986. Trávnaté porasty Liptovskej kotliny. *Biol. Pr., Bratislava*, 32: 1–138.
Ruzičková, H. 1987. Lesné lúky juznej časti Slánskych vrchov. *Biologia* 42: 487–496.
Ruzičková, H. 1997. Sadové lúky myjavsko-bielokarpatských kopaníc a ich význam pre ochranu prírody na Slovensku. *Ochr. prír., Banská Bystrica*, 15: 83–94.
Ruzičková, H. 2002. Species-rich meadows of the Starohorské vrchy Mts and south-eastern parto of the Veľká Fatra Mts - a relict of the extensive and semi-intensive agriculture of the Central Western Carpathians. *Biológia* 57: 493–504.
Ruzičková, H., Dobrovodská, M. & Valachovič, M. 1999. Landscape-ecological evaluation of vegetation in relation to the forms of anthropogenic relief in the cadastre of Liptovská Teplička village, the Nízke Tatry Mts. *Ekológia* 18: 381–400.
Ruzičková, H. & Halada, L. 2005. Orchard meadows of Banská Stiavnica town (Central Slovakia). *Polish Botanical Studies* 19.
Ruzičková, H. & Michalko, J. 1982. Wiesen im Vihorlat-Gebirge. *Biologia* 37: 49–58.
Ruzičková, J. 1982. *Lemové a plástové spoločenstva Drieňovca v Slovenskom krase*. Dipl. práca (msc.) depon. in PriFUK Bratislava, SK.
Sádovský, M. 2004. Nové poznatky o rozsírení a náčrt cenologických väzieb hviezdovca bodkovaného (Galatella punctata) na Slovensku. *Rosalia, Nitra*, 17: 3–12.
Sádovský, M. & Eliás, P. ml. 2003. Súčasné poznatky o výskyte Iris spuria L. na Slovensku. *Ochr. Prír., Banská Bystrica*, 22: 5–11.
Sanda, V. Barabaş, N. & Biţă-Nicolae, C. 2005. *Breviar privind parametrii structurali şi caracteristicile ecologice ale fitocenozelor din România*. Editura Ion Borcea, Bacău, RO.
Sauberer, N. & Buchner, P. 2001. Die Trockenrasen-Vegetation des nördlichen Steinfeldes. *Stapfia* 77: 113–128.
Schneeweiss, G.M., Schönswetter, P., Tremetsberger, K. & Schratt-Ehrendorfer, L. 2002. Vegetation. In: Wiesbauer H. (ed.), *Naturkundliche Bedeutung und Schutz ausgewählter Sandlebensräume in Niederösterreich*, pp. 15-58. Amt der NÖ Landesregierung, St. Pölten, AT.
Schneider, E. 1997. Die Blaugras-Gesellschaften im Hügelland Siebenbürgens. In: Heltmann, H. & Wendelberger, G. (eds.), *Naturwissenschaftliche Forschungen über Siebenbürgen. V. Beiträge zur Flora, Vegetation und Fauna von Siebenbürgen*, pp. 107–131. Böhlau Verlag, Wien, AT.
Schneider-Binder, E. 1970. Aspecte din flora şi vegetaţia conglomeratelor Talmaci-Podul Olt (jud. Sibiu). *Studii şi Comunicari, Ştiintele Naturii, Sibiu* 15: 161–168.
Schneider-Binder, E. 1971. Pajiştile xeromezofile din Depresiunea Sibiului şi colinele ei marginale. *Studii şi Comunicari, Ştiintele Naturii, Sibiu* 16: 135–172.
Schneider-Binder, E. 1975. Pajistile xeroterme din ord. *Festucetalia valesiacae* Br.-Bl. et Tx. 1943 in zona colinelor marginale ale depresiunii Sibiului. *Studii şi Comunicari, Ştiintele Naturii, Sibiu* 19: 95–120.
Schneider-Binder, E. 1977. Consideraţii asupra asociaţiilor din alianţa *Stipion lessingianae* Soó 1947 in Romania. *Studii şi Comunicari, Ştiintele Naturii, Sibiu* 21: 91–113.
Schuster, B. 1977. Trockenrasen im Burgenland. *Ber. Biol. Forschungsinst. Burgenl.* 19: 1–40.
Schwarz, F. 1991. *Xerotherme Vegetationseinheiten im Donautal zwischen Engelhartszell und Aschach (Oberösterreichischer Donaudurchbruch).* Dissertation, Univ. Wien, AT.
Seda, Z. 1975a. *Inventarizační prùzkum vegetace státní přírodní rezervace "čubernice".* Ms., depon. AOPaK ČR, Praha, CZ.
Seda, Z. 1975b. *Inventarizační prùzkum vegetace státní přírodní rezervace "Dolní Vinohrádky.* Ms., depon. AOPaK ČR, Praha, CZ.
Seda, Z. 1975c. *Inventarizační prùzkum vegetačního krytu stát. přírodní rezervace "Bràniska". (Katastr. území Ohrozim, okres Prostìjov).* Ms., depon. AOPaK ČR, Praha, CZ.
Seda, Z. 1975d. *Inventarizační prùzkum vegetačního krytu státní přírodní rezervace "Za hrnčířkou".* Ms., depon. AOPaK ČR, Praha, CZ.
Seda, Z. 1976a. *Inventarizační prùzkum vegetace státní přírodní rezervace "Drázov".* Ms., depon. AOPaK ČR, Praha, CZ.
Seda, Z. 1976b. *Inventarizační prùzkum vegetačního krytu státní přírodní rezervace "Oulehla".* Ms., depon. AOPaK ČR, Praha, CZ.
Šegulja, N. 1974. *Biljni pokrov Vukomeričkih Gorica*. Disertacija, Sveučilište u Zagrebu, Zagreb, HR.
Šegulja, N. 1977. Asocijacija Bromo-Cynosuretum cristati na području Vukomeričkih gorica. *Poljopr. znan. smotra* 40 (50): 207–212.
Seljak, G. .1976. *Travniška vegetacija Porezna*. Diplomska naloga, Biotehniška fakulteta, Ljubljana, SL.
Semenishchenkov, Y.A. 2009. *Fitocenoticheskoye raznoobraziye Sudost'-Desnianskogo mezhdurechiya* [*Phytocoenotic diversity of the Sudost-Desna Interfluve Area*]. RIO BGU, Bryansk, RU. [in Russian.]
Sendtko, A. 1997. Die Xerothermvegetation brachgefallener Rebflachen im Raum Tokaj (Nordost-Ungarn); pflanzensoziologische und populationsbiologische Untersuchungen zur Sukzession. *Phytosociologia* 29: 345–448.
Shevchyk, V.L., Solomaha, V.A. & Voityuk, Yu.O. 1996. Syntaksonomiya roslynnosti ta spysok flory Kanivskogo pryrodnogo zapovidnyka [The Syntaxonomy of vegetation and list of the flora of Kaniv Natural Reserve]. *Ukrainskij fitocenologichnij zbirnik*, *Ser. B*, 1: 1–120. [in Ukrainian.].
Sibík, J. (ed.) 2011. Zaujímavejsie fytocenologické zápisy. *Bull. Slov. Bot. Spoločn., Bratislava,* 33/1.
Sillinger, P. 1929. Bílé Karpaty. Nástin geobotanických pomìrù se zvlástním zřetelem ke společenstvùm rostlinným. *Rozpr. Král. čes. Společ. Nauk, cl. math.-natur.,* 8/3: 1–73.
Sillinger, P. 1931. Vegetace Tematínských kopcù na západním Slovensku. Příspìvek k fytogeografii a fytosociologii vápencových obvodù v jihozápadních výbìzcích karpatských. *Rozpr., Tř. Čes. Akad*. 40/13: 1–46.
Sillinger, P. 1933. *Monografická studie o vegetaci Nízkych Tater*. Orbis, Praha, CZ.
Simeková, J. & Pitoniak, P. 1976. Reliktná lokalita Dryas octopetala L. pri Vernári v Slovenskom Raji. *Biológia* 31: 783–793.
Simon, T. 1964. Entdeckung und Zönologie der Festuca dalmatica (Hack.) Richt. in Ungarn und ihr statistischer Vergleich mit ssp. pseudodalmatica (Kraj.) Soó. *Ann. Univ. Budapest, sect. Biol*. 7: 143–156.
Simon, T. 1977. *Vegetationsuntersuchungen im Zempléner Gebirge*. Akadémiai Kiadó, Budapest, HU.
Siroki, Z. 1956. *A Debreceni Mezogazdasagi Akademia nyulasi legelojenek botanikai osszetetele.* Debreceni Mezogazdasagi Akademia Evkonyve, pp. 183–205.
Sitásová, E. 1999. Vegetačné pomery Prírodnej rezervácie Slanský hradný vrch. *Natura Carpatica* 40: 73–82.
Sitásová, E. 2000. Vegetačné pomery v Národnej Prírodnej Rezervácii Humenec. *Natura Carpatica* 41: 127–144.
Sitásová, E. 2002. Vegetačné pomery lokality Hradová pri Kosiciach. *Natura Carpatica* 43: 55–66.
Sitásová, E. & Kaduková, J. 1997. Vegetačné pomery skál pri Trebejove. *Natura Carpatica* 38: 23–36.
Six, U. 1986. *Die Vegetation der Perchtoldsdorfer Heide*. Diplomarbeit, Univ. f. Bodenkultur, Wien, AT.
Škodová, I., Hegedüšová, K. & Valachovič, M. 2005. Rastlinné spoločenstvá Vrchnej hory pri Stupave. *Ochr. prír*. 24: 72–86.
Skolek, J. 2004. Skalné spoločenstvá Festucetum tatrae Szafer et al. 1923 v Národnej prírodnej rezervácii Demänovská dolina. *Natura tutela, Liptovský Mikulás*, 8: 47–54.
Škornik, S. 2000. *Suha in polsuha travisča reda Brometalia erecti Koch 1926 v Sloveniji*. Disertacija, Univerza v Ljubljani, Ljubljana, SL.
Škornik, S. 2003. Suha travisča reda Brometalia erecti Koch 1926 na Goričkem (SV Slovenia). *Hacquetia* 2: 71–90.

Škovirová, K. & Očka, S. 2005. Floristický príspevok k Dielniciam pri Klástore pod Znievom. *Zborník SNM, Kmetianum, Martin*, 10: 105–127.
Šmarda, J. 1942. Mykologická pozorování na Klucaninì u Tisnova. *Příroda (Brno)* 35: 50–51.
Šmarda, J. 1961. *Vegetační pomìry Spisské kotliny. Studie travinných porostu*. Vyd. SAV, Bratislava, SK.
Šmarda, J. 1967. Vegetační pomìry Moravského krasu. (Příspìvek k řesení bioindikace krasového reliéfu). *Část I. Čs. Ochr. Prír*. 3: 139–168.
Šmarda, J. 1970. Flora a vegetace Slovenského ráje. *Pr.Stud. Cs. Ochr. Prir. SUPSOP, Bratislava,* 4: 5–43.
Šmarda, J. 1975. Rostlinná společenstva skalnaté lesostepi Pavlovských kopcù na Moravì (ČSSR). *Čs. Ochr. Prír*. 14: 5–58.
Šmarda, J. & Šmarda, J. 1968. Charakteristika význačných lokalit v Moravském Krasu. *Čs. Ochr. Prír.* 7: 111–137.
Šmeralová, Z. 1980. *Vegetační pomìry navrhovaného chrànìného území Hornek*. Ms., depon. CHKO Moravský kras, Blansko, CZ.
Šmiták, J. & Grüll, F. 1984. *Chrànìný přírodní výtvor červený kopec. Inventarizační prùzkum*. Ms., depon. AOPaK ČR, Brno, CZ.
Soó, R. 1944. A Székelyföld növényszövetkezeteiről. Über die Pflanzengesellschaften des Seklerlandes. *Múzeumi Füzetek* II, 12.
Soó, R. 1949. Les associations végétales de la Moyenne-Transylvanie. II. Les associations des marais, des prairies et des steppes. *Acta Geobotanica Hungarica* 6: 3–107.
Stavělová, E. 1976. *Vegetační poměry v povodí Pasovického potoka*. Ms. dipl. pr., depon. in Knih. kat. bot., Přír. Fak. MU, Brno. CZ.
Stevanović, V. 1984. *Ekologija, fitocenologija i floristička struktura stepske vegetacije Fruške gore.* Doktorska disertacija, Univerzitet u Beogradu, Prirodno-matematički fakultet, Odsek za biološke nauke, Beograd, RS.
Stjepanović-Veselčić, L. 1953. *Vegetacija Deliblatske peščare*. Nauč. Kńiga, Beograd, RS.
Stojanović, S. 1981. *Vegetacija Titelskog brega*. Doktorska disertacija, Univerzitet u Novom Sadu, Prirodno-matematički fakultet, Novi Sad, RS.
Šugar, I. 1972. *Biljni svijet Samoborskog gorja*. Disertacija, Sveučilište u Zagrebu, Zagreb, HR.
Šumberová, K. 1995. Stepní lokalita Zimarky u Velkých Bílovic. *Zpr. Čes. Bot. Společ*. 30: 9–12.
Suňalová, K. 1999. *Flora a vegetácia lokalít Babiná a Krivoklátske lúky v Bielych Karpatoch*. Dipl. Pr., (msc.) depon in kat. pedol. PrF UK Bratislava, SK.
Şuteu, Ş. 1968. Vegetaţia ierboasa de stancarie din Cheile Rameţului (jud. Alba). *Contribuţii Botanice* 243–266.
Şuteu, Ş. 1969. *Flora şi vegetaţia Cheilor Rametului*. PhD thesis, Cluj-Napoca, RO.
Şuteu, Ş. 1979. Cercetari de vegetaţie pe Coasta Almaşului (Tirimia-jud. Mureş). *Contribuţii Botanice* 143–154.
Suza, J. & Zlatník, A. 1928. Sociologické snímky. In: Suza J., Geobotanický prùvodce serpentinovou oblastí u Mohelna na jihozápadní Moravě. *Rozpravy II. třídy České akademie věd* 37, č. 31.
Svandová-Ursíniová, Ľ. 1966. *Skalné spoločenstvá a spoločenstvá plytkých vápencových pôd Chočského pohoria.* Diplomová práca (msc.), depon in. PríF UK Bratislava, SK.
Szabo, T.A. & Galan, P. 1966. Vegetaţia terenurilor erodate din regiunea Saratel. *Contribuţii Botanice* 103–115.
Szaboóvá-Baxandale, I. 1994. *Zhodnotenie stavu prírodného prostredia SPR Zádielska tiesňava na základe synantropizácie fl'ory vplyvom náučného chodníka*. Dipl. Pr. (msc.) depon in PriF UK Bratislava, SK.
Takács, A.A. & Takácsné Kovács, A. 1999. A sárszentágotai Sós-tó vegetációtérképe. *Bot. Közlem.* 57–66.
Tauber, F. & Weber, P. 1976. Dealul cu bulbuci (Trollius europaeus L.) de langa Mediaş. *Ocrotirea Naturii* 20: 23–34.
Tichý, L. 2006. Diverzita vápencových lomù a moznosti jejich rekultivace s vyuzitím přirozené sukcese na příkladu Rùzenina lomu. *Zpr. Čes. Bot. Společ*. 41, Mater. 21: 89–103.
Tichý, L. & Chytrý, M. 1996. Festuco pallentis-Alyssetum saxatilis na jihozápadní Moravě. *Zpr. Čes. Bot. Společ*. 31: 187–192.
Tichý, L., Chytrý, M., Pokorny-Strudl, M. , Strudl, M. & Vicherek, J. 1997. Wenig bekannte Trockenrasen-Gesellschaften in den Flusstälern am Südostrand der Böhmischen Masse. *Tuexenia* 17: 223–237.
Tlusták, V. 1972. *Xerotermní travinná společenstva lesostepního obvodu Bílých Karpat*. Ms. Dipl. pr., depon in Přir. Fak. MU, Brno, CZ.
Tlusták, V. 1975. Syntaxonomický přehled travinných společenstev Bílých Karpat. *Preslia* 47: 129–144.
Tlusták, V. 1976. *Vegetační pomìry "Malých stran" u Rousínova*. Ms., záver. pr. postgrad. kurzu taxonomie vyssích rostl.
Tlusták, V. 1990. *Ruderální společenstva Olomouce*. Ms., kand. dis. pr., depon. Bot. Ústav AV ČR, Pruhonice, CZ.
Toman, M. 1975. Materiál k fytocenologii společenstev třídy Festuco-Brometea na Pavlovských kopcích (jizní Morava). *Zborn. Ped. Fak. Presov Univ. P. J. Safárika Košice, Prír. Vedy* 14/1: 127–134.
Tomazič, G. 1949. Asociacije borovih gozdov v Sloveniji. III. *Jasast.Razprave* 4: 159–203.
Towpasz, K. & Kotańska, M. 1999. Nowe stanowisko Reseda phyteuma (Resedaceae) w Polsce. *Fragm. Flor. Geobot. Ser. Polonica* 6: 284–286.
Tracey, R. H. 1980. *Beiträge zur Karyologie, Verbreitung und Systematik des Festuca ovina-Formenkreises im Osten Österreichs*. Dissertation, Univ. Wien, AT.
Trávníček, B. 1987. *Fytocenologická studie xerotermních a semixerotermních travinných a bylinných společenstev střední Moravy (Středomoravské Karpaty).* Ms., dipl. pr., depon. PřF UP, Olomouc, CZ.
Trávníčková, V. 1992. *Floristický výzkum ostrùvkù teplomilné vegetace v území severozápadnì od Prostìjova*. Ms., dipl. pr., depon. PřF UP, Olomouc, CZ.
Trinajstić, I., Regula-Bevilacqua, Lj. & Cerovečki, Z. 2006. Fitocenološko-sintaksonomske značajke vrste Sesleria sadleriana Janka u Hrvatskoj. *Agronomski glasnik* 68: 3–12.
Uhliarová, E. 2005. *Trávnaté porasty Javoria, ich produkčné a mimoprodukčné funkcie*. KDP (msc.), Depon in ÚKE SAV, Bratislava, SZ.
Uhlířová, J. 2006. Variabilita a syntaxonómia asociácie Pulsatillo slavicae-Caricetum humilis. *Acta Rer. Natur. Mus. Nat. Slov., Bratislava*, 52: 12–22.
Uhlířová, J. & Bernátová, D. 2002. K flore a vegetácii kopcovitých vyvýsenín Turčianskej kotliny. *Zb. Slov. Nár. Múz., Bratislava*, 58: 44–47.
Uhlířová, J. & Bernátová, D. 2003. Príspevok k flore a vegetácii skalných stanovísť Muránskej planiny. *Acta Rer. Natur. Mus. Nat. Slov., Bratislava*, 49: 55–67.
Uhlířová, J. & Bernátová, D. 2004. A new syntaxonomical view on the association Pulsatillo slavicae-Caricetum humilis. *Annot. Zool. Bot., Bratislava*, 227: 3–12.
Uhlířová, J. & Petrík, A. 2006. Festuco tatrae-Caricetum humilis - a new plant community from the Slovenský raj Mts. *Annot. Zool. Bot., Bratislava*, 228: 3–16.
Uhlmann, J. 1938. *Die Pflanzengesellschaften auf dem Westhang des Bisamberges und ihre Abhängigkeit von der Bodengestalt*. Dissertation, Univ. Wien, AT.
Uhrin, S. 2002. *Vegetácia,návrh manazmentu a ochrana doliny Chotinky-NP Poloniny*. Dipl. Pr. (msc.) depon in PriF UK Bratislava, SK.
Ujházy, K., Vlčko, J. & Uhliarová, E. 2003. Nová lokalita vstavačovitých (Orchis coriophora, O. ustalata, O. morio) na severozápadnom úpätí Poľany. *Bull. Slov. Bot. Spoločn., Bratislava*, 25: 61–68.
Unar, J. 1975. *Xerothermní a subxerothermní vegetace Moravského krasu*. Ms., závìr. zpr., depon. BÚ ČSAV Pruhonice, CZ.

Unar, J. 1979a. *Studie o vegetačních a floristických pomìrech SPR Kočičí kámen*. Ms., depon. AOPaK ČR, Praha, CZ.
Unar, J. 1979b. *Studie o vegetačních a floristických pomìrech SPR Stolová hora*. Ms., depon. AOPaK ČR, Praha, CZ.
Unar, J. 1980. *Xerothermní a subxerothermní bylinné vegetace na vápencových ostrùvcích střední Moravy*. Ms., závìr. zpr., depon. Bot. Úst. AV ČR, Pruhonice, CZ.
Unar, J. 2002. *Vegetační a floristické pomìry NPP Rudické propadání*. Ms., depon. CHKO Moravský kras, Blansko, CZ.
Unar, J. & Grüll, F. 1984. Teucrio chamaedrys-Festucetum rupicolae, eine neue Assoziation aus dem Gebiet des Mährischen Karstes. *Folia Geobot. Phytotax*. 19: 139–155.
Urban, B. 1992. *Die Grünlandvegetation im Raum Furth a.d. Triesting*. Diplomarbeit, Univ. Wien, AT.
Urbanová, V. 1971. *Asociácia Anthoxantho-Agrostietum na území Kysuckej vrchoviny*. Zb. prednások zo zjazdu SBS, Tisovec 5.-11. júl 1970, č. II., BA, 398–415.
Urbanová, V. 1977. *Rastlinné spoločenstvá Kysuckých vrchov*. Kand. dizert. práca (msc.).
Urbanová, V. 2002. Zaujímavejsie floristické nálezy v Zilinskej kotline a v blízkom okolí. *Vlastivedný zborník Povazia* XXI: 117–120.
Urvichiarová, E. 1963. *Vegetačné pomery alúvia rieky Muráň*. Dipl. pr. (msc.), depon in Kat. botaniky PrFUK, Brno, CZ.
Valachovič, M. 1992. *Vegetácia vápencových sutín Západných Karpát*. Kand. dizert. práca (msc.).
Valachovič, M. 2004. Spoločenstvá lemov na Borskej nízine - príklad edaficky vyvolanej variability. *Bull. Slov. Bot. Spoločn., Bratislava*, 26: 193–200.
Valachovič, M. (ed.).1995. *Rastlinné spoločenstvá Slovenska. 1. Pionierska vegetácia*. Veda, vydavateľstvo SAV, Bratislava, SK.
Valachovič, M. & Jarolímek, I. 1994. Rastlinné spoločenstvá s výskytom Daphne arbuscula čelak. na Muránskej planine. *Bull. Slov. Bot. Spoloč., Bratislava*, 16: 75–82.
Válková-Tomisová, D. 1978. *Pasienky Silickej planiny*. Dipl. práca (msc.).
Veselá, I. 2007. *Přirozená a polopřirozená vegetace Vizovické pahorkatiny*. Ms. dipl. pr., depon. in Knih. kat. bot., Přír. Fak. MU, Brno, CZ.
Vicherek, J. 1957a. Kamenná u Staříče, významná lokalita teplobytné kvìteny v severovýchodní Moravì. *Přírod. Sborn. Ostrav. Kraje* 18: 169–182.
Vicherek, J. 1957b. Poznámky ke kvìtenì okolí Nového Jičína. *Přírod. Sborn. Ostrav. Kraje* 18: 400–416.
Vicherek, J. 1959. Scabioso-Phleetum as. nov. a Scabioso-Phleetum peucedanetosum Vicherek subas. nov., společenstva xerothermní kvìteny slezské. *Přírod. čas. Slez*. 20: 13–27.
Vicherek, J. 1967. Pozoruhodné xerotermní rostlinné společenstvo na horní Orave. *Biológia* 22: 143–147.
Vicherek, J. 1975. *Synekologická charakteristika psamofytní vegetace jizní Moravy*. Ms., závìr. zpr., depon. in: BÚ AV ČR Pruhonice, CZ.
Vicherek, J. & Unar, J. 1971. *Fytocenologická charakteristika stepní vegetace jizní Moravy*. Ms., depon. Botanický ústav ČSAV Pruhonice, CZ.
Vozárová, M. 1986. Xerotermné trávovo-bylinné spoločenstvá Zoborskej skupiny Tríbeča. *Zborn. Slov. Nár. Múz., Bratislava*, 32: 3–31.
Vozárová, M. 1990. Asociácia Inulo oculus-christi-Festucetum pseudodalmaticae Májovský et Jurko 1956 v sirsom okolí Mochoviec. *Zb. Slov. Nár. Múz. Prír. Vedy* 36: 15–32.
Vrbek, M. 2005. *Flora i nešumska vegetacija Žumberka*. Disertacija. Sveučilište u Zagrebu, Prirodoslovno-matematički fakultet, Biološki odsjek, Zagreb, HR.
Vučković, M. 1991. *Livadska i livadsko-stepska vegetacija Vršačkih planina*. Matica srpska, Novi Sad, RS.
Vučković, R. 1980. *Zeljasta (močvarna i livadsko-pašnjačka) vegetacija okoline Sečnja. Magistarski rad, Univerzitet u Beogradu, Prirodno-matematički fakultet, Odsek za biološke* nauke, Beograd, RS.
Vynokurov, D.S. 2014a. Syntaksonomiya kserotermnoyi roslynnosti dolyny r. Ingul (klas Festuco-Brometea). Chastyna 1. Petrofitno-stepova roslynnist. [Syntaxonomy of xerothermic vegetation of the Ingul River valley (class Festuco-Brometea). Part 1. Petrophytic steppe vegetation]. *Ukrainian Botanical Journal* 71: 148–160. [in Ukrainian.]
Vynokurov, D.S. 2014b. Syntaksonomiya kserotermnoyi roslynnosti dolyny r. Ingul (klas Festuco-Brometea). Chastyna 2. Luchno-stepova, chagarnykovo-stepova, spravzhniostepova roslynnist. [Syntaxonomy of xerothermic vegetation of the Ingul River valley (class Festuco-Brometea). Part 2. Meadow, shrub and true steppe vegetation]. *Ukrainian Botanical Journal* 71: 537–548. [in Ukrainian.]
Wagner, H. 1941. Die Trockenrasengesellschaften am Alpenostrand. *Denkschr. Akad. Wiss. Math.-Nat. Kl.* 104: 1–81.
Wagner, H. 1950. Das Molinietum coeruleae (Pfeifengraswiese) im Wiener Becken. *Vegetatio* 2: 128–165.
Waitzbauer, W. 1990. Die Naturschutzgebiete der Hundsheimer Berge in Niederösterreich. Entwicklung, Gefährdung, Schutz. *Abh. Zool.-Bot. Ges. Österr*. 24: 1–88.
Willner, W., Jakomini, C., Sauberer, N. & Zechmeister, H. 2004. Zur Kenntnis kleiner Trockenraseninseln im Osten Österreichs. *Tuexenia* 24: 215–226.
Willner, W., Sauberer, N., Staudinger, M., Grass, V., Kraus, R., Moser, D., Rötzer, H. & Wrbka, T. (2013): Syntaxonomic revision of the Pannonian grasslands of Austria – Part II: Vienna Woods (Wienerwald). *Tuexenia* 33: 421–458.
Wójciak, H. & Urban, D. 2011. Diversity of xerothermic grasslands in the Bug River valley (in the neighbourhood of Kryłów and Stare Stulno). *Annales Universitatis Mariae Curie-Skłodowska Lublin - Polonia. Sectio C*, Vol. LXVI, 2.
Wünschová, A. 2003. *Biologie, ekologie a rozsíření Laser trilobum (L.) Borkh., Scrophularia vernalis L. a Iris humilis Georgi subsp. arenaria (Waldst. et. Kit.) Á. et D. Löve na Moravì*. Ms. dipl. pr., depon. in Knih. kat. bot., Přír. Fak. MU, Brno, CZ.
Wurm, G. 1991. *Untersuchungen auf gelenkten Brachen zur Entwicklung von Weiderasen im pannonischen Raum.* Diplomarbeit, Univ. Wien, AT.
Záborský, J. 1971. Trifolium diffusum Ehrh., neue Art der Flora der Slowakei und Bemerkungen zu deren Karyologie. *Acta Fac. Rer. Natur., Univ. Comen., Bratislava*, 19: 237–248.
Zahirović, Ž. 2000. *Rijetke i ugrožene biljne vrste sjeveroistočne Hrvatske*. Magistarski rad, Prirodoslovno-matematički fakultet, Sveučilište u Zagrebu, Zagreb, HR.
Zahradníková-Rosetzká, K. 1965. Geobotanická charakteristika slatinných lúk a pasienkov (Molinion Koch 1926) na Zitnom ostrove. *Biol. Pr., Bratislava, vyd. SAV*, 11/5: 5–45.
Zajacová, V. 1961. *Lotus corniculatus L. - so zvlástnym zameraním na okolie Bratislavy*. Dipl. Pr., (msc.) depon in Kat. botan. PrFUK, Bratislava, SK.
Zinöcker, M. 1992. *Vegetationskundliche Untersuchungen im "Weingarten Lassee" (Marchfeld) zur Erstellung eines Landschaftsentwicklungskonzeptes und Pflegeplanes*. Diplomarbeit, Univ. Wien, AT.
Zlatník, A. 1928. Études écologiques et sociologiques sur le Sesleria coerulea et le Seslerion calcariae en Tchécoslovaquie. *Rozpr. Král. Čes. Společ. Nauk, cl. math.-natur*., 8/1: 1–116.
Zlinská, J. 1987. *Lúčna a pasienková vegetácia Povazského Inovca*. KDP, ÚEBE SAV, odd. geobotaniky, Bratislava, SK.

Zlinská, J. 2000. Vegetácia Holubyho lesostepi pri Vinosadoch v Malých Karpatoch. *Acta Environm. Univ. Comen., Bratislava,* 10: 139–152.
Zlinská, J. 2004. Adonido-Brachypodietum pinnati (Libbert 1933) Krausch 1961 na Devínskej Kobyle. *Biosozologia* 2: 49–61.
Zolyomi, B. 1939. Felsenvegetationstudien in Siebenbürgen und im Banat. *Annales Musei Nationalis Hungarici pars Botanica* 32: 63–145.
